# A genetic element in the SARS-CoV-2 genome is shared with multiple insect species

**DOI:** 10.1099/jgv.0.001551

**Published:** 2021-01-11

**Authors:** Torstein Tengs, Charles F. Delwiche, Christine Monceyron Jonassen

**Affiliations:** ^1^​ Section of Molecular Toxicology, Department of Environmental Health, Norwegian Institute of Public Health, Norway; ^2^​ Cell Biology and Molecular Genetics, University of Maryland, College Park, MD 20742, USA; ^3^​ Centre for Laboratory Medicine, Østfold Hospital Trust, Norway

**Keywords:** s2m, horizontal gene transfer, invertebrates, positive-sense ssRNA viruses, COVID-19, coronaviruses

## Abstract

SARS-CoV-2 is a member of the subgenus Sarbecovirus and thus contains the genetic element s2m. We have extensively mined nucleotide data in GenBank in order to obtain a comprehensive list of s2m sequences both in the four virus families where s2m has previously been described and in other groups of organisms. Surprisingly, there seems to be a xenologue of s2m in a large number of insect species. The function of s2m is unknown, but our data show a very high degree of sequence conservation both in insects and in viruses and that the version of s2m found in SARS-CoV-2 has unique features, not seen in any other virus or insect strains.

ssRNA viruses, such as coronaviruses, are known to have genomes with strong secondary structural features, such as stem-loop regions and pseudoknots. We have previously reported the presence of a 41–43 nt long hairpin-forming element, referred to as stem-loop II-like motif (s2m) [1], in several families of positive-sense ssRNA [(+)ssRNA] viruses [2]. The molecular structure has been mapped in great detail for severe acute respiratory syndrome coronavirus (SARS-CoV) [3]. Its sequence and secondary structure are highly conserved despite relatively high overall mutation rates. The phylogenetic distribution among viral genomes is patchy, and the function of s2m remains unknown, but the element is always present near the 3′ end of the genome, and in all virus families where s2m has been reported, there are examples of species carrying two (non-identical) back-to-back copies [2, 4].

Severe acute respiratory syndrome coronavirus 2 (SARS-CoV-2) [5] is a member of the SARS-related subgenus Sarbecovirus [6] and this group of coronaviruses is known to contain s2m [7]. The presence of s2m in the SARS-CoV-2 genome (GenBank accession MN908947, position 29 727–29 768; Figs 1 and 2) and other members of this group is probably the result of a single horizontal transfer event, pre-dating the divergence of SARS-related viruses [2, 7]. To further characterize the specific s2m sequence found in SARS-CoV-2, blastn [8] with word size 11 was used to search the entire virus section of GenBank using all s2m sequences reported in the literature (*n*=97) [1–3, 7] as query sequences. A total of 5553 s2m-containing accessions were identified with Expect values (E values)<0.2, representing at least four virus families (Table S1, available in the online version of this article). As expected, there was significant bias towards SARS-CoV-2, with the great majority of the s2m accessions stemming from this strain (3984/5553; 72 %). Notably, s2m found in SARS-CoV-2 contains a G to U transversion in position 31 (Fig. 2) that is consistent in all available SARS-CoV-2 accessions. This guanine is perfectly conserved outside of the SARS-CoV-2 sequences, with 100 % of the other s2m sequences having a G in this position (Table S1). As SARS-CoV-2 seems to be embedded within the Sarbecoviruses, it is likely that the unique G to U mutation has occurred specifically during the evolution of the current pandemic strain of SARS-CoV (Fig. 2). An Australian isolate with a 10 base deletion in s2m was also discovered (accession MT007544). Intriguingly, the deletion occurred after passaging isolates in Vero cells [9]. This could represent an attenuated SARS-CoV-2 strain, as culturing in permissive cell lines may have altered the selective pressure and alleviated the need to maintain a functional version of the motif. In addition to this, other Australian isolates have been shown to have a G to U transversion in residue 15, and it has been suggested that this might be indicative of a recombination event [10].

**Fig. 1.**
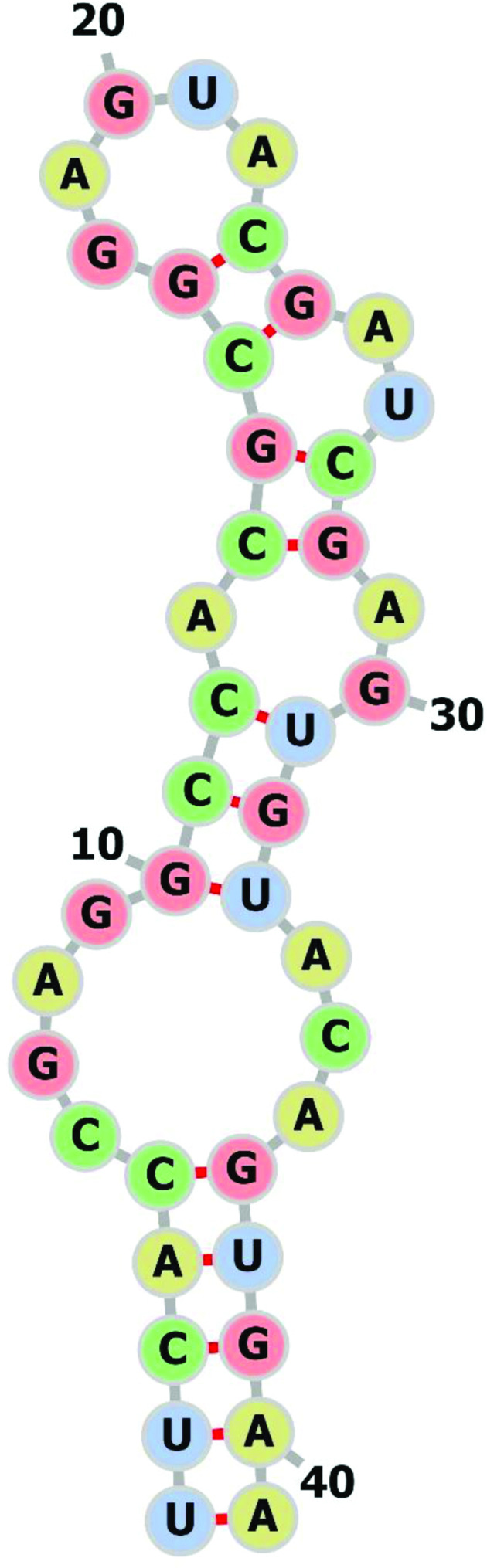
Secondary structure of s2m in SARS-CoV-2 based on the SARS-CoV model [3].

**Fig. 2.**
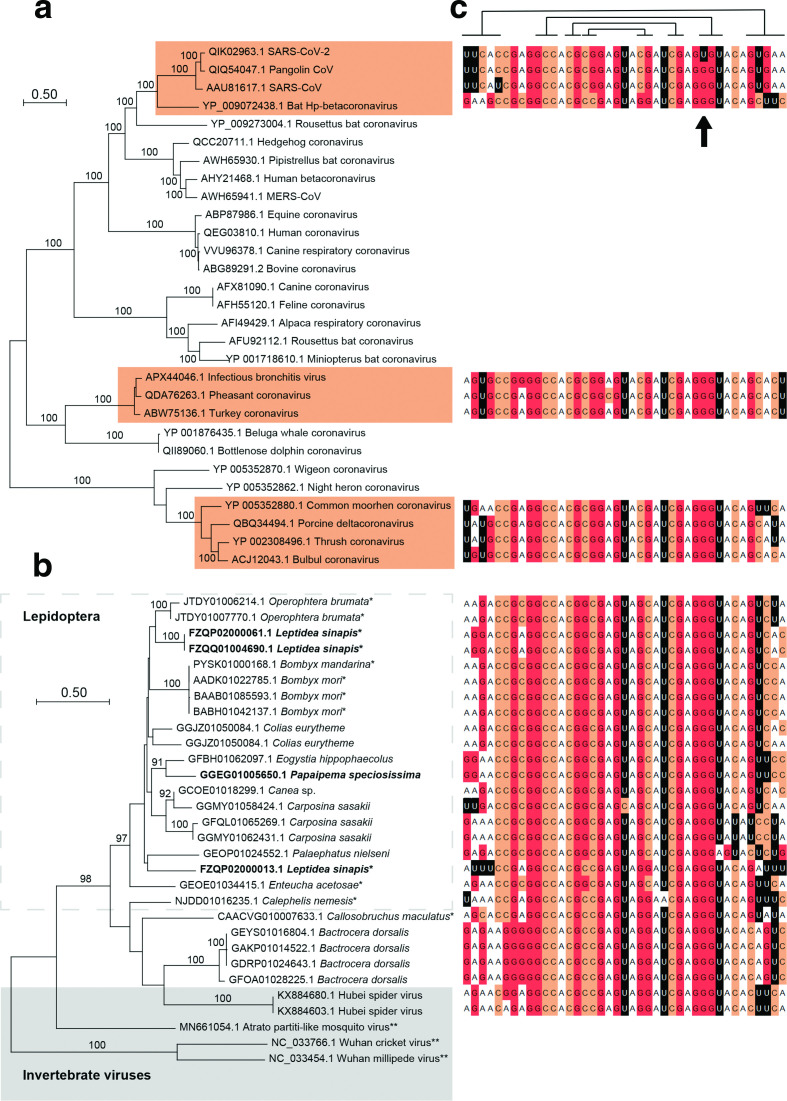
s2m in insects, spider-associated viruses and coronaviruses. (a) A maximum-likelihood phylogenetic analysis was performed on ORF1ab polyprotein sequences from selected coronavirus species using mega x [16]. Sequences were aligned using the clustal w algorithm [17], and the James–Taylor–Thornton (JTT) substitution model was used with gamma distribution (five categories) and invariable sites. Branch swapping was done using the subtree–pruning–regrafting method with ‘very strong’ filter. s2m-containing accessions have been highlighted and bootstrap values >90% are indicated (100 psedoreplicates). (b) Data from the s2m-associated hypothetical protein (see main text for details) were aligned and analysed as described in (a). Sequences in bold type stem from reading frames with (multiple) internal stop codons. *Genomic data, **accessions without s2m. (c) s2m sequences corresponding to operational taxonomic units in the phylogenetic trees. Lines above alignment show (non-Watson–Crick) base pairing residues [3], and the position with the unique G to U mutation in SARS-CoV-2 is indicated (arrow).

Also important is that two of the s2m virus accessions identified were from a recently published RNA-based invertebrate virosphere project [11]. These two highly similar sequences, derived from the spider species *Tetragnatha maxillosa*, encode a 447 aa long hypothetical protein immediately upstream of s2m that could not readily be identified using protein sequence similarity searches. This protein does not appear to be present in SARS-CoV-2 or other coronaviruses. Protein–protein blast (blastp) searches against the non-redundant (nr) GenBank protein database revealed the best matching putative homologue to be from winter moth (*Operophtera brumata*; E value 1×10^−81^), followed by accessions from the virosphere project referenced above. In the *O. brumata* genome sequence, an s2m motif could be identified immediately downstream from the stop codon of the 331 aa long uncharacterized protein (Fig. 2, Table S2). To explore the occurrence of this protein among other insects, the ORF sequence was used as a query in a tblastn search against the insect section of the GenBank Transcriptome Shotgun Assembly (TSA) database. A total of 92 protein sequences could be identified where >200 amino acids could reliably be aligned with the *O. brumata* accession. A phylogenetic analysis of these sequences revealed a complex tree, probably containing both orthologues and paralogues. Some of the transcriptome sequences may have incomplete coverage and thus lack s2m motifs due to missing data, but the *O. brumata* accession and the two spider virus accessions clustered within a well-supported group comprising primarily lepidopteran species (Fig. S1).

The primary stem region of s2m is 5 nt long (Fig. 1) and has been shown to be highly variable, but with residues generally supporting the secondary structure [1]. All virus s2m sequences found (above) were tabulated and the first and last five residues were removed. These 31 nt s2m core sequences were used to check for the presence of s2m motifs in insects [TSA and whole genome shotgun contigs (wgs) databases] using megablast [8] with word size 28. A total of 139 putative s2m-containing insect contigs were identified with at least one perfect 28-bp match, corresponding to E values of 0.001 (TSA) and 0.0002 (wgs), and representing >50 species (Table S2). The number of s2m motifs per accession ranged from one to five (Table S2). To assess if this approach would lead to a large number of random hits due the short query sequences used, the exact same search strategy was used to search the entire Nucleotide collection (nt) section of GenBank (excluding SARS-CoV-2). This search gave a total of 1528 hits; two were from the insect species *Carposina sasakii* and 1526 were from (+)ssRNA viruses. Correlating the insect s2m findings with the tree topology based on the hypothetical *O. brumata* protein, 21/24 of the insect accessions in the *O. brumata* cluster were found to stem from s2m-carrying insect genera (Fig. S2). Only one genus outside this cluster had a putative s2m motif in our initial megablast search, represented by a single species (*Mesovelia mulsanti*). Conversely, proteins encoded by the s2m-containing contigs could reliably be matched with the *O. brumata* protein for 63 (45 %) of the accessions (Table S2).

Focusing on s2m accessions, a phylogenetic analysis was performed using the longest and most similar amino acid sequences obtained from the TSA, wgs and nr databases (Table S2). The resulting topology was biased towards lepidopteran species (Fig. 2b). Whether this represents a bias in the available data or is the result of mutational processes is unclear. There also appears to be a few species where the base pairing that maintains the longest stem-forming region of s2m is not well supported, even when including the non-Watson–Crick base pairing G:U (see, for instance, *Bactrocera dorsalis*; Fig. 2c). In spite of this, the great majority of the 64 unique, putative s2m motifs identified seemed to have complementary RNA residues (GC/CG or UA/AU) in this stem-forming region (Fig. 1; residues 1–5 and 37–41), with the nucleotide positions closest to the most basal loop region showing the highest frequency of Watson–Crick base pairing potential (Fig. S2). Interestingly, translation of both genomic and transcriptomic data in some cases gave reading frames containing several internal stop codons, but amino acid sequences that could still be aligned to (near) full length (Fig. 2b, Table S2). Assuming the sequences are accurate, possible explanations would include mRNA editing or an alternative genetic code.

In the four virus families where s2m has previously been described, the flanking protein is easily recognizable. Looking at the list of blastp hits for the *O. brumata* protein, the best scoring accession that had been specifically annotated seemed to be a capsid protein from the Nilaparvata lugens commensal X virus [12]. The overall sequence similarity was low (273 aa alignment with 32 % identity, 45 % similarity and 8 % gaps), but a low E value (4×10^−21^) suggests that these sequences might be evolutionarily related. An interesting possibility is that this protein is an endogenous viral element (EVE) [13] that functions as a restriction factor and is part of the host’s defence against exogenous viruses [14].

We were unable to identify any signature of a retroviral origin for the s2m locus in any of the insect genome accessions, and there were no other indications of any of the insect s2m contigs being of a viral origin. In one of the genomic contigs (GenBank accession JTDY01007770.1), the ORF covering the uncharacterized protein also appeared to contain an intron, generally considered a hallmark of eukaryotic genes. In addition, PCR and Sanger sequencing was performed to confirm the presence of s2m and the upstream ORF in the *O. brumata* genome (Fig. S3), making it very unlikely that the downloaded sequence data stem from (RNA) viruses and do not represent *bona fide* insect sequences.

The insect species that contain s2m (and the associated protein) are distantly related, indicating either a deep evolutionary origin with multiple losses or that this genetic construct has been endogenized multiple times, perhaps with viruses as vectors [15]. The *T. maxillosa*-associated virus could represent such a vector, albeit no s2m sequences or proteins similar to the *O. brumata* protein could be found in any arachnid species using sequence similarity searches. The *T. maxillosa* sequence data were generated using crude tissue samples and annotated using bioinformatics, so it is possible that these accessions have been incorrectly identified, and that the sequences actually stem from viruses infecting insects that have in turn been ingested by the spider, or perhaps even the spider’s own transcriptome.

We believe that the most likely mode of transfer for s2m in viruses is through template switching between non-homologous RNA molecules, but the exact evolutionary link between the xenologues of s2m and the unknown protein found in insects and viruses cannot be established based on our data. s2m found in insects and viruses has similar primary sequence profiles, albeit there appear to be some subtle differences (Fig. 3). Future studies might elucidate the function of s2m, and this could perhaps allow functional screening to be performed using s2m sequences obtained from insects. In addition to carefully annotated and assembled sequence data from more insect and invertebrate virus taxa, this could help in tracing the evolutionary link of s2m from viruses to cellular organism. Outside the astroviruses, SARS-CoV and SARS-CoV-2 represent the only known examples of s2m-carrying viruses that infect humans, but it seems probable that s2m is still evolutionarily active and that this element will continue to affect the evolution of (+)ssRNA viruses.

**Fig. 3.**
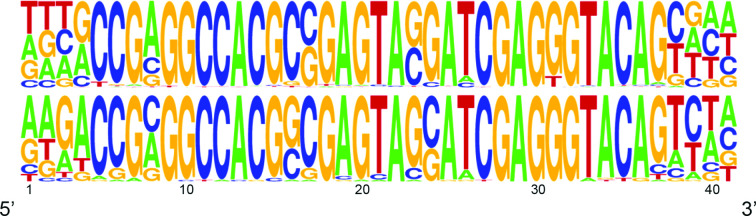
DNA logo frequency plot for s2m found in viruses (top panel) and insects (bottom panel). The plot was generated using WebLogo [18].

## Supplementary Data

Supplementary material 1
