# Supplementary material for: A genetic element in the SARS-CoV-2 genome is shared with multiple insect species
Source: J Gen Virol. 2021 Jan 11;102(3):001551. doi: 10.1099/jgv.0.001551 (PMC8515862; doi:10.1099/jgv.0.001551)
Supplement: Supplementary material 1 [file jgv-102-1551-s001.pdf]

Supplementary figure 1. Phylogenetic tree based the hypothetical protein found in *Operophtera brumata*, related insect species and invertebrate viruses. Identical sequences from the same species were removed and a maximum likelihood analysis was performed using MEGA X (1) after aligning amino acid sequences using the Clustal W algorithm (2). The James-Taylor-Thornton (JTT) substitution model was used with gamma distribution (5 categories) and invariable sites. Branch swapping was done using the subtree-pruning-regrafting method with 'very strong' filter. s2m-containing accessions have been indicated (red: insect species, blue: invertebrate viruses) and bootstrap values > 90 % are shown (100 psedoreplicates). * - A closely related member of the *Palaephatus* genus was show to contain s2m (*Palaephatus nielseni*; Supplementary table 2).

1. Kumar S, Stecher G, Li M, Knyaz C, Tamura K. 2018. MEGA X: Molecular Evolutionary Genetics Analysis across Computing Platforms. Mol Biol Evol 35:1547-1549.
2. Thompson JD, Higgins DG, Gibson TJ. 1994. CLUSTAL W: improving the sensitivity of progressive multiple sequence alignment through sequence weighting, position-specific gap penalties and weight matrix choice. Nucleic Acids Res 22:4673-80.

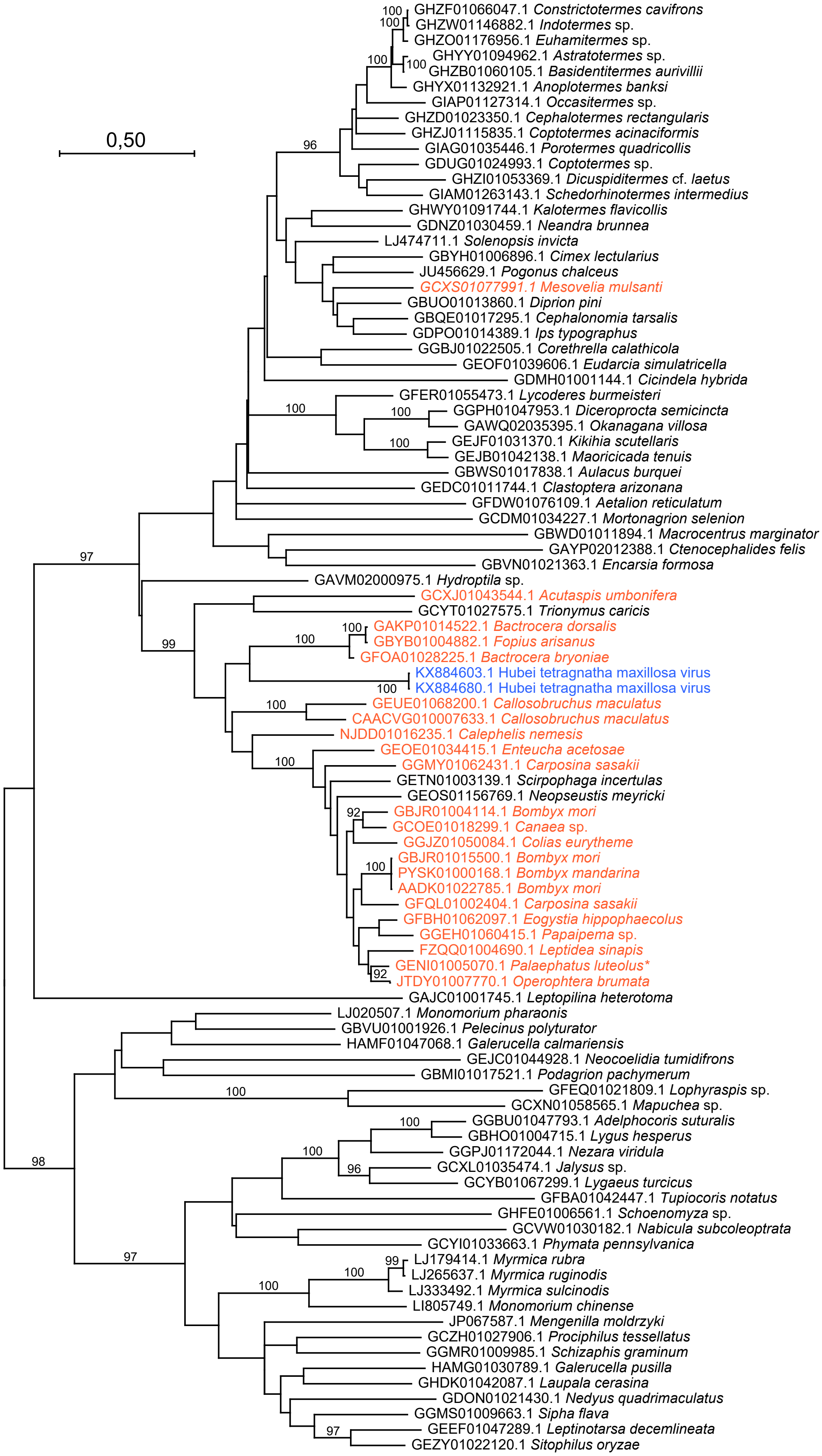
0,50
GHZF01066047.1 Constrictotermes cavifrons
GHZW01146882.1 Indotermes sp.
GHZO01176956.1 Euhamitermes sp.
GHYY01094962.1 Astratotermes sp.
GHZB01060105.1 Basidentitermes aurivillii
GHYX01132921.1 Anoplotermes banksi
GIAP01127314.1 Occasitermes sp.
GHZD01023350.1 Cephalotermes rectangularis
GHZJ01115835.1 Coptotermes acinaciformis
GIAG01035446.1 Porotermes quadricollis
GDUG01024993.1 Coptotermes sp.
GHZI01053369.1 Dicuspiditermes cf. laetus
GIAM01263143.1 Schedorhinotermes intermedius
GHWY01091744.1 Kalotermes flavicollis
GDNZ01030459.1 Neandra brunnea
LJ474711.1 Solenopsis invicta
GBYH01006896.1 Cimex lectularius
JU456629.1 Pogonus chalceus
GCXS01077991.1 Mesovelia mulsanti
GBUO01013860.1 Diprion pini
GBQE01017295.1 Cephalonomia tarsalis
GDPO01014389.1 Ips typographus
GGBJ01022505.1 Corethrella calathicola
GEOF01039606.1 Eudarcia simulatricella
GDMH01001144.1 Cicindela hybrida
GFER01055473.1 Lycoderes burmeisteri
GGPH01047953.1 Diceroprocta semicincta
GAWQ02035395.1 Okanagana villosa
GEJF01031370.1 Kikihia scutellaris
GEJB01042138.1 Maoricicada tenuis
GBWS01017838.1 Aulacus burquei
GEDC01011744.1 Clastoptera arizonana
GFDW01076109.1 Aetalion reticulatum
GCDM01034227.1 Mortonagrion selenion
GBWD01011894.1 Macrocentrus marginator
GAYP02012388.1 Ctenocephalides felis
GBVN01021363.1 Encarsia formosa
GAVM02000975.1 Hydroptila sp.
GCXJ01043544.1 Acutaspis umbonifera
GCYT01027575.1 Trionymus caricis
GAKP01014522.1 Bactrocera dorsalis
GBYB01004882.1 Fopius arisanus
GFOA01028225.1 Bactrocera bryoniae
KX884603.1 Hubei tetragnatha maxillosa virus
KX884680.1 Hubei tetragnatha maxillosa virus
GEUE01068200.1 Callosobruchus maculatus
CAACVG010007633.1 Callosobruchus maculatus
NJDD01016235.1 Calephelis nemesis
GEOE01034415.1 Enteucha acetosae
GGMY01062431.1 Carposina sasakii
GETN01003139.1 Scirpophaga incertulas
GEOS01156769.1 Neopseustis meyricki
GBJR01004114.1 Bombyx mori
GCOE01018299.1 Canaea sp.
GGJZ01050084.1 Colias eurytheme
GBJR01015500.1 Bombyx mori
PYSK01000168.1 Bombyx mandarina
AADK01022785.1 Bombyx mori
GFQL01002404.1 Carposina sasakii
GFBH01062097.1 Eogystia hippophaecolus
GGEH01060415.1 Papaipema sp.
FZQQ01004690.1 Leptidea sinapis
GENI01005070.1 Palaephatus luteolus*
JTDY01007770.1 Operophtera brumata
GAJC01001745.1 Leptopilina heterotoma
LJ020507.1 Monomorium pharaonis
GBVU01001926.1 Pelecinus polyturator
HAMF01047068.1 Galerucella calmariensis
GEJC01044928.1 Neocoelidia tumidifrons
GBMI01017521.1 Podagrion pachymerum
GFEQ01021809.1 Lophyraspis sp.
GCXN01058565.1 Mapuchea sp.
GGBU01047793.1 Adelphocoris suturalis
GBHO01004715.1 Lygus hesperus
GGPJ01172044.1 Nezara viridula
GCXL01035474.1 Jalysus sp.
GCYB01067299.1 Lygaeus turcicus
GFBA01042447.1 Tupiocoris notatus
GHFE01006561.1 Schoenomyza sp.
GCVW01030182.1 Nabicula subcoleoptrata
GCYI01033663.1 Phymata pennsylvanica
LJ179414.1 Myrmica rubra
LJ265637.1 Myrmica ruginodis
LJ333492.1 Myrmica sulcinodis
LI805749.1 Monomorium chinense
JP067587.1 Mengenilla moldrzyki
GCZH01027906.1 Prociphilus tessellatus
GGMR01009985.1 Schizaphis graminum
HAMG01030789.1 Galerucella pusilla
GHDK01042087.1 Laupala cerasina
GDON01021430.1 Nedyus quadrimaculatus
GGMS01009663.1 Sipha flava
GEEF01047289.1 Leptinotarsa decemlineata
GEZY01022120.1 Sitophilus oryzae

Supplementary figure 2. Percent (base pairing) residues in the primary stem region of s2m sequences from insects and viruses. Sequence information from the first and last five nucleotide positions of the s2m candidate sequences was analyzed. Residues 1/41 correspond to the most basal part of s2m (Figure 1) whereas 5/37 corresponds to the (base paring) residues closest to the most basal s2m loop-region. For simplicity, numbers from nucleotide combinations have been combined, as well as reverse complement nucleotide pairs (for instance, UG = GU + UG).

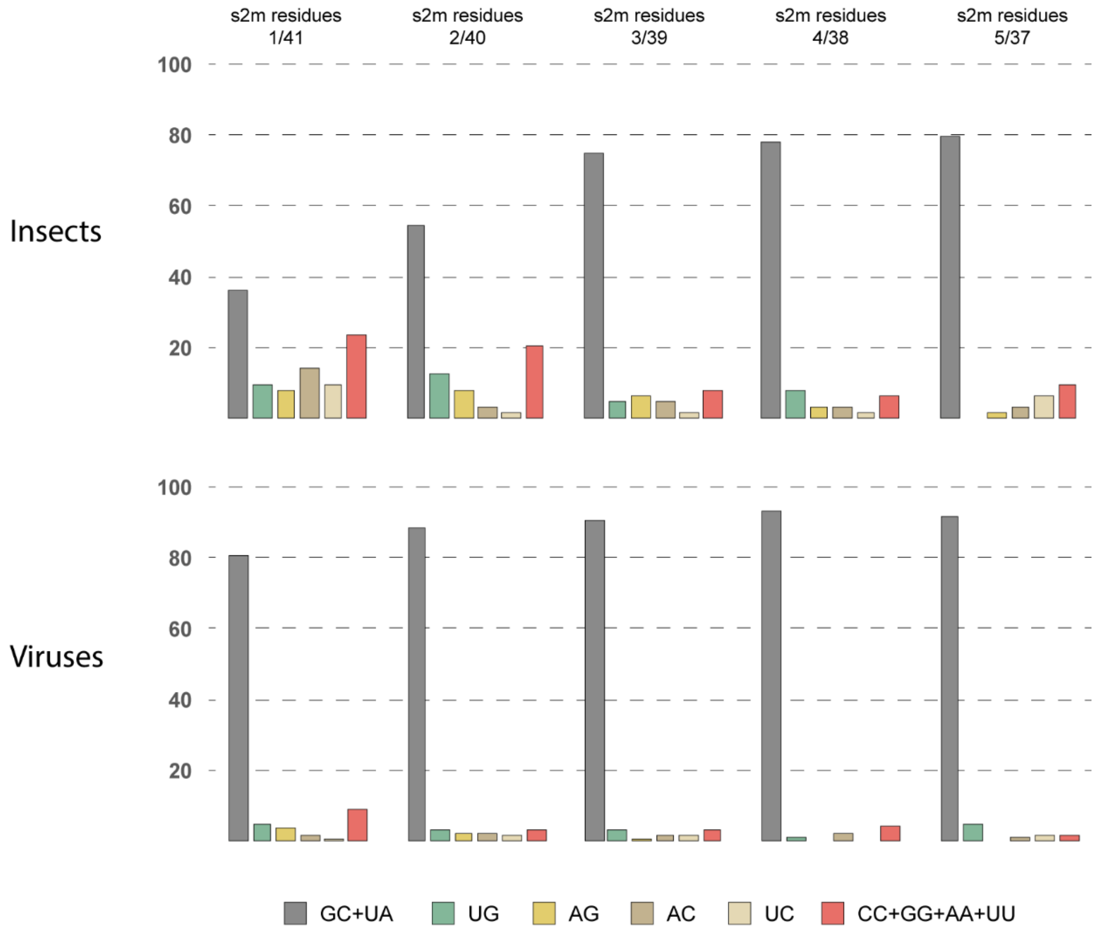

Supplementary figure 3. PCR amplification and Sanger sequencing of the s2m locus in the *Operophtera brumata* genome. PCR was performed using the AmpliTaq Gold 360 PCR Master Mix (Thermo Fisher Scientific, Oslo, Norway) and amplification products (lane B) were visualized on a QIAxcel Advanced System (Qiagen, Venlo, Netherlands), using the QX 100 bp-2.5 kb DNA Size marker (Qiagen; lane A). Primer sequences, ORF stop codon (TAA) and s2m sequence have been underlined.

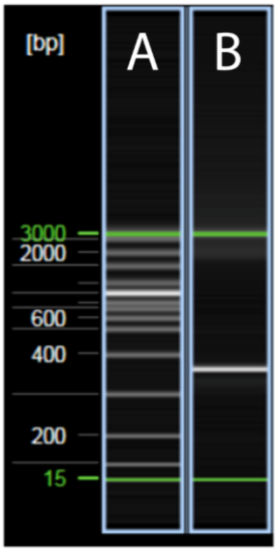

TTTCGTCTCCATTTCCGATTCCCAAAGGAA
CAGAACAAACGCAATTGTGCGTCGGATGAT
TATCAGCCAACGATAATCAGAACGAATCAA
GACCGCGGCCACGGCGAGTAGCATCGAGGG
TACAGTCTAGAACCGTTCATTTACTAATTA
ACATTAATTAATTAATTTAAGTTTCCATTG
TATTTAAAATAAACTTAGCATAGGGGAGCA
TTAGTCCGGTCGACACGCATCGTCATTTTA
CTCAGTTGTCATAAGTCCTAAACATTTGGT
TACAAAATGCAGTGCGGAGTGTGTGGAGCT
AATTTTAATGATGGGGTTCAGTGCAGTACC
TGTAGCAAACACCTCGATTTTGGT

Supplementary table 1. s2m motifs found in virus accessions using BLASTN (nt database).

GenBank fasta defline	s2m sequence
AB005257.1 Human astrovirus type 6 RNA for capsid protein, partial cds	GAAGCCGCGGCCACGCCGAGTAGGATCGAGGGTACAGCTTC
AB013618.2 Mamastrovirus 1 gene for capsid protein precursor, complete cds	GAAGCCGCGGCCACGCCGAGTAGGATCGAGGGTACAGCTTC
AB031030.1 Mamastrovirus 1 gene for capsid protein, complete cds, strain:katano24	GAAGCCGCGGCCACGCCGAGTAGGATCGAGGGTACAGCTTC
AB031031.1 Mamastrovirus 1 gene for capsid protein, complete cds, strain:Katano23-6	GAAGCCGCGGCCACGCCGAGTAGGATCGAGGGTACAGCTTC
AB033998.1 Avian nephritis virus 1 genes for structural polyprotein, non-structural polyprotein, complete cds	TTTCCCGAGGCCACGGCGAGTAGCATCGAGGGTACAGGAAA
AB037272.1 Mamastrovirus 3 gene for capsid protein precursor, complete cds	AAAGCCGAGGCCACGCCGAGTAGGAACGAGGGTACAGCTTT
AB037273.1 Human astrovirus 5 gene for capsid protein precursor, complete cds, strain: CHN146	GAAGCCGCGGCCACGCCGAGTAGGATCGAGGGTACAGCTTC
AB037274.1 Human astrovirus 5 gene for capsid protein precursor, complete cds, strain: CHN198	GAAGCCGCGGCCACGCCGAGTAGGATCGAGGGTACAGCTTC
AB046864.1 Avian nephritis virus 2 ORF 2 gene for virus capsid polyprotein, complete cds	TTTCCCGAGGCCACGGCGAGTAGCATCGAGGGTACAGGAAT
AB257344.1 SARS coronavirus Frankfurt 1 genomic RNA, nearly complete genome, clone: persistent virus #21	TTCATCGAGGCCACGCGGAGTACGATCGAGGGTACAGTGAA
AB496913.1 Human astrovirus 4 gene for capsid protein, complete cds	GGAGCCGCGGCCACGCCGAGTAGGATCGAGGGTACAGCTCC
AF056197.1 Feline astrovirus capsid precursor protein mRNA, complete cds	AGAGCCGAGGCCACGCCGAGTAGGATCGAGGGTACAGCTCT
AF111995.1 Turkey coronavirus strain Indiana nucleocapsid protein gene, complete cds	AGTGCCGGGGCCACGCGGCGTACGATCGTGGGTACAGCACT
AF111996.1 Turkey coronavirus strain Minnesota nucleocapsid protein gene, complete cds	AGTGCCGAGGCCACGCGGAGTACGATCGAGGGTACAGCACT
AF111997.1 Turkey coronavirus strain NC95 nucleocapsid protein gene, complete cds	AGTGCCGGGGCCACGCGGAGTACGATCGAGGGTACAGCACA
AF117209.1 Human astrovirus type 3 capsid protein precursor, gene, complete cds	GAGACCGCGGCCACGCCGAGTAGGATCGAGGGTACAGTCTC
AF141381.1 Human astrovirus putative serine protease gene, complete cds; putative RNA-dependent RNA polymeras	GAGACCGCGGCCACGCCGAGTAGGATCGAGGGTACAGTCTC
AF203002.1 Avian infectious bronchitis virus strain DE072 3' noncoding region	AGTGCCGAGGCCACGCGGAGTACGATCGAGGGTACAGCACT
AF203007.1 Avian infectious bronchitis virus strain D1466 3' noncoding region	AGTGCCGGGGCCACGCGGAGTACGATCGAGGGTACAGCACT
AF248738.2 Human astrovirus type 7 ORF1b gene, partial cds; and capsid protein precursor, gene, complete cds	GAGACCGCGGCCACGCCGAGTAGGATCGAGGGTACAGTCTC
AF257226.1 Human astrovirus type 2 M358 capsid precursor protein gene, partial cds	GAGACCGCGGCCACGCCGAGTAGGATCGAGGGTACAGTCTC
AF260508.1 Human astrovirus type 8, complete genome	GAAGCCGCGACCACGCCGAGTAGGATCGAGGGTACAGCTTC
AF322368.2 Avian infectious bronchitis virus strain D41 3' noncoding region	AGTGCCGGGGCCACGCGGAGTACGATCGAGGGTACAGCACT
AF361253.1 Equine rhinovirus 3 strain P313/75, complete genome	CTCGCCGAGGCCACGCCGAGTAGGACCGAGGGTACAGCGAG
AF395733.1 Human astrovirus type 1 strain HUN-1b capsid protein precursor, gene, partial cds	GAAGCCGCGGCCACGCCGAGTAGGATCGAGGGTACAGCTTC
AF395734.1 Human astrovirus type 1 strain HUN-1a capsid protein precursor, gene, partial cds	GAAGCCGCGGCCACGCCGAGTAGGAACGAGGGTACAGCTTC
AF395735.1 Human astrovirus type 3 strain HUN-3 capsid protein precursor, gene, partial cds	GAGACCGCGGCCACGCCGAGTAGGATCGAGGGTACAGTCTC
AF395737.1 Human astrovirus type 5 strain HUN-5 capsid protein precursor, gene, partial cds	GAAGCCGCGGCCACGCCGAGTAGGATCGAGGGTACAGCTTC
AF395738.1 Human astrovirus type 8 strain HUN-8 capsid protein precursor, gene, partial cds	GAAGCCGCGGCCACGCCGAGTAGGATCGAGGGTACAGCTTC
AH012999.2 SARS coronavirus Shanhgai LY polyprotein gene, partial cds, alternatively spliced; orf1ab polyprotein, orf	TTCATCGAGGCCACGCGGAGTACGATCGAGGGTACAGTGAA
AH013708.2 SARS coronavirus Sin0409, partial sequence	TTCATCGAGGCCACGCGGAGTACGATCGAGGGTACAGTGAA

AH013709.2 SARS coronavirus Sin_WNV, partial sequence	TTCATCGAGGCCACGCGGAGTACGATCGAGGGTACAGTGAA
AJ278335.1 Avian infectious bronchitis virus, strain D207, 3' UTR	AGTGCCAGGGCCACGCGGAGTACGATCGAGGGTACAGCACT
AJ278336.1 Avian infectious bronchitis virus, strain H120, 3' UTR	AGTGCCGGGGCCACGCGGAGTACGATCGAGGGTACAGCACT
AJ278337.1 Avian infectious bronchitis virus, strain HV10, 3' UTR	AGTGCCGGGGCCACGCGGAGTACGATCGAGGGTACAGCACT
AJ278338.1 Avian infectious bronchitis virus, strain HVI-140, 3' UTR	AGTGCCGGGGCCACGCGGAGTACGATCGAGGGTACAGCACT
AJ310642.1 Turkey coronavirus genomic RNA, partial 3' UTR, strain turkey/UK/412/00	AGTGCCGGGGCCACGCGGAGTACGATCGAGGGTACAGCACA
AJ311317.1 Avian infectious bronchitis virus (strain Beaudette CK) complete genomic RNA	AGTGCCGGGGCCACGCGGAGTACGATCGAGGGTACAGCACT
AJ311362.1 Avian infectious bronchitis virus (strain Beaudette US) s gene, 3a gene, 3b gene, 3c gene, m gene, 5a gene	AGTGCCGGGGCCACGCGGAGTACGATCGAGGGTACAGCACT
AJ619579.1 Pheasant coronavirus conserved 3' untranslated region, genomic RNA, isolate ph/UK/438/94	AGTGCCGAGGCCACGCGGCGTACGATCGAGGGTACAGCACT
AJ619580.1 Pheasant coronavirus conserved 3' untranslated region, genomic RNA, isolate ph/UK/602/95	AGTGCCGAGGCCACGCGGAGAACGATCGAGGGTACAGCACT
AJ619581.1 Pheasant coronavirus conserved 3' untranslated region, genomic RNA, isolate ph/UK/750/83	AGTGCCGAGGCCACGCGGCGTACGATCGAGGGTACAGCACT
AJ619582.1 Pheasant coronavirus conserved 3' untranslated region, genomic RNA, isolate ph/UK/1/99	AGTGCCGAGGCCACGCGGCGTACGATCGAGGGTACAGCACT
AJ619584.1 Pheasant coronavirus conserved 3' untranslated region, genomic RNA, isolate ph/UK/5/99	AGTGCCGAGGCCACGCGGAGTACGATCGAGGGTACAGCACT
AJ619586.1 Pheasant coronavirus conserved 3' untranslated region, genomic RNA, isolate ph/UK/8/99	AGTGCCGGGGCCACGCGGAGTACGATCGAGGGTACAGCACT
AJ619588.1 Pheasant coronavirus conserved 3' untranslated region, genomic RNA, isolate ph/UK/12/98	AGTGCCGAGGCCACGCGGCGTACGATCGAGGGTACAGCACT
AJ619590.1 Pheasant coronavirus conserved 3' untranslated region, genomic RNA, isolate ph/UK/14/99	AGTGCCGGGGCCACGCGGAGTACGATCGAGGGTACAGCACT
AJ619591.1 Pheasant coronavirus conserved 3' untranslated region, genomic RNA, isolate ph/UK/15/99	AGTGCCGAGGCCACGCGGCGTACGATCGAGGGTACAGCACT
AJ619592.1 Pheasant coronavirus conserved 3' untranslated region, genomic RNA, isolate ph/UK/17/99	AGTGCCGGGGCCACGCGGAGTACGACCGAGGGTACAGCACT
AJ619594.1 Pheasant coronavirus conserved 3' untranslated region, genomic RNA, isolate ph/UK/20/98	AGTGCCGAGGCCACGCGGGGTACGATCGAGGGTACAGCACT
AJ620751.1 Human astrovirus type 1 partial mRNA for capsid precursor protein (CP gene), isolate HUN-479	GAAGCCGCGGCCACGCCGAGTAGGAACGAGGGTACAGCTTC
AJ620752.1 Human astrovirus type 1 partial mRNA for capsid precursor protein (CP gene), isolate HUN-867	GAAGCCGCGGCCACGCCGAGTAGGAACGAGGGTACAGCTTC
AJ620753.1 Human astrovirus type 4 partial mRNA for capsid precursor protein (CP gene), isolate HUN-991	GGAGCCGCGGCCACGCCGAGTAGGATCGAGGGTACAGCTCC
AJ620754.1 Human astrovirus type 4 partial mRNA for capsid precursor protein (CP gene), isolate HUN-1065	GGAGCCGCGGCCACGCCGAGTAGGATCGAGGGTACAGCTCC
AJ620755.1 Human astrovirus type 1 partial mRNA for capsid precursor protein (CP gene), isolate HUN-1276	GAAGCCGCGGCCACGCCGAGTAGGAACGAGGGTACAGCTTC
AJ620756.1 Human astrovirus type 1 partial mRNA for capsid precursor protein (CP gene), isolate HUN-1284	GAAGCCGCGGCCACGCCGAGTAGGAACGAGGGTACAGCTTC
AJ620757.1 Human astrovirus type 8 partial mRNA for capsid precursor protein (CP gene), isolate HUN-1562	GAAGCCGCGGCCACGCCGAGTAGGATCGAGGGTACAGCTTC
AJ620758.1 Human astrovirus type 4 partial mRNA for capsid precursor protein (CP gene), isolate HUN-1760	GGAGCCGCGGCCACGCCGAGTAGGATCGAGGGTACAGCTCC
AJ620759.1 Human astrovirus type 1 partial mRNA for capsid precursor protein (CP gene), isolate HUN-1830	GAAGCCGCGGCCACGCCGAGTAGGAACGAGGGTACAGCTTC
AJ620760.1 Human astrovirus type 3 partial mRNA for capsid precursor protein (CP gene), isolate HUN-1854	GAGACCGCGGCCACGCCGAGTAGGATCGAGGGTACAGTCTC
AJ871017.1 Goose coronavirus NC gene, ORFxg and ORFyg, genomic RNA, isolate 03/586-50	TGTGCCGGGGCCACGCGGAGTACGATCGAGGGTACAGCACA
AJ871018.1 Goose coronavirus partial NC gene, ORFxg and ORFyg, genomic RNA, isolate 03/586-77	TATGCCGGGGCCACGCGGAGTACGATCGAGGGTACAGCATA
AJ871019.1 Goose coronavirus partial NC gene, ORFxg and ORFyg, genomic RNA, isolate 03/586-30	TGTGCCGGGGCCACGCGGAGTACGATCGAGGGTACAGCACA
AJ871020.1 Goose coronavirus partial NC gene, ORFxg and ORFyg, genomic RNA, isolate 03/586-86	TGTGCCGGGGCCACGCGGAGTACGATCGAGGGTACAGCACA

AJ871022.1 Pigeon coronavirus NC gene for putative nucleocapsid protein and ORFyp for hypothetical protein, genor	AATGCCGAGGCCACGCGGAGTACGATCGAGGGTACAGCATT
AJ871023.1 Pigeon coronavirus partial NC gene for putative nucleocapsid protein and ORFyp for hypothetical protein	AATGCCGAGGCCACGCGGAGTACGATCGAGGGTACAGCATT
AJ871024.1 Duck coronavirus partial ORFyd for hypothetical protein, genomic RNA, isolate 03/1094	AGTGCCGGGGCCACGCGGAGTACGATCGAGGGTACAGCACT
AP006557.1 SARS coronavirus TWH genomic RNA, complete genome	TTCATCGAGGCCACGCGGAGTACGATCGAGGGTACAGTGAA
AP006558.1 SARS coronavirus TWJ genomic RNA, complete genome	TTCATCGAGGCCACGCGGAGTACGATCGAGGGTACAGTGAA
AP006559.1 SARS coronavirus TWK genomic RNA, complete genome	TTCATCGAGGCCACGCGGAGTACGATCGAGGGTACAGTGAA
AP006560.1 SARS coronavirus TWS genomic RNA, complete genome	TTCATCGAGGCCACGCGGAGTACGATCGAGGGTACAGTGAA
AP006561.1 SARS coronavirus TWY genomic RNA, complete genome	TTCATCGAGGCCACGCGGAGTACGATCGAGGGTACAGTGAA
AY044186.1 Avian infectious bronchitis virus mRNA, 3'UTR	AGTGCCGGGGCCACGCTGAGTACGACCGAGGGTACAGCACT
AY179509.1 Mink astrovirus, complete genome	TACCCGAGGCCACGCCGAGTTAGGATCGAGGGTACAGGTAG
AY188183.1 Human astrovirus strain T1/EG/B16/2000 capsid protein precursor, gene, partial cds	GAAGCCGCGGCCACGCCGAGTAGGATCGAGGGTACAGCTTC
AY188184.1 Human astrovirus strain T1/EG/A49/2000 capsid protein precursor, gene, partial cds	GAAGCCGCGGCCACGCCGAGTAGGATCGAGGGTACAGCTTC
AY188185.1 Human astrovirus strain T1/EG/B43/2000 capsid protein precursor, gene, partial cds	GAAGCCGCGGCCACGCCGAGTAGGATCGAGGGTACAGCTTC
AY188186.1 Human astrovirus strain T2/EG/BHS0074/2001 capsid protein precursor, gene, partial cds	GAGACCGCGGCCACGCCGAGTAGGATCGAGGGTACAGTCTC
AY188187.1 Human astrovirus strain T2/EG/B199/2000 capsid protein precursor, gene, partial cds	GAGACCGCGGCCACGCCGAGTAGGAACGAGGGTACAGTCTC
AY188188.1 Human astrovirus strain T3/EG/B11/2000 capsid protein precursor, gene, partial cds	GAGACCGCGGCCACGCCGAGTAGGATCGAGGGTACAGTCTC
AY188189.1 Human astrovirus strain T3/EG/A37/2000 capsid protein precursor, gene, partial cds	GAGACCGCGGCCACGCCGAGTAGGATCGAGGGTACAGTCTC
AY188190.1 Human astrovirus strain T3/EG/B68/2000 capsid protein precursor, gene, partial cds	GAGACCGCGGCCACGCCGAGTAGGACCGAGGGTACAGTCTC
AY188191.1 Human astrovirus strain T3/EG/B33/2000 capsid protein precursor, gene, partial cds	GAGACCGCGGCCACGCGGAGTAGGATCGAGGGTACAGTCTC
AY188192.1 Human astrovirus strain T3/EG/B24/2000 capsid protein precursor, gene, partial cds	GAGACCGCGGCCACGCCGAGTAGGATCGAGGGTACAGTCTC
AY188193.1 Human astrovirus strain T3/EG/B9/2000 capsid protein precursor, gene, partial cds	GAGACCGCGGCCACGCCGAGTAGGATCGAGGGTACAGTCTC
AY188194.1 Human astrovirus strain T4/EG/A33/2000 capsid protein precursor, gene, partial cds	GGAGCCGCGGCCACGCCGAGTAGGATCGAGGGTACAGCTCC
AY188195.1 Human astrovirus strain T4/EG/A5/2000 capsid protein precursor, gene, partial cds	GGAGCCGCGGCCACGCCGAGTAGGATCGAGGGTACAGCTCC
AY188196.1 Human astrovirus strain T4/EG/A70/2000 capsid protein precursor, gene, partial cds	GGAGCCGCGGCCACGCCGAGTAGGATCGAGGGTACAGCTCC
AY188197.1 Human astrovirus strain T4/EG/A86/2000 capsid protein precursor, gene, partial cds	GGAGCCGCGGCCACGCCGAGTAGGATCGAGGGTACAGCTCC
AY188198.1 Human astrovirus strain T4/EG/B177/2000 capsid protein precursor, gene, partial cds	GGAGCCGCGGCCACGCCGAGTAGGATCGAGGGTACAGCTCC
AY188199.1 Human astrovirus strain T4/EG/B249/2001 capsid protein precursor, gene, partial cds	GGAGCCGCGGCCACGCCGAGTAGGATCGAGGGTACAGCTCC
AY188200.1 Human astrovirus strain T5/EG/A215/2001 capsid protein precursor, gene, partial cds	GAAGCCGCGGCCACGCCGAGTAGGATCGAGGGTACAGCTTC
AY188201.1 Human astrovirus strain T5/EG/A31/2000 capsid protein precursor, gene, partial cds	GAAGCCGCGGCCACGCCGAGTAGGATCGAGGGTACAGCTTC
AY188202.1 Human astrovirus strain T5/EG/A67/2000 capsid protein precursor, gene, partial cds	GAAGCCGCGGCCACGCCGAGTAGGATCGAGGGTACAGCTTC
AY188203.1 Human astrovirus strain T6/EG/A30/2000 capsid protein precursor, gene, partial cds	GAAGCCGCGGCCACGCCGAGTAGGATCGAGGGTACAGCTTC
AY188204.1 Human astrovirus strain T8/EG/A226/2001 capsid protein precursor, gene, partial cds	GAAGCCGCGGCCACGCCGAGTAGGATCGAGGGTACAGCTTC
AY188205.1 Human astrovirus strain T8/EG/A64/2000 capsid protein precursor, gene, partial cds	GAAGCCGCGGCCACGCCGAGTAGGATCGAGGGTACAGCTTC

AY188206.1 Human astrovirus strain T8/EG/B189/2000 capsid protein precursor, gene, partial cds	GAAGCCGCGGCCACGCCGAGTAGGATCGAGGGTACAGCTTC
AY188207.1 Human astrovirus strain T8/EG/B230/2001 capsid protein precursor, gene, partial cds	GAAGCCGCGGCCACGCCGAGTAGGATCGAGGGTACAGCTTC
AY188208.1 Human astrovirus strain T8/EG/B275/2001 capsid protein precursor, gene, partial cds	GAAGCCGCGGCCACGCCGAGTAGGATCGAGGGTACAGCTTC
AY274119.3 SARS coronavirus Tor2, complete genome	TTCATCGAGGCCACGCGGAGTACGATCGAGGGTACAGTGAA
AY278487.3 SARS coronavirus BJ02, complete genome	TTCATCGAGGCCACGCGGAGTACGATCGAGGGTACAGTGAA
AY278488.2 SARS coronavirus BJ01, complete genome	TTCATCGAGGCCACGCGGAGTACGATCGAGGGTACAGTGAA
AY278489.2 SARS coronavirus GD01, complete genome	TTCATCGAGGCCACGCGGAGTACGATCGAGGGTACAGTGAA
AY278490.3 SARS coronavirus BJ03, complete genome	TTCATCGAGGCCACGCGGAGTACGATCGAGGGTACAGTGAA
AY278491.2 SARS coronavirus HKU-39849, complete genome	TTCATCGAGGCCACGCGGAGTACGATCGAGGGTACAGTGAA
AY278554.2 SARS coronavirus CUHK-W1, complete genome	TTCATCGAGGCCACGCGGAGTACGATCGAGGGTACAGTGAA
AY278741.1 SARS coronavirus Urbani, complete genome	TTCATCGAGGCCACGCGGAGTACGATCGAGGGTACAGTGAA
AY279354.2 SARS coronavirus BJ04, complete genome	TTCATCGAGGCCACGCGGAGTACGATCGAGGGTACAGTGAA
AY282752.2 SARS coronavirus CUHK-Su10, complete genome	TTCATCGAGGCCACGCGGAGTACGATCGAGGGTACAGTGAA
AY283794.1 SARS coronavirus Sin2500, complete genome	TTCATCGAGGCCACGCGGAGTACGATCGAGGGTACAGTGAA
AY283795.1 SARS coronavirus Sin2677, complete genome	TTCATCGAGGCCACGCGGAGTACGATCGAGGGTACAGTGAA
AY283796.1 SARS coronavirus Sin2679, complete genome	TTCATCGAGGCCACGCGGAGTACGATCGAGGGTACAGTGAA
AY283797.1 SARS coronavirus Sin2748, complete genome	TTCATCGAGGCCACGCGGAGTACGATCGAGGGTACAGTGAA
AY283798.2 SARS coronavirus Sin2774, complete genome	TTCATCGAGGCCACGCGGAGTACGATCGAGGGTACAGTGAA
AY290752.2 SARS coronavirus ZJ01 isolate ZJ01b uncharacterized protein 6, uncharacterized protein 7a, uncharacteri	TTCATCGAGGCCACGCGGAGTACGATCGAGGGTACAGTGAA
AY291315.1 SARS coronavirus Frankfurt 1, complete genome	TTCATCGAGGCCACGCGGAGTACGATCGAGGGTACAGTGAA
AY291451.1 SARS coronavirus TW1, complete genome	TTCATCGAGGCCACGCGGAGTACGATCGAGGGTACAGTGAA
AY297028.1 SARS coronavirus ZJ01, complete genome	TTCATCGAGGCCACGCGGAGTACGATCGAGGGTACAGTGAA
AY304451.1 Human astrovirus type 1 T1/US/KL1417/1998 capsid protein precursor, gene, partial cds	GAAGCCGCGGCCACGCCGAGTAGGATCGAGGGTACAGCTTC
AY304452.1 Human astrovirus type 1 T1/US/KL1638/1999 nonfunctional capsid protein precursor, gene, partial seque	GAAGCCGCGGCCACGCCGAGTGGGAACGAGGGTACAGCTTC
AY304453.1 Human astrovirus type 1 T1/US/KL1900/1999 capsid protein precursor, gene, partial cds	GAAGCCGCGGCCACGCCGAGTAGGAACGAGGGTACAGCTTC
AY304454.1 Human astrovirus type 1 T1/US/KL1947/1999 capsid protein precursor, gene, partial cds	GAAGCCGCGGCCACGCCGAGTAGGAACGAGGGTACAGCTTC
AY304455.1 Human astrovirus type 1 T1/US/NR2501/1998 capsid protein precursor, gene, partial cds	GAAGCCGCGGCCACGCCGAGTAGGAACGAGGGTACAGCTTC
AY304456.1 Human astrovirus type 1 T1/US/CN2932/1998 capsid protein precursor, gene, partial cds	GAAGCCGCGGCCACGCCGAGTAGGAACGAGGGTACAGCTTC
AY304457.1 Human astrovirus type 1 T1/US/CN2882/1998 capsid protein precursor, gene, partial cds	GAAGCCGCGGCCACGCCGAGTAGGCACGAGGGTACAGCTTC
AY304458.1 Human astrovirus type 1 T1/US/CN2814/1998 capsid protein precursor, gene, partial cds	GAAGCCGCGGCCACGCCGAGTAGGATCGAGGGTACAGCTTC
AY304460.1 Human astrovirus type 1 T1/US/NR2311/1999 capsid protein precursor, gene, partial cds	GAAGCCGCGGCCACGCCGAGTAGGAACGAGGGTACAGCTTC
AY304461.1 Human astrovirus type 2 T2/US/KL1299/1998 capsid protein precursor, gene, partial cds	GAGACCGCGGCCACGCCGAGTAGGATCGAGGGTACAGTCTC
AY304462.1 Human astrovirus type 2 T2/US/CN2722/1998 capsid protein precursor, gene, partial cds	GAGACCGCGGCCACGCCGAGTAGGAACGAGGGTACAGTCTC

AY304463.1 Human astrovirus type 2 T2/US/CN3080/1998 capsid protein precursor, gene, partial cds	GAGACCGCGGCCACGCCGAGTAGGAACGAGGGTACAGTCTC
AY304464.1 Human astrovirus type 3 T3/US/CN3207/1999 capsid protein precursor, gene, partial cds	GAGACCGCGGCCACGCCGAGTAGGATCGAGGGTACAGTCTC
AY304465.1 Human astrovirus type 3 T3/US/KL754/1998 capsid protein precursor, gene, partial cds	GAGACCGCGGCCACGCCGAGTAGGATCGAGGGTACAGTCTC
AY304466.1 Human astrovirus type 4 T4/US/NR2657/1998 capsid protein precursor, gene, partial cds	GGAGCCGCGGCCACGCCGAGTAGGATCGAGGGTACAGCTCC
AY304467.1 Human astrovirus type 5 T5/US/KL1783/1999 capsid protein precursor, gene, partial cds	GAAGCCGCGGCCACGCCGAGTAGGATCGAGGGTACAGCTTC
AY304468.1 Human astrovirus type 7 T7/US/CN486/1999 capsid protein precursor, gene, partial cds	GAGACCGCGGCCACGCCGAGTAGGATCGAGGGTACAGTCTC
AY304469.1 Human astrovirus type 8 T8/US/KL1553/1998 capsid protein precursor, gene, partial cds	GAAGCCGCGGCCACGCCGAGTAGGATCGAGGGTACAGCTTC
AY304470.1 Human astrovirus type 8 T8/US/CN3383/1999 capsid protein precursor, gene, partial cds	GAAGCCGCGGCCACGCGGAGTAGGATCGAGGGTACAGCTTC
AY304486.1 SARS coronavirus SZ3, complete genome	TTCATCGAGGCCACGCGGAGTACGATCGAGGGTACAGTGAA
AY304487.1 SARS coronavirus SZ13, partial genome	TTCATCGAGGCCACGCGGAGTACGATCGAGGGTACAGTGAA
AY304488.1 SARS coronavirus SZ16, complete genome	TTCATCGAGGCCACGCGGAGTACGATCGAGGGTACAGTGAA
AY304490.1 SARS coronavirus GZ43, partial genome	TTCATCGAGGCCACGCGGAGTACGATCGAGGGTACAGTGAA
AY304491.1 SARS coronavirus GZ60, partial genome	TTCATCGAGGCCACGCGGAGTACGATCGAGGGTACAGTGAA
AY304492.1 SARS coronavirus HKU-36871, partial genome	TTCATCGAGGCCACGCGGAGTACGATCGAGGGTACAGTGAA
AY304493.1 SARS coronavirus HKU-65806, partial genome	TTCATCGAGGCCACGCGGAGTACGATCGAGGGTACAGTGAA
AY304494.1 SARS coronavirus HKU-66078, partial genome	TTCATCGAGGCCACGCGGAGTACGATCGAGGGTACAGTGAA
AY304495.1 SARS coronavirus GZ50, complete genome	TTCATCGAGGCCACGCGGAGTACGATCGAGGGTACAGTGAA
AY310120.1 SARS coronavirus FRA, complete genome	TTCATCGAGGCCACGCGGAGTACGATCGAGGGTACAGTGAA
AY313906.1 SARS coronavirus GD69, complete genome	TTCATCGAGGCCACGCGGAGTACGATCGAGGGTACAGTGAA
AY319651.1 Avian infectious bronchitis virus isolate BJ, complete genome	AGTGCCGGGGCCACGCGGAGTACGATCGAGGGTACAGCACT
AY321118.1 SARS coronavirus TWC, complete genome	TTCATCGAGGCCACGCGGAGTACGATCGAGGGTACAGTGAA
AY323977.2 SARS coronavirus HSR 1, complete genome	TTCATCGAGGCCACGCGGAGTACGATCGAGGGTACAGTGAA
AY338174.1 SARS coronavirus Taiwan TC1, complete genome	TTCATCGAGGCCACGCGGAGTACGATCGAGGGTACAGTGAA
AY338175.1 SARS coronavirus Taiwan TC2, complete genome	TTCATCGAGGCCACGCGGAGTACGATCGAGGGTACAGTGAA
AY345986.1 SARS coronavirus CUHK-AG01, complete genome	TTCATCGAGGCCACGCGGAGTACGATCGAGGGTACAGTGAA
AY345987.1 SARS coronavirus CUHK-AG02, complete genome	TTCATCGAGGCCACGCGGAGTACGATCGAGGGTACAGTGAA
AY345988.1 SARS coronavirus CUHK-AG03, complete genome	TTCATCGAGGCCACGCGGAGTACGATCGAGGGTACAGTGAA
AY348314.1 SARS coronavirus Taiwan TC3, complete genome	TTCATCGAGGCCACGCGGAGTACGATCGAGGGTACAGTGAA
AY350750.1 SARS coronavirus PUMC01, complete genome	TTCATCGAGGCCACGCGGAGTACGATCGAGGGTACAGTGAA
AY351680.1 SARS coronavirus ZMY 1, complete genome	TTCATCGAGGCCACGCGGAGTACGATCGAGGGTACAGTGAA
AY357075.1 SARS coronavirus PUMC02, complete genome	TTCATCGAGGCCACGCGGAGTACGATCGAGGGTACAGTGAA
AY357076.1 SARS coronavirus PUMC03, complete genome	TTCATCGAGGCCACGCGGAGTACGATCGAGGGTACAGTGAA
AY362698.1 SARS coronavirus TWC2, complete genome	TTCATCGAGGCCACGCGGAGTACGATCGAGGGTACAGTGAA

AY362699.1 SARS coronavirus TWC3, complete genome	TTCATCGAGGCCACGCGGAGTACGATCGAGGGTACAGTGAA
AY390556.1 SARS coronavirus GZ02, complete genome	TTCATCGAGGCCACGCGGAGTACGATCGAGGGTACAGTGAA
AY394850.2 SARS coronavirus WHU, complete genome	TTCATCGAGGCCACGCGGAGTACGATCGAGGGTACAGTGAA
AY394978.1 SARS coronavirus GZ-B, complete genome	TTCATCGAGGCCACGCGGAGTACGATCGAGGGTACAGTGAA
AY394979.1 SARS coronavirus GZ-C, complete genome	TTCATCGAGGCCACGCGGAGTACGATCGAGGGTACAGTGAA
AY394980.1 SARS coronavirus GZ-D, partial genome	TTCATCGAGGCCACGCGGAGTACGATCGAGGGTACAGTGAA
AY394981.1 SARS coronavirus HGZ8L1-A, partial genome	TTCATCGAGGCCACGCGGAGTACGATCGAGGGTACAGTGAA
AY394982.1 SARS coronavirus HGZ8L1-B, partial genome	TTCATCGAGGCCACGCGGAGTACGATCGAGGGTACAGTGAA
AY394983.1 SARS coronavirus HSZ2-A, complete genome	TTCATCGAGGCCACGCGGAGTACGATCGAGGGTACAGTGAA
AY394985.1 SARS coronavirus HSZ-Bb, complete genome	TTCATCGAGGCCACGCGGAGTACGATCGAGGGTACAGTGAA
AY394986.1 SARS coronavirus HSZ-Cb, complete genome	TTCATCGAGGCCACGCGGAGTACGATCGAGGGTACAGTGAA
AY394987.1 SARS coronavirus HZS2-Fb, complete genome	TTCATCGAGGCCACGCGGAGTACGATCGAGGGTACAGTGAA
AY394989.1 SARS coronavirus HZS2-D, complete genome	TTCATCGAGGCCACGCGGAGTACGATCGAGGGTACAGTGAA
AY394990.1 SARS coronavirus HZS2-E, complete genome	TTCATCGAGGCCACGCGGAGTACGATCGAGGGTACAGTGAA
AY394991.1 SARS coronavirus HZS2-Fc, complete genome	TTCATCGAGGCCACGCGGAGTACGATCGAGGGTACAGTGAA
AY394992.1 SARS coronavirus HZS2-C, complete genome	TTCATCGAGGCCACGCGGAGTACGATCGAGGGTACAGTGAA
AY394993.1 SARS coronavirus HGZ8L2, complete genome	TTCATCGAGGCCACGCGGAGTACGATCGAGGGTACAGTGAA
AY394994.1 SARS coronavirus HSZ-Bc, complete genome	TTCATCGAGGCCACGCGGAGTACGATCGAGGGTACAGTGAA
AY394995.1 SARS coronavirus HSZ-Cc, complete genome	TTCATCGAGGCCACGCGGAGTACGATCGAGGGTACAGTGAA
AY394996.1 SARS coronavirus ZS-B, complete genome	TTCATCGAGGCCACGCGGAGTACGATCGAGGGTACAGTGAA
AY394997.1 SARS coronavirus ZS-A, complete genome	TTCATCGAGGCCACGCGGAGTACGATCGAGGGTACAGTGAA
AY394998.1 SARS coronavirus LC1, complete genome	TTCATCGAGGCCACGCGGAGTACGATCGAGGGTACAGTGAA
AY394999.1 SARS coronavirus LC2, complete genome	TTCATCGAGGCCACGCGGAGTACGATCGAGGGTACAGTGAA
AY395000.1 SARS coronavirus LC3, complete genome	TTCATCGAGGCCACGCGGAGTACGATCGAGGGTACAGTGAA
AY395001.1 SARS coronavirus LC4, complete genome	TTCATCGAGGCCACGCGGAGTACGATCGAGGGTACAGTGAA
AY395002.1 SARS coronavirus LC5, complete genome	TTCATCGAGGCCACGCGGAGTACGATCGAGGGTACAGTGAA
AY395003.1 SARS coronavirus ZS-C, complete genome	TTCATCGAGGCCACGCGGAGTACGATCGAGGGTACAGTGAA
AY395004.1 SARS coronavirus HZS2-Bb, partial genome	TTCATCGAGGCCACGCGGAGTACGATCGAGGGTACAGTGAA
AY427439.1 SARS coronavirus AS, complete genome	TTCATCGAGGCCACGCGGAGTACGATCGAGGGTACAGTGAA
AY461660.1 SARS coronavirus SoD, complete genome	TTCATCGAGGCCACGCGGAGTACGATCGAGGGTACAGTGAA
AY463059.1 SARS coronavirus ShanghaiQXC1, complete genome	TTCATCGAGGCCACGCGGAGTACGATCGAGGGTACAGTGAA
AY463060.1 SARS coronavirus ShanghaiQXC2, complete genome	TTCATCGAGGCCACGCGGAGTACGATCGAGGGTACAGTGAA
AY485277.1 SARS coronavirus Sino1-11, complete genome	TTCATCGAGGCCACGCGGAGTACGATCGAGGGTACAGTGAA

AY485278.1 SARS coronavirus Sino3-11, complete genome	TTCATCGAGGCCACGCGGAGTACGATCGAGGGTACAGTGAA
AY502923.1 SARS coronavirus TW10, complete genome	TTCATCGAGGCCACGCGGAGTACGATCGAGGGTACAGTGAA
AY502925.1 SARS coronavirus TW2, complete genome	TTCATCGAGGCCACGCGGAGTACGATCGAGGGTACAGTGAA
AY502926.1 SARS coronavirus TW3, complete genome	TTCATCGAGGCCACGCGGAGTACGATCGAGGGTACAGTGAA
AY502927.1 SARS coronavirus TW4, complete genome	TTCATCGAGGCCACGCGGAGTACGATCGAGGGTACAGTGAA
AY502928.1 SARS coronavirus TW5, complete genome	TTCATCGAGGCCACGCGGAGTACGATCGAGGGTACAGTGAA
AY502929.1 SARS coronavirus TW6, complete genome	TTCATCGAGGCCACGCGGAGTACGATCGAGGGTACAGTGAA
AY502930.1 SARS coronavirus TW7, complete genome	TTCATCGAGGCCACGCGGAGTACGATCGAGGGTACAGTGAA
AY502931.1 SARS coronavirus TW8, complete genome	TTCATCGAGGCCACGCGGAGTACGATCGAGGGTACAGTGAA
AY502932.1 SARS coronavirus TW9, complete genome	TTCATCGAGGCCACGCGGAGTACGATCGAGGGTACAGTGAA
AY508724.1 SARS coronavirus NS-1, complete genome	TTCATCGAGGCCACGCGGAGTACGATCGAGGGTACAGTGAA
AY514485.1 Infectious bronchitis virus serotype California 99, complete genome	AGTGCCGAGGCCACGCGGAGTACGATCGAGGGTACAGCACT
AY515512.1 SARS coronavirus HC/SZ/61/03, complete genome	TTCATCGAGGCCACGCGGAGTACGATCGAGGGTACAGTGAA
AY536760.3 SARS coronavirus BJ01 nucleocapsid protein mRNA, complete cds	TTCATCGTGGCCACGCGGAGTACGATCGAGGGTACAGTGAA
AY545914.1 SARS coronavirus isolate HC/SZ/79/03, complete genome	TTCATCGAGGCCACGCGGAGTACGATCGAGGGTACAGTGAA
AY545915.1 SARS coronavirus isolate HC/SZ/DM1/03, complete genome	TTCATCGAGGCCACGCGGAGTACGATCGAGGGTACAGTGAA
AY545916.1 SARS coronavirus isolate HC/SZ/266/03, complete genome	TTCATCGAGGCCACGCGGAGTACGATCGAGGGTACAGTGAA
AY545917.1 SARS coronavirus isolate HC/GZ/81/03, complete genome	TTCATCGAGGCCACGCGGAGTACGATCGAGGGTACAGTGAA
AY545918.1 SARS coronavirus isolate HC/GZ/32/03, complete genome	TTCATCGAGGCCACGCGGAGTACGATCGAGGGTACAGTGAA
AY545919.1 SARS coronavirus isolate CFB/SZ/94/03, complete genome	TTCATCGAGGCCACGCGGAGTACGATCGAGGGTACAGTGAA
AY559081.1 SARS coronavirus Sin842, complete genome	TTCATCGAGGCCACGCGGAGTACGATCGAGGGTACAGTGAA
AY559082.1 SARS coronavirus Sin852, complete genome	TTCATCGAGGCCACGCGGAGTACGATCGAGGGTACAGTGAA
AY559083.1 SARS coronavirus Sin3408, complete genome	TTCATCGAGGCCACGCGGAGTACGATCGAGGGTACAGTGAA
AY559084.1 SARS coronavirus Sin3765V, complete genome	TTCATCGAGGCCACGCGGAGTACGATCGAGGGTACAGTGAA
AY559085.1 SARS coronavirus Sin848, complete genome	TTCATCGAGGCCACGCGGAGTACGATCGAGGGTACAGTGAA
AY559086.1 SARS coronavirus Sin849, complete genome	TTCATCGAGGCCACGCGGAGTACGATCGAGGGTACAGTGAA
AY559087.1 SARS coronavirus Sin3725V, complete genome	TTCATCGAGGCCACGCGGAGTACGATCGAGGGTACAGTGAA
AY559088.1 SARS coronavirus SinP1, complete genome	TTCATCGAGGCCACGCGGAGTACGATCGAGGGTACAGTGAA
AY559090.1 SARS coronavirus SinP3, complete genome	TTCATCGAGGCCACGCGGAGTACGATCGAGGGTACAGTGAA
AY559091.1 SARS coronavirus SinP4, complete genome	TTCATCGAGGCCACGCGGAGTACGATCGAGGGTACAGTGAA
AY559092.1 SARS coronavirus SinP5, complete genome	TTCATCGAGGCCACGCGGAGTACGATCGAGGGTACAGTGAA
AY559093.1 SARS coronavirus Sin845, complete genome	TTCATCGAGGCCACGCGGAGTACGATCGAGGGTACAGTGAA
AY559094.1 SARS coronavirus Sin846, complete genome	TTCATCGAGGCCACGCGGAGTACGATCGAGGGTACAGTGAA

AY559095.1 SARS coronavirus Sin847, complete genome	TTCATCGAGGCCACGCGGAGTACGATCGAGGGTACAGTGAA
AY559096.1 SARS coronavirus Sin850, complete genome	TTCATCGAGGCCACGCGGAGTACGATCGAGGGTACAGTGAA
AY559097.1 SARS coronavirus Sin3408L, complete genome	TTCATCGAGGCCACGCGGAGTACGATCGAGGGTACAGTGAA
AY568539.1 SARS coronavirus GZ0401, complete genome	TTCATCGAGGCCACGCGGAGTACGATCGAGGGTACAGTGAA
AY572038.1 SARS coronavirus civet020, complete genome	TTCATCGAGGCCACGCGGAGTACGATCGAGGGTACAGTGAA
AY595412.1 SARS coronavirus LLJ-2004, complete genome	TTCATCGAGGCCACGCGGAGTACGATCGAGGGTACAGTGAA
AY613947.1 SARS coronavirus GZ0402, complete genome	TTCATCGAGGCCACGCGGAGTACGATCGAGGGTACAGTGAA
AY613948.1 SARS coronavirus PC4-13, complete genome	TTCATCGAGGCCACGCGGAGTACGATCGAGGGTACAGTGAA
AY613949.1 SARS coronavirus PC4-136, complete genome	TTCATCGAGGCCACGCGGAGTACGATCGAGGGTACAGTGAA
AY613950.1 SARS coronavirus PC4-227, complete genome	TTCATCGAGGCCACGCGGAGTACGATCGAGGGTACAGTGAA
AY641576.1 Avian infectious bronchitis virus isolate Peafowl/GD/KQ6/2003, complete genome	AGTGCCGGGGCCACGCTGAGTACGACCGAGGGTACAGCACT
AY646283.1 Avian infectious bronchitis virus partridge/GD/S14/2003, complete genome	AGTGCCGGGGCCACGCGGAGTACGATCGAGGGTACAGCACT
AY654624.1 SARS coronavirus TJF, complete genome	TTCATCGAGGCCACGCGGAGTACGATCGAGGGTACAGTGAA
AY692454.1 Avian infectious bronchitis virus polyprotein 1a, polyprotein 1b, spike protein, 3a protein, 3b protein, sm	AGTGCCGGGGCCACGCGGAGTACGATCGAGGGTACAGCACT
AY714217.1 SARS Coronavirus CDC#200301157, complete genome	TTCATCGAGGCCACGCGGAGTACGATCGAGGGTACAGTGAA
AY720891.1 Human astrovirus type 4 strain Dresden, complete genome	GGAGCCGCGGCCACGCCGAGTAGGATCGAGGGTACAGCTCC
AY720892.1 Human astrovirus type 1 strain Dresden, complete genome	GAAGCCGCGGCCACGCCGAGTAGGAACGAGGGTACAGCTTC
AY772062.1 SARS coronavirus WH20, complete genome	TTCATCGAGGCCACGCGGAGTACGATCGAGGGTACAGTGAA
AY851295.1 Avian infectious bronchitis virus strain Mass 41, complete genome	AGTGCCGGGGCCACGCGGAGTACGACCGAGGGTACAGCACT
AY864805.1 SARS coronavirus BJ162, complete genome	TTCATCGAGGCCACGCGGAGTACGATCGAGGGTACAGTGAA
AY864806.1 SARS coronavirus BJ202, complete genome	TTCATCGAGGCCACGCGGAGTACGATCGAGGGTACAGTGAA
CQ903009.1 Sequence 13 from Patent WO2004094667	TTCATCGAGGCCACGCGGAGTACGATCGAGGGTACAGTGAA
CQ918584.1 Sequence 1 from Patent WO2004096842	TTCATCGAGGCCACGCGGAGTACGATCGAGGGTACAGTGAA
CQ918585.1 Sequence 2 from Patent WO2004096842	TTCATCGAGGCCACGCGGAGTACGATCGAGGGTACAGTGAA
CQ918598.1 Sequence 15 from Patent WO2004096842	TTCATCGAGGCCACGCGGAGTACGATCGAGGGTACAGTGAA
CQ918599.1 Sequence 16 from Patent WO2004096842	TTCATCGAGGCCACGCGGAGTACGATCGAGGGTACAGTGAA
CQ918601.1 Sequence 18 from Patent WO2004096842	TTCATCGAGGCCACGCGGAGTACGATCGAGGGTACAGTGAA
CQ918615.1 Sequence 32 from Patent WO2004096842	AGTGCCGGGGCCACGCGGAGTACGATCGAGGGTACAGCACT
CQ918737.1 Sequence 154 from Patent WO2004096842	TTCATCGAGGCCACGCGGAGTACGATCGAGGGTACAGTGAA
CQ918739.1 Sequence 156 from Patent WO2004096842	TTCATCGAGGCCACGCGGAGTACGATCGAGGGTACAGTGAA
CS079026.1 Sequence 14 from Patent WO2005035556	TTCATCGAGGCCACGCGGAGTACGATCGAGGGTACAGTGAA
CS079027.1 Sequence 15 from Patent WO2005035556	TTCATCGAGGCCACGCGGAGTACGATCGAGGGTACAGTGAA
CS079028.1 Sequence 16 from Patent WO2005035556	TTCATCGAGGCCACGCGGAGTACGATCGAGGGTACAGTGAA

CS079029.1 Sequence 17 from Patent WO2005035556	TTCATCGAGGCCACGCGGAGTACGATCGAGGGTACAGTGAA
CS116934.1 Sequence 1 from Patent WO2005056584	TTCATCGAGGCCACGCGGAGTACGATCGAGGGTACAGTGAA
CS116973.1 Sequence 40 from Patent WO2005056584	TTCATCGAGGCCACGCGGAGTACGATCGAGGGTACAGTGAA
CS117006.1 Sequence 73 from Patent WO2005056584	TTCATCGAGGCCACGCGGAGTACGATCGAGGGTACAGTGAA
CS117114.1 Sequence 1 from Patent WO2005056781	TTCATCGAGGCCACGCGGAGTACGATCGAGGGTACAGTGAA
CS117153.1 Sequence 40 from Patent WO2005056781	TTCATCGAGGCCACGCGGAGTACGATCGAGGGTACAGTGAA
CS117186.1 Sequence 73 from Patent WO2005056781	TTCATCGAGGCCACGCGGAGTACGATCGAGGGTACAGTGAA
CS254197.1 Sequence 67 from Patent WO2004011647	TTCATCGAGGCCACGCGGAGTACGATCGAGGGTACAGTGAA
CS569493.1 Sequence 1 from Patent WO2006039656	TTCATCGAGGCCACGCGGAGTACGATCGAGGGTACAGTGAA
DI195813.1 KR 1020100019423-A/41: ATTENUATED VIRUSES USEFUL FOR VACCINES	TTCATCGAGGCCACGCGGAGTACGATCGAGGGTACAGTGAA
DJ045279.1 IMAGEABLE ANIMAL MODEL OF SARS INFECTION	TTCATCGAGGCCACGCGGAGTACGATCGAGGGTACAGTGAA
DJ059765.1 NOVEL STRAIN OF SARS-ASSOCIATED CORONAVIRUS AND APPLICATIONS THEREOF	TTCATCGAGGCCACGCGGAGTACGATCGAGGGTACAGTGAA
DJ059791.1 NOVEL STRAIN OF SARS-ASSOCIATED CORONAVIRUS AND APPLICATIONS THEREOF	TTCATCGAGGCCACGCGGAGTACGATCGAGGGTACAGTGAA
DJ059821.1 NOVEL STRAIN OF SARS-ASSOCIATED CORONAVIRUS AND APPLICATIONS THEREOF	TTCATCGAGGCCACGCGGAGTACGATCGAGGGTACAGTGAA
DJ066921.1 A sensitive and specific test to detect SARS coronavirus	TTCATCGAGGCCACGCGGAGTACGATCGAGGGTACAGTGAA
DL008527.1 NOVEL STRAIN OF SARS-ASSOCIATED CORONAVIRUS AND APPLICATIONS THEREOF	TTCATCGAGGCCACGCGGAGTACGATCGAGGGTACAGTGAA
DL008553.1 NOVEL STRAIN OF SARS-ASSOCIATED CORONAVIRUS AND APPLICATIONS THEREOF	TTCATCGAGGCCACGCGGAGTACGATCGAGGGTACAGTGAA
DL008583.1 NOVEL STRAIN OF SARS-ASSOCIATED CORONAVIRUS AND APPLICATIONS THEREOF	TTCATCGAGGCCACGCGGAGTACGATCGAGGGTACAGTGAA
DL476508.1 MODIFIED SMALL INTERFERING RNA MOLECULES AND METHODS OF USE	TTCATCGAGGCCACGCGGAGTACGATCGAGGGTACAGTGAA
DQ001338.1 Avian infectious bronchitis virus isolate IBV-EP3, complete genome	AGTGCCGGGGCCACGCGGAGTACGATCGAGGGTACAGCACT
DQ001339.1 Avian infectious bronchitis virus isolate IBV-p65, complete genome	AGTGCCGGGGCCACGCGGAGTACGATCGAGGGTACAGCACT
DQ022305.2 Bat SARS coronavirus HKU3-1, complete genome	TTCACCGAGGCCACGCGGAGTACGATCGAGGGTACAGTGAA
DQ028633.1 Human astrovirus 5 isolate Goiania/GO/12/94/Brazil, complete genome	GAAGCCGCGGCCACGCCGAGTAGGACCGAGGGTACAGCTTC
DQ059621.1 Infectious bronchitis virus isolate N4/02 nucleocapsid protein (N) mRNA, complete cds	AGTGCCGGGGCCACGCGGAGTACGATCGAGGGTACAGCACT
DQ059622.1 Infectious bronchitis virus isolate N5/03 nucleocapsid protein (N) mRNA, complete cds	AGTGCCGGGGCCACGCGGAGTACGATCGAGGGTACAGCACT
DQ059623.1 Infectious bronchitis virus isolate N4/03 nucleocapsid protein (N) mRNA, complete cds	AGTGCCGGGGCCACGCGGAGTACGATCGAGGGTACAGCACT
DQ070852.1 Human astrovirus 4 isolate Goiania/GO/12/95/Brazil, complete genome	GGAGCCGCGGCCACGCCGAGTAGGATCGAGGGTACAGCTCC
DQ071615.1 Bat SARS coronavirus Rp3, complete genome	TTCACCGAGGCCACGCGGAGTACGATCGAGGGTACAGTGAA
DQ084199.1 bat SARS coronavirus HKU3-2, complete genome	TTCACCGAGGCCACGCGGAGTACGATCGAGGGTACAGTGAA
DQ084200.1 bat SARS coronavirus HKU3-3, complete genome	TTCACCGAGGCCACGCGGAGTACGATCGAGGGTACAGTGAA
DQ182595.1 SARS coronavirus ZJ0301 from China, complete genome	TTCATCGAGGCCACGCGGAGTACGATCGAGGGTACAGTGAA
DQ288927.1 Avian infectious bronchitis virus isolate SAIBK, complete genome	AGTGCCGAGGCCACGCGGAGTACGATCGAGGGTACAGCACT
DQ344027.1 Human astrovirus 4 isolate Guangzhou from China, complete genome	GGAGCCGCGGCCACGCCGAGTAGGATCGAGGGTACAGCTCC

DQ412042.1 Bat SARS coronavirus Rf1, complete genome	TTCACCGAGGCCACGCGGAGTACGATCGAGGGTACAGTGAA
DQ412043.1 Bat SARS coronavirus Rm1, complete genome	TTCACCGAGGCCACGCGGAGTACGATCGAGGGTACAGTGAA
DQ497008.1 SARS coronavirus strain MA-15, complete genome	TTCATCGAGGCCACGCGGAGTACGATCGAGGGTACAGTGAA
DQ630763.4 Human astrovirus 3 isolate WH1859 capsid protein gene, complete cds	GAGACCGCGGCCACGCCGAGTAGGATCGAGGGTACAGTCTC
DQ640652.1 SARS coronavirus GDH-BJH01, complete genome	TTCATCGAGGCCACGCGGAGTACGATCGAGGGTACAGTGAA
DQ646404.1 Infectious bronchitis virus isolate TW2296/95 spike protein (S), 3a protein (3a), 3b protein (3b), E protein	AGTGCCGGGGCCACGCGGAGTACGATCGAGGGTACAGCACT
DQ646405.2 Infectious bronchitis virus isolate TW2575/98, complete genome	AGTGCCGGGGCCACGCGGAGTACGATCGAGGGTACAGCACT
DQ646406.1 Infectious bronchitis virus isolate TW1171/92 spike protein (S), 3a protein (3a), 3b protein (3b), E protein	AGTGCCGGGGCCACGCGGAGTACGATCGAGGGTACAGCACT
DQ648856.1 Bat coronavirus (BtCoV/273/2005), complete genome	TTCACCGAGGCCACGCGGAGTACGATCGAGGGTACAGTGAA
DQ648857.1 Bat coronavirus (BtCoV/279/2005), complete genome	TTCACCGAGGCCACGCGGAGTACGATCGAGGGTACAGTGAA
DQ834384.1 Infectious bronchitis virus strain M41, complete genome	AGTGCCGGGGCCACGCGGAGTACGACCGAGGGTACAGCACT
DQ898174.1 SARS coronavirus strain CV7, complete genome	TTCATCGAGGCCACGCGGAGTACGATCGAGGGTACAGTGAA
EF165621.1 Infectious bronchitis virus isolate VaccineM48 3' UTR	TATGCCCAGGCCACCCGAACTAAGATCGCGGGTACCGCTTT
EF544158.1 Infectious bronchitis virus isolate SAIB9 nucleocapsid protein gene, partial cds; and 3' UTR	AGTGCCGGGGCCACGCGGAGTACGATCGAGGGTACAGCACT
EF544159.1 Infectious bronchitis virus isolate SAIB14 nucleocapsid protein gene, partial cds; and 3' UTR	AGTGCCGGGGCCACGCGGAGTACGATCGAGGGTACAGCACT
EF544160.1 Infectious bronchitis virus isolate SAIB20 nucleocapsid protein gene, partial cds; and 3' UTR	AGTGCCGAGGCCACGCGGAGTACGATCGAGGGTACAGCACT
EF544161.1 Infectious bronchitis virus isolate SAIBb2 nucleocapsid protein gene, partial cds; and 3' UTR	AGTGCCGGGGCCACGCGGAGTACGATCGAGGGTACAGCACT
EF544162.1 Infectious bronchitis virus isolate SAIBb6 nucleocapsid protein gene, partial cds; and 3' UTR	AGTGCCGATGCCACGCGGAGTACGATCGAGGGTACAGCACT
EF544163.1 Infectious bronchitis virus isolate SAIBbs nucleocapsid protein gene, partial cds; and 3' UTR	AGTGCCGGGGCCACGCGGAGTACGATCGAGGGTACAGCACT
EF544164.1 Infectious bronchitis virus isolate SAIBcq nucleocapsid protein gene, partial cds; and 3' UTR	AGTGCCGGGGCCACGCGGAGTACGATCGAGGGTACAGCACT
EF544165.1 Infectious bronchitis virus isolate SAIBdy nucleocapsid protein gene, partial cds; and 3' UTR	AGTGCCGGGGCCACGCGGAGTACGATCGAGGGTACAGCACT
EF544166.1 Infectious bronchitis virus isolate SAIBK nucleocapsid protein gene, partial cds; and 3' UTR	AGTGCCGAGGCCACGCGGAGTACGATCGAGGGTACAGCACT
EF544167.1 Infectious bronchitis virus isolate SAIBw6 nucleocapsid protein gene, partial cds; and 3' UTR	AGTGCCGGGGCCACGCGGAGTACGATCGAGGGTACAGCACT
EF544168.1 Infectious bronchitis virus isolate SAIBwj nucleocapsid protein gene, partial cds; and 3' UTR	AGTGCCGGGGCCACGCTGAGTACGACCGAGGGTACAGCACT
EF583300.1 Human astrovirus 3 strain WH1859 capsid protein precursor, gene, complete cds	GAGACCGCGGCCACGCCGAGTAGGATCGAGGGTACAGTCTC
EU022525.1 Turkey coronavirus isolate TCoV-540, complete genome	AGTGCCGGGGCCACGCGGCGTACGATCGAGGGTACAGCACT
EU022526.1 Turkey coronavirus isolate TCoV-ATCC, complete genome	AGTGCCGAGGCCACGCGGAGTACGATCGAGGGTACAGCACT
EU095850.1 Turkey coronavirus isolate MG10, complete genome	AGTGCCGGGGCCACGCGGAGTACGATCGAGGGTACAGCACA
EU116941.1 Infectious bronchitis virus strain M41 nucleocapsid protein gene, complete cds	AGTGCCGGGGCCACGCGGAGTACGACCGAGGGTACAGCACT
EU224456.1 Norovirus dog/170/07/Ita polymerase gene, partial cds; and capsid protein and basic polypeptide genes,	CTGACCGCGGCCACGCCGAGTAGGATCGAGGGTACAGTCAG
EU371559.1 SARS coronavirus ZJ02, complete genome	TTCATCGAGGCCACGCGGAGTACGATCGAGGGTACAGTGAA
EU371560.1 SARS coronavirus BJ182a, complete genome	TTCATCGAGGCCACGCGGAGTACGATCGAGGGTACAGTGAA
EU371561.1 SARS coronavirus BJ182b, complete genome	TTCATCGAGGCCACGCGGAGTACGATCGAGGGTACAGTGAA

EU371562.1 SARS coronavirus BJ182-4, complete genome	TTCATCGAGGCCACGCGGAGTACGATCGAGGGTACAGTGAA
EU371563.1 SARS coronavirus BJ182-8, complete genome	TTCATCGAGGCCACGCGGAGTACGATCGAGGGTACAGTGAA
EU371564.1 SARS coronavirus BJ182-12, complete genome	TTCATCGAGGCCACGCGGAGTACGATCGAGGGTACAGTGAA
EU418975.1 Infectious bronchitis virus strain ArkDPI101, complete genome	AGTGCCGGGGCCACGCGGAGTACGATCGAGGGTACAGCACT
EU418976.1 Infectious bronchitis virus strain ArkDPI11, complete genome	AGTGCCGGGGCCACGCGGAGTACGATCGAGGGTACAGCACT
EU526388.1 Infectious bronchitis virus strain A2, complete genome	AGTGCCGAGGCCACGCGGAGTACGATCGAGGGTACAGCACT
EU637854.1 Infectious bronchitis virus strain CK/CH/LSD/05I from China, complete genome	AGTGCCGGGGCCACGCGGAGTACGATCGAGGGTACAGCACT
EU714028.1 Infectious bronchitis virus isolate ZJ971, complete genome	AGTGCCGGGGCCACGCGGAGTACGATCGAGGGTACAGCACT
EU714029.1 Infectious bronchitis virus isolate SC021202, complete genome	AGTGCCGGGGCCACGCGGAGTACGATCGAGGGTACAGCACT
EU817497.1 Infectious bronchitis virus strain H52, complete genome	AGTGCCGGGGCCACGCGGAGTACGATCGAGGGTACAGCACT
EU822336.1 Infectious bronchitis virus isolate 3468/07 spike protein (S), 3a protein (3a), 3b protein (3b), envelope pr	AGTGCCGGGGCCACGCGGAGTACGATCGAGGGTACAGCACT
EU822337.1 Infectious bronchitis virus isolate 3374/05 spike protein (S), 3a protein (3a), truncated 3b protein (3b), er	AGTGCCGGGGCCACGCGGAGTACGATCGAGGGTACAGCACT
EU822338.1 Infectious bronchitis virus isolate 3263/04 spike protein (S), 3a protein (3a), 3b protein (3b), envelope pr	AGTGCCGGGGCCACGCGGAGTACGATCGAGGGTACAGCACT
EU822339.1 Infectious bronchitis virus isolate 3071/03 spike protein (S), 3a protein (3a), truncated 3b protein (3b), er	AGTGCCGGGGCCACGCGGAGTACGATCGAGGGTACAGCACT
EU822340.1 Infectious bronchitis virus isolate 2992/02 spike protein (S), 3a protein (3a), 3b protein (3b), truncated er	AGTGCCGGGGCCACGCGGAGTACGATCGAGGGTACAGCACT
EU822341.1 Infectious bronchitis virus strain H120 spike protein (S), 3a protein (3a), 3b protein (3b), envelope protei	AGTGCCGGGGCCACGCGGAGTACGATCGAGGGTACAGCACT
EU847144.2 Mamastrovirus 14 isolate AFCD57 polyprotein 1AB gene, partial cds; and capsid protein precursor, gene,	AGAGCCGAGGCCACGCCAAGTCGGATCGAGGGTACAGCTCA
EU847145.2 Mamastrovirus 16 isolate AFCD11 polyprotein 1AB gene, partial cds; and capsid protein precursor, gene,	TGAGCCGAGGCCACGCCGAGTAGGATCGAGGGTACAGCTCC
FJ008695.1 Infectious bronchitis virus strain Md27 spike protein, 3a protein, 3b protein, small envelope protein, mem	AGTGCCGAGGCCACGCGGAGTACGATCGAGGGTACAGCACT
FJ375759.1 Human astrovirus isolate SH1, complete genome	GAAGCCGCGGCCACGCCGAGTAGGATCGAGGGTACAGCTTC
FJ376619.2 Bulbul coronavirus HKU11-934, complete genome	TGTGCCGAGGCCACGCGGAGTACGATCGAGGGTACAGCACA
FJ376620.1 Bulbul coronavirus HKU11-796, complete genome	TGTGCCGAGGCCACGCGGAGTACGATCGAGGGTACAGCACA
FJ376621.1 Thrush coronavirus HKU12-600, complete genome	TATGCCGAGGCCACGCGGAGTACGATCGAGGGTACAGCATA
FJ376622.1 Munia coronavirus HKU13-3514, complete genome	TGTGTCGAGGCCACGCGGAGTACGATCGAGGGTACAGCACA
FJ434664.1 Avastrovirus 3 strain C-NGB, complete genome	GCAGCCGCGGCCACGCCGAGTAGGATCGAGGGTACAGCTGC
FJ571068.1 Bat astrovirus Ha/Guangxi/LS11/2007 non-structural polyprotein 1AB (pol) gene, partial cds; and capsid p	CACCCCGCGGCCACGCCGAGTAGGAACGAGGGTACAGGGTG
FJ588686.1 Bat SARS CoV Rs672/2006, complete genome	TTCACCGAGGCCACGCGGAGTACGATCGAGGGTACAGTGAA
FJ755402.1 Human astrovirus 1 Beijing/128/2005/CHN, complete genome	GAAGCCGCGGCCACGCCGAGTAGGAACGAGGGTACAGCTTC
FJ755403.1 Human astrovirus 1 Beijing/176/2006/CHN, complete genome	GAAGCCGCGGCCACGCCGAGTAGGAACGAGGGTACAGCTTC
FJ755404.1 Human astrovirus 1 Beijing/291/2007/CHN, complete genome	GAAGCCGCGGCCACGCCGAGTAGGAACGAGGGTACAGCTTC
FJ755405.1 Human astrovirus 1 Beijing/293/2007/CHN, complete genome	GAAGCCGCGGCCACGCCGAGTAGGAACGAGGGTACAGCTTC
FJ807652.1 Infectious bronchitis virus strain H120, complete genome	AGTGCCGGGGCCACGCGGAGTACGATCGAGGGTACAGCACT
FJ807653.1 Infectious bronchitis virus isolate chicken/JS/YZ07/2008 spike protein, 3a protein, 3b protein, small virion	AGTGCCGGGGCCACGCGGAGTACGATCGAGGGTACAGCACT

FJ842148.1 Human astrovirus isolate NK180 capsid protein gene, partial cds	GAGACCGCGGCCACGCCGAGTAGGATCGAGGGTACAGTCTC
FJ875027.1 Norovirus dog/GVI.1/Bari/91/2007/ITA polymerase gene, partial cds; and VP1 and minor capsid protein g	TTAGCCGCGGCCACGCCGAGTAGGATCGAGGGTACAGCTGA
FJ882926.1 SARS coronavirus ExoN1, complete genome	TTCATCGAGGCCACGCGGAGTACGATCGAGGGTACAGTGAA
FJ882927.1 SARS coronavirus wtic-MB isolate P1pp1, complete genome	TTCATCGAGGCCACGCGGAGTACGATCGAGGGTACAGTGAA
FJ882928.1 SARS coronavirus ExoN1 isolate P1pp1, complete genome	TTCATCGAGGCCACGCGGAGTACGATCGAGGGTACAGTGAA
FJ882929.1 SARS coronavirus ExoN1 isolate P3pp1, complete genome	TTCATCGAGGCCACGCGGAGTACGATCGAGGGTACAGTGAA
FJ882930.1 SARS coronavirus ExoN1, complete genome	TTCATCGAGGCCACGCGGAGTACGATCGAGGGTACAGTGAA
FJ882931.1 SARS coronavirus ExoN1 isolate P3pp12, complete genome	TTCATCGAGGCCACGCGGAGTACGATCGAGGGTACAGTGAA
FJ882932.1 SARS coronavirus wtic-MB isolate P3pp14, complete genome	TTCATCGAGGCCACGCGGAGTACGATCGAGGGTACAGTGAA
FJ882933.1 SARS coronavirus wtic-MB isolate P3pp6, complete genome	TTCATCGAGGCCACGCGGAGTACGATCGAGGGTACAGTGAA
FJ882934.1 SARS coronavirus wtic-MB isolate P3pp29, complete genome	TTCATCGAGGCCACGCGGAGTACGATCGAGGGTACAGTGAA
FJ882935.1 SARS coronavirus wtic-MB isolate P3pp21, complete genome	TTCATCGAGGCCACGCGGAGTACGATCGAGGGTACAGTGAA
FJ882936.1 SARS coronavirus wtic-MB isolate P3pp2, complete genome	TTCATCGAGGCCACGCGGAGTACGATCGAGGGTACAGTGAA
FJ882937.1 SARS coronavirus wtic-MB isolate P3pp18, complete genome	TTCATCGAGGCCACGCGGAGTACGATCGAGGGTACAGTGAA
FJ882938.1 SARS coronavirus wtic-MB, complete genome	TTCATCGAGGCCACGCGGAGTACGATCGAGGGTACAGTGAA
FJ882939.1 SARS coronavirus wtic-MB isolate P3pp16, complete genome	TTCATCGAGGCCACGCGGAGTACGATCGAGGGTACAGTGAA
FJ882940.1 SARS coronavirus ExoN1 isolate P3pp37, complete genome	TTCATCGAGGCCACGCGGAGTACGATCGAGGGTACAGTGAA
FJ882941.1 SARS coronavirus ExoN1 isolate P3pp8, complete genome	TTCATCGAGGCCACGCGGAGTACGATCGAGGGTACAGTGAA
FJ882942.1 SARS coronavirus MA15 ExoN1 isolate P3pp5, complete genome	TTCATCGAGGCCACGCGGAGTACGATCGAGGGTACAGTGAA
FJ882943.1 SARS coronavirus MA15 ExoN1, complete genome	TTCATCGAGGCCACGCGGAGTACGATCGAGGGTACAGTGAA
FJ882944.1 SARS coronavirus ExoN1 isolate P3pp23, complete genome	TTCATCGAGGCCACGCGGAGTACGATCGAGGGTACAGTGAA
FJ882945.1 SARS coronavirus MA15 isolate P3pp6, complete genome	TTCATCGAGGCCACGCGGAGTACGATCGAGGGTACAGTGAA
FJ882946.1 SARS coronavirus wtic-MB isolate P3pp13, complete genome	TTCATCGAGGCCACGCGGAGTACGATCGAGGGTACAGTGAA
FJ882947.1 SARS coronavirus wtic-MB isolate P3pp7, complete genome	TTCATCGAGGCCACGCGGAGTACGATCGAGGGTACAGTGAA
FJ882948.1 SARS coronavirus MA15 isolate P3pp3, complete genome	TTCATCGAGGCCACGCGGAGTACGATCGAGGGTACAGTGAA
FJ882949.1 SARS coronavirus wtic-MB isolate P3pp23, complete genome	TTCATCGAGGCCACGCGGAGTACGATCGAGGGTACAGTGAA
FJ882950.1 SARS coronavirus ExoN1 isolate P3pp60, complete genome	TTCATCGAGGCCACGCGGAGTACGATCGAGGGTACAGTGAA
FJ882951.1 SARS coronavirus MA15 ExoN1 isolate P3pp3, complete genome	TTCATCGAGGCCACGCGGAGTACGATCGAGGGTACAGTGAA
FJ882952.1 SARS coronavirus MA15 isolate P3pp4, complete genome	TTCATCGAGGCCACGCGGAGTACGATCGAGGGTACAGTGAA
FJ882953.1 SARS coronavirus MA15 ExoN1 isolate P3pp4, complete genome	TTCATCGAGGCCACGCGGAGTACGATCGAGGGTACAGTGAA
FJ882954.1 SARS coronavirus ExoN1 isolate P3pp46, complete genome	TTCATCGAGGCCACGCGGAGTACGATCGAGGGTACAGTGAA
FJ882955.1 SARS coronavirus ExoN1 isolate P3pp19, complete genome	TTCATCGAGGCCACGCGGAGTACGATCGAGGGTACAGTGAA
FJ882957.1 SARS coronavirus MA15, complete genome	TTCATCGAGGCCACGCGGAGTACGATCGAGGGTACAGTGAA

FJ882958.1 SARS coronavirus MA15 isolate P3pp7, complete genome	TTCATCGAGGCCACGCGGAGTACGATCGAGGGTACAGTGAA
FJ882959.1 SARS coronavirus MA15 ExoN1 isolate P3pp6, complete genome	TTCATCGAGGCCACGCGGAGTACGATCGAGGGTACAGTGAA
FJ882960.1 SARS coronavirus ExoN1 isolate P3pp34, complete genome	TTCATCGAGGCCACGCGGAGTACGATCGAGGGTACAGTGAA
FJ882961.1 SARS coronavirus MA15 isolate P3pp5, complete genome	TTCATCGAGGCCACGCGGAGTACGATCGAGGGTACAGTGAA
FJ882962.1 SARS coronavirus MA15 ExoN1 isolate P3pp10, complete genome	TTCATCGAGGCCACGCGGAGTACGATCGAGGGTACAGTGAA
FJ882963.1 SARS coronavirus P2, complete genome	TTCATCGAGGCCACGCGGAGTACGATCGAGGGTACAGTGAA
FJ888351.1 Infectious bronchitis virus strain H120, complete genome	AGTGCCGGGGCCACGCGGAGTACGATCGAGGGTACAGCACT
FJ890351.1 Mamastrovirus 11 RNA-dependent RNA polymerase gene, partial cds; and capsid protein gene, complete	CTTCCCGAGGCCACGCCGAGTAGGACCGAGGGTACAGGGAG
FJ890352.1 California sea lion astrovirus 2 RNA-dependent RNA polymerase gene, partial cds; and capsid protein gen	CAAGCCGAGGCCACGCCGAGTAGGATCGAGGGTACAGCTTG
FJ904714.1 Infectious bronchitis virus strain Cal 1995, complete genome	AGTGCCGAGGCCACGCGGAGTACGATCGAGGGTACAGCACT
FJ904715.1 Infectious bronchitis virus strain Cal557 2003, complete genome	AGTGCCGGGGCCACGCGGAGTACGATCGAGGGTACAGCACT
FJ904716.1 Infectious bronchitis virus strain Conn46 1996, complete genome	AGTGCCGAGGCCACGCGGAGTACGATCGAGGGTACAGCACT
FJ904717.1 Infectious bronchitis virus strain Conn46 1972, complete genome	AGTGCCGAGGCCACGCGGAGTACGATCGAGGGTACAGCACT
FJ904718.1 Infectious bronchitis virus strain Conn46 1983, complete genome	AGTGCCGAGGCCACGCGGAGTACGATCGAGGGTACAGCACT
FJ904719.1 Infectious bronchitis virus strain Conn46 1991, complete genome	AGTGCCGAGGCCACGCGGAGTACGATCGAGGGTACAGCACT
FJ904720.1 Infectious bronchitis virus strain Mass41 1965, complete genome	AGTGCCGGGGCCACGCGGAGTACGACCGAGGGTACAGCACT
FJ904721.1 Infectious bronchitis virus strain Mass41 1972, complete genome	AGTGCCGGGGCCACGCGGAGTACGACCGAGGGTACAGCACT
FJ904722.1 Infectious bronchitis virus strain Mass41 1979, complete genome	AGTGCCGGGGCCACGCGGAGTACGATCGAGGGTACAGCACT
FJ904723.1 Infectious bronchitis virus strain Mass41 1985, complete genome	AGTGCCGGGGCCACGCGGAGTACGACCGAGGGTACAGCACT
FJ919225.1 Duck astrovirus 1 strain DA06, complete genome	GCAGCCGCGGCCACGCCGAGTAGGATCGAGGGTACAGCTGC
FJ919226.1 Duck astrovirus 1 strain DA07, complete genome	GCAGCCGCGGCCACGCCGAGTAGGATCGAGGGTACAGCTGC
FJ919227.1 Duck astrovirus 1 strain DA08, complete genome	GCAGCCGCGGCCACGCCGAGTAGGATCGAGGGCACAGCTGC
FJ919228.1 Duck astrovirus 1 strain DA93, complete genome	GCAGCCGCGGCCACGCCGAGTAGGATCGAGGGTACAGCTGC
FJ959407.1 SARS coronavirus isolate A001, complete genome	TTCATCGATGCCACGCGGAGTACGATCGAGGGTACAGTGAA
FJ973620.1 Astrovirus VA1, complete genome	TGCGCCGAGGCCACGCCGAGTAGGATCGAGGGTACAGCGCT
FM213330.1 Astrovirus dogfaeces/Italy/2005 partial RNA-dependant RNA polymerase, genomic RNA	GTTCCCGAGGCCACGCCGAGTAGGATCGAGGGTACAGGTTC
FN430414.1 Infectious bronchitis virus ITA/90254/2005, complete genome	AGTGCCGGGGCCACGCGGAGTACGATCGAGGGTACAGCACT
FN430415.1 Infectious bronchitis virus NGA/A116E7/2006, complete genome	AGTGCCGGGGCCACGCGGAGTACGATCGAGGGTACAGCACT
FR727146.1 Feral pigeon astrovirus partial RNA-dependent RNA polymerase and capsid protein precursor, strain 03/5	TATACCGAGGCCACGGCGAGTAGCATCGAGGGTACAGTATA
FR727147.1 Feral pigeon astrovirus partial RNA-dependent RNA polymerase and capsid protein precursor, strain 03/5	TATACCGAGGCCACGGCGAGTAGCATCGAGGGTACAGTATA
FR727148.1 Feral pigeon astrovirus partial RNA-dependent RNA polymerase and capsid protein precursor, strain 03/6	TTTACCGAGGCCACGGCGAGTAGCATCGAGGGTACAGTAAA
FV537210.1 Modified Microbial Nucleic Acid	TTTATTGAGGTTATGTGGAGTATGATTGAGGGTATAGTGAA
FV537211.1 Modified Microbial Nucleic Acid	TTTATTGAGGTTATGTGGAGTATGATTGAGGGTATAGTGAA

FW503166.1 Attenuated Viruses Useful For Vaccines	TTCATCGAGGCCACGCGGAGTACGATCGAGGGTACAGTGAA
GN350553.1 Sequence 317 from Patent WO2007130519	TTTCCCGAGGCCACGGCGAGTAGCATCGAGGGTACAGGAAA
GN350554.1 Sequence 318 from Patent WO2007130519	TTTCCCGAGGCCACGGCGAGTAGCATCGAGGGTACAGGAAA
GN351429.1 Sequence 1193 from Patent WO2007130519	CTCGCCGAGGCCACGCCGAGTAGGACCGAGGGTACAGCGAG
GN351430.1 Sequence 1194 from Patent WO2007130519	CTCGCCGAGGCCACGCCGAGTAGGACCGAGGGTACAGCGAG
GN352049.1 Sequence 1813 from Patent WO2007130519	GAAGCCGCGGCCACGCCGAGTAGGAACGAGGGTACAGCTTC
GN352050.1 Sequence 1814 from Patent WO2007130519	GAAGCCGCGGCCACGCCGAGTAGGAACGAGGGTACAGCTTC
GN354837.1 Sequence 4601 from Patent WO2007130519	AATCCCGAGGCCACGCCGAGTAGGATCGAGGGTACAGGATT
GN354838.1 Sequence 4602 from Patent WO2007130519	AATCCCGAGGCCACGCCGAGTAGGATCGAGGGTACAGGATT
GN355917.1 Sequence 5681 from Patent WO2007130519	GATGCCGAGGCCACGCCGGGTAGGATCGAGGGTACAGCATC
GN355918.1 Sequence 5682 from Patent WO2007130519	GATGCCGAGGCCACGCCGGGTAGGATCGAGGGTACAGCATC
GQ153539.1 Bat SARS coronavirus HKU3-4, complete genome	TTCACCGAGGCCACGCGGAGTACGATCGAGGGTACAGTGAA
GQ153540.1 Bat SARS coronavirus HKU3-5, complete genome	TTCACCGAGGCCACGCGGAGTACGATCGAGGGTACAGTGAA
GQ153541.1 Bat SARS coronavirus HKU3-6, complete genome	TTCACCGAGGCCACGCGGAGTACGATCGAGGGTACAGTGAA
GQ153542.1 Bat SARS coronavirus HKU3-7, complete genome	TTCACCGAGGCCACGCGGAGTACGATCGAGGGTACAGTGAA
GQ153543.1 Bat SARS coronavirus HKU3-8, complete genome	TTCACCGAGGCCACGCGGAGTACGATCGAGGGTACAGTGAA
GQ153544.1 Bat SARS coronavirus HKU3-9, complete genome	TTCACCGAGGCCACGCGGAGTACGATCGAGGGTACAGTGAA
GQ153545.1 Bat SARS coronavirus HKU3-10, complete genome	TTCACCGAGGCCACGCGGAGTACGATCGAGGGTACAGTGAA
GQ153546.1 Bat SARS coronavirus HKU3-11, complete genome	TTCACCGAGGCCACGCGGAGTACGATCGAGGGTACAGTGAA
GQ153547.1 Bat SARS coronavirus HKU3-12, complete genome	TTCACCGAGGCCACGCGGAGTACGATCGAGGGTACAGTGAA
GQ153548.1 Bat SARS coronavirus HKU3-13, complete genome	TTCACCGAGGCCACGCGGAGTACGATCGAGGGTACAGTGAA
GQ267696.1 Astrovirus human/SZ908/China/2008 3C-like serine proteinase and RNA-dependent RNA polymerase mR	TGCGCCGAGGCCACGCCGAGTAGGATCGAGGGTACAGCGCT
GQ405855.1 Human astrovirus 1 isolate Dalian/2007/CHN outer capsid protein gene, complete cds	GAAGCCGCGGCCACGCCGAGTAGGAACGAGGGTACAGCTTC
GQ405856.1 Human astrovirus 1 isolate Shenyang/2007/CHN outer capsid protein gene, complete cds	GAAGCCGCGGCCACGCCGAGTAGGAACGAGGGTACAGCTTC
GQ405857.1 Human astrovirus 4 isolate Panjin/2007/CHN outer capsid protein gene, complete cds	GGAGCCGCGGCCACGCCGAGTAGGATCGAGGGTACAGCTCC
GQ415660.1 Mamastrovirus 8 isolate NI-295, complete genome	TCCGCCGAGGCCACGCCGAGTAGGATCGAGGGTACAGCGGA
GQ415662.1 HMO Astrovirus C isolate NE-3010 non-structural protein gene, partial cds; and capsid gene, complete cd	TGCGCCGAGGCCACGCCGAGTAGGATCGAGGGTACAGCGCT
GQ427173.1 Turkey coronavirus strain TCoV/VA-74/03, complete genome	AGTGCCGGGGCCACGCGGCGTACGATCGTGGGTACAGCACT
GQ427175.1 Turkey coronavirus strain TCoV/IN-517/94, complete genome	AGTGCCGGGGCCACGCGGAGTACGATCGAGGGTACAGCACT
GQ427176.1 Turkey coronavirus strain TCoV/TX-1038/98, complete genome	AGTGCCGGGGCCACGCGGAGTACGATCGAGGGTACAGCACA
GQ443611.1 Norovirus dog/C33/Viseu/2007/PRT RNA-dependent RNA polymerase gene, partial cds; and major capsi	TTGACCGCGGCCACGCCGAGTAGGATCGAGGGTACAGTCAG
GQ495608.1 Human astrovirus 6 isolate 192-BJ07-CHN, complete genome	GAAGCCGCGGCCACGCCGAGTAGGATCGAGGGTACAGCTTC
GQ502193.2 Astrovirus VA2 isolate VA2/human/Stl/WD0680/2009, complete genome	TCCGCCGAGGCCACGCCGAGTAGGATCGAGGGTACAGCGGA

GQ504720.1 Infectious bronchitis virus strain Arkansas DPI, complete genome	AGTGCCGGGGCCACGCGGAGTACGATCTAGGGTTACAGCAC
GQ504721.2 Infectious bronchitis virus strain Arkansas Vaccine, complete genome	AGTGCCGGGGCCACCCGGAGTACGATCGAGGGTACAGCACT
GQ504723.1 Infectious bronchitis virus strain Georgia 1998 Vaccine, complete genome	AGTGCCGGGGCCACGCGGAGTACGATCGAGGGTACAGCACT
GQ504724.1 Infectious bronchitis virus strain Massachusetts, complete genome	AGTGCCGGGGCCACGGGGAGTACGACCGAGGGTACAGCACT
GQ891990.1 Astrovirus SG, complete genome	TGCGCCGAGGCCACGCCGAGTAGGATCGAGGGTACAGCGCT
GQ901902.2 Human astrovirus 6 isolate Rus-Nsc09-B4 putative serine protease gene, complete cds; putative RNA-de	GAAGCCGCGGCCACGCCGAGTAGGATCGAGGGTACAGCTTC
GQ914773.1 Porcine astrovirus 2 Shanghai/2008 polyprotein and capsid protein genes, complete cds	AAAGCCGAGGCCACGCCGAGTAGGTTCGAGGGTACAGCTTT
GU182408.1 Turdivirus 2 strain 10717, complete genome	AGACCCGAGGCCACGCCGAGTAGGATCGAGGGTACAGGTCT
GU182409.1 Turdivirus 2 strain 007167, complete genome	AGACCCGAGGCCACGCCGAGTAGGATCGAGGGTACAGGTCT
GU182410.1 Turdivirus 3 strain 10878, complete genome	GCTCCCGCGGCCACGCCGAGTAGGATCGAGGGTACAGGAGC
GU182411.1 Turdivirus 3 strain 00742, complete genome	GCTCCCGCGGCCACGCCGAGTAGGATCGAGGGTACAGGAGC
GU190215.1 Bat coronavirus BM48-31/BGR/2008, complete genome	GTCACCGAGGCCACGCGGAGTACGATCGAGGGTACAGTGAC
GU222330.1 Avian nephritis virus AFBI-2010 isolate VF04-1/2 structural protein gene, partial sequence; and 3' UTR	TTTCCCGAGGCCACGGCGAGTAGCATCGAGGGTACAGGAAT
GU222331.1 Avian nephritis virus AFBI-2010 isolate VF07-13/7 structural protein gene, partial sequence; and 3' UTR	TTTCCCGAGGCCACGGCGAGTAGCATCGAGGGTACAGGAAA
GU222332.1 Avian nephritis virus AFBI-2010 isolate VF08-3a structural protein gene, partial sequence; and 3' UTR	TTTCCCGAGGCCACGGCGAGTAGCATCGAGGGTACAGGAAA
GU222333.1 Avian nephritis virus AFBI-2010 isolate VF08-18/5 structural protein gene, partial sequence; and 3' UTR	TTTCCCGAGGCCACGGCGAGTAGCATCGAGGGTACAGGAAA
GU222334.1 Avian nephritis virus AFBI-2010 isolate VF08-29a structural protein gene, partial sequence; and 3' UTR	TTTCCCGAGGCCACGGCGAGTAGCATCGAGGGTACAGGAAA
GU223905.2 Human astrovirus 3 isolate Rus-Nsc03-H191 putative serine protease gene, complete cds; putative RNA-(	GAGACCGCGGCCACGCCGAGTAGGATCGAGGGTACAGTCTC
GU393331.1 Infectious bronchitis virus serotype Cal56b, complete genome	AGTGCCGGGGCCACGCGGAGTACGATCGAGGGTACAGCACT
GU393332.1 Infectious bronchitis virus serotype Delaware 072, complete genome	GTGCCGAGGGCCACGCGGAGTACGATCGAGGGTACAGCACT
GU393333.1 Infectious bronchitis virus serotype FL18288, complete genome	AGTGCCGAGGCCACGCGGAGTACGATCGAGGGTACAGCACT
GU393334.1 Infectious bronchitis virus serotype Gray, complete genome	AGTGCCGGGGCCACGCGGAGTACGATCGAGGGTACAGCACT
GU393335.1 Infectious bronchitis virus serotype H120, complete genome	AGTGCCGGGGCCACGCGGAGTACGATCGAGGGTACAGCACT
GU393337.1 Infectious bronchitis virus serotype Iowa 97, complete genome	AGTGCCGAGGCCACGCGGAGTACGATCGAGGGCACAGCACT
GU393338.1 Infectious bronchitis virus serotype JMK, complete genome	AGTGCCGGGGCCACGCGGAGTACGATCGAGGGTACAGCACT
GU553363.1 SARS coronavirus HKU-39849 isolate TCVSP-HARROD-00001, complete genome	TTCATCGAGGCCACGCGGAGTACGATCGAGGGTACAGTGAA
GU553364.1 SARS coronavirus HKU-39849 isolate TCVSP-HARROD-00002, complete genome	TTCATCGAGGCCACGCGGAGTACGATCGAGGGTACAGTGAA
GU553365.1 SARS coronavirus HKU-39849 isolate TCVSP-HARROD-00003, complete genome	TTCATCGAGGCCACGCGGAGTACGATCGAGGGTACAGTGAA
GU732187.1 Human astrovirus 3 isolate Rus-Nsc08-3364 putative serine protease gene, complete cds; putative RNA-(	GAGACCGCGGCCACGCCGAGTAGGATCGAGGGTACAGTCTC
HC086767.1 Sequence 1 from Patent WO2009133054	TTTCCCGAGGCCACGGCGAGTAGCATCGAGGGTACAGGAAA
HI553383.1 Sequence 41 from Patent EP2139515	TTCATCGAGGCCACGCGGAGTACGATCGAGGGTACAGTGAA
HM029238.1 Avian nephritis virus 1 from China, complete genome	TTTCCCGAGGCCACGGCGAATAGCATCGAGGGTACAGGAAA
HM237363.1 Human astrovirus 6 isolate Katano, complete genome	GAAGCCGCGGCCACGCCGAGTAGGATCGAGGGTACAGCTTC

HM245923.1 Infectious bronchitis virus isolate DY07, complete genome	AGTGCCGGGGCCACGCGGAGTACGATCGAGGGTACAGCACT
HM245924.1 Infectious bronchitis virus isolate CQ04-1, complete genome	AGTGCCGAGGCCACGCGGAGTACGATCGAGGGTACAGCACT
HM484374.1 Astrovirus VA1 isolate VS34 ORF1ab and putative serine protease genes, partial cds; and capsid protein	TGCGCCGAGGCCACGCCGAGTAGGACCGAGGGTACAGCGCT
HM756258.1 Astrovirus swine/PoAstV12-3/Canada/2006 RNA polymerase gene, partial cds; and capsid gene, comple	AAAGCCGAGGCCACGCCGAGTAGGAACGAGGGTACAGCTTT
HM756261.1 Astrovirus swine/PoAstV16-2/Canada/2006 RNA polymerase gene, partial cds; and capsid gene, comple	GCCCCCGAGGCCACGCCGAGTAGGAACGAGGGTACAGGGGC
HQ398856.2 Human astrovirus 1 strain Hu/Nyergesujfalu/HUN4520/2010/HUN, complete genome	GAAGCCGCGGCCACGCCGAGTAGGAACGAGGGTACAGCTTC
HQ595344.1 Bat picornavirus 3 strain TLC5F, complete genome	AAGACCGAGGCCACGCGGAGTACGAACGAGGGTACAGTCTT
HQ595345.1 Bat picornavirus 3 strain TLC21F, complete genome	AAGACCGAGGCCACGCGGAGTACGAACGAGGGTACAGTCTT
HQ848267.1 Infectious bronchitis virus isolate GX-YL5, complete genome	AGTGCCGAGGCCACGCGGAGTACGATCGAGGGTACAGCACT
HQ850618.1 Infectious bronchitis virus isolate GX-YL9, complete genome	AGTGCCGGGGCCACGCGGAGTACGATCGAGGGTACAGCACT
HQ889774.1 Pigeon avian nephritis virus strain SH10 non-structural polyprotein and structural polyprotein genes, cor	TTTCCCGAGGCCACGGCGAGTAGCATCGAGGGTACAGGAAA
HQ890526.1 SARS coronavirus MA15 ExoN1 isolate d2ym1, complete genome	TTCATCGAGGCCACGCGGAGTACGATCGAGGGTACAGTGAA
HQ890527.1 SARS coronavirus MA15 ExoN1 isolate d2ym2, complete genome	TTCATCGAGGCCACGCGGAGTACGATCGAGGGTACAGTGAA
HQ890528.1 SARS coronavirus MA15 ExoN1 isolate d2ym3, complete genome	TTCATCGAGGCCACGCGGAGTACGATCGAGGGTACAGTGAA
HQ890529.1 SARS coronavirus MA15 ExoN1 isolate d2ym4, complete genome	TTCATCGAGGCCACGCGGAGTACGATCGAGGGTACAGTGAA
HQ890530.1 SARS coronavirus MA15 ExoN1 isolate d2ym5, complete genome	TTCATCGAGGCCACGCGGAGTACGATCGAGGGTACAGTGAA
HQ890531.1 SARS coronavirus MA15 ExoN1 isolate d4ym1, complete genome	TTCATCGAGGCCACGCGGAGTACGATCGAGGGTACAGTGAA
HQ890532.1 SARS coronavirus MA15 ExoN1 isolate d4ym2, complete genome	TTCATCGAGGCCACGCGGAGTACGATCGAGGGTACAGTGAA
HQ890533.1 SARS coronavirus MA15 ExoN1 isolate d4ym3, complete genome	TTCATCGAGGCCACGCGGAGTACGATCGAGGGTACAGTGAA
HQ890534.1 SARS coronavirus MA15 ExoN1 isolate d2om1, complete genome	TTCATCGAGGCCACGCGGAGTACGATCGAGGGTACAGTGAA
HQ890535.1 SARS coronavirus MA15 ExoN1 isolate d2om2, complete genome	TTCATCGAGGCCACGCGGAGTACGATCGAGGGTACAGTGAA
HQ890536.1 SARS coronavirus MA15 ExoN1 isolate d2om3, complete genome	TTCATCGAGGCCACGCGGAGTACGATCGAGGGTACAGTGAA
HQ890537.1 SARS coronavirus MA15 ExoN1 isolate d2om4, complete genome	TTCATCGAGGCCACGCGGAGTACGATCGAGGGTACAGTGAA
HQ890538.1 SARS coronavirus MA15 ExoN1 isolate d2om5, complete genome	TTCATCGAGGCCACGCGGAGTACGATCGAGGGTACAGTGAA
HQ890539.1 SARS coronavirus MA15 ExoN1 isolate d3om1, complete genome	TTCATCGAGGCCACGCGGAGTACGATCGAGGGTACAGTGAA
HQ890540.1 SARS coronavirus MA15 ExoN1 isolate d3om2, complete genome	TTCATCGAGGCCACGCGGAGTACGATCGAGGGTACAGTGAA
HQ890541.1 SARS coronavirus MA15 isolate d2ym1, complete genome	TTCATCGAGGCCACGCGGAGTACGATCGAGGGTACAGTGAA
HQ890542.1 SARS coronavirus MA15 isolate d2om1, complete genome	TTCATCGAGGCCACGCGGAGTACGATCGAGGGTACAGTGAA
HQ890543.1 SARS coronavirus MA15 isolate d2om2, complete genome	TTCATCGAGGCCACGCGGAGTACGATCGAGGGTACAGTGAA
HQ890544.1 SARS coronavirus MA15 isolate d2om3, complete genome	TTCATCGAGGCCACGCGGAGTACGATCGAGGGTACAGTGAA
HQ890545.1 SARS coronavirus MA15 isolate d2om4, complete genome	TTCATCGAGGCCACGCGGAGTACGATCGAGGGTACAGTGAA
HQ890546.1 SARS coronavirus MA15 isolate d2om5, complete genome	TTCATCGAGGCCACGCGGAGTACGATCGAGGGTACAGTGAA
HV511037.1 JP 2011520430-A/1: Novel avian Astrovirus	TTTCCCGAGGCCACGGCGAGTAGCATCGAGGGTACAGGAAA

HW269828.1 JP 2012105686-A/1: MODIFIED SMALL INTERFERING RNA MOLECULES AND METHODS OF USE	TTCATCGAGGCCACGCGGAGTACGATCGAGGGTACAGTGAA
HW364297.1 JP 2012165750-A/1: NOVEL STRAIN OF SARS-ASSOCIATED CORONAVIRUS AND APPLICATIONS THEREOF	TTCATCGAGGCCACGCGGAGTACGATCGAGGGTACAGTGAA
HW364323.1 JP 2012165750-A/27: NOVEL STRAIN OF SARS-ASSOCIATED CORONAVIRUS AND APPLICATIONS THEREO	TTCATCGAGGCCACGCGGAGTACGATCGAGGGTACAGTGAA
HW364353.1 JP 2012165750-A/57: NOVEL STRAIN OF SARS-ASSOCIATED CORONAVIRUS AND APPLICATIONS THEREO	TTCATCGAGGCCACGCGGAGTACGATCGAGGGTACAGTGAA
HW375992.1 JP 2012255024-A/1: MODIFIED SMALL INTERFERING RNA MOLECULES AND METHODS OF USE	TTCATCGAGGCCACGCGGAGTACGATCGAGGGTACAGTGAA
HZ038027.1 JP 2015091247-A/41: ATTENUATED VIRUSES USEFUL FOR VACCINES	TTCATCGAGGCCACGCGGAGTACGATCGAGGGTACAGTGAA
JA816485.1 Sequence 1 from Patent EP2467393	TCCGCCGAGGCCACGCCGAGTAGGATCGAGGGTACAGCGGA
JA816489.1 Sequence 5 from Patent EP2467393	TGCGCCGAGGCCACGCCGAGTAGGATCGAGGGTACAGGTAG
JF274479.2 Infectious bronchitis virus strain ck/CH/LHLJ/07VII, complete genome	AGTGCCGGGGCCACGCGGAGTACGATCGAGGGTACAGCACT
JF292902.1 SARS coronavirus MA15 ExoN1 isolate d4ym4, complete genome	TTCATCGAGGCCACGCGGAGTACGATCGAGGGTACAGTGAA
JF292903.1 SARS coronavirus MA15 ExoN1 isolate d4ym5, complete genome	TTCATCGAGGCCACGCGGAGTACGATCGAGGGTACAGTGAA
JF292904.1 SARS coronavirus MA15 ExoN1 isolate d3om3, complete genome	TTCATCGAGGCCACGCGGAGTACGATCGAGGGTACAGTGAA
JF292905.1 SARS coronavirus MA15 ExoN1 isolate d3om4, complete genome	TTCATCGAGGCCACGCGGAGTACGATCGAGGGTACAGTGAA
JF292906.1 SARS coronavirus MA15 ExoN1 isolate d3om5, complete genome	TTCATCGAGGCCACGCGGAGTACGATCGAGGGTACAGTGAA
JF292907.1 SARS coronavirus MA15 isolate d2ym2, complete genome	TTCATCGAGGCCACGCGGAGTACGATCGAGGGTACAGTGAA
JF292908.1 SARS coronavirus MA15 isolate d2ym3, complete genome	TTCATCGAGGCCACGCGGAGTACGATCGAGGGTACAGTGAA
JF292909.1 SARS coronavirus MA15 isolate d2ym4, complete genome	TTCATCGAGGCCACGCGGAGTACGATCGAGGGTACAGTGAA
JF292910.1 SARS coronavirus MA15 isolate d2ym5, complete genome	TTCATCGAGGCCACGCGGAGTACGATCGAGGGTACAGTGAA
JF292911.1 SARS coronavirus MA15 isolate d4ym1, complete genome	TTCATCGAGGCCACGCGGAGTACGATCGAGGGTACAGTGAA
JF292912.1 SARS coronavirus MA15 isolate d4ym2, complete genome	TTCATCGAGGCCACGCGGAGTACGATCGAGGGTACAGTGAA
JF292913.1 SARS coronavirus MA15 isolate d4ym3, complete genome	TTCATCGAGGCCACGCGGAGTACGATCGAGGGTACAGTGAA
JF292914.1 SARS coronavirus MA15 isolate d4ym4, complete genome	TTCATCGAGGCCACGCGGAGTACGATCGAGGGTACAGTGAA
JF292916.1 SARS coronavirus MA15 isolate d3om1, complete genome	TTCATCGAGGCCACGCGGAGTACGATCGAGGGTACAGTGAA
JF292917.1 SARS coronavirus MA15 isolate d3om2, complete genome	TTCATCGAGGCCACGCGGAGTACGATCGAGGGTACAGTGAA
JF292918.1 SARS coronavirus MA15 isolate d3om3, complete genome	TTCATCGAGGCCACGCGGAGTACGATCGAGGGTACAGTGAA
JF292919.1 SARS coronavirus MA15 isolate d3om4, complete genome	TTCATCGAGGCCACGCGGAGTACGATCGAGGGTACAGTGAA
JF292920.1 SARS coronavirus MA15 isolate d3om5, complete genome	TTCATCGAGGCCACGCGGAGTACGATCGAGGGTACAGTGAA
JF292921.1 SARS coronavirus wtic-MB isolate c1P1, complete genome	TTCATCGAGGCCACGCGGAGTACGATCGAGGGTACAGTGAA
JF292922.1 SARS coronavirus ExoN1 isolate c5P1, complete genome	TTCATCGAGGCCACGCGGAGTACGATCGAGGGTACAGTGAA
JF327666.1 Human astrovirus strain Pune/063681/India, complete genome	GAAGCCGCGGCCACGCCGAGTAGGAACGAGGGTACAGCTTC
JF330898.1 Infectious bronchitis virus strain ck/CH/LHB/100801, complete genome	AGTGCCGGGGCCACGCGGAGTACGATCGAGGGTACAGCACT
JF330899.1 Infectious bronchitis virus strain ck/CH/LNM/091017, complete genome	AGTGCCGGGGCCACGCGGAGTACGATCGAGGGTACAGCACT
JF491430.1 Human astrovirus 3 isolate Rus-Nsc04-H355 putative serine protease gene, complete cds; putative RNA-d	GAGACCGCGGCCACGCCGAGTAGGATCGAGGGTACAGTCTC

JF705860.1 Duck coronavirus isolate DK/CH/HN/ZZ2004, complete genome	AGTGCCGAGGCCACGCGGAGTACGATCGAGGGTACAGCACT
JF713711.1 Porcine astrovirus 5 strain 33/USA, complete genome	TGAGCCGAGGCCACGCCGAGTAGGACCGAGGGTACAGCTCA
JF732903.1 Infectious bronchitis virus strain Sczy3, complete genome	AGTGCCGGGGCCACGCGGAGTACGATCGAGGGTACAGCACT
JF755422.1 Mouse astrovirus M-52/USA/2008, complete genome	CCTGCCGCGGCCACGCCTAGTCGGAACGAGGGTACAGCAGG
JF828980.1 Infectious bronchitis virus strain ck/CH/LHLJ/100902, complete genome	AGTGCCGGGGCCACGCTGAGGACGACCGAGGGTACAGCACT
JF828981.1 Infectious bronchitis virus strain ck/CH/LDL/101212, complete genome	AGTGCCGGGGCCACGCGGAGTACGATCGAGGGTACAGCACT
JF893452.2 Infectious bronchitis virus isolate YN, complete genome	AGTGCCGGGGCCACGCGGAGTACGATCGAGGGTACAGCACT
JF930689.1 Norovirus dog/FD53/2007/Ita RNA dependent RNA polymerase gene, partial cds; and capsid protein and	TTGACCGCGGCCACGCCGAGTAGGATCGAGGGTACAGTCAG
JF939046.1 Norovirus dog/FD210/2007/Ita RNA dependent RNA polymerase gene, partial cds; and capsid protein and	TTAGCCGCGGCCACGCCGAGTAGGATCGAGGGTACAGCTAA
JN088537.1 Porcine astrovirus 5 isolate CC12 polymerase gene, partial cds; and capsid gene, complete cds	TGAGCCGAGGCCACGCCGAGTAGGATCGAGGGTACAGCTCA
JN193534.1 Canine astrovirus VM-2011 strain GI.E/Dog/ITA/2010/Zoid RNA-dependent polymerase (ORF1b) gene, pa	GTTCCCGAGGCCACGCCGAGTAGGATCGAGGGTACAGGTTC
JN420356.1 California sea lion astrovirus 9 isolate 1234 nonstructural protein gene, complete cds; putative RNA-depe	CAAGCCGAGGCCACGCCGAGTAGGATCGAGGGTACAGCTTG
JN592482.1 Ovine astrovirus OAstV-2/Hungary/2009 nonstructural protein gene, partial cds; and structural protein ge	TGAGCCGAGGCCACGCCGAGTAGGATCGAGGGTACAGCTCT
JN831356.1 Canine picornavirus strain 325F, complete genome	TTGTCCGAGGCCACGCCGAGTAGGATCGAGGGTACAGACTT
JN854286.1 SARS coronavirus HKU-39849 isolate recSARS-CoV HKU-39849, complete genome	TTCATCGAGGCCACGCGGAGTACGATCGAGGGTACAGTGAA
JN887820.1 Human astrovirus 1 strain lhar/2011/kor, complete genome	GAAGCCGCGGCCACGCCGAGTAGGAACGAGGGTACAGCTTC
JQ065042.2 Porcine coronavirus HKU15 strain HKU15-44, complete genome	TATGCCGAGGCCACGCGGAGTACGATCGAGGGTACAGCATA
JQ065043.2 Porcine coronavirus HKU15 strain HKU15-155, complete genome	TATGCCGAGGCCACGCGGAGTACGATCGAGGGTACAGCATA
JQ065044.1 White-eye coronavirus HKU16 strain HKU16-6847, complete genome	TGCACCGAGGCCACGCGGAGTACGATCGAGGGTACAGTGCA
JQ065045.1 Sparrow coronavirus HKU17 strain HKU17-6124, complete genome	TATGCCGAGGCCACGCGGAGTACGATCGAGGGTACAGCATA
JQ065046.1 Magpie-robin coronavirus HKU18 strain HKU18-chu3, complete genome	TGTGCCGAGGCCACGCGGAGTACGATCGAGGGTACAGCACA
JQ065049.1 Common-moorhen coronavirus HKU21 strain HKU21-8295, complete genome	TGAACCGAGGCCACGCGGAGTACGATCGAGGGTACAGTTCA
JQ088078.1 Infectious bronchitis virus strain CK/SWE/0658946/10, complete genome	AGTGCCGGGGCCACGCGGAGTACGATCGAGGGTACAGCACT
JQ316196.1 SARS coronavirus HKU-39849 isolate UOB, complete genome	TTCATCGAGGCCACGCGGAGTACGATCGAGGGTACAGTGAA
JQ403108.1 Human astrovirus 5 isolate DL030, complete genome	GAAGCCGCGGCCACGCCGAGTAGGATCGAGGGTACAGCTTC
JQ408745.1 Murine astrovirus strain TF18LM RNA-dependent RNA polymerase mRNA, partial cds; and capsid protein	AAAGCCGCGGCCACGCCGAGTAGGATCGAGGGTACAGCTTT
JQ916917.1 Bat picornavirus isolate BtPV/BB89-24/Rhi_bla/BGR/2008 polyprotein gene, partial cds	AGAGCCGAGGCCACGCGGAGTACGAACGAGGGTACAGCTCA
JQ916918.1 Bat picornavirus isolate BtPV/BB89-95/Rhi_eur/BGR/2008 polyprotein gene, partial cds	AGAGCCGAGGCCACGCGGAGTACGAACGAGGGTACAGCTCA
JQ916920.1 Bat picornavirus isolate BtPV/BR89-2/Rhi_eur/BGR/2008 polyprotein gene, partial cds	AAAGCCGAGGCCACGCGGAGTACGATCGAGGGTACAGCTTA
JQ977697.1 Infectious bronchitis virus isolate SNU8067, complete genome	AGTGCCGAGGCCACGCGGAGTACGATCGAGGGTACAGCACT
JQ977698.1 Infectious bronchitis virus isolate KM91, complete genome	AGTGCCGAGGCCACGCGGAGTACGATCGAGGGTACAGCACT
JX087963.1 Human astrovirus 2 strain ITA/2002/PA65R/type2c RNA-dependent RNA polymerase (ORF1b) gene, partia	GAGACCGCGGCCACGCCGAGTAGGATGGAGGGTACAGTCTC
JX087964.1 Human astrovirus 2 strain ITA/2009/PR5142/type2d RNA-dependent RNA polymerase (ORF1b) gene, part	GAGACCGCGGCCACGCCGAGTAGGATCGAGGGTACAGTCTC

JX087965.1 Human astrovirus 1 strain ITA/2005/PA124/type1d RNA-dependent RNA polymerase (ORF1b) gene, partia	GAAGCCGCGGCCACGCCGAGTAGGAACGAGGGTACAGCTTC
JX162087.1 SARS coronavirus ExoN1 isolate c5P10, complete genome	TTCATCGAGGCCACGCGGAGTACGATCGAGGGTACAGTGAA
JX163923.1 SARS coronavirus Tor2 isolate Tor2/FP1-10912, complete genome	TTCATCGAGGCCACGCGGAGTACGATCGAGGGTACAGTGAA
JX163924.1 SARS coronavirus Tor2 isolate Tor2/FP1-10851, complete genome	TTCATCGAGGCCACGCGGAGTACGATCGAGGGTACAGTGAA
JX163925.1 SARS coronavirus Tor2 isolate Tor2/FP1-10895, complete genome	TTCATCGAGGCCACGCGGAGTACGATCGAGGGTACAGTGAA
JX163926.1 SARS coronavirus Tor2 isolate Tor2/FP1-10912, complete genome	TTCATCGAGGCCACGCGGAGTACGATCGAGGGTACAGTGAA
JX163927.1 SARS coronavirus Tor2 isolate Tor2/FP1-10851, complete genome	TTCATCGAGGCCACGCGGAGTACGATCGAGGGTACAGTGAA
JX163928.1 SARS coronavirus Tor2 isolate Tor2/FP1-10895, complete genome	TTCATCGAGGCCACGCGGAGTACGATCGAGGGTACAGTGAA
JX195175.1 Infectious bronchitis virus strain ck/CH/LDL/091022, complete genome	AGTGCCGGGGCCACGCGGAGTACGATCGAGGGTACAGCACT
JX195176.1 Infectious bronchitis virus strain ck/CH/LZJ/111113, complete genome	AGTGCCGGGGCCACGCGGAGTACGATCGAGGGTACAGCACT
JX195177.1 Infectious bronchitis virus strain ck/CH/LDL/97I substrain P5, complete genome	AGTGCCGAGGCCACGCGGAGTACGATCGAGGGTACAGCACT
JX195178.1 Infectious bronchitis virus strain ck/CH/LDL/97I substrain P115, complete genome	AGTGCCGAGGCCACGCGGAGTACGATCGAGGGTACAGCACT
JX439643.1 Duck astrovirus 1 strain WF1201, complete genome	GCAGCCGCGGCCACGCCGAGTAGGATCGAGGGTACAGCTGC
JX544743.1 Murine astrovirus strain STL 1, complete genome	AAAGCCGCGGCCACGCCGAGTAGGATCGAGGGTACAGCTTT
JX544744.1 Murine astrovirus strain STL 2, complete genome	AAAGCCGCGGCCACGCCGAGTAGGATCGAGGGTACAGCTTT
JX544745.1 Murine astrovirus strain STL 3, partial genome	AAAGCCGCGGCCACGCCGAGTAGGATCGAGGGTACAGCTTT
JX544746.1 Murine astrovirus strain STL 4, partial genome	AAAGCCGCGGCCACGCCGAGTAGGATCGAGGGTACAGCTTT
JX556691.1 Porcine astrovirus 3 isolate US-MO123, complete genome	GCCCCCGAGGCCACGCCGAGTAGGAACGAGGGTACAGGGGC
JX556693.1 Porcine astrovirus 5 isolate AstV5-US-IA122, complete genome	TGAGCCGAGGCCACGCCGAGTAGGATCGAGGGTACAGCTCA
JX624774.1 Duck astrovirus strain YP2, complete genome	GCAGCCGCGGCCACGCCGAGTAGGATCGAGGGTACAGCTGC
JX684072.1 Porcine astrovirus 5 ORF1b gene, partial cds; and ORF2 gene, complete cds	TGAGCCGAGGCCACGCCGAGTAGGATCGAGGGTACAGCTCA
JX840411.1 Infectious bronchitis virus strain YX10, complete genome	AGTGCCGGGGCCACGCGGAGTACGATCGAGGGTACAGCACT
JX857868.1 Astrovirus VA3 isolate VA3/human/Vellore/28054/2005, complete genome	TGCGCCGAGGCCACGCCGAGTAGGATCGAGGGTACAGCGCT
JX857869.1 Astrovirus VA4 isolate VA4/human/Nepal/s5363, complete genome	ATCCCCGAGGCCACGCCGAGTAGGATCGAGGGTACAGGGAT
JX897900.1 UNVERIFIED: Infectious bronchitis virus isolate GX-NN09032, complete genome	AGTGCCGGGGCCACGCGGAGTACGATCGAGGGTACAGCACT
KC008600.1 Infectious bronchitis virus, complete genome	AGTGCCGGGGCCACGCGGAGTACGATCGAGGGTACAGCACT
KC013541.1 Infectious bronchitis virus strain Ck/CH/LGD/120723, complete genome	AGTGCCGGGGCCACGCGGAGTACGATCGAGGGTACAGCACT
KC119407.1 Infectious bronchitis virus strain Ck/CH/LGD/120724, complete genome	AGTGCCGGGGCCACGCGGAGTACGATCGAGGGTACAGCACT
KC136209.1 Infectious bronchitis virus strain ck/CH/LJL/110302, complete genome	AGTGCCGGGGCCACGCGGAGTACGATCGAGGGTACAGCACT
KC285152.2 Mamastrovirus 1 isolate HAstV-2/RUS/Novosibirsk/Nsc11-N2411/2011, complete genome	GAGACCGCGGCCACGCCGAGTAGGATCGAGGGTACAGTCTC
KC342249.1 Mamastrovirus 1 isolate km1 nonstructural protein and structural protein genes, complete cds	GAAGCCGCGGCCACGCCGAGTAGGATCGAGGGTACAGCTTC
KC506155.1 Infectious bronchitis virus strain ck/CH/LJL/111054, complete genome	AGTGCCGAGGCCACGCGGAGTACGATCGAGGGTACAGCACT
KC609001.1 Murine astrovirus strain BSRI1 nonstructural protein (ORF1a) gene, partial cds; and RNA-dependent RNA	AAAGCCGCGGCCACGCCGAGTAGGATCGAGGGTACAGCTTT

KC692365.1 Fox astrovirus isolate fox 5 non-structural polyprotein gene, partial cds; and RNA-dependent RNA polyme	ACCGCCGCGGCCCCGCCGAGTAGGATCGAGGGTACAGTGGT
KC881005.1 Bat SARS-like coronavirus RsSHC014, complete genome	TTCACCGAGGCCACGCGGAGTACGATCGAGGGTACAGTGAA
KC881006.1 Bat SARS-like coronavirus Rs3367, complete genome	TTCACCGAGGCCACGCGGAGTACGATCGAGGGTACAGTGAA
KC915035.1 Mamastrovirus 1 strain ITA/2003/PA73 RNA-dependent RNA polymerase (ORF1b) gene, partial cds; and c	GGAGCCGCGGCCACGCCGAGTAGGATCGAGGGTACAGCTCC
KF039910.1 Human astrovirus 2 isolate Rus-Nsc05-430, complete genome	GAGACCGCGGCCACGCCGAGTAGGAACGAGGGTACAGTCTC
KF039911.1 Human astrovirus 2 isolate Rus-Nsc06-1029, complete genome	GAGACCGCGGCCACGCCGAGTAGGATCGAGGGTACAGTCTC
KF039912.1 Human astrovirus 4 isolate Rus-Nsc05-623, complete genome	GGAGCCGCGGCCACGCCGAGTAGGATCGAGGGTACAGCTCC
KF039913.1 Human astrovirus 4 isolate Rus-Nsc10-N358, complete genome	GGAGCCGCGGCCACGCCGAGTAGGATCGAGGGTACAGCTCC
KF157967.1 Human astrovirus 5 strain Hu/Budapest/HUN5186/2012/HUN nonstructural protein (ORF1a) and nonstru	GAAGCCGCGGCCACGCCGAGTAGGATCGAGGGTACAGCTTC
KF211475.1 Human astrovirus 1 isolate JZ, complete genome	GAAGCCGCGGCCACGCCGAGTAGGAACGAGGGTACAGCTTC
KF294457.1 SARS-related bat coronavirus isolate Longquan-140 orf1ab polyprotein, spike glycoprotein, envelope prot	TTCACCGAGGCCACGCGGAGTACGATCGAGGGTACAGTGAA
KF367457.1 Bat SARS-like coronavirus WIV1, complete genome	TTCACCGAGGCCACGCGGAGTACGATCGAGGGTACAGTGAA
KF374704.1 Feline astrovirus Viseu, complete genome	AGAGCCGAGGCCACGCCGAGTAGGATCGAGGGTACAGCTCT
KF377577.1 Infectious bronchitis virus strain 4/91 vaccine, complete genome	AGTGCCGGGGCCACGCGGAGTACGATCGAGGGTACAGCACT
KF411040.1 Infectious bronchitis virus strain CK/CH/LLN/111169, complete genome	AGTGCCGGGGCCACGCGGAGTACGATCGAGGGTACAGCACT
KF411041.1 Infectious bronchitis virus strain CK/CH/LGX/091109, complete genome	AGTGCCGGGGCCACGCGGAGTACGATCGAGGGTACAGCACT
KF460437.1 Infectious bronchitis virus isolate VicS-v, complete genome	AGTGCCGGGGCCACGCGGAGTACGATCGAGGGTACAGCACT
KF499111.1 Feline astrovirus 2 strain 1637F, complete genome	AGAGCCGAGGCCACGCCGAGTAGGATCGAGGGTACAGCTCT
KF514388.1 SARS coronavirus wtic-MB strain SARS/VeroE6_lab/USA/WTic_c1.5P20/2010, complete genome	TTCATCGAGGCCACGCGGAGTACGATCGAGGGTACAGTGAA
KF514389.1 SARS coronavirus ExoN1 strain SARS/VeroE6_lab/USA/ExoN1_c8P10/2009, complete genome	TTCATCGAGGCCACGCGGAGTACGATCGAGGGTACAGTGAA
KF514390.1 SARS coronavirus ExoN1 strain SARS/VeroE6_lab/USA/ExoN1_c5.4P20/2010, complete genome	TTCATCGAGGCCACGCGGAGTACGATCGAGGGTACAGTGAA
KF514391.1 SARS coronavirus ExoN1 strain SARS/VeroE6_lab/USA/ExoN1_c5.9P20/2010, complete genome	TTCATCGAGGCCACGCGGAGTACGATCGAGGGTACAGTGAA
KF514392.1 SARS coronavirus wtic-MB strain SARS/VeroE6_lab/USA/WTic_c1.4P20/2010, complete genome	TTCATCGAGGCCACGCGGAGTACGATCGAGGGTACAGTGAA
KF514393.1 SARS coronavirus ExoN1 strain SARS/VeroE6_lab/USA/ExoN1_c5.10P20/2010, complete genome	TTCATCGAGGCCACGCGGAGTACGATCGAGGGTACAGTGAA
KF514394.1 SARS coronavirus wtic-MB strain SARS/VeroE6_lab/USA/WTic_c1P20/2009, complete genome	TTCATCGAGGCCACGCGGAGTACGATCGAGGGTACAGTGAA
KF514395.1 SARS coronavirus ExoN1 strain SARS/VeroE6_lab/USA/ExoN1_c8P1/2009, complete genome	TTCATCGAGGCCACGCGGAGTACGATCGAGGGTACAGTGAA
KF514396.1 SARS coronavirus wtic-MB strain SARS/VeroE6_lab/USA/WTic_c3P10/2009, complete genome	TTCATCGAGGCCACGCGGAGTACGATCGAGGGTACAGTGAA
KF514397.1 SARS coronavirus wtic-MB strain SARS/VeroE6_lab/USA/WTic_c2P10/2009, complete genome	TTCATCGAGGCCACGCGGAGTACGATCGAGGGTACAGTGAA
KF514398.1 SARS coronavirus wtic-MB strain SARS/VeroE6_lab/USA/WTic_c1.10P20/2010, complete genome	TTCATCGAGGCCACGCGGAGTACGATCGAGGGTACAGTGAA
KF514399.1 SARS coronavirus wtic-MB strain SARS/VeroE6_lab/USA/WTic_c1.1P20/2010, complete genome	TTCATCGAGGCCACGCGGAGTACGATCGAGGGTACAGTGAA
KF514400.1 SARS coronavirus wtic-MB strain SARS/VeroE6_lab/USA/WTic_c1.8P20/2010, complete genome	TTCATCGAGGCCACGCGGAGTACGATCGAGGGTACAGTGAA
KF514401.1 SARS coronavirus ExoN1 strain SARS/VeroE6_lab/USA/ExoN1_c5.5P20/2010, complete genome	TTCATCGAGGCCACGCGGAGTACGATCGAGGGTACAGTGAA
KF514402.1 SARS coronavirus ExoN1 strain SARS/VeroE6_lab/USA/ExoN1_c5.6P20/2010, complete genome	TTCATCGAGGCCACGCGGAGTACGATCGAGGGTACAGTGAA

KF514403.1 SARS coronavirus ExoN1 strain SARS/VeroE6_lab/USA/ExoN1_c5.1P20/2010, complete genome	TTCATCGAGGCCACGCGGAGTACGATCGAGGGTACAGTGAA
KF514404.1 SARS coronavirus wtic-MB strain SARS/VeroE6_lab/USA/WTic_c1.9P20/2010, complete genome	TTCATCGAGGCCACGCGGAGTACGATCGAGGGTACAGTGAA
KF514405.1 SARS coronavirus ExoN1 strain SARS/VeroE6_lab/USA/ExoN1_c5.2P20/2010, complete genome	TTCATCGAGGCCACGCGGAGTACGATCGAGGGTACAGTGAA
KF514406.1 SARS coronavirus ExoN1 strain SARS/VeroE6_lab/USA/ExoN1_c13P1/2009, complete genome	TTCATCGAGGCCACGCGGAGTACGATCGAGGGTACAGTGAA
KF514407.1 SARS coronavirus ExoN1 strain SARS/VeroE6_lab/USA/ExoN1_c5.7P20/2010, complete genome	TTCATCGAGGCCACGCGGAGTACGATCGAGGGTACAGTGAA
KF514408.1 SARS coronavirus wtic-MB strain SARS/VeroE6_lab/USA/WTic_c2P1/2009, complete genome	TTCATCGAGGCCACGCGGAGTACGATCGAGGGTACAGTGAA
KF514409.1 SARS coronavirus wtic-MB strain SARS/VeroE6_lab/USA/WTic_c2P20/2009, complete genome	TTCATCGAGGCCACGCGGAGTACGATCGAGGGTACAGTGAA
KF514410.1 SARS coronavirus ExoN1 strain SARS/VeroE6_lab/USA/ExoN1_c8P20/2009, complete genome	TTCATCGAGGCCACGCGGAGTACGATCGAGGGTACAGTGAA
KF514411.1 SARS coronavirus ExoN1 strain SARS/VeroE6_lab/USA/ExoN1_c13P10/2009, complete genome	TTCATCGAGGCCACGCGGAGTACGATCGAGGGTACAGTGAA
KF514412.1 SARS coronavirus ExoN1 strain SARS/VeroE6_lab/USA/ExoN1_c13P20/2009, complete genome	TTCATCGAGGCCACGCGGAGTACGATCGAGGGTACAGTGAA
KF514413.1 SARS coronavirus wtic-MB strain SARS/VeroE6_lab/USA/WTic_c1.6P20/2010, complete genome	TTCATCGAGGCCACGCGGAGTACGATCGAGGGTACAGTGAA
KF514414.1 SARS coronavirus ExoN1 strain SARS/VeroE6_lab/USA/ExoN1_c5P20/2009, complete genome	TTCATCGAGGCCACGCGGAGTACGATCGAGGGTACAGTGAA
KF514415.1 SARS coronavirus wtic-MB strain SARS/VeroE6_lab/USA/WTic_c1.7P20/2010, complete genome	TTCATCGAGGCCACGCGGAGTACGATCGAGGGTACAGTGAA
KF514416.1 SARS coronavirus ExoN1 strain SARS/VeroE6_lab/USA/ExoN1_c5.8P20/2010, complete genome	TTCATCGAGGCCACGCGGAGTACGATCGAGGGTACAGTGAA
KF514417.1 SARS coronavirus ExoN1 strain SARS/VeroE6_lab/USA/ExoN1_c5.3P20/2010, complete genome	TTCATCGAGGCCACGCGGAGTACGATCGAGGGTACAGTGAA
KF514418.1 SARS coronavirus wtic-MB strain SARS/VeroE6_lab/USA/WTic_c3P1/2009, complete genome	TTCATCGAGGCCACGCGGAGTACGATCGAGGGTACAGTGAA
KF514419.1 SARS coronavirus wtic-MB strain SARS/VeroE6_lab/USA/WTic_c1P10/2009, complete genome	TTCATCGAGGCCACGCGGAGTACGATCGAGGGTACAGTGAA
KF514420.1 SARS coronavirus ExoN1 strain SARS/VeroE6_lab/USA/ExoN1_c5P10/2009, complete genome	TTCATCGAGGCCACGCGGAGTACGATCGAGGGTACAGTGAA
KF514421.1 SARS coronavirus wtic-MB strain SARS/VeroE6_lab/USA/WTic_c1.2P20/2010, complete genome	TTCATCGAGGCCACGCGGAGTACGATCGAGGGTACAGTGAA
KF514422.1 SARS coronavirus wtic-MB strain SARS/VeroE6_lab/USA/WTic_c1.3P20/2010, complete genome	TTCATCGAGGCCACGCGGAGTACGATCGAGGGTACAGTGAA
KF514423.1 SARS coronavirus wtic-MB strain SARS/VeroE6_lab/USA/WTic_c3P20/2009, complete genome	TTCATCGAGGCCACGCGGAGTACGATCGAGGGTACAGTGAA
KF569996.1 Rhinolophus affinis coronavirus isolate LYRa11, complete genome	TTCACCGAGGCCACGCGGAGTACGATCGAGGGTACAGTGAA
KF574761.1 Infectious bronchitis virus isolate SDIB821/2012, complete genome	AGTGCCGGGGCCACGCGGAGTACGATCGAGGGTACAGCACT
KF636752.1 Bat Hp-betacoronavirus/Zhejiang2013, complete genome	CACACCGAGGCCACGCCGAGTAGGAACGAGGGTACAGTGTG
KF663559.1 Infectious bronchitis virus isolate ck/CH/IBTZ/2012, complete genome	AGTGCCGGGGCCACGCGGAGTACGATCGAGGGTACAGCACT
KF663560.1 Infectious bronchitis virus isolate ck/CH/IBWF/2007, complete genome	AGTGCCGGGGCCACGCGGAGTACGATCGAGGGTACAGCACT
KF663561.1 Infectious bronchitis virus isolate ck/CH/IBYZ/2011, complete genome	AGTGCCGGGGCCACGCGGAGTACGATCGAGGGTACAGCACT
KF668570.1 Mamastrovirus 1 strain ITA/2012/PR1365 RNA-dependent RNA polymerase (ORF1b) gene, partial cds; an	GAGACCGCGGCCACGCCGAGTAGGATCGAGGGTACAGTCTC
KF668605.1 Infectious bronchitis virus isolate CK/CH/SD09/005, complete genome	AGTGCCGAGGCCACGTGGAGTACGATCGAGGGTACAGCACT
KF696629.1 Infectious bronchitis virus strain Connecticut vaccine, complete genome	AGTGCCGAGGCCACGCGGAGTACGATCGAGGGTACAGCACT
KF753804.1 Duck astrovirus strain SL1, complete genome	GCAGTGGAGGCCACGCCGAGTAGGATCGAGGGTACAACTGC
KF753805.1 Duck astrovirus strain SL2, complete genome	GCAGTGGAGGCCACGCCGAGTAGGATCGAGGGTACAACTGC
KF753806.1 Duck astrovirus strain SL4, complete genome	GCAGTGGAGGCCACGCCGAGTAGGATCGAGGGTACAACTGC

KF753807.1 Duck astrovirus strain SL5, complete genome	GCAGTGGAGGCCACGCCGAGTAGGATCGAGGGTACAACTGC
KF787112.2 Mamastrovirus 3 isolate PAstV-GX1, complete genome	AAAGCCGAGGCCACGCCGAGTAGGATCGAGGGTACAGCTTT
KF859964.1 Human astrovirus BF34, complete genome	GTCGCCGAGGCCACGCCGAGTAGGATCGAGGGTACAGCGAT
KF931628.1 Infectious bronchitis virus isolate VicS-del, complete genome	AGTGCCGGGGCCACGCGGAGTACGATCGAGGGTACAGCACT
KJ020899.1 Duck astrovirus CPH, complete genome	GCAGCCGTGGCCACGCCGAGTAGGATCGAGGGTACAGCTGC
KJ128295.1 Infectious bronchitis virus isolate CK/CH/SD/121220, complete genome	AGTGCCGGGGCCACGCGGAGTACGATCGAGGGTACAGCACT
KJ135013.1 Infectious bronchitis virus isolate IBVUkr27-11, complete genome	AGTGCCGGGGCCACGCGGAGTACGATCGAGGGTACAGCACT
KJ425485.1 Infectious bronchitis virus strain ck/CH/LDL/110931, complete genome	AGTGCCGAGGCCACGCGGAGTACGATCGAGGGTACAGCACT
KJ425486.1 Infectious bronchitis virus strain ck/CH/LDL/120557, complete genome	AGTGCCGGGGCCACGCGGAGTACGATCGAGGGTACAGCACT
KJ425487.1 Infectious bronchitis virus strain ck/CH/LHB/110526, complete genome	AGTGCCGGGGCCACGCGGAGTACGATCGAGGGTACAGCACT
KJ425488.1 Infectious bronchitis virus strain ck/CH/LHB/110825, complete genome	AGTGCCGGGGCCACGCGGAGTACGATCGAGGGTACAGCACT
KJ425489.1 Infectious bronchitis virus strain ck/CH/LHB/111172, complete genome	AGTGCCGGGGCCACGCGGAGTACGATCGAGGGTACAGCACT
KJ425490.1 Infectious bronchitis virus strain ck/CH/LHB/111232, complete genome	AGTGCCGGGGCCACGCGGAGTACGATCGAGGGTACAGCACT
KJ425491.1 Infectious bronchitis virus strain ck/CH/LHB/111268, complete genome	AGTGCCGGGGCCACGCGGAGTACGATCGAGGGTACAGCACT
KJ425492.1 Infectious bronchitis virus strain ck/CH/LHB/120403, complete genome	AGTGCCGGGGCCACGCGGAGTACGATCGAGGGTACAGCACT
KJ425493.1 Infectious bronchitis virus strain ck/CH/LHB/120749, complete genome	AGTGCCGGGGCCACGCGGAGTACGATCGAGGGTACAGCACT
KJ425494.1 Infectious bronchitis virus strain ck/CH/LHB/121024, complete genome	AGTGCCGGGGCCACGCGGAGTACGATCGAGGGTACAGCACT
KJ425495.1 Infectious bronchitis virus strain ck/CH/LHB/121040, complete genome	AGTGCCGGGGCCACGCGGAGTACGATCGAGGGTACAGCACT
KJ425496.1 Infectious bronchitis virus strain ck/CH/LHB/130573, complete genome	AGTGCCGGGGCCACGCGGAGTACGATCGAGGGTACAGCACT
KJ425497.1 Infectious bronchitis virus strain ck/CH/LHB/130598, complete genome	AGTGCCGGGGCCACGCGGAGTACGATCGAGGGTACAGCACT
KJ425498.1 Infectious bronchitis virus strain ck/CH/LHB/130642, complete genome	AGTGCCGGGGCCACGCGGAGTACGATCGAGGGTACAGCACT
KJ425499.1 Infectious bronchitis virus strain ck/CH/LHB/131118, complete genome	AGTGCCGGGGCCACGCGGAGTACGATCGAGGGTACAGCACT
KJ425500.1 Infectious bronchitis virus strain ck/CH/LHB/131132, complete genome	AGTGCCGGGGCCACGCGGAGTACGATCGAGGGTACAGCACT
KJ425501.1 Infectious bronchitis virus strain ck/CH/LHB/131142, complete genome	AGTGCCGGGGCCACGCGGAGTACGATCGAGGGTACAGCACT
KJ425502.1 Infectious bronchitis virus strain ck/CH/LHB/131143, complete genome	AGTGCCGGGGCCACGCGGAGTACGATCGAGGGTACAGCACT
KJ425503.1 Infectious bronchitis virus strain ck/CH/LHLJ/090908, complete genome	AGTGCCGGGGCCACGCGGAGTACGATCGAGGGTACAGCACT
KJ425504.1 Infectious bronchitis virus strain ck/CH/LHLJ/091205, complete genome	AGTGCCGGGGCCACGCGGAGTACGACCGAGGGTACAGCACT
KJ425505.1 Infectious bronchitis virus strain ck/CH/LHLJ/110310, complete genome	AGTGCCGGGGCCACGCGGAGTACGATCGAGGGTACAGCACT
KJ425506.1 Infectious bronchitis virus strain ck/CH/LHLJ/111050, complete genome	AGTGCCGGGGCCACGCGGAGTACGATCGAGGGTACAGCACT
KJ425507.1 Infectious bronchitis virus strain ck/CH/LHLJ/131216, complete genome	AGTGCCGGGGCCACGCGGAGTACGATCGAGGGTACAGCACT
KJ425508.1 Infectious bronchitis virus strain ck/CH/LHN/090909, complete genome	AGTGCCGGGGCCACGCGGAGTACGATCGAGGGTACAGCACT
KJ425509.1 Infectious bronchitis virus strain ck/CH/LJL/121059, complete genome	AGTGCCGGGGCCACGCGGAGTACGATCGAGGGTACAGCACT
KJ425510.1 Infectious bronchitis virus strain ck/CH/LSD/110505, complete genome	AGTGCCGGGGCCACGCGGAGTACGATCGAGGGTACAGCACT

KJ425511.1 Infectious bronchitis virus strain ck/CH/LSD/110529, complete genome	AGTGCCGGGGCCACGCGGAGTACGATCGAGGGTACAGCACT
KJ425512.1 Infectious bronchitis virus strain ck/CH/LSD/110726, complete genome	AGTGCCGGGGCCACGCGGAGTACGATCGAGGGTACAGCACT
KJ435283.1 Infectious bronchitis virus strain ck/CH/LSD/111219, complete genome	AGTGCCGGGGCCACGCGGAGTACGATCGAGGGTACAGCACT
KJ435284.1 Infectious bronchitis virus strain ck/CH/LSD/111241, complete genome	AGTGCCGGGGCCACGCGGAGTACGATCGAGGGTACAGCACT
KJ435285.1 Infectious bronchitis virus strain ck/CH/LSD/121228, complete genome	AGTGCCGGGGCCACGCGGAGTACGATCGAGGGTACAGCACT
KJ435286.1 Infectious bronchitis virus strain ck/CH/LSD/1112150, complete genome	AGTGCCGGGGCCACGCTGAGTACGACCGAGGGTACAGCACT
KJ462462.1 Porcine coronavirus HKU15 strain OH1987, complete genome	TATGCCGAGGCCACGCGGAGTACGATCGAGGGTACAGCATA
KJ473811.1 BtRf-BetaCoV/JL2012, complete genome	TTCACCGAGGCCACGCGGAGTACGATCGAGGGTACAGTGAA
KJ473812.1 BtRf-BetaCoV/HeB2013, complete genome	TTCACCGAGGCCACGCGGAGTACGATCGAGGGTACAGTGAA
KJ473813.1 BtRf-BetaCoV/SX2013, complete genome	TTCACCGAGGCCACGCGGAGTACGATCGAGGGTACAGTGAA
KJ473814.1 BtRs-BetaCoV/HuB2013, complete genome	TTCACCGAGGCCACGCGGAGTACGATCGAGGGTACAGTGAA
KJ481931.1 Deltacoronavirus PDCoV/USA/Illinois121/2014 from USA, complete genome	TATGCCGAGGCCACGCGGAGTACGATCGAGGGTACAGCATA
KJ495991.1 Porcine astrovirus 3 clone PFP-24 ORF1ab gene, partial cds; and ORF2 gene, complete cds	GCCCCCGAGGCCACGCCGAGTAGGAACGAGGGTACAGGGGT
KJ495996.1 Porcine astrovirus 5 clone PFP-33 ORF1ab gene, partial cds; and ORF2 gene, complete cds	TGAGCCGAGGCCACGCCGAGTAGGATCGAGGGTACAGCTCA
KJ495999.1 Porcine astrovirus 3 clone PFP-36 ORF1ab gene, partial cds; and ORF2 gene, complete cds	GCCCCCGAGGCCACGCCGAGTAGGAACGAGGGTACAGGGGT
KJ567050.1 Porcine deltacoronavirus 8734/USA-IA/2014, complete genome	TATGCCGAGGCCACGCGGAGTACGATCGAGGGTACAGCATA
KJ569769.1 Porcine coronavirus HKU15 strain IN2847, complete genome	TATGCCGAGGCCACGCGGAGTACGATCGAGGGTACAGCATA
KJ584355.1 Porcine coronavirus HKU15 strain IL2768, complete genome	TATGCCGAGGCCACGCGGAGTACGATCGAGGGTACAGCATA
KJ584356.1 Porcine coronavirus HKU15 strain SD3424, complete genome	TATGCCGAGGCCACGCGGAGTACGATCGAGGGTACAGCATA
KJ584357.1 Porcine coronavirus HKU15 strain KY4813, complete genome	TATGCCGAGGCCACGCGGAGTACGATCGAGGGTACAGCATA
KJ584358.1 Porcine coronavirus HKU15 strain PA3148, complete genome	TATGCCGAGGCCACGCGGAGTACGATCGAGGGTACAGCATA
KJ584359.1 Porcine coronavirus HKU15 strain NE3579, complete genome	TATGCCGAGGCCACGCGGAGTACGATCGAGGGTACAGCATA
KJ584360.1 Porcine coronavirus HKU15 strain MN3092, partial genome	TATGCCGAGGCCACGCGGAGTACGATCGAGGGTACAGCATA
KJ601777.1 Deltacoronavirus PDCoV/USA/Illinois133/2014 from USA, complete genome	TATGCCGAGGCCACGCGGAGTACGATCGAGGGTACAGCATA
KJ601778.1 Deltacoronavirus PDCoV/USA/Illinois134/2014 from USA, complete genome	TATGCCGAGGCCACGCGGAGTACGATCGAGGGTACAGCATA
KJ601779.1 Deltacoronavirus PDCoV/USA/Illinois136/2014 from USA, complete genome	TATGCCGAGGCCACGCGGAGTACGATCGAGGGTACAGCATA
KJ601780.1 Deltacoronavirus PDCoV/USA/Ohio137/2014 from USA, complete genome	TATGCCGAGGCCACGCGGAGTACGATCGAGGGTACAGCATA
KJ620016.1 Porcine coronavirus HKU15 strain MI6148, complete genome	TATGCCGAGGCCACGCGGAGTACGATCGAGGGTACAGCATA
KJ621030.1 Chicken astrovirus isolate VRDC/CAstV/NZ/VHINP-4 capsid protein (ORF2) gene, complete cds	GCAGCCGCGGCCACGCCGAGTAGGATCGAGGGTACAGCTGC
KJ621031.1 Chicken astrovirus isolate VRDC/CAstV/NZ/VHINP-5 capsid protein (ORF2) gene, complete cds	GCAGCCGCGGCCACGCCGAGTAGGATCGAGGGTACAGCTGC
KJ621032.1 Chicken astrovirus isolate VRDC/CAstV/NZ/VHINP-6 capsid protein (ORF2) gene, complete cds	GCAGCCGCGGCCACGCCGAGTAGGATCGAGGGTACAGCTGC
KJ621033.1 Chicken astrovirus isolate VRDC/CAstV/NZ/VHINP-7 capsid protein (ORF2) gene, complete cds	GCAGCCGCGGCCACGCCGAGTAGGATCGAGGGTACAGCTGC
KJ641693.1 Bat picornavirus isolate BtRh-PicoV/SC2013 polyprotein gene, complete cds	AAGACCGAGGCCACGCGGAGTACGAACGAGGGTACAGTCTT

KJ641694.1 Bat picornavirus isolate BtRs-PicoV/YN2010 polyprotein gene, complete cds	ACAGCCGAGGCCACGCGGAGTACGATCGAGGGTACAGCTGT
KJ641695.1 Bat picornavirus isolate BtRs-PicoV/GD2012 polyprotein gene, partial cds	ACAGCCGAGGCCACGCGGAGTACGATCGAGGGTACAGCTGT
KJ656124.1 Astrovirus VA5 isolate VA5/human/Gambia/102139/2009 putative serine protease gene, complete cds; p	GTCGCCGAGGCCACGCCGAGTAGGATCGAGGGTACAGCGAT
KJ690964.1 Goose coronavirus isolate ACoV/Goose/PL-MW162/2009 3' UTR	AGTGCCGGGGCCACGCGGAGTACGATCGAGGGTACAGCACT
KJ690965.1 Duck coronavirus isolate ACoV/Duck/PL-MW283/2009 3' UTR	AGTGCCGGGGCCACGCGGAGTACGATCGAGGGTACAGCACT
KJ690966.1 Duck coronavirus isolate ACoV/Duck/PL-MW284/2009 3' UTR	AGTGCCGGGGCCACGCGGAGTACGATCGAGGGTACAGCACT
KJ690967.1 Mallard coronavirus isolate ACoV/Mallard/PL-MW345/2009 3' UTR	AGTGCCGAGGCCACGCGGAGTACGATCGAGGGTACAGCACT
KJ690969.1 Bean goose coronavirus isolate ACoV/Bean goose/PL-MW434/2009 3' UTR	AGTGCCGAGGCCACGCGGAGTACGATCGAGGGTACAGCACT
KJ690970.1 Bean goose coronavirus isolate ACoV/Bean goose/PL-MW435/2009 3' UTR	AGTGCCGAGGCCACGCGGAGTACGATCGAGGGTACAGCACT
KJ690971.1 Mallard coronavirus isolate ACoV/Mallard/PL-MW272/2011 3' UTR	AGTGCCGGGGCCACGCGGAGTACGATCGAGGGTACAGCACT
KJ769231.1 Swine deltacoronavirus OhioCVM1/2014, complete genome	TATGCCGAGGCCACGCGGAGTACGATCGAGGGTACAGCATA
KJ920196.1 Astrovirus VA1/HMO-C isolate HMO-CLondon1, partial genome	TGCGCCGAGGCCACGCCGAGTAGGATCGAGGGTACAGCGCT
KJ920197.1 Astrovirus VA1/HMO-C isolate HMO-CLondon2, partial genome	TGCGCCGAGGCCACGCCGAGTAGGATCGAGGGTACAGCGCT
KM012168.1 Porcine coronavirus HKU15 strain Michigan/8977/2014, complete genome	TATGCCGAGGCCACGCGGAGTACGATCGAGGGTACAGCATA
KM017741.1 Feline astrovirus D1 isolate FAstV-D1, complete genome	CCACCCGAGGCCACGCCGAGTAGGATCGAGGGTACAGGTGG
KM017742.1 Mamastrovirus 2 isolate FAstV-D2, complete genome	AGAGCCGAGGCCACGCCGAGTAGGATCGAGGGTACAGCTCT
KM017743.1 Mamastrovirus 2 isolate FAstV-D3, complete genome	AGAGCCGAGGCCACGCCGAGTAGGATCGAGGGTACAGCTCT
KM035759.1 Bovine astrovirus CH13, complete genome	AATCCCGAGGCCACGCCGAGTAGGATCGAGGGTACAGGATT
KM213963.1 Infectious bronchitis virus isolate CK/CH/XDC- 2/2013, complete genome	AGTGCCGAGGCCACGCGGAGTACGATCGAGGGTACAGCACT
KM358468.1 Human astrovirus UK1, complete genome	TGCGCCGAGGCCACGCCGAGTAGGATCGAGGGTACAGCGCT
KM401565.1 Astrovirus PA strain Paris, complete genome	TGCGCCGAGGCCACGCCGAGTAGGACCGAGGGTACAGCGCT
KM454473.1 Duck coronavirus isolate DK/GD/27/2014, complete genome	AGTGCCGGGGCCACGCGGAGTACGATCGAGGGTACAGCACT
KM586818.1 Infectious bronchitis virus isolate P100, complete genome	AGTGCCGAGGCCACGCGGAGTACGATCGAGGGTACAGCACT
KM820765.1 Porcine deltacoronavirus KNU14-04, complete genome	TATGCCGAGGCCACGCGGAGTACGATCGAGGGTACAGCATA
KM985690.1 Avian nephritis virus isolate NSW-1a non-structural polyprotein gene, partial cds; and capsid protein ger	TTTCCCGAGGCCACGGCGAGTAGCATCGAGGGTACAGGAAA
KM985691.1 Avian nephritis virus isolate NSW-1b non-structural polyprotein gene, partial cds; and capsid protein ger	TTTCCCGAGGCCACGGCGAGTAGCATCGAGGGTACAGGAAA
KM985692.1 Avian nephritis virus isolate NSW-3a non-structural polyprotein gene, partial cds; and capsid protein ger	TTTCCCGAGGCCACGGCGAGTAGCATCGAGGGTACAGGAAA
KM985693.1 Avian nephritis virus isolate NSW-3b non-structural polyprotein gene, partial cds; and capsid protein ger	TTTCCCGAGGCCACGGCGAGTAGCATCGAGGGTACAGGAAA
KM985694.1 Avian nephritis virus isolate NSW-4a non-structural polyprotein gene, partial cds; and capsid protein ger	TTTCCCGAGGCCACGGCGAGTAGCATCGAGGGTACAGGAAA
KM985695.1 Avian nephritis virus isolate NSW-4b non-structural polyprotein gene, partial cds; and capsid protein-like	TTTCCCGAGGCCACGGCGAGTAGCATCGAGGGTACAGGAAA
KM985696.1 Avian nephritis virus isolate VIC-3a non-structural polyprotein gene, partial cds; and capsid protein gene	TTTCCCGAGGCCACGGCGAGTAGCATCGAGGGTACAGGAAA
KM985697.1 Avian nephritis virus isolate VIC-3b non-structural polyprotein gene, partial cds; and capsid protein gene	TTTCCCGAGGCCACGGCGAGTAGCATCGAGGGTACAGGAAA
KM985698.1 Avian nephritis virus isolate VIC-3c non-structural polyprotein gene, partial cds; and capsid protein gene	TTTCCCGAGGCCACGGCGAGTAGCATCGAGGGTACAGGAAA

KM985699.1 Avian nephritis virus isolate VIC-5a non-structural polyprotein gene, partial cds; and capsid protein gene	TTTCCCGAGGCCACGGCGAGTAGCATCGAGGGTACAGGAAA
KM985700.1 Avian nephritis virus isolate VIC-5b non-structural polyprotein gene, partial cds; and capsid protein gene	TTTCCCGAGGCCACGGCGAGTAGCATCGAGGGTACAGGAAA
KM985701.1 Avian nephritis virus isolate VIC-5c non-structural polyprotein gene, partial cds; and capsid protein gene	TTTCCCGAGGCCACGGCGAGTAGCATCGAGGGTACAGGAAA
KM985702.1 Avian nephritis virus isolate VIC-6a non-structural polyprotein gene, partial cds; and capsid protein gene	TTTCCCGAGGCCACGGCGAGTAGCATCGAGGGTACAGGAAA
KM985703.1 Avian nephritis virus isolate VIC-6b non-structural polyprotein gene, partial cds; and capsid protein gene	TTTCCCGAGGCCACGGCGAGTAGCATCGAGGGTACAGGAAA
KP036502.1 Infectious bronchitis virus strain ck/CH/LHLJ/140906, complete genome	AGTGCCGGGGCCACGCGGAGTACGATCGAGGGTACAGCACT
KP036503.1 Infectious bronchitis virus strain ck/CH/LHB/121010, complete genome	AGTGCCGGGGCCACGCGGAGTACGATCGAGGGTACAGCACT
KP036504.1 Infectious bronchitis virus strain ck/CH/LHB/130630, complete genome	AGTGCCGGGGCCACGCGGAGTACGATCGAGGGTACAGCACT
KP036505.1 Infectious bronchitis virus strain ck/CH/LJL/130925, complete genome	AGTGCCGGGGCCACGCGGAGTACGATCGAGGGTACAGCACT
KP118880.1 Infectious bronchitis virus strain ck/CH/LHB/130927, complete genome	AGTGCCGGGGCCACGCGGAGTACGATCGAGGGTACAGCACT
KP118881.1 Infectious bronchitis virus strain ck/CH/LBJ/140413, complete genome	AGTGCCGGGGCCACGCGGAGTACGATCGAGGGTACAGCACT
KP118882.1 Infectious bronchitis virus strain ck/CH/LBJ/140402, complete genome	AGTGCCGGGGCCACGCGGAGTACGATCGAGGGTACAGCACT
KP118883.1 Infectious bronchitis virus strain ck/CH/LHB/121041, complete genome	AGTGCCGGGGCCACGCGGAGTACGATCGAGGGTACAGCACT
KP118884.1 Infectious bronchitis virus strain ck/CH/LSD/110851, complete genome	AGTGCCGGGGCCACGCGGAGTACGATCGAGGGTACAGCACT
KP118885.1 Infectious bronchitis virus strain ck/CH/LSD/110857, complete genome	AGTGCCGGGGCCACGCGGAGTACGATCGAGGGTACAGCACT
KP118886.1 Infectious bronchitis virus strain ck/CH/LSD/111235, complete genome	AGTGCCGGGGCCACGCGGAGTACGATCGAGGGTACAGCACT
KP118887.1 Infectious bronchitis virus strain ck/CH/LHB/140532, complete genome	AGTGCCGGGGCCACGCGGAGTACGATCGAGGGTACAGCACT
KP118888.1 Infectious bronchitis virus strain ck/CH/LLN/130102, complete genome	AGTGCCGGGGCCACGCGGAGTACGATCGAGGGTACAGCACT
KP118889.1 Infectious bronchitis virus strain ck/CH/LHB/130575, complete genome	AGTGCCGGGGCCACGCGGAGTACGATCGAGGGTACAGCACT
KP118890.1 Infectious bronchitis virus strain ck/CH/LHB/130578, complete genome	AGTGCCGGGGCCACGCGGAGTACGATCGAGGGTACAGCACT
KP118891.1 Infectious bronchitis virus strain ck/CH/LHLJ/111246, complete genome	AGTGCCGGGGCCACGCGGAGTACGATCGAGGGTACAGCACT
KP118892.1 Infectious bronchitis virus strain ck/CH/LLN/130101, complete genome	AGTGCCGGGGCCACGCGGAGTACGATCGAGGGTACAGCACT
KP118893.1 Infectious bronchitis virus strain ck/CH/LSD/110410, complete genome	AGTGCCGGGGCCACGCGGAGTACGATCGAGGGTACAGCACT
KP118894.1 Infectious bronchitis virus strain ck/CH/LGD/090907, complete genome	AGTGCCGGGGCCACGCGGAGTACGATCGAGGGTACAGCACT
KP343691.1 Infectious bronchitis virus strain ck/CH/LGX/130530, complete genome	AGTGCCGGGGCCACGCGGAGTACGATCGAGGGTACAGCACT
KP404149.1 Canine astrovirus strain Gillingham/2012/UK, complete genome	GTTCCCGAGGCCACGCCGAGTAGGATCGAGGGTACAGGTTC
KP404150.1 Canine astrovirus strain Lincoln/2012/UK, complete genome	GTTCCCGAGGCCACGCCGAGTAGGATCGAGGGTACAGGTTC
KP662631.1 Infectious bronchitis virus isolate ck/ZA/3665/11, complete genome	AGTGCCGGGGCCACGCGGAGTACGATCGAGGGTACAGCACT
KP747574.1 Mamastrovirus 3 isolate AstV-LL-2, complete genome	TGAGCCGAGGCCACGCCGAGTAGGATCGAGGGTACAGCTCA
KP757890.1 Porcine deltacoronavirus isolate CHN-AH-2004, complete genome	TATGCCGAGGCCACGCGGAGCACGATCGAGGGTACAGCATA
KP757891.1 Porcine deltacoronavirus isolate CHN-HB-2014, complete genome	TATGCCGAGGCCACGCGGAGTACGATCGAGGGTACAGCATA
KP757892.1 Porcine deltacoronavirus isolate CHN-JS-2014, complete genome	TATGCCGAGGCCACGCGGAGTACGATCGAGGGTACAGCATA
KP790143.1 Infectious bronchitis virus strain CK/CH/LDL/140520, complete genome	AGTGCCGGGGCCACGCGGAGTACGATCGAGGGTACAGCACT

KP790144.1 Infectious bronchitis virus strain CK/CH/LHLJ/140756, complete genome	AGTGCCGGGGCCACGCGGAGTACGATCGAGGGTACAGCACT
KP790145.1 Infectious bronchitis virus strain CK/CH/LHLJ/141105, complete genome	AGTGCCGGGGCCACGCGGAGTACGATCGAGGGTACAGCACT
KP790146.1 Infectious bronchitis virus strain CK/CH/LHLJ/140901, complete genome	AGTGCCGGGGCCACGCGGAGTACGATCGAGGGTACAGCACT
KP862744.1 Mamastrovirus 1 isolate kor85, complete genome	GAAGCCGCGGCCACGCCGAGTAGGATCGAGGGTACAGCTTC
KP868572.1 Infectious bronchitis virus strain ck/CH/LHLJ/111043, complete genome	AGTGCCGGGGCCACGCGGAGTACGATCGAGGGTACAGCACT
KP868573.1 Infectious bronchitis virus strain CK/CH/LJL/130908, complete genome	AGTGCCGGGGCCACGCGGAGTACGATCGAGGGTACAGCACT
KP886808.1 Bat SARS-like coronavirus YNLF_31C, complete genome	TTCACCGAGGCCACGCGGAGTACGATCGAGGGTACAGTGAA
KP886809.1 Bat SARS-like coronavirus YNLF_34C, complete genome	TTCACCGAGGCCACGCGGAGTACGATCGAGGGTACAGTGAA
KP981395.1 Porcine deltacoronavirus strain USA/IL/2014/026PDV_P11, complete genome	TATGCCGAGGCCACGCGGAGTACGATCGAGGGTACAGCATA
KR131621.1 Porcine deltacoronavirus isolate PDCoV/CHJXNI2/2015, complete genome	TATGCCGAGGCCACGCGGAGTACGATCGAGGGTACAGCATA
KR150443.1 Porcine deltacoronavirus strain USA/Arkansas61/2015, complete genome	TATGCCGAGGCCACGCGGAGTACGATCGAGGGTACAGCATA
KR231009.1 Infectious bronchitis virus strain B1648, complete genome	AGTGCCGGGGCCACGCGGAGTACGATCGAGGGTACAGCACT
KR265847.1 Porcine deltacoronavirus strain USA/Minnesota442/2014, complete genome	TATGCCGAGGCCACGCGGAGTACGATCGAGGGTACAGCATA
KR265848.1 Porcine deltacoronavirus strain USA/Minnesota214/2014, complete genome	TATGCCGAGGCCACGCGGAGTACGATCGAGGGTACAGCATA
KR265849.1 Porcine deltacoronavirus strain USA/Michigan447/2014, complete genome	TATGCCGAGGCCACGCGGAGTACGATCGAGGGTACAGCATA
KR265850.1 Porcine deltacoronavirus strain USA/Michigan448/2014, complete genome	TATGCCGAGGCCACGCGGAGTACGATCGAGGGTACAGCATA
KR265851.1 Porcine deltacoronavirus strain USA/Indiana453/2014, complete genome	TATGCCGAGGCCACGCGGAGTACGATCGAGGGTACAGCATA
KR265852.1 Porcine deltacoronavirus strain USA/Illinois449/2014, complete genome	TATGCCGAGGCCACGCGGAGTACGATCGAGGGTACAGCATA
KR265853.1 Porcine deltacoronavirus strain USA/Minnesota/2013, complete genome	TATGCCGAGGCCACGCGGAGTACGATCGAGGGTACAGCATA
KR265854.1 Porcine deltacoronavirus strain USA/Minnesota454/2014, complete genome	TATGCCGAGGCCACGCGGAGTACGATCGAGGGTACAGCATA
KR265855.1 Porcine deltacoronavirus strain USA/Minnesota455/2014, complete genome	TATGCCGAGGCCACGCGGAGTACGATCGAGGGTACAGCATA
KR265856.1 Porcine deltacoronavirus strain USA/Illinois272/2014, complete genome	TATGCCGAGGCCACGCGGAGTACGATCGAGGGTACAGCATA
KR265857.1 Porcine deltacoronavirus strain USA/Illinois273/2014, complete genome	TATGCCGAGGCCACGCGGAGTACGATCGAGGGTACAGCATA
KR265858.1 Porcine deltacoronavirus strain USA/NorthCarolina452/2014, complete genome	TATGCCGAGGCCACGCGGAGTACGATCGAGGGTACAGCATA
KR265859.1 Porcine deltacoronavirus strain USA/Minnesota159/2014, complete genome	TATGCCGAGGCCACGCGGAGTACGATCGAGGGTACAGCATA
KR265860.1 Porcine deltacoronavirus strain USA/Nebraska209/2014, complete genome	TATGCCGAGGCCACGCGGAGTACGATCGAGGGTACAGCATA
KR265862.1 Porcine deltacoronavirus strain USA/Ohio444/2014, complete genome	TATGCCGAGGCCACGCGGAGTACGATCGAGGGTACAGCATA
KR265863.1 Porcine deltacoronavirus strain USA/Ohio445/2014, complete genome	TATGCCGAGGCCACGCGGAGTACGATCGAGGGTACAGCATA
KR265865.1 Porcine deltacoronavirus strain USA/Iowa459/2014, complete genome	TATGCCGAGGCCACGCGGAGTACGATCGAGGGTACAGCATA
KR608272.1 Infectious bronchitis virus isolate LDT3-A, complete genome	AGTGCCGGGGCCACGCGGAGTACGATCGAGGGTACAGCACT
KR822424.1 European turkey coronavirus 080385d, complete genome	AGTGCCGGGGCCACGCGGAGTACGATCGAGGGTACAGCACT
KR902510.1 Infectious bronchitis virus isolate Ind-TN92-03, complete genome	AGTGCCGGGGCCACGCGGAGTACGACCGAGGGTACAGCACT
KT021234.1 Porcine deltacoronavirus strain CH/SXD1/2015, complete genome	TATGCCGAGGCCACGCGGAGTACGATCGAGGGTACAGCATA

KT203557.1 Infectious bronchitis virus isolate B17, complete genome	AGTGCCGGGGCCACGCGGAGTACGATCGAGGGTACAGCACT
KT245136.1 Norovirus cat/GVI.2/TE/77-13/ITA RNA dependent RNA polymerase gene, partial cds; and capsid protein	CTGACCGCGGTCAAGCCGAGTAGGGTCGAGGGTACAGTCGG
KT266822.1 Porcine deltacoronavirus strain CH/Sichuan/S27/2012, complete genome	TATGCCGAGGCCACGCGGAGTACGATCGAGGGTACAGCATA
KT336560.1 Porcine deltacoronavirus isolate CHN-HN-2014, complete genome	TATGCCGAGGCCACGCGGAGTACGATCGAGGGTACAGCATA
KT381613.1 Porcine coronavirus HKU15 strain OH11846, complete genome	TATGCCGAGGCCACGCGGAGTACGATCGAGGGTACAGCATA
KT444582.1 SARS-like coronavirus WIV16, complete genome	TTCACCGAGGCCACGCGGAGTACGATCGAGGGTACAGTGAA
KT736031.1 Infectious bronchitis virus isolate ck/CH/LDL/150434-I, complete genome	AGTGCCGGGGCCACGCGGAGTACGATCGAGGGTACAGCACT
KT736032.1 Infectious bronchitis virus isolate ckCHLDL150434-II, complete genome	AGTGCCGGGGCCACGCGGAGTACGATCGAGGGTACAGCACT
KT852992.1 Infectious bronchitis virus isolate tl/CH/LDT3/03, complete genome	AGTGCCGGGGCCACGCGGAGTACGATCGAGGGTACAGCACT
KT886453.1 Chicken astrovirus isolate CAstV/Poland/G059/2014 nonstructural polyprotein gene, partial cds; and RNA	GCAGCCGCGGCCACGCCGAGTAGGATCGAGGGTACAGCTGC
KT946726.1 Rodent astrovirus isolate HK-22103F, complete genome	GGAGTCGAGGCCACGCCGAGTAGGATCGAGGGTACAACTCC
KT946727.1 Rodent astrovirus isolate HK-4214F, complete genome	GGAGTCGAGGCCACGCCGAGTAGGATCGAGGGTACAGCTCC
KT946728.1 Rodent astrovirus isolate HK-12111F, complete genome	GGAGTCGAGGCCACGCCGAGTAGGATCGAGGGTACAGCTCC
KT946729.1 Rodent astrovirus isolate HK-13112F, complete genome	GGAGTCGAGGCCACGCCGAGTAGGATCGAGGGTACAGCTCC
KT946730.1 Rodent astrovirus isolate HK-25315A, complete genome	GGAGTCGAGGCCACGCCGAGTAGGATCGAGGGTACAACTCC
KT946731.1 Rodent astrovirus isolate HK-CWRN1RV, complete genome	GGAGTCGAGGCCACGCCGAGTAGGATCGAGGGTACAACTCC
KT946735.1 Rodent astrovirus isolate HN-014, partial genome	GAAGCCGAGGCCACGCCGAGTAGGATCGAGGGTACAGCTCC
KT946736.1 Rodent astrovirus isolate GX-006, complete genome	GAAGCCGAGGCCACGCCGAGTAGGATCGAGGGTACAGCTCC
KT946798.1 Infectious bronchitis virus strain CK/CH/GD/GZ14, complete genome	AGTGCCGGGGCCACGCGGAGTACGATCGAGGGTACAGCACT
KT956903.2 Bovine astrovirus isolate CH15, partial genome	AACCCCGAGGCCACGCCGAGTAGGATCGAGGGTACAGGGTG
KU051641.1 Porcine deltacoronavirus strain PDCoV/Swine/Thailand/S5011/2015, complete genome	TATGCCGAGGCCACGCGGAGTACGATCGAGGGTACAGCATA
KU051649.1 Porcine deltacoronavirus strain PDCoV/Swine/Thailand/S5015L/2015, complete genome	TATGCCGAGGCCACGCGGAGTACGATCGAGGGTACAGCATA
KU182964.1 Bat coronavirus isolate JTMC15, complete genome	TTCACCGAGGCCACGCGGAGTACGATCGAGGGTACAGTGAA
KU253616.1 Infectious bronchitis virus isolate THA20151 S2 protein gene, partial cds; and 3a protein, 3b protein, E pr	AGTGCCGGGGCCACGCGGAGTACGATCGAGGGTACAGCACT
KU253617.1 Infectious bronchitis virus isolate THA40151 S2 protein gene, partial cds; and 3a protein, 3b protein, E pr	AGTGCCGGGGCCACGCGGAGTACGATCGAGGGTACAGCACT
KU253618.1 Infectious bronchitis virus isolate THA50151 S2 protein gene, partial cds; and 3a protein, 3b protein, E pr	AGTGCCGGGGCCACGCGGAGTACGATCGAGGGTACAGCACT
KU253619.1 Infectious bronchitis virus isolate THA90151 S2 protein gene, partial cds; and 3a protein, 3b protein, E pr	AGTGCCGGGGCCACGCGGAGTACGATCGAGGGTACAGCACT
KU253620.1 Infectious bronchitis virus isolate THA80151 S2 protein gene, partial cds; and 3a protein, 3b protein, E pr	AGTGCCGGGGCCACGCGGAGTACGATCGAGGGTACAGCACT
KU253621.1 Infectious bronchitis virus isolate THA371052 S2 protein gene, partial cds; and 3a protein, 3b protein, E p	AGTGCCGGGGCCACGCGGAGTACGATCGAGGGTACAGCACT
KU253622.1 Infectious bronchitis virus isolate THA431252 S2 protein gene, partial cds; and 3a protein, 3b protein, E p	AGTGCCGGGGCCACGCGGAGTACGATCGAGGGTACAGCACT
KU253623.1 Infectious bronchitis virus isolate THA441252 S2 protein gene, partial cds; and 3a protein, 3b protein, E p	AGTGCCGGGGCCACGCGGAGTACGATCGAGGGTACAGCACT
KU253624.1 Infectious bronchitis virus isolate THA470153 S2 protein gene, partial cds; and 3a protein, 3b protein, E p	AGTGCCGGGGCCACGCGGAGTACGATCGAGGGTACAGCACT
KU317090.1 Infectious bronchitis virus strain SAIBK2, complete genome	AGTGCCGGGGCCACGCGGAGTACGATCGAGGGTACAGCACT

KU356856.1 Infectious bronchitis virus strain ck/CH/SCYB/140913, complete genome	AGTGCCGGGGCCACGCGGAGTACGATCGAGGGTACAGCACT
KU361187.1 Infectious bronchitis virus isolate CK/CH/2010/JT-1, complete genome	AGTGCCGAGGCCACGCGGAATACGATCGAGGGTACAGCACT
KU361188.1 Infectious bronchitis virus isolate CK/CH/2014/QL1403, complete genome	AGTGCCGGGGCCACGCGGAGTACGATCGAGGGTACAGCACT
KU556805.1 Infectious bronchitis virus isolate Armidale A3, partial genome	AGTGCCGGGGCCACGCGGAGTACGATCGAGGGTACAGCACT
KU556807.1 Infectious bronchitis virus isolate Ck/Aus/N1/08, partial genome	AGTGCCGTGGCCACGCGGAGTACGATCGAGGGTACAGCACT
KU665558.1 Porcine deltacoronavirus strain CHN-LYG-2014, complete genome	TATGCCGAGGCCACGCGGAGTACGATCGAGGGTACAGCATA
KU871312.1 Canine picornavirus strain 6D, complete genome	TTGTCCGAGGCCACGCCGAGTAGGATCGAGGGTACAGACTT
KU871313.1 Canine picornavirus strain 244F, complete genome	TTGTCCGAGGCCACGCCGAGTAGGATCGAGGGTACAGACTT
KU900738.1 Infectious bronchitis virus strain SNU-8065, complete genome	AGTGCCGGGGCCACGCGGAGTACGATCGAGGGTACAGCACT
KU900739.1 Infectious bronchitis virus strain QIA-03342, complete genome	AGTGCCGAGGCCACGCGGAGTACGATCGAGGGTACAGCACT
KU900740.1 Infectious bronchitis virus strain QIA-KR/D79/05, complete genome	AGTGCCGAGGCCACGCGGAGTACGATCGAGGGTACAGCACT
KU900741.1 Infectious bronchitis virus strain SNU-9106, complete genome	AGTGTCGAGGCCACGCGGAGTACGATCGAGGGTACAGCACT
KU900742.1 Infectious bronchitis virus strain SNU-11045, complete genome	AGTGCCGGGGCCACGCGGAGTACGATCGAGGGTACAGCACT
KU900743.1 Infectious bronchitis virus strain SNU-10043, complete genome	AGTGCCGAGGCCACGCGGAGTACGATCGAGGGTACAGCACT
KU900744.1 Infectious bronchitis virus strain QIA-Q43, complete genome	AGTGCCGAGGCCACGCGGAGTACGATCGAGGGTACAGCACT
KU973692.1 UNVERIFIED: SARS-related coronavirus isolate F46, complete genome	TTCACCGAGGCCACGCGGAGTACGATCGAGGGTACAGTGAA
KU981059.1 Porcine deltacoronavirus strain NH, complete genome	TATGCCGAGGCCACGCGGAGTACGATCGAGGGTACAGCATA
KU981060.1 Porcine deltacoronavirus strain NH isolate passage 0, complete genome	TATGCCGAGGCCACGCGGAGTACGATCGAGGGTACAGCATA
KU981061.1 Porcine deltacoronavirus strain NH isolate passage 5, complete genome	TATGCCGAGGCCACGCGGAGTACGATCGAGGGTACAGCATA
KU981062.1 Porcine deltacoronavirus strain NH isolate passage 10, complete genome	TATGCCGAGGCCACGCGGAGTACGATCGAGGGTACAGCATA
KU984334.1 Porcine deltacoronavirus isolate TT_1115, complete genome	TATGCCGAGGCCACGCGGAGTACGATCGAGGGTACAGCATA
KX022602.1 Porcine deltacoronavirus strain PDCoV/USA/Iowa136/2015, complete genome	TATGCCGAGGCCACGCGGAGTACGATCGAGGGTACAGCATA
KX022603.1 Porcine deltacoronavirus strain PDCoV/USA/Minnesota140/2015, complete genome	TATGCCGAGGCCACGCGGAGTACGATCGAGGGTACAGCATA
KX022604.1 Porcine deltacoronavirus strain PDCoV/USA/Nebraska137/2015, complete genome	TATGCCGAGGCCACGCGGAGTACGATCGAGGGTACAGCATA
KX022605.1 Porcine deltacoronavirus strain PDCoV/USA/Nebraska145/2015, complete genome	TATGCCGAGGCCACGCGGAGTACGATCGAGGGTACAGCATA
KX077987.1 Infectious bronchitis virus strain ck/CH/LDL/150434-III, complete genome	AGTGCCGGGGCCACGCGGAGTACGATCGAGGGTACAGCACT
KX118627.1 Porcine deltacoronavirus isolate P1_16_BTL_0115/PDCoV/2016/Lao, complete genome	TATGCCGAGGCCACGCGGAGTACGATCGAGGGTACAGCATA
KX185056.1 Infectious bronchitis virus strain LD3, complete genome	AGTGCCGAGGCCACGCGGAGTACGATCGAGGGTACAGCACT
KX185057.1 Infectious bronchitis virus strain ck/CH/LHLJ/95I, complete genome	AGTGCCGGGGCCACGCGGAGTACGATCGAGGGTACAGCACT
KX185058.1 Infectious bronchitis virus strain ck/CH/LGS/08I, complete genome	AGTGCCGGGGCCACGCGTAGTACGATCGAGGGTACAGCACT
KX185059.1 Infectious bronchitis virus strain LH1, complete genome	AGTGCCGAGGCCACGCGGAGTACGATCGAGGGTACAGCACT
KX219791.1 Infectious bronchitis virus strain ck/CH/LHLJ/07I, complete genome	AGTGCCGGGGCCACGCGGAGTACGATCGAGGGTACAGCACT
KX219792.1 Infectious bronchitis virus strain ck/CH/LGS/06I, complete genome	AGTGCCGGGGCCACGCGGAGTACGATCGAGGGTACAGCACT

KX219793.1 Infectious bronchitis virus strain ck/CH/LHB/111168, complete genome	AGTGCCGGGGCCACGCGGAGTACGATCGAGGGTACAGCACT
KX219794.1 Infectious bronchitis virus strain ck/CH/LJS/101109, complete genome	AGTGCCGGGGCCACGCGGAGTACGATCGAGGGTACAGCACT
KX219795.1 Infectious bronchitis virus strain ck/CH/LSD/150311, complete genome	AGTGCCGGGGCCACGCGGAGTACGATCGAGGGTACAGCACT
KX219796.1 Infectious bronchitis virus strain ck/CH/LXJ/111265, complete genome	AGTGCCGAGGCCACGCGGAGTACGATCGAGGGTACAGCACT
KX219797.1 Infectious bronchitis virus strain ck/CH/LXJ/02I, complete genome	AGTGCCGGGGCCACGCGGAGTACGATCGAGGGTACAGCACT
KX219798.1 Infectious bronchitis virus strain ck/CH/LSD/120913, complete genome	AGTGCCGGGGCCACGCGGAGTACGATCGAGGGTACAGCACT
KX219799.1 Infectious bronchitis virus strain ck/CH/LSD/130211, complete genome	AGTGCCGGGGCCACGCGGAGTACGATCGAGGGTACAGCACT
KX219800.1 Infectious bronchitis virus strain ck/CH/LSD/130611, complete genome	AGTGCCGAGGCCACGCGGAGTACGATCGAGGGTACAGCACT
KX219801.1 Infectious bronchitis virus strain ck/CH/LSD/120437, complete genome	AGTGCCGGGGCCACGCGGAGTACGATCGAGGGTACAGCACT
KX236000.1 Infectious bronchitis virus strain ck/CH/LSD/110912, complete genome	AGTGCCGGGGCCACGCGGAGTACGATCGAGGGTACAGCACT
KX236001.1 Infectious bronchitis virus strain ck/CH/LSD/03I, complete genome	AGTGCCGGGGCCACGCGGAGTACGATCGAGGGTACAGCACT
KX236002.1 Infectious bronchitis virus strain ck/CH/LSD/111037, complete genome	AGTGCCGGGGCCACGCGGAGTACGATCGAGGGTACAGCACT
KX236003.1 Infectious bronchitis virus strain ck/CH/LSD/111025, complete genome	AGTGCCGGGGCCACGCGGAGTACGATCGAGGGTACAGCACT
KX236004.1 Infectious bronchitis virus strain ck/CH/LHLJ/150701, complete genome	AGTGCCGGGGCCACGCGGAGTACGATCGAGGGTACAGCACT
KX236005.1 Infectious bronchitis virus strain ck/CH/LJL/08-1, complete genome	AGTGCCGGGGCCACGCGGAGTACGATCGAGGGTACAGCACT
KX236006.1 Infectious bronchitis virus strain ck/CH/LSD/100305, complete genome	AGTGCCGAGGCCACGCGGAGTACGATCGAGGGTACAGCACT
KX236007.1 Infectious bronchitis virus strain ck/CH/LSD/100408, complete genome	AGTGCCGGGGCCACGCGGAGTACGATCGAGGGTACAGCACT
KX236008.1 Infectious bronchitis virus strain ck/CH/LDL/130325, complete genome	AGTGCCGGGGCCACGCGGAGTACGATCGAGGGTACAGCACT
KX236009.1 Infectious bronchitis virus strain ck/CH/LSX/130132, complete genome	AGTGCCGGGGCCACGCGGAGTACGATCGAGGGTACAGCACT
KX236010.1 Infectious bronchitis virus strain ck/CH/LSD/100412, complete genome	AGTGCCGAGGCCACGCGGAGTACGATCGAGGGTACAGCACT
KX236011.1 Infectious bronchitis virus strain ck/CH/LSD/091203, complete genome	AGTGCCGGGGCCACGCGGAGTACGATCGAGGGTACAGCACT
KX236012.1 Infectious bronchitis virus strain ck/CH/LSD/091003, complete genome	AGTGCCGGGGCCACGCGGAGTACGATCGAGGGTACAGCACT
KX236013.1 Infectious bronchitis virus strain ck/CH/LSD/120742, complete genome	AGTGCCGGGGCCACGCGGAGTACGATCGAGGGTACAGCACT
KX236014.1 Infectious bronchitis virus strain ck/CH/LHB/120497, complete genome	AGTGCCGGGGCCACGCGGAGTACGATCGAGGGTACAGCACT
KX236015.1 Infectious bronchitis virus strain ck/CH/LDL/140709, complete genome	AGTGCCGGGGCCACGCGGAGTACGATCGAGGGTACAGCACT
KX236016.1 Infectious bronchitis virus strain ck/CH/LHB/090921 complete genome	AGTGCCGGGGCCACGCGGAGTACGATCGAGGGTACAGCACT
KX247127.1 Infectious bronchitis virus strain ck/CH/LHB/111190, complete genome	AGTGCCGGGGCCACGCGGAGTACGATCGAGGGTACAGCACT
KX247128.1 Infectious bronchitis virus strain ck/CH/LHB/130569, complete genome	AGTGCCGGGGCCACGCGGAGTACGATCGAGGGTACAGCACT
KX247129.1 Infectious bronchitis virus strain ck/CH/LHB/130337, complete genome	AGTGCCGGGGCCACGCGGAGTACGATCGAGGGTACAGCACT
KX247130.1 Infectious bronchitis virus strain ck/CH/LHB/120402, complete genome	AGTGCCGGGGCCACGCGGAGTACGATCGAGGGTACAGCACT
KX252772.1 Infectious bronchitis virus strain ck/CH/LSHH/03II, complete genome	AGTGCCGGGGCCACGCGGAGTACGATCGAGGGTACAGCACT
KX252773.1 Infectious bronchitis virus strain ck/CH/LHB/140542, complete genome	AGTGCCGGGGCCACGCGGAGTACGATCGAGGGTACAGCACT
KX252774.1 Infectious bronchitis virus strain ck/CH/LHB/130589, complete genome	AGTGCCGGGGCCACGCGGAGTACGATCGAGGGTACAGCACT

KX252775.1 Infectious bronchitis virus strain ck/CH/LHB/130628, complete genome	AGTGCCGGGGCCACGCGGAGTACGATCGAGGGTACAGCACT
KX252776.1 Infectious bronchitis virus strain ck/CH/LHB/131144, complete genome	AGTGCCGGGGCCACGCGGAGTACGATCGAGGGTACAGCACT
KX252777.1 Infectious bronchitis virus strain ck/CH/LSD/07I, complete genome	AGTGCCGAGGCCACGCGGAGTACGATCGAGGGTACAGCACT
KX252778.1 Infectious bronchitis virus strain ck/CH/LJL/05I, complete genome	AGTGCCGGGGCCACGCGGAGTACGATCGAGGGTACAGCACT
KX252779.1 Infectious bronchitis virus strain ck/CH/LLN/06I, complete genome	AGTGCCGGGGCCACGCGGAGTACGATCGAGGGTACAGCACT
KX252780.1 Infectious bronchitis virus strain ck/CH/LLN/090312, complete genome	AGTGCCGGGGCCACGCGGAGTACGATCGAGGGTACAGCACT
KX252781.1 Infectious bronchitis virus strain ck/CH/LHB/150619, complete genome	AGTGCCGGGGCCACGCGGAGTACGATCGAGGGTACAGCACT
KX252782.1 Infectious bronchitis virus strain ck/CH/LLN/07I, complete genome	AGTGCCGAGGCCACGCGGAGTACGATCGAGGGTACAGCACT
KX252783.1 Infectious bronchitis virus strain ck/CH/LHLJ/090510, complete genome	AGTGCCGGGGCCACGCGGAGTACGATCGAGGGTACAGCACT
KX252784.1 Infectious bronchitis virus strain ck/CH/LLN/090910, complete genome	AGTGCCGGGGCCACGCGGAGTACGATCGAGGGTACAGCACT
KX252785.1 Infectious bronchitis virus strain ck/CH/LSD/100311, complete genome	AGTGCCGGGGCCACGCGGAGTACGATCGAGGGTACAGCACT
KX252786.1 Infectious bronchitis virus strain ck/CH/LLN/120605, complete genome	AGTGCCGGGGCCACGCGGAGTACGATCGAGGGTACAGCACT
KX252787.1 Infectious bronchitis virus strain ck/CH/LLN/131040, complete genome	AGTGCCGGGGCCACGCGGAGTACGATCGAGGGTACAGCACT
KX252788.1 Infectious bronchitis virus strain ck/CH/LHLJ/08-6, complete genome	AGTGCCGAGGCCACGCGGAGTACGATCGAGGGTACAGCACT
KX252789.1 Infectious bronchitis virus strain ck/CH/LHLJ/090605, complete genome	AGTGCCGGGGCCACGCGGAGTACGATCGAGGGTACAGCACT
KX252790.1 Infectious bronchitis virus strain ck/CH/LHLJ/090515, complete genome	AGTGCCGGGGCCACGCGGAGTACGATCGAGGGTACAGCACT
KX252791.1 Infectious bronchitis virus strain ck/CH/LLN/98I, complete genome	AGTGCCGAGGCCACGCGGAGTACGATCGAGGGTACAGCACT
KX258195.1 Avian coronavirus isolate 23/2013, complete genome	AGTGCCGGGGCCACGCGGAGTACGATCGAGGGTACAGCACT
KX259248.1 Infectious bronchitis virus strain ck/CH/LHN/101211, complete genome	AGTGCCGGGGCCACGCGGAGTACGATCGAGGGTACAGCACT
KX259249.1 Infectious bronchitis virus strain ck/CH/LJL/090330, complete genome	AGTGCCGGGGCCACGCGGAGTACGATCGAGGGTACAGCACT
KX259250.1 Infectious bronchitis virus strain ck/CH/LJS/101113, complete genome	AGTGCCGGGGCCACGCGGAGTACGATCGAGGGTACAGCACT
KX259251.1 Infectious bronchitis virus strain ck/CH/LJS/101237, complete genome	AGTGCCGGGGCCACGCGGAGTACGATCGAGGGTACAGCACT
KX259252.1 Infectious bronchitis virus strain ck/CH/LJS/110439, complete genome	AGTGCCGGGGCCACGCGGAGTACGATCGAGGGTACAGCACT
KX259253.1 Infectious bronchitis virus strain ck/CH/LHN/120338, complete genome	AGTGCCGGGGCCACGCGGAGTACGATCGAGGGTACAGCACT
KX259254.1 Infectious bronchitis virus strain ck/CH/LSD/090314, complete genome	AGTGCCGAGGCCACGCGGAGTACGATCGAGGGTACAGCACT
KX259255.1 Infectious bronchitis virus strain ck/CH/LJS/111111, complete genome	AGTGCCGGGGCCACGCGGAGTACGATCGAGGGTACAGCACT
KX259256.1 Infectious bronchitis virus strain ck/CH/LJS/120552, complete genome	AGTGCCGGGGCCACGCGGAGTACGATCGAGGGTACAGCACT
KX259257.1 Infectious bronchitis virus strain ck/CH/LJS/120848, complete genome	AGTGCCGGGGCCACGCGGAGTACGATCGAGGGTACAGCACT
KX260138.1 Equine rhinitis B virus strain 303, complete genome	CTCGCCGAGGCCACGCCGAGTAGGACCGAGGGTACAGCGAG
KX260139.1 Equine rhinitis B virus strain 396, complete genome	CTCGCCGAGGCCACGCCGAGTAGGACCGAGGGTACAGCGAG
KX260140.1 Equine rhinitis B virus strain 421, complete genome	CTCGCCGAGGCCACGCCGAGTAGGACCGAGGGTACAGCGAG
KX260141.1 Equine rhinitis B virus 2 strain 1228, complete genome	CTCGCCGAGGCCACGCCGAGTAGGACCGAGGGTACAGCGAG
KX266901.1 Bovine astrovirus CH13/NeuroS1 isolate 23871, complete genome	AATCCCGAGGCCACGCCGAGTAGGATCGAGGGTACAGGATT

KX266902.1 Bovine astrovirus CH13/NeuroS1 isolate 26730, complete genome	AATCCCGAGGCCACGCCGAGTAGGATCGAGGGTACAGGATT
KX266903.1 Bovine astrovirus CH13/NeuroS1 isolate 26875, complete genome	AATCCCGAGGCCACGCCGAGTAGGATCGAGGGTACAGGATT
KX266904.1 Bovine astrovirus CH13/NeuroS1 isolate 36716, complete genome	AGTCCCGAGGCCACGCCGAGTAGGATCGAGGGTACAGGATT
KX266905.1 Bovine astrovirus CH13/NeuroS1 isolate 23985, complete genome	AGTCCCGAGGCCACGCCGAGTAGGATCGAGGGTACAGGATT
KX266906.1 Bovine astrovirus CH13/NeuroS1 isolate 42799, complete genome	AATCCCGAGGCCACGCCGAGTAGGATCGAGGGTACAGGATT
KX266907.1 Bovine astrovirus CH13/NeuroS1 isolate 43661, complete genome	AATCCCGAGGCCACGCCGAGTAGGATCGAGGGTACAGGATT
KX266908.1 Bovine astrovirus CH13/NeuroS1 isolate 43660, complete genome	AATCCCGAGGCCACGCCGAGTAGGATCGAGGGTACAGGATT
KX272465.1 Infectious bronchitis virus isolate AR251-15, complete genome	AGTGCCGGGGCCACGCGGAGTACGATCGAGGGTACAGCACT
KX275390.1 Infectious bronchitis virus strain ck/CH/LHLJ/141103, complete genome	AGTGCCGGGGCCACGCGGAGTACGATCGAGGGTACAGCACT
KX275391.1 Infectious bronchitis virus strain ck/CH/LHLJ/140927, complete genome	AGTGCCGGGGCCACGCGGAGTACGATCGAGGGTACAGCACT
KX275392.1 Infectious bronchitis virus strain ck/CH/LHLJ/110943, complete genome	AGTGCCGGGGCCACGCGGAGTACGATCGAGGGTACAGCACT
KX275393.1 Infectious bronchitis virus strain ck/CH/LHLJ/130705, complete genome	AGTGCCGGGGCCACGCGGAGTACGATCGAGGGTACAGCACT
KX275394.1 Infectious bronchitis virus strain ck/CH/LSD08-8, complete genome	AGTGCCGGGGCCACGCGGAGTACGATCGAGGGTACAGCACT
KX302860.1 Infectious bronchitis virus strain ck/CH/LSD08-7, complete genome	AGTGCCGAGGCCACGCGGAGTACGATCGAGGGTACAGCACT
KX302861.1 Infectious bronchitis virus strain ck/CH/LJS/131049, complete genome	AGTGCCGGGGCCACGCGGAGTACGATCGAGGGTACAGCACT
KX302862.1 Infectious bronchitis virus strain ck/CH/LJS/101111, complete genome	AGTGCCGGGGCCACGCGGAGTACGATCGAGGGTACAGCACT
KX302863.1 Infectious bronchitis virus strain ck/CH/LSD/090334, complete genome	AGTGCCGAGGCCACGCGGAGTACGATCGAGGGTACAGCACT
KX302864.1 Infectious bronchitis virus strain ck/CH/LSD/09091, complete genome	AGTGCCGGGGCCACGCGGAGTACGATCGAGGGTACAGCACT
KX302865.1 Infectious bronchitis virus strain ck/CH/LJS/131102, complete genome	AGTGCCGGGGCCACGCGGAGTACGATCGAGGGTACAGCACT
KX302866.1 Infectious bronchitis virus strain ck/CH/LJL/04I, complete genome	AGTGCCGGGGCCACGCGGAGTACGATCGAGGGTACAGCACT
KX302867.1 Infectious bronchitis virus strain ck/CH/LJL/150430, complete genome	AGTGTCGGGGCCACGCGGAGTACGATCGAGGGTACAGCACT
KX302868.1 Infectious bronchitis virus strain ck/CH/LHLJ/130744, complete genome	AGTGCCGGGGCCACGCGGAGTACGATCGAGGGTACAGCACT
KX302869.1 Infectious bronchitis virus strain ck/CH/LHLJ/090806, complete genome	AGTGCCGGGGCCACGCGGAGTACGATCGAGGGTACAGCACT
KX302870.1 Infectious bronchitis virus strain ck/CH/LHLJ/130822, complete genome	AGTGCCGGGGCCACGCGGAGTACGATCGAGGGTACAGCACT
KX302871.1 Infectious bronchitis virus strain ck/CH/LHuB/131123, complete genome	AGTGCCGGGGCCACGCGGAGTACGATCGAGGGTACAGCACT
KX302872.1 Infectious bronchitis virus strain ck/CH/LSD/101115, complete genome	AGTGCCGAGGCCACGCGGAGTACGATCGAGGGTACAGCACT
KX302873.1 Infectious bronchitis virus strain ck/CH/LJL/140924, complete genome	AGTGCCGGGGCCACGCGGAGTACGATCGAGGGTACAGCACT
KX302874.1 Infectious bronchitis virus strain ck/CH/LGS/131148, complete genome	AGTGCCGGGGCCACGCGGAGTACGATCGAGGGTACAGCACT
KX302875.1 Infectious bronchitis virus strain ck/CH/LJL/100512, complete genome	AGTGCCGGGGCCACGCGGAGTACGATCGAGGGTACAGCACT
KX348114.1 Infectious bronchitis virus strain ck/CH/LDL/05III, complete genome	AGTGCCGGGGCCACGCGGAGTACGATCGAGGGTACAGCACT
KX348115.1 Infectious bronchitis virus strain ck/CH/LAH/120907, complete genome	AGTGCCGGGGCCACGCGGAGTACGATCGAGGGTACAGCACT
KX348116.1 Infectious bronchitis virus strain ck/CH/LBJ/140514, complete genome	AGTGCCGGGGCCACGCGGAGTACGATCGAGGGTACAGCACT
KX348117.1 Infectious bronchitis virus strain ck/CH/LAH/120721, complete genome	AGTGCCGGGGCCACGCGGAGTACGATCGAGGGTACAGCACT

KX361343.1 Porcine deltacoronavirus isolate P1_13_ST1_0213/PDCoV/0213/Thailand, complete genome	TATGCCGAGGCCACGCGGAGTACGATCGAGGGTACAGCATA
KX361344.1 Porcine deltacoronavirus isolate P2_13_ST2_0313/PDCoV/0213/Thailand, complete genome	TATGCCGAGGCCACGCGGAGTACGATCGAGGGTACAGCATA
KX361345.1 Porcine deltacoronavirus isolate P24_15_NT1_1215/PDCoV/2015/Thailand, complete genome	TATGCCGAGGCCACGCGGAGTACGATCGAGGGTACAGCATA
KX364290.1 Infectious bronchitis virus strain ck/CH/LJL/101150, complete genome	AGTGCCGGGGCCACGCGGAGTACGATCGAGGGTACAGCACT
KX364291.1 Infectious bronchitis virus strain ck/CH/LSD/110409, complete genome	AGTGCCGGGGCCACGCGGAGTACGATCGAGGGTACAGCACT
KX364292.1 Infectious bronchitis virus strain ck/CH/LSD/110739, complete genome	AGTGCCGGGGCCACGCGGAGTACGATCGAGGGTACAGCACT
KX364293.1 Infectious bronchitis virus strain ck/CH/LJL/07III, complete genome	AGTGCCGGGGCCACGCGGAGTACGATCGAGGGTACAGCACT
KX364294.1 Infectious bronchitis virus strain ck/CH/LBJ/140528, complete genome	AGTGCCGGGGCCACGCGGAGTACGATCGAGGGTACAGCACT
KX364295.1 Infectious bronchitis virus strain ck/CH/LBJ/140511, complete genome	AGTGCCGGGGCCACGCGGAGTACGATCGAGGGTACAGCACT
KX364296.1 Infectious bronchitis virus strain ck/CH/LJL/08-9, complete genome	AGTGCCGGGGCCACGCGGAGTACGATCGAGGGTACAGCACT
KX364297.1 Infectious bronchitis virus strain ck/CH/LJL/131006, complete genome	AGTGCCGGGGCCACGCGGAGTACGATCGAGGGTACAGCACT
KX364298.1 Infectious bronchitis virus strain ck/CH/LDL/05II, complete genome	AGTGCCGGGGCCACGCGGAGTACGATCGAGGGTACGGCACT
KX364299.1 Infectious bronchitis virus strain ck/CH/LSD/101223, complete genome	AGTGCCGGGGCCACGCGGAGTACGATCGAGGGTACAGCACT
KX364300.1 Infectious bronchitis virus strain ck/CH/LSD/111218, complete genome	AGTGCCGGGGCCACGCGGAGTACGATCGAGGGTACAGCACT
KX372249.1 Infectious bronchitis virus strain ck/CH/LHLJ/121219, complete genome	AGTGCCGGGGCCACGCGGAGTACGATCGAGGGTACAGCACT
KX372250.1 Infectious bronchitis virus strain ck/CH/LSD/130205, complete genome	AGTGCCGGGGCCACGCGGAGTACGATCGAGGGTACAGCACT
KX375805.1 Infectious bronchitis virus strain ck/CH/LJS/07V, complete genome	AGTGCCGGGGCCACGCGGAGTACGATCGAGGGTACAGCACT
KX375806.1 Infectious bronchitis virus strain ck/CH/LHLJ/110673, complete genome	AGTGCCGGGGCCACGCGGAGTACGATCGAGGGTACAGCACT
KX375807.1 Infectious bronchitis virus strain ck/CH/LHLJ/110836, complete genome	AGTGCCGGGGCCACGCGGAGTACGATCGAGGGTACAGCACT
KX375808.1 Infectious bronchitis virus strain ck/CH/LHLJ/99I, complete genome	AGTGCCGGGGCCACGCGGAGTACGATCGAGGGTACAGCACT
KX389094.1 Infectious bronchitis virus strain ck/CH/LBJ/120481, complete genome	AGTGCCGGGGCCACGCGGAGTACGATCGAGGGTACAGCACT
KX400753.1 Infectious bronchitis virus strain ck/CH/LHB/110123, complete genome	AGTGCCGGGGCCACGCGGAGTACGATCGAGGGTACAGCACT
KX425847.1 Infectious bronchitis virus strain ck/CH/LJL/140734, complete genome	AGTGCCGGGGCCACGCGGAGTACGATCGAGGGTACAGCACT
KX434788.1 Infectious bronchitis virus strain ck/CH/LHLJ/130622, complete genome	AGTGCCGGGGCCACGCGGAGTACGATCGAGGGTACAGCACT
KX434789.1 Infectious bronchitis virus strain ck/CH/LJL/140530, complete genome	AGTGCCGGGGCCACGCGGAGTACGATCGAGGGTACAGCACT
KX434790.1 Infectious bronchitis virus strain ck/CH/LJS/111210, complete genome	AGTGCCGGGGCCACGCGGAGTACGATCGAGGGTACAGCACT
KX443143.2 Porcine deltacoronavirus strain CH-01, complete genome	TATGCCGAGGCCACGCGGAGTACGATCGAGGGTACAGCATA
KX599349.1 Canine astrovirus strain HUN/2012/2, complete genome	GTTCCCGAGGCCACGCCGAGTAGGATCGAGGGTACAGGTTC
KX599350.1 Canine astrovirus strain HUN/2012/6, complete genome	GTTCCCGAGGCCACGCCGAGTAGGATCGAGGGTACAGGTTC
KX599351.1 Canine astrovirus strain HUN/2012/115, complete genome	GTTCCCGGGGCCACGCCGAGTAGGATCGAGGGTACAGGTTC
KX599352.1 Canine astrovirus strain HUN/2012/126, complete genome	GTTCCCGAGGCCACGCCGAGTAGGATCGAGGGTACAGGTTC
KX599353.1 Canine astrovirus strain HUN/2012/135, complete genome	GTTCCCGAGGCCACGCCGAGTAGGATCGAGGGTACAGGTTC
KX599354.1 Canine astrovirus strain HUN/2012/8 ORF1b gene, partial cds; and ORF2 gene, complete cds	CTATCCGCGGCCACGCCGAGTAGGATCGAGGGTACAGATAG

KX640829.1 Infectious bronchitis virus strain ck/CH/LGX/111119, complete genome	AGTGCCGAGGCCACGCGGAGTACGATCGAGGGTACAGCACT
KX668222.1 Infectious bronchitis virus isolate S78/14 spike protein (s), 3a protein (3a), 3b protein (3b), envelope prot	AGTGCCGGGGCCACGCGGAGTACGATCGAGGGTACAGCACT
KX721498.1 Infectious bronchitis virus isolate SCZJ-2, partial genome	AGTGCCGAGGCCACGCGGAGTACGATCGAGGGTACAGCACT
KX756441.1 Canine astrovirus isolate DD1, partial genome	GTTCCCGAGGCCACGCCGAGTAGGATCGAGGGTACAGGTTC
KX834351.1 Porcine deltacoronavirus strain PDCoV/Swine/Vietnam/HaNoi6/2015, complete genome	TATGCCGAGGCCACGCGGAGTACGATCGAGGGTACAGCATA
KX834352.1 Porcine deltacoronavirus strain PDCoV/Swine/Vietnam/Binh21/2015, complete genome	TATGCCGAGGCCACGCGGAGTACGATCGAGGGTACAGCATA
KX884602.1 Hubei tetragnatha maxillosa virus 9 strain arthropodmix14049 hypothetical protein 4 gene, complete cds	AGAAGAGAGGCCACGCCGAGTAGGATCGAGGGTACACTTCA
KX884603.1 Hubei tetragnatha maxillosa virus 9 strain arthropodmix14049 hypothetical protein 5 gene, complete cds	AGAACAGAGGCCACGCCGAGTAGGATCGAGGGTACACTTCA
KX884679.1 Hubei tetragnatha maxillosa virus 9 strain QTM27093 hypothetical protein 4 gene, complete cds	AGAAGAGAGGCCACGCCGAGTAGGATCGAGGGTACACTTCA
KX884680.1 Hubei tetragnatha maxillosa virus 9 strain QTM27093 hypothetical protein 5 gene, complete cds	AGAACGGAGGCCACGCCGAGTAGGATCGAGGGTACACTTCA
KX884707.1 Hubei tetragnatha maxillosa virus 9 strain SSZZ3444 hypothetical protein 4 gene, complete cds	AGAAGAGAGGCCACGCCGAGTAGGATCGAGGGTACACTTCA
KX884708.1 Hubei tetragnatha maxillosa virus 9 strain SSZZ3444 hypothetical protein 5 gene, complete cds	AGAAGAGAGGCCACGCCGAGTAGGATCGAGGGTACACTTCA
KX998969.1 Porcine deltacoronavirus isolate P29_15_VN_1215, complete genome	TATGCCGAGGCCACGCGGAGTACGATCGAGGGTACAGCATA
KY024237.1 Qinghai Himalayan marmot astrovirus 1 isolate HHMAstV1, complete genome	TGAGCCGCGGCCACGCCGAGTAGGATCGAGGGTACAGCTCA
KY024238.1 Qinghai Himalayan marmot astrovirus 2 isolate HHMAstV2, complete genome	CTAGCCGAGGCCACGCCGAGTAGGATCGAGGGTACAGCTAG
KY047602.1 Infectious bronchitis virus isolate gammaCoV/Ck/Poland/G052/2016, complete genome	AGTGCCGGGGCCACGCGGAGTACGATCGAGGGTACAGCACT
KY065120.1 Porcine deltacoronavirus strain CHN/Tianjin/2016, complete genome	TATGCCGAGGCCACGCGGAGTACGATCGAGGGTACAGCATA
KY073229.1 Porcine astrovirus 3 strain NI-Brain/9-2016a/HUN, complete genome	GCCCCCGAGGCCACGCCGAGTAGGAACGAGGGTACAGGGGC
KY073230.1 Porcine astrovirus 3 strain NI-SC/9-2016a/HUN ORF1ab gene, partial cds; and ORF2 gene, complete cds	GCCCCCGAGGCCACGCCGAGTAGGAACGAGGGTACAGGGGC
KY073231.1 Porcine astrovirus 3 strain NI-Brain/173-2016a/HUN, complete genome	GCCCCCGAGGCCACGCCGAGTAGGAACGAGGGTACAGGGGC
KY073232.1 Porcine astrovirus 3 strain NI-Brain/386-2015/HUN, complete genome	GCCCCCGAGGCCACGCCGAGTAGGAACGAGGGTACAGGGGC
KY073233.1 Porcine astrovirus 3 strain Faeces/TM1205-2013/HUN ORF1ab gene, partial cds; and ORF2 gene, complet	GCCCCCGAGGCCACGCCGAGTAGGAACGAGGGTACAGGGGC
KY271945.1 Human astrovirus strain USA/TN/2015-OB2038A, complete genome	GAAGCCGCGGCCACGCCGAGTAGGAACGAGGGTACAGCTTC
KY293677.1 Porcine deltacoronavirus isolate CH/JXJGS01/2016, complete genome	TATGCCGAGGCCACGCGGAGTACGATCGAGGGTACAGCATA
KY293678.1 Porcine deltacoronavirus isolate CH/JXJGS02/2016, complete genome	TATGCCGAGGCCACGCGGAGTACGATCGAGGGTACAGCATA
KY352407.1 Severe acute respiratory syndrome-related coronavirus strain BtKY72, complete genome	TTCGCCGAGGCCACGCGGAGTACGATCGAGGGTACAGTGAA
KY354363.1 Porcine deltacoronavirus isolate DH1, complete genome	TATGCCGAGGCCACGCGGAGTACGATCGAGGGTACAGCATA
KY354364.1 Porcine deltacoronavirus isolate DH2, complete genome	TATGCCGAGGCCACGCGGAGTACGATCGAGGGTACAGCATA
KY363867.1 Porcine deltacoronavirus isolate CHN-GD16-03, complete genome	TATGCCGAGGCCACGCGGAGTACGATCGAGGGTACAGCATA
KY363868.1 Porcine deltacoronavirus isolate CHN-GD16-05, complete genome	TATGCCGAGGCCACGCGGAGTACGATCGAGGGTACAGCATA
KY364365.1 Porcine deltacoronavirus isolate KNU16-07, complete genome	TATGCCGAGGCCACGCGGAGTACGATCGAGGGTACAGCATA
KY407556.1 Infectious bronchitis virus strain gammaCoV/ck/China/I0114/14, complete genome	AGTGCCGGGGCCACGCGGAGTACGATCGAGGGTACAGCACT
KY407557.1 Infectious bronchitis virus strain gammaCoV/ck/China/I0111/14, complete genome	AGTGCCGGGGCCACGCGGAGTACGATCGAGGGTACAGCACT

KY407558.1 Infectious bronchitis virus strain gammaCoV/ck/China/I0118/14, complete genome	AGTGCCGGGGCCACGCGGAGTACGATCGAGGGTACAGCACT
KY417142.1 Bat SARS-like coronavirus isolate As6526, complete genome	TTCACCGAGGCCACGCGGAGTACGATCGAGGGTACAGTGAA
KY417143.1 Bat SARS-like coronavirus isolate Rs4081, complete genome	TTCACCGAGGCCACGCGGAGTACGATCGAGGGTACAGTGAA
KY417144.1 Bat SARS-like coronavirus isolate Rs4084, complete genome	TTCACCGAGGCCACGCGGAGTACGATCGAGGGTACAGTGAA
KY417145.1 Bat SARS-like coronavirus isolate Rf4092, complete genome	TTCACCGAGGCCACGCGGAGTACGATCGAGGGTACAGTGAA
KY417146.1 Bat SARS-like coronavirus isolate Rs4231, complete genome	TTCACCGAGGCCACGCGGAGTACGATCGAGGGTACAGTGAA
KY417147.1 Bat SARS-like coronavirus isolate Rs4237, complete genome	TTCACCGAGGCCACGCGGAGTACGATCGAGGGTACAGTGAA
KY417148.1 Bat SARS-like coronavirus isolate Rs4247, complete genome	TTCACCGAGGCCACGCGGAGTACGATCGAGGGTACAGTGAA
KY417149.1 Bat SARS-like coronavirus isolate Rs4255, complete genome	TTCACCGAGGCCACGCGGAGTACGATCGAGGGTACAGTGAA
KY417150.1 Bat SARS-like coronavirus isolate Rs4874, complete genome	TTCACCGAGGCCACGCGGAGTACGATCGAGGGTACAGTGAA
KY417151.1 Bat SARS-like coronavirus isolate Rs7327, complete genome	TTCACCGAGGCCACGCGGAGTACGATCGAGGGTACAGTGAA
KY417152.1 Bat SARS-like coronavirus isolate Rs9401, complete genome	TTCACCGAGGCCACGCGGAGTACGATCGAGGGTACAGTGAA
KY421672.1 Infectious bronchitis virus isolate SZ, complete genome	AGTGCCGGGGCCACGCGGAGTACGATCGAGGGTACAGCACT
KY421673.1 Infectious bronchitis virus isolate SD, complete genome	AGTGCCGGGGCCACGCGGAGTACGATCGAGGGTACAGCACT
KY432924.1 Picornavirales sp. isolate RtRn-PicoV/YN2014 polyprotein gene, complete cds	CTCACCGAGGCCACGCCGAGTAGGATCGAGGGTACAGTGAG
KY432934.1 Picornavirales sp. isolate RtNn-PicoV/HuB2015-2 polyprotein gene, complete cds	CTCGCCGAGGCCACGCGGAGTACGATCGAGGGTACAGCGAG
KY486328.1 Norovirus GVI strain Dog/5010/2009/ITA nonstructural polyprotein gene, partial cds; and VP1 and VP2 ge	TTAGCCGCGGCCACGCCGAGTAGGATCGAGGGTACAGCTAA
KY486329.1 Norovirus GVI strain 63.15/2015/ITA nonstructural polyprotein gene, partial cds; and VP1 and VP2 genes	TTGACCGCGGCCACGCCGAGTAGGATCGAGGGTACAGTCAA
KY513724.1 Porcine deltacoronavirus strain CH/Hunan/2014, complete genome	TATGCCGAGGCCACGCGGAGTACGATCGAGGGTACAGCATA
KY513725.1 Porcine deltacoronavirus strain CH/Jiangsu/2014, complete genome	TATGCCGAGGCCACGCGGAGTACGATCGAGGGTACAGCATA
KY588134.1 Infectious bronchitis virus isolate Pakistan/Mass/1003/2A/2015, partial genome	AGTGCCGGGGCCACGCGGAGTACGATCGAGGGTACAGCACT
KY588135.1 Infectious bronchitis virus isolate Pakistan/Mass/1009/13A/2015, partial genome	AGTGCCGGGGCCACGCGGAGTACGATCGAGGGTACAGCACT
KY620116.1 Infectious bronchitis virus strain gammaCoV/ck/China/I1101/16, complete genome	AGTGCCGGGGCCACGCGGAGTACGATCGAGGGTACAGCACT
KY626044.1 Avian coronavirus strain BR-I, complete genome	AGTGCCGGGGCCACGCGGAGTACGATCGAGGGTACAGCACT
KY626045.1 Avian coronavirus strain Ma5, complete genome	AGTGCCGGGGCCACGCGGAGTACGATCGAGGGTACAGCACT
KY646154.1 Duck astrovirus 1 isolate HB2015, complete genome	GCAGCCGCGGCCACGCCGAGTAGGATCGAGGGTACAGCTGC
KY744137.1 Human astrovirus 1 strain ITA/2000/PA762 RNA-dependent RNA polymerase (ORF1b) gene, partial cds; a	GAAGCCGCGGCCACGCCGAGTAGGATCGAGGGTACAGCTTC
KY744138.1 Human astrovirus 1 strain ITA/1999/PA364 RNA-dependent RNA polymerase (ORF1b) gene, partial cds; a	GAAGCCGCGGCCACGCCGAGTAGGAACGAGGGTACAGCTTC
KY744139.1 Human astrovirus 1 strain ITA/2004/PA70R- RNA-dependent RNA polymerase (ORF1b) gene, partial cds; a	GAAGCCGCGGCCACGCCGAGTAGGAACGAGGGTACAGCTTC
KY744141.1 Human astrovirus 1 strain ITA/2011/PA387 RNA-dependent RNA polymerase (ORF1b) gene, partial cds; a	GAAGCCGCGGCCACGCCGAGTAGGAACGAGGGTACAGCTTC
KY765684.1 Mamastrovirus 5 strain Crab-eating_fox/2016/BRA, complete genome	GTTCCCGAGGCCACGCCGAGTAGGATCGAGGGTACAGGTTC
KY770858.1 Bat coronavirus isolate Anlong-103, complete genome	TTCACCGAGGCCACGCGGAGTACGATCGAGGGTACAGTGAA
KY770859.1 Bat coronavirus isolate Anlong-112, complete genome	TTCACCGAGGCCACGCGGAGTACGATCGAGGGTACAGTGAA

KY770860.1 Bat coronavirus isolate Jiyuan-84, complete genome	TTCACCGAGGCCACGCGGAGTACGATCGAGGGTACAGTGAA
KY776700.1 Infectious bronchitis virus strain gammaCoV/ck/China/I0712/11, complete genome	AGTGCCGGGGCCACGCGGAGTACGATCGAGGGTACAGCACT
KY776701.1 Infectious bronchitis virus strain gammaCoV/ck/China/I0108/17, complete genome	AGTGCCGGGGCCACGCGGAGTACGATCGAGGGTACAGCACT
KY799582.1 Infectious bronchitis virus strain ck/CH/LSC/99I, complete genome	AGTGCCGGGGCCACGCGGAGTACGATCGAGGGTACAGCACT
KY805845.1 Infectious bronchitis virus isolate IBV/Ck/EG/CU/1/2014, complete genome	AGTGCCGGGGCCACGCGGAGTACGATCGAGGGTACAGCACT
KY805846.1 Infectious bronchitis virus isolate IBV/Ck/EG/CU/4/2014, complete genome	AGTGCCGGGGCCACGCGGAGTACGATCGAGGGTACAGCACT
KY807085.1 Goose astrovirus isolate HN1G, complete genome	GCAGCCGCGGCCACGCCGAGTAGGATCGAGGGTACAGCTGC
KY855433.1 Marmot sapelovirus 2 strain HT6, complete genome	GTAGCCGAGGCCACGCGGAGTACGTTCGAGGGTACAGCTAC
KY855437.1 Marmot astrovirus 1 strain HT10, complete genome	CTAGCCGAGGCCACGCCGAGTAGGATCGAGGGTACAGCTAG
KY855438.1 Marmot astrovirus 2 strain HT11, complete genome	CTAGCCGAGGCCACGCCGAGTAGGATCGAGGGTACAGCTAG
KY855439.1 Marmot astrovirus 3 strain HT12, complete genome	AGAACCGAGGCCACGCCGAGTAGGATCGAGGGTACAGCTAG
KY855440.1 Marmot astrovirus 4 strain HT13, complete genome	AGAACCGAGGCCACGCCGAGTAGGATCGAGGGTACAGCTCA
KY855441.1 Marmot astrovirus 5 strain HT14, complete genome	AGAACCGAGGCCACGCCGAGTAGGATCGAGGGTACAGCTCA
KY855442.1 Marmot astrovirus 6 strain HT15, complete genome	AGAACCGAGGCCACGCCGAGTAGGATCGAGGGTACAGCTCA
KY859988.1 Mamastrovirus 13 strain CH16, complete genome	AATCCCGAGGCCACGCCGAGTAGGATCGAGGGTACAGGGTG
KY926512.1 Porcine deltacoronavirus isolate KNU16-11, complete genome	TATGCCGAGGCCACGCGTAGTACGATCGAGGGTACAGCATA
KY933089.1 Avian coronavirus strain 1148-A, complete genome	AGTGCCGAGGCCACGCGGAGTACGATCGAGGGTACAGCACT
KY933090.1 Avian coronavirus strain L1148, complete genome	AGTGCCGAGGCCACGCGGAGTACGATCGAGGGTACAGCACT
KY933670.1 Astrovirus VA1 non-structural protein 1ab, non-structural protein 1a, and capsid protein genes, complete	TGCGCCGAGGCCACGCCGAGTAGGATCGAGGGTACAGCGCT
KY938558.1 Bat coronavirus strain 16BO133, complete genome	TTCACCGAGGCCACGCGGAGTACGATCGAGGGTACAGTGAA
KY940545.1 Porcine astrovirus 3 strain USA/IA/7023/2017, complete genome	GCCCCCGAGGCCACGCCGAGTAGGAACGAGGGTACAGGGGC
L06251.1 Avian infectious bronchitis virus hypervariable region - related RNA sequence	AGTGCCGAGGCCACGCGGAGTACGATCGAGGGTACAGCACT
L06252.1 Avian infectious bronchitis virus hypervariable region - related RNA sequence	AGTGCCAGGGCCACGCGGAGTACGATCGAGGGTACAGCACC
L06253.1 Avian infectious bronchitis virus hypervariable region - related RNA sequence	AGTGCCGGGGCCACGCGGAGTACGATCGAGGGTACAGCACT
L06802.1 Human astrovirus capsid protein mRNA, complete cds	GAGACCGCGGCCACGCCGAGTAGGATCGAGGGTACAGTCTC
L13745.1 Human astrovirus serotype 2, complete sequence of genomic RNA	GAGACCGCGGCCACGCCGAGTAGGATCGAGGGTACAGTCTC
L23513.1 Human astrovirus type 1 strain Oxford, complete genome	GAAGCCGCGGCCACGCCGAGTAGGAACGAGGGTACAGCTTC
LC047793.1 Bovine astrovirus genomic RNA, nearly complete genome, strain: BoAstV/JPN/Hokkaido12-25/2009	TGAGCCGAGGCCACGCCGAGTAGGATCGAGGGTACAGCTCA
LC201595.1 Porcine astrovirus 3 genes for ORF1ab, ORF1a, ORF2, complete cds, strain: PoAstV3/JPN/Bu2-5/2014	GCCCCCGAGGCCACGCCGAGTAGGAACGAGGGTACAGGGGC
LC201596.1 Porcine astrovirus 3 genes for ORF1ab, ORF1a, ORF2, complete cds, strain: PoAstV3/JPN/Bu4-2-1/2014	GCCCCCGAGGCCACGCCGAGTAGGAACGAGGGTACAGGGGC
LC201597.1 Porcine astrovirus 3 genes for ORF1ab, ORF1a, ORF2, complete cds, strain: PoAstV3/JPN/Bu4-4/2014	GCCCCCGAGGCCACGCCGAGTAGGAACGAGGGTACAGGGGC
LC201598.1 Porcine astrovirus 3 genes for ORF1ab, ORF1a, ORF2, complete cds, strain: PoAstV3/JPN/Bu7-9/2014	TGCCCCGAGGCCACGCCGAGTAGGATCGAGGGTACAGGGCG
LC201615.1 Porcine astrovirus 5 genes for ORF1ab, ORF1a, ORF2, complete cds, strain: PoAstV5/JPN/HgTa2-1-3/2015	TGAGCCGAGGCCACGCCGAGTAGGATCGAGGGTACAGCTCA

LC201616.1 Porcine astrovirus 5 genes for ORF1ab, ORF1a, ORF2, complete cds, strain: PoAstV/JPN/HgTa2-2/2015	TGAGCCGAGGCCACGCCGAGTAGGATCGAGGGTACAGCTCA
LC201617.1 Porcine astrovirus 5 genes for ORF1ab, ORF1a, ORF2, complete cds, strain: PoAstV5/JPN/MoI2-1-3/2015	TGAGCCGAGGCCACGCCGAGTAGGATCGAGGGTACAGCTCA
LC201618.1 Porcine astrovirus 5 genes for ORF1ab, ORF1a, ORF2, complete cds, strain: PoAstV5/JPN/MoI2-3-1/2015	TGAGCCGAGGCCACGCCGAGTAGGATCGAGGGTACAGCTCA
LC201619.1 Porcine astrovirus 5 genes for ORF1ab, ORF1a, ORF2, complete cds, strain: PoAstV5/JPN/Ishi-Im1-1/2015	TGAGCCGAGGCCACGCCGAGTAGGATCGAGGGTACAGCTCA
LC216914.1 Coronavirus HKU15 genomic RNA, complete genome, strain: S579N	TATGCCGAGGCCACGCGGAGTACGATCGAGGGTACAGCATA
LC216915.1 Coronavirus HKU15 genomic RNA, complete genome, strain: S582N	TATGCCGAGGCCACGCGGAGTACGATCGAGGGTACAGCATA
LC260038.1 Porcine deltacoronavirus genomic RNA, complete genome, strain: AKT/JPN/2014	TATGCCGAGGCCACGCGGAGTACGATCGAGGGTACAGCATA
LC260039.1 Porcine deltacoronavirus genomic RNA, complete genome, strain: GNM-1/JPN/2014	TATGCCGAGGCCACGCGGAGTACGATCGAGGGTACAGCATA
LC260040.1 Porcine deltacoronavirus genomic RNA, complete genome, strain: GNM-2/JPN/2014	TATGCCGAGGCCACGCGGAGTACGATCGAGGGTACAGCATA
LC260041.1 Porcine deltacoronavirus genomic RNA, complete genome, strain: IWT/JPN/2014	TATGCCGAGGCCACGCGGAGTACGATCGAGGGTACAGCATA
LC260042.1 Porcine deltacoronavirus genomic RNA, complete genome, strain: MYZ/JPN/2014	TATGCCGAGGCCACGCGGAGTACGATCGAGGGTACAGCATA
LC260043.1 Porcine deltacoronavirus genomic RNA, complete genome, strain: OKN/JPN/2014	TATGCCGAGGCCACGCGGAGTACGATCGAGGGTACAGCATA
LC260044.1 Porcine deltacoronavirus genomic RNA, complete genome, strain: YMG/JPN/2014	TATGCCGAGGCCACGCGGAGTACGATCGAGGGTACAGCATA
LC260045.1 Porcine deltacoronavirus genomic RNA, complete genome, strain: HKD/JPN/2016	TATGCCGAGGCCACGCGGAGCACGATCGAGGGTACAGCATA
LC341267.1 Bovine astrovirus BoAstV/JPN/KagoshimaSR28-462/2016 ORF1ab, ORF1a, ORF2 genes for non-structural	AGTCCCGAGGCCACGCCGAGTAGGATCGAGGGTACAGGATT
LC364342.1 Falcon coronavirus UAE-HKU27 988F genomic RNA, complete genome	TGAACCGAGGCCACGCGGAGTACGATCGAGGGTACAGTTCA
LC364343.1 Houbara coronavirus UAE-HKU28 285F genomic RNA, complete genome	TGAACCGAGGCCACGCGGAGTACGATCGAGGGTACAGTTCA
LC364345.1 Quail coronavirus UAE-HKU30 411F genomic RNA, complete genome	TATGCCGAGGCCACGCGTAGTCCGATCGAGGGTACAGCATA
LC364346.1 Quail coronavirus UAE-HKU30 1101F genomic RNA, complete genome	TATGCCGAGGCCACGCGTAGTCCGATCGAGGGTACAGCATA
LN879482.1 Bovine astrovirus BH89/14, genomic sequence	AATCCCGAGGCCACGCCGAGTAGGATCGAGGGTACAGGATG
LP994923.2 Sequence 1 from Patent WO2017129975	AGTGCCGGGGCCACGCGGAGTACGACCGAGGGTACAGCACT
LQ302036.1 Sequence 1 from Patent WO2016064841	AGTGCCGGGGCCACGCGGAGTACGATCGAGGGTACAGCACT
LT706530.1 Mamastrovirus 13 isolate UK/2014/lamb/lib01455 genome assembly, complete genome: monopartite	AATCCCGAGGCCACGCCGAGTAGGATCGAGGGTACAGGATG
LT706531.1 Mamastrovirus 13 isolate UK/2013/ewe/lib01454 genome assembly, complete genome: monopartite	AATCCCGAGGCCACGCCGAGTAGGATCGAGGGTACAGGATG
LT898424.1 Mamastrovirus 3 isolate PAstV_GER_L00919-K17_14-02_2014 genome assembly, complete genome: mor	GCCCCCGAGGCCACGCCGAGTAGGAACGAGGGTACAGGGGC
LY505956.1 KR 1020170032441-A/1: CORONAVIRUS	AGTGCCGGGGCCACGCGGAGTACGACCGAGGGTACAGCACT
LY611929.1 KR 1020180104088-A/1: ATTENUATED INFECTIOUS BRONCHITIS VIRUS	AGTGCCGGGGCCACGCGGAGTACGACCGAGGGTACAGCACT
M21515.1 Avian infectious bronchitis virus spike, membrane, and nucleocapsid protein genes, complete cds, and fusi	AGTGCCGGGGCCACGCGGAGTACGATCGAGGGTACAGCACT
M28565.1 Avian infectious bronchitis virus (Beaudette) nucleocapsid protein genomic RNA, complete cds	AGTGCCGGGGCCACGCGGAGTACGATCGAGGGTACAGCACT
M28566.1 Avian infectious bronchitis virus (M41) nucleocapsid protein genomic RNA, complete cds	AGTGCCGGGGCCACGCGGAGTACGACCGAGGGTACAGCACT
M87668.1 Human astrovirus type 1 RNA, 3' terminal sequence	GAAGCCGCGGCCACGCCGAGTAGGAACGAGGGTACAGCTTC
M95169.1 Avian infectious bronchitis virus pol protein, spike protein, small virion-associated protein, membrane prot	AGTGCCGGGGCCACGCGGAGTACGATCGAGGGTACAGCACT
MA765825.1 JP 2017522907-A/1: Coronavirus	AGTGCCGGGGCCACGCGGAGTACGACCGAGGGTACAGCACT

MF041982.1 Porcine deltacoronavirus strain SHJS/SL/2016, complete genome	TATGCCGAGGCCACGCGGAGTACGATCGAGGGTACAGCATA
MF095123.1 Porcine deltacoronavirus isolate CHN-HG-2017, complete genome	TATGCCGAGGCCACGCGGAGTACGATCGAGGGTACAGCATA
MF280390.1 Porcine deltacoronavirus strain CHN-GD-2016, complete genome	TATGCCGAGGCCACGCGGAGTACGATCGAGGGTACAGCATA
MF421319.1 Infectious bronchitis virus isolate UY/09/CA/01, complete genome	AGTGCCGGGGCCACGCGGAGTACGATCGAGGGTACAGCACT
MF421320.1 Infectious bronchitis virus isolate UY/11/CA/18, complete genome	AGTGCCGAGGCCACGCGGAGTACGATCGAGGGTACAGCACT
MF431742.1 Porcine deltacoronavirus strain GD, complete genome	TATGCCGAGGCCACGCGGAGTACGATCGAGGGTACAGCATA
MF431743.1 Porcine deltacoronavirus strain SD, complete genome	TATGCCGAGGCCACGCGGAGTACGATCGAGGGTACAGCATA
MF508703.1 Infectious bronchitis virus isolate YX10 D90 vaccine, complete genome	AGTGCCGGGGCCACGCGGAGTACGATCGAGGGTACAGCACT
MF673729.1 Infectious bronchitis virus isolate TC3, partial genome	AGTGCCGGGGCCACGCGGAGTACGATCGAGGGTACAGCACT
MF684776.1 Human astrovirus 5 isolate 2013/Fuzhou/85, complete genome	GAAGCCGCGGCCACGCCGAGTAGGATCGAGGGTACAGCTTC
MF768270.1 Avian astrovirus strain pigeon/China/20/2014 non-structural polyprotein and capsid protein precursor, g	TATACCGAGGCCACGGCGAGTAGCATCGAGGGTACAGTATA
MF772821.1 Goose astrovirus strain AAstV/Goose/CHN/2017/SD01, partial genome	GCAGCCGCGGCCACGCCGAGTAGGATCGAGGGTACAGCTGC
MF882923.1 Infectious bronchitis virus isolate CK/CH/HB/2016, complete genome	AGTGCCGGGGCCACGCGGAGTACGATCGAGGGTACAGCACT
MF924724.1 Infectious bronchitis virus isolate BP-CaKII, complete genome	AGTGCCGAGGCCACGCGGAGTACGATCGAGGGTACAGCACT
MF924725.1 Infectious bronchitis virus isolate K2, complete genome	AGTGCCGAGGCCACGCGGAGTACGATCGAGGGTACAGCACT
MF948005.1 Porcine deltacoronavirus strain HB-BD, complete sequence	TATGCCGAGGCCACGCGGAGTACGATCGAGGGTACAGCATA
MF973500.1 Canine astrovirus strain CHN/2017/44, complete genome	GTTCCCGAGGCCACGCCGAGTAGGATCGAGGGTACAGGTTC
MF973501.1 Canine astrovirus strain CHN/2017/58, complete genome	GTTCCCGAGGCCACGCCGAGTAGGATCGAGGGTACAGGTTC
MG021194.1 Infectious bronchitis virus isolate gammaCoV/AvCov/Ck/Italy/624I/96, complete genome	AGTGCCGGGGCCACGCGGAGTACGATCGAGGGTACAGCACT
MG197727.1 Infectious bronchitis virus isolate CK/CH/GD/QY16, complete genome	AGTGCCGGGGCCACGCGGAGTACGATCGAGGGTACAGCACT
MG242062.1 Porcine deltacoronavirus isolate CHN-HeB1-2017, complete genome	TATGCCGAGGCCACGCGGAGTACGATCGAGGGTACAGCATA
MG448607.1 Infectious bronchitis virus strain ck/CH/LHB/121042, complete genome	AGTGCCGGGGCCACGCGGAGTACGATCGAGGGTACAGCACT
MG517474.1 Infectious bronchitis virus isolate GZ14_F80_vaccine, complete genome	AGTGCCGGGGCCACGCGGAGTACGATCGAGGGTACAGCACT
MG571777.1 Human astrovirus 3 clone V1A nonstructural protein gene, complete cds; nonstructural protein gene, pa	GAGACCGCGGCCTATCCGAGTAGGATCGAGGGTACAGTCTC
MG572714.1 California sea lion norovirus strain Csl/NoV1/PF080916-1 ORF1 polyprotein, VP1 capsid protein, and VP2	ATGACCGAGGCCACGCCGAGTAGGATCGAGGGTACAGTCAG
MG572715.1 California sea lion norovirus strain Csl/NoV2/PF080916-2 ORF1 polyprotein gene, partial cds; and VP1 ca	TGAGCCGCGGCCACGCCGAGTAGGATCGAGGGTACAGCTCA
MG572716.1 California sea lion norovirus strain Csl/NoV2/PF090207 ORF1 polyprotein gene, partial cds; and VP1 cap	TGAGCCGCGGCCACGCCGAGTAGGATCGAGGGTACAGCTCA
MG693176.1 Bat astrovirus isolate CMR/Bat-AsV/P02 ORF1a gene, partial cds; and ORF1b and ORF2 genes, complete	GCAGCCGCGGCCACGCCGAGTAGGATCGAGGGTACAGGAAA
MG738154.1 Infectious bronchitis virus isolate IBS037A/2014, complete genome	AGTGCCGGGGCCACGCGGAGTACGATCGAGGGTACAGCACT
MG738155.1 Infectious bronchitis virus isolate IBS130/2015, complete genome	AGTGCCGGGGCCACGCGGAGTACGATCGAGGGTACAGCACT
MG763935.1 Infectious bronchitis virus strain IBV/Chicken/Haryana/53/2013, complete genome	AGTGCCGGGGCCACGCGGAGTACGATCGAGGGTACAGCACT
MG772933.1 Bat SARS-like coronavirus isolate bat-SL-CoVZC45, complete genome	TTCACCGAGGCCACGCGGAGTACGATCGAGGGTACAGCCAA
MG772934.1 Bat SARS-like coronavirus isolate bat-SL-CoVZXC21, complete genome	CTCACCGAGGCCACGCGGAGTACGATCGAGGGTACAGTGAA

MG812375.1 Sparrow deltacoronavirus strain ISU690-4, complete genome	TATGCCGAGGCCACGCGGAGTACGATCGAGGGTACAGCATA
MG812376.1 Sparrow deltacoronavirus strain ISU690-7, complete genome	TATGCCGAGGCCACGCGGAGTACGATCGAGGGTACAGCATA
MG812377.1 Sparrow deltacoronavirus strain ISU42824, complete genome	TATGCTGAGGCCACGCGGAGTACGATCGAGGGTACAGCATA
MG832584.1 Porcine deltacoronavirus isolate CHN-HN-1601, complete genome	TATGCCGAGGCCACGCGGAGTACGATCGAGGGTACAGCATA
MG837130.1 Porcine deltacoronavirus isolate KNU16-07-P5, complete genome	TATGCCGAGGCCACGCGGAGTACGATCGAGGGTACAGCATA
MG837131.1 Porcine deltacoronavirus isolate KNU16-07-P10, complete genome	TATGCCGAGGCCACGCGGAGTACGATCGAGGGTACAGCATA
MG837132.1 Porcine deltacoronavirus isolate KNU16-07-P20, complete genome	TATGCCGAGGCCACGCGGAGTACGATCGAGGGTACAGCATA
MG837133.1 Porcine deltacoronavirus isolate KNU16-07-P30, complete genome	TATGCCGAGGCCACGCGGAGTACGATCGAGGGTACAGCATA
MG846414.1 Avian nephritis virus capsid protein gene, partial cds	TTTCCCGAGGCCACGGCGAGTAGCATCGAGGGTACAGGAAA
MG846415.1 Avian nephritis virus non-structural polyprotein gene, partial cds; and capsid protein gene, complete cds	TTTCCCGAGGCCACGGCGAGTAGCATCGAGGGTACAGGAAA
MG846416.1 Chicken astrovirus strain RS/BR/15/6R-3 capsid protein gene, partial cds	GCAGCCGCGGCCACGCCGAGTAGGATCGAGGGTACAGCTGC
MG846418.1 Chicken astrovirus strain RS/BR/15/4R-1 capsid protein gene, complete cds	GCAGCCGCGGCCACGCCGAGTAGGATCGAGGGTACAGCTGC
MG882764.1 Goose astrovirus strain AHCZ2 ORF1b gene, partial cds; and ORF2 gene, complete cds	GCAGCCGCGGCCACGCCGAGTAGGATCGAGGGTACAGCTGC
MG882765.1 Goose astrovirus strain AHCZ4 ORF1b gene, partial cds; and ORF2 gene, complete cds	GCAGCCGCGGCCACGCCGAGTAGGATCGAGGGTACAGCTGC
MG882766.1 Goose astrovirus strain JSCZ4 ORF1b gene, partial cds; and ORF2 gene, complete cds	GCAGCCGCGGCCACGCCGAGTAGGATCGAGGGTACAGCTGC
MG913342.1 Avian coronavirus isolate AvCoV/Gallus gallus/Brazil/sample 38/2013 GI-11, complete genome, complet	AGTGCCGGGGCCACGCGGAGTACGATCGAGGGTACAGCACT
MG913343.1 Avian coronavirus isolate AvCoV/Gallus gallus/Brazil/sample 22/2013, complete genome	AGTGCCGGGGCCACGCGGAGTACGATCGAGGGTACAGCACT
MG921619.1 Human astrovirus isolate Yu/1-CHN, complete genome	GAAGCCGCGGCCACGCCGAGTAGGATCGAGGGTACAGCTTC
MG934571.1 Goose astrovirus isolate GD, complete genome	GCAGCCGCGGCCACGCCGAGTAGGATCGAGGGTACAGCTGC
MH020185.1 Infectious bronchitis virus isolate CK/CH/HD/171018, complete genome	AGTGCCGGGGCCACGCGGAGTACGATCGAGGGTACAGCACT
MH021175.1 Avian coronavirus strain D274, complete genome	AGTGCCGGGGCCACGCGGAGTACGATCGAGGGTACAGCACT
MH025762.1 Porcine deltacoronavirus strain CH/JXJGS01/P7, complete genome	TATGCCGAGGCCACGCGGAGTACGATCGAGGGTACAGCATA
MH025763.1 Porcine deltacoronavirus strain CH/JXJGS01/P20, complete genome	TATGCCGAGGCCACGCGGAGTACGATCGAGGGTACAGCATA
MH025764.1 Porcine deltacoronavirus strain CH/JXJGS01/P50, complete genome	TATGCCGAGGCCACGCGGAGTACGATCGAGGGTACAGCATA
MH028405.1 Avian nephritis virus 2 strain AVE52/ANV2, complete genome	TTTCCCGAGGCCACGGCGAGTAGCATCGAGGGTACAGGAAA
MH052598.1 Goose astrovirus strain AstV/SDPY/Goose/1116/17, complete genome	GCAGCCGCGGCCACGCCGAGTAGGATCGAGGGTACAGCTGC
MH181793.1 Infectious bronchitis virus strain HH06, complete genome	AGTGCCGAGGCCACGCGGAGTCCGATGGCGGGTCCAGCATT
MH453800.1 Avastrovirus 2 isolate AstV/Red-necked Avocet/MW08/Interior genomic sequence	CGTACCGAGGCCACGGCGAGCAGCATCGAGGGTACAGTACG
MH532440.1 Quail deltacoronavirus strain G032/2015, complete genome	TATGCCGAGGCCACGCGTAGTCCGATCGAGGGTACAGCATA
MH539771.1 Infectious bronchitis virus isolate IBV_SES_15AB-01, complete genome	AGTGCCGGGGCCACGCGGAGTACGACCGAGGGTACAGCACT
MH539772.1 Infectious bronchitis virus isolate IBV_SES_15SK-02, complete genome	AGTGCCGGGGCCACGCGGAGTACGACCGAGGGTACAGCACT
MH708123.1 Porcine deltacoronavirus strain HNZK-02, complete genome	TATGCCGAGGCCACGCGGAGTACGATCGAGGGTACAGCATA
MH708124.1 Porcine deltacoronavirus strain HNZK-04, complete genome	TATGCCGAGGCCACGCGGAGTACGATCGAGGGTACAGCATA

MH708125.1 Porcine deltacoronavirus strain HNZK-06, complete genome	TATGCCGAGGCCACGCGGAGTACGATCGAGGGTACAGCATA
MH712856.1 Duck astrovirus 1 strain D51, complete genome	GCAGCCGCGGCCACGCCGAGTAGGATCGAGGGTACAGCTGC
MH715491.1 Porcine deltacoronavirus strain PDCoV/CHGD/2016, complete genome	TATGCCGAGGCCACGCGGAGTACGATCGAGGGTACAGCATA
MH779856.1 Infectious bronchitis virus isolate ArkGA_P1, complete genome	AGTGCCGAGGCCACGCGGAGTACGATCGAGGGTACAGCACT
MH779857.1 Infectious bronchitis virus isolate ArkGA_P20, complete genome	AGTGCCGAGGCCACGCGGAGTACGATCGAGGGTACAGCACT
MH779858.1 Infectious bronchitis virus isolate ArkGA_P40, complete genome	AGTGCCGAGGCCACGCGGAGTACGATCGAGGGTACAGCACT
MH779859.1 Infectious bronchitis virus isolate ArkGA_P60, complete genome	AGTGCCGAGGCCACGCGGAGTACGATCGAGGGTACAGCACT
MH779860.1 Infectious bronchitis virus isolate Ark99_pathogenic_field_virus, complete genome	AGTGCCGAGGCCACGCGGAGTACGATCGAGGGTACAGCACT
MH807626.1 Goose astrovirus strain AstV/Goose/CXZ/18, complete genome	GCAGCCGCGGCCACGCCGAGTAGGATCGAGGGTACAGCTGC
MH878976.1 Infectious bronchitis virus isolate VFAR-047, complete genome	AGTGCCGGGGCCACGCGGAGTACGATCGAGGGTACAGCACT
MH924835.1 Infectious bronchitis virus strain gammaCoV/ck/China/I0636/16, complete genome	AGTGCCGGGGCCACGCGGAGTACGATCGAGGGTACAGCACT
MH933752.1 Human astrovirus isolate CMRHP2 nonstructural protein and capsid protein genes, complete cds	GAAGCCGCGGCCACGCCGAGTAGGATCGAGGGTACAGCTTC
MH933753.1 Human astrovirus isolate CMRHP3 nonstructural protein and capsid protein genes, complete cds	GAAGCCGCGGCCACGCCGAGTAGGATCGAGGGTACAGCTTC
MH933754.1 Human astrovirus isolate CMRHP6 nonstructural protein gene, partial cds; and nonstructural protein an	TGCGCCGAGGCCACGCCGAGTAGGATCGAGGGTACAGCGCT
MH933757.1 Human astrovirus isolate CMRHP35D nonstructural protein and capsid protein genes, complete cds	GAAGCCGCGGCCACGCCGAGTAGGATCGAGGGTACAGCTTC
MH933758.1 Human astrovirus isolate CMRHP34 nonstructural protein and capsid protein genes, complete cds	GAAGCCGCGGCCACGCCGAGTAGGATCGAGGGTACAGCTTC
MH933759.1 Human astrovirus isolate CMRHP43 nonstructural protein and capsid protein genes, complete cds	GAAGCCGCGGCCACGCCGAGTAGGATCGAGGGTACAGCTTC
MK005882.1 Porcine deltacoronavirus strain Swine/CHN/SC/2018/1, complete genome	TATGCCGAGGCCACGCGGAGTACGATCGAGGGTACAGCATA
MK026166.1 Canine astrovirus isolate DF-BC15-CAV-AUS-2017, complete genome	GTTCCCGAGGCCACGCCGAGTAGGATCGAGGGTACAGGTTC
MK032177.1 Infectious bronchitis virus strain gammaCoV/ck/China/I0724/17, complete genome	AGTGCCGGGGCCACGCGGAGTACGATCGAGGGTACAGCACT
MK032178.1 Infectious bronchitis virus strain gammaCoV/ck/China/I0718/17, complete genome	AGTGCCGGGGCCACGCGGAGTACGATCGAGGGTACAGCACT
MK032179.1 Infectious bronchitis virus strain gammaCoV/ck/China/I0722/17, complete genome	AGTGCCGGGGCCACGCGGAGTACGATCGAGGGTACAGCACT
MK032180.1 Infectious bronchitis virus strain gammaCoV/ck/China/I0737/17, complete genome	AGTGCCGGGGCCACGCGGAGTACGATCGAGGGTACAGCACT
MK032181.1 Infectious bronchitis virus strain gammaCoV/ck/China/I0347/11, complete genome	AGTGCCGGGGCCACGCGGAGTACGATCGAGGGTACAGCACT
MK059949.1 Human astrovirus 1 strain Oxford, complete genome	GAAGCCGCGGCCACGCCGAGTAGGAACGAGGGTACAGCTTC
MK059950.1 Human astrovirus 2 strain Oxford, complete genome	GAGACCGCGGCCACGCCGAGTAGGATCGAGGGTACAGTCTC
MK059951.1 Human astrovirus 3 strain Oxford, complete genome	GAGACCGCGGCCACGCCGAGTAGGATCGAGGGTACAGTCTC
MK059952.1 Human astrovirus 4 strain Oxford, complete genome	GGAGCCGCGGCCACGCCGAGTAGGATCGAGGGTACAGCTCC
MK059953.1 Human astrovirus 5 strain Oxford, complete genome	GAAGCCGCGGCCACGCCGAGTAGGATCGAGGGTACAGCTTC
MK059954.1 Human astrovirus 6 strain Oxford, complete genome	GAAGCCGCGGCCACGCCGAGTAGGATCGAGGGTACAGCTTC
MK059955.1 Human astrovirus 7 strain Oxford, complete genome	GAGACCGCGGCCACGCCGAGTAGGATCGAGGGTACAGTCTC
MK059956.1 Human astrovirus 8 strain Oxford, complete genome	GAAGCCGCGGCCACGCCGAGTAGGATCGAGGGTACAGCTTC
MK062179.1 SARS coronavirus Urbani isolate icSARS, complete genome	TTCATCGAGGCCACGCGGAGTACGATCGAGGGTACAGTGAA

MK062180.1 SARS coronavirus Urbani isolate icSARS-MA, complete genome	TTCATCGAGGCCACGCGGAGTACGATCGAGGGTACAGTGAA
MK062181.1 SARS coronavirus Urbani isolate icSARS-C3, complete genome	TTCATCGAGGCCACGCGGAGTACGATCGAGGGTACAGTGAA
MK062182.1 SARS coronavirus Urbani isolate icSARS-C3-MA, complete genome	TTCATCGAGGCCACGCGGAGTACGATCGAGGGTACAGTGAA
MK062183.1 SARS coronavirus Urbani isolate icSARS-C7, complete genome	TTCATCGAGGCCACGCGGAGTACGATCGAGGGTACAGTGAA
MK062184.1 SARS coronavirus Urbani isolate icSARS-C7-MA, complete genome	TTCATCGAGGCCACGCGGAGTACGATCGAGGGTACAGTGAA
MK067288.1 Norovirus GIV isolate dog/GIV.2/AN1610/USA/2017 nonstructural polyprotein (ORF1) gene, partial cds;	CTGACCGCGGCCACGCCGAGTAGGATCGAGGGTACAGTCAG
MK067289.1 Norovirus GIV isolate dog/GIV.2/AN843/USA/2011 nonstructural polyprotein (ORF1) gene, partial cds; a	CTGACCGCGGCCACGCCGAGTAGGATCGAGGGTACAGTCAG
MK067290.1 Norovirus GIV isolate dog/GIV.2/AN1638/USA/2017 nonstructural polyprotein (ORF1) gene, partial cds;	CTGACCGCGGCCACGCCGAGTAGGATCGAGGGTACAGTCAG
MK067291.1 Norovirus GIV isolate dog/GIV.2/AN1663/USA/2017 nonstructural polyprotein (ORF1) gene, partial cds;	CTGACCGCGGCCACGCCGAGTAGGATCGAGGGTACAGTCAG
MK067292.1 Norovirus GVI isolate dog/GVI.1/AN1632/USA/2017 nonstructural polyprotein (ORF1) gene, partial cds;	TTAGCCGCGGCCACGCCGAGTAGGACCGAGGGTACAGCTAA
MK067293.1 Norovirus GVI isolate dog/GVI.1/AN1633/USA/2017 nonstructural polyprotein (ORF1) gene, partial cds;	TTAGCCGCGGCCACGCCGAGTAGGACCGAGGGTACAGCTAA
MK067294.1 Norovirus GVI isolate dog/GVI.1/AN1634/USA/2017 nonstructural polyprotein (ORF1) gene, partial cds;	TTAGCCGCGGCCACGCCGAGTAGGACCGAGGGTACAGCTAA
MK067295.1 Norovirus GVI isolate dog/GVI.2/AN1640/USA/2017 nonstructural polyprotein (ORF1) gene, partial cds;	CTGACCGCGGCCACGCCGAGTAGGATCGAGGGTACAGTCGG
MK067296.1 Norovirus GVI isolate dog/GVI.2/AN1656/USA/2017 VP1 (ORF2) gene, partial cds; and VP2 (ORF3) gene,	CTGACCGCGGCCACGCCGAGTAGGATCGAGGGTACAGTCGG
MK067297.1 Norovirus GVI isolate dog/GVI.2/AN1655/USA/2017 VP1 (ORF2) gene, partial cds; and VP2 (ORF3) gene,	CTGACCGCGGCCACGCCGAGTAGGATCGAGGGTACAGTCGG
MK071267.1 Avian coronavirus AvCoV/Gallus gallus//H120 complete genome	AGTGCCGGGGCCACGCGGAGTACGATCGAGGGTACAGCACT
MK125058.1 Goose astrovirus isolate JSHA, complete genome	GCAGCCGCGGCCACGCCGAGTAGGATCGAGGGTACAGCTGC
MK142676.1 Infectious bronchitis virus isolate ahysx-1, complete genome	AGTGCCGGGGCCACGCGGAGTACGATCGAGGGTACAGCACT
MK189093.1 UNVERIFIED_ORG: Ruddy turnstone astrovirus ORF1a, ORF1b, and ORF2 genes, complete cds	AGAATCGAGGCCACGGCGAGTAGCATCGAGGGTACAATTCT
MK204393.1 Avian coronavirus non-structural polyprotein (ORF1ab) and spike protein (S) genes, complete cds	AGTGCCGGGGCCACGCGGAGTACGATCGAGGGTACAGCACT
MK204411.1 Avian coronavirus non-structural polyprotein (ORF1ab), spike protein (S), 3a (3a), 3b (3b), envelope prot	AGTGCCGGGGCCACGCGGAGTACGATCGAGGGTACAGCACT
MK211169.1 Porcine deltacoronavirus strain CHN/Sichuan/2017, complete genome	TATGCCGAGGCCACGCGGAGTACGATCGAGGGTACAGCATA
MK211323.1 Mamastrovirus 13 strain MOxAstV-CH18, complete genome	AGTCCCGAGGCCACGCCGAGTAGGATCGAGGGTACAGGATT
MK211374.1 Coronavirus BtRl-BetaCoV/SC2018, complete genome	TTCACCGAGGCCACGCGGAGTACGATCGAGGGTACAGTGAA
MK211375.1 Coronavirus BtRs-BetaCoV/YN2018A, complete genome	TTCACCGAGGCCACGCGGAGTACGATCGAGGGTACAGTGAA
MK211376.1 Coronavirus BtRs-BetaCoV/YN2018B, complete genome	TTCACCGAGGCCACGCGGAGTACGATCGAGGGTACAGTGAA
MK211377.1 Coronavirus BtRs-BetaCoV/YN2018C, complete genome	TTCACCGAGGCCACGCGGAGTACGATCGAGGGTACAGTGAA
MK211378.1 Coronavirus BtRs-BetaCoV/YN2018D, complete genome	TTCACCGAGGCCACGCGGAGTACGATCGAGGGTACAGTGAA
MK217372.1 Infectious bronchitis virus strain I0221/17, complete genome	AGTGCCGGGGCCACGCGGAGTACGATCGAGGGTACAGCACT
MK217373.1 Infectious bronchitis virus strain I0725/17, complete genome	AGTGCCGGTGCCACGCGGAGTACGATCGAGGGTACAGCACT
MK217374.1 Infectious bronchitis virus strain I0916/16, complete genome	AGTGCCGGGGCCACGCGGAGTACGATCGAGGGTACAGCACT
MK217375.1 Infectious bronchitis virus strain I1209/16, complete genome	AGTGCCGGGGCCACGCGGAGTACGATCGAGGGTACAGCACT
MK286562.1 Mamastrovirus 13 strain OvAstV-CH17, partial genome	AATCCCGAGGCCACGCCGAGTAGGATCGAGGGTACAGGATT

MK296753.1 Human astrovirus 3 isolate 17W1028 ORF1a, ORF1b, and ORF2 genes, complete cds	GAGACCGCGGCCACGCCGAGTAGGATCGAGGGTACAGTCTC
MK309398.1 Infectious bronchitis virus isolate CK/CH/GD/HY16, complete genome	AGTGCCGGGGCCACGCGGAGTACGATCGAGGGTACAGCACT
MK329221.1 Infectious bronchitis virus isolate CK-CH-GX-YL17, complete genome	AGTGCCGGGGCCACGCGGAGTACGATCGAGGGTACAGCACT
MK330604.1 Porcine deltacoronavirus strain CHN/Sichuan/2017, complete genome	TATGCCGAGGCCACGCGGAGTACGATCGAGGGTACAGCATA
MK330605.1 Porcine deltacoronavirus strain CHN/Sichuan/2018, complete genome	TATGCCGAGGCCACGCGGAGTACGATCGAGGGTACAGCATA
MK355396.1 Porcine deltacoronavirus strain CHN-SC2015, complete genome	TATGCCGAGGCCACGCGGAGTACGATCGAGGGTACAGCATA
MK359255.1 Canada goose coronavirus strain Cambridge_Bay_2017, complete genome	TGTGCCGGGGCCACGCGGAGTACGATCGAGGGTACAGCACA
MK395165.1 Murine astrovirus isolate SJ001, partial genome	GAAGCCGCGGCCACGCCGAGTAGGATCGAGGGTACAGCTTT
MK395166.1 Murine astrovirus isolate SJ002, complete genome	AAAGCCGCGGCCACGCCGAGTAGGATCGAGGGTACAGCTTT
MK423876.1 Pheasant coronavirus strain gammaCoV/ph/China/I0710/17, complete genome	AGTGCCGAGGCCACGCGGCGTACGATCGAGGGTACAGCACT
MK423877.1 Pheasant coronavirus strain gammaCoV/ph/China/I0623/17, complete genome	AGTGCCGAGGCCACGCGGCGTACGATCGAGGGTACAGCACT
MK521912.1 Tasmanian devil-associated astrovirus 1 isolate Stony Head/Tasmania/Sarcophilus_harrisii/2016/DN108[illegible]	TAACCCGAGGCCACGCGGAGTACGATCGAGGGTACAGGTTA
MK572803.1 Porcine deltacoronavirus strain SCNC201705, complete genome	TATGCCGAGGCCACGCGGAGTACGATCGAGGGTACAGCATA
MK574042.1 Infectious bronchitis virus strain ck/CH/LHB/110615, complete genome	AGTGCCGGGGCCACGCGGAGTACGATCGAGGGTACAGCACT
MK574043.1 Infectious bronchitis virus strain ck/CH/LHB/110617, complete genome	AGTGCCGGGGCCACGCGGAGTACGATCGAGGGTACAGCACT
MK581201.1 Infectious bronchitis virus strain gammaCoV/Ck/Poland/79/1989, complete genome	AGTGCCGGGGCCACGCGGAGTACGATCGAGGGTACAGCACT
MK581202.1 Infectious bronchitis virus strain gammaCoV/Ck/Poland/80/1989, complete genome	AGTGCCGGGGCCACGCGGAGTACGATCGAGGGTACAGCACT
MK581203.1 Infectious bronchitis virus strain gammaCoV/Ck/Poland/162/1997, complete genome	AGTGCCGAGGCCACGCGGAGTACGATCGAGGGTACAGCACT
MK581204.1 Infectious bronchitis virus strain gammaCoV/Ck/Poland/255/1997, complete genome	AGTGCCGGGGCCACGCGGAGTACGATCGAGGGTACAGCACT
MK581205.1 Infectious bronchitis virus strain gammaCoV/Ck/Poland/548/2004, complete genome	AGTGCCGAGGCCACGCGGAGTACGATCGAGGGTACAGCACT
MK581207.1 Infectious bronchitis virus strain gammaCoV/Ck/Poland/G103/2016, complete genome	AGTGCCGGGGCCACGCGGAGTACGATCGAGGGTACAGCACT
MK581208.1 Infectious bronchitis virus strain gammaCoV/Ck/Poland/G225/2017, complete genome	AGTGCCGAGGCCACGCGGAGTACGATCGAGGGTACAGCACT
MK618759.1 Infectious bronchitis virus isolate K047-12, partial genome	AGTGCCGGGGCCACGCGGAGTACGATCGAGGGTACAGCACT
MK644086.1 Infectious bronchitis virus strain E160_YN, complete genome	AGTGCCGGGGCCACGCGGAGTACGATCGAGGGTACAGCACT
MK671306.1 Mamastrovirus 2 isolate MAV2/17CC0308, complete genome	AGAGCCGAGGCCACGCCGAGTAGGATCGAGGGTACAGCTCT
MK671307.1 Mamastrovirus 2 isolate MAV2/17HRB0511, complete genome	AGAGCCGAGGCCACGCCGAGTAGGATCGAGGGTACAGCTCT
MK671308.1 Mamastrovirus 2 isolate MAV2/17JL0318, complete genome	AGAGCCGAGGCCACGCCGAGTAGGATCGAGGGTACAGCTCT
MK671309.1 Mamastrovirus 2 isolate MAV2/17SP0801, complete genome	AGAGCCGAGGCCACGCCGAGTAGGATCGAGGGTACAGCTCT
MK671310.1 Mamastrovirus 2 isolate MAV2/18CC0502, complete genome	AGAGCCGAGGCCACGCCGAGTAGGATCGAGGGTACAGCTCT
MK671311.1 Mamastrovirus 2 isolate MAV2/18JL0310, complete genome	AGAGCCGAGGCCACGCCGAGTAGGATCGAGGGTACAGCTCT
MK671312.1 Mamastrovirus 2 isolate MAV2/18JL0705, complete genome	AGAGCCGAGGCCACGCCGAGTAGGATCGAGGGTACAGCTCT
MK671313.1 Mamastrovirus 2 isolate MAV2/18SY0803, complete genome	AGAGCCGAGGCCACGCCGAGTAGGATCGAGGGTACAGCTCT
MK728875.1 Infectious bronchitis virus strain M41-CK, complete genome	AGTGCCGGGGCCACGCGGAGTACGACCGAGGGTACAGCACT

MK878536.1 Infectious bronchitis virus isolate GA9977/2019, complete genome	AGTGCCGGGGCCACGCGGAGTACGATCGAGGGTACAGCACT
MK937828.1 Infectious bronchitis virus strain I1124/16, complete genome	AGTGCCGAGGCCACGCGGAGTACGATCGAGGGTACAGCACT
MK937829.1 Infectious bronchitis virus strain I0306/17, complete genome	AGTGCCGGGGCCACGCGGAGTACGATCGAGGGTACAGCACT
MK937830.1 Infectious bronchitis virus strain M41, complete genome	AGTGCCGGGGCCACGCGGAGTACGACCGAGGGTACAGCACT
MK937831.1 Infectious bronchitis virus strain H120, complete genome	AGTGCCGGGGCCACGCGGAGTACGATCGAGGGTACAGCACT
MK937832.1 Infectious bronchitis virus strain ck/CH/LJL/130906, complete genome	AGTGCCGAGGCCACGCGGAGTACGATCGAGGGTACAGCACT
MK937833.1 Infectious bronchitis virus strain ck/CH/LJL/140820, complete genome	AGTGCCGGGGCCACGCGGAGTACGATCGAGGGTACAGCACT
MK962341.1 Mamastrovirus 3 strain AstV3/Pig-wt/ESP/B333/2017, complete genome	GCCCCCGAGGCCACGCCGAGTAGGAACGAGGGTACAGGGGC
MK962342.1 Mamastrovirus 3 strain AstV3/Pig-wt/ESP/B377/2017, complete genome	GCCCCCGAGGCCACGCCGAGTAGGAACGAGGGTACAGGGGC
MK962352.1 San Miguel sea lion virus 12 isolate 2615T, complete genome	GTTGCCGCGGCCACGCCGAGTAGGATCGAGGGTACAGCAAC
MK987100.1 Bovine astrovirus isolate BoAstV-VC34/346 nonstructural protein 1ab (ORF1ab) and nonstructrial protei	AGTCCCGAGGCCACGCCGAGTAGGATCGAGGGTACAGGATT
MK987103.1 Bovine astrovirus isolate BoAstV-VC65/698 nonstructural protein 1ab (ORF1ab) and nonstructrial protei	AATCCCGAGGCCACGCCGAGTAGGATCGAGGGTACAGGATT
MK993519.1 Porcine deltacoronavirus isolate CHN/Sichuan/2019, complete genome	TATGCCGAGGCCACGCGGAGTACGATCGAGGGTACAGCATA
MN025260.1 Porcine deltacoronavirus, complete genome	TATGCCGAGGCCACGCGGAGTACGATCGAGGGTACAGCATA
MN068023.1 Goose astrovirus isolate GTF-04, complete genome	GCAGCCGCGGCCACGCCGAGTAGGATCGAGGGTACAGCTGC
MN068024.1 Goose astrovirus isolate GTF-07, complete genome	GCAGCCGCGGCCACGCCGAGTAGGATCGAGGGTACAGCTGC
MN096598.1 Infectious bronchitis virus strain ck/CH/YNSL/160501, complete genome	AGTGCCGGGGCCACGCGGAGTACGATCGAGGGTACAGCACT
MN128086.1 Infectious bronchitis virus strain TW2296/95vac, complete genome	AGTGCCGGGGCCACGCGGAGTACGATCGAGGGTACAGCACT
MN128087.1 Infectious bronchitis virus strain TW2575/98vac, complete genome	AGTGCCGGGGCCACGCGGAGTACGATCGAGGGTACAGCACT
MN128088.1 Infectious bronchitis virus strain TW2296/95w, complete genome	AGTGCCGGGGCCACGCGGAGTACGATCGAGGGTACAGCACT
MN148428.1 Tiger astrovirus CS-2018, complete genome	AGAGCCGAGGCCACGCCGAGTAGGATCGAGGGTACAGCTCT
MN149392.1 Duck astrovirus 1 isolate D17, complete genome	GCAGCCGCGGCCACGCCGAGTAGGATCGAGGGTACAGCTGC
MN175321.1 Goose astrovirus strain AstV/Goose/2018/HLJ01, complete genome	GCAGCCGCGGCCACGCCGAGTAGGATCGAGGGTACAGCTGC
MN249445.1 Porcine deltacoronavirus isolate CHN-JS-2017, complete genome	TATGCCGAGGCCACGCGGAGTACGATCGAGGGTACAGCATA
MN262644.1 Avian coronavirus strain CV10, complete genome	AGTGCCGGGGCCACGCGGAGTACGATCGAGGGTACAGCACT
MN307884.1 Infectious bronchitis virus isolate ck/CH/LJX/2017/07, complete genome	AGTGCCGGGGCCACGCGGAGTACGATCGAGGGTACAGCACT
MN337323.1 Goose astrovirus isolate XX, complete genome	GCAGCCGCGGCCACGCCGAGTAGGATCGAGGGTACAGCTGC
MN399857.1 Goose astrovirus isolate SDXT, complete genome	GCAGCCGCGGCCACGCCGAGTAGGATCGAGGGTACAGCTGC
MN428641.1 Goose astrovirus isolate AHAU1, complete genome	GCAGCCGCGGCCACGCCGAGTAGGATCGAGGGTACAGCTGC
MN428642.1 Goose astrovirus isolate GD AHAU2, complete genome	GCAGCCGCGGCCACGCCGAGTAGGATCGAGGGTACAGCTGC
MN428643.1 Goose astrovirus isolate AHAU3, complete genome	GCAGCCGCGGCCACGCCGAGTAGGATCGAGGGTACAGCTGC
MN428644.1 Goose astrovirus isolate AHAU4, complete genome	GCAGCCGCGGCCACGCCGAGTAGGATCGAGGGTACAGCTGC
MN428645.1 Goose astrovirus isolate AHAU5, complete genome	GCAGCCGCGGCCACGCCGAGTAGGATCGAGGGTACAGCTGC

MN444721.1 Human astrovirus 3 strain Hu/US/2017/CA-RGDS-1074, complete genome	GAGACCGCGGCCACGCCGAGTAGGATCGAGGGTACAGTCTC
MN464146.1 Bovine astrovirus non-structural polyprotein 1ab (ORF1ab) and non-structural polyprotein 1A (ORF1A) g	AATCCCGAGGCCACGCCGAGTAGGATCGAGGGTACAGGATT
MN509587.1 Infectious bronchitis virus strain cK/CH/LDL/091021, complete genome	AGTGCCGGGGCCACGCGGAGTACGATCGAGGGTACAGCACT
MN509588.1 Infectious bronchitis virus strain I0730/17, complete genome	AGTGCCGGGGCCACGCGGAGTACGATCGAGGGTACAGCACT
MN509589.1 Infectious bronchitis virus strain cK/CH/LHLJ/110664, complete genome	AGTGCCGGGGCCACGCGGAGTACGATCGAGGGTACAGCACT
MN510439.1 UNVERIFIED: Human astrovirus 1 isolate G19051 genomic sequence	GAAGCCGCGGCCACGCCGAGTAGGAACGAGGGTACAGCTTC
MN510440.1 UNVERIFIED: Human astrovirus 2 isolate G19052 genomic sequence	GAGACCGCGGCCACGCCGAGTAGGAACGAGGGTACAGTCTC
MN512434.1 Infectious bronchitis virus isolate IBV/Ck/Can/17-035614, complete genome	AGTGCCGGGGCCACGCGGAGTACGATCGAGGGTACAGCACT
MN512435.1 Infectious bronchitis virus isolate IBV/Ck/Can/17-036989, complete genome	AGTGCCGGGGCCACGCGGAGTACGATCGAGGGTACAGCACT
MN512436.1 Infectious bronchitis virus isolate IBV/Ck/Can/18-048192T, complete genome	AGTGCCGGGGCCACGCGGAGTACGATCGAGGGTACAGCACT
MN512437.1 Infectious bronchitis virus isolate IBV/Ck/Can/18-048430, complete genome	AGTGCCGGGGCCACGCGGAGTACGATCGAGGGTACAGCACT
MN512438.1 Infectious bronchitis virus isolate IBV/Ck/Can/18-049707, complete genome	AGTGCCGGGGCCACGCGGAGTACGATCGAGGGTACAGCACT
MN517816.1 Infectious bronchitis virus strain cK/CH/LJL/090608, complete genome	AGTGCCGGGGCCACGCGGAGTACGATCGAGGGTACAGCACT
MN517817.1 Infectious bronchitis virus strain cK/CH/LSD/110723, complete genome	AGTGCCGGGGCCACGCGGAGTACGATCGAGGGTACAGCACT
MN531554.1 Infectious bronchitis virus strain cK/CH/LLN/120611, complete genome	AGTGCCGGGGCCACGCGGAGTACGATCGAGGGTACAGCACT
MN531555.1 Infectious bronchitis virus strain cK/CH/LSD/1112145, complete genome	AGTGCCGGGGCCACGCGGAGTACGATCGAGGGTACAGCACT
MN531556.1 Infectious bronchitis virus strain cK/CH/LSD/110856, complete genome	AGTGCCGGGGCCACGCGGAGTACGATCGAGGGTACAGCACT
MN566147.1 Infectious bronchitis virus isolate GA/1359/1994, complete genome	AGTGCCGGGGCCACGCGGAGTACGATCGAGGGTACAGCACT
MN599049.1 Infectious bronchitis virus isolate GA/1476/2015, complete genome	AGTGCCGGGGCCACGCGGAGTACGATCGAGGGTACAGCACT
MN711790.1 Infectious bronchitis virus isolate GA/1472/2004, complete genome	AGTGCCGGGGCCACGCGGAGTACGATCGAGGGTACAGCACT
MN732558.1 Avian nephritis virus strain ANV/CHN/BJCP510-2/2018, complete genome	TTTCCCGAGGCCACGGCGAGTAGCATCGAGGGTACAGGAAA
MN732559.1 Avian nephritis virus strain ANV/CHN/GXJL815/2017, complete genome	TTTCCCGAGGCCACGGCGAGTAGCATCGAGGGTACAGGAAA
MN780842.1 Murine astrovirus isolate JS1, complete genome	AAAGCCGCGGCCACGCCGAGTAGGATCGAGGGTACAGCTTT
MN794188.1 Infectious bronchitis virus strain I0305/19, complete genome	AGTGCCGGGGCCACGCGGAGTACGATCGAGGGTACAGCACT
MN996532.1 Bat coronavirus RaTG13, complete genome	TTCACCGAGGCCACGCGGAGTACGATCGAGGGTACAGTGAA
MT040333.1 Pangolin coronavirus isolate PCoV_GX-P4L, complete genome	TTCACCGAGGCCACGCGGAGTACGATCGAGGGTACAGTGAA
MT040335.1 Pangolin coronavirus isolate PCoV_GX-P5L, complete genome	TTCACCGAGGCCACGCGGAGTACGATCGAGGGTACAGTGAA
MT040336.1 Pangolin coronavirus isolate PCoV_GX-P5E, complete genome	TTCACCGAGGCCACGCGGAGTACGATCGAGGGTACAGTGAA
MT072864.1 Pangolin coronavirus isolate PCoV_GX-P2V, complete genome	TTCACCGAGGCCACGCGGAGTACGATCGAGGGTACAGTGAA
MT072865.1 Pangolin coronavirus isolate PCoV_GX-P3B genomic sequence	TTCACCGAGGCCACGCGGAGTACGATCGAGGGTACAGTGAA
MT138366.1 Picornaviridae sp. isolate thr106pic1 genomic sequence	GCTCCCGCGGCCACGCCGAGTAGGATCGAGGGTACAGGAGC
MT308984.1 Mutant SARS coronavirus Urbani clone SARS-Urbani-MA_SHC014-spike, complete genome	TTCATCGAGGCCACGCGGAGTACGATCGAGGGTACAGTGAA
NC_001451.1 Avian infectious bronchitis virus, complete genome	AGTGCCGGGGCCACGCGGAGTACGATCGAGGGTACAGCACT

NC_001943.1 Human astrovirus, complete genome	GAAGCCGCGGCCACGCCGAGTAGGAACGAGGGTACAGCTTC
NC_002469.1 Ovine astrovirus, complete genome	AATCCCGAGGCCACGCCGAGTAGGATCGAGGGTACAGGATT
NC_003790.1 Chicken astrovirus, complete genome	TTTCCCGAGGCCACGGCGAGTAGCATCGAGGGTACAGGAAA
NC_003983.1 Equine rhinitis B virus 1, complete genome	CTCGCCGAGGCCACGCCGAGTAGGACCGAGGGTACAGCGAG
NC_004579.1 Mink astrovirus, complete genome	TACCCGAGGCCACGCCGAGTTAGGATCGAGGGTACAGGTAG
NC_004718.3 SARS coronavirus, complete genome	TTCATCGAGGCCACGCGGAGTACGATCGAGGGTACAGTGAA
NC_010800.1 Turkey coronavirus, complete genome	AGTGCCGGGGCCACGCGGAGTACGATCGAGGGTACAGCACA
NC_011547.1 Bulbul coronavirus HKU11-934, complete genome	TGTGCCGAGGCCACGCGGAGTACGATCGAGGGTACAGCACA
NC_011549.1 Thrush coronavirus HKU12-600, complete genome	TATGCCGAGGCCACGCGGAGTACGATCGAGGGTACAGCATA
NC_011550.1 Munia coronavirus HKU13-3514, complete genome	TGTGTCGAGGCCACGCGGAGTACGATCGAGGGTACAGCACA
NC_012437.1 Duck astrovirus C-NGB, complete genome	GCAGCCGCGGCCACGCCGAGTAGGATCGAGGGTACAGCTGC
NC_013060.1 Astrovirus VA1, complete genome	TGCGCCGAGGCCACGCCGAGTAGGATCGAGGGTACAGCGCT
NC_013443.1 HMO Astrovirus A, complete genome	TCCGCCGAGGCCACGCCGAGTAGGATCGAGGGTACAGCGGA
NC_014412.1 Turdivirus 2, complete genome	AGACCCGAGGCCACGCCGAGTAGGATCGAGGGTACAGGTCT
NC_014413.1 Turdivirus 3, complete genome	GCTCCCGCGGCCACGCCGAGTAGGATCGAGGGTACAGGAGC
NC_014470.1 Bat coronavirus BM48-31/BGR/2008, complete genome	GTCACCGAGGCCACGCGGAGTACGATCGAGGGTACAGTGAC
NC_015934.1 Bat picornavirus 3, complete genome	AAGACCGAGGCCACGCGGAGTACGAACGAGGGTACAGTCTT
NC_015935.1 Mouse astrovirus M-52/USA/2008, complete genome	CCTGCCGCGGCCACGCCTAGTCGGAACGAGGGTACAGCAGG
NC_016964.1 Canine picornavirus, complete genome	TTGTCCGAGGCCACGCCGAGTAGGATCGAGGGTACAGACTT
NC_016991.1 White-eye coronavirus HKU16, complete genome	TGCACCGAGGCCACGCGGAGTACGATCGAGGGTACAGTGCA
NC_016992.1 Sparrow coronavirus HKU17, complete genome	TATGCCGAGGCCACGCGGAGTACGATCGAGGGTACAGCATA
NC_016993.1 Magpie-robin coronavirus HKU18, complete genome	TGTGCCGAGGCCACGCGGAGTACGATCGAGGGTACAGCACA
NC_016996.1 Common-moorhen coronavirus HKU21, complete genome	TGAACCGAGGCCACGCGGAGTACGATCGAGGGTACAGTTCA
NC_018702.1 Murine astrovirus, complete genome	AAAGCCGCGGCCACGCCGAGTAGGATCGAGGGTACAGCTTT
NC_019026.1 Astrovirus VA3 isolate VA3/human/Vellore/28054/2005, complete genome	TGCGCCGAGGCCACGCCGAGTAGGATCGAGGGTACAGCGCT
NC_019027.1 Astrovirus VA4 isolate VA4/human/Nepal/s5363, complete genome	ATCCCCGAGGCCACGCCGAGTAGGATCGAGGGTACAGGGAT
NC_019494.1 Porcine astrovirus 3 isolate US-MO123, complete genome	GCCCCCGAGGCCACGCCGAGTAGGAACGAGGGTACAGGGGC
NC_022249.1 Feline astrovirus 2 strain 1637F, complete genome	AGAGCCGAGGCCACGCCGAGTAGGATCGAGGGTACAGCTCT
NC_023636.1 Porcine astrovirus 5 isolate AstV5-US-IA122, complete genome	TGAGCCGAGGCCACGCCGAGTAGGATCGAGGGTACAGCTCA
NC_024472.1 Human astrovirus BF34, complete genome	GTCGCCGAGGCCACGCCGAGTAGGATCGAGGGTACAGCGAT
NC_024498.1 Bovine astrovirus CH13, complete genome	AATCCCGAGGCCACGCCGAGTAGGATCGAGGGTACAGGATT
NC_024701.1 Feline astrovirus D1 isolate FAstV-D1, complete genome	CCACCCGAGGCCACGCCGAGTAGGATCGAGGGTACAGGTGG
NC_025217.1 Bat Hp-betacoronavirus/Zhejiang2013, complete genome	CACACCGAGGCCACGCCGAGTAGGAACGAGGGTACAGTGTG

NC_025379.1 Mamastrovirus 3 isolate PAstV-GX1, complete genome	AAAGCCGAGGCCACGCCGAGTAGGATCGAGGGTACAGCTTT
NC_026814.1 Canine astrovirus strain Gillingham/2012/UK, complete genome	GTTCCCGAGGCCACGCCGAGTAGGATCGAGGGTACAGGTTC
NC_033792.1 Qinghai Himalayan marmot astrovirus 1 isolate HHMAstV1, complete genome	TGAGCCGCGGCCACGCCGAGTAGGATCGAGGGTACAGCTCA
NC_033821.1 Qinghai Himalayan marmot astrovirus 2 isolate HHMAstV2, complete genome	CTAGCCGAGGCCACGCCGAGTAGGATCGAGGGTACAGCTAG
NC_034975.1 California sea lion astrovirus 2 RNA-dependent RNA polymerase gene, partial cds; and capsid protein ge	CAAGCCGAGGCCACGCCGAGTAGGATCGAGGGTACAGCTTG
NC_036583.1 Rodent astrovirus isolate GX-006, complete genome	GAAGCCGAGGCCACGCCGAGTAGGATCGAGGGTACAGCTCC
NC_039208.1 Porcine coronavirus HKU15 strain HKU15-155, complete genome	TATGCCGAGGCCACGCGGAGTACGATCGAGGGTACAGCATA
NC_043097.1 Mamastrovirus 11 RNA-dependent RNA polymerase gene, partial cds; and capsid protein gene, comple	CTTCCCGAGGCCACGCCGAGTAGGACCGAGGGTACAGGGAG
NC_043099.1 Mamastrovirus 14 isolate AFCD57 polyprotein 1AB gene, partial cds; and capsid protein precursor, gene	AGAGCCGAGGCCACGCCAAGTCGGATCGAGGGTACAGCTCA
NC_043101.1 Mamastrovirus 16 isolate AFCD11 polyprotein 1AB gene, partial cds; and capsid protein precursor, gene	TGAGCCGAGGCCACGCCGAGTAGGATCGAGGGTACAGCTCC
NC_043416.1 Astrovirus dogfaeces/Italy/2005 partial RNA-dependant RNA polymerase, genomic RNA	GTTCCCGAGGCCACGCCGAGTAGGATCGAGGGTACAGGTTC
NC_046965.1 Canada goose coronavirus strain Cambridge_Bay_2017, complete genome	TGTGCCGGGGCCACGCGGAGTACGATCGAGGGTACAGCACA
NC_048213.1 Infectious bronchitis virus isolate Ind-TN92-03, complete genome	AGTGCCGGGGCCACGCGGAGTACGACCGAGGGTACAGCACT
NC_048214.1 Duck coronavirus isolate DK/GD/27/2014, complete genome	AGTGCCGGGGCCACGCGGAGTACGATCGAGGGTACAGCACT
S47249.1 {astrovirus genome 3' end} [human astrovirus type 1, Genomic RNA, 118 nt]	GAAGCCGCGGCCACGCCGAGTAGGAACGAGGGTACAGCTTC
S68561.1 non-structural protein, capsid protein [human astrovirus serotype 1, isolate A88/2, Newcastle, Genomic RN	GAAGCCGCGGCCACGCCGAGTAGGAACGAGGGTACAGCTTC
U04804.1 Avian infectious bronchitis virus 3' noncoding region	AGTGCCGGGGCCACGCGGAGTACGATCGAGGGTACAGCACT
U49858.1 Avian infectious bronchitis virus strain CU-T2 spike protein (gene 2), gene 3, membrane protein (gene 4), ge	AGTGCCGGGGCCACGCGGAGTACGATCGAGGGTACAGCACT
U52594.1 Avian infectious bronchitis virus Vic S nucleocapsid protein mRNA, complete cds	AGTGCCGGGGCCACGCGGAGTACGATCGAGGGTACAGCACT
U52595.1 Avian infectious bronchitis virus V5/90 nucleocapsid protein mRNA, partial cds	AGTGCCGGGGCCACGCGGAGTACGATCGAGGGTACAGCACT
U52596.1 Avian infectious bronchitis virus N1/62 nucleocapsid protein mRNA, partial cds	AGTGCCGGGGCCACGCGGAGTACGATCGAGGGTACAGCACT
U52597.1 Avian infectious bronchitis virus N9/74 nucleocapsid protein mRNA, partial cds	AGTGCCGGGGCCACGCGGAGTACGATCGAGGGTACAGCACT
U52598.1 Avian infectious bronchitis virus N2/75 nucleocapsid protein mRNA, partial cds	AGTGCCGAGGCCACGCGGAGTACGATCGAGGGTACAGCACT
U52599.1 Avian infectious bronchitis virus N1/88 nucleocapsid protein mRNA, complete cds	AGTGCCGGGGCCACGCGGAGTACGATCGAGGGTACAGCACT
U52600.1 Avian infectious bronchitis virus Q3/88 nucleocapsid protein mRNA, complete cds	AGTGCCGAGGCCACTCGGAGTACGATCGAGGGTACAGCACT
U52601.1 Avian infectious bronchitis virus V18/91 nucleocapsid protein mRNA, complete cds	AGTGCCGGGGCCACGCGGAGTACGATCGAGGGTACAGCACT
X96871.1 Equine Rhinovirus type 2 genomic sequence	CTCGCCGAGGCCACGCCGAGTAGGACCGAGGGTACAGCGAG
Y08627.1 Human astrovirus type 1 gene encoding capsid protein precursor (partial)	GAAGCCGCGGCCACGCCGAGTAGGAACGAGGGTACAGCTTC
Y08628.1 Human astrovirus type 2 gene encoding capsid protein precursor (partial)	GAGACCGCGGCCACGCCGAGTAGGATCGAGGGTACAGTCTC
Y08629.1 Human astrovirus type 3 gene encoding capsid protein precursor (partial)	GAGACCGCGGCCACGCCGAGTAGGATCGAGGGTACAGTCTC
Y08630.1 Human astrovirus type 4 gene encoding capsid protein precursor (partial)	GGAGCCGCGGCCACGCCGAGTAGGATCGAGGGTACAGCTCC
Y08631.1 Human astrovirus type 5 gene encoding capsid protein precursor (partial)	GAAGCCGCGGCCACGCCGAGTAGGATCGAGGGTACAGCTTC
Y08632.2 Human astrovirus type 7 gene for capsid protein precursor	GAGACCGCGGCCACGCCGAGTAGGATCGAGGGTACAGTCTC

Y08633.1 Human astrovirus gene encoding capsid protein precursor (partial), isolate GII33	GAAGCCGCGGCCACGCCGAGTAGGATCGAGGGTACAGCTTC
Y08634.1 Human astrovirus gene encoding capsid protein precursor (partial), isolate B55	GAAGCCGCGGCCACGCCGAGTAGGAACGAGGGTACAGCTTC
Y15937.2 Sheep astrovirus, complete genome, genomic RNA	AATCCCGAGGCCACGCCGAGTAGGATCGAGGGTACAGGATT
Y15938.2 Porcine astrovirus gene for capsid protein precursor, genomic RNA	AAAGCCGAGGCCACGCCGAGTAGGAACGAGGGTACAGCTTT
Z11682.1 Human astrovirus type 1, 3' terminal sequence RNA	GAAGCCGCGGCCACGCCGAGTAGGAACGAGGGTACAGCTTC
Z25771.1 Human astrovirus type 1 genes for capsid protein and nonstructural protein	GAAGCCGCGGCCACGCCGAGTAGGAACGAGGGTACAGCTTC
Z30541.1 Avian infectious bronchitis virus mRNA for chimeric gene	AGTGCCGGGGCCACGCGGAGTACGATCGAGGGTACAGCACT
Z33883.1 Human astrovirus type 4 gene encoding structural polyprotein vp1-3	GGAGCCGCGGCCACGCCGAGTAGGATCGAGGGTACAGCTCC
Z46658.1 Human astrovirus type 6 gene for capsid polyprotein precursor	GAAGCCGCGGCCACGCCGAGTAGGATCGAGGGTACAGCTTC
Z66541.1 Human astrovirus type 8 orf2 gene for capsid protein	GAAGCCGCGACCACGCCGAGTAGGATCGAGGGTACAGCTTC
Z69629.1 Infectious bronchitis virus RNA (defective RNA CD-61)	AGTGCCGGGGCCACGCGGAGTACGATCGAGGGTACAGCACT
GQ415661.1 HMO Astrovirus B isolate NI-196 non-structural protein gene	TGCGCCGAGGCCACGCCGAGTAGGATCGAGGGTACAGGTAG
MG846421.1 Chicken astrovirus strain RS/BR/15/6R-1 RNA-dependent RNA polymerase protein gene	GATGCCGAGGCCACGCCGAGTAGGATCGAGGGTACAGCATC
FJ692500.1 Norovirus dog/GVI.1/HKU_Ca026F/2007/HKG ORF1 polyprotein, copy2	GTTGTCGAGGCCACGCCGAGTAGGATCGAGGGTACAACAAC
FJ692501.1 Norovirus dog/GVI.1/HKU_Ca035F/2007/HKG ORF1 polyprotein, copy2	GTTGTCGAGGCCACGCCGAGTAGGATCGAGGGTACAACAAC
NC_044047.1 Norovirus dog/GVI.1/HKU_Ca026F/2007/HKG ORF1 polyprotein, copy2	GTTGTCGAGGCCACGCCGAGTAGGATCGAGGGTACAACAAC
FJ571066.1 Bat astrovirus Tm/Guangxi/LD77/2007 non-structural polyprotein 1AB (pol) gene, copy2	GCGACCGAGGCCACGCCGAGTAGGATCGAGGGTACAGTCAC
KJ790198.1 Bat norovirus polyprotein gene, copy2	GCAGCCGCGGCCACGCCGAGTAGGACCGAGGGTACAGCTGC
MN725025.1 Chicken astrovirus strain CAstV/CHN/HBLP717-1/2018, copy3	CACACCGGGGCCTCGCCGAGTAGGAACGAGGGTACAGTGCC
MN725026.1 Chicken astrovirus strain CAstV/CHN/GDYHTJ718-6/2018, copy3	CACACCGGGGCCTCGCCGAGTAGGAACGAGGGTACAGTGCC
NC_043100.1 Bat astrovirus Tm/Guangxi/LD77/2007 non-structural polyprotein 1AB (pol) gene, copy2	GCGACCGAGGCCACGCCGAGTAGGATCGAGGGTACAGTCAC
JF414802.1 Chicken astrovirus isolate GA2011, copy2	GGCACCGGGGCCACGCCGAGTAGGATCGAGGGTACAGTGCC
JN582327.1 Chicken astrovirus isolate 11672 polymerase gene, copy2	GGCACCGGGGCCACGCCGAGCAGGAACGAGGGTACAGTGCC
JN582328.1 Chicken astrovirus isolate FP3 polymerase gene, copy2	GGCACCGGGGCCACGCCGAGTAGGAACGAGGGTACAGTGCC
KJ621028.1 Chicken astrovirus isolate VRDC/CAstV/SZ/VHINP-2 capsid protein (ORF2) gene, copy2	GGCACCGGGGCCACGCCGAGTAGGAACGAGGGTACAGTGCC
KJ621029.1 Chicken astrovirus isolate VRDC/CAstV/SZ/VHINP-3 polymerase (ORF1b) gene, copy2	GGCACCGGGGCCACGCCGAGTAGGAACGAGGGTACAGTGCC
KX397575.1 Chicken astrovirus isolate CC_CkAstV, copy2	GGCACCGGGGCCACGCCGAGTAGGAACGAGGGTACAGTGCC
KX397576.1 Chicken astrovirus isolate CkP5, copy2	GGCACCGGGGCCACGCCGAGTAGGAACGAGGGTACAGTGCC
KY038163.1 Chicken astrovirus isolate CAstV/INDIA/ANAND/2016, copy2	GGCACCGGGGCCACGCCGAGTAGGAACGAGGGTACAGTGCC
KY271027.1 Goose astrovirus isolate FLX, copy2	AGCCCCGGGGCCACGCGGAGTACGATCGAGGGTACAGGGCG
MH410610.1 Goose astrovirus strain AHDY, copy2	AGCCCCGGGGCCACGCGGAGTACGATCGAGGGTACAGGGCG
MH453801.1 Avastrovirus 2 isolate AstV/Red-necked Avocet/MW09/Interior genomic sequence , copy2	CTGCCCGAGGCCACGGCGAGTAGCATCGAGGGTACAGGCAG
MK746105.1 Chicken astrovirus isolate AAstV/Chicken/CHN/2017/NJ1701, copy2	GGCACCGGGGCCACGCCGAGTAGGAACGAGGGTACAGTGCC

MN725025.1 Chicken astrovirus strain CAstV/CHN/HBLP717-1/2018, copy2	CACACCGGGGCCTCGCCGAGTAGGAACGAGGGTACAGTGCC
MN725026.1 Chicken astrovirus strain CAstV/CHN/GDYHTJ718-6/2018, copy2	CACACCGGGGCCTCGCCGAGTAGGAACGAGGGTACAGTGCC
NC_034567.1 Goose astrovirus isolate FLX, copy2	AGCCCCGGGGCCACGCGGAGTACGATCGAGGGTACAGGGCG
FR727144.1 Pigeon picornavirus B, copy2	TGACCCGGGGCCACGCCGAGTAGGATCGAGGGTACAGGTCA
HQ166910.1 Zaria bat coronavirus strain ZBCoV, copy2	AACACCGAGGCCACGCCGAGTAGGATCGAGGGTACAGTGTT
KC560801.3 Pigeon picornavirus B strain GAL-7/2010/Hungary, copy2	TGACCCGGGGCCACGCCGAGTAGGATCGAGGGTACAGGTCA
KY684213.1 Pigeon picornavirus B strain 1P RNA-dependent RNA polymerase gene, copy2	TGACCCGGGGCCACGCCGAGTAGGATCGAGGGTACAGGTCA
KY684214.1 Pigeon picornavirus B strain 13P RNA-dependent RNA polymerase gene, copy2	TGACCCGGGGCCACGCCGAGTAGGATCGAGGGTACAGGTCA
KY684215.1 Pigeon picornavirus B strain 23P RNA-dependent RNA polymerase gene, copy2	TGACCCGGGGCCACGCCGAGTAGGATCGAGGGTACAGGTCA
MF373609.1 Bat norovirus isolate NPIH26, copy2	GACGCCGCGGCCACGCCGAGTAGGATCGAGGGCACAGCGTC
MK204389.1 Sharp-tailed sandpiper Picornavirus B-like polyprotein gene, copy2	GAGGCCGCGGCCACGCGGAGTACGATCGAGGGTACAGCCTC
NC_015626.1 Pigeon picornavirus B, copy2	TGACCCGGGGCCACGCCGAGTAGGATCGAGGGTACAGGTCA
FJ571066.1 Bat astrovirus Tm/Guangxi/LD77/2007 non-structural polyprotein 1AB (pol) gene, copy1	GCGACCGAGGCCACGCCGAGTAGGATCGAGGGTACAGTCAC
FJ692500.1 Norovirus dog/GVI.1/HKU_Ca026F/2007/HKG ORF1 polyprotein, copy1	TGCGCCGCGGCCACGCCGAGTAGGATCGAGGGTACAGCGCA
FJ692501.1 Norovirus dog/GVI.1/HKU_Ca035F/2007/HKG ORF1 polyprotein, copy1	TGCGCCGCGGCCACGCCGAGTAGGATCGAGGGTACAGCGCA
FR727144.1 Pigeon picornavirus B, copy1	TGACCCGGGGCCACGCCGAGTAGGATCGAGGGTACAGGTCA
FR727145.1 Pigeon picornavirus A, copy1	GAGACCGAGGCCACGCCGAGTAGGATCGAGGGTACAGTCTC
HQ166910.1 Zaria bat coronavirus strain ZBCoV, copy1	TAGACCGAGGCCACGCCGAGTAGGATCGAGGGTACAGTTTA
JF414802.1 Chicken astrovirus isolate GA2011, copy1	GCAGCCGCGGCCACGCCGAGTAGGATCGAGGGTACAGCTGC
JN582327.1 Chicken astrovirus isolate 11672 polymerase gene, copy1	GCAGCCGCGGCCACGCCGAGTAGGATCGAGGGTACAGCTGC
JN582328.1 Chicken astrovirus isolate FP3 polymerase gene, copy1	GCAGCCGCGGCCACGCCGAGTAGGATCGAGGGTACAGCTGC
KC560801.3 Pigeon picornavirus B strain GAL-7/2010/Hungary, copy1	TGACCCGGGGCCACGCCGAGTAGGATCGAGGGTACAGGTCA
KJ621028.1 Chicken astrovirus isolate VRDC/CAstV/SZ/VHINP-2 capsid protein (ORF2) gene, copy1	GCAGCCGCGGCCACGCCGAGTAGGATCGAGGGTACAGCTGC
KJ621029.1 Chicken astrovirus isolate VRDC/CAstV/SZ/VHINP-3 polymerase (ORF1b) gene, copy1	GCAGCCGCGGCCACGCCGAGTAGGATCGAGGGTACAGCTGC
KJ790198.1 Bat norovirus polyprotein gene, copy1	GCAGCCGCGGCCACGCCGAGTAGGACCGAGGGTACAGCTGC
KX397575.1 Chicken astrovirus isolate CC_CkAstV, copy1	GCAGCCGCGGCCACGCCGAGTAGGAACGAGGGTACAGCTGC
KX397576.1 Chicken astrovirus isolate CkP5, copy1	GCAGCCGCGGCCACGCCGAGTAGGAACGAGGGTACAGCTGC
KY038163.1 Chicken astrovirus isolate CAstV/INDIA/ANAND/2016, copy1	GCAGCCGCGGCCACGCCGAGTAGGATCGAGGGTACAGCTGC
KY271027.1 Goose astrovirus isolate FLX, copy1	GCAGCCGCGGCCACGCCGAGTAGGATCGAGGGTACAGCTGC
KY684213.1 Pigeon picornavirus B strain 1P RNA-dependent RNA polymerase gene, copy1	TGACCCGGGGCCACGCCGAGTAGGATCGAGGGTACAGGTCA
KY684214.1 Pigeon picornavirus B strain 13P RNA-dependent RNA polymerase gene, copy1	TGACCCGGGGCCACGCCGAGTAGGATCGAGGGTACAGGTCA
KY684215.1 Pigeon picornavirus B strain 23P RNA-dependent RNA polymerase gene, copy1	TGACCCGGGGCCACGCCGAGTAGGATCGAGGGTACAGGTCA
MF373609.1 Bat norovirus isolate NPIH26, copy1	GACGCCGCGGCCACGCCGAGTAGGATCGAGGGCACAGCGTC

MH410610.1 Goose astrovirus strain AHDY, copy1	GCAGCCGCGGCCACGCCGAGTAGGATCGAGGGTACAGCTGC
MH453801.1 Avastrovirus 2 isolate AstV/Red-necked Avocet/MW09/Interior genomic sequence , copy1	CTGCCCGAGGCCACGGCGAGTAGCATCGAGGGTACAGGCAG
MK204389.1 Sharp-tailed sandpiper Picornavirus B-like polyprotein gene, copy1	TCAACCGAGGCCACGCGGAGTACGATCGAGGGTACAGTTGA
MK746105.1 Chicken astrovirus isolate AAstV/Chicken/CHN/2017/NJ1701, copy1	GCAGCCGCGGCCACGCCGAGTAGGATCGAGGGTACAGCTGC
MN725025.1 Chicken astrovirus strain CAstV/CHN/HBLP717-1/2018, copy1	GCAGCCGCGGCCACGCCGAGTAGGATCGAGGGTACAGCTGC
MN725026.1 Chicken astrovirus strain CAstV/CHN/GDYHTJ718-6/2018, copy1	GCAGCCGCGGCCACGCCGAGTAGGATCGAGGGTACAGCTGC
NC_015626.1 Pigeon picornavirus B, copy1	TGACCCGGGGCCACGCCGAGTAGGATCGAGGGTACAGGTCA
NC_034567.1 Goose astrovirus isolate FLX, copy1	GCAGCCGCGGCCACGCCGAGTAGGATCGAGGGTACAGCTGC
NC_043100.1 Bat astrovirus Tm/Guangxi/LD77/2007 non-structural polyprotein 1AB (pol) gene, copy1	GCGACCGAGGCCACGCCGAGTAGGATCGAGGGTACAGTCAC
NC_044047.1 Norovirus dog/GVI.1/HKU_Ca026F/2007/HKG ORF1 polyprotein, copy1	TGCGCCGCGGCCACGCCGAGTAGGATCGAGGGTACAGCGCA
FR727145.1 Pigeon picornavirus A, copy2	GAGACCGAGGCCACGCCGAGTAGGATCGAGGGTACAGTCTC
JQ081297.1 UNVERIFIED: Dog astrovirus ORF1a-like gene, partial sequence; ORF1b gene, partial cds; and ORF2 gene,	GTTCCCGAGGCCACGCCGAGTAGGATCGAGGGAATCTCTAG
MN622961.1 Avian coronavirus strain PNRJ46 3' UTR	AGTGCCGGGGCCACGCGGAGTACGATCGAGGGTACTTGGAA
MN920667.1 Astroviridae sp. isolate tom152ast2, complete genome	ATGACCGAGGCCACGGCGAGTAGCATCGAGGGTACAGTCAT
MT025105.1 Fuchs virus isolate Antarctic41 non-structural polyprotein and hypothetical protein genes, complete cds	ACTACCGAGGCCACGGCGAGTAGCATCGAGGGTACAGTAGT
MT040334.1 Pangolin coronavirus isolate PCoV_GX-P1E, complete genome	TAAACCGAGGCCACGCGGAGTACGATCGAGGGTACAGCCAA
GQ504720.1 Infectious bronchitis virus strain Arkansas DPI, complete genome	AGTGCCGGGGCCACGCGGAGTACGATCTAGGGTTACAGCACT
AY179509.1 Mink astrovirus, complete genome	CTACCCGAGGCCACGCCGAGTTAGGATCGAGGGTACAGGTAG
GU393332.1 Infectious bronchitis virus serotype Delaware 072, complete genome	TGTGCCGAGGGCCACGCGGAGTACGATCGAGGGTACAGCACT
NC_004579.1 Mink astrovirus, complete genome	CTACCCGAGGCCACGCCGAGTTAGGATCGAGGGTACAGGTAG
MT385439.1 Severe acute respiratory syndrome coronavirus 2 isolate SARS-CoV-2/human/USA/CA-CZB0100/2020, co	TTCACCGAGGCCACGCGGAGTACGATCGAGTGTACAGTGGG
LC364344.1 Pigeon coronavirus UAE-HKU29 271F genomic RNA, complete genome	TGAACCGAGGCCACGCGGAGTACGATCGATGGTACAGTTCA
MT215193.1 Severe acute respiratory syndrome coronavirus 2 isolate SARS-CoV-2/canine/HKG/20-02756/2020 ORF1	TTTACCGAGGCCACGCGGAGTACGATCGAGTGTACAGTGAA
MT293192.1 Severe acute respiratory syndrome coronavirus 2 isolate SARS-CoV-2/human/USACT-UW427/2020, com	TTCACTGAGGCCACGCGGAGTACGATCGAGTGTACAGTGAA
MT293219.1 Severe acute respiratory syndrome coronavirus 2 isolate SARS-CoV-2/human/USA/WA-UW-1440/2020,	TTCACCCAGGCCACGCGGAGTACGATCGAGTGTACAGTGAA
MT325599.1 Severe acute respiratory syndrome coronavirus 2 isolate SARS-CoV-2/human/USA/PA_1802/2020, comp	TTCACCCAGGCCACGCGGAGTACGATCGAGTGTACAGTGAA
MT326076.1 Severe acute respiratory syndrome coronavirus 2 isolate SARS-CoV-2/human/USA/WA-UW-1944/2020,	TTCACCGAGGCCACGCGGAGTACGATCGAGCGTACAGTGAA
MT350257.1 Severe acute respiratory syndrome coronavirus 2 isolate SARS-CoV-2/human/USA/VA-DCLS-0068/2020,	TTCACTGAGGCCACGCGGAGTACGATCGAGTGTACAGTGAA
MT374111.1 Severe acute respiratory syndrome coronavirus 2 isolate SARS-CoV-2/human/TWN/CGMH-CGU-15/2020	TTCACCCAGGCCACGCGGAGTACGATCGAGTGTACAGTGAA
MT375477.1 Severe acute respiratory syndrome coronavirus 2 isolate SARS-CoV-2/human/USA/WA-UW-5152/2020,	TTCACCGAGGCCACGCGGAGTACGATCGAGTGTATAGTGAA
MT407657.1 Severe acute respiratory syndrome coronavirus 2 isolate SARS-CoV-2/human/CHN/OS3/2020, complete	TTCACCCAGGCCACGCGGAGTACGATCGAGTGTACAGTGAA
MT450999.1 Severe acute respiratory syndrome coronavirus 2 isolate SARS-CoV-2/human/AUS/VIC89/2020, complet	TTCACCCAGGCCACGCGGAGTACGATCGAGTGTACAGTGAA
MT451061.1 Severe acute respiratory syndrome coronavirus 2 isolate SARS-CoV-2/human/AUS/VIC157/2020, comple	TTCACCCAGGCCACGCGGAGTACGATCGAGTGTACAGTGAA

MT451067.1 Severe acute respiratory syndrome coronavirus 2 isolate SARS-CoV-2/human/AUS/VIC164/2020 ORF1ab	TTCACCCAGGCCACGCGGAGTACGATCGAGTGTACAGTGAA
MT451097.1 Severe acute respiratory syndrome coronavirus 2 isolate SARS-CoV-2/human/AUS/VIC195/2020 ORF1ab	TTCACCCAGGCCACGCGGAGTACGATCGAGTGTACAGTGAA
MT451166.1 Severe acute respiratory syndrome coronavirus 2 isolate SARS-CoV-2/human/AUS/VIC270/2020, comple	TTCACCGAGGCTACGCGGAGTACGATCGAGTGTACAGTGAA
MT451167.1 Severe acute respiratory syndrome coronavirus 2 isolate SARS-CoV-2/human/AUS/VIC271/2020, comple	TTCACCGAGGCTACGCGGAGTACGATCGAGTGTACAGTGAA
MT451178.1 Severe acute respiratory syndrome coronavirus 2 isolate SARS-CoV-2/human/AUS/VIC284/2020, comple	TTCACCGAGGCTACGCGGAGTACGATCGAGTGTACAGTGAA
MT451228.1 Severe acute respiratory syndrome coronavirus 2 isolate SARS-CoV-2/human/AUS/VIC337/2020 ORF1ab	TTCACCGAGGCTACGCGGAGTACGATCGAGTGTACAGTGAA
MT451578.1 Severe acute respiratory syndrome coronavirus 2 isolate SARS-CoV-2/human/AUS/VIC820/2020, comple	TTCATCGAGGCCACGCGGAGTACGATCGAGTGTACAGTGAA
MT451580.1 Severe acute respiratory syndrome coronavirus 2 isolate SARS-CoV-2/human/AUS/VIC823/2020, comple	TTCATCGAGGCCACGCGGAGTACGATCGAGTGTACAGTGAA
MT451682.1 Severe acute respiratory syndrome coronavirus 2 isolate SARS-CoV-2/human/AUS/VIC997/2020 ORF1ab	TTCACCCAGGCCACGCGGAGTACGATCGAGTGTACAGTGAA
MT451711.1 Severe acute respiratory syndrome coronavirus 2 isolate SARS-CoV-2/human/AUS/VIC1041/2020, comp	TTCACCGAGGTCACGCGGAGTACGATCGAGTGTACAGTGAA
MT509498.1 Severe acute respiratory syndrome coronavirus 2 isolate SARS-CoV-2/human/IND/GBRC107/2020, comp	TTCACCTAGGCCACGCGGAGTACGATCGAGTGTACAGTGAA
MT509499.1 Severe acute respiratory syndrome coronavirus 2 isolate SARS-CoV-2/human/IND/GBRC104/2020, comp	TTCACCTAGGCCACGCGGAGTACGATCGAGTGTACAGTGAA
MT509508.1 Severe acute respiratory syndrome coronavirus 2 isolate SARS-CoV-2/human/IND/GBRC106/2020, comp	TTCACCTAGGCCACGCGGAGTACGATCGAGTGTACAGTGAA
MT520376.1 Severe acute respiratory syndrome coronavirus 2 isolate SARS-CoV-2/human/USA/MA_MGH_00159/202	TTCACCGAGGCCACGCGGAGTACCATCGAGTGTACAGTGAA
LC528232.1 Severe acute respiratory syndrome coronavirus 2 SARS-CoV-2/Hu/DP/Kng/19-020 RNA, complete genome	TTCACCGAGGCCACGCGGAGTACGATCGAGTGTACAGTGAA
LC528233.1 Severe acute respiratory syndrome coronavirus 2 SARS-CoV-2/Hu/DP/Kng/19-027 RNA, complete genome	TTCACCGAGGCCACGCGGAGTACGATCGAGTGTACAGTGAA
LC529905.1 Severe acute respiratory syndrome coronavirus 2 TKYE6182_2020 RNA, complete genome	TTCACCGAGGCCACGCGGAGTACGATCGAGTGTACAGTGAA
LC534418.1 Severe acute respiratory syndrome coronavirus 2 SARS-CoV-2/Hu/DP/Kng/19-031 RNA, complete genome	TTCACCGAGGCCACGCGGAGTACGATCGAGTGTACAGTGAA
LC534419.1 Severe acute respiratory syndrome coronavirus 2 SARS-CoV-2/Hu/Kng/19-437 RNA, complete genome	TTCACCGAGGCCACGCGGAGTACGATCGAGTGTACAGTGAA
LC542809.1 Severe acute respiratory syndrome coronavirus 2 TKYE6947_2020 RNA, complete genome	TTCACCGAGGCCACGCGGAGTACGATCGAGTGTACAGTGAA
LC542976.1 Severe acute respiratory syndrome coronavirus 2 TKYE6968_2020 RNA, complete genome	TTCACCGAGGCCACGCGGAGTACGATCGAGTGTACAGTGAA
LC546038.1 Severe acute respiratory syndrome coronavirus 2 SARS-CoV-2/Hu/Kng/19-865 RNA, complete genome	TTCACCGAGGCCACGCGGAGTACGATCGAGTGTACAGTGAA
LC547518.1 Severe acute respiratory syndrome coronavirus 2 hCoV-19/Japan/P1/2020 RNA, complete genome	TTCACCGAGGCCACGCGGAGTACGATCGAGTGTACAGTGAA
LC547519.1 Severe acute respiratory syndrome coronavirus 2 hCoV-19/Japan/P2-1/2020 RNA, complete genome	TTCACCGAGGCCACGCGGAGTACGATCGAGTGTACAGTGAA
LC547520.1 Severe acute respiratory syndrome coronavirus 2 hCoV-19/Japan/P2-2/2020 RNA, complete genome	TTCACCGAGGCCACGCGGAGTACGATCGAGTGTACAGTGAA
LC547521.1 Severe acute respiratory syndrome coronavirus 2 hCoV-19/Japan/P3-1/2020 RNA, complete genome	TTCACCGAGGCCACGCGGAGTACGATCGAGTGTACAGTGAA
LC547522.1 Severe acute respiratory syndrome coronavirus 2 hCoV-19/Japan/P3-2/2020 RNA, complete genome	TTCACCGAGGCCACGCGGAGTACGATCGAGTGTACAGTGAA
LC547523.1 Severe acute respiratory syndrome coronavirus 2 hCoV-19/Japan/P4-1/2020 RNA, complete genome	TTCACCGAGGCCACGCGGAGTACGATCGAGTGTACAGTGAA
LC547524.1 Severe acute respiratory syndrome coronavirus 2 hCoV-19/Japan/P4-2/2020 RNA, complete genome	TTCACCGAGGCCACGCGGAGTACGATCGAGTGTACAGTGAA
LC547525.1 Severe acute respiratory syndrome coronavirus 2 hCoV-19/Japan/P4-3/2020 RNA, complete genome	TTCACCGAGGCCACGCGGAGTACGATCGAGTGTACAGTGAA
LC547526.1 Severe acute respiratory syndrome coronavirus 2 hCoV-19/Japan/P4-4/2020 RNA, complete genome	TTCACCGAGGCCACGCGGAGTACGATCGAGTGTACAGTGAA
LC547527.1 Severe acute respiratory syndrome coronavirus 2 hCoV-19/Japan/P4-5/2020 RNA, complete genome	TTCACCGAGGCCACGCGGAGTACGATCGAGTGTACAGTGAA
LC547528.1 Severe acute respiratory syndrome coronavirus 2 hCoV-19/Japan/P4-6/2020 RNA, complete genome	TTCACCGAGGCCACGCGGAGTACGATCGAGTGTACAGTGAA

LC547529.1 Severe acute respiratory syndrome coronavirus 2 hCoV-19/Japan/P4-7/2020 RNA, complete genome	TTCACCGAGGCCACGCGGAGTACGATCGAGTGTACAGTGAA
LC547530.1 Severe acute respiratory syndrome coronavirus 2 hCoV-19/Japan/P4-8/2020 RNA, complete genome	TTCACCGAGGCCACGCGGAGTACGATCGAGTGTACAGTGAA
LC547531.1 Severe acute respiratory syndrome coronavirus 2 hCoV-19/Japan/P5-1/2020 RNA, complete genome	TTCACCGAGGCCACGCGGAGTACGATCGAGTGTACAGTGAA
LC547532.1 Severe acute respiratory syndrome coronavirus 2 hCoV-19/Japan/P5-2/2020 RNA, complete genome	TTCACCGAGGCCACGCGGAGTACGATCGAGTGTACAGTGAA
LC547533.1 Severe acute respiratory syndrome coronavirus 2 hCoV-19/Japan/P5-3/2020 RNA, complete genome	TTCACCGAGGCCACGCGGAGTACGATCGAGTGTACAGTGAA
LR757995.1 Wuhan seafood market pneumonia virus genome assembly, chromosome: whole_genome	TTCACCGAGGCCACGCGGAGTACGATCGAGTGTACAGTGAA
LR757996.1 Wuhan seafood market pneumonia virus genome assembly, chromosome: whole_genome	TTCACCGAGGCCACGCGGAGTACGATCGAGTGTACAGTGAA
LR757998.1 Wuhan seafood market pneumonia virus genome assembly, chromosome: whole_genome	TTCACCGAGGCCACGCGGAGTACGATCGAGTGTACAGTGAA
MF642322.1 Porcine deltacoronavirus strain CHN/GS/2016/1, complete genome	TATGCCGAGGCCACGCGGAGCACGATCGAGGCTACAGCATA
MF642323.1 Porcine deltacoronavirus strain CHN/GS/2016/2, complete genome	TATGCCGAGGCCACGCGGAGCATGATCGAGGCTACATCATA
MF642324.1 Porcine deltacoronavirus strain CHN/GS/2017/1, complete genome	TATGCCGAGGCCACGCGGAGCATGATCGAGGCTACATCATA
MF642325.1 Porcine deltacoronavirus strain CHN/QH/2017/1, complete genome	TATGCCGAGGCCACGCGGAGCATGATCGAGGCTACATCATA
MN908947.3 Severe acute respiratory syndrome coronavirus 2 isolate Wuhan-Hu-1, complete genome	TTCACCGAGGCCACGCGGAGTACGATCGAGTGTACAGTGAA
MN938384.1 Severe acute respiratory syndrome coronavirus 2 isolate 2019-nCoV_HKU-SZ-002a_2020, complete gen	TTCACCGAGGCCACGCGGAGTACGATCGAGTGTACAGTGAA
MN975262.1 Severe acute respiratory syndrome coronavirus 2 isolate 2019-nCoV_HKU-SZ-005b_2020, complete gen	TTCACCGAGGCCACGCGGAGTACGATCGAGTGTACAGTGAA
MN985325.1 Severe acute respiratory syndrome coronavirus 2 isolate SARS-CoV-2/human/USA/USA-WA1/2020, com	TTCACCGAGGCCACGCGGAGTACGATCGAGTGTACAGTGAA
MN988668.1 Severe acute respiratory syndrome coronavirus 2 isolate 2019-nCoV WHU01, complete genome	TTCACCGAGGCCACGCGGAGTACGATCGAGTGTACAGTGAA
MN988669.1 Severe acute respiratory syndrome coronavirus 2 isolate 2019-nCoV WHU02, complete genome	TTCACCGAGGCCACGCGGAGTACGATCGAGTGTACAGTGAA
MN988713.1 Severe acute respiratory syndrome coronavirus 2 isolate 2019-nCoV/USA-IL1/2020, complete genome	TTCACCGAGGCCACGCGGAGTACGATCGAGTGTACAGTGAA
MN994467.1 Severe acute respiratory syndrome coronavirus 2 isolate 2019-nCoV/USA-CA1/2020, complete genome	TTCACCGAGGCCACGCGGAGTACGATCGAGTGTACAGTGAA
MN994468.1 Severe acute respiratory syndrome coronavirus 2 isolate 2019-nCoV/USA-CA2/2020, complete genome	TTCACCGAGGCCACGCGGAGTACGATCGAGTGTACAGTGAA
MN996527.1 Severe acute respiratory syndrome coronavirus 2 isolate WIV02, complete genome	TTCACCGAGGCCACGCGGAGTACGATCGAGTGTACAGTGAA
MN996528.1 Severe acute respiratory syndrome coronavirus 2 isolate WIV04, complete genome	TTCACCGAGGCCACGCGGAGTACGATCGAGTGTACAGTGAA
MN996529.1 Severe acute respiratory syndrome coronavirus 2 isolate WIV05, complete genome	TTCACCGAGGCCACGCGGAGTACGATCGAGTGTACAGTGAA
MN996530.1 Severe acute respiratory syndrome coronavirus 2 isolate WIV06, complete genome	TTCACCGAGGCCACGCGGAGTACGATCGAGTGTACAGTGAA
MN996531.1 Severe acute respiratory syndrome coronavirus 2 isolate WIV07, complete genome	TTCACCGAGGCCACGCGGAGTACGATCGAGTGTACAGTGAA
MN997409.1 Severe acute respiratory syndrome coronavirus 2 isolate 2019-nCoV/USA-AZ1/2020, complete genome	TTCACCGAGGCCACGCGGAGTACGATCGAGTGTACAGTGAA
MT012098.1 Severe acute respiratory syndrome coronavirus 2 isolate SARS-CoV-2/human/IND/29/2020, complete ge	TTCACCGAGGCCACGCGGAGTACGATCGAGTGTACAGTGAA
MT019529.1 Severe acute respiratory syndrome coronavirus 2 isolate BetaCoV/Wuhan/IPBCAMS-WH-01/2019, comp	TTCACCGAGGCCACGCGGAGTACGATCGAGTGTACAGTGAA
MT019530.1 Severe acute respiratory syndrome coronavirus 2 isolate BetaCoV/Wuhan/IPBCAMS-WH-02/2019, comp	TTCACCGAGGCCACGCGGAGTACGATCGAGTGTACAGTGAA
MT019531.1 Severe acute respiratory syndrome coronavirus 2 isolate BetaCoV/Wuhan/IPBCAMS-WH-03/2019, comp	TTCACCGAGGCCACGCGGAGTACGATCGAGTGTACAGTGAA
MT019532.1 Severe acute respiratory syndrome coronavirus 2 isolate BetaCoV/Wuhan/IPBCAMS-WH-04/2019, comp	TTCACCGAGGCCACGCGGAGTACGATCGAGTGTACAGTGAA
MT019533.1 Severe acute respiratory syndrome coronavirus 2 isolate BetaCoV/Wuhan/IPBCAMS-WH-05/2020, comp	TTCACCGAGGCCACGCGGAGTACGATCGAGTGTACAGTGAA

MT020781.2 Severe acute respiratory syndrome coronavirus 2 isolate nCoV-FIN-29-Jan-2020, partial genome	TTCACCGAGGCCACGCGGAGTACGATCGAGTGTACAGTGAA
MT020880.1 Severe acute respiratory syndrome coronavirus 2 isolate 2019-nCoV/USA-WA1-A12/2020, complete gen	TTCACCGAGGCCACGCGGAGTACGATCGAGTGTACAGTGAA
MT020881.1 Severe acute respiratory syndrome coronavirus 2 isolate 2019-nCoV/USA-WA1-F6/2020, complete geno	TTCACCGAGGCCACGCGGAGTACGATCGAGTGTACAGTGAA
MT027062.1 Severe acute respiratory syndrome coronavirus 2 isolate 2019-nCoV/USA-CA3/2020, complete genome	TTCACCGAGGCCACGCGGAGTACGATCGAGTGTACAGTGAA
MT027063.1 Severe acute respiratory syndrome coronavirus 2 isolate 2019-nCoV/USA-CA4/2020, complete genome	TTCACCGAGGCCACGCGGAGTACGATCGAGTGTACAGTGAA
MT027064.1 Severe acute respiratory syndrome coronavirus 2 isolate 2019-nCoV/USA-CA5/2020, complete genome	TTCACCGAGGCCACGCGGAGTACGATCGAGTGTACAGTGAA
MT039873.1 Severe acute respiratory syndrome coronavirus 2 isolate HZ-1, complete genome	TTCACCGAGGCCACGCGGAGTACGATCGAGTGTACAGTGAA
MT039874.1 Severe acute respiratory syndrome coronavirus 2 isolate SARS-CoV-2/human/CHN/IME-HZ01/2020, com	TTCACCGAGGCCACGCGGAGTACGATCGAGTGTACAGTGAA
MT039887.1 Severe acute respiratory syndrome coronavirus 2 isolate 2019-nCoV/USA-WI1/2020, complete genome	TTCACCGAGGCCACGCGGAGTACGATCGAGTGTACAGTGAA
MT039888.1 Severe acute respiratory syndrome coronavirus 2 isolate 2019-nCoV/USA-MA1/2020, complete genome	TTCACCGAGGCCACGCGGAGTACGATCGAGTGTACAGTGAA
MT039890.1 Severe acute respiratory syndrome coronavirus 2 isolate SNU01, complete genome	TTCACCGAGGCCACGCGGAGTACGATCGAGTGTACAGTGAA
MT044257.1 Severe acute respiratory syndrome coronavirus 2 isolate 2019-nCoV/USA-IL2/2020, complete genome	TTCACCGAGGCCACGCGGAGTACGATCGAGTGTACAGTGAA
MT044258.1 Severe acute respiratory syndrome coronavirus 2 isolate 2019-nCoV/USA-CA6/2020, complete genome	TTCACCGAGGCCACGCGGAGTACGATCGAGTGTACAGTGAA
MT049951.1 Severe acute respiratory syndrome coronavirus 2 isolate SARS-CoV-2/human/CHN/Yunnan-01/2020, cor	TTCACCGAGGCCACGCGGAGTACGATCGAGTGTACAGTGAA
MT050493.1 Severe acute respiratory syndrome coronavirus 2 isolate SARS-CoV-2/human/IND/166/2020, complete g	TTCACCGAGGCCACGCGGAGTACGATCGAGTGTACAGTGAA
MT066156.1 Severe acute respiratory syndrome coronavirus 2 isolate SARS-CoV-2/human/ITA/INMI1/2020, complete	TTCACCGAGGCCACGCGGAGTACGATCGAGTGTACAGTGAA
MT066175.1 Severe acute respiratory syndrome coronavirus 2 isolate SARS-CoV-2/NTU01/TWN/human/2020, comple	TTCACCGAGGCCACGCGGAGTACGATCGAGTGTACAGTGAA
MT066176.1 Severe acute respiratory syndrome coronavirus 2 isolate SARS-CoV-2/NTU02/TWN/human/2020, comple	TTCACCGAGGCCACGCGGAGTACGATCGAGTGTACAGTGAA
MT072688.1 Severe acute respiratory syndrome coronavirus 2 isolate SARS-CoV-2/human/NPL/61-TW/2020, complet	TTCACCGAGGCCACGCGGAGTACGATCGAGTGTACAGTGAA
MT077125.1 Severe acute respiratory syndrome coronavirus 2 isolate SARS-CoV-2/human/ITA/INMI1/2020, complete	TTCACCGAGGCCACGCGGAGTACGATCGAGTGTACAGTGAA
MT079843.1 Severe acute respiratory syndrome coronavirus 2 isolate SARS-CoV-2/human/CHN/WHUHnCoV001/2020	TTCACCGAGGCCACGCGGAGTACGATCGAGTGTACAGTGAA
MT079844.1 Severe acute respiratory syndrome coronavirus 2 isolate SARS-CoV-2/human/CHN/WHUHnCoV002/2020	TTCACCGAGGCCACGCGGAGTACGATCGAGTGTACAGTGAA
MT079845.1 Severe acute respiratory syndrome coronavirus 2 isolate SARS-CoV-2/human/CHN/WHUHnCoV003/2020	TTCACCGAGGCCACGCGGAGTACGATCGAGTGTACAGTGAA
MT079846.1 Severe acute respiratory syndrome coronavirus 2 isolate SARS-CoV-2/human/CHN/WHUHnCoV004/2020	TTCACCGAGGCCACGCGGAGTACGATCGAGTGTACAGTGAA
MT079847.1 Severe acute respiratory syndrome coronavirus 2 isolate SARS-CoV-2/human/CHN/WHUHnCoV005/2020	TTCACCGAGGCCACGCGGAGTACGATCGAGTGTACAGTGAA
MT079848.1 Severe acute respiratory syndrome coronavirus 2 isolate SARS-CoV-2/human/CHN/WHUHnCoV006/2020	TTCACCGAGGCCACGCGGAGTACGATCGAGTGTACAGTGAA
MT079849.1 Severe acute respiratory syndrome coronavirus 2 isolate SARS-CoV-2/human/CHN/WHUHnCoV007/2020	TTCACCGAGGCCACGCGGAGTACGATCGAGTGTACAGTGAA
MT079850.1 Severe acute respiratory syndrome coronavirus 2 isolate SARS-CoV-2/human/CHN/WHUHnCoV008/2020	TTCACCGAGGCCACGCGGAGTACGATCGAGTGTACAGTGAA
MT079851.1 Severe acute respiratory syndrome coronavirus 2 isolate SARS-CoV-2/human/CHN/WHUHnCoV011/2020	TTCACCGAGGCCACGCGGAGTACGATCGAGTGTACAGTGAA
MT079852.1 Severe acute respiratory syndrome coronavirus 2 isolate SARS-CoV-2/human/CHN/WHUHnCoV012/2020	TTCACCGAGGCCACGCGGAGTACGATCGAGTGTACAGTGAA
MT079853.1 Severe acute respiratory syndrome coronavirus 2 isolate SARS-CoV-2/human/CHN/WHUHnCoV020/2020	TTCACCGAGGCCACGCGGAGTACGATCGAGTGTACAGTGAA
MT079854.1 Severe acute respiratory syndrome coronavirus 2 isolate SARS-CoV-2/human/CHN/WHUHnCoV021/2020	TTCACCGAGGCCACGCGGAGTACGATCGAGTGTACAGTGAA
MT093571.1 Severe acute respiratory syndrome coronavirus 2 isolate SARS-CoV-2/human/SWE/01/2020, complete g	TTCACCGAGGCCACGCGGAGTACGATCGAGTGTACAGTGAA

MT093631.2 Severe acute respiratory syndrome coronavirus 2 isolate SARS-CoV-2/human/CHN/WH-09/2020, comple	TTCACCGAGGCCACGCGGAGTACGATCGAGTGTACAGTGAA
MT106052.1 Severe acute respiratory syndrome coronavirus 2 isolate 2019-nCoV/USA-CA7/2020, complete genome	TTCACCGAGGCCACACGGAGTACGATCGAGTGTACAGTGAA
MT106053.1 Severe acute respiratory syndrome coronavirus 2 isolate 2019-nCoV/USA-CA8/2020, complete genome	TTCACCGAGGCCACGCGGAGTACGATCGAGTGTACAGTGAA
MT106054.1 Severe acute respiratory syndrome coronavirus 2 isolate 2019-nCoV/USA-TX1/2020, complete genome	TTCACCGAGGCCACGCGGAGTACGATCGAGTGTACAGTGAA
MT114412.1 Severe acute respiratory syndrome coronavirus 2 isolate SARS-CoV-2/human/HKG/HKU-902a/2020, com	TTCACCGAGGCCACGCGGAGTACGATCGAGTGTACAGTGAA
MT114413.1 Severe acute respiratory syndrome coronavirus 2 isolate SARS-CoV-2/human/HKG/HKU-902b/2020, com	TTCACCGAGGCCACGCGGAGTACGATCGAGTGTACAGTGAA
MT114416.1 Severe acute respiratory syndrome coronavirus 2 isolate SARS-CoV-2/human/HKG/HKU-907a/2020, com	TTCACCGAGGCCACGCGGAGTACGATCGAGTGTACAGTGAA
MT114417.1 Severe acute respiratory syndrome coronavirus 2 isolate SARS-CoV-2/human/HKG/HKU-907b/2020, com	TTCACCGAGGCCACGCGGAGTACGATCGAGTGTACAGTGAA
MT114418.1 Severe acute respiratory syndrome coronavirus 2 isolate SARS-CoV-2/human/HKG/HKU-908a/2020, com	TTCACCGAGGCCACGCGGAGTACGATCGAGTGTACAGTGAA
MT114419.1 Severe acute respiratory syndrome coronavirus 2 isolate SARS-CoV-2/human/HKG/HKU-908b/2020, com	TTCACCGAGGCCACGCGGAGTACGATCGAGTGTACAGTGAA
MT118835.1 Severe acute respiratory syndrome coronavirus 2 isolate 2019-nCoV/USA-CA9/2020, complete genome	TTCACCGAGGCCACGCGGAGTACGATCGAGTGTACAGTGAA
MT121215.1 Severe acute respiratory syndrome coronavirus 2 isolate SARS-CoV-2/human/CHN/SH01/2020, complete	TTCACCGAGGCCACGCGGAGTACGATCGAGTGTACAGTGAA
MT123290.1 Severe acute respiratory syndrome coronavirus 2 isolate SARS-CoV-2/human/CHN/IQTC01/2020, comple	TTCACCGAGGCCACGCGGAGTACGATCGAGTGTACAGTGAA
MT123291.2 Severe acute respiratory syndrome coronavirus 2 isolate SARS-CoV-2/human/CHN/IQTC02/2020, comple	TTCACCGAGGCCACGCGGAGTACGATCGAGTGTACAGTGAA
MT123292.2 Severe acute respiratory syndrome coronavirus 2 isolate SARS-CoV-2/human/CHN/IQTC04/2020, comple	TTCACCGAGGCCACGCGGAGTACGATCGAGTGTACAGTGAA
MT123293.2 Severe acute respiratory syndrome coronavirus 2 isolate SARS-CoV-2/human/CHN/IQTC03/2020, comple	TTCACCGAGGCCACGCGGAGTACGATCGAGTGTACAGTGAA
MT126808.1 Severe acute respiratory syndrome coronavirus 2 isolate SARS-CoV-2/human/BRA/SP02/2020, complete	TTCACCGAGGCCACGCGGAGTACGATCGAGTGTACAGTGAA
MT135041.1 Severe acute respiratory syndrome coronavirus 2 isolate SARS-CoV-2/human/CHN/105/2020, complete	TTCACCGAGGCCACGCGGAGTACGATCGAGTGTACAGTGAA
MT135042.1 Severe acute respiratory syndrome coronavirus 2 isolate SARS-CoV-2/human/CHN/231/2020, complete	TTCACCGAGGCCACGCGGAGTACGATCGAGTGTACAGTGAA
MT135043.1 Severe acute respiratory syndrome coronavirus 2 isolate SARS-CoV-2/human/CHN/233/2020, complete	TTCACCGAGGCCACGCGGAGTACGATCGAGTGTACAGTGAA
MT135044.1 Severe acute respiratory syndrome coronavirus 2 isolate SARS-CoV-2/human/CHN/235/2020, complete	TTCACCGAGGCCACGCGGAGTACGATCGAGTGTACAGTGAA
MT152824.1 Severe acute respiratory syndrome coronavirus 2 isolate SARS-CoV-2/human/USA/WA2/2020, complete	TTCACCGAGGCCACGCGGAGTACGATCGAGTGTACAGTGAA
MT159705.2 Severe acute respiratory syndrome coronavirus 2 isolate SARS-CoV-2/human/USA/CruiseA-7/2020, com	TTCACCGAGGCCACGCGGAGTACGATCGAGTGTACAGTGAA
MT159706.2 Severe acute respiratory syndrome coronavirus 2 isolate SARS-CoV-2/human/USA/CruiseA-8/2020, com	TTCACCGAGGCCACGCGGAGTACGATCGAGTGTACAGTGAA
MT159707.2 Severe acute respiratory syndrome coronavirus 2 isolate SARS-CoV-2/human/USA/CruiseA-10/2020, cor	TTCACCGAGGCCACGCGGAGTACGATCGAGTGTACAGTGAA
MT159708.2 Severe acute respiratory syndrome coronavirus 2 isolate SARS-CoV-2/human/USA/CruiseA-11/2020, cor	TTCACCGAGGCCACGCGGAGTACGATCGAGTGTACAGTGAA
MT159709.2 Severe acute respiratory syndrome coronavirus 2 isolate SARS-CoV-2/human/USA/CruiseA-12/2020, cor	TTCACCGAGGCCACGCGGAGTACGATCGAGTGTACAGTGAA
MT159710.2 Severe acute respiratory syndrome coronavirus 2 isolate SARS-CoV-2/human/USA/CruiseA-9/2020, com	TTCACCGAGGCCACGCGGAGTACGATCGAGTGTACAGTGAA
MT159711.2 Severe acute respiratory syndrome coronavirus 2 isolate SARS-CoV-2/human/USA/CruiseA-13/2020, cor	TTCACCGAGGCCACGCGGAGTACGATCGAGTGTACAGTGAA
MT159712.2 Severe acute respiratory syndrome coronavirus 2 isolate SARS-CoV-2/human/USA/CruiseA-14/2020, cor	TTCACCGAGGCCACGCGGAGTACGATCGAGTGTACAGTGAA
MT159713.2 Severe acute respiratory syndrome coronavirus 2 isolate SARS-CoV-2/human/USA/CruiseA-15/2020, cor	TTCACCGAGGCCACGCGGAGTACGATCGAGTGTACAGTGAA
MT159714.2 Severe acute respiratory syndrome coronavirus 2 isolate SARS-CoV-2/human/USA/CruiseA-16/2020, cor	TTCACCGAGGCCACGCGGAGTACGATCGAGTGTACAGTGAA
MT159715.2 Severe acute respiratory syndrome coronavirus 2 isolate SARS-CoV-2/human/USA/CruiseA-17/2020, cor	TTCACCGAGGCCACGCGGAGTACGATCGAGTGTACAGTGAA

MT159716.2 Severe acute respiratory syndrome coronavirus 2 isolate SARS-CoV-2/human/USA/CruiseA-18/2020, co	TTCACCGAGGCCACGCGGAGTACGATCGAGTGTACAGTGAA
MT159717.2 Severe acute respiratory syndrome coronavirus 2 isolate SARS-CoV-2/human/USA/CruiseA-1/2020, com	TTCACCGAGGCCACGCGGAGTACGATCGAGTGTACAGTGAA
MT159718.2 Severe acute respiratory syndrome coronavirus 2 isolate SARS-CoV-2/human/USA/CruiseA-2/2020, com	TTCACCGATGCCACGCGGAGTACGATCGAGTGTACAGTGAA
MT159719.2 Severe acute respiratory syndrome coronavirus 2 isolate SARS-CoV-2/human/USA/CruiseA-3/2020, com	TTCACCGAGGCCACGCGGAGTACGATCGAGTGTACAGTGAA
MT159720.2 Severe acute respiratory syndrome coronavirus 2 isolate SARS-CoV-2/human/USA/CruiseA-4/2020, com	TTCACCGAGGCCACGCGGAGTACGATCGAGTGTACAGTGAA
MT159721.2 Severe acute respiratory syndrome coronavirus 2 isolate SARS-CoV-2/human/USA/CruiseA-5/2020, com	TTCACCGAGGCCACGCGGAGTACGATCGAGTGTACAGTGAA
MT159722.2 Severe acute respiratory syndrome coronavirus 2 isolate SARS-CoV-2/human/USA/CruiseA-6/2020, com	TTCACCGAGGCCACGCGGAGTACGATCGAGTGTACAGTGAA
MT163716.1 Severe acute respiratory syndrome coronavirus 2 isolate SARS-CoV-2/human/USA/WA3-UW1/2020, co	TTCACCGAGGCCACGCGGAGTACGATCGAGTGTACAGTGAA
MT163717.1 Severe acute respiratory syndrome coronavirus 2 isolate SARS-CoV-2/human/USA/WA4-UW2/2020, co	TTCACCGAGGCCACGCGGAGTACGATCGAGTGTACAGTGAA
MT163718.1 Severe acute respiratory syndrome coronavirus 2 isolate SARS-CoV-2/human/USA/WA6-UW3/2020, co	TTCACCGAGGCCACGCGGAGTACGATCGAGTGTACAGTGAA
MT163719.1 Severe acute respiratory syndrome coronavirus 2 isolate SARS-CoV-2/human/USA/WA7-UW4/2020, co	TTCACCGAGGCCACGCGGAGTACGATCGAGTGTACAGTGAA
MT163720.1 Severe acute respiratory syndrome coronavirus 2 isolate SARS-CoV-2/human/USA/WA8-UW5/2020, par	TTCACCGAGGCCACGCGGAGTACGATCGAGTGTACAGTGAA
MT163721.1 Severe acute respiratory syndrome coronavirus 2 isolate SARS-CoV-2/human/USA/WA9-UW6/2020, par	TTCACCGAGGCCACGCGGAGTACGATCGAGTGTACAGTGAA
MT184907.2 Severe acute respiratory syndrome coronavirus 2 isolate SARS-CoV-2/human/USA/CruiseA-19/2020, co	TTCACCGAGGCCACGCGGAGTACGATCGAGTGTACAGTGAA
MT184908.2 Severe acute respiratory syndrome coronavirus 2 isolate SARS-CoV-2/human/USA/CruiseA-21/2020, co	TTCACCGATGCCACGCGGAGTACCATCGAGTGTACAGTGAA
MT184909.2 Severe acute respiratory syndrome coronavirus 2 isolate SARS-CoV-2/human/USA/CruiseA-22/2020, co	TTCACCGAGGCCACGCGGAGTACGATCGAGTGTACAGTGAA
MT184910.1 Severe acute respiratory syndrome coronavirus 2 isolate 2019-nCoV/USA-CruiseA-23/2020, complete ge	TTCACCGATGCCACGCGGAGTACCATCGAGTGTACAGTGAA
MT184911.1 Severe acute respiratory syndrome coronavirus 2 isolate 2019-nCoV/USA-CruiseA-24/2020, complete ge	TTCACCGAGGCCACGCGGAGTACGATCGAGTGTACAGTGAA
MT184912.1 Severe acute respiratory syndrome coronavirus 2 isolate 2019-nCoV/USA-CruiseA-25/2020, complete ge	TTCACCGAGGCCACGCGGAGTACGATCGAGTGTACAGTGAA
MT184913.1 Severe acute respiratory syndrome coronavirus 2 isolate 2019-nCoV/USA-CruiseA-26/2020, complete ge	TTCACCGAGGCCACGCGGAGTACGATCGAGTGTACAGTGAA
MT186683.1 Severe acute respiratory syndrome coronavirus 2 isolate SARS-CoV-2/human/HKG/HK20/2020, complete	TTCACCGAGGCCACGCGGAGTACGATCGAGTGTACAGTGAA
MT188339.1 Severe acute respiratory syndrome coronavirus 2 isolate USA/MN3-MDH3/2020, complete genome	TTCACCGAGGCCACGCGGAGTACGATCGAGTGTACAGTGAA
MT188340.1 Severe acute respiratory syndrome coronavirus 2 isolate USA/MN2-MDH2/2020, complete genome	TTCACCGAGGCCACGCGGAGTACGATCGAGTGTACAGTGAA
MT188341.1 Severe acute respiratory syndrome coronavirus 2 isolate USA/MN1-MDH1/2020, complete genome	TTCACCGAGGCCACGCGGAGTACGATCGAGTGTACAGTGAA
MT192759.1 Severe acute respiratory syndrome coronavirus 2 isolate SARS-CoV-2/human/TWN/CGMH-CGU-01/2020	TTCACCGAGGCCACGCGGAGTACGATCGAGTGTACAGTGAA
MT192765.1 Severe acute respiratory syndrome coronavirus 2 isolate SARS-CoV-2/human/USA/PC00101P/2020, com	TTCACCGAGGCCACGCGGAGTACGATCGAGTGTACAGTGAA
MT192772.1 Severe acute respiratory syndrome coronavirus 2 isolate SARS-CoV-2/human/VNM/nCoV-19-01S/2020,	TTCACCGAGGCCACGCGGAGTACGATCGAGTGTACAGTGAA
MT192773.1 Severe acute respiratory syndrome coronavirus 2 isolate SARS-CoV-2/human/VNM/nCoV-19-02S/2020,	TTCACCGAGGCCACGCGGAGTACGATCGAGTGTACAGTGAA
MT198652.2 Severe acute respiratory syndrome coronavirus 2 isolate SARS-CoV-2/human/ESP/Valencia003/2020, co	TTCACCGAGGCCACGCGGAGTACGATCGAGTGTACAGTGAA
MT215194.1 Severe acute respiratory syndrome coronavirus 2 isolate SARS-CoV-2/human/HKG.85_VM20002868/202	TTCACCGAGGCCACGCGGAGTACGATCGAGTGTACAGTGAA
MT215195.1 Severe acute respiratory syndrome coronavirus 2 isolate SARS-CoV-2/human/HKG/90_VM20002907/20	TTCACCGAGGCCACGCGGAGTACGATCGAGTGTACAGTGAA
MT226610.1 Severe acute respiratory syndrome coronavirus 2 isolate SARS-CoV-2/human/CHN/KMS1/2020, complet	TTCACCGAGGCCACGCGGAGTACGATCGAGTGTACAGTGAA
MT230904.1 Severe acute respiratory syndrome coronavirus 2 isolate SARS-CoV-2/human/HKG/HKU-001a/2020, com	TTCACCGAGGCCACGCGGAGTACGATCGAGTGTACAGTGAA

MT233519.1 Severe acute respiratory syndrome coronavirus 2 isolate SARS-CoV-2/human/ESP/Valencia5/2020, comp	TTCACCGAGGCCACGCGGAGTACGATCGAGTGTACAGTGAA
MT233520.1 Severe acute respiratory syndrome coronavirus 2 isolate SARS-CoV-2/human/ESP/Valencia4/2020, parti	TTCACCGAGGCCACGCGGAGTACGATCGAGTGTACAGTGAA
MT233521.1 Severe acute respiratory syndrome coronavirus 2 isolate SARS-CoV-2/human/ESP/Valencia6/2020, parti	TTCACCGAGGCCACGCGGAGTACGATCGAGTGTACAGTGAA
MT233522.1 Severe acute respiratory syndrome coronavirus 2 isolate SARS-CoV-2/human/ESP/Valencia7/2020, comp	TTCACCGAGGCCACGCGGAGTACGATCGAGTGTACAGTGAA
MT233523.1 Severe acute respiratory syndrome coronavirus 2 isolate SARS-CoV-2/human/ESP/Valencia8/2020, comp	TTCACCGAGGCCACGCGGAGTACGATCGAGTGTACAGTGAA
MT233526.1 Severe acute respiratory syndrome coronavirus 2 strain FDAARGOS_983 isolate 2019-nCoV/USA-WA1/2(	TTCACCGAGGCCACGCGGAGTACGATCGAGTGTACAGTGAA
MT240479.1 Severe acute respiratory syndrome coronavirus 2 isolate SARS-CoV-2/human/PAK/Gilgit1/2020, complet	TTCACCGAGGCCACGCGGAGTACGATCGAGTGTACAGTGAA
MT246450.1 Severe acute respiratory syndrome coronavirus 2 isolate SARS-CoV-2/human/USA/WA-UW193/2020, co	TTCACCGAGGCCACGCGGAGTACGATCGAGTGTACAGTGAA
MT246452.1 Severe acute respiratory syndrome coronavirus 2 isolate SARS-CoV-2/human/USA/WA-UW195/2020, co	TTCACCGAGGCCACGCGGAGTACGATCGAGTGTACAGTGAA
MT246455.1 Severe acute respiratory syndrome coronavirus 2 isolate SARS-CoV-2/human/USA/WA-UW198/2020, co	TTCACCGAGGCCACGCGGAGTACGATCGAGTGTACAGTGAA
MT246457.1 Severe acute respiratory syndrome coronavirus 2 isolate SARS-CoV-2/human/USA/WA-UW200/2020, co	TTCACCGAGGCCACGCGGAGTACGATCGAGTGTACAGTGAA
MT246462.1 Severe acute respiratory syndrome coronavirus 2 isolate SARS-CoV-2/human/USA/WA-UW205/2020, co	TTCACCGAGGCCACGCGGAGTACGATCGAGTGTACAGTGAA
MT246464.1 Severe acute respiratory syndrome coronavirus 2 isolate SARS-CoV-2/human/USA/WA-UW207/2020, co	TTCACCGAGGCCACGCGGAGTACGATCGAGTGTACAGTGAA
MT246466.1 Severe acute respiratory syndrome coronavirus 2 isolate SARS-CoV-2/human/USA/WA-UW209/2020, co	TTCACCGAGGCCACGCGGAGTACGATCGAGTGTACAGTGAA
MT246467.1 Severe acute respiratory syndrome coronavirus 2 isolate SARS-CoV-2/human/USA/WA-UW210/2020, co	TTCACCGAGGCCACGCGGAGTACGATCGAGTGTACAGTGAA
MT246468.1 Severe acute respiratory syndrome coronavirus 2 isolate SARS-CoV-2/human/USA/WA-UW211/2020, co	TTCACCGAGGCCACGCGGAGTACGATCGAGTGTACAGTGAA
MT246469.1 Severe acute respiratory syndrome coronavirus 2 isolate SARS-CoV-2/human/USA/WA-UW212/2020, co	TTCACCGAGGCCACGCGGAGTACGATCGAGTGTACAGTGAA
MT246470.1 Severe acute respiratory syndrome coronavirus 2 isolate SARS-CoV-2/human/USA/WA-UW213/2020, co	TTCACCGAGGCCACGCGGAGTACGATCGAGTGTACAGTGAA
MT246471.1 Severe acute respiratory syndrome coronavirus 2 isolate SARS-CoV-2/human/USA/WA-UW214/2020, co	TTCACCGAGGCCACGCGGAGTACGATCGAGTGTACAGTGAA
MT246474.1 Severe acute respiratory syndrome coronavirus 2 isolate SARS-CoV-2/human/USA/WA-UW217/2020, co	TTCACCGAGGCCACGCGGAGTACGATCGAGTGTACAGTGAA
MT246476.1 Severe acute respiratory syndrome coronavirus 2 isolate SARS-CoV-2/human/USA/WA-UW219/2020, co	TTCACCGAGGCCACGCGGAGTACGATCGAGTGTACAGTGAA
MT246477.1 Severe acute respiratory syndrome coronavirus 2 isolate SARS-CoV-2/human/USA/WA-UW220/2020, co	TTCACCGAGGCCACGCGGAGTACGATCGAGTGTACAGTGAA
MT246478.1 Severe acute respiratory syndrome coronavirus 2 isolate SARS-CoV-2/human/USA/WA-UW221/2020, co	TTCACCGAGGCCACGCGGAGTACGATCGAGTGTACAGTGAA
MT246480.1 Severe acute respiratory syndrome coronavirus 2 isolate SARS-CoV-2/human/USA/WA-UW223/2020, co	TTCACCGAGGCCACGCGGAGTACGATCGAGTGTACAGTGAA
MT246481.1 Severe acute respiratory syndrome coronavirus 2 isolate SARS-CoV-2/human/USA/WA-UW224/2020, co	TTCACCGAGGCCACGCGGAGTACGATCGAGTGTACAGTGAA
MT246485.1 Severe acute respiratory syndrome coronavirus 2 isolate SARS-CoV-2/human/USA/WA-UW228/2020, co	TTCACCGAGGCCACGCGGAGTACGATCGAGTGTACAGTGAA
MT246487.1 Severe acute respiratory syndrome coronavirus 2 isolate SARS-CoV-2/human/USA/WA-UW230/2020, co	TTCACCGAGGCCACGCGGAGTACGATCGAGTGTACAGTGAA
MT246488.1 Severe acute respiratory syndrome coronavirus 2 isolate SARS-CoV-2/human/USA/WA-UW231/2020, co	TTCACCGAGGCCACGCGGAGTACGATCGAGTGTACAGTGAA
MT246667.1 Severe acute respiratory syndrome coronavirus 2 strain FDAARGOS_983 isolate 2019-nCoV/USA-WA1/2(	TTCACCGAGGCCACGCGGAGTACGATCGAGTGTACAGTGAA
MT251973.1 Severe acute respiratory syndrome coronavirus 2 isolate SARS-CoV-2/human/USA/WA-UW237/2020, co	TTCACCGAGGCCACGCGGAGTACGATCGAGTGTACAGTGAA
MT251974.1 Severe acute respiratory syndrome coronavirus 2 isolate SARS-CoV-2/human/USA/CT-UW238/2020, con	TTCACCGAGGCCACGCGGAGTACGATCGAGTGTACAGTGAA
MT251976.1 Severe acute respiratory syndrome coronavirus 2 isolate SARS-CoV-2/human/USA/WA-UW240/2020, co	TTCACCGAGGCCACGCGGAGTACGATCGAGTGTACAGTGAA
MT252678.1 Severe acute respiratory syndrome coronavirus 2 isolate SARS-CoV-2/human/USA/WA-UW78/2020, con	TTCACCGAGGCCACGCGGAGTACGATCGAGTGTACAGTGAA

MT252679.1 Severe acute respiratory syndrome coronavirus 2 isolate SARS-CoV-2/human/USA/WA-UW79/2020, com	TTCACCGAGGCCACGCGGAGTACGATCGAGTGTACAGTGAA
MT252680.1 Severe acute respiratory syndrome coronavirus 2 isolate SARS-CoV-2/human/USA/WA-UW80/2020, com	TTCACCGAGGCCACGCGGAGTACGATCGAGTGTACAGTGAA
MT252683.1 Severe acute respiratory syndrome coronavirus 2 isolate SARS-CoV-2/human/USA/WA-UW84/2020, com	TTCACCGAGGCCACGCGGAGTACGATCGAGTGTACAGTGAA
MT252687.1 Severe acute respiratory syndrome coronavirus 2 isolate SARS-CoV-2/human/USA/WA-UW88/2020, com	TTCACCGAGGCCACGCGGAGTACGATCGAGTGTACAGTGAA
MT252688.1 Severe acute respiratory syndrome coronavirus 2 isolate SARS-CoV-2/human/USA/WA-UW89/2020, com	TTCACCGAGGCCACGCGGAGTACGATCGAGTGTACAGTGAA
MT252690.1 Severe acute respiratory syndrome coronavirus 2 isolate SARS-CoV-2/human/USA/WA-UW91/2020, com	TTCACCGAGGCCACGCGGAGTACGATCGAGTGTACAGTGAA
MT252691.1 Severe acute respiratory syndrome coronavirus 2 isolate SARS-CoV-2/human/USA/WA-UW92/2020, com	TTCACCGAGGCCACGCGGAGTACGATCGAGTGTACAGTGAA
MT252692.1 Severe acute respiratory syndrome coronavirus 2 isolate SARS-CoV-2/human/USA/WA-UW93/2020 ORF	TTCACCGAGGCCACGCGGAGTACGATCGAGTGTACAGTGAA
MT252696.1 Severe acute respiratory syndrome coronavirus 2 isolate SARS-CoV-2/human/USA/WA-UW191/2020, co	TTCACCGAGGCCACGCGGAGTACGATCGAGTGTACAGTGAA
MT252697.1 Severe acute respiratory syndrome coronavirus 2 isolate SARS-CoV-2/human/USA/WA-UW178/2020, co	TTCACCGAGGCCACGCGGAGTACGATCGAGTGTACAGTGAA
MT252698.1 Severe acute respiratory syndrome coronavirus 2 isolate SARS-CoV-2/human/USA/WA-UW183/2020, co	TTCACCGAGGCCACGCGGAGTACGATCGAGTGTACAGTGAA
MT252699.1 Severe acute respiratory syndrome coronavirus 2 isolate SARS-CoV-2/human/USA/WA-UW167/2020, co	TTCACCGAGGCCACGCGGAGTACGATCGAGTGTACAGTGAA
MT252700.1 Severe acute respiratory syndrome coronavirus 2 isolate SARS-CoV-2/human/USA/WA-UW168/2020, co	TTCACCGAGGCCACGCGGAGTACGATCGAGTGTACAGTGAA
MT252701.1 Severe acute respiratory syndrome coronavirus 2 isolate SARS-CoV-2/human/USA/WA-UW184/2020, co	TTCACCGAGGCCACGCGGAGTACGATCGAGTGTACAGTGAA
MT252703.1 Severe acute respiratory syndrome coronavirus 2 isolate SARS-CoV-2/human/USA/WA-UW189/2020, co	TTCACCGAGGCCACGCGGAGTACGATCGAGTGTACAGTGAA
MT252704.1 Severe acute respiratory syndrome coronavirus 2 isolate SARS-CoV-2/human/USA/WA-UW190/2020, co	TTCACCGAGGCCACGCGGAGTACGATCGAGTGTACAGTGAA
MT252705.1 Severe acute respiratory syndrome coronavirus 2 isolate SARS-CoV-2/human/USA/WA-UW186/2020, co	TTCACCGAGGCCACGCGGAGTACGATCGAGTGTACAGTGAA
MT252706.1 Severe acute respiratory syndrome coronavirus 2 isolate SARS-CoV-2/human/USA/WA-UW187/2020, co	TTCACCGAGGCCACGCGGAGTACGATCGAGTGTACAGTGAA
MT252708.1 Severe acute respiratory syndrome coronavirus 2 isolate SARS-CoV-2/human/USA/WA-UW172/2020, co	TTCACCGAGGCCACGCGGAGTACGATCGAGTGTACAGTGAA
MT252709.1 Severe acute respiratory syndrome coronavirus 2 isolate SARS-CoV-2/human/USA/WA-UW174/2020, co	TTCACCGAGGCCACGCGGAGTACGATCGAGTGTACAGTGAA
MT252710.1 Severe acute respiratory syndrome coronavirus 2 isolate SARS-CoV-2/human/USA/WA-UW176/2020, co	TTCACCGAGGCCACGCGGAGTACGATCGAGTGTACAGTGAA
MT252711.1 Severe acute respiratory syndrome coronavirus 2 isolate SARS-CoV-2/human/USA/WA-UW181/2020 OR	TTCACCGAGGCCACGCGGAGTACGATCGAGTGTACAGTGAA
MT252712.1 Severe acute respiratory syndrome coronavirus 2 isolate SARS-CoV-2/human/USA/WA-UW169/2020, co	TTCACCGAGGCCACGCGGAGTACGATCGAGTGTACAGTGAA
MT252716.1 Severe acute respiratory syndrome coronavirus 2 isolate SARS-CoV-2/human/USA/WA-UW180/2020, co	TTCACCGAGGCCACGCGGAGTACGATCGAGTGTACAGTGAA
MT252717.1 Severe acute respiratory syndrome coronavirus 2 isolate SARS-CoV-2/human/USA/WA-UW185/2020, co	TTCACCGAGGCCACGCGGAGTACGATCGAGTGTACAGTGAA
MT252719.1 Severe acute respiratory syndrome coronavirus 2 isolate SARS-CoV-2/human/USA/WA-UW173/2020, co	TTCACCGAGGCCACGCGGAGTACGATCGAGTGTACAGTGAA
MT252721.1 Severe acute respiratory syndrome coronavirus 2 isolate SARS-CoV-2/human/USA/WA-UW163/2020, co	TTCACCGAGGCCACGCGGAGTACGATCGAGTGTACAGTGAA
MT252723.1 Severe acute respiratory syndrome coronavirus 2 isolate SARS-CoV-2/human/USA/WA-UW162/2020, co	TTCACCGAGGCCACGCGGAGTACGATCGAGTGTACAGTGAA
MT252726.1 Severe acute respiratory syndrome coronavirus 2 isolate SARS-CoV-2/human/USA/WA-UW155/2020, co	TTCACCGAGGCCACGCGGAGTACGATCGAGTGTACAGTGAA
MT252729.1 Severe acute respiratory syndrome coronavirus 2 isolate SARS-CoV-2/human/USA/WA-UW153/2020, co	TTCACCGAGGCCACGCGGAGTACGATCGAGTGTACAGTGAA
MT252734.1 Severe acute respiratory syndrome coronavirus 2 isolate SARS-CoV-2/human/USA/WA-UW154/2020 OR	TTCACCGAGGCCACGCGGAGTACGATCGAGTGTACAGTGAA
MT252735.1 Severe acute respiratory syndrome coronavirus 2 isolate SARS-CoV-2/human/USA/WA-UW149/2020, co	TTCACCGAGGCCACGCGGAGTACGATCGAGTGTACAGTGAA
MT252736.1 Severe acute respiratory syndrome coronavirus 2 isolate SARS-CoV-2/human/USA/WA-UW151/2020, co	TTCACCGAGGCCACGCGGAGTACGATCGAGTGTACAGTGAA

MT252737.1 Severe acute respiratory syndrome coronavirus 2 isolate SARS-CoV-2/human/USA/WA-UW146/2020, co	TTCACCGAGGCCACGCGGAGTACGATCGAGTGTACAGTGAA
MT252738.1 Severe acute respiratory syndrome coronavirus 2 isolate SARS-CoV-2/human/USA/WA-UW145/2020, co	TTCACCGAGGCCACGCGGAGTACGATCGAGTGTACAGTGAA
MT252740.1 Severe acute respiratory syndrome coronavirus 2 isolate SARS-CoV-2/human/USA/WA-UW150/2020, co	TTCACCGAGGCCACGCGGAGTACGATCGAGTGTACAGTGAA
MT252742.1 Severe acute respiratory syndrome coronavirus 2 isolate SARS-CoV-2/human/USA/WA-UW124/2020, co	TTCACCGAGGCCACGCGGAGTACGATCGAGTGTACAGTGAA
MT252745.1 Severe acute respiratory syndrome coronavirus 2 isolate SARS-CoV-2/human/USA/WA-UW127/2020, co	TTCACCGAGGCCACGCGGAGTACGATCGAGTGTACAGTGAA
MT252756.1 Severe acute respiratory syndrome coronavirus 2 isolate SARS-CoV-2/human/USA/WA-UW140/2020, co	TTCACCGAGGCCACGCGGAGTACGATCGAGTGTACAGTGAA
MT252757.1 Severe acute respiratory syndrome coronavirus 2 isolate SARS-CoV-2/human/USA/WA-UW141/2020, co	TTCACCGAGGCCACGCGGAGTACGATCGAGTGTACAGTGAA
MT252765.1 Severe acute respiratory syndrome coronavirus 2 isolate SARS-CoV-2/human/USA/WA-UW101/2020 OR	TTCACCGAGGCCACGCGGAGTACGATCGAGTGTACAGTGAA
MT252766.1 Severe acute respiratory syndrome coronavirus 2 isolate SARS-CoV-2/human/USA/WA-UW102/2020 OR	TTCACCGAGGCCACGCGGAGTACGATCGAGTGTACAGTGAA
MT252768.1 Severe acute respiratory syndrome coronavirus 2 isolate SARS-CoV-2/human/USA/WA-UW104/2020, co	TTCACCGAGGCCACGCGGAGTACGATCGAGTGTACAGTGAA
MT252769.1 Severe acute respiratory syndrome coronavirus 2 isolate SARS-CoV-2/human/USA/WA-UW105/2020, co	TTCACCGAGGCCACGCGGAGTACGATCGAGTGTACAGTGAA
MT252771.1 Severe acute respiratory syndrome coronavirus 2 isolate SARS-CoV-2/human/USA/WA-UW107/2020 OR	TTCACCGAGGCCACGCGGAGTACGATCGAGTGTACAGTGAA
MT252772.1 Severe acute respiratory syndrome coronavirus 2 isolate SARS-CoV-2/human/USA/WA-UW108/2020, co	TTCACCGAGGCCACGCGGAGTACGATCGAGTGTACAGTGAA
MT252773.1 Severe acute respiratory syndrome coronavirus 2 isolate SARS-CoV-2/human/USA/WA-UW109/2020, co	TTCACCGAGGCCACGCGGAGTACGATCGAGTGTACAGTGAA
MT252774.1 Severe acute respiratory syndrome coronavirus 2 isolate SARS-CoV-2/human/USA/WA-UW110/2020, co	TTCACCGAGGCCACGCGGAGTACGATCGAGTGTACAGTGAA
MT252778.1 Severe acute respiratory syndrome coronavirus 2 isolate SARS-CoV-2/human/USA/WA-UW114/2020, co	TTCACCGAGGCCACGCGGAGTACGATCGAGTGTACAGTGAA
MT252780.1 Severe acute respiratory syndrome coronavirus 2 isolate SARS-CoV-2/human/USA/WA-UW116/2020, co	TTCACCGAGGCCACGCGGAGTACGATCGAGTGTACAGTGAA
MT252782.1 Severe acute respiratory syndrome coronavirus 2 isolate SARS-CoV-2/human/USA/WA-UW118/2020, co	TTCACCGAGGCCACGCGGAGTACGATCGAGTGTACAGTGAA
MT252785.1 Severe acute respiratory syndrome coronavirus 2 isolate SARS-CoV-2/human/USA/WA-UW121/2020, co	TTCACCGAGGCCACGCGGAGTACGATCGAGTGTACAGTGAA
MT252787.1 Severe acute respiratory syndrome coronavirus 2 isolate SARS-CoV-2/human/USA/WA-UW64/2020, com	TTCACCGAGGCCACGCGGAGTACGATCGAGTGTACAGTGAA
MT252788.1 Severe acute respiratory syndrome coronavirus 2 isolate SARS-CoV-2/human/USA/WA-UW68/2020, com	TTCACCGAGGCCACGCGGAGTACGATCGAGTGTACAGTGAA
MT252795.1 Severe acute respiratory syndrome coronavirus 2 isolate SARS-CoV-2/human/USA/WA-UW74/2020, com	TTCACCGAGGCCACGCGGAGTACGATCGAGTGTACAGTGAA
MT252799.1 Severe acute respiratory syndrome coronavirus 2 isolate SARS-CoV-2/human/USA/WA-UW71/2020, com	TTCACCGAGGCCACGCGGAGTACGATCGAGTGTACAGTGAA
MT252801.1 Severe acute respiratory syndrome coronavirus 2 isolate SARS-CoV-2/human/USA/WA-UW40/2020, com	TTCACCGAGGCCACGCGGAGTACGATCGAGTGTACAGTGAA
MT252809.1 Severe acute respiratory syndrome coronavirus 2 isolate SARS-CoV-2/human/USA/WA-UW49/2020, com	TTCACCGAGGCCACGCGGAGTACGATCGAGTGTACAGTGAA
MT252811.1 Severe acute respiratory syndrome coronavirus 2 isolate SARS-CoV-2/human/USA/WA-UW51/2020, com	TTCACCGAGGCCACGCGGAGTACGATCGAGTGTACAGTGAA
MT252821.1 Severe acute respiratory syndrome coronavirus 2 isolate SARS-CoV-2/human/USA/WA-UW36/2020 ORF	TTCACCGAGGCCACGCGGAGTACGATCGAGTGTACAGTGAA
MT253696.1 Severe acute respiratory syndrome coronavirus 2 isolate SARS-CoV-2/human/CHN/HZ-162/2020, comple	TTCACCGAGGCCACGCGGAGTACGATCGAGTGTACAGTGAA
MT253697.1 Severe acute respiratory syndrome coronavirus 2 isolate SARS-CoV-2/human/CHN/HZ-178/2020, comple	TTCACCGAGGCCACGCGGAGTACGATCGAGTGTACAGTGAA
MT253698.1 Severe acute respiratory syndrome coronavirus 2 isolate SARS-CoV-2/human/CHN/HZ-185/2020, comple	TTCACCGAGGCCACGCGGAGTACGATCGAGTGTACAGTGAA
MT253699.1 Severe acute respiratory syndrome coronavirus 2 isolate SARS-CoV-2/human/CHN/HZ-477/2020, comple	TTCACCGAGGCCACGCGGAGTACGATCGAGTGTACAGTGAA
MT253700.1 Severe acute respiratory syndrome coronavirus 2 isolate SARS-CoV-2/human/CHN/HZ-481/2020, comple	TTCACCGAGGCCACGCGGAGTACGATCGAGTGTACAGTGAA
MT253701.1 Severe acute respiratory syndrome coronavirus 2 isolate SARS-CoV-2/human/CHN/HZ-48/2020, complet	TTCACCGAGGCCACGCGGAGTACGATCGAGTGTACAGTGAA

MT253702.1 Severe acute respiratory syndrome coronavirus 2 isolate SARS-CoV-2/human/CHN/HZ-49/2020, complet	TTCACCGAGGCCACGCGGAGTACGATCGAGTGTACAGTGAA
MT253703.1 Severe acute respiratory syndrome coronavirus 2 isolate SARS-CoV-2/human/CHN/HZ-551/2020, comple	TTCACCGAGGCCACGCGGAGTACGATCGAGTGTACAGTGAA
MT253704.1 Severe acute respiratory syndrome coronavirus 2 isolate SARS-CoV-2/human/CHN/HZ-576/2020, comple	TTCACCGAGGCCACGCGGAGTACGATCGAGTGTACAGTGAA
MT253705.1 Severe acute respiratory syndrome coronavirus 2 isolate SARS-CoV-2/human/CHN/HZ-60/2020, complet	TTCACCGAGGCCACGCGGAGTACGATCGAGTGTACAGTGAA
MT253706.1 Severe acute respiratory syndrome coronavirus 2 isolate SARS-CoV-2/human/CHN/HZ-62/2020, complet	TTCACCGAGGCCACGCGGAGTACGATCGAGTGTACAGTGAA
MT253707.1 Severe acute respiratory syndrome coronavirus 2 isolate SARS-CoV-2/human/CHN/HZ-638/2020, comple	TTCACCGAGGCCACGCGGAGTACGATCGAGTGTACAGTGAA
MT253708.1 Severe acute respiratory syndrome coronavirus 2 isolate SARS-CoV-2/human/CHN/HZ-79/2020, complet	TTCACCGAGGCCACGCGGAGTACGATCGAGTGTACAGTGAA
MT253709.1 Severe acute respiratory syndrome coronavirus 2 isolate SARS-CoV-2/human/CHN/HZ-90/2020, complet	TTCACCGAGGCCACGCGGAGTACGATCGAGTGTACAGTGAA
MT253710.1 Severe acute respiratory syndrome coronavirus 2 isolate SARS-CoV-2/human/CHN/HZ-91/2020, complet	TTCACCGAGGCCACGCGGAGTACGATCGAGTGTACAGTGAA
MT256917.1 Severe acute respiratory syndrome coronavirus 2 isolate SARS-CoV-2/human/ESP/Valencia9/2020, parti	TTCACCGAGGCCACGCGGAGTACGATCGAGTGTACAGTGAA
MT256918.1 Severe acute respiratory syndrome coronavirus 2 isolate SARS-CoV-2/human/ESP/Valencia10/2020, part	TTCACCGAGGCCACGCGGAGTACGATCGAGTGTACAGTGAA
MT256924.2 Severe acute respiratory syndrome coronavirus 2 isolate SARS-CoV-2/human/COL/79256_Antioquia/202	TTCACCGAGGCCACGCGGAGTACGATCGAGTGTACAGTGAA
MT258378.1 Severe acute respiratory syndrome coronavirus 2 isolate SARS-CoV-2/human/USA/CZB-RR057-006/2020	TTCACCGAGGCCACGCGGAGTACGATCGAGTGTACAGTGAA
MT258380.1 Severe acute respiratory syndrome coronavirus 2 isolate SARS-CoV-2/human/USA/CZB-RR057-011/2020	TTCACCGAGGCCACGCGGAGTACGATCGAGTGTACAGTGAA
MT259226.1 Severe acute respiratory syndrome coronavirus 2 isolate SARS-CoV-2/human/CHN/Wuhan_OS52/2020,	TTCACCGAGGCCACGCGGAGTACGATCGAGTGTACAGTGAA
MT259227.1 Severe acute respiratory syndrome coronavirus 2 isolate SARS-CoV-2/human/CHN/Wuhan_YB012611/2	TTCACCGAGGCCACGCGGAGTACGATCGAGTGTACAGTGAA
MT259228.1 Severe acute respiratory syndrome coronavirus 2 isolate SARS-CoV-2/human/CHN/Wuhan_YB012605/2	TTCACCGAGGCCACGCGGAGTACGATCGAGTGTACAGTGAA
MT259229.1 Severe acute respiratory syndrome coronavirus 2 isolate SARS-CoV-2/human/CHN/Wuhan_YB012602/2	TTCACCGAGGCCACGCGGAGTACGATCGAGTGTACAGTGAA
MT259230.1 Severe acute respiratory syndrome coronavirus 2 isolate SARS-CoV-2/human/CHN/Wuhan_YB012506/2	TTCACCGAGGCCACGCGGAGTACGATCGAGTGTACAGTGAA
MT259231.1 Severe acute respiratory syndrome coronavirus 2 isolate SARS-CoV-2/human/CHN/Wuhan_YB012504/2	TTCACCGAGGCCACGCGGAGTACGATCGAGTGTACAGTGAA
MT259235.1 Severe acute respiratory syndrome coronavirus 2 isolate SARS-CoV-2/human/USA/WA-UW243/2020, co	TTCACCGAGGCCACGCGGAGTACGATCGAGTGTACAGTGAA
MT259239.1 Severe acute respiratory syndrome coronavirus 2 isolate SARS-CoV-2/human/USA/WA-UW247/2020, co	TTCACCGAGGCCACGCGGAGTACGATCGAGTGTACAGTGAA
MT259240.1 Severe acute respiratory syndrome coronavirus 2 isolate SARS-CoV-2/human/USA/WA-UW248/2020, pa	TTCACCGAGGCCACGCGGAGTACGATCGAGTGTACAGTGAA
MT259241.1 Severe acute respiratory syndrome coronavirus 2 isolate SARS-CoV-2/human/USA/WA-UW249/2020, co	TTCACCGAGGCCACGCGGAGTACGATCGAGTGTACAGTGAA
MT259242.1 Severe acute respiratory syndrome coronavirus 2 isolate SARS-CoV-2/human/USA/CT-UW250/2020, par	TTCACCGAGGCCACGCGGAGTACGATCGAGTGTACAGTGAA
MT259243.1 Severe acute respiratory syndrome coronavirus 2 isolate SARS-CoV-2/human/USA/WA-UW251/2020, co	TTCACCGAGGCCACGCGGAGTACGATCGAGTGTACAGTGAA
MT259244.1 Severe acute respiratory syndrome coronavirus 2 isolate SARS-CoV-2/human/USA/WA-UW252/2020, co	TTCACCGAGGCCACGCGGAGTACGATCGAGTGTACAGTGAA
MT259246.1 Severe acute respiratory syndrome coronavirus 2 isolate SARS-CoV-2/human/USA/WA-UW254/2020, co	TTCACCGAGGCCACGCGGAGTACGATCGAGTGTACAGTGAA
MT259248.1 Severe acute respiratory syndrome coronavirus 2 isolate SARS-CoV-2/human/USA/CT-UW256/2020, com	TTCACCGAGGCCACGCGGAGTACGATCGAGTGTACAGTGAA
MT259249.1 Severe acute respiratory syndrome coronavirus 2 isolate SARS-CoV-2/human/USA/WA-UW257/2020, co	TTCACCGAGGCCACGCGGAGTACGATCGAGTGTACAGTGAA
MT259252.1 Severe acute respiratory syndrome coronavirus 2 isolate SARS-CoV-2/human/USA/WA-UW260/2020, co	TTCACCGAGGCCACGCGGAGTACGATCGAGTGTACAGTGAA
MT259253.1 Severe acute respiratory syndrome coronavirus 2 isolate SARS-CoV-2/human/USA/WA-UW261/2020, co	TTCACCGAGGCCACGCGGAGTACGATCGAGTGTACAGTGAA
MT259254.1 Severe acute respiratory syndrome coronavirus 2 isolate SARS-CoV-2/human/USA/WA-UW262/2020, co	TTCACCGAGGCCACGCGGAGTACGATCGAGTGTACAGTGAA

MT259256.1 Severe acute respiratory syndrome coronavirus 2 isolate SARS-CoV-2/human/USA/WA-UW264/2020, co	TTCACCGAGGCCACGCGGAGTACGATCGAGTGTACAGTGAA
MT259259.1 Severe acute respiratory syndrome coronavirus 2 isolate SARS-CoV-2/human/USA/WA-UW267/2020, pa	TTCACCGAGGCCACGCGGAGTACGATCGAGTGTACAGTGAA
MT259260.1 Severe acute respiratory syndrome coronavirus 2 isolate SARS-CoV-2/human/USA/WA-UW268/2020, co	TTCACCGAGGCCACGCGGAGTACGATCGAGTGTACAGTGAA
MT259261.1 Severe acute respiratory syndrome coronavirus 2 isolate SARS-CoV-2/human/USA/WA-UW269/2020, co	TTCACCGAGGCCACGCGGAGTACGATCGAGTGTACAGTGAA
MT259262.1 Severe acute respiratory syndrome coronavirus 2 isolate SARS-CoV-2/human/USA/WA-UW270/2020, pa	TTCACCGAGGCCACGCGGAGTACGATCGAGTGTACAGTGAA
MT259264.1 Severe acute respiratory syndrome coronavirus 2 isolate SARS-CoV-2/human/USA/WA-UW272/2020, co	TTCACCGAGGCCACGCGGAGTACGATCGAGTGTACAGTGAA
MT259265.1 Severe acute respiratory syndrome coronavirus 2 isolate SARS-CoV-2/human/USA/WA-UW273/2020, pa	TTCACCGAGGCCACGCGGAGTACGATCGAGTGTACAGTGAA
MT259266.1 Severe acute respiratory syndrome coronavirus 2 isolate SARS-CoV-2/human/USA/WA-UW274/2020, co	TTCACCGAGGCCACGCGGAGTACGATCGAGTGTACAGTGAA
MT259267.1 Severe acute respiratory syndrome coronavirus 2 isolate SARS-CoV-2/human/USA/WA-UW275/2020, co	TTCACCGAGGCCACGCGGAGTACGATCGAGTGTACAGTGAA
MT259269.1 Severe acute respiratory syndrome coronavirus 2 isolate SARS-CoV-2/human/USA/WA-UW277/2020, co	TTCACCGAGGCCACGCGGAGTACGATCGAGTGTACAGTGAA
MT259271.1 Severe acute respiratory syndrome coronavirus 2 isolate SARS-CoV-2/human/USA/WA-UW279/2020, co	TTCACCGAGGCCACGCGGAGTACGATCGAGTGTACAGTGAA
MT259272.1 Severe acute respiratory syndrome coronavirus 2 isolate SARS-CoV-2/human/USA/WA-UW280/2020, pa	TTCACCGAGGCCACGCGGAGTACGATCGAGTGTACAGTGAA
MT259275.1 Severe acute respiratory syndrome coronavirus 2 isolate SARS-CoV-2/human/USA/WA-UW285/2020, co	TTCACCGAGGCCACGCGGAGTACGATCGAGTGTACAGTGAA
MT259277.1 Severe acute respiratory syndrome coronavirus 2 isolate SARS-CoV-2/human/USA/WA-UW287/2020, co	TTCACCGAGGCCACGCGGAGTACGATCGAGTGTACAGTGAA
MT259278.1 Severe acute respiratory syndrome coronavirus 2 isolate SARS-CoV-2/human/USA/WA-UW288/2020, co	TTCACCGAGGCCACGCGGAGTACGATCGAGTGTACAGTGAA
MT259279.1 Severe acute respiratory syndrome coronavirus 2 isolate SARS-CoV-2/human/USA/WA-UW289/2020, pa	TTCACCGAGGCCACGCGGAGTACGATCGAGTGTACAGTGAA
MT259281.1 Severe acute respiratory syndrome coronavirus 2 isolate SARS-CoV-2/human/USA/WA-UW291/2020, co	TTCACCGAGGCCACGCGGAGTACGATCGAGTGTACAGTGAA
MT259282.1 Severe acute respiratory syndrome coronavirus 2 isolate SARS-CoV-2/human/USA/WA-UW292/2020, co	TTCACCGAGGCCACGCGGAGTACGATCGAGTGTACAGTGAA
MT259283.1 Severe acute respiratory syndrome coronavirus 2 isolate SARS-CoV-2/human/USA/WA-UW293/2020, pa	TTCACCGAGGCCACGCGGAGTACGATCGAGTGTACAGTGAA
MT259284.1 Severe acute respiratory syndrome coronavirus 2 isolate SARS-CoV-2/human/USA/WA-UW294/2020, co	TTCACCGAGGCCACGCGGAGTACGATCGAGTGTACAGTGAA
MT259286.1 Severe acute respiratory syndrome coronavirus 2 isolate SARS-CoV-2/human/USA/WA-UW296/2020, co	TTCACCGAGGCCACGCGGAGTACGATCGAGTGTACAGTGAA
MT259287.1 Severe acute respiratory syndrome coronavirus 2 isolate SARS-CoV-2/human/USA/WA-UW297/2020, co	TTCACCGAGGCCACGCGGAGTACGATCGAGTGTACAGTGAA
MT262896.1 Severe acute respiratory syndrome coronavirus 2 isolate SARS-CoV-2/human/USA/WA-NH2/2020, comp	TTCACCGAGGCCACGCGGAGTACGATCGAGTGTACAGTGAA
MT262897.1 Severe acute respiratory syndrome coronavirus 2 isolate SARS-CoV-2/human/USA/WA-NH3/2020, comp	TTCACCGAGGCCACGCGGAGTACGATCGAGTGTACAGTGAA
MT262898.1 Severe acute respiratory syndrome coronavirus 2 isolate SARS-CoV-2/human/USA/WA-NH4/2020, comp	TTCACCGAGGCCACGCGGAGTACGATCGAGTGTACAGTGAA
MT262899.1 Severe acute respiratory syndrome coronavirus 2 isolate SARS-CoV-2/human/USA/WA-NH5/2020, comp	TTCACCGAGGCCACGCGGAGTACGATCGAGTGTACAGTGAA
MT262900.1 Severe acute respiratory syndrome coronavirus 2 isolate SARS-CoV-2/human/USA/WA-NH6/2020, comp	TTCACCGAGGCCACGCGGAGTACGATCGAGTGTACAGTGAA
MT262901.1 Severe acute respiratory syndrome coronavirus 2 isolate SARS-CoV-2/human/USA/WA-NH7/2020, comp	TTCACCGAGGCCACGCGGAGTACGATCGAGTGTACAGTGAA
MT262902.1 Severe acute respiratory syndrome coronavirus 2 isolate SARS-CoV-2/human/USA/WA-NH8/2020, comp	TTCACCGAGGCCACGCGGAGTACGATCGAGTGTACAGTGAA
MT262903.1 Severe acute respiratory syndrome coronavirus 2 isolate SARS-CoV-2/human/USA/WA-NH9/2020, comp	TTCACCGAGGCCACGCGGAGTACGATCGAGTGTACAGTGAA
MT262904.1 Severe acute respiratory syndrome coronavirus 2 isolate SARS-CoV-2/human/USA/WA-NH10/2020, com	TTCACCGAGGCCACGCGGAGTACGATCGAGTGTACAGTGAA
MT262905.1 Severe acute respiratory syndrome coronavirus 2 isolate SARS-CoV-2/human/USA/WA-NH11/2020, com	TTCACCGAGGCCACGCGGAGTACGATCGAGTGTACAGTGAA
MT262906.1 Severe acute respiratory syndrome coronavirus 2 isolate SARS-CoV-2/human/USA/WA-NH12/2020, com	TTCACCGAGGCCACGCGGAGTACGATCGAGTGTACAGTGAA

MT262907.1 Severe acute respiratory syndrome coronavirus 2 isolate SARS-CoV-2/human/USA/WA-NH14/2020, com	TTCACCGAGGCCACGCGGAGTACGATCGAGTGTACAGTGAA
MT262908.1 Severe acute respiratory syndrome coronavirus 2 isolate SARS-CoV-2/human/USA/WA-NH17/2020, com	TTCACCGAGGCCACGCGGAGTACGATCGAGTGTACAGTGAA
MT262909.1 Severe acute respiratory syndrome coronavirus 2 isolate SARS-CoV-2/human/USA/WA-NH18/2020, com	TTCACCGAGGCCACGCGGAGTACGATCGAGTGTACAGTGAA
MT262910.1 Severe acute respiratory syndrome coronavirus 2 isolate SARS-CoV-2/human/USA/WA-NH19/2020, com	TTCACCGAGGCCACGCGGAGTACGATCGAGTGTACAGTGAA
MT262911.1 Severe acute respiratory syndrome coronavirus 2 isolate SARS-CoV-2/human/USA/WA-NH20/2020, com	TTCACCGAGGCCACGCGGAGTACGATCGAGTGTACAGTGAA
MT262912.1 Severe acute respiratory syndrome coronavirus 2 isolate SARS-CoV-2/human/USA/WA-NH21/2020, com	TTCACCGAGGCCACGCGGAGTACGATCGAGTGTACAGTGAA
MT262913.1 Severe acute respiratory syndrome coronavirus 2 isolate SARS-CoV-2/human/USA/WA-NH22/2020, com	TTCACCGAGGCCACGCGGAGTACGATCGAGTGTACAGTGAA
MT262914.1 Severe acute respiratory syndrome coronavirus 2 isolate SARS-CoV-2/human/USA/WA-NH24/2020, com	TTCACCGAGGCCACGCGGAGTACGATCGAGTGTACAGTGAA
MT262915.1 Severe acute respiratory syndrome coronavirus 2 isolate SARS-CoV-2/human/USA/WA-NH13/2020, com	TTCACCGAGGCCACGCGGAGTACGATCGAGTGTACAGTGAA
MT262916.1 Severe acute respiratory syndrome coronavirus 2 isolate SARS-CoV-2/human/USA/WA-NH23/2020, com	TTCACCGAGGCCACGCGGAGTACGATCGAGTGTACAGTGAA
MT262993.1 Severe acute respiratory syndrome coronavirus 2 isolate SARS-Cov-2/human/PAK/Manga1/2020, compl	TTCACCGAGGCCACGCGGAGTACGATCGAGTGTACAGTGAA
MT263074.1 Severe acute respiratory syndrome coronavirus 2 isolate SARS-CoV-2/human/PER/Peru-10/2020, compl	TTCACCGAGGCCACGCGGAGTACGATCGAGTGTACAGTGAA
MT263383.1 Severe acute respiratory syndrome coronavirus 2 isolate SARS-CoV-2/human/USA/WA-UW300/2020, co	TTCACCGAGGCCACGCGGAGTACGATCGAGTGTACAGTGAA
MT263388.1 Severe acute respiratory syndrome coronavirus 2 isolate SARS-CoV-2/human/USA/WA-UW305/2020, co	TTCACCGAGGCCACGCGGAGTACGATCGAGTGTACAGTGAA
MT263392.1 Severe acute respiratory syndrome coronavirus 2 isolate SARS-CoV-2/human/USA/WA-UW310/2020, co	TTCACCGAGGCCACGCGGAGTACGATCGAGTGTACAGTGAA
MT263394.1 Severe acute respiratory syndrome coronavirus 2 isolate SARS-CoV-2/human/USA/WA-UW313/2020, co	TTCACCGAGGCCACGCGGAGTACGATCGAGTGTACAGTGAA
MT263396.1 Severe acute respiratory syndrome coronavirus 2 isolate SARS-CoV-2/human/USA/WA-UW315/2020, co	TTCACCGAGGCCACGCGGAGTACGATCGAGTGTACAGTGAA
MT263399.1 Severe acute respiratory syndrome coronavirus 2 isolate SARS-CoV-2/human/USA/WA-UW319/2020, co	TTCACCGAGGCCACGCGGAGTACGATCGAGTGTACAGTGAA
MT263401.1 Severe acute respiratory syndrome coronavirus 2 isolate SARS-CoV-2/human/USA/WA-UW321/2020, pa	TTCACCGAGGCCACGCGGAGTACGATCGAGTGTACAGTGAA
MT263402.1 Severe acute respiratory syndrome coronavirus 2 isolate SARS-CoV-2/human/USA/WA-UW322/2020, co	TTCACCGAGGCCACGCGGAGTACGATCGAGTGTACAGTGAA
MT263404.1 Severe acute respiratory syndrome coronavirus 2 isolate SARS-CoV-2/human/USA/WA-UW324/2020, co	TTCACCGAGGCCACGCGGAGTACGATCGAGTGTACAGTGAA
MT263406.1 Severe acute respiratory syndrome coronavirus 2 isolate SARS-CoV-2/human/USA/MN-UW326/2020, co	TTCACCGAGGCCACGCGGAGTACGATCGAGTGTACAGTGAA
MT263407.1 Severe acute respiratory syndrome coronavirus 2 isolate SARS-CoV-2/human/USA/CT-UW327/2020, par	TTCACCGAGGCCACGCGGAGTACGATCGAGTGTACAGTGAA
MT263408.1 Severe acute respiratory syndrome coronavirus 2 isolate SARS-CoV-2/human/USA/WA-UW328/2020, co	TTCACCGAGGCCACGCGGAGTACGATCGAGTGTACAGTGAA
MT263409.1 Severe acute respiratory syndrome coronavirus 2 isolate SARS-CoV-2/human/USA/WA-UW379/2020, pa	TTCACCGAGGCCACGCGGAGTACGATCGAGTGTACAGTGAA
MT263410.1 Severe acute respiratory syndrome coronavirus 2 isolate SARS-CoV-2/human/USA/WA-UW330/2020, co	TTCACCGAGGCCACGCGGAGTACGATCGAGTGTACAGTGAA
MT263411.1 Severe acute respiratory syndrome coronavirus 2 isolate SARS-CoV-2/human/USA/CT-UW331/2020, com	TTCACCGAGGCCACGCGGAGTACGATCGAGTGTACAGTGAA
MT263414.1 Severe acute respiratory syndrome coronavirus 2 isolate SARS-CoV-2/human/USA/WA-UW334/2020, co	TTCACCGAGGCCACGCGGAGTACGATCGAGTGTACAGTGAA
MT263415.1 Severe acute respiratory syndrome coronavirus 2 isolate SARS-CoV-2/human/USA/MN-UW335/2020, co	TTCACCGAGGCCACGCGGAGTACGATCGAGTGTACAGTGAA
MT263416.1 Severe acute respiratory syndrome coronavirus 2 isolate SARS-CoV-2/human/USA/WA-UW336/2020, co	TTCACCGAGGCCACGCGGAGTACGATCGAGTGTACAGTGAA
MT263418.1 Severe acute respiratory syndrome coronavirus 2 isolate SARS-CoV-2/human/USA/WA-UW338/2020, co	TTCACCGAGGCCACGCGGAGTACGATCGAGTGTACAGTGAA
MT263419.1 Severe acute respiratory syndrome coronavirus 2 isolate SARS-CoV-2/human/USA/WA-UW339/2020, co	TTCACCGAGGCCACGCGGAGTACGATCGAGTGTACAGTGAA
MT263420.1 Severe acute respiratory syndrome coronavirus 2 isolate SARS-CoV-2/human/USA/WA-UW340/2020, co	TTCACCGAGGCCACGCGGAGTACGATCGAGTGTACAGTGAA

MT263421.1 Severe acute respiratory syndrome coronavirus 2 isolate SARS-CoV-2/human/USA/WA-UW341/2020, co	TTCACCGAGGCCACGCGGAGTACGATCGAGTGTACAGTGAA
MT263424.1 Severe acute respiratory syndrome coronavirus 2 isolate SARS-CoV-2/human/USA/WA-UW344/2020, co	TTCACCGAGGCCACGCGGAGTACGATCGAGTGTACAGTGAA
MT263425.1 Severe acute respiratory syndrome coronavirus 2 isolate SARS-CoV-2/human/USA/WA-UW345/2020, co	TTCACCGAGGCCACGCGGAGTACGATCGAGTGTACAGTGAA
MT263426.1 Severe acute respiratory syndrome coronavirus 2 isolate SARS-CoV-2/human/USA/WA-UW346/2020, co	TTCACCGAGGCCACGCGGAGTACGATCGAGTGTACAGTGAA
MT263429.1 Severe acute respiratory syndrome coronavirus 2 isolate SARS-CoV-2/human/USA/IL-UW349/2020, com	TTCACCGAGGCCACGCGGAGTACGATCGAGTGTACAGTGAA
MT263431.1 Severe acute respiratory syndrome coronavirus 2 isolate SARS-CoV-2/human/USA/WA-UW351/2020, co	TTCACCGAGGCCACGCGGAGTACGATCGAGTGTACAGTGAA
MT263434.1 Severe acute respiratory syndrome coronavirus 2 isolate SARS-CoV-2/human/USA/WA-UW354/2020, co	TTCACCGAGGCCACGCGGAGTACGATCGAGTGTACAGTGAA
MT263435.1 Severe acute respiratory syndrome coronavirus 2 isolate SARS-CoV-2/human/USA/WA-UW355/2020, co	TTCACCGAGGCCACGCGGAGTACGATCGAGTGTACAGTGAA
MT263436.1 Severe acute respiratory syndrome coronavirus 2 isolate SARS-CoV-2/human/USA/WA-UW356/2020, co	TTCACCGAGGCCACGCGGAGTACGATCGAGTGTACAGTGAA
MT263437.1 Severe acute respiratory syndrome coronavirus 2 isolate SARS-CoV-2/human/USA/WA-UW357/2020, co	TTCACCGAGGCCACGCGGAGTACGATCGAGTGTACAGTGAA
MT263438.1 Severe acute respiratory syndrome coronavirus 2 isolate SARS-CoV-2/human/USA/UNKNOWN-UW358/2	TTCACCGAGGCCACGCGGAGTACGATCGAGTGTACAGTGAA
MT263440.1 Severe acute respiratory syndrome coronavirus 2 isolate SARS-CoV-2/human/USA/WA-UW360/2020, co	TTCACCGAGGCCACGCGGAGTACGATCGAGTGTACAGTGAA
MT263443.1 Severe acute respiratory syndrome coronavirus 2 isolate SARS-CoV-2/human/USA/WA-UW363/2020, co	TTCACCGAGGCCACGCGGAGTACGATCGAGTGTACAGTGAA
MT263445.1 Severe acute respiratory syndrome coronavirus 2 isolate SARS-CoV-2/human/USA/WA-UW365/2020, co	TTCACCGAGGCCACGCGGAGTACGATCGAGTGTACAGTGAA
MT263446.1 Severe acute respiratory syndrome coronavirus 2 isolate SARS-CoV-2/human/USA/WA-UW366/2020, co	TTCACCGAGGCCACGCGGAGTACGATCGAGTGTACAGTGAA
MT263447.1 Severe acute respiratory syndrome coronavirus 2 isolate SARS-CoV-2/human/USA/WA-UW367/2020, co	TTCACCGAGGCCACGCGGAGTACGATCGAGTGTACAGTGAA
MT263450.1 Severe acute respiratory syndrome coronavirus 2 isolate SARS-CoV-2/human/USA/WA-UW370/2020, co	TTCACCGAGGCCACGCGGAGTACGATCGAGTGTACAGTGAA
MT263451.1 Severe acute respiratory syndrome coronavirus 2 isolate SARS-CoV-2/human/USA/WA-UW371/2020, co	TTCACCGAGGCCACGCGGAGTACGATCGAGTGTACAGTGAA
MT263452.1 Severe acute respiratory syndrome coronavirus 2 isolate SARS-CoV-2/human/USA/WA-UW372/2020, co	TTCACCGAGGCCACGCGGAGTACGATCGAGTGTACAGTGAA
MT263454.1 Severe acute respiratory syndrome coronavirus 2 isolate SARS-CoV-2/human/USA/WA-UW374/2020, co	TTCACCGAGGCCACGCGGAGTACGATCGAGTGTACAGTGAA
MT263455.1 Severe acute respiratory syndrome coronavirus 2 isolate SARS-CoV-2/human/USA/WA-UW375/2020, co	TTCACCGAGGCCACGCGGAGTACGATCGAGTGTACAGTGAA
MT263457.1 Severe acute respiratory syndrome coronavirus 2 isolate SARS-CoV-2/human/USA/WA-UW390/2020, co	TTCACCGAGGCCACGCGGAGTACGATCGAGTGTACAGTGAA
MT263458.1 Severe acute respiratory syndrome coronavirus 2 isolate SARS-CoV-2/human/USA/WA-UW378/2020, co	TTCACCGAGGCCACGCGGAGTACGATCGAGTGTACAGTGAA
MT263462.1 Severe acute respiratory syndrome coronavirus 2 isolate SARS-CoV-2/human/USA/WA-UW383/2020, co	TTCACCGAGGCCACGCGGAGTACGATCGAGTGTACAGTGAA
MT263463.1 Severe acute respiratory syndrome coronavirus 2 isolate SARS-CoV-2/human/USA/CT-UW329/2020, con	TTCACCGAGGCCACGCGGAGTACGATCGAGTGTACAGTGAA
MT263464.1 Severe acute respiratory syndrome coronavirus 2 isolate SARS-CoV-2/human/USA/WA-UW384/2020, co	TTCACCGAGGCCACGCGGAGTACGATCGAGTGTACAGTGAA
MT263466.1 Severe acute respiratory syndrome coronavirus 2 isolate SARS-CoV-2/human/USA/WA-UW386/2020, pa	TTCACCGAGGCCACGCGGAGTACGATCGAGTGTACAGTGAA
MT263468.1 Severe acute respiratory syndrome coronavirus 2 isolate SARS-CoV-2/human/USA/WA-UW388/2020, co	TTCACCGAGGCCACGCGGAGTACGATCGAGTGTACAGTGAA
MT263469.1 Severe acute respiratory syndrome coronavirus 2 isolate SARS-CoV-2/human/USA/WA-UW389/2020, co	TTCACCGAGGCCACGCGGAGTACGATCGAGTGTACAGTGAA
MT270101.1 Severe acute respiratory syndrome coronavirus 2 isolate SARS-CoV-2/human/DEU/BavPat1-ChVir929/20	TTCACCGAGGCCACGCGGAGTACGATCGAGTGTACAGTGAA
MT270102.1 Severe acute respiratory syndrome coronavirus 2 isolate SARS-CoV-2/human/DEU/BavPat4-ChVir1019/2	TTCACCGAGGCCACGCGGAGTACGATCGAGTGTACAGTGAA
MT270103.1 Severe acute respiratory syndrome coronavirus 2 isolate SARS-CoV-2/human/DEU/BavPat3-ChVir1020/2	TTCACCGAGGCCACGCGGAGTACGATCGAGTGTACAGTGAA
MT270104.1 Severe acute respiratory syndrome coronavirus 2 isolate SARS-CoV-2/human/DEU/BavPat6-ChVir1247/2	TTCACCGAGGCCACGCGGAGTACGATCGAGTGTACAGTGAA

MT270105.1 Severe acute respiratory syndrome coronavirus 2 isolate SARS-CoV-2/human/DEU/BavPat5-ChVir1248/2	TTCACCGAGGCCACGCGGAGTACGATCGAGTGTACAGTGAA
MT270106.1 Severe acute respiratory syndrome coronavirus 2 isolate SARS-CoV-2/human/DEU/BavPat7-ChVir1289/2	TTCACCGAGGCCACGCGGAGTACGATCGAGTGTACAGTGAA
MT270107.1 Severe acute respiratory syndrome coronavirus 2 isolate SARS-CoV-2/human/DEU/BavPat8-ChVir1356/2	TTCACCGAGGCCACGCGGAGTACGATCGAGTGTACAGTGAA
MT270108.1 Severe acute respiratory syndrome coronavirus 2 isolate SARS-CoV-2/human/DEU/BavPat9-ChVir1433/2	TTCACCGAGGCCACGCGGAGTACGATCGAGTGTACAGTGAA
MT270109.1 Severe acute respiratory syndrome coronavirus 2 isolate SARS-CoV-2/human/DEU/BavPat11-ChVir1459/	TTCACCGAGGCCACGCGGAGTACGATCGAGTGTACAGTGAA
MT270110.1 Severe acute respiratory syndrome coronavirus 2 isolate SARS-CoV-2/human/DEU/BavPat10-ChVir1482/	TTCACCGAGGCCACGCGGAGTACGATCGAGTGTACAGTGAA
MT270111.1 Severe acute respiratory syndrome coronavirus 2 isolate SARS-CoV-2/human/DEU/BavPat14-ChVir1483/	TTCACCGAGGCCACGCGGAGTACGATCGAGTGTACAGTGAA
MT270112.1 Severe acute respiratory syndrome coronavirus 2 isolate SARS-CoV-2/human/DEU/BavPat2-ChVir984-Ch	TTCACCGAGGCCACGCGGAGTACGATCGAGTGTACAGTGAA
MT270113.1 Severe acute respiratory syndrome coronavirus 2 isolate SARS-CoV-2/human/DEU/BavPat16-ChVir1530/	TTCACCGAGGCCACGCGGAGTACGATCGAGTGTACAGTGAA
MT270114.1 Severe acute respiratory syndrome coronavirus 2 isolate SARS-CoV-2/human/DEU/BavPat15-ChVir1484-	TTCACCGAGGCCACGCGGAGTACGATCGAGTGTACAGTGAA
MT270814.1 Severe acute respiratory syndrome coronavirus 2 isolate SARS-CoV-2/canine/HKG/20-03695/2020, comp	TTCACCGAGGCCACGCGGAGTACGATCGAGTGTACAGTGAA
MT270815.1 Severe acute respiratory syndrome coronavirus 2 isolate SARS-CoV-2/human/HKG/case163_VM2000957	TTCACCGAGGCCACGCGGAGTACGATCGAGTGTACAGTGAA
MT276323.1 Severe acute respiratory syndrome coronavirus 2 isolate SARS-CoV-2/human/USA/RI_0520/2020, compl	TTCACCGAGGCCACGCGGAGTACGATCGAGTGTACAGTGAA
MT276324.1 Severe acute respiratory syndrome coronavirus 2 isolate SARS-CoV-2/human/USA/CA_2602/2020, comp	TTCACCGAGGCCACGCGGAGTACGATCGAGTGTACAGTGAA
MT276325.2 Severe acute respiratory syndrome coronavirus 2 isolate SARS-CoV-2/human/USA/WA_5030/2020, com	TTCACCGAGGCCACGCGGAGTACGATCGAGTGTACAGTGAA
MT276326.2 Severe acute respiratory syndrome coronavirus 2 isolate SARS-CoV-2/human/USA/GA_2741/2020, comp	TTCACCGAGGCCACGCGGAGTACGATCGAGTGTACAGTGAA
MT276327.1 Severe acute respiratory syndrome coronavirus 2 isolate SARS-CoV-2/human/USA/GA_2742/2020, comp	TTCACCGAGGCCACGCGGAGTACGATCGAGTGTACAGTGAA
MT276328.2 Severe acute respiratory syndrome coronavirus 2 isolate SARS-CoV-2/human/USA/OR_2656/2020, comp	TTCACCGAGGCCACGCGGAGTACGATCGAGTGTACAGTGAA
MT276329.1 Severe acute respiratory syndrome coronavirus 2 isolate SARS-CoV-2/human/USA/FL_5125/2020, comp	TTCACCGAGGCCACGCGGAGTACGATCGAGTGTACAGTGAA
MT276330.2 Severe acute respiratory syndrome coronavirus 2 isolate SARS-CoV-2/human/USA/FL_5091/2020, comp	TTCACCGAGGCCACGCGGAGTACGATCGAGTGTACAGTGAA
MT276597.1 Severe acute respiratory syndrome coronavirus 2 isolate SARS-CoV-2/human/ISR/ISR_JP0320/2020, com	TTCACCGAGGCCACGCGGAGTACGATCGAGTGTACAGTGAA
MT276598.1 Severe acute respiratory syndrome coronavirus 2 isolate SARS-CoV-2/human/ISR/ISR_IT0320/2020, com	TTCACCGAGGCCACGCGGAGTACGATCGAGTGTACAGTGAA
MT276600.1 Severe acute respiratory syndrome coronavirus 2 isolate SARS-CoV-2/human/HKG/case113_VM2003179	TTCACCGAGGCCACGCGGAGTACGATCGAGTGTACAGTGAA
MT281577.1 Severe acute respiratory syndrome coronavirus 2 isolate SARS-CoV-2/human/CHN/Fuyang_FY002/2020,	TTCACCGAGGCCACGCGGAGTACGATCGAGTGTACAGTGAA
MT291826.1 Severe acute respiratory syndrome coronavirus 2 isolate SARS-CoV-2/human/CHN/Wuhan_IME-WH01/2	TTCACCGAGGCCACGCGGAGTACGATCGAGTGTACAGTGAA
MT291827.1 Severe acute respiratory syndrome coronavirus 2 isolate SARS-CoV-2/human/CHN/Wuhan_IME-WH02/2	TTCACCGAGGCCACGCGGAGTACGATCGAGTGTACAGTGAA
MT291828.1 Severe acute respiratory syndrome coronavirus 2 isolate SARS-CoV-2/human/CHN/Wuhan_IME-WH03/2	TTCACCGAGGCCACGCGGAGTACGATCGAGTGTACAGTGAA
MT291829.1 Severe acute respiratory syndrome coronavirus 2 isolate SARS-CoV-2/human/CHN/Wuhan_IME-WH04/2	TTCACCGAGGCCACGCGGAGTACGATCGAGTGTACAGTGAA
MT291830.1 Severe acute respiratory syndrome coronavirus 2 isolate SARS-CoV-2/human/CHN/Wuhan_IME-WH05/2	TTCACCGAGGCCACGCGGAGTACGATCGAGTGTACAGTGAA
MT291831.1 Severe acute respiratory syndrome coronavirus 2 isolate SARS-CoV-2/human/CHN/Beijing_IME-BJ01/20	TTCACCGAGGCCACGCGGAGTACGATCGAGTGTACAGTGAA
MT291832.1 Severe acute respiratory syndrome coronavirus 2 isolate SARS-CoV-2/human/CHN/Beijing_IME-BJ02/20	TTCACCGAGGCCACGCGGAGTACGATCGAGTGTACAGTGAA
MT291833.1 Severe acute respiratory syndrome coronavirus 2 isolate SARS-CoV-2/human/CHN/Beijing_IME-BJ03/20	TTCACCGAGGCCACGCGGAGTACGATCGAGTGTACAGTGAA
MT291834.1 Severe acute respiratory syndrome coronavirus 2 isolate SARS-CoV-2/human/CHN/Beijing_IME-BJ04/20	TTCACCGAGGCCACGCGGAGTACGATCGAGTGTACAGTGAA

MT291835.2 Severe acute respiratory syndrome coronavirus 2 isolate SARS-CoV-2/human/CHN/Beijing_IME-BJ05/20	TTCACCGAGGCCACGCGGAGTACGATCGAGTGTACAGTGAA
MT291836.1 Severe acute respiratory syndrome coronavirus 2 isolate SARS-CoV-2/human/CHN/Beijing_IME-BJ07/20	TTCACCGAGGCCACGCGGAGTACGATCGAGTGTACAGTGAA
MT292569.1 Severe acute respiratory syndrome coronavirus 2 isolate SARS-CoV-2/human/ESP/Valencia13/2020, com	TTCACCGAGGCCACGCGGAGTACGATCGAGTGTACAGTGAA
MT292570.1 Severe acute respiratory syndrome coronavirus 2 isolate SARS-CoV-2/human/ESP/Valencia17/2020, com	TTCACCGAGGCCACGCGGAGTACGATCGAGTGTACAGTGAA
MT292571.1 Severe acute respiratory syndrome coronavirus 2 isolate SARS-CoV-2/human/ESP/Valencia12/2020, com	TTCACCGAGGCCACGCGGAGTACGATCGAGTGTACAGTGAA
MT292572.1 Severe acute respiratory syndrome coronavirus 2 isolate SARS-CoV-2/human/ESP/Valencia11/2020, com	TTCACCGAGGCCACGCGGAGTACGATCGAGTGTACAGTGAA
MT292573.1 Severe acute respiratory syndrome coronavirus 2 isolate SARS-CoV-2/human/ESP/Valencia14/2020, com	TTCACCGAGGCCACGCGGAGTACGATCGAGTGTACAGTGAA
MT292574.1 Severe acute respiratory syndrome coronavirus 2 isolate SARS-CoV-2/human/ESP/Valencia15/2020, com	TTCACCGAGGCCACGCGGAGTACGATCGAGTGTACAGTGAA
MT292575.1 Severe acute respiratory syndrome coronavirus 2 isolate SARS-CoV-2/human/ESP/Valencia16/2020, com	TTCACCGAGGCCACGCGGAGTACGATCGAGTGTACAGTGAA
MT292576.1 Severe acute respiratory syndrome coronavirus 2 isolate SARS-CoV-2/human/ESP/Valencia18/2020 ORF	TTCACCGAGGCCACGCGGAGTACGATCGAGTGTACAGTGAA
MT292577.1 Severe acute respiratory syndrome coronavirus 2 isolate SARS-CoV-2/human/ESP/Valencia19/2020 ORF	TTCACCGAGGCCACGCGGAGTACGATCGAGTGTACAGTGAA
MT292578.1 Severe acute respiratory syndrome coronavirus 2 isolate SARS-CoV-2/human/ESP/Valencia20/2020 ORF	TTCACCGAGGCCACGCGGAGTACGATCGAGTGTACAGTGAA
MT292580.1 Severe acute respiratory syndrome coronavirus 2 isolate SARS-CoV-2/human/ESP/Valencia25/2020 ORF	TTCACCGAGGCCACGCGGAGTACGATCGAGTGTACAGTGAA
MT293156.1 Severe acute respiratory syndrome coronavirus 2 isolate SARS-CoV-2/human/USAWA-UW391/2020, cor	TTCACCGAGGCCACGCGGAGTACGATCGAGTGTACAGTGAA
MT293158.1 Severe acute respiratory syndrome coronavirus 2 isolate SARS-CoV-2/human/USA/WA-UW-1271/2020,	TTCACCGAGGCCACGCGGAGTACGATCGAGTGTACAGTGAA
MT293159.1 Severe acute respiratory syndrome coronavirus 2 isolate SARS-CoV-2/human/USA/WA-UW-1304/2020,	TTCACCGAGGCCACGCGGAGTACGATCGAGTGTACAGTGAA
MT293160.1 Severe acute respiratory syndrome coronavirus 2 isolate SARS-CoV-2/human/USA/WA-UW-1315/2020,	TTCACCGAGGCCACGCGGAGTACGATCGAGTGTACAGTGAA
MT293162.1 Severe acute respiratory syndrome coronavirus 2 isolate SARS-CoV-2/human/USA/WA-UW-1302/2020,	TTCACCGAGGCCACGCGGAGTACGATCGAGTGTACAGTGAA
MT293163.1 Severe acute respiratory syndrome coronavirus 2 isolate SARS-CoV-2/human/USAUNKNOWN-UW398/2	TTCACCGAGGCCACGCGGAGTACGATCGAGTGTACAGTGAA
MT293164.1 Severe acute respiratory syndrome coronavirus 2 isolate SARS-CoV-2/human/USA/WA-UW-1287/2020,	TTCACCGAGGCCACGCGGAGTACGATCGAGTGTACAGTGAA
MT293165.1 Severe acute respiratory syndrome coronavirus 2 isolate SARS-CoV-2/human/USA/WA-UW-1269/2020,	TTCACCGAGGCCACGCGGAGTACGATCGAGTGTACAGTGAA
MT293167.1 Severe acute respiratory syndrome coronavirus 2 isolate SARS-CoV-2/human/USA/WA-UW-1319/2020,	TTCACCGAGGCCACGCGGAGTACGATCGAGTGTACAGTGAA
MT293169.1 Severe acute respiratory syndrome coronavirus 2 isolate SARS-CoV-2/human/USA/WA-UW-1279/2020,	TTCACCGAGGCCACGCGGAGTACGATCGAGTGTACAGTGAA
MT293171.1 Severe acute respiratory syndrome coronavirus 2 isolate SARS-CoV-2/human/USA/WA-UW-1306/2020,	TTCACCGAGGCCACGCGGAGTACGATCGAGTGTACAGTGAA
MT293172.1 Severe acute respiratory syndrome coronavirus 2 isolate SARS-CoV-2/human/USA/WA-UW-1309/2020,	TTCACCGAGGCCACGCGGAGTACGATCGAGTGTACAGTGAA
MT293175.1 Severe acute respiratory syndrome coronavirus 2 isolate SARS-CoV-2/human/USAWA-UW410/2020, cor	TTCACCGAGGCCACGCGGAGTACGATCGAGTGTACAGTGAA
MT293176.1 Severe acute respiratory syndrome coronavirus 2 isolate SARS-CoV-2/human/USA/WA-UW-1417/2020,	TTCACCGAGGCCACGCGGAGTACGATCGAGTGTACAGTGAA
MT293178.1 Severe acute respiratory syndrome coronavirus 2 isolate SARS-CoV-2/human/USAWA-UW413/2020, cor	TTCACCGAGGCCACGCGGAGTACGATCGAGTGTACAGTGAA
MT293180.1 Severe acute respiratory syndrome coronavirus 2 isolate SARS-CoV-2/human/USA/WA-UW-1268/2020,	TTCACCGAGGCCACGCGGAGTACGATCGAGTGTACAGTGAA
MT293181.1 Severe acute respiratory syndrome coronavirus 2 isolate SARS-CoV-2/human/USA/WA-UW-1338/2020,	TTCACCGAGGCCACGCGGAGTACGATCGAGTGTACAGTGAA
MT293183.1 Severe acute respiratory syndrome coronavirus 2 isolate SARS-CoV-2/human/USA/WA-UW-1296/2020,	TTCACCGAGGCCACGCGGAGTACGATCGAGTGTACAGTGAA
MT293186.1 Severe acute respiratory syndrome coronavirus 2 isolate SARS-CoV-2/human/USA/WA-UW-1314/2020,	TTCACCGAGGCCACGCGGAGTACGATCGAGTGTACAGTGAA
MT293188.1 Severe acute respiratory syndrome coronavirus 2 isolate SARS-CoV-2/human/USACT-UW423/2020, com	TTCACCGAGGCCACGCGGAGTACGATCGAGTGTACAGTGAA

MT293189.1 Severe acute respiratory syndrome coronavirus 2 isolate SARS-CoV-2/human/USA/WA-UW-1322/2020,	TTCACCGAGGCCACGCGGAGTACGATCGAGTGTACAGTGAA
MT293190.1 Severe acute respiratory syndrome coronavirus 2 isolate SARS-CoV-2/human/USA/WA-UW-1334/2020,	TTCACCGAGGCCACGCGGAGTACGATCGAGTGTACAGTGAA
MT293191.1 Severe acute respiratory syndrome coronavirus 2 isolate SARS-CoV-2/human/USA/WA-UW-1346/2020,	TTCACCGAGGCCACGCGGAGTACGATCGAGTGTACAGTGAA
MT293193.1 Severe acute respiratory syndrome coronavirus 2 isolate SARS-CoV-2/human/USACT-UW428/2020 ORF1	TTCACCGAGGCCACGCGGAGTACGATCGAGTGTACAGTGAA
MT293195.1 Severe acute respiratory syndrome coronavirus 2 isolate SARS-CoV-2/human/USACT-UW430/2020, com	TTCACCGAGGCCACGCGGAGTACGATCGAGTGTACAGTGAA
MT293196.1 Severe acute respiratory syndrome coronavirus 2 isolate SARS-CoV-2/human/USA/WA-UW-1567/2020,	TTCACCGAGGCCACGCGGAGTACGATCGAGTGTACAGTGAA
MT293197.1 Severe acute respiratory syndrome coronavirus 2 isolate SARS-CoV-2/human/USA/WA-UW-1593/2020,	TTCACCGAGGCCACGCGGAGTACGATCGAGTGTACAGTGAA
MT293198.1 Severe acute respiratory syndrome coronavirus 2 isolate SARS-CoV-2/human/USA/WA-UW-1583/2020,	TTCACCGAGGCCACGCGGAGTACGATCGAGTGTACAGTGAA
MT293199.1 Severe acute respiratory syndrome coronavirus 2 isolate SARS-CoV-2/human/USA/WA-UW-1581/2020,	TTCACCGAGGCCACGCGGAGTACGATCGAGTGTACAGTGAA
MT293200.1 Severe acute respiratory syndrome coronavirus 2 isolate SARS-CoV-2/human/USA/WA-UW-1603/2020,	TTCACCGAGGCCACGCGGAGTACGATCGAGTGTACAGTGAA
MT293201.1 Severe acute respiratory syndrome coronavirus 2 isolate SARS-CoV-2/human/USA/WA-UW-1611/2020,	TTCACCGAGGCCACGCGGAGTACGATCGAGTGTACAGTGAA
MT293202.1 Severe acute respiratory syndrome coronavirus 2 isolate SARS-CoV-2/human/USA/WA-UW-1448/2020,	TTCACCGAGGCCACGCGGAGTACGATCGAGTGTACAGTGAA
MT293203.1 Severe acute respiratory syndrome coronavirus 2 isolate SARS-CoV-2/human/USA/WA-UW-1426/2020 O	TTCACCGAGGCCACGCGGAGTACGATCGAGTGTACAGTGAA
MT293204.1 Severe acute respiratory syndrome coronavirus 2 isolate SARS-CoV-2/human/USA/WA-UW-1446/2020,	TTCACCGAGGCCACGCGGAGTACGATCGAGTGTACAGTGAA
MT293205.1 Severe acute respiratory syndrome coronavirus 2 isolate SARS-CoV-2/human/USA/WA-UW-1561/2020,	TTCACCGAGGCCACGCGGAGTACGATCGAGTGTACAGTGAA
MT293206.1 Severe acute respiratory syndrome coronavirus 2 isolate SARS-CoV-2/human/USA/WA-UW-1588/2020,	TTCACCGAGGCCACGCGGAGTACGATCGAGTGTACAGTGAA
MT293207.1 Severe acute respiratory syndrome coronavirus 2 isolate SARS-CoV-2/human/USA/WA-UW-1586/2020,	TTCACCGAGGCCACGCGGAGTACGATCGAGTGTACAGTGAA
MT293209.1 Severe acute respiratory syndrome coronavirus 2 isolate SARS-CoV-2/human/USA/WA-UW-1552/2020,	TTCACCGAGGCCACGCGGAGTACGATCGAGTGTACAGTGAA
MT293210.1 Severe acute respiratory syndrome coronavirus 2 isolate SARS-CoV-2/human/USA/WA-UW-1578/2020,	TTCACCGAGGCCACGCGGAGTACGATCGAGTGTACAGTGAA
MT293212.1 Severe acute respiratory syndrome coronavirus 2 isolate SARS-CoV-2/human/USA/WA-UW-1576/2020,	TTCACCGAGGCCACGCGGAGTACGATCGAGTGTACAGTGAA
MT293213.1 Severe acute respiratory syndrome coronavirus 2 isolate SARS-CoV-2/human/USA/WA-UW-1574/2020,	TTCACCGAGGCCACGCGGAGTACGATCGAGTGTACAGTGAA
MT293214.1 Severe acute respiratory syndrome coronavirus 2 isolate SARS-CoV-2/human/USA/WA-UW-1570/2020 O	TTCACCGAGGCCACGCGGAGTACGATCGAGTGTACAGTGAA
MT293216.1 Severe acute respiratory syndrome coronavirus 2 isolate SARS-CoV-2/human/USA/WA-UW-1562/2020,	TTCACCGAGGCCACGCGGAGTACGATCGAGTGTACAGTGAA
MT293220.1 Severe acute respiratory syndrome coronavirus 2 isolate SARS-CoV-2/human/USA/WA-UW-1470/2020,	TTCACCGAGGCCACGCGGAGTACGATCGAGTGTACAGTGAA
MT293221.1 Severe acute respiratory syndrome coronavirus 2 isolate SARS-CoV-2/human/USA/WA-UW-1471/2020 O	TTCACCGAGGCCACGCGGAGTACGATCGAGTGTACAGTGAA
MT293222.1 Severe acute respiratory syndrome coronavirus 2 isolate SARS-CoV-2/human/USA/WA-UW-1433/2020,	TTCACCGAGGCCACGCGGAGTACGATCGAGTGTACAGTGAA
MT293224.1 Severe acute respiratory syndrome coronavirus 2 isolate SARS-CoV-2/human/USA/WA-UW-1608/2020,	TTCACCGAGGCCACGCGGAGTACGATCGAGTGTACAGTGAA
MT293225.1 Severe acute respiratory syndrome coronavirus 2 isolate SARS-CoV-2/human/USA/WA-UW-1599/2020,	TTCACCGAGGCCACGCGGAGTACGATCGAGTGTACAGTGAA
MT295464.1 Severe acute respiratory syndrome coronavirus 2 isolate SARS CoV-2/human/USA/UF-1/2020, complete	TTCACCGAGGCCACGCGGAGTACGATCGAGTGTACAGTGAA
MT295465.1 Severe acute respiratory syndrome coronavirus 2 isolate SARS-CoV-2/human/USA/UF-2/2020, complete	TTCACCGAGGCCACGCGGAGTACGATCGAGTGTACAGTGAA
MT300186.2 Severe acute respiratory syndrome coronavirus 2 isolate SARS-CoV-2/human/USA/UNC_200173/2020, c	TTCACCGAGGCCACGCGGAGTACGATCGAGTGTACAGTGAA
MT304474.1 Severe acute respiratory syndrome coronavirus 2 isolate SARS-CoV-2/human/KOR/BA-ACH_2604/2020,	TTCACCGAGGCCACGCGGAGTACGATCGAGTGTACAGTGAA
MT304475.1 Severe acute respiratory syndrome coronavirus 2 isolate SARS-CoV-2/human/KOR/BA-ACH_2718/2020,	TTCACCGAGGCCACGCGGAGTACGATCGAGTGTACAGTGAA

MT304476.1 Severe acute respiratory syndrome coronavirus 2 isolate SARS-CoV-2/human/KOR/BA-ACH_2719/2020,	TTCACCGAGGCCACGCGGAGTACGATCGAGTGTACAGTGAA
MT304477.1 Severe acute respiratory syndrome coronavirus 2 isolate SARS-CoV-2/human/USA/AZ_4811/2020, comp	TTCACCGAGGCCACGCGGAGTACGATCGAGTGTACAGTGAA
MT304478.1 Severe acute respiratory syndrome coronavirus 2 isolate SARS-CoV-2/human/USA/FL_6318/2020, comp	TTCACCGAGGCCACGCGGAGTACGATCGAGTGTACAGTGAA
MT304479.1 Severe acute respiratory syndrome coronavirus 2 isolate SARS-CoV-2/human/USA/GA_1299/2020, comp	TTCACCGAGGCCACGCGGAGTACGATCGAGTGTACAGTGAA
MT304480.1 Severe acute respiratory syndrome coronavirus 2 isolate SARS-CoV-2/human/USA/GA_1320/2020, comp	TTCACCGAGGCCACGCGGAGTACGATCGAGTGTACAGTGAA
MT304481.1 Severe acute respiratory syndrome coronavirus 2 isolate SARS-CoV-2/human/USA/GA_1445/2020, comp	TTCACCGAGGCCACGCGGAGTACGATCGAGTGTACAGTGAA
MT304482.1 Severe acute respiratory syndrome coronavirus 2 isolate SARS-CoV-2/human/USA/IL_1293/2020, compl	TTCACCGAGGCCACGCGGAGTACGATCGAGTGTACAGTGAA
MT304483.1 Severe acute respiratory syndrome coronavirus 2 isolate SARS-CoV-2/human/USA/IL_1375/2020, compl	TTCACCGAGGCCACGCGGAGTACGATCGAGTGTACAGTGAA
MT304484.1 Severe acute respiratory syndrome coronavirus 2 isolate SARS-CoV-2/human/USA/NH_0004/2020, comp	TTCACCGAGGCCACGCGGAGTACGATCGAGTGTACAGTGAA
MT304485.1 Severe acute respiratory syndrome coronavirus 2 isolate SARS-CoV-2/human/USA/NH_0008/2020, comp	TTCACCGAGGCCACGCGGAGTACGATCGAGTGTACAGTGAA
MT304486.1 Severe acute respiratory syndrome coronavirus 2 isolate SARS-CoV-2/human/USA/NY_2929/2020, comp	TTCACCGAGGCCACGCGGAGTACGATCGAGTGTACAGTGAA
MT304487.1 Severe acute respiratory syndrome coronavirus 2 isolate SARS-CoV-2/human/USA/OR_5430/2020, comp	TTCACCGAGGCCACGCGGAGTACGATCGAGTGTACAGTGAA
MT304488.1 Severe acute respiratory syndrome coronavirus 2 isolate SARS-CoV-2/human/USA/RI_0556/2020, compl	TTCACCGAGGCCACGCGGAGTACGATCGAGTGTACAGTGAA
MT304489.1 Severe acute respiratory syndrome coronavirus 2 isolate SARS-CoV-2/human/USA/TX_2039/2020, comp	TTCACCGAGGCCACGCGGAGTACGATCGAGTGTACAGTGAA
MT304490.1 Severe acute respiratory syndrome coronavirus 2 isolate SARS-CoV-2/human/USA/TX_2817/2020, comp	TTCACCGAGGCCACGCGGAGTACGATCGAGTGTACAGTGAA
MT304491.1 Severe acute respiratory syndrome coronavirus 2 isolate SARS-CoV-2/human/USA/TX_2967/2020, comp	TTCACCGAGGCCACGCGGAGTACGATCGAGTGTACAGTGAA
MT308692.1 Severe acute respiratory syndrome coronavirus 2 isolate SARS-CoV-2/human/USA/MI-SC2-0001/2020 O	TTCACCGAGGCCACGCGGAGTACGATCGAGTGTACAGTGAA
MT308693.1 Severe acute respiratory syndrome coronavirus 2 isolate SARS-CoV-2/human/USA/MI-SC2-0002/2020 O	TTCACCGAGGCCACGCGGAGTACGATCGAGTGTACAGTGAA
MT308694.1 Severe acute respiratory syndrome coronavirus 2 isolate SARS-CoV-2/human/USA/MI-SC2-0003/2020 O	TTCACCGAGGCCACGCGGAGTACGATCGAGTGTACAGTGAA
MT308695.1 Severe acute respiratory syndrome coronavirus 2 isolate SARS-CoV-2/human/USA/MI-SC2-0004/2020 O	TTCACCGAGGCCACGCGGAGTACGATCGAGTGTACAGTGAA
MT308696.1 Severe acute respiratory syndrome coronavirus 2 isolate SARS-CoV-2/human/USA/MI-SC2-0005/2020 O	TTCACCGAGGCCACGCGGAGTACGATCGAGTGTACAGTGAA
MT308697.1 Severe acute respiratory syndrome coronavirus 2 isolate SARS-CoV-2/human/USA/MI-SC2-0006/2020 O	TTCACCGAGGCCACGCGGAGTACGATCGAGTGTACAGTGAA
MT308698.1 Severe acute respiratory syndrome coronavirus 2 isolate SARS-CoV-2/human/USA/MI-SC2-0007/2020 O	TTCACCGAGGCCACGCGGAGTACGATCGAGTGTACAGTGAA
MT308699.1 Severe acute respiratory syndrome coronavirus 2 isolate SARS-CoV-2/human/USA/MI-SC2-0008/2020 O	TTCACCGAGGCCACGCGGAGTACGATCGAGTGTACAGTGAA
MT308700.1 Severe acute respiratory syndrome coronavirus 2 isolate SARS-CoV-2/human/USA/MI-SC2-0009/2020 O	TTCACCGAGGCCACGCGGAGTACGATCGAGTGTACAGTGAA
MT308702.1 Severe acute respiratory syndrome coronavirus 2 isolate SARS-CoV-2/human/USA/UNC_200191/2020, c	TTCACCGAGGCCACGCGGAGTACGATCGAGTGTACAGTGAA
MT308703.1 Severe acute respiratory syndrome coronavirus 2 isolate SARS-CoV-2/human/USA/UNC_200181/2020, c	TTCACCGAGGCCACGCGGAGTACGATCGAGTGTACAGTGAA
MT308704.1 Severe acute respiratory syndrome coronavirus 2 isolate SARS-CoV-2/human/USA/UNC_200189/2020, c	TTCACCGAGGCCACGCGGAGTACGATCGAGTGTACAGTGAA
MT318827.1 Severe acute respiratory syndrome coronavirus 2 isolate SARS-CoV-2/human/DEU/HH-1/2020, complete	TTCACCGAGGCCACGCGGAGTACGATCGAGTGTACAGTGAA
MT320538.2 Severe acute respiratory syndrome coronavirus 2 isolate SARS-CoV-2/human/FRA/KRA-ROB/2020, comp	TTCACCGAGGCCACGCGGAGTACGATCGAGTGTACAGTGAA
MT322394.1 Severe acute respiratory syndrome coronavirus 2 isolate SARS-CoV-2/human/USA/VA-DCLS-0001/2020,	TTCACCGAGGCCACGCGGAGTACGATCGAGTGTACAGTGAA
MT322395.1 Severe acute respiratory syndrome coronavirus 2 isolate SARS-CoV-2/human/USA/VA-DCLS-0002/2020,	TTCACCGAGGCCACGCGGAGTACGATCGAGTGTACAGTGAA
MT322401.1 Severe acute respiratory syndrome coronavirus 2 isolate SARS-CoV-2/human/USA/VA-DCLS-0008/2020,	TTCACCGAGGCCACGCGGAGTACGATCGAGTGTACAGTGAA

MT322403.1 Severe acute respiratory syndrome coronavirus 2 isolate SARS-CoV-2/human/USA/VA-DCLS-0010/2020,	TTCACCGAGGCCACGCGGAGTACGATCGAGTGTACAGTGAA
MT322404.1 Severe acute respiratory syndrome coronavirus 2 isolate SARS-CoV-2/human/USA/VA-DCLS-0011/2020,	TTCACCGAGGCCACGCGGAGTACGATCGAGTGTACAGTGAA
MT322405.1 Severe acute respiratory syndrome coronavirus 2 isolate SARS-CoV-2/human/USA/VA-DCLS-0012/2020,	TTCACCGAGGCCACGCGGAGTACGATCGAGTGTACAGTGAA
MT322408.1 Severe acute respiratory syndrome coronavirus 2 isolate SARS-CoV-2/human/USA/VA-DCLS-0018/2020,	TTCACCGAGGCCACGCGGAGTACGATCGAGTGTACAGTGAA
MT322412.1 Severe acute respiratory syndrome coronavirus 2 isolate SARS-CoV-2/human/USA/VA-DCLS-0024/2020,	TTCACCGAGGCCACGCGGAGTACGATCGAGTGTACAGTGAA
MT322413.1 Severe acute respiratory syndrome coronavirus 2 isolate SARS-CoV-2/human/USA/VA-DCLS-0031/2020,	TTCACCGAGGCCACGCGGAGTACGATCGAGTGTACAGTGAA
MT322414.1 Severe acute respiratory syndrome coronavirus 2 isolate SARS-CoV-2/human/USA/VA-DCLS-0033/2020,	TTCACCGAGGCCACGCGGAGTACGATCGAGTGTACAGTGAA
MT322415.1 Severe acute respiratory syndrome coronavirus 2 isolate SARS-CoV-2/human/USA/VA-DCLS-0034/2020,	TTCACCGAGGCCACGCGGAGTACGATCGAGTGTACAGTGAA
MT322417.1 Severe acute respiratory syndrome coronavirus 2 isolate SARS-CoV-2/human/USA/VA-DCLS-0038/2020,	TTCACCGAGGCCACGCGGAGTACGATCGAGTGTACAGTGAA
MT322421.1 Severe acute respiratory syndrome coronavirus 2 isolate SARS-CoV-2/human/USA/VA-DCLS-0043/2020,	TTCACCGAGGCCACGCGGAGTACGATCGAGTGTACAGTGAA
MT324062.1 Severe acute respiratory syndrome coronavirus 2 isolate SARS-CoV-2/human/ZAF/R03006/2020, comple	TTCACCGAGGCCACGCGGAGTACGATCGAGTGTACAGTGAA
MT324684.1 Severe acute respiratory syndrome coronavirus 2 isolate SARS-CoV-2/ENV/USA/UF-3/2020, complete ge	TTCACCGAGGCCACGCGGAGTACGATCGAGTGTACAGTGAA
MT325561.1 Severe acute respiratory syndrome coronavirus 2 isolate SARS-CoV-2/human/USA/AZ_8132/2020, comp	TTCACCGAGGCCACGCGGAGTACGATCGAGTGTACAGTGAA
MT325562.1 Severe acute respiratory syndrome coronavirus 2 isolate SARS-CoV-2/human/USA/AZ_8135/2020, comp	TTCACCGAGGCCACGCGGAGTACGATCGAGTGTACAGTGAA
MT325563.1 Severe acute respiratory syndrome coronavirus 2 isolate SARS-CoV-2/human/USA/DC_0004/2020, comp	TTCACCGAGGCCACGCGGAGTACGATCGAGTGTACAGTGAA
MT325564.1 Severe acute respiratory syndrome coronavirus 2 isolate SARS-CoV-2/human/USA/FL_1177/2020, comp	TTCACCGAGGCCACGCGGAGTACGATCGAGTGTACAGTGAA
MT325565.1 Severe acute respiratory syndrome coronavirus 2 isolate SARS-CoV-2/human/USA/FL_7169/2020, comp	TTCACCGAGGCCACGCGGAGTACGATCGAGTGTACAGTGAA
MT325566.1 Severe acute respiratory syndrome coronavirus 2 isolate SARS-CoV-2/human/USA/FL_9590/2020, comp	TTCACCGAGGCCACGCGGAGTACGATCGAGTGTACAGTGAA
MT325567.1 Severe acute respiratory syndrome coronavirus 2 isolate SARS-CoV-2/human/USA/FL_9655/2020, comp	TTCACCGAGGCCACGCGGAGTACGATCGAGTGTACAGTGAA
MT325568.1 Severe acute respiratory syndrome coronavirus 2 isolate SARS-CoV-2/human/USA/FL_9656/2020, comp	TTCACCGAGGCCACGCGGAGTACGATCGAGTGTACAGTGAA
MT325569.1 Severe acute respiratory syndrome coronavirus 2 isolate SARS-CoV-2/human/USA/GA_1621/2020, comp	TTCACCGAGGCCACGCGGAGTACGATCGAGTGTACAGTGAA
MT325570.1 Severe acute respiratory syndrome coronavirus 2 isolate SARS-CoV-2/human/USA/GA_1847/2020, comp	TTCACCGAGGCCACGCGGAGTACGATCGAGTGTACAGTGAA
MT325571.1 Severe acute respiratory syndrome coronavirus 2 isolate SARS-CoV-2/human/USA/GA_2055/2020, comp	TTCACCGAGGCCACGCGGAGTACGATCGAGTGTACAGTGAA
MT325572.1 Severe acute respiratory syndrome coronavirus 2 isolate SARS-CoV-2/human/USA/GA_2118/2020, comp	TTCACCGAGGCCACGCGGAGTACGATCGAGTGTACAGTGAA
MT325573.1 Severe acute respiratory syndrome coronavirus 2 isolate SARS-CoV-2/human/USA/GA_2120/2020, comp	TTCACCGAGGCCACGCGGAGTACGATCGAGTGTACAGTGAA
MT325574.1 Severe acute respiratory syndrome coronavirus 2 isolate SARS-CoV-2/human/USA/GA_2185/2020, comp	TTCACCGAGGCCACGCGGAGTACGATCGAGTGTACAGTGAA
MT325575.1 Severe acute respiratory syndrome coronavirus 2 isolate SARS-CoV-2/human/USA/GA_2343/2020, comp	TTCACCGAGGCCACGCGGAGTACGATCGAGTGTACAGTGAA
MT325576.1 Severe acute respiratory syndrome coronavirus 2 isolate SARS-CoV-2/human/USA/IA_6394/2020, comp	TTCACCGAGGCCACGCGGAGTACGATCGAGTGTACAGTGAA
MT325577.1 Severe acute respiratory syndrome coronavirus 2 isolate SARS-CoV-2/human/USA/IL_0087/2020, compl	TTCACCGAGGCCACGCGGAGTACGATCGAGTGTACAGTGAA
MT325578.1 Severe acute respiratory syndrome coronavirus 2 isolate SARS-CoV-2/human/USA/IL_0089/2020, compl	TTCACCGAGGCCACGCGGAGTACGATCGAGTGTACAGTGAA
MT325579.1 Severe acute respiratory syndrome coronavirus 2 isolate SARS-CoV-2/human/USA/IN_2001/2020, comp	TTCACCGAGGCCACGCGGAGTACGATCGAGTGTACAGTGAA
MT325580.1 Severe acute respiratory syndrome coronavirus 2 isolate SARS-CoV-2/human/USA/KS_4126/2020, comp	TTCACCGAGGCCACGCGGAGTACGATCGAGTGTACAGTGAA
MT325581.1 Severe acute respiratory syndrome coronavirus 2 isolate SARS-CoV-2/human/USA/LA_0842/2020, comp	TTCACCGAGGCCACGCGGAGTACGATCGAGTGTACAGTGAA

MT325582.1 Severe acute respiratory syndrome coronavirus 2 isolate SARS-CoV-2/human/USA/MA_0020/2020, com	TTCACCGAGGCCACGCGGAGTACGATCGAGTGTACAGTGAA
MT325583.1 Severe acute respiratory syndrome coronavirus 2 isolate SARS-CoV-2/human/USA/MA_3672/2020, com	TTCACCGAGGCCACGCGGAGTACGATCGAGTGTACAGTGAA
MT325584.1 Severe acute respiratory syndrome coronavirus 2 isolate SARS-CoV-2/human/USA/MA_3699/2020, com	TTCACCGAGGCCACGCGGAGTACGATCGAGTGTACAGTGAA
MT325585.1 Severe acute respiratory syndrome coronavirus 2 isolate SARS-CoV-2/human/USA/MA_3878/2020, com	TTCACCGAGGCCACGCGGAGTACGATCGAGTGTACAGTGAA
MT325586.1 Severe acute respiratory syndrome coronavirus 2 isolate SARS-CoV-2/human/USA/MA_4811/2020, com	TTCACCGAGGCCACGCGGAGTACGATCGAGTGTACAGTGAA
MT325587.1 Severe acute respiratory syndrome coronavirus 2 isolate SARS-CoV-2/human/USA/MD_0026/2020, com	TTCACCGAGGCCACGCGGAGTACGATCGAGTGTACAGTGAA
MT325588.1 Severe acute respiratory syndrome coronavirus 2 isolate SARS-CoV-2/human/USA/MO_4470/2020, com	TTCACCGAGGCCACGCGGAGTACGATCGAGTGTACAGTGAA
MT325589.1 Severe acute respiratory syndrome coronavirus 2 isolate SARS-CoV-2/human/USA/NC_0024/2020, comp	TTCACCGAGGCCACGCGGAGTACGATCGAGTGTACAGTGAA
MT325590.1 Severe acute respiratory syndrome coronavirus 2 isolate SARS-CoV-2/human/USA/NC_0031/2020, comp	TTCACCGAGGCCACGCGGAGTACGATCGAGTGTACAGTGAA
MT325591.1 Severe acute respiratory syndrome coronavirus 2 isolate SARS-CoV-2/human/USA/NC_6999/2020, comp	TTCACCGAGGCCACGCGGAGTACGATCGAGTGTACAGTGAA
MT325592.1 Severe acute respiratory syndrome coronavirus 2 isolate SARS-CoV-2/human/USA/NE_6605/2020, comp	TTCACCGAGGCCACGCGGAGTACGATCGAGTGTACAGTGAA
MT325593.1 Severe acute respiratory syndrome coronavirus 2 isolate SARS-CoV-2/human/USA/NE_7025/2020, comp	TTCACCGAGGCCACGCGGAGTACGATCGAGTGTACAGTGAA
MT325594.1 Severe acute respiratory syndrome coronavirus 2 isolate SARS-CoV-2/human/USA/NH_0029/2020, comp	TTCACCGAGGCCACGCGGAGTACGATCGAGTGTACAGTGAA
MT325595.1 Severe acute respiratory syndrome coronavirus 2 isolate SARS-CoV-2/human/USA/NH_0033/2020, comp	TTCACCGAGGCCACGCGGAGTACGATCGAGTGTACAGTGAA
MT325596.1 Severe acute respiratory syndrome coronavirus 2 isolate SARS-CoV-2/human/USA/NJ_3592/2020, comp	TTCACCGAGGCCACGCGGAGTACGATCGAGTGTACAGTGAA
MT325597.1 Severe acute respiratory syndrome coronavirus 2 isolate SARS-CoV-2/human/USA/NV_0052/2020, comp	TTCACCGAGGCCACGCGGAGTACGATCGAGTGTACAGTGAA
MT325598.1 Severe acute respiratory syndrome coronavirus 2 isolate SARS-CoV-2/human/USA/OH_0023/2020, comp	TTCACCGAGGCCACGCGGAGTACGATCGAGTGTACAGTGAA
MT325600.1 Severe acute respiratory syndrome coronavirus 2 isolate SARS-CoV-2/human/USA/PA_1881/2020, comp	TTCACCGAGGCCACGCGGAGTACGATCGAGTGTACAGTGAA
MT325601.1 Severe acute respiratory syndrome coronavirus 2 isolate SARS-CoV-2/human/USA/PA_2317/2020, comp	TTCACCGAGGCCACGCGGAGTACGATCGAGTGTACAGTGAA
MT325602.1 Severe acute respiratory syndrome coronavirus 2 isolate SARS-CoV-2/human/USA/PA_2937/2020, comp	TTCACCGAGGCCACGCGGAGTACGATCGAGTGTACAGTGAA
MT325603.1 Severe acute respiratory syndrome coronavirus 2 isolate SARS-CoV-2/human/USA/PA_4395/2020, comp	TTCACCGAGGCCACGCGGAGTACGATCGAGTGTACAGTGAA
MT325604.1 Severe acute respiratory syndrome coronavirus 2 isolate SARS-CoV-2/human/USA/PA_4405/2020, comp	TTCACCGAGGCCACGCGGAGTACGATCGAGTGTACAGTGAA
MT325605.1 Severe acute respiratory syndrome coronavirus 2 isolate SARS-CoV-2/human/USA/RI_0702/2020, compl	TTCACCGAGGCCACGCGGAGTACGATCGAGTGTACAGTGAA
MT325606.1 Severe acute respiratory syndrome coronavirus 2 isolate SARS-CoV-2/human/USA/SC_3569/2020, comp	TTCACCGAGGCCACGCGGAGTACGATCGAGTGTACAGTGAA
MT325607.1 Severe acute respiratory syndrome coronavirus 2 isolate SARS-CoV-2/human/USA/SC_3575/2020, comp	TTCACCGAGGCCACGCGGAGTACGATCGAGTGTACAGTGAA
MT325608.1 Severe acute respiratory syndrome coronavirus 2 isolate SARS-CoV-2/human/USA/SC_6370/2020, comp	TTCACCGAGGCCACGCGGAGTACGATCGAGTGTACAGTGAA
MT325609.1 Severe acute respiratory syndrome coronavirus 2 isolate SARS-CoV-2/human/USA/UT_8199/2020, comp	TTCACCGAGGCCACGCGGAGTACGATCGAGTGTACAGTGAA
MT325610.1 Severe acute respiratory syndrome coronavirus 2 isolate SARS-CoV-2/human/USA/UT_8906/2020, comp	TTCACCGAGGCCACGCGGAGTACGATCGAGTGTACAGTGAA
MT325611.1 Severe acute respiratory syndrome coronavirus 2 isolate SARS-CoV-2/human/USA/VA_6352/2020, comp	TTCACCGAGGCCACGCGGAGTACGATCGAGTGTACAGTGAA
MT325612.1 Severe acute respiratory syndrome coronavirus 2 isolate SARS-CoV-2/human/USA/VA_6377/2020, comp	TTCACCGAGGCCACGCGGAGTACGATCGAGTGTACAGTGAA
MT325613.1 Severe acute respiratory syndrome coronavirus 2 isolate SARS-CoV-2/human/USA/VA_6382/2020, comp	TTCACCGAGGCCACGCGGAGTACGATCGAGTGTACAGTGAA
MT325614.1 Severe acute respiratory syndrome coronavirus 2 isolate SARS-CoV-2/human/USA/VA_6389/2020, comp	TTCACCGAGGCCACGCGGAGTACGATCGAGTGTACAGTGAA
MT325615.1 Severe acute respiratory syndrome coronavirus 2 isolate SARS-CoV-2/human/USA/IA_6391/2020, comp	TTCACCGAGGCCACGCGGAGTACGATCGAGTGTACAGTGAA

MT325616.1 Severe acute respiratory syndrome coronavirus 2 isolate SARS-CoV-2/human/USA/MA_3623/2020, com	TTCACCGAGGCCACGCGGAGTACGATCGAGTGTACAGTGAA
MT325617.1 Severe acute respiratory syndrome coronavirus 2 isolate SARS-CoV-2/human/USA/IA_6396/2020, comp	TTCACCGAGGCCACGCGGAGTACGATCGAGTGTACAGTGAA
MT325618.1 Severe acute respiratory syndrome coronavirus 2 isolate SARS-CoV-2/human/USA/IA_6395/2020, comp	TTCACCGAGGCCACGCGGAGTACGATCGAGTGTACAGTGAA
MT325619.1 Severe acute respiratory syndrome coronavirus 2 isolate SARS-CoV-2/human/USA/IA_6401/2020, comp	TTCACCGAGGCCACGCGGAGTACGATCGAGTGTACAGTGAA
MT325620.1 Severe acute respiratory syndrome coronavirus 2 isolate SARS-CoV-2/human/USA/IA_6399/2020, comp	TTCACCGAGGCCACGCGGAGTACGATCGAGTGTACAGTGAA
MT325621.1 Severe acute respiratory syndrome coronavirus 2 isolate SARS-CoV-2/human/USA/IA_6390/2020, comp	TTCACCGAGGCCACGCGGAGTACGATCGAGTGTACAGTGAA
MT325622.1 Severe acute respiratory syndrome coronavirus 2 isolate SARS-CoV-2/human/USA/NJ_3201/2020, comp	TTCACCGAGGCCACGCGGAGTACGATCGAGTGTACAGTGAA
MT325623.1 Severe acute respiratory syndrome coronavirus 2 isolate SARS-CoV-2/human/USA/SC_3520/2020, comp	TTCACCGAGGCCACGCGGAGTACGATCGAGTGTACAGTGAA
MT325624.1 Severe acute respiratory syndrome coronavirus 2 isolate SARS-CoV-2/human/USA/SC_3570/2020, comp	TTCACCGAGGCCACGCGGAGTACGATCGAGTGTACAGTGAA
MT325625.1 Severe acute respiratory syndrome coronavirus 2 isolate SARS-CoV-2/human/USA/SC_3571/2020, comp	TTCACCGAGGCCACGCGGAGTACGATCGAGTGTACAGTGAA
MT325626.1 Severe acute respiratory syndrome coronavirus 2 isolate SARS-CoV-2/human/USA/SC_3572/2020, comp	TTCACCGAGGCCACGCGGAGTACGATCGAGTGTACAGTGAA
MT325627.1 Severe acute respiratory syndrome coronavirus 2 isolate SARS-CoV-2/human/USA/NY_1922/2020, comp	TTCACCGAGGCCACGCGGAGTACGATCGAGTGTACAGTGAA
MT325628.1 Severe acute respiratory syndrome coronavirus 2 isolate SARS-CoV-2/human/USA/VA_6171/2020, comp	TTCACCGAGGCCACACGGAGTACGATCGAGTGTACAGTGAA
MT325629.1 Severe acute respiratory syndrome coronavirus 2 isolate SARS-CoV-2/human/USA/MA_8932/2020, com	TTCACCGAGGCCACGCGGAGTACGATCGAGTGTACAGTGAA
MT325630.1 Severe acute respiratory syndrome coronavirus 2 isolate SARS-CoV-2/human/USA/MA_8933/2020, com	TTCACCGAGGCCACGCGGAGTACGATCGAGTGTACAGTGAA
MT325631.1 Severe acute respiratory syndrome coronavirus 2 isolate SARS-CoV-2/human/USA/MA_9703/2020, com	TTCACCGAGGCCACGCGGAGTACGATCGAGTGTACAGTGAA
MT325632.1 Severe acute respiratory syndrome coronavirus 2 isolate SARS-CoV-2/human/USA/MA_9704/2020, com	TTCACCGAGGCCACGCGGAGTACGATCGAGTGTACAGTGAA
MT325633.1 Severe acute respiratory syndrome coronavirus 2 isolate SARS-CoV-2/human/USA/MA_9889/2020, com	TTCACCGAGGCCACGCGGAGTACGATCGAGTGTACAGTGAA
MT325634.1 Severe acute respiratory syndrome coronavirus 2 isolate SARS-CoV-2/human/USA/MA_1355/2020, com	TTCACCGAGGCCACGCGGAGTACGATCGAGTGTACAGTGAA
MT325635.1 Severe acute respiratory syndrome coronavirus 2 isolate SARS-CoV-2/human/USA/MA_2652/2020, com	TTCACCGAGGCCACGCGGAGTACGATCGAGTGTACAGTGAA
MT325636.1 Severe acute respiratory syndrome coronavirus 2 isolate SARS-CoV-2/human/USA/MA_3614/2020, com	TTCACCGAGGCCACGCGGAGTACGATCGAGTGTACAGTGAA
MT325637.1 Severe acute respiratory syndrome coronavirus 2 isolate SARS-CoV-2/human/USA/MA_3616/2020, com	TTCACCGAGGCCACGCGGAGTACGATCGAGTGTACAGTGAA
MT325638.1 Severe acute respiratory syndrome coronavirus 2 isolate SARS-CoV-2/human/USA/MA_3626/2020, com	TTCACCGAGGCCACGCGGAGTACGATCGAGTGTACAGTGAA
MT325639.1 Severe acute respiratory syndrome coronavirus 2 isolate SARS-CoV-2/human/USA/MA_3642/2020, com	TTCACCGAGGCCACGCGGAGTACGATCGAGTGTACAGTGAA
MT325640.1 Severe acute respiratory syndrome coronavirus 2 isolate SARS-CoV-2/human/USA/MA_3653/2020, com	TTCACCGAGGCCACGCGGAGTACGATCGAGTGTACAGTGAA
MT326023.1 Severe acute respiratory syndrome coronavirus 2 isolate SARS-CoV-2/human/USA/WA-UW-2006/2020,	TTCACCGAGGCCACGCGGAGTACGATCGAGTGTACAGTGAA
MT326024.1 Severe acute respiratory syndrome coronavirus 2 isolate SARS-CoV-2/human/USA/WA-UW-1994/2020,	TTCACCGAGGCCACGCGGAGTACGATCGAGTGTACAGTGAA
MT326025.1 Severe acute respiratory syndrome coronavirus 2 isolate SARS-CoV-2/human/USA/WA-UW-1998/2020 C	TTCACCGAGGCCACGCGGAGTACGATCGAGTGTACAGTGAA
MT326026.1 Severe acute respiratory syndrome coronavirus 2 isolate SARS-CoV-2/human/USA/WA-UW-1988/2020 C	TTCACCGAGGCCACGCGGAGTACGATCGAGTGTACAGTGAA
MT326027.1 Severe acute respiratory syndrome coronavirus 2 isolate SARS-CoV-2/human/USA/WA-UW-1977/2020,	TTCACCGAGGCCACGCGGAGTACGATCGAGTGTACAGTGAA
MT326028.1 Severe acute respiratory syndrome coronavirus 2 isolate SARS-CoV-2/human/USA/WA-UW-1986/2020,	TTCACCGAGGCCACGCGGAGTACGATCGAGTGTACAGTGAA
MT326030.1 Severe acute respiratory syndrome coronavirus 2 isolate SARS-CoV-2/human/USA/WA-UW-2009/2020,	TTCACCGAGGCCACGCGGAGTACGATCGAGTGTACAGTGAA
MT326033.1 Severe acute respiratory syndrome coronavirus 2 isolate SARS-CoV-2/human/USA/WA-UW-1866/2020 C	TTCACCGAGGCCACGCGGAGTACGATCGAGTGTACAGTGAA

MT326034.1 Severe acute respiratory syndrome coronavirus 2 isolate SARS-CoV-2/human/USA/WA-UW-1850/2020	TTCACCGAGGCCACGCGGAGTACGATCGAGTGTACAGTGAA
MT326036.1 Severe acute respiratory syndrome coronavirus 2 isolate SARS-CoV-2/human/USA/WA-UW-1864/2020,	TTCACCGAGGCCACGCGGAGTACGATCGAGTGTACAGTGAA
MT326037.1 Severe acute respiratory syndrome coronavirus 2 isolate SARS-CoV-2/human/USA/WA-UW-1835/2020	TTCACCGAGGCCACGCGGAGTACGATCGAGTGTACAGTGAA
MT326038.1 Severe acute respiratory syndrome coronavirus 2 isolate SARS-CoV-2/human/USA/WA-UW-1946/2020	TTCACCGAGGCCACGCGGAGTACGATCGAGTGTACAGTGAA
MT326039.1 Severe acute respiratory syndrome coronavirus 2 isolate SARS-CoV-2/human/USA/WA-UW-1863/2020,	TTCACCGAGGCCACGCGGAGTACGATCGAGTGTACAGTGAA
MT326040.1 Severe acute respiratory syndrome coronavirus 2 isolate SARS-CoV-2/human/USA/OR-UW-1851/2020,	TTCACCGAGGCCACGCGGAGTACGATCGAGTGTACAGTGAA
MT326041.1 Severe acute respiratory syndrome coronavirus 2 isolate SARS-CoV-2/human/USA/OR-UW-1849/2020,	TTCACCGAGGCCACGCGGAGTACGATCGAGTGTACAGTGAA
MT326043.1 Severe acute respiratory syndrome coronavirus 2 isolate SARS-CoV-2/human/USA/WA-UW-1846/2020	TTCACCGAGGCCACGCGGAGTACGATCGAGTGTACAGTGAA
MT326044.1 Severe acute respiratory syndrome coronavirus 2 isolate SARS-CoV-2/human/USA/WA-UW-1860/2020	TTCACCGAGGCCACGCGGAGTACGATCGAGTGTACAGTGAA
MT326048.1 Severe acute respiratory syndrome coronavirus 2 isolate SARS-CoV-2/human/USA/WA-UW-1870/2020,	TTCACCGAGGCCACGCGGAGTACGATCGAGTGTACAGTGAA
MT326049.1 Severe acute respiratory syndrome coronavirus 2 isolate SARS-CoV-2/human/USA/WA-UW-1872/2020,	TTCACCGAGGCCACGCGGAGTACGATCGAGTGTACAGTGAA
MT326050.1 Severe acute respiratory syndrome coronavirus 2 isolate SARS-CoV-2/human/USA/WA-UW-1905/2020	TTCACCGAGGCCACGCGGAGTACGATCGAGTGTACAGTGAA
MT326051.1 Severe acute respiratory syndrome coronavirus 2 isolate SARS-CoV-2/human/USA/WA-UW-1919/2020,	TTCACCGAGGCCACGCGGAGTACGATCGAGTGTACAGTGAA
MT326052.1 Severe acute respiratory syndrome coronavirus 2 isolate SARS-CoV-2/human/USA/WA-UW-1913/2020,	TTCACCGAGGCCACGCGGAGTACGATCGAGTGTACAGTGAA
MT326054.1 Severe acute respiratory syndrome coronavirus 2 isolate SARS-CoV-2/human/USA/WA-UW-1904/2020,	TTCACCGAGGCCACGCGGAGTACGATCGAGTGTACAGTGAA
MT326057.1 Severe acute respiratory syndrome coronavirus 2 isolate SARS-CoV-2/human/USA/OR-UW-1937/2020 O	TTCACCGAGGCCACGCGGAGTACGATCGAGTGTACAGTGAA
MT326058.1 Severe acute respiratory syndrome coronavirus 2 isolate SARS-CoV-2/human/USA/WA-UW-1930/2020,	TTCACCGAGGCCACGCGGAGTACGATCGAGTGTACAGTGAA
MT326059.1 Severe acute respiratory syndrome coronavirus 2 isolate SARS-CoV-2/human/USA/WA-UW-1877/2020	TTCACCGAGGCCACGCGGAGTACGATCGAGTGTACAGTGAA
MT326062.1 Severe acute respiratory syndrome coronavirus 2 isolate SARS-CoV-2/human/USA/OR-UW-1887/2020 O	TTCACCGAGGCCACGCGGAGTACGATCGAGTGTACAGTGAA
MT326063.1 Severe acute respiratory syndrome coronavirus 2 isolate SARS-CoV-2/human/USA/WA-UW-1881/2020,	TTCACCGAGGCCACGCGGAGTACGATCGAGTGTACAGTGAA
MT326064.1 Severe acute respiratory syndrome coronavirus 2 isolate SARS-CoV-2/human/USA/WA-UW-1888/2020	TTCACCGAGGCCACGCGGAGTACGATCGAGTGTACAGTGAA
MT326065.1 Severe acute respiratory syndrome coronavirus 2 isolate SARS-CoV-2/human/USA/WA-UW-1880/2020,	TTCACCGAGGCCACGCGGAGTACGATCGAGTGTACAGTGAA
MT326066.1 Severe acute respiratory syndrome coronavirus 2 isolate SARS-CoV-2/human/USA/WA-UW-1955/2020,	TTCACCGAGGCCACGCGGAGTACGATCGAGTGTACAGTGAA
MT326067.1 Severe acute respiratory syndrome coronavirus 2 isolate SARS-CoV-2/human/USA/WA-UW-1956/2020,	TTCACCGAGGCCACGCGGAGTACGATCGAGTGTACAGTGAA
MT326068.1 Severe acute respiratory syndrome coronavirus 2 isolate SARS-CoV-2/human/USA/WA-UW-1921/2020,	TTCACCGAGGCCACGCGGAGTACGATCGAGTGTACAGTGAA
MT326070.1 Severe acute respiratory syndrome coronavirus 2 isolate SARS-CoV-2/human/USA/WA-UW-1928/2020,	TTCACCGAGGCCACGCGGAGTACGATCGAGTGTACAGTGAA
MT326071.1 Severe acute respiratory syndrome coronavirus 2 isolate SARS-CoV-2/human/USA/ID-UW-1938/2020, c	TTCACCGAGGCCACGCGGAGTACGATCGAGTGTACAGTGAA
MT326072.1 Severe acute respiratory syndrome coronavirus 2 isolate SARS-CoV-2/human/USA/ID-UW-1935/2020 O	TTCACCGAGGCCACGCGGAGTACGATCGAGTGTACAGTGAA
MT326073.1 Severe acute respiratory syndrome coronavirus 2 isolate SARS-CoV-2/human/USA/ID-UW-1939/2020 O	TTCACCGAGGCCACGCGGAGTACGATCGAGTGTACAGTGAA
MT326074.1 Severe acute respiratory syndrome coronavirus 2 isolate SARS-CoV-2/human/USA/ID-UW-1925/2020, c	TTCACCGAGGCCACGCGGAGTACGATCGAGTGTACAGTGAA
MT326075.1 Severe acute respiratory syndrome coronavirus 2 isolate SARS-CoV-2/human/USA/ID-UW-1934/2020, c	TTCACCGAGGCCACGCGGAGTACGATCGAGTGTACAGTGAA
MT326078.1 Severe acute respiratory syndrome coronavirus 2 isolate SARS-CoV-2/human/USA/WA-UW-1954/2020,	TTCACCGAGGCCACGCGGAGTACGATCGAGTGTACAGTGAA
MT326079.1 Severe acute respiratory syndrome coronavirus 2 isolate SARS-CoV-2/human/USA/WA-UW-1968/2020	TTCACCGAGGCCACGCGGAGTACGATCGAGTGTACAGTGAA

MT326080.1 Severe acute respiratory syndrome coronavirus 2 isolate SARS-CoV-2/human/USA/WA-UW-1963/2020 C	TTCACCGAGGCCACGCGGAGTACGATCGAGTGTACAGTGAA
MT326082.1 Severe acute respiratory syndrome coronavirus 2 isolate SARS-CoV-2/human/USA/WA-UW-1865/2020,	TTCACCGAGGCCACGCGGAGTACGATCGAGTGTACAGTGAA
MT326083.1 Severe acute respiratory syndrome coronavirus 2 isolate SARS-CoV-2/human/USA/WA-UW-1907/2020,	TTCACCGAGGCCACGCGGAGTACGATCGAGTGTACAGTGAA
MT326084.1 Severe acute respiratory syndrome coronavirus 2 isolate SARS-CoV-2/human/USA/WA-UW-1917/2020,	TTCACCGAGGCCACGCGGAGTACGATCGAGTGTACAGTGAA
MT326086.1 Severe acute respiratory syndrome coronavirus 2 isolate SARS-CoV-2/human/USA/WA-UW-1785/2020,	TTCACCGAGGCCACGCGGAGTACGATCGAGTGTACAGTGAA
MT326087.1 Severe acute respiratory syndrome coronavirus 2 isolate SARS-CoV-2/human/USA/WA-UW-1801/2020,	TTCACCGAGGCCACGCGGAGTACGATCGAGTGTACAGTGAA
MT326088.1 Severe acute respiratory syndrome coronavirus 2 isolate SARS-CoV-2/human/USA/WA-UW-1840/2020,	TTCACCGAGGCCACGCGGAGTACGATCGAGTGTACAGTGAA
MT326089.1 Severe acute respiratory syndrome coronavirus 2 isolate SARS-CoV-2/human/USA/WA-UW-1792/2020,	TTCACCGAGGCCACGCGGAGTACGATCGAGTGTACAGTGAA
MT326091.1 Severe acute respiratory syndrome coronavirus 2 isolate SARS-CoV-2/human/USA/UNKNOWN-UW-1832	TTCACCGAGGCCACGCGGAGTACGATCGAGTGTACAGTGAA
MT326092.1 Severe acute respiratory syndrome coronavirus 2 isolate SARS-CoV-2/human/USA/WA-UW-1774/2020,	TTCACCGAGGCCACGCGGAGTACGATCGAGTGTACAGTGAA
MT326093.1 Severe acute respiratory syndrome coronavirus 2 isolate SARS-CoV-2/human/USA/WA-UW-1775/2020,	TTCACCGAGGCCACGCGGAGTACGATCGAGTGTACAGTGAA
MT326097.1 Severe acute respiratory syndrome coronavirus 2 isolate SARS-CoV-2/human/USA/WA-UW-1784/2020,	TTCACCGAGGCCACGCGGAGTACGATCGAGTGTACAGTGAA
MT326100.1 Severe acute respiratory syndrome coronavirus 2 isolate SARS-CoV-2/human/USA/WA-UW-1805/2020,	TTCACCGAGGCCACGCGGAGTACGATCGAGTGTACAGTGAA
MT326104.1 Severe acute respiratory syndrome coronavirus 2 isolate SARS-CoV-2/human/USA/UNKNOWN-UW-1816	TTCACCGAGGCCACGCGGAGTACGATCGAGTGTACAGTGAA
MT326105.1 Severe acute respiratory syndrome coronavirus 2 isolate SARS-CoV-2/human/USA/WA-UW-1828/2020,	TTCACCGAGGCCACGCGGAGTACGATCGAGTGTACAGTGAA
MT326109.1 Severe acute respiratory syndrome coronavirus 2 isolate SARS-CoV-2/human/USA/WA-UW-1826/2020 C	TTCACCGAGGCCACGCGGAGTACGATCGAGTGTACAGTGAA
MT326112.1 Severe acute respiratory syndrome coronavirus 2 isolate SARS-CoV-2/human/USA/WA-UW-1644/2020,	TTCACCGAGGCCACGCGGAGTACGATCGAGTGTACAGTGAA
MT326113.1 Severe acute respiratory syndrome coronavirus 2 isolate SARS-CoV-2/human/USA/WA-UW-1646/2020,	TTCACCGAGGCCACGCGGAGTACGATCGAGTGTACAGTGAA
MT326114.1 Severe acute respiratory syndrome coronavirus 2 isolate SARS-CoV-2/human/USA/WA-UW-1645/2020 C	TTCACCGAGGCCACGCGGAGTACGATCGAGTGTACAGTGAA
MT326115.1 Severe acute respiratory syndrome coronavirus 2 isolate SARS-CoV-2/human/USA/WA-UW-1675/2020 C	TTCACCGAGGCCACGCGGAGTACGATCGAGTGTACAGTGAA
MT326116.1 Severe acute respiratory syndrome coronavirus 2 isolate SARS-CoV-2/human/USA/WA-UW-1683/2020,	TTCACCGAGGCCACGCGGAGTACGATCGAGTGTACAGTGAA
MT326117.1 Severe acute respiratory syndrome coronavirus 2 isolate SARS-CoV-2/human/USA/WA-UW-1688/2020,	TTCACCGAGGCCACGCGGAGTACGATCGAGTGTACAGTGAA
MT326118.1 Severe acute respiratory syndrome coronavirus 2 isolate SARS-CoV-2/human/USA/WA-UW-1728/2020,	TTCACCGAGGCCACGCGGAGTACGATCGAGTGTACAGTGAA
MT326119.1 Severe acute respiratory syndrome coronavirus 2 isolate SARS-CoV-2/human/USA/WA-UW-1661/2020,	TTCACCGAGGCCACGCGGAGTACGATCGAGTGTACAGTGAA
MT326120.1 Severe acute respiratory syndrome coronavirus 2 isolate SARS-CoV-2/human/USA/WA-UW-1726/2020,	TTCACCGAGGCCACGCGGAGTACGATCGAGTGTACAGTGAA
MT326123.1 Severe acute respiratory syndrome coronavirus 2 isolate SARS-CoV-2/human/USA/WA-UW-1656/2020,	TTCACCGAGGCCACGCGGAGTACGATCGAGTGTACAGTGAA
MT326127.1 Severe acute respiratory syndrome coronavirus 2 isolate SARS-CoV-2/human/USA/WA-UW-1651/2020,	TTCACCGAGGCCACGCGGAGTACGATCGAGTGTACAGTGAA
MT326129.1 Severe acute respiratory syndrome coronavirus 2 isolate SARS-CoV-2/human/USA/WA-UW-1698/2020,	TTCACCGAGGCCACGCGGAGTACGATCGAGTGTACAGTGAA
MT326131.1 Severe acute respiratory syndrome coronavirus 2 isolate SARS-CoV-2/human/USA/WA-UW-1673/2020 C	TTCACCGAGGCCACGCGGAGTACGATCGAGTGTACAGTGAA
MT326132.1 Severe acute respiratory syndrome coronavirus 2 isolate SARS-CoV-2/human/USA/WA-UW-1716/2020,	TTCACCGAGGCCACGCGGAGTACGATCGAGTGTACAGTGAA
MT326133.1 Severe acute respiratory syndrome coronavirus 2 isolate SARS-CoV-2/human/USA/WA-UW-1712/2020,	TTCACCGAGGCCACGCGGAGTACGATCGAGTGTACAGTGAA
MT326134.1 Severe acute respiratory syndrome coronavirus 2 isolate SARS-CoV-2/human/USA/WA-UW-1765/2020,	TTCACCGAGGCCACGCGGAGTACGATCGAGTGTACAGTGAA
MT326135.1 Severe acute respiratory syndrome coronavirus 2 isolate SARS-CoV-2/human/USA/WA-UW-1718/2020,	TTCACCGAGGCCACGCGGAGTACGATCGAGTGTACAGTGAA

MT326137.1 Severe acute respiratory syndrome coronavirus 2 isolate SARS-CoV-2/human/USA/WA-UW-1715/2020, c	TTCACCGAGGCCACGCGGAGTACGATCGAGTGTACAGTGAA
MT326139.1 Severe acute respiratory syndrome coronavirus 2 isolate SARS-CoV-2/human/USA/WA-UW-1677/2020 C	TTCACCGAGGCCACGCGGAGTACGATCGAGTGTACAGTGAA
MT326140.1 Severe acute respiratory syndrome coronavirus 2 isolate SARS-CoV-2/human/USA/WA-UW-1680/2020, c	TTCACCGAGGCCACGCGGAGTACGATCGAGTGTACAGTGAA
MT326141.1 Severe acute respiratory syndrome coronavirus 2 isolate SARS-CoV-2/human/USA/WA-UW-1690/2020 C	TTCACCGAGGCCACGCGGAGTACGATCGAGTGTACAGTGAA
MT326142.1 Severe acute respiratory syndrome coronavirus 2 isolate SARS-CoV-2/human/USA/WA-UW-1684/2020 C	TTCACCGAGGCCACGCGGAGTACGATCGAGTGTACAGTGAA
MT326145.1 Severe acute respiratory syndrome coronavirus 2 isolate SARS-CoV-2/human/USA/WA-UW-1729/2020 C	TTCACCGAGGCCACGCGGAGTACGATCGAGTGTACAGTGAA
MT326146.1 Severe acute respiratory syndrome coronavirus 2 isolate SARS-CoV-2/human/USA/WA-UW-1748/2020 C	TTCACCGAGGCCACGCGGAGTACGATCGAGTGTACAGTGAA
MT326147.1 Severe acute respiratory syndrome coronavirus 2 isolate SARS-CoV-2/human/USA/WA-UW-1700/2020 C	TTCACCGAGGCCACGCGGAGTACGATCGAGTGTACAGTGAA
MT326148.1 Severe acute respiratory syndrome coronavirus 2 isolate SARS-CoV-2/human/USA/WA-UW-1744/2020, c	TTCACCGAGGCCACGCGGAGTACGATCGAGTGTACAGTGAA
MT326149.1 Severe acute respiratory syndrome coronavirus 2 isolate SARS-CoV-2/human/USA/WA-UW-1745/2020, c	TTCACCGAGGCCACGCGGAGTACGATCGAGTGTACAGTGAA
MT326150.1 Severe acute respiratory syndrome coronavirus 2 isolate SARS-CoV-2/human/USA/WA-UW-1707/2020, c	TTCACCGAGGCCACGCGGAGTACGATCGAGTGTACAGTGAA
MT326153.1 Severe acute respiratory syndrome coronavirus 2 isolate SARS-CoV-2/human/USA/WA-UW-1702/2020, c	TTCACCGAGGCCACGCGGAGTACGATCGAGTGTACAGTGAA
MT326154.1 Severe acute respiratory syndrome coronavirus 2 isolate SARS-CoV-2/human/USA/WA-UW-1724/2020, c	TTCACCGAGGCCACGCGGAGTACGATCGAGTGTACAGTGAA
MT326159.1 Severe acute respiratory syndrome coronavirus 2 isolate SARS-CoV-2/human/USA/WA-UW-1722/2020, c	TTCACCGAGGCCACGCGGAGTACGATCGAGTGTACAGTGAA
MT326160.1 Severe acute respiratory syndrome coronavirus 2 isolate SARS-CoV-2/human/USA/WA-UW-1733/2020, c	TTCACCGAGGCCACGCGGAGTACGATCGAGTGTACAGTGAA
MT326161.1 Severe acute respiratory syndrome coronavirus 2 isolate SARS-CoV-2/human/USA/WA-UW-1732/2020, c	TTCACCGAGGCCACGCGGAGTACGATCGAGTGTACAGTGAA
MT326164.1 Severe acute respiratory syndrome coronavirus 2 isolate SARS-CoV-2/human/USA/WA-UW-1750/2020, c	TTCACCGAGGCCACGCGGAGTACGATCGAGTGTACAGTGAA
MT326167.1 Severe acute respiratory syndrome coronavirus 2 isolate SARS-CoV-2/human/USA/WA-UW-1753/2020, c	TTCACCGAGGCCACGCGGAGTACGATCGAGTGTACAGTGAA
MT326169.1 Severe acute respiratory syndrome coronavirus 2 isolate SARS-CoV-2/human/USA/WA-UW-1770/2020, c	TTCACCGAGGCCACGCGGAGTACGATCGAGTGTACAGTGAA
MT326170.1 Severe acute respiratory syndrome coronavirus 2 isolate SARS-CoV-2/human/USA/WA-UW-1762/2020 C	TTCACCGAGGCCACGCGGAGTACGATCGAGTGTACAGTGAA
MT326171.1 Severe acute respiratory syndrome coronavirus 2 isolate SARS-CoV-2/human/USA/WA-UW-1758/2020 C	TTCACCGAGGCCACGCGGAGTACGATCGAGTGTACAGTGAA
MT326172.1 Severe acute respiratory syndrome coronavirus 2 isolate SARS-CoV-2/human/USA/WA-UW-1760/2020 C	TTCACCGAGGCCACGCGGAGTACGATCGAGTGTACAGTGAA
MT326173.1 Severe acute respiratory syndrome coronavirus 2 isolate SARS-CoV-2/human/USA/UNKNOWN-UW-1773	TTCACCGAGGCCACGCGGAGTACGATCGAGTGTACAGTGAA
MT326174.1 Severe acute respiratory syndrome coronavirus 2 isolate SARS-CoV-2/human/USA/WA-UW-1620/2020 C	TTCACCGAGGCCACGCGGAGTACGATCGAGTGTACAGTGAA
MT326175.1 Severe acute respiratory syndrome coronavirus 2 isolate SARS-CoV-2/human/USA/WA-UW-1616/2020 C	TTCACCGAGGCCACGCGGAGTACGATCGAGTGTACAGTGAA
MT326176.1 Severe acute respiratory syndrome coronavirus 2 isolate SARS-CoV-2/human/USA/WA-UW-1638/2020 C	TTCACCGAGGCCACGCGGAGTACGATCGAGTGTACAGTGAA
MT326177.1 Severe acute respiratory syndrome coronavirus 2 isolate SARS-CoV-2/human/USA/WA-UW-1538/2020 C	TTCACCGAGGCCACGCGGAGTACGATCGAGTGTACAGTGAA
MT326178.1 Severe acute respiratory syndrome coronavirus 2 isolate SARS-CoV-2/human/USA/WA-UW-1595/2020, c	TTCACCGAGGCCACGCGGAGTACGATCGAGTGTACAGTGAA
MT326183.1 Severe acute respiratory syndrome coronavirus 2 isolate SARS-CoV-2/human/USA/WA-UW-1362/2020 C	TTCACCGAGGCCACGCGGAGTACGATCGAGTGTACAGTGAA
MT326184.1 Severe acute respiratory syndrome coronavirus 2 isolate SARS-CoV-2/human/USA/WA-UW-1472/2020, c	TTCACCGAGGCCACGCGGAGTACGATCGAGTGTACAGTGAA
MT326188.1 Severe acute respiratory syndrome coronavirus 2 isolate SARS-CoV-2/human/USA/UNKNOWN-UW-1402	TTCACCGAGGCCACGCGGAGTACGATCGAGTGTACAGTGAA
MT328032.1 Severe acute respiratory syndrome coronavirus 2 isolate SARS-CoV-2/human/GRC/10/2020, complete ge	TTCACCGAGGCCACGCGGAGTACGATCGAGTGTACAGTGAA
MT328033.1 Severe acute respiratory syndrome coronavirus 2 isolate SARS-CoV-2/human/GRC/12/2020, complete ge	TTCACCGAGGCCACGCGGAGTACGATCGAGTGTACAGTGAA

MT328034.1 Severe acute respiratory syndrome coronavirus 2 isolate SARS-CoV-2/human/GRC/16/2020, complete ge	TTCACCGAGGCCACGCGGAGTACGATCGAGTGTACAGTGAA
MT328035.1 Severe acute respiratory syndrome coronavirus 2 isolate SARS-CoV-2/human/GRC/13/2020, complete ge	TTCACCGAGGCCACGCGGAGTACGATCGAGTGTACAGTGAA
MT334522.1 Severe acute respiratory syndrome coronavirus 2 isolate SARS-CoV-2/human/USA/UT-00001/2020 ORF1	TTCACCGAGGCCACGCGGAGTACGATCGAGTGTACAGTGAA
MT334523.1 Severe acute respiratory syndrome coronavirus 2 isolate SARS-CoV-2/human/USA/UT-00003/2020, com	TTCACCGAGGCCACGCGGAGTACGATCGAGTGTACAGTGAA
MT334526.1 Severe acute respiratory syndrome coronavirus 2 isolate SARS-CoV-2/human/USA/UT-00006/2020, com	TTCACCGAGGCCACGCGGAGTACGATCGAGTGTACAGTGAA
MT334527.1 Severe acute respiratory syndrome coronavirus 2 isolate SARS-CoV-2/human/USA/UT-00008/2020 ORF1	TTCACCGAGGCCACGCGGAGTACGATCGAGTGTACAGTGAA
MT334533.1 Severe acute respiratory syndrome coronavirus 2 isolate SARS-CoV-2/human/USA/UT-00014/2020, com	TTCACCGAGGCCACGCGGAGTACGATCGAGTGTACAGTGAA
MT334534.1 Severe acute respiratory syndrome coronavirus 2 isolate SARS-CoV-2/human/USA/UT-00015/2020, com	TTCACCGAGGCCACGCGGAGTACGATCGAGTGTACAGTGAA
MT334535.1 Severe acute respiratory syndrome coronavirus 2 isolate SARS-CoV-2/human/USA/UT-00016/2020, com	TTCACCGAGGCCACGCGGAGTACGATCGAGTGTACAGTGAA
MT334537.1 Severe acute respiratory syndrome coronavirus 2 isolate SARS-CoV-2/human/USA/UT-00019/2020, com	TTCACCGAGGCCACGCGGAGTACGATCGAGTGTACAGTGAA
MT334538.1 Severe acute respiratory syndrome coronavirus 2 isolate SARS-CoV-2/human/USA/UT-00020/2020, com	TTCACCGAGGCCACGCGGAGTACGATCGAGTGTACAGTGAA
MT334540.1 Severe acute respiratory syndrome coronavirus 2 isolate SARS-CoV-2/human/USA/UT-00022/2020, com	TTCACCGAGGCCACGCGGAGTACGATCGAGTGTACAGTGAA
MT334541.1 Severe acute respiratory syndrome coronavirus 2 isolate SARS-CoV-2/human/USA/UT-00023/2020, com	TTCACCGAGGCCACGCGGAGTACGATCGAGTGTACAGTGAA
MT334542.1 Severe acute respiratory syndrome coronavirus 2 isolate SARS-CoV-2/human/USA/UT-00025/2020, com	TTCACCGAGGCCACGCGGAGTACGATCGAGTGTACAGTGAA
MT334544.1 Severe acute respiratory syndrome coronavirus 2 isolate SARS-CoV-2/human/USA/UT-00028/2020, com	TTCACCGAGGCCACGCGGAGTACGATCGAGTGTACAGTGAA
MT334546.1 Severe acute respiratory syndrome coronavirus 2 isolate SARS-CoV-2/human/USA/UT-00032/2020, com	TTCACCGAGGCCACGCGGAGTACGATCGAGTGTACAGTGAA
MT334547.1 Severe acute respiratory syndrome coronavirus 2 isolate SARS-CoV-2/human/USA/UT-00033/2020, com	TTCACCGAGGCCACGCGGAGTACGATCGAGTGTACAGTGAA
MT334548.1 Severe acute respiratory syndrome coronavirus 2 isolate SARS-CoV-2/human/USA/UT-00034/2020 ORF1	TTCACCGAGGCCACGCGGAGTACGATCGAGTGTACAGTGAA
MT334550.1 Severe acute respiratory syndrome coronavirus 2 isolate SARS-CoV-2/human/USA/UT-00089/2020 ORF1	TTCACCGAGGCCACGCGGAGTACGATCGAGTGTACAGTGAA
MT334552.1 Severe acute respiratory syndrome coronavirus 2 isolate SARS-CoV-2/human/USA/UT-00159/2020 ORF1	TTCACCGAGGCCACGCGGAGTACGATCGAGTGTACAGTGAA
MT334554.1 Severe acute respiratory syndrome coronavirus 2 isolate SARS-CoV-2/human/USA/UT-00287/2020 ORF1	TTCACCGAGGCCACGCGGAGTACGATCGAGTGTACAGTGAA
MT334556.1 Severe acute respiratory syndrome coronavirus 2 isolate SARS-CoV-2/human/USA/UT-00289/2020 ORF1	TTCACCGAGGCCACGCGGAGTACGATCGAGTGTACAGTGAA
MT334557.1 Severe acute respiratory syndrome coronavirus 2 isolate SARS-CoV-2/human/USA/UT-00290/2020, com	TTCACCGAGGCCACGCGGAGTACGATCGAGTGTACAGTGAA
MT334558.1 Severe acute respiratory syndrome coronavirus 2 isolate SARS-CoV-2/human/USA/UT-00291/2020 ORF1	TTCACCGAGGCCACGCGGAGTACGATCGAGTGTACAGTGAA
MT334559.1 Severe acute respiratory syndrome coronavirus 2 isolate SARS-CoV-2/human/USA/UT-00297/2020 ORF1	TTCACCGAGGCCACGCGGAGTACGATCGAGTGTACAGTGAA
MT334560.1 Severe acute respiratory syndrome coronavirus 2 isolate SARS-CoV-2/human/USA/UT-00300/2020 ORF1	TTCACCGAGGCCACGCGGAGTACGATCGAGTGTACAGTGAA
MT334561.1 Severe acute respiratory syndrome coronavirus 2 isolate SARS-CoV-2/human/USA/UT-00301/2020, com	TTCACCGAGGCCACGCGGAGTACGATCGAGTGTACAGTGAA
MT334563.1 Severe acute respiratory syndrome coronavirus 2 isolate SARS-CoV-2/human/USA/UT-00303/2020 ORF1	TTCACCGAGGCCACGCGGAGTACGATCGAGTGTACAGTGAA
MT334569.1 Severe acute respiratory syndrome coronavirus 2 isolate SARS-CoV-2/human/USA/UT-00348/2020 ORF1	TTCACCGAGGCCACGCGGAGTACGATCGAGTGTACAGTGAA
MT334570.1 Severe acute respiratory syndrome coronavirus 2 isolate SARS-CoV-2/human/USA/UT-00349/2020 ORF1	TTCACCGAGGCCACGCGGAGTACGATCGAGTGTACAGTGAA
MT334571.1 Severe acute respiratory syndrome coronavirus 2 isolate SARS-CoV-2/human/USA/UT-00350/2020 ORF1	TTCACCGAGGCCACGCGGAGTACGATCGAGTGTACAGTGAA
MT334572.1 Severe acute respiratory syndrome coronavirus 2 isolate SARS-CoV-2/human/USA/UT-00352/2020 ORF1	TTCACCGAGGCCACGCGGAGTACGATCGAGTGTACAGTGAA
MT334573.1 Severe acute respiratory syndrome coronavirus 2 isolate SARS-CoV-2/human/USA/UT-00361/2020 ORF1	TTCACCGAGGCCACGCGGAGTACGATCGAGTGTACAGTGAA

MT339039.1 Severe acute respiratory syndrome coronavirus 2 isolate SARS-CoV-2/human/USA/AZ-ASU2922/2020, co	TTCACCGAGGCCACGCGGAGTACGATCGAGTGTACAGTGAA
MT339040.1 Severe acute respiratory syndrome coronavirus 2 isolate SARS-CoV-2/human/USA/AZ-ASU2923/2020, co	TTCACCGAGGCCACGCGGAGTACGATCGAGTGTACAGTGAA
MT339041.1 Severe acute respiratory syndrome coronavirus 2 isolate SARS-CoV-2/human/USA/AZ-ASU2936/2020, co	TTCACCGAGGCCACGCGGAGTACGATCGAGTGTACAGTGAA
MT344944.1 Severe acute respiratory syndrome coronavirus 2 isolate SARS-CoV-2/human/USA/CT_9849/2020, comp	TTCACCGAGGCCACGCGGAGTACGATCGAGTGTACAGTGAA
MT344945.1 Severe acute respiratory syndrome coronavirus 2 isolate SARS-CoV-2/human/USA/GA_2070/2020, comp	TTCACCGAGGCCACGCGGAGTACGATCGAGTGTACAGTGAA
MT344946.1 Severe acute respiratory syndrome coronavirus 2 isolate SARS-CoV-2/human/USA/GA_2147/2020, comp	TTCACCGAGGCCACGCGGAGTACGATCGAGTGTACAGTGAA
MT344947.1 Severe acute respiratory syndrome coronavirus 2 isolate SARS-CoV-2/human/USA/GA_2275/2020, comp	TTCACCGAGGCCACGCGGAGTACGATCGAGTGTACAGTGAA
MT344948.1 Severe acute respiratory syndrome coronavirus 2 isolate SARS-CoV-2/human/USA/HI_4970/2020, comp	TTCACCGAGGCCACGCGGAGTACGATCGAGTGTACAGTGAA
MT344949.1 Severe acute respiratory syndrome coronavirus 2 isolate SARS-CoV-2/human/USA/HI_7881/2020, comp	TTCACCGAGGCCACGCGGAGTACGATCGAGTGTACAGTGAA
MT344954.1 Severe acute respiratory syndrome coronavirus 2 isolate SARS-CoV-2/human/USA/MN_0100/2020, com	TTCACCGAGGCCACGCGGAGTACGATCGAGTGTACAGTGAA
MT344955.1 Severe acute respiratory syndrome coronavirus 2 isolate SARS-CoV-2/human/USA/OH_0019/2020, comp	TTCACCGAGGCCACGCGGAGTACGATCGAGTGTACAGTGAA
MT344956.1 Severe acute respiratory syndrome coronavirus 2 isolate SARS-CoV-2/human/USA/OH_0020/2020, comp	TTCACCGAGGCCACGCGGAGTACGATCGAGTGTACAGTGAA
MT344957.1 Severe acute respiratory syndrome coronavirus 2 isolate SARS-CoV-2/human/USA/PA_2734/2020, comp	TTCACCGAGGCCACGCGGAGTACGATCGAGTGTACAGTGAA
MT344958.1 Severe acute respiratory syndrome coronavirus 2 isolate SARS-CoV-2/human/USA/PA_2735/2020, comp	TTCACCGAGGCCACGCGGAGTACGATCGAGTGTACAGTGAA
MT344959.1 Severe acute respiratory syndrome coronavirus 2 isolate SARS-CoV-2/human/USA/PA_2887/2020, comp	TTCACCGAGGCCACGCGGAGTACGATCGAGTGTACAGTGAA
MT344960.1 Severe acute respiratory syndrome coronavirus 2 isolate SARS-CoV-2/human/USA/RI_0882/2020, compl	TTCACCGAGGCCACGCGGAGTACGATCGAGTGTACAGTGAA
MT344961.1 Severe acute respiratory syndrome coronavirus 2 isolate SARS-CoV-2/human/USA/NV_0016/2020, comp	TTCACCGAGGCCACGCGGAGTACGATCGAGTGTACAGTGAA
MT344962.1 Severe acute respiratory syndrome coronavirus 2 isolate SARS-CoV-2/human/USA/MN_0101/2020, com	TTCACCGAGGCCACGCGGAGTACGATCGAGTGTACAGTGAA
MT344963.1 Severe acute respiratory syndrome coronavirus 2 isolate SARS-CoV-2/human/USA/NC_0025/2020, comp	TTCACCGAGGCCACGCGGAGTACGATCGAGTGTACAGTGAA
MT345798.1 Severe acute respiratory syndrome coronavirus 2 isolate SARS-CoV-2/human/USA/WA-UW-2091/2020, c	TTCACCGAGGCCACGCGGAGTACGATCGAGTGTACAGTGAA
MT345799.1 Severe acute respiratory syndrome coronavirus 2 isolate SARS-CoV-2/human/USA/WA-UW-2169/2020, c	TTCACCGAGGCCACGCGGAGTACGATCGAGTGTACAGTGAA
MT345800.1 Severe acute respiratory syndrome coronavirus 2 isolate SARS-CoV-2/human/USA/WA-UW-2082/2020 C	TTCACCGAGGCCACGCGGAGTACGATCGAGTGTACAGTGAA
MT345801.1 Severe acute respiratory syndrome coronavirus 2 isolate SARS-CoV-2/human/USA/WA-UW-2131/2020, c	TTCACCGAGGCCACGCGGAGTACGATCGAGTGTACAGTGAA
MT345803.1 Severe acute respiratory syndrome coronavirus 2 isolate SARS-CoV-2/human/USA/ID-UW-2127/2020, co	TTCACCGAGGCCACGCGGAGTACGATCGAGTGTACAGTGAA
MT345804.1 Severe acute respiratory syndrome coronavirus 2 isolate SARS-CoV-2/human/USA/WA-UW-2072/2020 C	TTCACCGAGGCCACGCGGAGTACGATCGAGTGTACAGTGAA
MT345807.1 Severe acute respiratory syndrome coronavirus 2 isolate SARS-CoV-2/human/USA/WA-UW-2074/2020 C	TTCACCGAGGCCACGCGGAGTACGATCGAGTGTACAGTGAA
MT345808.1 Severe acute respiratory syndrome coronavirus 2 isolate SARS-CoV-2/human/USA/WA-UW-2132/2020, c	TTCACCGAGGCCACGCGGAGTACGATCGAGTGTACAGTGAA
MT345809.1 Severe acute respiratory syndrome coronavirus 2 isolate SARS-CoV-2/human/USA/WA-UW-2119/2020, c	TTCACCGAGGCCACGCGGAGTACGATCGAGTGTACAGTGAA
MT345810.1 Severe acute respiratory syndrome coronavirus 2 isolate SARS-CoV-2/human/USA/WA-UW-2086/2020 C	TTCACCGAGGCCACGCGGAGTACGATCGAGTGTACAGTGAA
MT345811.1 Severe acute respiratory syndrome coronavirus 2 isolate SARS-CoV-2/human/USA/WA-UW-2108/2020, c	TTCACCGAGGCCACGCGGAGTACGATCGAGTGTACAGTGAA
MT345813.1 Severe acute respiratory syndrome coronavirus 2 isolate SARS-CoV-2/human/USA/WA-UW-2115/2020 C	TTCACCGAGGCCACGCGGAGTACGATCGAGTGTACAGTGAA
MT345814.1 Severe acute respiratory syndrome coronavirus 2 isolate SARS-CoV-2/human/USA/WA-UW-2104/2020, c	TTCACCGAGGCCACGCGGAGTACGATCGAGTGTACAGTGAA
MT345815.1 Severe acute respiratory syndrome coronavirus 2 isolate SARS-CoV-2/human/USA/WA-UW-2137/2020, c	TTCACCGAGGCCACGCGGAGTACGATCGAGTGTACAGTGAA

MT345816.1 Severe acute respiratory syndrome coronavirus 2 isolate SARS-CoV-2/human/USA/UNKNOWN-UW-2139	TTCACCGAGGCCACGCGGAGTACGATCGAGTGTACAGTGAA
MT345819.1 Severe acute respiratory syndrome coronavirus 2 isolate SARS-CoV-2/human/USA/WA-UW-2112/2020, c	TTCACCGAGGCCACGCGGAGTACGATCGAGTGTACAGTGAA
MT345820.1 Severe acute respiratory syndrome coronavirus 2 isolate SARS-CoV-2/human/USA/WA-UW-2129/2020 O	TTCACCGAGGCCACGCGGAGTACGATCGAGTGTACAGTGAA
MT345821.1 Severe acute respiratory syndrome coronavirus 2 isolate SARS-CoV-2/human/USA/WA-UW-2100/2020, c	TTCACCGAGGCCACGCGGAGTACGATCGAGTGTACAGTGAA
MT345822.1 Severe acute respiratory syndrome coronavirus 2 isolate SARS-CoV-2/human/USA/WA-UW-2141/2020, c	TTCACCGAGGCCACGCGGAGTACGATCGAGTGTACAGTGAA
MT345823.1 Severe acute respiratory syndrome coronavirus 2 isolate SARS-CoV-2/human/USA/WA-UW-2164/2020, c	TTCACCGAGGCCACGCGGAGTACGATCGAGTGTACAGTGAA
MT345826.1 Severe acute respiratory syndrome coronavirus 2 isolate SARS-CoV-2/human/USA/WA-UW-2144/2020, c	TTCACCGAGGCCACGCGGAGTACGATCGAGTGTACAGTGAA
MT345827.1 Severe acute respiratory syndrome coronavirus 2 isolate SARS-CoV-2/human/USA/WA-UW-2146/2020, c	TTCACCGAGGCCACGCGGAGTACGATCGAGTGTACAGTGAA
MT345828.1 Severe acute respiratory syndrome coronavirus 2 isolate SARS-CoV-2/human/USA/ID-UW-2160/2020, co	TTCACCGAGGCCACGCGGAGTACGATCGAGTGTACAGTGAA
MT345829.1 Severe acute respiratory syndrome coronavirus 2 isolate SARS-CoV-2/human/USA/WA-UW-2142/2020, c	TTCACCGAGGCCACGCGGAGTACGATCGAGTGTACAGTGAA
MT345830.1 Severe acute respiratory syndrome coronavirus 2 isolate SARS-CoV-2/human/USA/WA-UW-2149/2020, c	TTCACCGAGGCCACGCGGAGTACGATCGAGTGTACAGTGAA
MT345831.1 Severe acute respiratory syndrome coronavirus 2 isolate SARS-CoV-2/human/USA/ID-UW-2159/2020 OR	TTCACCGAGGCCACGCGGAGTACGATCGAGTGTACAGTGAA
MT345833.1 Severe acute respiratory syndrome coronavirus 2 isolate SARS-CoV-2/human/USA/WA-UW-2203/2020, c	TTCACCGAGGCCACGCGGAGTACGATCGAGTGTACAGTGAA
MT345834.1 Severe acute respiratory syndrome coronavirus 2 isolate SARS-CoV-2/human/USA/WA-UW-2240/2020, c	TTCACCGAGGCCACGCGGAGTACGATCGAGTGTACAGTGAA
MT345836.1 Severe acute respiratory syndrome coronavirus 2 isolate SARS-CoV-2/human/USA/WA-UW-2228/2020, c	TTCACCGAGGCCACGCGGAGTACGATCGAGTGTACAGTGAA
MT345840.1 Severe acute respiratory syndrome coronavirus 2 isolate SARS-CoV-2/human/USA/WA-UW-2249/2020, c	TTCACCGAGGCCACGCGGAGTACGATCGAGTGTACAGTGAA
MT345841.1 Severe acute respiratory syndrome coronavirus 2 isolate SARS-CoV-2/human/USA/WA-UW-2247/2020, c	TTCACCGAGGCCACGCGGAGTACGATCGAGTGTACAGTGAA
MT345843.1 Severe acute respiratory syndrome coronavirus 2 isolate SARS-CoV-2/human/USA/WA-UW-2179/2020, c	TTCACCGAGGCCACGCGGAGTACGATCGAGTGTACAGTGAA
MT345844.1 Severe acute respiratory syndrome coronavirus 2 isolate SARS-CoV-2/human/USA/WA-UW-2180/2020, c	TTCACCGAGGCCACGCGGAGTACGATCGAGTGTACAGTGAA
MT345845.1 Severe acute respiratory syndrome coronavirus 2 isolate SARS-CoV-2/human/USA/WA-UW-2177/2020 O	TTCACCGAGGCCACGCGGAGTACGATCGAGTGTACAGTGAA
MT345848.1 Severe acute respiratory syndrome coronavirus 2 isolate SARS-CoV-2/human/USA/WA-UW-2238/2020, c	TTCACCGAGGCCACGCGGAGTACGATCGAGTGTACAGTGAA
MT345850.1 Severe acute respiratory syndrome coronavirus 2 isolate SARS-CoV-2/human/USA/WA-UW-2193/2020, c	TTCACCGAGGCCACGCGGAGTACGATCGAGTGTACAGTGAA
MT345852.1 Severe acute respiratory syndrome coronavirus 2 isolate SARS-CoV-2/human/USA/WA-UW-2192/2020, c	TTCACCGAGGCCACGCGGAGTACGATCGAGTGTACAGTGAA
MT345853.1 Severe acute respiratory syndrome coronavirus 2 isolate SARS-CoV-2/human/USA/WA-UW-2230/2020, c	TTCACCGAGGCCACGCGGAGTACGATCGAGTGTACAGTGAA
MT345854.1 Severe acute respiratory syndrome coronavirus 2 isolate SARS-CoV-2/human/USA/WA-UW-2219/2020, c	TTCACCGAGGCCACGCGGAGTACGATCGAGTGTACAGTGAA
MT345856.1 Severe acute respiratory syndrome coronavirus 2 isolate SARS-CoV-2/human/USA/WA-UW-2218/2020, c	TTCACCGAGGCCACGCGGAGTACGATCGAGTGTACAGTGAA
MT345858.1 Severe acute respiratory syndrome coronavirus 2 isolate SARS-CoV-2/human/USA/ID-UW-2174/2020, co	TTCACCGAGGCCACGCGGAGTACGATCGAGTGTACAGTGAA
MT345859.1 Severe acute respiratory syndrome coronavirus 2 isolate SARS-CoV-2/human/USA/WA-UW-2215/2020, c	TTCACCGAGGCCACGCGGAGTACGATCGAGTGTACAGTGAA
MT345864.1 Severe acute respiratory syndrome coronavirus 2 isolate SARS-CoV-2/human/USA/ID-UW-2195/2020 OR	TTCACCGAGGCCACGCGGAGTACGATCGAGTGTACAGTGAA
MT345865.1 Severe acute respiratory syndrome coronavirus 2 isolate SARS-CoV-2/human/USA/WA-UW-2208/2020, c	TTCACCGAGGCCACGCGGAGTACGATCGAGTGTACAGTGAA
MT345866.1 Severe acute respiratory syndrome coronavirus 2 isolate SARS-CoV-2/human/USA/ID-UW-2255/2020, co	TTCACCGAGGCCACGCGGAGTACGATCGAGTGTACAGTGAA
MT345868.1 Severe acute respiratory syndrome coronavirus 2 isolate SARS-CoV-2/human/USA/CT-UW-4344/2020, co	TTCACCGAGGCCACGCGGAGTACGATCGAGTGTACAGTGAA
MT345869.1 Severe acute respiratory syndrome coronavirus 2 isolate SARS-CoV-2/human/USA/CT-UW-4346/2020, co	TTCACCGAGGCCACGCGGAGTACGATCGAGTGTACAGTGAA

MT345870.1 Severe acute respiratory syndrome coronavirus 2 isolate SARS-CoV-2/human/USA/CT-UW-4347/2020, c	TTCACCGAGGCCACGCGGAGTACGATCGAGTGTACAGTGAA
MT345871.1 Severe acute respiratory syndrome coronavirus 2 isolate SARS-CoV-2/human/USA/ID-UW-4355/2020, c	TTCACCGAGGCCACGCGGAGTACGATCGAGTGTACAGTGAA
MT345872.1 Severe acute respiratory syndrome coronavirus 2 isolate SARS-CoV-2/human/USA/ID-UW-4357/2020, c	TTCACCGAGGCCACGCGGAGTACGATCGAGTGTACAGTGAA
MT345873.1 Severe acute respiratory syndrome coronavirus 2 isolate SARS-CoV-2/human/USA/CT-UW-4366/2020, c	TTCACCGAGGCCACGCGGAGTACGATCGAGTGTACAGTGAA
MT345874.1 Severe acute respiratory syndrome coronavirus 2 isolate SARS-CoV-2/human/USA/CT-UW-4372/2020, c	TTCACCGAGGCCACGCGGAGTACGATCGAGTGTACAGTGAA
MT345875.1 Severe acute respiratory syndrome coronavirus 2 isolate SARS-CoV-2/human/USA/ID-UW-4375/2020, c	TTCACCGAGGCCACGCGGAGTACGATCGAGTGTACAGTGAA
MT345877.1 Severe acute respiratory syndrome coronavirus 2 isolate SARS-CoV-2/human/USA/ID-UW-4380/2020, c	TTCACCGAGGCCACGCGGAGTACGATCGAGTGTACAGTGAA
MT345879.1 Severe acute respiratory syndrome coronavirus 2 isolate SARS-CoV-2/human/USA/ID-UW-4396/2020, c	TTCACCGAGGCCACGCGGAGTACGATCGAGTGTACAGTGAA
MT345881.1 Severe acute respiratory syndrome coronavirus 2 isolate SARS-CoV-2/human/USA/WA-UW-4404/2020,	TTCACCGAGGCCACGCGGAGTACGATCGAGTGTACAGTGAA
MT345883.1 Severe acute respiratory syndrome coronavirus 2 isolate SARS-CoV-2/human/USA/WA-UW-4407/2020,	TTCACCGAGGCCACGCGGAGTACGATCGAGTGTACAGTGAA
MT345884.1 Severe acute respiratory syndrome coronavirus 2 isolate SARS-CoV-2/human/USA/ID-UW-4424/2020, c	TTCACCGAGGCCACGCGGAGTACGATCGAGTGTACAGTGAA
MT345885.1 Severe acute respiratory syndrome coronavirus 2 isolate SARS-CoV-2/human/USA/ID-UW-4427/2020, c	TTCACCGAGGCCACGCGGAGTACGATCGAGTGTACAGTGAA
MT345886.1 Severe acute respiratory syndrome coronavirus 2 isolate SARS-CoV-2/human/USA/ID-UW-4433/2020, c	TTCACCGAGGCCACGCGGAGTACGATCGAGTGTACAGTGAA
MT345887.1 Severe acute respiratory syndrome coronavirus 2 isolate SARS-CoV-2/human/USA/ID-UW-4448/2020, c	TTCACCGAGGCCACGCGGAGTACGATCGAGTGTACAGTGAA
MT345888.1 Severe acute respiratory syndrome coronavirus 2 isolate SARS-CoV-2/human/USA/ID-UW-4461/2020, c	TTCACCGAGGCCACGCGGAGTACGATCGAGTGTACAGTGAA
MT350236.1 Severe acute respiratory syndrome coronavirus 2 isolate SARS-CoV-2/human/USA/VA-DCLS-0041/2020,	TTCACCGAGGCCACGCGGAGTACGATCGAGTGTACAGTGAA
MT350238.1 Severe acute respiratory syndrome coronavirus 2 isolate SARS-CoV-2/human/USA/VA-DCLS-0048/2020,	TTCACCGAGGCCACGCGGAGTACGATCGAGTGTACAGTGAA
MT350239.1 Severe acute respiratory syndrome coronavirus 2 isolate SARS-CoV-2/human/USA/VA-DCLS-0049/2020,	TTCACCGAGGCCACGCGGAGTACGATCGAGTGTACAGTGAA
MT350241.1 Severe acute respiratory syndrome coronavirus 2 isolate SARS-CoV-2/human/USA/VA-DCLS-0051/2020,	TTCACCGAGGCCACGCGGAGTACGATCGAGTGTACAGTGAA
MT350243.1 Severe acute respiratory syndrome coronavirus 2 isolate SARS-CoV-2/human/USA/VA-DCLS-0053/2020,	TTCACCGAGGCCACGCGGAGTACGATCGAGTGTACAGTGAA
MT350244.1 Severe acute respiratory syndrome coronavirus 2 isolate SARS-CoV-2/human/USA/VA-DCLS-0054/2020,	TTCACCGAGGCCACGCGGAGTACGATCGAGTGTACAGTGAA
MT350246.1 Severe acute respiratory syndrome coronavirus 2 isolate SARS-CoV-2/human/USA/VA-DCLS-0056/2020,	TTCACCGAGGCCACGCGGAGTACGATCGAGTGTACAGTGAA
MT350250.1 Severe acute respiratory syndrome coronavirus 2 isolate SARS-CoV-2/human/USA/VA-DCLS-0060/2020,	TTCACCGAGGCCACGCGGAGTACGATCGAGTGTACAGTGAA
MT350255.1 Severe acute respiratory syndrome coronavirus 2 isolate SARS-CoV-2/human/USA/VA-DCLS-0066/2020,	TTCACCGAGGCCACGCGGAGTACGATCGAGTGTACAGTGAA
MT350256.1 Severe acute respiratory syndrome coronavirus 2 isolate SARS-CoV-2/human/USA/VA-DCLS-0067/2020,	TTCACCGAGGCCACGCGGAGTACGATCGAGTGTACAGTGAA
MT350263.1 Severe acute respiratory syndrome coronavirus 2 isolate SARS-CoV-2/human/USA/USA-WA_0480/2020,	TTCACCGAGGCCACGCGGAGTACGATCGAGTGTACAGTGAA
MT350264.1 Severe acute respiratory syndrome coronavirus 2 isolate SARS-CoV-2/human/USA/USA-WA_0428/2020,	TTCACCGAGGCCACGCGGAGTACGATCGAGTGTACAGTGAA
MT350265.1 Severe acute respiratory syndrome coronavirus 2 isolate SARS-CoV-2/human/USA/USA-WA_0427/2020,	TTCACCGAGGCCACGCGGAGTACGATCGAGTGTACAGTGAA
MT350266.1 Severe acute respiratory syndrome coronavirus 2 isolate SARS-CoV-2/human/USA/USA-WA_0434/2020,	TTCACCGAGGCCACGCGGAGTACGATCGAGTGTACAGTGAA
MT350267.1 Severe acute respiratory syndrome coronavirus 2 isolate SARS-CoV-2/human/USA/USA-WA_0442/2020,	TTCACCGAGGCCACGCGGAGTACGATCGAGTGTACAGTGAA
MT350268.1 Severe acute respiratory syndrome coronavirus 2 isolate SARS-CoV-2/human/USA/USA-WA_0418/2020,	TTCACCGAGGCCACGCGGAGTACGATCGAGTGTACAGTGAA
MT350269.1 Severe acute respiratory syndrome coronavirus 2 isolate SARS-CoV-2/human/USA/USA-WA_0797/2020,	TTCACCGAGGCCACGCGGAGTACGATCGAGTGTACAGTGAA
MT350270.1 Severe acute respiratory syndrome coronavirus 2 isolate SARS-CoV-2/human/USA/USA-WA_0432/2020,	TTCACCGAGGCCACGCGGAGTACGATCGAGTGTACAGTGAA

MT350271.1 Severe acute respiratory syndrome coronavirus 2 isolate SARS-CoV-2/human/USA/USA-WA_0457/2020,	TTCACCGAGGCCACGCGGAGTACGATCGAGTGTACAGTGAA
MT350272.1 Severe acute respiratory syndrome coronavirus 2 isolate SARS-CoV-2/human/USA/USA-WA_0448/2020,	TTCACCGAGGCCACGCGGAGTACGATCGAGTGTACAGTGAA
MT350273.1 Severe acute respiratory syndrome coronavirus 2 isolate SARS-CoV-2/human/USA/USA-WA_0478/2020,	TTCACCGAGGCCACGCGGAGTACGATCGAGTGTACAGTGAA
MT350274.1 Severe acute respiratory syndrome coronavirus 2 isolate SARS-CoV-2/human/USA/USA-WA_0422/2020,	TTCACCGAGGCCACGCGGAGTACGATCGAGTGTACAGTGAA
MT350275.1 Severe acute respiratory syndrome coronavirus 2 isolate SARS-CoV-2/human/USA/USA-WA_0484/2020,	TTCACCGAGGCCACGCGGAGTACGATCGAGTGTACAGTGAA
MT350276.1 Severe acute respiratory syndrome coronavirus 2 isolate SARS-CoV-2/human/USA/USA-WA_0429/2020,	TTCACCGAGGCCACGCGGAGTACGATCGAGTGTACAGTGAA
MT350277.1 Severe acute respiratory syndrome coronavirus 2 isolate SARS-CoV-2/human/USA/USA-WA_0441/2020,	TTCACCGAGGCCACGCGGAGTACGATCGAGTGTACAGTGAA
MT350278.1 Severe acute respiratory syndrome coronavirus 2 isolate SARS-CoV-2/human/USA/USA-WA_0440/2020,	TTCACCGAGGCCACGCGGAGTACGATCGAGTGTACAGTGAA
MT350279.1 Severe acute respiratory syndrome coronavirus 2 isolate SARS-CoV-2/human/USA/USA-WA_0711/2020,	TTCACCGAGGCCACGCGGAGTACGATCGAGTGTACAGTGAA
MT350280.1 Severe acute respiratory syndrome coronavirus 2 isolate SARS-CoV-2/human/USA/USA-WA_0447/2020,	TTCACCGAGGCCACGCGGAGTACGATCGAGTGTACAGTGAA
MT350282.1 Severe acute respiratory syndrome coronavirus 2 isolate SARS-CoV-2/human/BRA/SP02cc/2020, comple	TTCACCGAGGCCACGCGGAGTACGATCGAGTGTACAGTGAA
MT358401.1 Severe acute respiratory syndrome coronavirus 2 isolate SARS-CoV-2/human/USA/LA096/2020, complet	TTCACCGAGGCCACGCGGAGTACGATCGAGTGTACAGTGAA
MT358402.1 Severe acute respiratory syndrome coronavirus 2 isolate SARS-CoV-2/human/USA/LA097/2020, complet	TTCACCGAGGCCACGCGGAGTACGATCGAGTGTACAGTGAA
MT358637.1 Severe acute respiratory syndrome coronavirus 2 isolate SARS-CoV-2/human/IND/GBRC1/2020, complet	TTCACCGAGGCCACGCGGAGTACGATCGAGTGTACAGTGAA
MT358638.1 Severe acute respiratory syndrome coronavirus 2 isolate SARS-CoV-2/human/DEU/FFM1/2020, complet	TTCACCGAGGCCACGCGGAGTACGATCGAGTGTACAGTGAA
MT358639.1 Severe acute respiratory syndrome coronavirus 2 isolate SARS-CoV-2/human/DEU/FFM3/2020, complet	TTCACCGAGGCCACGCGGAGTACGATCGAGTGTACAGTGAA
MT358641.1 Severe acute respiratory syndrome coronavirus 2 isolate SARS-CoV-2/human/DEU/FFM5/2020, complet	TTCACCGAGGCCACGCGGAGTACGATCGAGTGTACAGTGAA
MT358643.1 Severe acute respiratory syndrome coronavirus 2 isolate SARS-CoV-2/human/DEU/FFM7/2020, complet	TTCACCGAGGCCACGCGGAGTACGATCGAGTGTACAGTGAA
MT358644.1 Severe acute respiratory syndrome coronavirus 2 isolate SARS-CoV-2/human/USA/WA-UW-4180/2020, c	TTCACCGAGGCCACGCGGAGTACGATCGAGTGTACAGTGAA
MT358645.1 Severe acute respiratory syndrome coronavirus 2 isolate SARS-CoV-2/human/USA/WA-UW-4188/2020, c	TTCACCGAGGCCACGCGGAGTACGATCGAGTGTACAGTGAA
MT358646.1 Severe acute respiratory syndrome coronavirus 2 isolate SARS-CoV-2/human/USA/WA-UW-4197/2020, c	TTCACCGAGGCCACGCGGAGTACGATCGAGTGTACAGTGAA
MT358647.1 Severe acute respiratory syndrome coronavirus 2 isolate SARS-CoV-2/human/USA/WA-UW-4213/2020, c	TTCACCGAGGCCACGCGGAGTACGATCGAGTGTACAGTGAA
MT358648.1 Severe acute respiratory syndrome coronavirus 2 isolate SARS-CoV-2/human/USA/WA-UW-4217/2020, c	TTCACCGAGGCCACGCGGAGTACGATCGAGTGTACAGTGAA
MT358649.1 Severe acute respiratory syndrome coronavirus 2 isolate SARS-CoV-2/human/USA/WA-UW-4227/2020, c	TTCACCGAGGCCACGCGGAGTACGATCGAGTGTACAGTGAA
MT358651.1 Severe acute respiratory syndrome coronavirus 2 isolate SARS-CoV-2/human/USA/CT-UW-4238/2020, c	TTCACCGAGGCCACGCGGAGTACGATCGAGTGTACAGTGAA
MT358652.1 Severe acute respiratory syndrome coronavirus 2 isolate SARS-CoV-2/human/USA/ID-UW-4239/2020, c	TTCACCGAGGCCACGCGGAGTACGATCGAGTGTACAGTGAA
MT358653.1 Severe acute respiratory syndrome coronavirus 2 isolate SARS-CoV-2/human/USA/CT-UW-4244/2020, c	TTCACCGAGGCCACGCGGAGTACGATCGAGTGTACAGTGAA
MT358656.1 Severe acute respiratory syndrome coronavirus 2 isolate SARS-CoV-2/human/USA/WA-UW-4281/2020, c	TTCACCGAGGCCACGCGGAGTACGATCGAGTGTACAGTGAA
MT358657.1 Severe acute respiratory syndrome coronavirus 2 isolate SARS-CoV-2/human/USA/WA-UW-4289/2020, c	TTCACCGAGGCCACGCGGAGTACGATCGAGTGTACAGTGAA
MT358658.1 Severe acute respiratory syndrome coronavirus 2 isolate SARS-CoV-2/human/USA/WA-UW-4290/2020, c	TTCACCGAGGCCACGCGGAGTACGATCGAGTGTACAGTGAA
MT358659.1 Severe acute respiratory syndrome coronavirus 2 isolate SARS-CoV-2/human/USA/WA-UW-4291/2020, c	TTCACCGAGGCCACGCGGAGTACGATCGAGTGTACAGTGAA
MT358660.1 Severe acute respiratory syndrome coronavirus 2 isolate SARS-CoV-2/human/USA/WA-UW-4292/2020, c	TTCACCGAGGCCACGCGGAGTACGATCGAGTGTACAGTGAA
MT358661.1 Severe acute respiratory syndrome coronavirus 2 isolate SARS-CoV-2/human/USA/OR-UW-4300/2020, c	TTCACCGAGGCCACGCGGAGTACGATCGAGTGTACAGTGAA

MT358662.1 Severe acute respiratory syndrome coronavirus 2 isolate SARS-CoV-2/human/USA/WA-UW-4315/2020,	TTCACCGAGGCCACGCGGAGTACGATCGAGTGTACAGTGAA
MT358663.1 Severe acute respiratory syndrome coronavirus 2 isolate SARS-CoV-2/human/USA/WA-UW-4316/2020,	TTCACCGAGGCCACGCGGAGTACGATCGAGTGTACAGTGAA
MT358664.1 Severe acute respiratory syndrome coronavirus 2 isolate SARS-CoV-2/human/USA/WA-UW-4318/2020,	TTCACCGAGGCCACGCGGAGTACGATCGAGTGTACAGTGAA
MT358665.1 Severe acute respiratory syndrome coronavirus 2 isolate SARS-CoV-2/human/USA/WA-UW-4321/2020,	TTCACCGAGGCCACGCGGAGTACGATCGAGTGTACAGTGAA
MT358666.1 Severe acute respiratory syndrome coronavirus 2 isolate SARS-CoV-2/human/USA/WA-UW-4322/2020,	TTCACCGAGGCCACGCGGAGTACGATCGAGTGTACAGTGAA
MT358667.1 Severe acute respiratory syndrome coronavirus 2 isolate SARS-CoV-2/human/USA/WA-UW-4324/2020,	TTCACCGAGGCCACGCGGAGTACGATCGAGTGTACAGTGAA
MT358669.1 Severe acute respiratory syndrome coronavirus 2 isolate SARS-CoV-2/human/USA/WA-UW-4329/2020,	TTCACCGAGGCCACGCGGAGTACGATCGAGTGTACAGTGAA
MT358671.1 Severe acute respiratory syndrome coronavirus 2 isolate SARS-CoV-2/human/USA/WA-UW-4335/2020,	TTCACCGAGGCCACGCGGAGTACGATCGAGTGTACAGTGAA
MT358672.1 Severe acute respiratory syndrome coronavirus 2 isolate SARS-CoV-2/human/USA/WA-UW-4340/2020,	TTCACCGAGGCCACGCGGAGTACGATCGAGTGTACAGTGAA
MT358674.1 Severe acute respiratory syndrome coronavirus 2 isolate SARS-CoV-2/human/USA/WA-UW-3809/2020,	TTCACCGAGGCCACGCGGAGTACGATCGAGTGTACAGTGAA
MT358675.1 Severe acute respiratory syndrome coronavirus 2 isolate SARS-CoV-2/human/USA/WA-UW-3810/2020,	TTCACCGAGGCCACGCGGAGTACGATCGAGTGTACAGTGAA
MT358676.1 Severe acute respiratory syndrome coronavirus 2 isolate SARS-CoV-2/human/USA/WA-UW-3812/2020 C	TTCACCGAGGCCACGCGGAGTACGATCGAGTGTACAGTGAA
MT358677.1 Severe acute respiratory syndrome coronavirus 2 isolate SARS-CoV-2/human/USA/WA-UW-3814/2020,	TTCACCGAGGCCACGCGGAGTACGATCGAGTGTACAGTGAA
MT358678.1 Severe acute respiratory syndrome coronavirus 2 isolate SARS-CoV-2/human/USA/WA-UW-3815/2020,	TTCACCGAGGCCACGCGGAGTACGATCGAGTGTACAGTGAA
MT358679.1 Severe acute respiratory syndrome coronavirus 2 isolate SARS-CoV-2/human/USA/WA-UW-3816/2020,	TTCACCGAGGCCACGCGGAGTACGATCGAGTGTACAGTGAA
MT358680.1 Severe acute respiratory syndrome coronavirus 2 isolate SARS-CoV-2/human/USA/WA-UW-3819/2020,	TTCACCGAGGCCACGCGGAGTACGATCGAGTGTACAGTGAA
MT358681.1 Severe acute respiratory syndrome coronavirus 2 isolate SARS-CoV-2/human/USA/WA-UW-3826/2020,	TTCACCGAGGCCACGCGGAGTACGATCGAGTGTACAGTGAA
MT358682.1 Severe acute respiratory syndrome coronavirus 2 isolate SARS-CoV-2/human/USA/WA-UW-3827/2020,	TTCACCGAGGCCACGCGGAGTACGATCGAGTGTACAGTGAA
MT358683.1 Severe acute respiratory syndrome coronavirus 2 isolate SARS-CoV-2/human/USA/WA-UW-3828/2020,	TTCACCGAGGCCACGCGGAGTACGATCGAGTGTACAGTGAA
MT358684.1 Severe acute respiratory syndrome coronavirus 2 isolate SARS-CoV-2/human/USA/WA-UW-3829/2020,	TTCACCGAGGCCACGCGGAGTACGATCGAGTGTACAGTGAA
MT358686.1 Severe acute respiratory syndrome coronavirus 2 isolate SARS-CoV-2/human/USA/WA-UW-3833/2020 C	TTCACCGAGGCCACGCGGAGTACGATCGAGTGTACAGTGAA
MT358687.1 Severe acute respiratory syndrome coronavirus 2 isolate SARS-CoV-2/human/USA/WA-UW-3834/2020,	TTCACCGAGGCCACGCGGAGTACGATCGAGTGTACAGTGAA
MT358688.1 Severe acute respiratory syndrome coronavirus 2 isolate SARS-CoV-2/human/USA/WA-UW-3838/2020,	TTCACCGAGGCCACGCGGAGTACGATCGAGTGTACAGTGAA
MT358689.1 Severe acute respiratory syndrome coronavirus 2 isolate SARS-CoV-2/human/USA/WA-UW-3839/2020,	TTCACCGAGGCCACGCGGAGTACGATCGAGTGTACAGTGAA
MT358690.1 Severe acute respiratory syndrome coronavirus 2 isolate SARS-CoV-2/human/USA/WA-UW-3841/2020,	TTCACCGAGGCCACGCGGAGTACGATCGAGTGTACAGTGAA
MT358691.1 Severe acute respiratory syndrome coronavirus 2 isolate SARS-CoV-2/human/USA/WA-UW-3843/2020,	TTCACCGAGGCCACGCGGAGTACGATCGAGTGTACAGTGAA
MT358692.1 Severe acute respiratory syndrome coronavirus 2 isolate SARS-CoV-2/human/USA/WA-UW-3844/2020,	TTCACCGAGGCCACGCGGAGTACGATCGAGTGTACAGTGAA
MT358693.1 Severe acute respiratory syndrome coronavirus 2 isolate SARS-CoV-2/human/USA/CT-UW-3845/2020, c	TTCACCGAGGCCACGCGGAGTACGATCGAGTGTACAGTGAA
MT358695.1 Severe acute respiratory syndrome coronavirus 2 isolate SARS-CoV-2/human/USA/WA-UW-3855/2020,	TTCACCGAGGCCACGCGGAGTACGATCGAGTGTACAGTGAA
MT358696.1 Severe acute respiratory syndrome coronavirus 2 isolate SARS-CoV-2/human/USA/WA-UW-3858/2020,	TTCACCGAGGCCACGCGGAGTACGATCGAGTGTACAGTGAA
MT358697.1 Severe acute respiratory syndrome coronavirus 2 isolate SARS-CoV-2/human/USA/WA-UW-3862/2020,	TTCACCGAGGCCACGCGGAGTACGATCGAGTGTACAGTGAA
MT358698.1 Severe acute respiratory syndrome coronavirus 2 isolate SARS-CoV-2/human/USA/WA-UW-3868/2020,	TTCACCGAGGCCACGCGGAGTACGATCGAGTGTACAGTGAA
MT358699.1 Severe acute respiratory syndrome coronavirus 2 isolate SARS-CoV-2/human/USA/WA-UW-3871/2020,	TTCACCGAGGCCACGCGGAGTACGATCGAGTGTACAGTGAA

MT358700.1 Severe acute respiratory syndrome coronavirus 2 isolate SARS-CoV-2/human/USA/WA-UW-3880/2020,	TTCACCGAGGCCACGCGGAGTACGATCGAGTGTACAGTGAA
MT358701.1 Severe acute respiratory syndrome coronavirus 2 isolate SARS-CoV-2/human/USA/WA-UW-3881/2020,	TTCACCGAGGCCACGCGGAGTACGATCGAGTGTACAGTGAA
MT358702.1 Severe acute respiratory syndrome coronavirus 2 isolate SARS-CoV-2/human/USA/WA-UW-3883/2020,	TTCACCGAGGCCACGCGGAGTACGATCGAGTGTACAGTGAA
MT358703.1 Severe acute respiratory syndrome coronavirus 2 isolate SARS-CoV-2/human/USA/ID-UW-3888/2020, co	TTCACCGAGGCCACGCGGAGTACGATCGAGTGTACAGTGAA
MT358704.1 Severe acute respiratory syndrome coronavirus 2 isolate SARS-CoV-2/human/USA/ID-UW-4462/2020 OR	TTCACCGAGGCCACGCGGAGTACGATCGAGTGTACAGTGAA
MT358705.1 Severe acute respiratory syndrome coronavirus 2 isolate SARS-CoV-2/human/USA/ID-UW-4486/2020, co	TTCACCGAGGCCACGCGGAGTACGATCGAGTGTACAGTGAA
MT358706.1 Severe acute respiratory syndrome coronavirus 2 isolate SARS-CoV-2/human/USA/WA-UW-4499/2020,	TTCACCGAGGCCACGCGGAGTACGATCGAGTGTACAGTGAA
MT358707.1 Severe acute respiratory syndrome coronavirus 2 isolate SARS-CoV-2/human/USA/WA-UW-4500/2020,	TTCACCGAGGCCACGCGGAGTACGATCGAGTGTACAGTGAA
MT358708.1 Severe acute respiratory syndrome coronavirus 2 isolate SARS-CoV-2/human/USA/WA-UW-3889/2020,	TTCACCGAGGCCACGCGGAGTACGATCGAGTGTACAGTGAA
MT358709.1 Severe acute respiratory syndrome coronavirus 2 isolate SARS-CoV-2/human/USA/ID-UW-3892/2020, co	TTCACCGAGGCCACGCGGAGTACGATCGAGTGTACAGTGAA
MT358711.1 Severe acute respiratory syndrome coronavirus 2 isolate SARS-CoV-2/human/USA/WA-UW-3900/2020,	TTCACCGAGGCCACGCGGAGTACGATCGAGTGTACAGTGAA
MT358712.1 Severe acute respiratory syndrome coronavirus 2 isolate SARS-CoV-2/human/USA/WA-UW-3903/2020,	TTCACCGAGGCCACGCGGAGTACGATCGAGTGTACAGTGAA
MT358713.1 Severe acute respiratory syndrome coronavirus 2 isolate SARS-CoV-2/human/USA/WA-UW-3908/2020,	TTCACCGAGGCCACGCGGAGTACGATCGAGTGTACAGTGAA
MT358714.1 Severe acute respiratory syndrome coronavirus 2 isolate SARS-CoV-2/human/USA/WA-UW-3911/2020,	TTCACCGAGGCCACGCGGAGTACGATCGAGTGTACAGTGAA
MT358715.1 Severe acute respiratory syndrome coronavirus 2 isolate SARS-CoV-2/human/USA/WA-UW-3918/2020,	TTCACCGAGGCCACGCGGAGTACGATCGAGTGTACAGTGAA
MT358716.1 Severe acute respiratory syndrome coronavirus 2 isolate SARS-CoV-2/human/USA/WA-UW-3923/2020,	TTCACCGAGGCCACGCGGAGTACGATCGAGTGTACAGTGAA
MT358717.1 Severe acute respiratory syndrome coronavirus 2 isolate SARS-CoV-2/human/USA/WA-UW-3932/2020,	TTCACCGAGGCCACGCGGAGTACGATCGAGTGTACAGTGAA
MT358718.1 Severe acute respiratory syndrome coronavirus 2 isolate SARS-CoV-2/human/USA/WA-UW-3935/2020,	TTCACCGAGGCCACGCGGAGTACGATCGAGTGTACAGTGAA
MT358719.1 Severe acute respiratory syndrome coronavirus 2 isolate SARS-CoV-2/human/USA/WA-UW-3939/2020,	TTCACCGAGGCCACGCGGAGTACGATCGAGTGTACAGTGAA
MT358720.1 Severe acute respiratory syndrome coronavirus 2 isolate SARS-CoV-2/human/USA/WA-UW-3940/2020,	TTCACCGAGGCCACGCGGAGTACGATCGAGTGTACAGTGAA
MT358721.1 Severe acute respiratory syndrome coronavirus 2 isolate SARS-CoV-2/human/USA/WA-UW-3941/2020,	TTCACCGAGGCCACGCGGAGTACGATCGAGTGTACAGTGAA
MT358722.1 Severe acute respiratory syndrome coronavirus 2 isolate SARS-CoV-2/human/USA/WA-UW-3942/2020,	TTCACCGAGGCCACGCGGAGTACGATCGAGTGTACAGTGAA
MT358723.1 Severe acute respiratory syndrome coronavirus 2 isolate SARS-CoV-2/human/USA/WA-UW-3946/2020,	TTCACCGAGGCCACGCGGAGTACGATCGAGTGTACAGTGAA
MT358724.1 Severe acute respiratory syndrome coronavirus 2 isolate SARS-CoV-2/human/USA/WA-UW-3950/2020,	TTCACCGAGGCCACGCGGAGTACGATCGAGTGTACAGTGAA
MT358725.1 Severe acute respiratory syndrome coronavirus 2 isolate SARS-CoV-2/human/USA/WA-UW-3954/2020,	TTCACCGAGGCCACGCGGAGTACGATCGAGTGTACAGTGAA
MT358727.1 Severe acute respiratory syndrome coronavirus 2 isolate SARS-CoV-2/human/USA/WA-UW-3963/2020 C	TTCACCGAGGCCACGCGGAGTACGATCGAGTGTACAGTGAA
MT358728.1 Severe acute respiratory syndrome coronavirus 2 isolate SARS-CoV-2/human/USA/WA-UW-3964/2020,	TTCACCGAGGCCACGCGGAGTACGATCGAGTGTACAGTGAA
MT358729.1 Severe acute respiratory syndrome coronavirus 2 isolate SARS-CoV-2/human/USA/WA-UW-3969/2020,	TTCACCGAGGCCACGCGGAGTACGATCGAGTGTACAGTGAA
MT358730.1 Severe acute respiratory syndrome coronavirus 2 isolate SARS-CoV-2/human/USA/WA-UW-3983/2020,	TTCACCGAGGCCACGCGGAGTACGATCGAGTGTACAGTGAA
MT358732.1 Severe acute respiratory syndrome coronavirus 2 isolate SARS-CoV-2/human/USA/WA-UW-3985/2020,	TTCACCGAGGCCACGCGGAGTACGATCGAGTGTACAGTGAA
MT358733.1 Severe acute respiratory syndrome coronavirus 2 isolate SARS-CoV-2/human/USA/WA-UW-3986/2020,	TTCACCGAGGCCACGCGGAGTACGATCGAGTGTACAGTGAA
MT358734.1 Severe acute respiratory syndrome coronavirus 2 isolate SARS-CoV-2/human/USA/WA-UW-3988/2020,	TTCACCGAGGCCACGCGGAGTACGATCGAGTGTACAGTGAA
MT358736.1 Severe acute respiratory syndrome coronavirus 2 isolate SARS-CoV-2/human/USA/WA-UW-3990/2020,	TTCACCGAGGCCACGCGGAGTACGATCGAGTGTACAGTGAA

MT358737.1 Severe acute respiratory syndrome coronavirus 2 isolate SARS-CoV-2/human/USA/WA-UW-3994/2020, c	TTCACCGAGGCCACGCGGAGTACGATCGAGTGTACAGTGAA
MT358739.1 Severe acute respiratory syndrome coronavirus 2 isolate SARS-CoV-2/human/USA/WA-UW-3997/2020, c	TTCACCGAGGCCACGCGGAGTACGATCGAGTGTACAGTGAA
MT358740.1 Severe acute respiratory syndrome coronavirus 2 isolate SARS-CoV-2/human/USA/WA-UW-4035/2020, c	TTCACCGAGGCCACGCGGAGTACGATCGAGTGTACAGTGAA
MT358741.1 Severe acute respiratory syndrome coronavirus 2 isolate SARS-CoV-2/human/USA/WA-UW-4038/2020, c	TTCACCGAGGCCACGCGGAGTACGATCGAGTGTACAGTGAA
MT358742.1 Severe acute respiratory syndrome coronavirus 2 isolate SARS-CoV-2/human/USA/WA-UW-4039/2020, c	TTCACCGAGGCCACGCGGAGTACGATCGAGTGTACAGTGAA
MT358743.1 Severe acute respiratory syndrome coronavirus 2 isolate SARS-CoV-2/human/USA/WA-UW-4068/2020, c	TTCACCGAGGCCACGCGGAGTACGATCGAGTGTACAGTGAA
MT358744.1 Severe acute respiratory syndrome coronavirus 2 isolate SARS-CoV-2/human/USA/WA-UW-4082/2020, c	TTCACCGAGGCCACGCGGAGTACGATCGAGTGTACAGTGAA
MT358745.1 Severe acute respiratory syndrome coronavirus 2 isolate SARS-CoV-2/human/USA/ID-UW-4100/2020, co	TTCACCGAGGCCACGCGGAGTACGATCGAGTGTACAGTGAA
MT358746.1 Severe acute respiratory syndrome coronavirus 2 isolate SARS-CoV-2/human/USA/WA-UW-4105/2020, c	TTCACCGAGGCCACGCGGAGTACGATCGAGTGTACAGTGAA
MT358747.1 Severe acute respiratory syndrome coronavirus 2 isolate SARS-CoV-2/human/USA/WA-UW-4109/2020, c	TTCACCGAGGCCACGCGGAGTACGATCGAGTGTACAGTGAA
MT358748.1 Severe acute respiratory syndrome coronavirus 2 isolate SARS-CoV-2/human/USA/WA-UW-4111/2020, c	TTCACCGAGGCCACGCGGAGTACGATCGAGTGTACAGTGAA
MT359865.1 Severe acute respiratory syndrome coronavirus 2 isolate SARS-CoV-2/human/ESP/VH000001133/2020, c	TTCACCGAGGCCACGCGGAGTACGATCGAGTGTACAGTGAA
MT359866.1 Severe acute respiratory syndrome coronavirus 2 isolate SARS-CoV-2/human/ESP/VH198152683/2020, c	TTCACCGAGGCCACGCGGAGTACGATCGAGTGTACAGTGAA
MT365029.1 Severe acute respiratory syndrome coronavirus 2 isolate SARS-CoV-2/human/HKG/HKU-911a/2020, com	TTCACCGAGGCCACGCGGAGTACGATCGAGTGTACAGTGAA
MT365030.1 Severe acute respiratory syndrome coronavirus 2 isolate SARS-CoV-2/human/HKG/HKU-913a/2020, com	TTCACCGAGGCCACGCGGAGTACGATCGAGTGTACAGTGAA
MT365031.1 Severe acute respiratory syndrome coronavirus 2 isolate SARS-CoV-2/human/HKG/HKU-915a/2020, com	TTCACCGAGGCCACGCGGAGTACGATCGAGTGTACAGTGAA
MT365032.1 Severe acute respiratory syndrome coronavirus 2 isolate SARS-CoV-2/human/HKG/HKU-904a/2020, com	TTCACCGAGGCCACGCGGAGTACGATCGAGTGTACAGTGAA
MT365033.1 Severe acute respiratory syndrome coronavirus 2 isolate SARS-CoV-2/tiger/NY/040420/2020, complete g	TTCACCGAGGCCACGCGGAGTACGATCGAGTGTACAGTGAA
MT370831.1 Severe acute respiratory syndrome coronavirus 2 isolate SARS-CoV-2/human/USA/NY-PV08464/2020, co	TTCACCGAGGCCACGCGGAGTACGATCGAGTGTACAGTGAA
MT370832.1 Severe acute respiratory syndrome coronavirus 2 isolate SARS-CoV-2/human/USA/NY-PV08139/2020 OF	TTCACCGAGGCCACGCGGAGTACGATCGAGTGTACAGTGAA
MT370833.1 Severe acute respiratory syndrome coronavirus 2 isolate SARS-CoV-2/human/USA/NY-PV08432/2020, co	TTCACCGAGGCCACGCGGAGTACGATCGAGTGTACAGTGAA
MT370834.1 Severe acute respiratory syndrome coronavirus 2 isolate SARS-CoV-2/human/USA/NY-PV08478/2020, co	TTCACCGAGGCCACGCGGAGTACGATCGAGTGTACAGTGAA
MT370835.1 Severe acute respiratory syndrome coronavirus 2 isolate SARS-CoV-2/human/USA/NY-PV08426/2020, co	TTCACCGAGGCCACGCGGAGTACGATCGAGTGTACAGTGAA
MT370836.1 Severe acute respiratory syndrome coronavirus 2 isolate SARS-CoV-2/human/USA/NY-PV08436/2020, co	TTCACCGAGGCCACGCGGAGTACGATCGAGTGTACAGTGAA
MT370837.1 Severe acute respiratory syndrome coronavirus 2 isolate SARS-CoV-2/human/USA/NY-PV08486/2020, co	TTCACCGAGGCCACGCGGAGTACGATCGAGTGTACAGTGAA
MT370838.1 Severe acute respiratory syndrome coronavirus 2 isolate SARS-CoV-2/human/USA/NY-PV08456/2020, co	TTCACCGAGGCCACGCGGAGTACGATCGAGTGTACAGTGAA
MT370839.1 Severe acute respiratory syndrome coronavirus 2 isolate SARS-CoV-2/human/USA/NY-PV08124/2020, co	TTCACCGAGGCCACGCGGAGTACGATCGAGTGTACAGTGAA
MT370840.1 Severe acute respiratory syndrome coronavirus 2 isolate SARS-CoV-2/human/USA/NY-PV08140/2020 OF	TTCACCGAGGCCACGCGGAGTACGATCGAGTGTACAGTGAA
MT370841.1 Severe acute respiratory syndrome coronavirus 2 isolate SARS-CoV-2/human/USA/NY-PV08490/2020, co	TTCACCGAGGCCACGCGGAGTACGATCGAGTGTACAGTGAA
MT370842.1 Severe acute respiratory syndrome coronavirus 2 isolate SARS-CoV-2/human/USA/NY2-PV08100/2020, c	TTCACCGAGGCCACGCGGAGTACGATCGAGTGTACAGTGAA
MT370843.1 Severe acute respiratory syndrome coronavirus 2 isolate SARS-CoV-2/human/USA/NY-PV08121/2020, co	TTCACCGAGGCCACGCGGAGTACGATCGAGTGTACAGTGAA
MT370844.1 Severe acute respiratory syndrome coronavirus 2 isolate SARS-CoV-2/human/USA/NY-PV08463/2020, co	TTCACCGAGGCCACGCGGAGTACGATCGAGTGTACAGTGAA
MT370845.1 Severe acute respiratory syndrome coronavirus 2 isolate SARS-CoV-2/human/USA/NY-PV08492/2020, co	TTCACCGAGGCCACGCGGAGTACGATCGAGTGTACAGTGAA

MT370846.1 Severe acute respiratory syndrome coronavirus 2 isolate SARS-CoV-2/human/USA/NY-PV08401/2020, co	TTCACCGAGGCCACGCGGAGTACGATCGAGTGTACAGTGAA
MT370847.1 Severe acute respiratory syndrome coronavirus 2 isolate SARS-CoV-2/human/USA/NY-PV08438/2020 OF	TTCACCGAGGCCACGCGGAGTACGATCGAGTGTACAGTGAA
MT370848.1 Severe acute respiratory syndrome coronavirus 2 isolate SARS-CoV-2/human/USA/NY-PV08441/2020, co	TTCACCGAGGCCACGCGGAGTACGATCGAGTGTACAGTGAA
MT370849.1 Severe acute respiratory syndrome coronavirus 2 isolate SARS-CoV-2/human/USA/NY-PV08146/2020, co	TTCACCGAGGCCACGCGGAGTACGATCGAGTGTACAGTGAA
MT370850.1 Severe acute respiratory syndrome coronavirus 2 isolate SARS-CoV-2/human/USA/NY-PV08500/2020, co	TTCACCGAGGCCACGCGGAGTACGATCGAGTGTACAGTGAA
MT370851.1 Severe acute respiratory syndrome coronavirus 2 isolate SARS-CoV-2/human/USA/NY-PV09000/2020, co	TTCACCGAGGCCACGCGGAGTACGATCGAGTGTACAGTGAA
MT370852.1 Severe acute respiratory syndrome coronavirus 2 isolate SARS-CoV-2/human/USA/NY-PV08137/2020, co	TTCACCGAGGCCACGCGGAGTACGATCGAGTGTACAGTGAA
MT370853.1 Severe acute respiratory syndrome coronavirus 2 isolate SARS-CoV-2/human/USA/NY-PV08494/2020, co	TTCACCGAGGCCACGCGGAGTACGATCGAGTGTACAGTGAA
MT370854.1 Severe acute respiratory syndrome coronavirus 2 isolate SARS-CoV-2/human/USA/NY-PV08127/2020, co	TTCACCGAGGCCACGCGGAGTACGATCGAGTGTACAGTGAA
MT370855.1 Severe acute respiratory syndrome coronavirus 2 isolate SARS-CoV-2/human/USA/NY-PV08420/2020 OF	TTCACCGAGGCCACGCGGAGTACGATCGAGTGTACAGTGAA
MT370856.1 Severe acute respiratory syndrome coronavirus 2 isolate SARS-CoV-2/human/USA/NY-PV08445/2020, co	TTCACCGAGGCCACGCGGAGTACGATCGAGTGTACAGTGAA
MT370857.1 Severe acute respiratory syndrome coronavirus 2 isolate SARS-CoV-2/human/USA/NY-PV08148/2020, co	TTCACCGAGGCCACACGGAGTACGATCGAGTGTACAGTGAA
MT370858.1 Severe acute respiratory syndrome coronavirus 2 isolate SARS-CoV-2/human/USA/NY-PV08468/2020, co	TTCACCGAGGCCACGCGGAGTACGATCGAGTGTACAGTGAA
MT370859.1 Severe acute respiratory syndrome coronavirus 2 isolate SARS-CoV-2/human/USA/NY-PV08403/2020, co	TTCACCGAGGCCACGCGGAGTACGATCGAGTGTACAGTGAA
MT370860.1 Severe acute respiratory syndrome coronavirus 2 isolate SARS-CoV-2/human/USA/NY-PV08434/2020, co	TTCACCGAGGCCACGCGGAGTACGATCGAGTGTACAGTGAA
MT370861.1 Severe acute respiratory syndrome coronavirus 2 isolate SARS-CoV-2/human/USA/NY-PV08109/2020, co	TTCACCGAGGCCACGCGGAGTACGATCGAGTGTACAGTGAA
MT370862.1 Severe acute respiratory syndrome coronavirus 2 isolate SARS-CoV-2/human/USA/NY-PV08412/2020, co	TTCACCGAGGCCACGCGGAGTACGATCGAGTGTACAGTGAA
MT370863.1 Severe acute respiratory syndrome coronavirus 2 isolate SARS-CoV-2/human/USA/NY-PV08471/2020, co	TTCACCGAGGCCACGCGGAGTACGATCGAGTGTACAGTGAA
MT370864.1 Severe acute respiratory syndrome coronavirus 2 isolate SARS-CoV-2/human/USA/NY-PV08495/2020, co	TTCACCGAGGCCACGCGGAGTACGATCGAGTGTACAGTGAA
MT370865.1 Severe acute respiratory syndrome coronavirus 2 isolate SARS-CoV-2/human/USA/NY-PV08453/2020, co	TTCACCGAGGCCACGCGGAGTACGATCGAGTGTACAGTGAA
MT370866.1 Severe acute respiratory syndrome coronavirus 2 isolate SARS-CoV-2/human/USA/NY-PV08135/2020, co	TTCACCGAGGCCACGCGGAGTACGATCGAGTGTACAGTGAA
MT370867.1 Severe acute respiratory syndrome coronavirus 2 isolate SARS-CoV-2/human/USA/NY-PV08400/2020 OF	TTCACCGAGGCCACGCGGAGTACGATCGAGTGTACAGTGAA
MT370868.1 Severe acute respiratory syndrome coronavirus 2 isolate SARS-CoV-2/human/USA/NY-PV08485/2020, co	TTCACCGAGGCCACGCGGAGTACGATCGAGTGTACAGTGAA
MT370869.1 Severe acute respiratory syndrome coronavirus 2 isolate SARS-CoV-2/human/USA/NY-PV08462/2020, co	TTCACCGAGGCCACGCGGAGTACGATCGAGTGTACAGTGAA
MT370870.1 Severe acute respiratory syndrome coronavirus 2 isolate SARS-CoV-2/human/USA/NY-PV08469/2020, co	TTCACCGAGGCCACGCGGAGTACGATCGAGTGTACAGTGAA
MT370871.1 Severe acute respiratory syndrome coronavirus 2 isolate SARS-CoV-2/human/USA/NY-PV08446/2020, co	TTCACCGAGGCCACGCGGAGTACGATCGAGTGTACAGTGAA
MT370872.1 Severe acute respiratory syndrome coronavirus 2 isolate SARS-CoV-2/human/USA/NY-PV08491/2020, co	TTCACCGAGGCCACGCGGAGTACGATCGAGTGTACAGTGAA
MT370873.1 Severe acute respiratory syndrome coronavirus 2 isolate SARS-CoV-2/human/USA/NY-PV08404/2020 OF	TTCACCGAGGCCACGCGGAGTACGATCGAGTGTACAGTGAA
MT370874.1 Severe acute respiratory syndrome coronavirus 2 isolate SARS-CoV-2/human/USA/NY-PV08149/2020, co	TTCACCGAGGCCACGCGGAGTACGATCGAGTGTACAGTGAA
MT370875.1 Severe acute respiratory syndrome coronavirus 2 isolate SARS-CoV-2/human/USA/NY-PV08473/2020, co	TTCACCGAGGCCACGCGGAGTACGATCGAGTGTACAGTGAA
MT370876.1 Severe acute respiratory syndrome coronavirus 2 isolate SARS-CoV-2/human/USA/NY-PV08448/2020, co	TTCACCGAGGCCACGCGGAGTACGATCGAGTGTACAGTGAA
MT370877.1 Severe acute respiratory syndrome coronavirus 2 isolate SARS-CoV-2/human/USA/NY-PV08474/2020, co	TTCACCGAGGCCACGCGGAGTACGATCGAGTGTACAGTGAA
MT370878.1 Severe acute respiratory syndrome coronavirus 2 isolate SARS-CoV-2/human/USA/NY-PV08413/2020, co	TTCACCGAGGCCACGCGGAGTACGATCGAGTGTACAGTGAA

MT370879.1 Severe acute respiratory syndrome coronavirus 2 isolate SARS-CoV-2/human/USA/NY-PV08405/2020 OF	TTCACCGAGGCCACGCGGAGTACGATCGAGTGTACAGTGAA
MT370880.1 Severe acute respiratory syndrome coronavirus 2 isolate SARS-CoV-2/human/USA/NY-PV08428/2020, co	TTCACCGAGGCCACGCGGAGTACGATCGAGTGTACAGTGAA
MT370881.1 Severe acute respiratory syndrome coronavirus 2 isolate SARS-CoV-2/human/USA/NY-PV08138/2020, co	TTCACCGAGGCCACGCGGAGTACGATCGAGTGTACAGTGAA
MT370882.1 Severe acute respiratory syndrome coronavirus 2 isolate SARS-CoV-2/human/USA/NY-PV08481/2020, co	TTCACCGAGGCCACGCGGAGTACGATCGAGTGTACAGTGAA
MT370883.1 Severe acute respiratory syndrome coronavirus 2 isolate SARS-CoV-2/human/USA/NY-PV08425/2020, co	TTCACCGAGGCCACGCGGAGTACGATCGAGTGTACAGTGAA
MT370884.1 Severe acute respiratory syndrome coronavirus 2 isolate SARS-CoV-2/human/USA/NY-PV08417/2020 OF	TTCACCGAGGCCACGCGGAGTACGATCGAGTGTACAGTGAA
MT370885.1 Severe acute respiratory syndrome coronavirus 2 isolate SARS-CoV-2/human/USA/NY-PV08461/2020, co	TTCACCGAGGCCACGCGGAGTACGATCGAGTGTACAGTGAA
MT370886.1 Severe acute respiratory syndrome coronavirus 2 isolate SARS-CoV-2/human/USA/NY-PV08450/2020, co	TTCACCGAGGCCACGCGGAGTACGATCGAGTGTACAGTGAA
MT370887.1 Severe acute respiratory syndrome coronavirus 2 isolate SARS-CoV-2/human/USA/NY-PV08476/2020 OF	TTCACCGAGGCCACGCGGAGTACGATCGAGTGTACAGTGAA
MT370888.1 Severe acute respiratory syndrome coronavirus 2 isolate SARS-CoV-2/human/USA/NY-PV08498/2020, co	TTCACCGAGGCCACGCGGAGTACGATCGAGTGTACAGTGAA
MT370889.1 Severe acute respiratory syndrome coronavirus 2 isolate SARS-CoV-2/human/USA/NY-PV08472/2020, co	TTCACCGAGGCCACGCGGAGTACGATCGAGTGTACAGTGAA
MT370890.1 Severe acute respiratory syndrome coronavirus 2 isolate SARS-CoV-2/human/USA/NY-PV08477/2020, co	TTCACCGAGGCCACGCGGAGTACGATCGAGTGTACAGTGAA
MT370891.1 Severe acute respiratory syndrome coronavirus 2 isolate SARS-CoV-2/human/USA/NY-PV08402/2020, co	TTCACCGAGGCCACGCGGAGTACGATCGAGTGTACAGTGAA
MT370892.1 Severe acute respiratory syndrome coronavirus 2 isolate SARS-CoV-2/human/USA/NY-PV08122/2020, co	TTCACCGAGGCCACGCGGAGTACGATCGAGTGTACAGTGAA
MT370893.1 Severe acute respiratory syndrome coronavirus 2 isolate SARS-CoV-2/human/USA/NY-PV08419/2020 OF	TTCACCGAGGCCACGCGGAGTACGATCGAGTGTACAGTGAA
MT370894.1 Severe acute respiratory syndrome coronavirus 2 isolate SARS-CoV-2/human/USA/NY-PV08496/2020, co	TTCACCGAGGCCACGCGGAGTACGATCGAGTGTACAGTGAA
MT370895.1 Severe acute respiratory syndrome coronavirus 2 isolate SARS-CoV-2/human/USA/NY-PV08415/2020, co	TTCACCGAGGCCACGCGGAGTACGATCGAGTGTACAGTGAA
MT370896.1 Severe acute respiratory syndrome coronavirus 2 isolate SARS-CoV-2/human/USA/NY-PV08459/2020, co	TTCACCGAGGCCACGCGGAGTACGATCGAGTGTACAGTGAA
MT370897.1 Severe acute respiratory syndrome coronavirus 2 isolate SARS-CoV-2/human/USA/NY-PV08452/2020, co	TTCACCGAGGCCACGCGGAGTACGATCGAGTGTACAGTGAA
MT370898.1 Severe acute respiratory syndrome coronavirus 2 isolate SARS-CoV-2/human/USA/NY-PV08487/2020, co	TTCACCGAGGCCACGCGGAGTACGATCGAGTGTACAGTGAA
MT370899.1 Severe acute respiratory syndrome coronavirus 2 isolate SARS-CoV-2/human/USA/NY-PV08455/2020, co	TTCACCGAGGCCACGCGGAGTACGATCGAGTGTACAGTGAA
MT370900.1 Severe acute respiratory syndrome coronavirus 2 isolate SARS-CoV-2/human/USA/NY-PV08410/2020 OF	TTCACCGAGGCCACGCGGAGTACGATCGAGTGTACAGTGAA
MT370901.1 Severe acute respiratory syndrome coronavirus 2 isolate SARS-CoV-2/human/USA/NY-PV08444/2020, co	TTCACCGAGGCCACGCGGAGTACGATCGAGTGTACAGTGAA
MT370902.1 Severe acute respiratory syndrome coronavirus 2 isolate SARS-CoV-2/human/USA/NY-PV08449/2020, co	TTCACCGAGGCCACGCGGAGTACGATCGAGTGTACAGTGAA
MT370903.1 Severe acute respiratory syndrome coronavirus 2 isolate SARS-CoV-2/human/USA/NY-PV08136/2020, co	TTCACCGAGGCCACGCGGAGTACGATCGAGTGTACAGTGAA
MT370905.1 Severe acute respiratory syndrome coronavirus 2 isolate SARS-CoV-2/human/USA/NY-PV08150/2020, co	TTCACCGAGGCCACGCGGAGTACGATCGAGTGTACAGTGAA
MT370906.1 Severe acute respiratory syndrome coronavirus 2 isolate SARS-CoV-2/human/USA/NY-PV08120/2020, co	TTCACCGAGGCCACGCGGAGTACGATCGAGTGTACAGTGAA
MT370907.1 Severe acute respiratory syndrome coronavirus 2 isolate SARS-CoV-2/human/USA/NY-PV08416/2020, co	TTCACCGAGGCCACGCGGAGTACGATCGAGTGTACAGTGAA
MT370908.1 Severe acute respiratory syndrome coronavirus 2 isolate SARS-CoV-2/human/USA/NY-PV08435/2020, co	TTCACCGAGGCCACGCGGAGTACGATCGAGTGTACAGTGAA
MT370909.1 Severe acute respiratory syndrome coronavirus 2 isolate SARS-CoV-2/human/USA/NY-PV08489/2020, co	TTCACCGAGGCCACGCGGAGTACGATCGAGTGTACAGTGAA
MT370910.1 Severe acute respiratory syndrome coronavirus 2 isolate SARS-CoV-2/human/USA/NY-PV08133/2020 OF	TTCACCGAGGCCACGCGGAGTACGATCGAGTGTACAGTGAA
MT370911.1 Severe acute respiratory syndrome coronavirus 2 isolate SARS-CoV-2/human/USA/NY-PV08493/2020, co	TTCACCGAGGCCACGCGGAGTACGATCGAGTGTACAGTGAA
MT370912.1 Severe acute respiratory syndrome coronavirus 2 isolate SARS-CoV-2/human/USA/NY-PV08427/2020, co	TTCACCGAGGCCACGCGGAGTACGATCGAGTGTACAGTGAA

MT370913.1 Severe acute respiratory syndrome coronavirus 2 isolate SARS-CoV-2/human/USA/NY-PV08143/2020, co	TTCACCGAGGCCACGCGGAGTACGATCGAGTGTACAGTGAA
MT370914.1 Severe acute respiratory syndrome coronavirus 2 isolate SARS-CoV-2/human/USA/NY-PV08447/2020, co	TTCACCGAGGCCACGCGGAGTACGATCGAGTGTACAGTGAA
MT370915.1 Severe acute respiratory syndrome coronavirus 2 isolate SARS-CoV-2/human/USA/NY-PV08125/2020, co	TTCACCGAGGCCACGCGGAGTACGATCGAGTGTACAGTGAA
MT370916.1 Severe acute respiratory syndrome coronavirus 2 isolate SARS-CoV-2/human/USA/NY-PV08443/2020, co	TTCACCGAGGCCACGCGGAGTACGATCGAGTGTACAGTGAA
MT370917.1 Severe acute respiratory syndrome coronavirus 2 isolate SARS-CoV-2/human/USA/NY-PV08429/2020, co	TTCACCGAGGCCACGCGGAGTACGATCGAGTGTACAGTGAA
MT370918.1 Severe acute respiratory syndrome coronavirus 2 isolate SARS-CoV-2/human/USA/NY-PV08414/2020 OF	TTCACCGAGGCCACGCGGAGTACGATCGAGTGTACAGTGAA
MT370919.1 Severe acute respiratory syndrome coronavirus 2 isolate SARS-CoV-2/human/USA/NY-PV08123/2020, co	TTCACCGAGGCCACGCGGAGTACGATCGAGTGTACAGTGAA
MT370920.1 Severe acute respiratory syndrome coronavirus 2 isolate SARS-CoV-2/human/USA/NY-PV08466/2020, co	TTCACCGAGGCCACGCGGAGTACGATCGAGTGTACAGTGAA
MT370925.1 Severe acute respiratory syndrome coronavirus 2 isolate SARS-CoV-2/human/USA/NY-PV09145/2020, co	TTCACCGAGGCCACGCGGAGTACGATCGAGTGTACAGTGAA
MT370927.1 Severe acute respiratory syndrome coronavirus 2 isolate SARS-CoV-2/human/USA/NY-PV09103/2020, co	TTCACCGAGGCCACGCGGAGTACGATCGAGTGTACAGTGAA
MT370928.1 Severe acute respiratory syndrome coronavirus 2 isolate SARS-CoV-2/human/USA/NY-PV09121/2020 OF	TTCACCGAGGCCACGCGGAGTACGATCGAGTGTACAGTGAA
MT370930.1 Severe acute respiratory syndrome coronavirus 2 isolate SARS-CoV-2/human/USA/NY-PV09153/2020, co	TTCACCGAGGCCACGCGGAGTACGATCGAGTGTACAGTGAA
MT370931.1 Severe acute respiratory syndrome coronavirus 2 isolate SARS-CoV-2/human/USA/NY-PV09164/2020, co	TTCACCGAGGCCACGCGGAGTACGATCGAGTGTACAGTGAA
MT370932.1 Severe acute respiratory syndrome coronavirus 2 isolate SARS-CoV-2/human/USA/NY-PV09195/2020, co	TTCACCGAGGCCACGCGGAGTACGATCGAGTGTACAGTGAA
MT370933.1 Severe acute respiratory syndrome coronavirus 2 isolate SARS-CoV-2/human/USA/NJ-PV09072/2020, co	TTCACCGAGGCCACGCGGAGTACGATCGAGTGTACAGTGAA
MT370934.1 Severe acute respiratory syndrome coronavirus 2 isolate SARS-CoV-2/human/USA/NY-PV09079/2020, co	TTCACCGAGGCCACGCGGAGTACGATCGAGTGTACAGTGAA
MT370935.1 Severe acute respiratory syndrome coronavirus 2 isolate SARS-CoV-2/human/USA/NY-PV09060/2020, co	TTCACCGAGGCCACGCGGAGTACGATCGAGTGTACAGTGAA
MT370937.1 Severe acute respiratory syndrome coronavirus 2 isolate SARS-CoV-2/human/USA/NY-PV09142/2020, co	TTCACCGAGGCCACGCGGAGTACGATCGAGTGTACAGTGAA
MT370939.1 Severe acute respiratory syndrome coronavirus 2 isolate SARS-CoV-2/human/USA/NY-PV09119/2020, co	TTCACCGAGGCCACGCGGAGTACGATCGAGTGTACAGTGAA
MT370940.1 Severe acute respiratory syndrome coronavirus 2 isolate SARS-CoV-2/human/USA/NY-PV09182/2020, co	TTCACCGAGGCCACGCGGAGTACGATCGAGTGTACAGTGAA
MT370942.1 Severe acute respiratory syndrome coronavirus 2 isolate SARS-CoV-2/human/USA/NY-PV09128/2020, co	TTCACCGAGGCCACGCGGAGTACGATCGAGTGTACAGTGAA
MT370944.1 Severe acute respiratory syndrome coronavirus 2 isolate SARS-CoV-2/human/USA/NY-PV09120/2020, co	TTCACCGAGGCCACGCGGAGTACGATCGAGTGTACAGTGAA
MT370946.1 Severe acute respiratory syndrome coronavirus 2 isolate SARS-CoV-2/human/USA/NY-PV09077/2020, co	TTCACCGAGGCCACGCGGAGTACGATCGAGTGTACAGTGAA
MT370947.1 Severe acute respiratory syndrome coronavirus 2 isolate SARS-CoV-2/human/USA/NY-PV09039/2020, co	TTCACCGAGGCCACGCGGAGTACGATCGAGTGTACAGTGAA
MT370948.1 Severe acute respiratory syndrome coronavirus 2 isolate SARS-CoV-2/human/USA/NY-PV09144/2020, co	TTCACCGAGGCCACGCGGAGTACGATCGAGTGTACAGTGAA
MT370949.1 Severe acute respiratory syndrome coronavirus 2 isolate SARS-CoV-2/human/USA/NY-PV09075/2020, co	TTCACCGAGGCCACGCGGAGTACGATCGAGTGTACAGTGAA
MT370950.1 Severe acute respiratory syndrome coronavirus 2 isolate SARS-CoV-2/human/USA/NY-PV09193/2020, co	TTCACCGAGGCCACGCGGAGTACGATCGAGTGTACAGTGAA
MT370952.1 Severe acute respiratory syndrome coronavirus 2 isolate SARS-CoV-2/human/USA/NY-PV09041/2020, co	TTCACCGAGGCCACGCGGAGTACGATCGAGTGTACAGTGAA
MT370954.1 Severe acute respiratory syndrome coronavirus 2 isolate SARS-CoV-2/human/USA/NY-PV09156/2020, co	TTCACCGAGGCCACGCGGAGTACGATCGAGTGTACAGTGAA
MT370956.1 Severe acute respiratory syndrome coronavirus 2 isolate SARS-CoV-2/human/USA/NY-PV09129/2020, co	TTCACCGAGGCCACGCGGAGTACGATCGAGTGTACAGTGAA
MT370957.1 Severe acute respiratory syndrome coronavirus 2 isolate SARS-CoV-2/human/USA/NY-PV09099/2020, co	TTCACCGAGGCCACGCGGAGTACGATCGAGTGTACAGTGAA
MT370958.1 Severe acute respiratory syndrome coronavirus 2 isolate SARS-CoV-2/human/USA/NY-PV09132/2020, co	TTCACCGAGGCCACGCGGAGTACGATCGAGTGTACAGTGAA
MT370960.1 Severe acute respiratory syndrome coronavirus 2 isolate SARS-CoV-2/human/USA/NY-PV09097/2020, co	TTCACCGAGGCCACGCGGAGTACGATCGAGTGTACAGTGAA

MT370961.1 Severe acute respiratory syndrome coronavirus 2 isolate SARS-CoV-2/human/USA/NY-PV09140/2020, co	TTCACCGAGGCCACGCGGAGTACGATCGAGTGTACAGTGAA
MT370962.1 Severe acute respiratory syndrome coronavirus 2 isolate SARS-CoV-2/human/USA/NY-PV09056/2020, co	TTCACCGAGGCCACGCGGAGTACGATCGAGTGTACAGTGAA
MT370963.1 Severe acute respiratory syndrome coronavirus 2 isolate SARS-CoV-2/human/USA/NY-PV09179/2020 OF	TTCACCGAGGCCACGCGGAGTACGATCGAGTGTACAGTGAA
MT370964.1 Severe acute respiratory syndrome coronavirus 2 isolate SARS-CoV-2/human/USA/NY-PV09032/2020, co	TTCACCGAGGCCACGCGGAGTACGATCGAGTGTACAGTGAA
MT370965.1 Severe acute respiratory syndrome coronavirus 2 isolate SARS-CoV-2/human/USA/NY-PV09116/2020, co	TTCACCGAGGCCACGCGGAGTACGATCGAGTGTACAGTGAA
MT370966.1 Severe acute respiratory syndrome coronavirus 2 isolate SARS-CoV-2/human/USA/NY-PV09180/2020, co	TTCACCGAGGCCACGCGGAGTACGATCGAGTGTACAGTGAA
MT370967.1 Severe acute respiratory syndrome coronavirus 2 isolate SARS-CoV-2/human/USA/NY-PV09303/2020, co	TTCACCGAGGCCACGCGGAGTACGATCGAGTGTACAGTGAA
MT370970.1 Severe acute respiratory syndrome coronavirus 2 isolate SARS-CoV-2/human/USA/NY-PV09192/2020, co	TTCACCGAGGCCACGCGGAGTACGATCGAGTGTACAGTGAA
MT370971.1 Severe acute respiratory syndrome coronavirus 2 isolate SARS-CoV-2/human/USA/NY-PV09061/2020, co	TTCACCGAGGCCACGCGGAGTACGATCGAGTGTACAGTGAA
MT370972.1 Severe acute respiratory syndrome coronavirus 2 isolate SARS-CoV-2/human/USA/NY-PV09138/2020, co	TTCACCGAGGCCACGCGGAGTACGATCGAGTGTACAGTGAA
MT370975.1 Severe acute respiratory syndrome coronavirus 2 isolate SARS-CoV-2/human/USA/NY-PV09134/2020, co	TTCACCGAGGCCACGCGGAGTACGATCGAGTGTACAGTGAA
MT370976.1 Severe acute respiratory syndrome coronavirus 2 isolate SARS-CoV-2/human/USA/NY-PV09069/2020, co	TTCACCGAGGCCACGCGGAGTACGATCGAGTGTACAGTGAA
MT370977.1 Severe acute respiratory syndrome coronavirus 2 isolate SARS-CoV-2/human/USA/NY-PV09023/2020, co	TTCACCGAGGCCACGCGGAGTACGATCGAGTGTACAGTGAA
MT370979.1 Severe acute respiratory syndrome coronavirus 2 isolate SARS-CoV-2/human/USA/NY-PV09044/2020, co	TTCACCGAGGCCACGCGGAGTACGATCGAGTGTACAGTGAA
MT370980.1 Severe acute respiratory syndrome coronavirus 2 isolate SARS-CoV-2/human/USA/NY-PV09067/2020, co	TTCACCGAGGCCACGCGGAGTACGATCGAGTGTACAGTGAA
MT370981.1 Severe acute respiratory syndrome coronavirus 2 isolate SARS-CoV-2/human/USA/NY-PV09123/2020, co	TTCACCGAGGCCACGCGGAGTACGATCGAGTGTACAGTGAA
MT370982.1 Severe acute respiratory syndrome coronavirus 2 isolate SARS-CoV-2/human/USA/NY-PV09170/2020 OF	TTCACCGAGGCCACGCGGAGTACGATCGAGTGTACAGTGAA
MT370984.1 Severe acute respiratory syndrome coronavirus 2 isolate SARS-CoV-2/human/USA/NY-PV09043/2020, co	TTCACCGAGGCCACGCGGAGTACGATCGAGTGTACAGTGAA
MT370985.1 Severe acute respiratory syndrome coronavirus 2 isolate SARS-CoV-2/human/USA/NY-PV09147/2020, co	TTCACCGAGGCCACGCGGAGTACGATCGAGTGTACAGTGAA
MT370986.1 Severe acute respiratory syndrome coronavirus 2 isolate SARS-CoV-2/human/USA/NY-PV09190/2020, co	TTCACCGAGGCCACGCGGAGTACGATCGAGTGTACAGTGAA
MT370987.1 Severe acute respiratory syndrome coronavirus 2 isolate SARS-CoV-2/human/USA/NY-PV09092/2020, co	TTCACCGAGGCCACGCGGAGTACGATCGAGTGTACAGTGAA
MT370988.1 Severe acute respiratory syndrome coronavirus 2 isolate SARS-CoV-2/human/USA/NY-PV09197/2020, co	TTCACCGAGGCCACGCGGAGTACGATCGAGTGTACAGTGAA
MT370991.1 Severe acute respiratory syndrome coronavirus 2 isolate SARS-CoV-2/human/USA/NY-PV09028/2020, co	TTCACCGAGGCCACGCGGAGTACGATCGAGTGTACAGTGAA
MT370993.1 Severe acute respiratory syndrome coronavirus 2 isolate SARS-CoV-2/human/USA/NY-PV09125/2020, co	TTCACCGAGGCCACGCGGAGTACGATCGAGTGTACAGTGAA
MT370994.1 Severe acute respiratory syndrome coronavirus 2 isolate SARS-CoV-2/human/USA/NY-PV09194/2020, co	TTCACCGAGGCCACGCGGAGTACGATCGAGTGTACAGTGAA
MT370996.1 Severe acute respiratory syndrome coronavirus 2 isolate SARS-CoV-2/human/USA/NY-PV09181/2020, co	TTCACCGAGGCCACGCGGAGTACGATCGAGTGTACAGTGAA
MT370997.1 Severe acute respiratory syndrome coronavirus 2 isolate SARS-CoV-2/human/USA/NY-PV09088/2020, co	TTCACCGAGGCCACGCGGAGTACGATCGAGTGTACAGTGAA
MT370998.1 Severe acute respiratory syndrome coronavirus 2 isolate SARS-CoV-2/human/USA/NY-PV09183/2020, co	TTCACCGAGGCCACGCGGAGTACGATCGAGTGTACAGTGAA
MT371000.1 Severe acute respiratory syndrome coronavirus 2 isolate SARS-CoV-2/human/USA/NY-PV09045/2020, co	TTCACCGAGGCCACGCGGAGTACGATCGAGTGTACAGTGAA
MT371001.1 Severe acute respiratory syndrome coronavirus 2 isolate SARS-CoV-2/human/USA/NY-PV09171/2020, co	TTCACCGAGGCCACGCGGAGTACGATCGAGTGTACAGTGAA
MT371002.1 Severe acute respiratory syndrome coronavirus 2 isolate SARS-CoV-2/human/USA/NY-PV09200/2020, co	TTCACCGAGGCCACGCGGAGTACGATCGAGTGTACAGTGAA
MT371003.1 Severe acute respiratory syndrome coronavirus 2 isolate SARS-CoV-2/human/USA/NY-PV09076/2020 OF	TTCACCGAGGCCACGCGGAGTACGATCGAGTGTACAGTGAA
MT371006.1 Severe acute respiratory syndrome coronavirus 2 isolate SARS-CoV-2/human/USA/NY-PV09068/2020, co	TTCACCGAGGCCACGCGGAGTACGATCGAGTGTACAGTGAA

MT371007.1 Severe acute respiratory syndrome coronavirus 2 isolate SARS-CoV-2/human/USA/NY-PV09177/2020, co	TTCACCGAGGCCACGCGGAGTACGATCGAGTGTACAGTGAA
MT371008.1 Severe acute respiratory syndrome coronavirus 2 isolate SARS-CoV-2/human/USA/NY-PV09165/2020, co	TTCACCGAGGCCACGCGGAGTACGATCGAGTGTACAGTGAA
MT371009.1 Severe acute respiratory syndrome coronavirus 2 isolate SARS-CoV-2/human/USA/NY-PV09057/2020, co	TTCACCGAGGCCACGCGGAGTACGATCGAGTGTACAGTGAA
MT371012.1 Severe acute respiratory syndrome coronavirus 2 isolate SARS-CoV-2/human/USA/NY-PV09169/2020, co	TTCACCGAGGCCACGCGGAGTACGATCGAGTGTACAGTGAA
MT371013.1 Severe acute respiratory syndrome coronavirus 2 isolate SARS-CoV-2/human/USA/NY-PV09102/2020, co	TTCACCGAGGCCACGCGGAGTACGATCGAGTGTACAGTGAA
MT371015.1 Severe acute respiratory syndrome coronavirus 2 isolate SARS-CoV-2/human/USA/NY-PV09187/2020, co	TTCACCGAGGCCACGCGGAGTACGATCGAGTGTACAGTGAA
MT371016.1 Severe acute respiratory syndrome coronavirus 2 isolate SARS-CoV-2/human/USA/NY-PV09308/2020, co	TTCACCGAGGCCACGCGGAGTACGATCGAGTGTACAGTGAA
MT371017.1 Severe acute respiratory syndrome coronavirus 2 isolate SARS-CoV-2/human/USA/NY-PV09309/2020, co	TTCACCGAGGCCACGCGGAGTACGATCGAGTGTACAGTGAA
MT371018.1 Severe acute respiratory syndrome coronavirus 2 isolate SARS-CoV-2/human/USA/NY-PV09186/2020, co	TTCACCGAGGCCACGCGGAGTACGATCGAGTGTACAGTGAA
MT371019.1 Severe acute respiratory syndrome coronavirus 2 isolate SARS-CoV-2/human/USA/NY-PV09029/2020, co	TTCACCGAGGCCACGCGGAGTACGATCGAGTGTACAGTGAA
MT371022.1 Severe acute respiratory syndrome coronavirus 2 isolate SARS-CoV-2/human/USA/NY-PV09151/2020, co	TTCACCGAGGCCACGCGGAGTACGATCGAGTGTACAGTGAA
MT371023.1 Severe acute respiratory syndrome coronavirus 2 isolate SARS-CoV-2/human/USA/NY-PV09046/2020, co	TTCACCGAGGCCACGCGGAGTACGATCGAGTGTACAGTGAA
MT371026.1 Severe acute respiratory syndrome coronavirus 2 isolate SARS-CoV-2/human/USA/NY-PV09090/2020, co	TTCACCGAGGCCACGCGGAGTACGATCGAGTGTACAGTGAA
MT371027.1 Severe acute respiratory syndrome coronavirus 2 isolate SARS-CoV-2/human/USA/NY-PV09073/2020, co	TTCACCGAGGCCACGCGGAGTACGATCGAGTGTACAGTGAA
MT371028.1 Severe acute respiratory syndrome coronavirus 2 isolate SARS-CoV-2/human/USA/NY-PV09021/2020, co	TTCACCGAGGCCACGCGGAGTACGATCGAGTGTACAGTGAA
MT371029.1 Severe acute respiratory syndrome coronavirus 2 isolate SARS-CoV-2/human/USA/NY-PV09137/2020, co	TTCACCGAGGCCACGCGGAGTACGATCGAGTGTACAGTGAA
MT371031.1 Severe acute respiratory syndrome coronavirus 2 isolate SARS-CoV-2/human/USA/NY-PV09050/2020 OF	TTCACCGAGGCCACGCGGAGTACGATCGAGTGTACAGTGAA
MT371032.1 Severe acute respiratory syndrome coronavirus 2 isolate SARS-CoV-2/human/USA/NY-PV09127/2020, co	TTCACCGAGGCCACGCGGAGTACGATCGAGTGTACAGTGAA
MT371033.1 Severe acute respiratory syndrome coronavirus 2 isolate SARS-CoV-2/human/USA/NY-PV09304/2020, co	TTCACCGAGGCCACGCGGAGTACGATCGAGTGTACAGTGAA
MT371034.1 Severe acute respiratory syndrome coronavirus 2 isolate SARS-CoV-2/human/USA/NY-PV09158/2020, co	TTCACCGAGGCCACGCGGAGTACGATCGAGTGTACAGTGAA
MT371035.1 Severe acute respiratory syndrome coronavirus 2 isolate SARS-CoV-2/human/USA/NY-PV09025/2020, co	TTCACCGAGGCCACGCGGAGTACGATCGAGTGTACAGTGAA
MT371036.1 Severe acute respiratory syndrome coronavirus 2 isolate SARS-CoV-2/human/USA/NY-PV09198/2020, co	TTCACCGAGGCCACGCGGAGTACGATCGAGTGTACAGTGAA
MT371038.1 Severe acute respiratory syndrome coronavirus 2 isolate SARS-CoV-2/human/USA/NY-PV09189/2020, co	TTCACCGAGGCCACGCGGAGTACGATCGAGTGTACAGTGAA
MT371048.1 Severe acute respiratory syndrome coronavirus 2 isolate SARS-CoV-2/human/LKA/COV53/2020, complet	TTCACCGAGGCCACGCGGAGTACGATCGAGTGTACAGTGAA
MT371049.1 Severe acute respiratory syndrome coronavirus 2 isolate SARS-CoV-2/human/LKA/COV91/2020, complet	TTCACCGAGGCCACGCGGAGTACGATCGAGTGTACAGTGAA
MT371050.1 Severe acute respiratory syndrome coronavirus 2 isolate SARS-CoV-2/human/LKA/COV486/2020, comple	TTCACCGAGGCCACGCGGAGTACGATCGAGTGTACAGTGAA
MT371568.1 Severe acute respiratory syndrome coronavirus 2 isolate SARS-CoV-2/human/CZE/2256/2020, complete	TTCACCGAGGCCACGCGGAGTACGATCGAGTGTACAGTGAA
MT371569.1 Severe acute respiratory syndrome coronavirus 2 isolate SARS-CoV-2/human/CZE/CzechiaMotol_2122/2	TTCACCGAGGCCACGCGGAGTACGATCGAGTGTACAGTGAA
MT371570.1 Severe acute respiratory syndrome coronavirus 2 isolate SARS-CoV-2/human/CZE/CzechiaMotol_2448/2	TTCACCGAGGCCACGCGGAGTACGATCGAGTGTACAGTGAA
MT371571.1 Severe acute respiratory syndrome coronavirus 2 isolate SARS-CoV-2/human/CZE/CzechiaMotol_1968/2	TTCACCGAGGCCACGCGGAGTACGATCGAGTGTACAGTGAA
MT371572.1 Severe acute respiratory syndrome coronavirus 2 isolate SARS-CoV-2/human/CZE/CzechiaMotol_2174/2	TTCACCGAGGCCACGCGGAGTACGATCGAGTGTACAGTGAA
MT371573.1 Severe acute respiratory syndrome coronavirus 2 isolate SARS-CoV-2/human/CZE/CzechiaMotol_1899/2	TTCACCGAGGCCACGCGGAGTACGATCGAGTGTACAGTGAA
MT371574.1 Severe acute respiratory syndrome coronavirus 2 isolate SARS-CoV-2/human/CZE/CzechiaMotol_1967/2	TTCACCGAGGCCACGCGGAGTACGATCGAGTGTACAGTGAA

MT372482.1 Severe acute respiratory syndrome coronavirus 2 isolate SARS-CoV-2/human/MYS/190300/2020, compl	TTCACCGAGGCCACGCGGAGTACGATCGAGTGTACAGTGAA
MT374101.1 Severe acute respiratory syndrome coronavirus 2 isolate SARS-CoV-2/human/TWN/CGMH-CGU-02/2020	TTCACCGAGGCCACGCGGAGTACGATCGAGTGTACAGTGAA
MT374105.1 Severe acute respiratory syndrome coronavirus 2 isolate SARS-CoV-2/human/TWN/CGMH-CGU-09/2020	TTCACCGAGGCCACGCGGAGTACGATCGAGTGTACAGTGAA
MT374108.1 Severe acute respiratory syndrome coronavirus 2 isolate SARS-CoV-2/human/TWN/CGMH-CGU-12/2020	TTCACCGAGGCCACGCGGAGTACGATCGAGTGTACAGTGAA
MT374109.1 Severe acute respiratory syndrome coronavirus 2 isolate SARS-CoV-2/human/TWN/CGMH-CGU-13/2020	TTCACCGAGGCCACGCGGAGTACGATCGAGTGTACAGTGAA
MT374110.1 Severe acute respiratory syndrome coronavirus 2 isolate SARS-CoV-2/human/TWN/CGMH-CGU-14/2020	TTCACCGAGGCCACGCGGAGTACGATCGAGTGTACAGTGAA
MT374112.1 Severe acute respiratory syndrome coronavirus 2 isolate SARS-CoV-2/human/TWN/CGMH-CGU-16/2020	TTCACCGAGGCCACGCGGAGTACGATCGAGTGTACAGTGAA
MT374113.1 Severe acute respiratory syndrome coronavirus 2 isolate SARS-CoV-2/human/TWN/CGMH-CGU-17/2020	TTCACCGAGGCCACGCGGAGTACGATCGAGTGTACAGTGAA
MT374114.1 Severe acute respiratory syndrome coronavirus 2 isolate SARS-CoV-2/human/TWN/CGMH-CGU-18/2020	TTCACCGAGGCCACGCGGAGTACGATCGAGTGTACAGTGAA
MT374115.1 Severe acute respiratory syndrome coronavirus 2 isolate SARS-CoV-2/human/TWN/CGMH-CGU-19/2020	TTCACCGAGGCCACGCGGAGTACGATCGAGTGTACAGTGAA
MT374116.1 Severe acute respiratory syndrome coronavirus 2 isolate SARS-CoV-2/human/TWN/CGMH-CGU-20/2020	TTCACCGAGGCCACGCGGAGTACGATCGAGTGTACAGTGAA
MT375428.1 Severe acute respiratory syndrome coronavirus 2 isolate SARS-CoV-2/human/USA/WA-UW-4538/2020, c	TTCACCGAGGCCACGCGGAGTACGATCGAGTGTACAGTGAA
MT375429.1 Severe acute respiratory syndrome coronavirus 2 isolate SARS-CoV-2/human/USA/ID-UW-4547/2020, co	TTCACCGAGGCCACGCGGAGTACGATCGAGTGTACAGTGAA
MT375430.1 Severe acute respiratory syndrome coronavirus 2 isolate SARS-CoV-2/human/USA/WA-UW-4549/2020, c	TTCACCGAGGCCACGCGGAGTACGATCGAGTGTACAGTGAA
MT375431.1 Severe acute respiratory syndrome coronavirus 2 isolate SARS-CoV-2/human/USA/ID-UW-4552/2020, co	TTCACCGAGGCCACGCGGAGTACGATCGAGTGTACAGTGAA
MT375432.1 Severe acute respiratory syndrome coronavirus 2 isolate SARS-CoV-2/human/USA/WA-UW-4564/2020, c	TTCACCGAGGCCACGCGGAGTACGATCGAGTGTACAGTGAA
MT375433.1 Severe acute respiratory syndrome coronavirus 2 isolate SARS-CoV-2/human/USA/WA-UW-4565/2020, c	TTCACCGAGGCCACGCGGAGTACGATCGAGTGTACAGTGAA
MT375434.1 Severe acute respiratory syndrome coronavirus 2 isolate SARS-CoV-2/human/USA/WA-UW-4578/2020, c	TTCACCGAGGCCACGCGGAGTACGATCGAGTGTACAGTGAA
MT375437.1 Severe acute respiratory syndrome coronavirus 2 isolate SARS-CoV-2/human/USA/WA-UW-4593/2020, c	TTCACCGAGGCCACGCGGAGTACGATCGAGTGTACAGTGAA
MT375438.1 Severe acute respiratory syndrome coronavirus 2 isolate SARS-CoV-2/human/USA/WA-UW-4594/2020, c	TTCACCGAGGCCACGCGGAGTACGATCGAGTGTACAGTGAA
MT375440.1 Severe acute respiratory syndrome coronavirus 2 isolate SARS-CoV-2/human/USA/WA-UW-4602/2020, c	TTCACCGAGGCCACGCGGAGTACGATCGAGTGTACAGTGAA
MT375441.1 Severe acute respiratory syndrome coronavirus 2 isolate SARS-CoV-2/human/USA/WA-UW-4609/2020, c	TTCACCGAGGCCACGCGGAGTACGATCGAGTGTACAGTGAA
MT375443.1 Severe acute respiratory syndrome coronavirus 2 isolate SARS-CoV-2/human/USA/WA-UW-4612/2020, c	TTCACCGAGGCCACGCGGAGTACGATCGAGTGTACAGTGAA
MT375445.1 Severe acute respiratory syndrome coronavirus 2 isolate SARS-CoV-2/human/USA/WA-UW-4732/2020, c	TTCACCGAGGCCACGCGGAGTACGATCGAGTGTACAGTGAA
MT375446.1 Severe acute respiratory syndrome coronavirus 2 isolate SARS-CoV-2/human/USA/WA-UW-4734/2020, c	TTCACCGAGGCCACGCGGAGTACGATCGAGTGTACAGTGAA
MT375447.1 Severe acute respiratory syndrome coronavirus 2 isolate SARS-CoV-2/human/USA/WA-UW-4741/2020, c	TTCACCGAGGCCACGCGGAGTACGATCGAGTGTACAGTGAA
MT375449.1 Severe acute respiratory syndrome coronavirus 2 isolate SARS-CoV-2/human/USA/WA-UW-4749/2020, c	TTCACCGAGGCCACGCGGAGTACGATCGAGTGTACAGTGAA
MT375450.1 Severe acute respiratory syndrome coronavirus 2 isolate SARS-CoV-2/human/USA/WA-UW-4760/2020, c	TTCACCGAGGCCACGCGGAGTACGATCGAGTGTACAGTGAA
MT375451.1 Severe acute respiratory syndrome coronavirus 2 isolate SARS-CoV-2/human/USA/WA-UW-4763/2020, c	TTCACCGAGGCCACGCGGAGTACGATCGAGTGTACAGTGAA
MT375453.1 Severe acute respiratory syndrome coronavirus 2 isolate SARS-CoV-2/human/USA/WA-UW-4769/2020, c	TTCACCGAGGCCACGCGGAGTACGATCGAGTGTACAGTGAA
MT375454.1 Severe acute respiratory syndrome coronavirus 2 isolate SARS-CoV-2/human/USA/WA-UW-4770/2020, c	TTCACCGAGGCCACGCGGAGTACGATCGAGTGTACAGTGAA
MT375456.1 Severe acute respiratory syndrome coronavirus 2 isolate SARS-CoV-2/human/USA/WA-UW-4777/2020, c	TTCACCGAGGCCACGCGGAGTACGATCGAGTGTACAGTGAA
MT375457.1 Severe acute respiratory syndrome coronavirus 2 isolate SARS-CoV-2/human/USA/UNKNOWN-UW-4645	TTCACCGAGGCCACGCGGAGTACGATCGAGTGTACAGTGAA

MT375458.1 Severe acute respiratory syndrome coronavirus 2 isolate SARS-CoV-2/human/USA/WA-UW-4649/2020, c	TTCACCGAGGCCACGCGGAGTACGATCGAGTGTACAGTGAA
MT375459.1 Severe acute respiratory syndrome coronavirus 2 isolate SARS-CoV-2/human/USA/WA-UW-4677/2020, c	TTCACCGAGGCCACGCGGAGTACGATCGAGTGTACAGTGAA
MT375460.1 Severe acute respiratory syndrome coronavirus 2 isolate SARS-CoV-2/human/USA/WA-UW-4695/2020, c	TTCACCGAGGCCACGCGGAGTACGATCGAGTGTACAGTGAA
MT375461.1 Severe acute respiratory syndrome coronavirus 2 isolate SARS-CoV-2/human/USA/WA-UW-4705/2020, c	TTCACCGAGGCCACGCGGAGTACGATCGAGTGTACAGTGAA
MT375462.1 Severe acute respiratory syndrome coronavirus 2 isolate SARS-CoV-2/human/USA/WA-UW-4707/2020, c	TTCACCGAGGCCACGCGGAGTACGATCGAGTGTACAGTGAA
MT375463.1 Severe acute respiratory syndrome coronavirus 2 isolate SARS-CoV-2/human/USA/WA-UW-4892/2020, c	TTCACCGAGGCCACGCGGAGTACGATCGAGTGTACAGTGAA
MT375464.1 Severe acute respiratory syndrome coronavirus 2 isolate SARS-CoV-2/human/USA/WA-UW-4897/2020 C	TTCACCGAGGCCACGCGGAGTACGATCGAGTGTACAGTGAA
MT375465.1 Severe acute respiratory syndrome coronavirus 2 isolate SARS-CoV-2/human/USA/WA-UW-4921/2020, c	TTCACCGAGGCCACGCGGAGTACGATCGAGTGTACAGTGAA
MT375466.1 Severe acute respiratory syndrome coronavirus 2 isolate SARS-CoV-2/human/USA/WA-UW-4925/2020, c	TTCACCGAGGCCACGCGGAGTACGATCGAGTGTACAGTGAA
MT375467.1 Severe acute respiratory syndrome coronavirus 2 isolate SARS-CoV-2/human/USA/WA-UW-4999/2020, c	TTCACCGAGGCCACGCGGAGTACGATCGAGTGTACAGTGAA
MT375468.1 Severe acute respiratory syndrome coronavirus 2 isolate SARS-CoV-2/human/USA/WA-UW-5025/2020, c	TTCACCGAGGCCACGCGGAGTACGATCGAGTGTACAGTGAA
MT375469.1 Severe acute respiratory syndrome coronavirus 2 isolate SARS-CoV-2/human/USA/CT-UW-5036/2020, co	TTCACCGAGGCCACGCGGAGTACGATCGAGTGTACAGTGAA
MT375470.1 Severe acute respiratory syndrome coronavirus 2 isolate SARS-CoV-2/human/USA/CT-UW-5038/2020, co	TTCACCGAGGCCACGCGGAGTACGATCGAGTGTACAGTGAA
MT375471.1 Severe acute respiratory syndrome coronavirus 2 isolate SARS-CoV-2/human/USA/CT-UW-5043/2020, co	TTCACCGAGGCCACGCGGAGTACGATCGAGTGTACAGTGAA
MT375472.1 Severe acute respiratory syndrome coronavirus 2 isolate SARS-CoV-2/human/USA/UNKNOWN-UW-5044	TTCACCGAGGCCACGCGGAGTACGATCGAGTGTACAGTGAA
MT375473.1 Severe acute respiratory syndrome coronavirus 2 isolate SARS-CoV-2/human/USA/WA-UW-5065/2020 C	TTCACCGAGGCCACGCGGAGTACGATCGAGTGTACAGTGAA
MT375474.1 Severe acute respiratory syndrome coronavirus 2 isolate SARS-CoV-2/human/USA/CT-UW-5073/2020, co	TTCACCGAGGCCACGCGGAGTACGATCGAGTGTACAGTGAA
MT375475.1 Severe acute respiratory syndrome coronavirus 2 isolate SARS-CoV-2/human/USA/WA-UW-5095/2020 C	TTCACCGAGGCCACGCGGAGTACGATCGAGTGTACAGTGAA
MT375478.1 Severe acute respiratory syndrome coronavirus 2 isolate SARS-CoV-2/human/USA/WA-UW-5158/2020, c	TTCACCGAGGCCACGCGGAGTACGATCGAGTGTACAGTGAA
MT375479.1 Severe acute respiratory syndrome coronavirus 2 isolate SARS-CoV-2/human/USA/WA-UW-5164/2020 C	TTCACCGAGGCCACGCGGAGTACGATCGAGTGTACAGTGAA
MT375480.1 Severe acute respiratory syndrome coronavirus 2 isolate SARS-CoV-2/human/USA/WA-UW-5166/2020, c	TTCACCGAGGCCACGCGGAGTACGATCGAGTGTACAGTGAA
MT375481.1 Severe acute respiratory syndrome coronavirus 2 isolate SARS-CoV-2/human/USA/WA-UW-5169/2020, c	TTCACCGAGGCCACGCGGAGTACGATCGAGTGTACAGTGAA
MT375482.1 Severe acute respiratory syndrome coronavirus 2 isolate SARS-CoV-2/human/USA/WA-UW-5170/2020, c	TTCACCGAGGCCACGCGGAGTACGATCGAGTGTACAGTGAA
MT375483.1 Severe acute respiratory syndrome coronavirus 2 isolate SARS-CoV-2/human/USA/UNKNOWN-UW-5172	TTCACCGAGGCCACGCGGAGTACGATCGAGTGTACAGTGAA
MT380728.1 Severe acute respiratory syndrome coronavirus 2 isolate SARS-CoV-2/human/USA/DC-CDC-1734/2020, c	TTCACCGAGGCCACGCGGAGTACGATCGAGTGTACAGTGAA
MT380729.1 Severe acute respiratory syndrome coronavirus 2 isolate SARS-CoV-2/human/USA/VI-CDC-1999/2020, co	TTCACCGAGGCCACGCGGAGTACGATCGAGTGTACAGTGAA
MT380731.1 Severe acute respiratory syndrome coronavirus 2 isolate SARS-CoV-2/human/USA/DC-CDC-9096/2020, c	TTCACCGAGGCCACGCGGAGTACGATCGAGTGTACAGTGAA
MT380732.1 Severe acute respiratory syndrome coronavirus 2 isolate SARS-CoV-2/human/USA/DC-CDC-9092/2020, c	TTCACCGAGGCCACGCGGAGTACGATCGAGTGTACAGTGAA
MT380733.1 Severe acute respiratory syndrome coronavirus 2 isolate SARS-CoV-2/human/USA/DC-CDC-1738/2020, c	TTCACCGAGGCCACGCGGAGTACGATCGAGTGTACAGTGAA
MT380734.1 Severe acute respiratory syndrome coronavirus 2 isolate SARS-CoV-2/human/USA/LA-CDC-1894/2020, c	TTCACCGAGGCCACGCGGAGTACGATCGAGTGTACAGTGAA
MT385414.1 Severe acute respiratory syndrome coronavirus 2 isolate SARS-CoV-2/human/USA/CA-CZB0104/2020, co	TTCACCGAGGCCACGCGGAGTACGATCGAGTGTACAGTGAA
MT385415.1 Severe acute respiratory syndrome coronavirus 2 isolate SARS-CoV-2/human/USA/CA-CZB071/2020, cor	TTCACCGAGGCCACGCGGAGTACGATCGAGTGTACAGTGAA
MT385416.1 Severe acute respiratory syndrome coronavirus 2 isolate SARS-CoV-2/human/USA/CA-CZB0138/2020, co	TTCACCGAGGCCACGCGGAGTACGATCGAGTGTACAGTGAA

MT385417.1 Severe acute respiratory syndrome coronavirus 2 isolate SARS-CoV-2/human/USA/CA-CZB0157/2020, co	TTCACCGAGGCCACGCGGAGTACGATCGAGTGTACAGTGAA
MT385418.1 Severe acute respiratory syndrome coronavirus 2 isolate SARS-CoV-2/human/USA/CA-CZB0703/2020, co	TTCACCGAGGCCACGCGGAGTACGATCGAGTGTACAGTGAA
MT385419.1 Severe acute respiratory syndrome coronavirus 2 isolate SARS-CoV-2/human/USA/CA-CZB0133/2020, co	TTCACCGAGGCCACGCGGAGTACGATCGAGTGTACAGTGAA
MT385420.1 Severe acute respiratory syndrome coronavirus 2 isolate SARS-CoV-2/human/USA/CA-CZB077/2020, cor	TTCACCGAGGCCACGCGGAGTACGATCGAGTGTACAGTGAA
MT385421.1 Severe acute respiratory syndrome coronavirus 2 isolate SARS-CoV-2/human/USA/CA-CZB015/2020, cor	TTCACCGAGGCCACGCGGAGTACGATCGAGTGTACAGTGAA
MT385422.1 Severe acute respiratory syndrome coronavirus 2 isolate SARS-CoV-2/human/USA/CA-CZB084/2020, cor	TTCACCGAGGCCACGCGGAGTACGATCGAGTGTACAGTGAA
MT385423.1 Severe acute respiratory syndrome coronavirus 2 isolate SARS-CoV-2/human/USA/CA-CZB069/2020, cor	TTCACCGAGGCCACGCGGAGTACGATCGAGTGTACAGTGAA
MT385424.1 Severe acute respiratory syndrome coronavirus 2 isolate SARS-CoV-2/human/USA/CA-CZB0103/2020, co	TTCACCGAGGCCACGCGGAGTACGATCGAGTGTACAGTGAA
MT385425.1 Severe acute respiratory syndrome coronavirus 2 isolate SARS-CoV-2/human/USA/CA-CZB046/2020, cor	TTCACCGAGGCCACGCGGAGTACGATCGAGTGTACAGTGAA
MT385426.1 Severe acute respiratory syndrome coronavirus 2 isolate SARS-CoV-2/human/USA/CA-CZB098/2020, cor	TTCACCGAGGCCACGCGGAGTACGATCGAGTGTACAGTGAA
MT385427.1 Severe acute respiratory syndrome coronavirus 2 isolate SARS-CoV-2/human/USA/CA-CZB0136/2020, co	TTCACCGAGGCCACGCGGAGTACGATCGAGTGTACAGTGAA
MT385428.1 Severe acute respiratory syndrome coronavirus 2 isolate SARS-CoV-2/human/USA/CA-CZB085/2020, cor	TTCACCGAGGCCACGCGGAGTACGATCGAGTGTACAGTGAA
MT385429.1 Severe acute respiratory syndrome coronavirus 2 isolate SARS-CoV-2/human/USA/CA-CZB079/2020, cor	TTCACCGAGGCCACGCGGAGTACGATCGAGTGTACAGTGAA
MT385430.1 Severe acute respiratory syndrome coronavirus 2 isolate SARS-CoV-2/human/USA/CA-CZB095/2020, cor	TTCACCGAGGCCACGCGGAGTACGATCGAGTGTACAGTGAA
MT385431.1 Severe acute respiratory syndrome coronavirus 2 isolate SARS-CoV-2/human/USA/CA-CZB0458/2020, co	TTCACCGAGGCCACGCGGAGTACGATCGAGTGTACAGTGAA
MT385432.1 Severe acute respiratory syndrome coronavirus 2 isolate SARS-CoV-2/human/USA/CA-CZB091/2020, cor	TTCACCGAGGCCACGCGGAGTACGATCGAGTGTACAGTGAA
MT385433.1 Severe acute respiratory syndrome coronavirus 2 isolate SARS-CoV-2/human/USA/CA-CZB0122/2020, co	TTCACCGAGGCCACGCGGAGTACGATCGAGTGTACAGTGAA
MT385434.1 Severe acute respiratory syndrome coronavirus 2 isolate SARS-CoV-2/human/USA/CA-CZB0108/2020, co	TTCACCGAGGCCACGCGGAGTACGATCGAGTGTACAGTGAA
MT385435.1 Severe acute respiratory syndrome coronavirus 2 isolate SARS-CoV-2/human/USA/CA-CZB0147/2020, co	TTCACCGAGGCCACGCGGAGTACGATCGAGTGTACAGTGAA
MT385436.1 Severe acute respiratory syndrome coronavirus 2 isolate SARS-CoV-2/human/USA/CA-CZB016/2020, cor	TTCACCGAGGCCACGCGGAGTACGATCGAGTGTACAGTGAA
MT385437.1 Severe acute respiratory syndrome coronavirus 2 isolate SARS-CoV-2/human/USA/CA-CZB080/2020, cor	TTCACCGAGGCCACGCGGAGTACGATCGAGTGTACAGTGAA
MT385438.1 Severe acute respiratory syndrome coronavirus 2 isolate SARS-CoV-2/human/USA/CA-CZB0142/2020, co	TTCACCGAGGCCACGCGGAGTACGATCGAGTGTACAGTGAA
MT385440.1 Severe acute respiratory syndrome coronavirus 2 isolate SARS-CoV-2/human/USA/CA-CZB0709/2020, co	TTCACCGAGGCCACGCGGAGTACGATCGAGTGTACAGTGAA
MT385441.1 Severe acute respiratory syndrome coronavirus 2 isolate SARS-CoV-2/human/USA/CA-CZB030/2020, cor	TTCACCGAGGCCACGCGGAGTACGATCGAGTGTACAGTGAA
MT385442.1 Severe acute respiratory syndrome coronavirus 2 isolate SARS-CoV-2/human/USA/CA-CZB0750/2020, co	TTCACCGAGGCCACGCGGAGTACGATCGAGTGTACAGTGAA
MT385443.1 Severe acute respiratory syndrome coronavirus 2 isolate SARS-CoV-2/human/USA/CA-CZB078/2020, cor	TTCACCGAGGCCACGCGGAGTACGATCGAGTGTACAGTGAA
MT385444.1 Severe acute respiratory syndrome coronavirus 2 isolate SARS-CoV-2/human/USA/CA-CZB0721/2020, co	TTCACCGAGGCCACGCGGAGTACGATCGAGTGTACAGTGAA
MT385445.1 Severe acute respiratory syndrome coronavirus 2 isolate SARS-CoV-2/human/USA/CA-CZB045/2020, cor	TTCACCGAGGCCACGCGGAGTACGATCGAGTGTACAGTGAA
MT385446.1 Severe acute respiratory syndrome coronavirus 2 isolate SARS-CoV-2/human/USA/CA-CZB0713/2020, co	TTCACCGAGGCCACGCGGAGTACGATCGAGTGTACAGTGAA
MT385447.1 Severe acute respiratory syndrome coronavirus 2 isolate SARS-CoV-2/human/USA/CA-CZB0740/2020, co	TTCACCGAGGCCACGCGGAGTACGATCGAGTGTACAGTGAA
MT385448.1 Severe acute respiratory syndrome coronavirus 2 isolate SARS-CoV-2/human/USA/CA-CZB0705/2020, co	TTCACCGAGGCCACGCGGAGTACGATCGAGTGTACAGTGAA
MT385449.1 Severe acute respiratory syndrome coronavirus 2 isolate SARS-CoV-2/human/USA/CA-CZB0737/2020, co	TTCACCGAGGCCACGCGGAGTACGATCGAGTGTACAGTGAA
MT385450.1 Severe acute respiratory syndrome coronavirus 2 isolate SARS-CoV-2/human/USA/CA-CZB0731/2020, co	TTCACCGAGGCCACGCGGAGTACGATCGAGTGTACAGTGAA

MT385451.1 Severe acute respiratory syndrome coronavirus 2 isolate SARS-CoV-2/human/USA/CA-CZB0137/2020, co	TTCACCGAGGCCACGCGGAGTACGATCGAGTGTACAGTGAA
MT385452.1 Severe acute respiratory syndrome coronavirus 2 isolate SARS-CoV-2/human/USA/CA-CZB0746/2020, co	TTCACCGAGGCCACGCGGAGTACGATCGAGTGTACAGTGAA
MT385454.1 Severe acute respiratory syndrome coronavirus 2 isolate SARS-CoV-2/human/USA/CA-CZB072/2020, com	TTCACCGAGGCCACGCGGAGTACGATCGAGTGTACAGTGAA
MT385455.1 Severe acute respiratory syndrome coronavirus 2 isolate SARS-CoV-2/human/USA/CA-CZB0139/2020, co	TTCACCGAGGCCACGCGGAGTACGATCGAGTGTACAGTGAA
MT385456.1 Severe acute respiratory syndrome coronavirus 2 isolate SARS-CoV-2/human/USA/CA-CZB082/2020, com	TTCACCGAGGCCACGCGGAGTACGATCGAGTGTACAGTGAA
MT385457.1 Severe acute respiratory syndrome coronavirus 2 isolate SARS-CoV-2/human/USA/CA-CZB086/2020, com	TTCACCGAGGCCACGCGGAGTACGATCGAGTGTACAGTGAA
MT385458.1 Severe acute respiratory syndrome coronavirus 2 isolate SARS-CoV-2/human/USA/CA-CZB0706/2020, co	TTCACCGAGGCCACGCGGAGTACGATCGAGTGTACAGTGAA
MT385459.1 Severe acute respiratory syndrome coronavirus 2 isolate SARS-CoV-2/human/USA/CA-CZB042/2020, com	TTCACCGAGGCCACGCGGAGTACGATCGAGTGTACAGTGAA
MT385460.1 Severe acute respiratory syndrome coronavirus 2 isolate SARS-CoV-2/human/USA/CA-CZB0107/2020, co	TTCACCGAGGCCACGCGGAGTACGATCGAGTGTACAGTGAA
MT385461.1 Severe acute respiratory syndrome coronavirus 2 isolate SARS-CoV-2/human/USA/CA-CZB09/2020, com	TTCACCGAGGCCACGCGGAGTACGATCGAGTGTACAGTGAA
MT385462.1 Severe acute respiratory syndrome coronavirus 2 isolate SARS-CoV-2/human/USA/CA-CZB0707/2020, co	TTCACCGAGGCCACGCGGAGTACGATCGAGTGTACAGTGAA
MT385463.1 Severe acute respiratory syndrome coronavirus 2 isolate SARS-CoV-2/human/USA/CA-CZB036/2020, com	TTCACCGAGGCCACGCGGAGTACGATCGAGTGTACAGTGAA
MT385464.1 Severe acute respiratory syndrome coronavirus 2 isolate SARS-CoV-2/human/USA/CA-CZB0701/2020, co	TTCACCGAGGCCACGCGGAGTACGATCGAGTGTACAGTGAA
MT385465.1 Severe acute respiratory syndrome coronavirus 2 isolate SARS-CoV-2/human/USA/CA-CZB096/2020, com	TTCACCGAGGCCACGCGGAGTACGATCGAGTGTACAGTGAA
MT385466.1 Severe acute respiratory syndrome coronavirus 2 isolate SARS-CoV-2/human/USA/CA-CZB0710/2020, co	TTCACCGAGGCCACGCGGAGTACGATCGAGTGTACAGTGAA
MT385467.1 Severe acute respiratory syndrome coronavirus 2 isolate SARS-CoV-2/human/USA/CA-CZB0156/2020, co	TTCACCGAGGCCACGCGGAGTACGATCGAGTGTACAGTGAA
MT385468.1 Severe acute respiratory syndrome coronavirus 2 isolate SARS-CoV-2/human/USA/CA-CZB0158/2020, co	TTCACCGAGGCCACGCGGAGTACGATCGAGTGTACAGTGAA
MT385469.1 Severe acute respiratory syndrome coronavirus 2 isolate SARS-CoV-2/human/USA/CA-CZB039/2020, com	TTCACCGAGGCCACGCGGAGTACGATCGAGTGTACAGTGAA
MT385470.1 Severe acute respiratory syndrome coronavirus 2 isolate SARS-CoV-2/human/USA/CA-CZB0125/2020, co	TTCACCGAGGCCACGCGGAGTACGATCGAGTGTACAGTGAA
MT385471.1 Severe acute respiratory syndrome coronavirus 2 isolate SARS-CoV-2/human/USA/CA-CZB0154/2020, co	TTCACCGAGGCCACGCGGAGTACGATCGAGTGTACAGTGAA
MT385472.1 Severe acute respiratory syndrome coronavirus 2 isolate SARS-CoV-2/human/USA/CA-CZB043/2020, com	TTCACCGAGGCCACGCGGAGTACGATCGAGTGTACAGTGAA
MT385473.1 Severe acute respiratory syndrome coronavirus 2 isolate SARS-CoV-2/human/USA/CA-CZB090/2020, com	TTCACCGAGGCCACGCGGAGTACGATCGAGTGTACAGTGAA
MT385474.1 Severe acute respiratory syndrome coronavirus 2 isolate SARS-CoV-2/human/USA/CA-CZB089/2020, com	TTCACCGAGGCCACGCGGAGTACGATCGAGTGTACAGTGAA
MT385475.1 Severe acute respiratory syndrome coronavirus 2 isolate SARS-CoV-2/human/USA/CA-CZB0741/2020, co	TTCACCGAGGCCACGCGGAGTACGATCGAGTGTACAGTGAA
MT385476.1 Severe acute respiratory syndrome coronavirus 2 isolate SARS-CoV-2/human/USA/CA-CZB0714/2020, co	TTCACCGAGGCCACGCGGAGTACGATCGAGTGTACAGTGAA
MT385477.1 Severe acute respiratory syndrome coronavirus 2 isolate SARS-CoV-2/human/USA/CA-CZB0167/2020, co	TTCACCGAGGCCACGCGGAGTACGATCGAGTGTACAGTGAA
MT385478.1 Severe acute respiratory syndrome coronavirus 2 isolate SARS-CoV-2/human/USA/CA-CZB0141/2020, co	TTCACCGAGGCCACGCGGAGTACGATCGAGTGTACAGTGAA
MT385479.1 Severe acute respiratory syndrome coronavirus 2 isolate SARS-CoV-2/human/USA/CA-CZB0726/2020, co	TTCACCGAGGCCACGCGGAGTACGATCGAGTGTACAGTGAA
MT385480.1 Severe acute respiratory syndrome coronavirus 2 isolate SARS-CoV-2/human/USA/CA-CZB0101/2020 OR	TTCACCGAGGCCACGCGGAGTACGATCGAGTGTACAGTGAA
MT385481.1 Severe acute respiratory syndrome coronavirus 2 isolate SARS-CoV-2/human/USA/CA-CZB076/2020 ORF	TTCACCGAGGCCACGCGGAGTACGATCGAGTGTACAGTGAA
MT385482.1 Severe acute respiratory syndrome coronavirus 2 isolate SARS-CoV-2/human/USA/CA-CZB0114/2020, co	TTCACCGAGGCCACGCGGAGTACGATCGAGTGTACAGTGAA
MT385484.1 Severe acute respiratory syndrome coronavirus 2 isolate SARS-CoV-2/human/USA/CA-CZB0725/2020 OR	TTCACCGAGGCCACGCGGAGTACGATCGAGTGTACAGTGAA
MT385485.1 Severe acute respiratory syndrome coronavirus 2 isolate SARS-CoV-2/human/USA/CA-CZB0151/2020, co	TTCACCGAGGCCACGCGGAGTACGATCGAGTGTACAGTGAA

MT385486.1 Severe acute respiratory syndrome coronavirus 2 isolate SARS-CoV-2/human/USA/CA-CZB0145/2020 OF	TTCACCGAGGCCACGCGGAGTACGATCGAGTGTACAGTGAA
MT385487.1 Severe acute respiratory syndrome coronavirus 2 isolate SARS-CoV-2/human/USA/CA-CZB011/2020 ORF	TTCACCGAGGCCACGCGGAGTACGATCGAGTGTACAGTGAA
MT385488.1 Severe acute respiratory syndrome coronavirus 2 isolate SARS-CoV-2/human/USA/CA-CZB040/2020 ORF	TTCACCGAGGCCACGCGGAGTACGATCGAGTGTACAGTGAA
MT385492.1 Severe acute respiratory syndrome coronavirus 2 isolate SARS-CoV-2/human/USA/CA-CZB027/2020 ORF	TTCACCGAGGCCACGCGGAGTACGATCGAGTGTACAGTGAA
MT385495.1 Severe acute respiratory syndrome coronavirus 2 isolate SARS-CoV-2/human/USA/CA-CZB099/2020 ORF	TTCACCGAGGCCACGCGGAGTACGATCGAGTGTACAGTGAA
MT385497.1 Severe acute respiratory syndrome coronavirus 2 isolate SARS-CoV-2/human/USA/CA-CZB017/2020 ORF	TTCACCGAGGCCACGCGGAGTACGATCGAGTGTACAGTGAA
MT394528.1 Severe acute respiratory syndrome coronavirus 2 isolate SARS-CoV-2/human/USA/CA-CZB-59X002/2020	TTCACCGAGGCCACGCGGAGTACGATCGAGTGTACAGTGAA
MT394529.1 Severe acute respiratory syndrome coronavirus 2 isolate SARS-CoV-2/human/USA/CA-CZB-59X001/2020	TTCACCGAGGCCACGCGGAGTACGATCGAGTGTACAGTGAA
MT394530.1 Severe acute respiratory syndrome coronavirus 2 isolate SARS-CoV-2/human/USA/CA-CZB-59X004/2020	TTCACCGAGGCCACGCGGAGTACGATCGAGTGTACAGTGAA
MT394531.1 Severe acute respiratory syndrome coronavirus 2 isolate SARS-CoV-2/human/USA/CA-CZB-59X003/2020	TTCACCGAGGCCACGCGGAGTACGATCGAGTGTACAGTGAA
MT396241.1 Severe acute respiratory syndrome coronavirus 2 isolate SARS-CoV-2/human/CHN/YN-0306-466/2020, c	TTCACCGAGGCCACGCGGAGTACGATCGAGTGTACAGTGAA
MT396242.1 Severe acute respiratory syndrome coronavirus 2 isolate SARS-CoV-2/human/IND/nimh-0182/2020 ORF	TTCACCGAGGCCACGCGGAGTACGATCGAGTGTACAGTGAA
MT396243.1 Severe acute respiratory syndrome coronavirus 2 isolate SARS-CoV-2/human/IND/nimh-0130/2020 ORF	TTCACCGAGGCCACGCGGAGTACGATCGAGTGTACAGTGAA
MT396244.1 Severe acute respiratory syndrome coronavirus 2 isolate SARS-CoV-2/human/IND/nimh-1071/2020 ORF	TTCACCGAGGCCACGCGGAGTACGATCGAGTGTACAGTGAA
MT396245.1 Severe acute respiratory syndrome coronavirus 2 isolate SARS-CoV-2/human/IND/nimh-0351/2020 ORF	TTCACCGAGGCCACGCGGAGTACGATCGAGTGTACAGTGAA
MT396246.1 Severe acute respiratory syndrome coronavirus 2 isolate SARS-CoV-2/human/IND/nimh-0318/2020 ORF	TTCACCGAGGCCACGCGGAGTACGATCGAGTGTACAGTGAA
MT396247.1 Severe acute respiratory syndrome coronavirus 2 isolate SARS-CoV-2/human/IND/nimh-0113/2020 ORF	TTCACCGAGGCCACGCGGAGTACGATCGAGTGTACAGTGAA
MT396248.1 Severe acute respiratory syndrome coronavirus 2 isolate SARS-CoV-2/human/IND/nimh-0996/2020 ORF	TTCACCGAGGCCACGCGGAGTACGATCGAGTGTACAGTGAA
MT396266.1 Severe acute respiratory syndrome coronavirus 2 isolate SARS-CoV-2/mink/NLD/1/2020, complete geno	TTCACCGAGGCCACGCGGAGTACGATCGAGTGTACAGTGAA
MT407649.1 Severe acute respiratory syndrome coronavirus 2 isolate SARS-CoV-2/human/CHN/CN1/2020, complete	TTCACCGAGGCCACGCGGAGTACGATCGAGTGTACAGTGAA
MT407650.1 Severe acute respiratory syndrome coronavirus 2 isolate SARS-CoV-2/human/CHN/CN2/2020, complete	TTCACCGAGGCCACGCGGAGTACGATCGAGTGTACAGTGAA
MT407651.1 Severe acute respiratory syndrome coronavirus 2 isolate SARS-CoV-2/human/CHN/CN5/2020, complete	TTCACCGAGGCCACGCGGAGTACGATCGAGTGTACAGTGAA
MT407652.1 Severe acute respiratory syndrome coronavirus 2 isolate SARS-CoV-2/human/CHN/CN3/2020, complete	TTCACCGAGGCCACGCGGAGTACGATCGAGTGTACAGTGAA
MT407653.1 Severe acute respiratory syndrome coronavirus 2 isolate SARS-CoV-2/human/CHN/CN4/2020, complete	TTCACCGAGGCCACGCGGAGTACGATCGAGTGTACAGTGAA
MT407654.1 Severe acute respiratory syndrome coronavirus 2 isolate SARS-CoV-2/human/CHN/OS1/2020, complete	TTCACCGAGGCCACGCGGAGTACGATCGAGTGTACAGTGAA
MT407655.1 Severe acute respiratory syndrome coronavirus 2 isolate SARS-CoV-2/human/CHN/OS5/2020, complete	TTCACCGAGGCCACGCGGAGTACGATCGAGTGTACAGTGAA
MT407656.1 Severe acute respiratory syndrome coronavirus 2 isolate SARS-CoV-2/human/CHN/OS6/2020, complete	TTCACCGAGGCCACGCGGAGTACGATCGAGTGTACAGTGAA
MT407658.1 Severe acute respiratory syndrome coronavirus 2 isolate SARS-CoV-2/human/CHN/OS2/2020, complete	TTCACCGAGGCCACGCGGAGTACGATCGAGTGTACAGTGAA
MT407659.1 Severe acute respiratory syndrome coronavirus 2 isolate SARS-CoV-2/human/CHN/OS4/2020, complete	TTCACCGAGGCCACGCGGAGTACGATCGAGTGTACAGTGAA
MT412134.1 Severe acute respiratory syndrome coronavirus 2 isolate SARS-CoV-2/human/CHN/HN03/2020, complet	TTCACCGAGGCCACGCGGAGTACGATCGAGTGTACAGTGAA
MT412135.1 Severe acute respiratory syndrome coronavirus 2 isolate SARS-CoV-2/human/USA/MI-MDHHS-SC20010/	TTCACCGAGGCCACGCGGAGTACGATCGAGTGTACAGTGAA
MT412136.1 Severe acute respiratory syndrome coronavirus 2 isolate SARS-CoV-2/human/USA/MI-MDHHS-SC20011/	TTCACCGAGGCCACGCGGAGTACGATCGAGTGTACAGTGAA
MT412138.1 Severe acute respiratory syndrome coronavirus 2 isolate SARS-CoV-2/human/USA/MI-MDHHS-SC20013/	TTCACCGAGGCCACGCGGAGTACGATCGAGTGTACAGTGAA

MT412139.1 Severe acute respiratory syndrome coronavirus 2 isolate SARS-CoV-2/human/USA/MI-MDHHS-SC20014/	TTCACCGAGGCCACGCGGAGTACGATCGAGTGTACAGTGAA
MT412141.1 Severe acute respiratory syndrome coronavirus 2 isolate SARS-CoV-2/human/USA/MI-MDHHS-SC20016/	TTCACCGAGGCCACGCGGAGTACGATCGAGTGTACAGTGAA
MT412142.1 Severe acute respiratory syndrome coronavirus 2 isolate SARS-CoV-2/human/USA/MI-MDHHS-SC20017/	TTCACCGAGGCCACGCGGAGTACGATCGAGTGTACAGTGAA
MT412143.1 Severe acute respiratory syndrome coronavirus 2 isolate SARS-CoV-2/human/USA/MI-MDHHS-SC20018/	TTCACCGAGGCCACGCGGAGTACGATCGAGTGTACAGTGAA
MT412146.1 Severe acute respiratory syndrome coronavirus 2 isolate SARS-CoV-2/human/USA/MI-MDHHS-SC20021/	TTCACCGAGGCCACGCGGAGTACGATCGAGTGTACAGTGAA
MT412147.1 Severe acute respiratory syndrome coronavirus 2 isolate SARS-CoV-2/human/USA/MI-MDHHS-SC20022/	TTCACCGAGGCCACGCGGAGTACGATCGAGTGTACAGTGAA
MT412149.1 Severe acute respiratory syndrome coronavirus 2 isolate SARS-CoV-2/human/USA/MI-MDHHS-SC20024/	TTCACCGAGGCCACGCGGAGTACGATCGAGTGTACAGTGAA
MT412150.1 Severe acute respiratory syndrome coronavirus 2 isolate SARS-CoV-2/human/USA/MI-MDHHS-SC20025/	TTCACCGAGGCCACGCGGAGTACGATCGAGTGTACAGTGAA
MT412151.1 Severe acute respiratory syndrome coronavirus 2 isolate SARS-CoV-2/human/USA/MI-MDHHS-SC20026/	TTCACCGAGGCCACGCGGAGTACGATCGAGTGTACAGTGAA
MT412152.1 Severe acute respiratory syndrome coronavirus 2 isolate SARS-CoV-2/human/USA/MI-MDHHS-SC20027/	TTCACCGAGGCCACGCGGAGTACGATCGAGTGTACAGTGAA
MT412153.1 Severe acute respiratory syndrome coronavirus 2 isolate SARS-CoV-2/human/USA/MI-MDHHS-SC20028/	TTCACCGAGGCCACGCGGAGTACGATCGAGTGTACAGTGAA
MT412154.1 Severe acute respiratory syndrome coronavirus 2 isolate SARS-CoV-2/human/USA/MI-MDHHS-SC20029/	TTCACCGAGGCCACGCGGAGTACGATCGAGTGTACAGTGAA
MT412155.1 Severe acute respiratory syndrome coronavirus 2 isolate SARS-CoV-2/human/USA/MI-MDHHS-SC20030/	TTCACCGAGGCCACGCGGAGTACGATCGAGTGTACAGTGAA
MT412156.1 Severe acute respiratory syndrome coronavirus 2 isolate SARS-CoV-2/human/USA/MI-MDHHS-SC20031/	TTCACCGAGGCCACGCGGAGTACGATCGAGTGTACAGTGAA
MT412157.1 Severe acute respiratory syndrome coronavirus 2 isolate SARS-CoV-2/human/USA/MI-MDHHS-SC20032/	TTCACCGAGGCCACGCGGAGTACGATCGAGTGTACAGTGAA
MT412158.1 Severe acute respiratory syndrome coronavirus 2 isolate SARS-CoV-2/human/USA/MI-MDHHS-SC20033/	TTCACCGAGGCCACGCGGAGTACGATCGAGTGTACAGTGAA
MT412160.1 Severe acute respiratory syndrome coronavirus 2 isolate SARS-CoV-2/human/USA/MI-MDHHS-SC20035/	TTCACCGAGGCCACGCGGAGTACGATCGAGTGTACAGTGAA
MT412161.1 Severe acute respiratory syndrome coronavirus 2 isolate SARS-CoV-2/human/USA/MI-MDHHS-SC20036/	TTCACCGAGGCCACGCGGAGTACGATCGAGTGTACAGTGAA
MT412162.1 Severe acute respiratory syndrome coronavirus 2 isolate SARS-CoV-2/human/USA/MI-MDHHS-SC20037/	TTCACCGAGGCCACGCGGAGTACGATCGAGTGTACAGTGAA
MT412163.1 Severe acute respiratory syndrome coronavirus 2 isolate SARS-CoV-2/human/USA/MI-MDHHS-SC20038/	TTCACCGAGGCCACGCGGAGTACGATCGAGTGTACAGTGAA
MT412165.1 Severe acute respiratory syndrome coronavirus 2 isolate SARS-CoV-2/human/USA/MI-MDHHS-SC20040/	TTCACCGAGGCCACGCGGAGTACGATCGAGTGTACAGTGAA
MT412166.1 Severe acute respiratory syndrome coronavirus 2 isolate SARS-CoV-2/human/USA/MI-MDHHS-SC20041/	TTCACCGAGGCCACGCGGAGTACGATCGAGTGTACAGTGAA
MT412167.1 Severe acute respiratory syndrome coronavirus 2 isolate SARS-CoV-2/human/USA/MI-MDHHS-SC20042/	TTCACCGAGGCCACGCGGAGTACGATCGAGTGTACAGTGAA
MT412168.1 Severe acute respiratory syndrome coronavirus 2 isolate SARS-CoV-2/human/USA/MI-MDHHS-SC20043/	TTCACCGAGGCCACGCGGAGTACGATCGAGTGTACAGTGAA
MT412169.1 Severe acute respiratory syndrome coronavirus 2 isolate SARS-CoV-2/human/USA/MI-MDHHS-SC20044/	TTCACCGAGGCCACGCGGAGTACGATCGAGTGTACAGTGAA
MT412170.1 Severe acute respiratory syndrome coronavirus 2 isolate SARS-CoV-2/human/USA/MI-MDHHS-SC20045/	TTCACCGAGGCCACGCGGAGTACGATCGAGTGTACAGTGAA
MT412173.1 Severe acute respiratory syndrome coronavirus 2 isolate SARS-CoV-2/human/USA/MI-MDHHS-SC20048/	TTCACCGAGGCCACGCGGAGTACGATCGAGTGTACAGTGAA
MT412175.1 Severe acute respiratory syndrome coronavirus 2 isolate SARS-CoV-2/human/USA/MI-MDHHS-SC20050/	TTCACCGAGGCCACGCGGAGTACGATCGAGTGTACAGTGAA
MT412176.1 Severe acute respiratory syndrome coronavirus 2 isolate SARS-CoV-2/human/USA/MI-MDHHS-SC20051/	TTCACCGAGGCCACGCGGAGTACGATCGAGTGTACAGTGAA
MT412178.1 Severe acute respiratory syndrome coronavirus 2 isolate SARS-CoV-2/human/USA/MI-MDHHS-SC20053/	TTCACCGAGGCCACGCGGAGTACGATCGAGTGTACAGTGAA
MT412179.1 Severe acute respiratory syndrome coronavirus 2 isolate SARS-CoV-2/human/USA/MI-MDHHS-SC20054/	TTCACCGAGGCCACGCGGAGTACGATCGAGTGTACAGTGAA
MT412180.1 Severe acute respiratory syndrome coronavirus 2 isolate SARS-CoV-2/human/USA/MI-MDHHS-SC20055/	TTCACCGAGGCCACGCGGAGTACGATCGAGTGTACAGTGAA
MT412181.1 Severe acute respiratory syndrome coronavirus 2 isolate SARS-CoV-2/human/USA/MI-MDHHS-SC20056/	TTCACCGAGGCCACGCGGAGTACGATCGAGTGTACAGTGAA

MT412183.1 Severe acute respiratory syndrome coronavirus 2 isolate SARS-CoV-2/human/USA/MI-MDHHS-SC20058/	TTCACCGAGGCCACGCGGAGTACGATCGAGTGTACAGTGAA
MT412184.1 Severe acute respiratory syndrome coronavirus 2 isolate SARS-CoV-2/human/USA/MI-MDHHS-SC20059/	TTCACCGAGGCCACGCGGAGTACGATCGAGTGTACAGTGAA
MT412185.1 Severe acute respiratory syndrome coronavirus 2 isolate SARS-CoV-2/human/USA/MI-MDHHS-SC20060/	TTCACCGAGGCCACGCGGAGTACGATCGAGTGTACAGTGAA
MT412186.1 Severe acute respiratory syndrome coronavirus 2 isolate SARS-CoV-2/human/USA/MI-MDHHS-SC20061/	TTCACCGAGGCCACGCGGAGTACGATCGAGTGTACAGTGAA
MT412189.1 Severe acute respiratory syndrome coronavirus 2 isolate SARS-CoV-2/human/USA/MI-MDHHS-SC20064/	TTCACCGAGGCCACGCGGAGTACGATCGAGTGTACAGTGAA
MT412190.1 Severe acute respiratory syndrome coronavirus 2 isolate SARS-CoV-2/human/USA/MI-MDHHS-SC20065/	TTCACCGAGGCCACGCGGAGTACGATCGAGTGTACAGTGAA
MT412191.1 Severe acute respiratory syndrome coronavirus 2 isolate SARS-CoV-2/human/USA/MI-MDHHS-SC20066/	TTCACCGAGGCCACGCGGAGTACGATCGAGTGTACAGTGAA
MT412192.1 Severe acute respiratory syndrome coronavirus 2 isolate SARS-CoV-2/human/USA/MI-MDHHS-SC20067/	TTCACCGAGGCCACGCGGAGTACGATCGAGTGTACAGTGAA
MT412193.1 Severe acute respiratory syndrome coronavirus 2 isolate SARS-CoV-2/human/USA/MI-MDHHS-SC20068/	TTCACCGAGGCCACGCGGAGTACGATCGAGTGTACAGTGAA
MT412194.1 Severe acute respiratory syndrome coronavirus 2 isolate SARS-CoV-2/human/USA/MI-MDHHS-SC20069/	TTCACCGAGGCCACGCGGAGTACGATCGAGTGTACAGTGAA
MT412195.1 Severe acute respiratory syndrome coronavirus 2 isolate SARS-CoV-2/human/USA/MI-MDHHS-SC20070/	TTCACCGAGGCCACGCGGAGTACGATCGAGTGTACAGTGAA
MT412196.1 Severe acute respiratory syndrome coronavirus 2 isolate SARS-CoV-2/human/USA/MI-MDHHS-SC20071/	TTCACCGAGGCCACGCGGAGTACGATCGAGTGTACAGTGAA
MT412197.1 Severe acute respiratory syndrome coronavirus 2 isolate SARS-CoV-2/human/USA/MI-MDHHS-SC20072/	TTCACCGAGGCCACGCGGAGTACGATCGAGTGTACAGTGAA
MT412198.1 Severe acute respiratory syndrome coronavirus 2 isolate SARS-CoV-2/human/USA/MI-MDHHS-SC20073/	TTCACCGAGGCCACGCGGAGTACGATCGAGTGTACAGTGAA
MT412199.1 Severe acute respiratory syndrome coronavirus 2 isolate SARS-CoV-2/human/USA/MI-MDHHS-SC20074/	TTCACCGAGGCCACGCGGAGTACGATCGAGTGTACAGTGAA
MT412200.1 Severe acute respiratory syndrome coronavirus 2 isolate SARS-CoV-2/human/USA/MI-MDHHS-SC20075/	TTCACCGAGGCCACGCGGAGTACGATCGAGTGTACAGTGAA
MT412201.1 Severe acute respiratory syndrome coronavirus 2 isolate SARS-CoV-2/human/USA/MI-MDHHS-SC20076/	TTCACCGAGGCCACGCGGAGTACGATCGAGTGTACAGTGAA
MT412202.1 Severe acute respiratory syndrome coronavirus 2 isolate SARS-CoV-2/human/USA/MI-MDHHS-SC20077/	TTCACCGAGGCCACGCGGAGTACGATCGAGTGTACAGTGAA
MT412203.1 Severe acute respiratory syndrome coronavirus 2 isolate SARS-CoV-2/human/USA/MI-MDHHS-SC20078/	TTCACCGAGGCCACGCGGAGTACGATCGAGTGTACAGTGAA
MT412204.1 Severe acute respiratory syndrome coronavirus 2 isolate SARS-CoV-2/human/USA/MI-MDHHS-SC20079/	TTCACCGAGGCCACGCGGAGTACGATCGAGTGTACAGTGAA
MT412205.1 Severe acute respiratory syndrome coronavirus 2 isolate SARS-CoV-2/human/USA/MI-MDHHS-SC20080/	TTCACCGAGGCCACGCGGAGTACGATCGAGTGTACAGTGAA
MT412206.1 Severe acute respiratory syndrome coronavirus 2 isolate SARS-CoV-2/human/USA/MI-MDHHS-SC20081/	TTCACCGAGGCCACGCGGAGTACGATCGAGTGTACAGTGAA
MT412207.1 Severe acute respiratory syndrome coronavirus 2 isolate SARS-CoV-2/human/USA/MI-MDHHS-SC20082/	TTCACCGAGGCCACGCGGAGTACGATCGAGTGTACAGTGAA
MT412208.1 Severe acute respiratory syndrome coronavirus 2 isolate SARS-CoV-2/human/USA/MI-MDHHS-SC20083/	TTCACCGAGGCCACGCGGAGTACGATCGAGTGTACAGTGAA
MT412209.1 Severe acute respiratory syndrome coronavirus 2 isolate SARS-CoV-2/human/USA/MI-MDHHS-SC20084/	TTCACCGAGGCCACGCGGAGTACGATCGAGTGTACAGTGAA
MT412210.1 Severe acute respiratory syndrome coronavirus 2 isolate SARS-CoV-2/human/USA/MI-MDHHS-SC20085/	TTCACCGAGGCCACGCGGAGTACGATCGAGTGTACAGTGAA
MT412211.1 Severe acute respiratory syndrome coronavirus 2 isolate SARS-CoV-2/human/USA/MI-MDHHS-SC20086/	TTCACCGAGGCCACGCGGAGTACGATCGAGTGTACAGTGAA
MT412212.1 Severe acute respiratory syndrome coronavirus 2 isolate SARS-CoV-2/human/USA/MI-MDHHS-SC20087/	TTCACCGAGGCCACGCGGAGTACGATCGAGTGTACAGTGAA
MT412213.1 Severe acute respiratory syndrome coronavirus 2 isolate SARS-CoV-2/human/USA/MI-MDHHS-SC20088/	TTCACCGAGGCCACGCGGAGTACGATCGAGTGTACAGTGAA
MT412214.1 Severe acute respiratory syndrome coronavirus 2 isolate SARS-CoV-2/human/USA/MI-MDHHS-SC20089/	TTCACCGAGGCCACGCGGAGTACGATCGAGTGTACAGTGAA
MT412215.1 Severe acute respiratory syndrome coronavirus 2 isolate SARS-CoV-2/human/USA/MI-MDHHS-SC20090/	TTCACCGAGGCCACGCGGAGTACGATCGAGTGTACAGTGAA
MT412216.1 Severe acute respiratory syndrome coronavirus 2 isolate SARS-CoV-2/human/USA/MI-MDHHS-SC20091/	TTCACCGAGGCCACGCGGAGTACGATCGAGTGTACAGTGAA
MT412217.1 Severe acute respiratory syndrome coronavirus 2 isolate SARS-CoV-2/human/USA/MI-MDHHS-SC20092/	TTCACCGAGGCCACGCGGAGTACGATCGAGTGTACAGTGAA

MT412218.1 Severe acute respiratory syndrome coronavirus 2 isolate SARS-CoV-2/human/USA/MI-MDHHS-SC20093/	TTCACCGAGGCCACGCGGAGTACGATCGAGTGTACAGTGAA
MT412219.1 Severe acute respiratory syndrome coronavirus 2 isolate SARS-CoV-2/human/USA/MI-MDHHS-SC20094/	TTCACCGAGGCCACGCGGAGTACGATCGAGTGTACAGTGAA
MT412220.1 Severe acute respiratory syndrome coronavirus 2 isolate SARS-CoV-2/human/USA/MI-MDHHS-SC20095/	TTCACCGAGGCCACGCGGAGTACGATCGAGTGTACAGTGAA
MT412221.1 Severe acute respiratory syndrome coronavirus 2 isolate SARS-CoV-2/human/USA/MI-MDHHS-SC20096/	TTCACCGAGGCCACGCGGAGTACGATCGAGTGTACAGTGAA
MT412222.1 Severe acute respiratory syndrome coronavirus 2 isolate SARS-CoV-2/human/USA/MI-MDHHS-SC20097/	TTCACCGAGGCCACGCGGAGTACGATCGAGTGTACAGTGAA
MT412223.1 Severe acute respiratory syndrome coronavirus 2 isolate SARS-CoV-2/human/USA/MI-MDHHS-SC20098/	TTCACCGAGGCCACGCGGAGTACGATCGAGTGTACAGTGAA
MT412224.1 Severe acute respiratory syndrome coronavirus 2 isolate SARS-CoV-2/human/USA/MI-MDHHS-SC20099/	TTCACCGAGGCCACGCGGAGTACGATCGAGTGTACAGTGAA
MT412225.1 Severe acute respiratory syndrome coronavirus 2 isolate SARS-CoV-2/human/USA/WA-UW-5177/2020 C	TTCACCGAGGCCACGCGGAGTACGATCGAGTGTACAGTGAA
MT412226.1 Severe acute respiratory syndrome coronavirus 2 isolate SARS-CoV-2/human/USA/WA-UW-5178/2020, c	TTCACCGAGGCCACGCGGAGTACGATCGAGTGTACAGTGAA
MT412227.1 Severe acute respiratory syndrome coronavirus 2 isolate SARS-CoV-2/human/USA/WA-UW-5181/2020, c	TTCACCGAGGCCACGCGGAGTACGATCGAGTGTACAGTGAA
MT412228.1 Severe acute respiratory syndrome coronavirus 2 isolate SARS-CoV-2/human/USA/WA-UW-5182/2020, c	TTCACCGAGGCCACGCGGAGTACGATCGAGTGTACAGTGAA
MT412229.1 Severe acute respiratory syndrome coronavirus 2 isolate SARS-CoV-2/human/USA/WA-UW-5189/2020, c	TTCACCGAGGCCACGCGGAGTACGATCGAGTGTACAGTGAA
MT412230.1 Severe acute respiratory syndrome coronavirus 2 isolate SARS-CoV-2/human/USA/WA-UW-5190/2020, c	TTCACCGAGGCCACGCGGAGTACGATCGAGTGTACAGTGAA
MT412231.1 Severe acute respiratory syndrome coronavirus 2 isolate SARS-CoV-2/human/USA/WA-UW-5192/2020 C	TTCACCGAGGCCACGCGGAGTACGATCGAGTGTACAGTGAA
MT412232.1 Severe acute respiratory syndrome coronavirus 2 isolate SARS-CoV-2/human/USA/WA-UW-5194/2020 C	TTCACCGAGGCCACGCGGAGTACGATCGAGTGTACAGTGAA
MT412233.1 Severe acute respiratory syndrome coronavirus 2 isolate SARS-CoV-2/human/USA/UNKNOWN-UW-531/	TTCACCGAGGCCACGCGGAGTACGATCGAGTGTACAGTGAA
MT412234.1 Severe acute respiratory syndrome coronavirus 2 isolate SARS-CoV-2/human/USA/WA-UW-604/2020, co	TTCACCGAGGCCACGCGGAGTACGATCGAGTGTACAGTGAA
MT412235.1 Severe acute respiratory syndrome coronavirus 2 isolate SARS-CoV-2/human/USA/WA-UW-558/2020, co	TTCACCGAGGCCACGCGGAGTACGATCGAGTGTACAGTGAA
MT412236.1 Severe acute respiratory syndrome coronavirus 2 isolate SARS-CoV-2/human/USA/WA-UW-586/2020, co	TTCACCGAGGCCACGCGGAGTACGATCGAGTGTACAGTGAA
MT412237.1 Severe acute respiratory syndrome coronavirus 2 isolate SARS-CoV-2/human/USA/WA-UW-602/2020, co	TTCACCGAGGCCACGCGGAGTACGATCGAGTGTACAGTGAA
MT412238.1 Severe acute respiratory syndrome coronavirus 2 isolate SARS-CoV-2/human/USA/WA-UW-596/2020, co	TTCACCGAGGCCACGCGGAGTACGATCGAGTGTACAGTGAA
MT412239.1 Severe acute respiratory syndrome coronavirus 2 isolate SARS-CoV-2/human/USA/UNKNOWN-UW-547/	TTCACCGAGGCCACGCGGAGTACGATCGAGTGTACAGTGAA
MT412240.1 Severe acute respiratory syndrome coronavirus 2 isolate SARS-CoV-2/human/USA/WA-UW-600/2020, co	TTCACCGAGGCCACGCGGAGTACGATCGAGTGTACAGTGAA
MT412241.1 Severe acute respiratory syndrome coronavirus 2 isolate SARS-CoV-2/human/USA/WA-UW-555/2020, co	TTCACCGAGGCCACGCGGAGTACGATCGAGTGTACAGTGAA
MT412242.1 Severe acute respiratory syndrome coronavirus 2 isolate SARS-CoV-2/human/USA/UNKNOWN-UW-526/	TTCACCGAGGCCACGCGGAGTACGATCGAGTGTACAGTGAA
MT412243.1 Severe acute respiratory syndrome coronavirus 2 isolate SARS-CoV-2/human/USA/UNKNOWN-UW-581/	TTCACCGAGGCCACGCGGAGTACGATCGAGTGTACAGTGAA
MT412245.1 Severe acute respiratory syndrome coronavirus 2 isolate SARS-CoV-2/human/USA/UNKNOWN-UW-536/	TTCACCGAGGCCACGCGGAGTACGATCGAGTGTACAGTGAA
MT412246.1 Severe acute respiratory syndrome coronavirus 2 isolate SARS-CoV-2/human/USA/WA-UW-525/2020, co	TTCACCGAGGCCACGCGGAGTACGATCGAGTGTACAGTGAA
MT412247.1 Severe acute respiratory syndrome coronavirus 2 isolate SARS-CoV-2/human/USA/WA-UW-530/2020, co	TTCACCGAGGCCACGCGGAGTACGATCGAGTGTACAGTGAA
MT412248.1 Severe acute respiratory syndrome coronavirus 2 isolate SARS-CoV-2/human/USA/WA-UW-524/2020, co	TTCACCGAGGCCACGCGGAGTACGATCGAGTGTACAGTGAA
MT412249.1 Severe acute respiratory syndrome coronavirus 2 isolate SARS-CoV-2/human/USA/WA-UW-543/2020, co	TTCACCGAGGCCACGCGGAGTACGATCGAGTGTACAGTGAA
MT412250.1 Severe acute respiratory syndrome coronavirus 2 isolate SARS-CoV-2/human/USA/WA-UW-542/2020, co	TTCACCGAGGCCACGCGGAGTACGATCGAGTGTACAGTGAA
MT412251.1 Severe acute respiratory syndrome coronavirus 2 isolate SARS-CoV-2/human/USA/WA-UW-546/2020, co	TTCACCGAGGCCACGCGGAGTACGATCGAGTGTACAGTGAA

MT412252.1 Severe acute respiratory syndrome coronavirus 2 isolate SARS-CoV-2/human/USA/WA-UW-517/2020, c	TTCACCGAGGCCACGCGGAGTACGATCGAGTGTACAGTGAA
MT412253.1 Severe acute respiratory syndrome coronavirus 2 isolate SARS-CoV-2/human/USA/WA-UW-518/2020, c	TTCACCGAGGCCACGCGGAGTACGATCGAGTGTACAGTGAA
MT412254.1 Severe acute respiratory syndrome coronavirus 2 isolate SARS-CoV-2/human/USA/UNKNOWN-UW-5199	TTCACCGAGGCCACGCGGAGTACGATCGAGTGTACAGTGAA
MT412256.1 Severe acute respiratory syndrome coronavirus 2 isolate SARS-CoV-2/human/USA/WA-UW-5203/2020,	TTCACCGAGGCCACGCGGAGTACGATCGAGTGTACAGTGAA
MT412257.1 Severe acute respiratory syndrome coronavirus 2 isolate SARS-CoV-2/human/USA/WA-UW-5205/2020,	TTCACCGAGGCCACGCGGAGTACGATCGAGTGTACAGTGAA
MT412258.1 Severe acute respiratory syndrome coronavirus 2 isolate SARS-CoV-2/human/USA/WA-UW-5359/2020,	TTCACCGAGGCCACGCGGAGTACGATCGAGTGTACAGTGAA
MT412259.1 Severe acute respiratory syndrome coronavirus 2 isolate SARS-CoV-2/human/USA/WA-UW-5364/2020,	TTCACCGAGGCCACGCGGAGTACGATCGAGTGTACAGTGAA
MT412260.1 Severe acute respiratory syndrome coronavirus 2 isolate SARS-CoV-2/human/USA/UNKNOWN-UW-5368	TTCACCGAGGCCACGCGGAGTACGATCGAGTGTACAGTGAA
MT412261.1 Severe acute respiratory syndrome coronavirus 2 isolate SARS-CoV-2/human/USA/WA-UW-5371/2020,	TTCACCGAGGCCACGCGGAGTACGATCGAGTGTACAGTGAA
MT412263.1 Severe acute respiratory syndrome coronavirus 2 isolate SARS-CoV-2/human/USA/WA-UW-5400/2020,	TTCACCGAGGCCACGCGGAGTACGATCGAGTGTACAGTGAA
MT412264.1 Severe acute respiratory syndrome coronavirus 2 isolate SARS-CoV-2/human/USA/CT-UW-5595/2020, c	TTCACCGAGGCCACGCGGAGTACGATCGAGTGTACAGTGAA
MT412265.1 Severe acute respiratory syndrome coronavirus 2 isolate SARS-CoV-2/human/USA/CT-UW-5599/2020 O	TTCACCGAGGCCACGCGGAGTACGATCGAGTGTACAGTGAA
MT412266.1 Severe acute respiratory syndrome coronavirus 2 isolate SARS-CoV-2/human/USA/UNKNOWN-UW-5605	TTCACCGAGGCCACGCGGAGTACGATCGAGTGTACAGTGAA
MT412267.1 Severe acute respiratory syndrome coronavirus 2 isolate SARS-CoV-2/human/USA/UNKNOWN-UW-5614	TTCACCGAGGCCACGCGGAGTACGATCGAGTGTACAGTGAA
MT412268.1 Severe acute respiratory syndrome coronavirus 2 isolate SARS-CoV-2/human/USA/WA-UW-5617/2020,	TTCACCGAGGCCACGCGGAGTACGATCGAGTGTACAGTGAA
MT412269.1 Severe acute respiratory syndrome coronavirus 2 isolate SARS-CoV-2/human/USA/WA-UW-5629/2020,	TTCACCGAGGCCACGCGGAGTACGATCGAGTGTACAGTGAA
MT412270.1 Severe acute respiratory syndrome coronavirus 2 isolate SARS-CoV-2/human/USA/WA-UW-5717/2020	TTCACCGAGGCCACGCGGAGTACGATCGAGTGTACAGTGAA
MT412272.1 Severe acute respiratory syndrome coronavirus 2 isolate SARS-CoV-2/human/USA/WA-UW-5737/2020,	TTCACCGAGGCCACGCGGAGTACGATCGAGTGTACAGTGAA
MT412273.1 Severe acute respiratory syndrome coronavirus 2 isolate SARS-CoV-2/human/USA/UNKNOWN-UW-5742	TTCACCGAGGCCACGCGGAGTACGATCGAGTGTACAGTGAA
MT412274.1 Severe acute respiratory syndrome coronavirus 2 isolate SARS-CoV-2/human/USA/CT-UW-5749/2020, c	TTCACCGAGGCCACGCGGAGTACGATCGAGTGTACAGTGAA
MT412275.1 Severe acute respiratory syndrome coronavirus 2 isolate SARS-CoV-2/human/USA/WA-UW-5750/2020,	TTCACCGAGGCCACGCGGAGTACGATCGAGTGTACAGTGAA
MT412276.1 Severe acute respiratory syndrome coronavirus 2 isolate SARS-CoV-2/human/USA/CT-UW-5754/2020, c	TTCACCGAGGCCACGCGGAGTACGATCGAGTGTACAGTGAA
MT412277.1 Severe acute respiratory syndrome coronavirus 2 isolate SARS-CoV-2/human/USA/WA-UW-5756/2020,	TTCACCGAGGCCACGCGGAGTACGATCGAGTGTACAGTGAA
MT412278.1 Severe acute respiratory syndrome coronavirus 2 isolate SARS-CoV-2/human/USA/CT-UW-5757/2020, c	TTCACCGAGGCCACGCGGAGTACGATCGAGTGTACAGTGAA
MT412279.1 Severe acute respiratory syndrome coronavirus 2 isolate SARS-CoV-2/human/USA/CT-UW-5763/2020, c	TTCACCGAGGCCACGCGGAGTACGATCGAGTGTACAGTGAA
MT412280.1 Severe acute respiratory syndrome coronavirus 2 isolate SARS-CoV-2/human/USA/CT-UW-5764/2020, c	TTCACCGAGGCCACGCGGAGTACGATCGAGTGTACAGTGAA
MT412281.1 Severe acute respiratory syndrome coronavirus 2 isolate SARS-CoV-2/human/USA/UNKNOWN-UW-5769	TTCACCGAGGCCACGCGGAGTACGATCGAGTGTACAGTGAA
MT412282.1 Severe acute respiratory syndrome coronavirus 2 isolate SARS-CoV-2/human/USA/CT-UW-5770/2020 O	TTCACCGAGGCCACGCGGAGTACGATCGAGTGTACAGTGAA
MT412283.1 Severe acute respiratory syndrome coronavirus 2 isolate SARS-CoV-2/human/USA/CT-UW-5772/2020, c	TTCACCGAGGCCACGCGGAGTACGATCGAGTGTACAGTGAA
MT412284.1 Severe acute respiratory syndrome coronavirus 2 isolate SARS-CoV-2/human/USA/CT-UW-5773/2020, c	TTCACCGAGGCCACGCGGAGTACGATCGAGTGTACAGTGAA
MT412285.1 Severe acute respiratory syndrome coronavirus 2 isolate SARS-CoV-2/human/USA/WA-UW-5879/2020,	TTCACCGAGGCCACGCGGAGTACGATCGAGTGTACAGTGAA
MT412286.1 Severe acute respiratory syndrome coronavirus 2 isolate SARS-CoV-2/human/USA/WA-UW-5884/2020,	TTCACCGAGGCCACGCGGAGTACGATCGAGTGTACAGTGAA
MT412287.1 Severe acute respiratory syndrome coronavirus 2 isolate SARS-CoV-2/human/USA/WA-UW-5911/2020,	TTCACCGAGGCCACGCGGAGTACGATCGAGTGTACAGTGAA

MT412288.1 Severe acute respiratory syndrome coronavirus 2 isolate SARS-CoV-2/human/USA/WA-UW-5932/2020,	TTCACCGAGGCCACGCGGAGTACGATCGAGTGTACAGTGAA
MT412289.1 Severe acute respiratory syndrome coronavirus 2 isolate SARS-CoV-2/human/USA/WA-UW-5947/2020,	TTCACCGAGGCCACGCGGAGTACGATCGAGTGTACAGTGAA
MT412291.1 Severe acute respiratory syndrome coronavirus 2 isolate SARS-CoV-2/human/USA/WA-UW-6164/2020,	TTCACCGAGGCCACGCGGAGTACGATCGAGTGTACAGTGAA
MT412293.1 Severe acute respiratory syndrome coronavirus 2 isolate SARS-CoV-2/human/USA/WA-UW-6173/2020,	TTCACCGAGGCCACGCGGAGTACGATCGAGTGTACAGTGAA
MT412294.1 Severe acute respiratory syndrome coronavirus 2 isolate SARS-CoV-2/human/USA/WA-UW-6178/2020,	TTCACCGAGGCCACGCGGAGTACGATCGAGTGTACAGTGAA
MT412295.1 Severe acute respiratory syndrome coronavirus 2 isolate SARS-CoV-2/human/USA/WA-UW-4226/2020,	TTCACCGAGGCCACGCGGAGTACGATCGAGTGTACAGTGAA
MT412296.1 Severe acute respiratory syndrome coronavirus 2 isolate SARS-CoV-2/human/USA/WA-UW-4243/2020,	TTCACCGAGGCCACGCGGAGTACGATCGAGTGTACAGTGAA
MT412297.1 Severe acute respiratory syndrome coronavirus 2 isolate SARS-CoV-2/human/USA/ID-UW-4255/2020, c	TTCACCGAGGCCACGCGGAGTACGATCGAGTGTACAGTGAA
MT412298.1 Severe acute respiratory syndrome coronavirus 2 isolate SARS-CoV-2/human/USA/WA-UW-4279/2020,	TTCACCGAGGCCACGCGGAGTACGATCGAGTGTACAGTGAA
MT412299.1 Severe acute respiratory syndrome coronavirus 2 isolate SARS-CoV-2/human/USA/ID-UW-4307/2020, c	TTCACCGAGGCCACGCGGAGTACGATCGAGTGTACAGTGAA
MT412300.1 Severe acute respiratory syndrome coronavirus 2 isolate SARS-CoV-2/human/USA/WA-UW-4338/2020,	TTCACCGAGGCCACGCGGAGTACGATCGAGTGTACAGTGAA
MT412301.1 Severe acute respiratory syndrome coronavirus 2 isolate SARS-CoV-2/human/USA/CT-UW-4343/2020, c	TTCACCGAGGCCACGCGGAGTACGATCGAGTGTACAGTGAA
MT412302.1 Severe acute respiratory syndrome coronavirus 2 isolate SARS-CoV-2/human/USA/CT-UW-4345/2020, c	TTCACCGAGGCCACGCGGAGTACGATCGAGTGTACAGTGAA
MT412303.1 Severe acute respiratory syndrome coronavirus 2 isolate SARS-CoV-2/human/USA/CT-UW-4348/2020, c	TTCACCGAGGCCACGCGGAGTACGATCGAGTGTACAGTGAA
MT412304.1 Severe acute respiratory syndrome coronavirus 2 isolate SARS-CoV-2/human/USA/ID-UW-4354/2020, c	TTCACCGAGGCCACGCGGAGTACGATCGAGTGTACAGTGAA
MT412306.1 Severe acute respiratory syndrome coronavirus 2 isolate SARS-CoV-2/human/USA/ID-UW-4402/2020, c	TTCACCGAGGCCACGCGGAGTACGATCGAGTGTACAGTGAA
MT412307.1 Severe acute respiratory syndrome coronavirus 2 isolate SARS-CoV-2/human/USA/OR-UW-4410/2020, c	TTCACCGAGGCCACGCGGAGTACGATCGAGTGTACAGTGAA
MT412308.1 Severe acute respiratory syndrome coronavirus 2 isolate SARS-CoV-2/human/USA/WA-UW-4413/2020,	TTCACCGAGGCCACGCGGAGTACGATCGAGTGTACAGTGAA
MT412309.1 Severe acute respiratory syndrome coronavirus 2 isolate SARS-CoV-2/human/USA/ID-UW-4418/2020, c	TTCACCGAGGCCACGCGGAGTACGATCGAGTGTACAGTGAA
MT412310.1 Severe acute respiratory syndrome coronavirus 2 isolate SARS-CoV-2/human/USA/WA-UW-6183/2020,	TTCACCGAGGCCACGCGGAGTACGATCGAGTGTACAGTGAA
MT412311.1 Severe acute respiratory syndrome coronavirus 2 isolate SARS-CoV-2/human/USA/WA-UW-6185/2020,	TTCACCGAGGCCACGCGGAGTACGATCGAGTGTACAGTGAA
MT412312.1 Severe acute respiratory syndrome coronavirus 2 isolate SARS-CoV-2/human/USA/WA-UW-6186/2020,	TTCACCGAGGCCACGCGGAGTACGATCGAGTGTACAGTGAA
MT412313.1 Severe acute respiratory syndrome coronavirus 2 isolate SARS-CoV-2/human/USA/WA-UW-6211/2020,	TTCACCGAGGCCACGCGGAGTACGATCGAGTGTACAGTGAA
MT412314.1 Severe acute respiratory syndrome coronavirus 2 isolate SARS-CoV-2/human/USA/WA-UW-6240/2020,	TTCACCGAGGCCACGCGGAGTACGATCGAGTGTACAGTGAA
MT412317.1 Severe acute respiratory syndrome coronavirus 2 isolate SARS-CoV-2/human/USA/CT-UW-6259/2020, c	TTCACCGAGGCCACGCGGAGTACGATCGAGTGTACAGTGAA
MT412319.1 Severe acute respiratory syndrome coronavirus 2 isolate SARS-CoV-2/human/USA/CT-UW-6271/2020, c	TTCACCGAGGCCACGCGGAGTACGATCGAGTGTACAGTGAA
MT412320.1 Severe acute respiratory syndrome coronavirus 2 isolate SARS-CoV-2/human/USA/CT-UW-6273/2020 O	TTCACCGAGGCCACGCGGAGTACGATCGAGTGTACAGTGAA
MT412321.1 Severe acute respiratory syndrome coronavirus 2 isolate SARS-CoV-2/human/USA/WA-UW-6302/2020,	TTCACCGAGGCCACGCGGAGTACGATCGAGTGTACAGTGAA
MT412322.1 Severe acute respiratory syndrome coronavirus 2 isolate SARS-CoV-2/human/USA/CT-UW-6314/2020, c	TTCACCGAGGCCACGCGGAGTACGATCGAGTGTACAGTGAA
MT412323.1 Severe acute respiratory syndrome coronavirus 2 isolate SARS-CoV-2/human/USA/WA-UW-6315/2020,	TTCACCGAGGCCACGCGGAGTACGATCGAGTGTACAGTGAA
MT412325.1 Severe acute respiratory syndrome coronavirus 2 isolate SARS-CoV-2/human/USA/WA-UW-6331/2020,	TTCACCGAGGCCACGCGGAGTACGATCGAGTGTACAGTGAA
MT412326.1 Severe acute respiratory syndrome coronavirus 2 isolate SARS-CoV-2/human/USA/WA-UW-6335/2020,	TTCACCGAGGCCACGCGGAGTACGATCGAGTGTACAGTGAA
MT412327.1 Severe acute respiratory syndrome coronavirus 2 isolate SARS-CoV-2/human/USA/ID-UW-6349/2020, c	TTCACCGAGGCCACGCGGAGTACGATCGAGTGTACAGTGAA

MT412328.1 Severe acute respiratory syndrome coronavirus 2 isolate SARS-CoV-2/human/USA/CT-UW-6362/2020, c	TTCACCGAGGCCACGCGGAGTACGATCGAGTGTACAGTGAA
MT412329.1 Severe acute respiratory syndrome coronavirus 2 isolate SARS-CoV-2/human/USA/CT-UW-6380/2020, c	TTCACCGAGGCCACGCGGAGTACGATCGAGTGTACAGTGAA
MT412330.1 Severe acute respiratory syndrome coronavirus 2 isolate SARS-CoV-2/human/USA/CT-UW-6385/2020, c	TTCACCGAGGCCACGCGGAGTACGATCGAGTGTACAGTGAA
MT412331.1 Severe acute respiratory syndrome coronavirus 2 isolate SARS-CoV-2/human/USA/WA-UW-6399/2020,	TTCACCGAGGCCACGCGGAGTACGATCGAGTGTACAGTGAA
MT412332.1 Severe acute respiratory syndrome coronavirus 2 isolate SARS-CoV-2/human/USA/WA-UW-6400/2020,	TTCACCGAGGCCACGCGGAGTACGATCGAGTGTACAGTGAA
MT412333.1 Severe acute respiratory syndrome coronavirus 2 isolate SARS-CoV-2/human/USA/WA-UW-6403/2020,	TTCACCGAGGCCACGCGGAGTACGATCGAGTGTACAGTGAA
MT412334.1 Severe acute respiratory syndrome coronavirus 2 isolate SARS-CoV-2/human/USA/WA-UW-6404/2020,	TTCACCGAGGCCACGCGGAGTACGATCGAGTGTACAGTGAA
MT412336.1 Severe acute respiratory syndrome coronavirus 2 isolate SARS-CoV-2/human/USA/WA-UW-6410/2020,	TTCACCGAGGCCACGCGGAGTACGATCGAGTGTACAGTGAA
MT412337.1 Severe acute respiratory syndrome coronavirus 2 isolate SARS-CoV-2/human/USA/WA-UW-6417/2020,	TTCACCGAGGCCACGCGGAGTACGATCGAGTGTACAGTGAA
MT415320.1 Severe acute respiratory syndrome coronavirus 2 isolate SARS-CoV-2/human/IND/OUMRK100/2020, cor	TTCACCGAGGCCACGCGGAGTACGATCGAGTGTACAGTGAA
MT415321.1 Severe acute respiratory syndrome coronavirus 2 isolate SARS-CoV-2/human/IND/GMCKN318/2020, cor	TTCACCGAGGCCACGCGGAGTACGATCGAGTGTACAGTGAA
MT415896.1 Severe acute respiratory syndrome coronavirus 2 isolate SARS-CoV-2/human/USA/VA-DCLS-0071/2020,	TTCACCGAGGCCACGCGGAGTACGATCGAGTGTACAGTGAA
MT415897.1 Severe acute respiratory syndrome coronavirus 2 isolate SARS-CoV-2/human/USA/VA-DCLS-0072/2020,	TTCACCGAGGCCACGCGGAGTACGATCGAGTGTACAGTGAA
MT415898.1 Severe acute respiratory syndrome coronavirus 2 isolate SARS-CoV-2/human/USA/VA-DCLS-0073/2020,	TTCACCGAGGCCACGCGGAGTACGATCGAGTGTACAGTGAA
MT415908.1 Severe acute respiratory syndrome coronavirus 2 isolate SARS-CoV-2/human/USA/VA-DCLS-0088/2020,	TTCACCGAGGCCACGCGGAGTACGATCGAGTGTACAGTGAA
MT416725.2 Severe acute respiratory syndrome coronavirus 2 isolate SARS-CoV-2/human/IND/8003/2020, complete	TTCACCGAGGCCACGCGGAGTACGATCGAGTGTACAGTGAA
MT416726.2 Severe acute respiratory syndrome coronavirus 2 isolate SARS-CoV-2/human/IND/8004/2020, complete	TTCACCGAGGCCACGCGGAGTACGATCGAGTGTACAGTGAA
MT418886.1 Severe acute respiratory syndrome coronavirus 2 isolate SARS-CoV-2/human/USA/VA-DCLS-0105/2020,	TTCACCGAGGCCACGCGGAGTACGATCGAGTGTACAGTGAA
MT418887.1 Severe acute respiratory syndrome coronavirus 2 isolate SARS-CoV-2/human/USA/VA-DCLS-0106/2020,	TTCACCGAGGCCACGCGGAGTACGATCGAGTGTACAGTGAA
MT418888.1 Severe acute respiratory syndrome coronavirus 2 isolate SARS-CoV-2/human/USA/VA-DCLS-0108/2020,	TTCACCGAGGCCACGCGGAGTACGATCGAGTGTACAGTGAA
MT418890.1 Severe acute respiratory syndrome coronavirus 2 isolate SARS-CoV-2/human/USA/VA-DCLS-0110/2020,	TTCACCGAGGCCACGCGGAGTACGATCGAGTGTACAGTGAA
MT418892.1 Severe acute respiratory syndrome coronavirus 2 isolate SARS-CoV-2/human/USA/VA-DCLS-0112/2020,	TTCACCGAGGCCACGCGGAGTACGATCGAGTGTACAGTGAA
MT419810.1 Severe acute respiratory syndrome coronavirus 2 isolate SARS-CoV2/human/USA/PR-CDC-S1/2020, com	TTCACCGAGGCCACGCGGAGTACGATCGAGTGTACAGTGAA
MT419811.1 Severe acute respiratory syndrome coronavirus 2 isolate SARS-CoV2/human/USA/PR-CDC-S2/2020 ORF1	TTCACCGAGGCCACGCGGAGTACGATCGAGTGTACAGTGAA
MT419812.1 Severe acute respiratory syndrome coronavirus 2 isolate SARS-CoV2/human/USA/PR-CDC-S3/2020, com	TTCACCGAGGCCACGCGGAGTACGATCGAGTGTACAGTGAA
MT419814.1 Severe acute respiratory syndrome coronavirus 2 isolate SARS-CoV2/human/USA/PR-CDC-S5/2020, com	TTCACCGAGGCCACGCGGAGTACGATCGAGTGTACAGTGAA
MT419815.1 Severe acute respiratory syndrome coronavirus 2 isolate SARS-CoV2/human/USA/PR-CDC-S6/2020, com	TTCACCGAGGCCACGCGGAGTACGATCGAGTGTACAGTGAA
MT419816.1 Severe acute respiratory syndrome coronavirus 2 isolate SARS-CoV2/human/USA/PR-CDC-S7/2020, com	TTCACCGAGGCCACGCGGAGTACGATCGAGTGTACAGTGAA
MT419818.1 Severe acute respiratory syndrome coronavirus 2 isolate SARS-CoV2/human/USA/PR-CDC-S9/2020, com	TTCACCGAGGCCACGCGGAGTACGATCGAGTGTACAGTGAA
MT419819.1 Severe acute respiratory syndrome coronavirus 2 isolate SARS-CoV2/human/USA/PR-CDC-S10/2020 ORF	TTCACCGAGGCCACGCGGAGTACGATCGAGTGTACAGTGAA
MT419820.1 Severe acute respiratory syndrome coronavirus 2 isolate SARS-CoV2/human/USA/PR-CDC-S11/2020, cor	TTCACCGAGGCCACGCGGAGTACGATCGAGTGTACAGTGAA
MT419821.1 Severe acute respiratory syndrome coronavirus 2 isolate SARS-CoV2/human/USA/PR-CDC-S18/2020 ORF	TTCACCGAGGCCACGCGGAGTACGATCGAGTGTACAGTGAA
MT419822.1 Severe acute respiratory syndrome coronavirus 2 isolate SARS-CoV2/human/USA/PR-CDC-S19/2020 ORF	TTCACCGAGGCCACGCGGAGTACGATCGAGTGTACAGTGAA

MT419828.1 Severe acute respiratory syndrome coronavirus 2 isolate SARS-CoV-2/human/USA/CA-SCCPHD-UC15/20	TTCACCGAGGCCACGCGGAGTACGATCGAGTGTACAGTGAA
MT419829.1 Severe acute respiratory syndrome coronavirus 2 isolate SARS-CoV-2/human/USA/CA-CDPH-UC1/2020,	TTCACCGAGGCCACGCGGAGTACGATCGAGTGTACAGTGAA
MT419830.1 Severe acute respiratory syndrome coronavirus 2 isolate SARS-CoV-2/human/USA/CA-CDPH-UC3/2020,	TTCACCGAGGCCACGCGGAGTACGATCGAGTGTACAGTGAA
MT419831.1 Severe acute respiratory syndrome coronavirus 2 isolate SARS-CoV-2/human/USA/CA-CDPH-UC2/2020,	TTCACCGAGGCCACGCGGAGTACGATCGAGTGTACAGTGAA
MT419834.1 Severe acute respiratory syndrome coronavirus 2 isolate SARS-CoV-2/human/USA/CA-CDPH-UC6/2020,	TTCACCGAGGCCACGCGGAGTACGATCGAGTGTACAGTGAA
MT419839.1 Severe acute respiratory syndrome coronavirus 2 isolate SARS-CoV-2/human/USA/CA-CDPH-UC11/2020	TTCACCGAGGCCACGCGGAGTACGATCGAGTGTACAGTGAA
MT419842.1 Severe acute respiratory syndrome coronavirus 2 isolate SARS-CoV-2/human/USA/CA-SCCPHD-UC16/20	TTCACCGAGGCCACGCGGAGTACGATCGAGTGTACAGTGAA
MT419851.1 Severe acute respiratory syndrome coronavirus 2 isolate SARS-CoV-2/human/USA/CA-CDPH-UC26/2020	TTCACCGAGGCCACGCGGAGTACGATCGAGTGTACAGTGAA
MT419854.1 Severe acute respiratory syndrome coronavirus 2 isolate SARS-CoV-2/human/USA/CA-SCCPHD-UC13/20	TTCACCGAGGCCACGCGGAGTACGATCGAGTGTACAGTGAA
MT419855.1 Severe acute respiratory syndrome coronavirus 2 isolate SARS-CoV-2/human/USA/CA-CDPH-UC30/2020	TTCACCGAGGCCACGCGGAGTACGATCGAGTGTACAGTGAA
MT419856.1 Severe acute respiratory syndrome coronavirus 2 isolate SARS-CoV-2/human/USA/CA-UCSF-UC32/2020	TTCACCGAGGCCACGCGGAGTACGATCGAGTGTACAGTGAA
MT419857.1 Severe acute respiratory syndrome coronavirus 2 isolate SARS-CoV-2/human/USA/CA-UCSF-UC33/2020	TTCACCGAGGCCACGCGGAGTACGATCGAGTGTACAGTGAA
MT419858.1 Severe acute respiratory syndrome coronavirus 2 isolate SARS-CoV-2/human/USA/CA-SCCPHD-UC34/20	TTCACCGAGGCCACGCGGAGTACGATCGAGTGTACAGTGAA
MT419859.1 Severe acute respiratory syndrome coronavirus 2 isolate SARS-CoV-2/human/USA/CA-SCCPHD-UC35/20	TTCACCGAGGCCACGCGGAGTACGATCGAGTGTACAGTGAA
MT419860.1 Severe acute respiratory syndrome coronavirus 2 isolate SARS-CoV-2/human/USA/CA-UCSF-UC36/2020,	TTCACCGAGGCCACGCGGAGTACGATCGAGTGTACAGTGAA
MT422806.1 Severe acute respiratory syndrome coronavirus 2 isolate SARS-CoV-2/human/USA/UF-4/2020, complete	TTCACCGAGGCCACGCGGAGTACGATCGAGTGTACAGTGAA
MT422807.1 Severe acute respiratory syndrome coronavirus 2 isolate SARS-CoV-2/human/USA/UF-5/2020, complete	TTCACCGAGGCCACGCGGAGTACGATCGAGTGTACAGTGAA
MT428551.1 Severe acute respiratory syndrome coronavirus 2 isolate SARS-CoV-2/human/KAZ/NCB-1/2020, complet	TTCACCGAGGCCACGCGGAGTACGATCGAGTGTACAGTGAA
MT428552.1 Severe acute respiratory syndrome coronavirus 2 isolate SARS-CoV-2/human/KAZ/NCB-2/2020, complet	TTCACCGAGGCCACGCGGAGTACGATCGAGTGTACAGTGAA
MT428553.1 Severe acute respiratory syndrome coronavirus 2 isolate SARS-CoV-2/human/KAZ/NCB-3/2020, complet	TTCACCGAGGCCACGCGGAGTACGATCGAGTGTACAGTGAA
MT428554.1 Severe acute respiratory syndrome coronavirus 2 isolate SARS-CoV-2/human/KAZ/NCB-5/2020, complet	TTCACCGAGGCCACGCGGAGTACGATCGAGTGTACAGTGAA
MT429183.1 Severe acute respiratory syndrome coronavirus 2 isolate SARS-CoV-2/human/USA/WI-UW-261/2020, co	TTCACCGAGGCCACGCGGAGTACGATCGAGTGTACAGTGAA
MT429184.1 Severe acute respiratory syndrome coronavirus 2 isolate SARS-CoV-2/human/USA/WI-UW-262/2020, co	TTCACCGAGGCCACGCGGAGTACGATCGAGTGTACAGTGAA
MT429185.1 Severe acute respiratory syndrome coronavirus 2 isolate SARS-CoV-2/human/USA/WI-UW-263/2020, co	TTCACCGAGGCCACGCGGAGTACGATCGAGTGTACAGTGAA
MT429186.1 Severe acute respiratory syndrome coronavirus 2 isolate SARS-CoV-2/human/USA/WI-UW-264/2020, co	TTCACCGAGGCCACGCGGAGTACGATCGAGTGTACAGTGAA
MT429187.1 Severe acute respiratory syndrome coronavirus 2 isolate SARS-CoV-2/human/USA/WI-UW-265/2020, co	TTCACCGAGGCCACGCGGAGTACGATCGAGTGTACAGTGAA
MT429188.1 Severe acute respiratory syndrome coronavirus 2 isolate SARS-CoV-2/human/USA/WI-UW-266/2020, co	TTCACCGAGGCCACGCGGAGTACGATCGAGTGTACAGTGAA
MT429189.1 Severe acute respiratory syndrome coronavirus 2 isolate SARS-CoV-2/human/USA/WI-UW-267/2020, co	TTCACCGAGGCCACGCGGAGTACGATCGAGTGTACAGTGAA
MT429190.1 Severe acute respiratory syndrome coronavirus 2 isolate SARS-CoV-2/human/USA/WI-UW-268/2020, co	TTCACCGAGGCCACGCGGAGTACGATCGAGTGTACAGTGAA
MT429191.1 Severe acute respiratory syndrome coronavirus 2 isolate SARS-CoV-2/human/USA/WI-UW-269/2020, co	TTCACCGAGGCCACGCGGAGTACGATCGAGTGTACAGTGAA
MT432195.1 Severe acute respiratory syndrome coronavirus 2 isolate SARS-CoV-2/human/USA/LA-EVTL01/2020, com	TTCACCGAGGCCACGCGGAGTACGATCGAGTGTACAGTGAA
MT434757.1 Severe acute respiratory syndrome coronavirus 2 isolate SARS-CoV-2/human/IND/nimh-2873/2020 ORF	TTCACCGAGGCCACGCGGAGTACGATCGAGTGTACAGTGAA
MT434758.1 Severe acute respiratory syndrome coronavirus 2 isolate SARS-CoV-2/human/IND/nimh-3952/2020 ORF	TTCACCGAGGCCACGCGGAGTACGATCGAGTGTACAGTGAA

MT434759.1 Severe acute respiratory syndrome coronavirus 2 isolate SARS-CoV-2/human/IND/nimh-1596/2020 ORF	TTCACCGAGGCCACGCGGAGTACGATCGAGTGTACAGTGAA
MT434760.1 Severe acute respiratory syndrome coronavirus 2 isolate SARS-CoV-2/human/IND/nimh-1598/2020 ORF	TTCACCGAGGCCACGCGGAGTACGATCGAGTGTACAGTGAA
MT434783.1 Severe acute respiratory syndrome coronavirus 2 isolate SARS-CoV-2/human/USA/NY-CDC-SURV0165NY	TTCACCGAGGCCACGCGGAGTACGATCGAGTGTACAGTGAA
MT434784.1 Severe acute respiratory syndrome coronavirus 2 isolate SARS-CoV-2/human/USA/NY-CDC-SURV0166NY	TTCACCGAGGCCACGCGGAGTACGATCGAGTGTACAGTGAA
MT434785.1 Severe acute respiratory syndrome coronavirus 2 isolate SARS-CoV-2/human/USA/NY-CDC-SURV0397NY	TTCACCGAGGCCACGCGGAGTACGATCGAGTGTACAGTGAA
MT434786.1 Severe acute respiratory syndrome coronavirus 2 isolate SARS-CoV-2/human/USA/NY-CDC-SURV0052NY	TTCACCGAGGCCACGCGGAGTACGATCGAGTGTACAGTGAA
MT434787.1 Severe acute respiratory syndrome coronavirus 2 isolate SARS-CoV-2/human/USA/NY-CDC-SURV0097NY	TTCACCGAGGCCACGCGGAGTACGATCGAGTGTACAGTGAA
MT434788.1 Severe acute respiratory syndrome coronavirus 2 isolate SARS-CoV-2/human/USA/NY-CDC-SURV0162NY	TTCACCGAGGCCACGCGGAGTACGATCGAGTGTACAGTGAA
MT434789.1 Severe acute respiratory syndrome coronavirus 2 isolate SARS-CoV-2/human/USA/NY-CDC-SURV0171NY	TTCACCGAGGCCACGCGGAGTACGATCGAGTGTACAGTGAA
MT434790.1 Severe acute respiratory syndrome coronavirus 2 isolate SARS-CoV-2/human/USA/NY-CDC-SURV0183NY	TTCACCGAGGCCACGCGGAGTACGATCGAGTGTACAGTGAA
MT434791.1 Severe acute respiratory syndrome coronavirus 2 isolate SARS-CoV-2/human/USA/NY-CDC-SURV0186NY	TTCACCGAGGCCACGCGGAGTACGATCGAGTGTACAGTGAA
MT434792.1 Severe acute respiratory syndrome coronavirus 2 isolate SARS-CoV-2/human/USA/NY-CDC-SURV0240NY	TTCACCGAGGCCACGCGGAGTACGATCGAGTGTACAGTGAA
MT434793.1 Severe acute respiratory syndrome coronavirus 2 isolate SARS-CoV-2/human/USA/NY-CDC-SURV0302NY	TTCACCGAGGCCACGCGGAGTACGATCGAGTGTACAGTGAA
MT434794.1 Severe acute respiratory syndrome coronavirus 2 isolate SARS-CoV-2/human/USA/NY-CDC-SURV0319NY	TTCACCGAGGCCACGCGGAGTACGATCGAGTGTACAGTGAA
MT434795.1 Severe acute respiratory syndrome coronavirus 2 isolate SARS-CoV-2/human/USA/NY-CDC-SURV0331NY	TTCACCGAGGCCACGCGGAGTACGATCGAGTGTACAGTGAA
MT434796.1 Severe acute respiratory syndrome coronavirus 2 isolate SARS-CoV-2/human/USA/NY-CDC-SURV0334NY	TTCACCGAGGCCACGCGGAGTACGATCGAGTGTACAGTGAA
MT434797.1 Severe acute respiratory syndrome coronavirus 2 isolate SARS-CoV-2/human/USA/NY-CDC-SURV0407NY	TTCACCGAGGCCACGCGGAGTACGATCGAGTGTACAGTGAA
MT434798.1 Severe acute respiratory syndrome coronavirus 2 isolate SARS-CoV-2/human/USA/NY-CDC-SURV0440NY	TTCACCGAGGCCACGCGGAGTACGATCGAGTGTACAGTGAA
MT434799.1 Severe acute respiratory syndrome coronavirus 2 isolate SARS-CoV-2/human/USA/NY-CDC-SURV0444NY	TTCACCGAGGCCACGCGGAGTACGATCGAGTGTACAGTGAA
MT434800.1 Severe acute respiratory syndrome coronavirus 2 isolate SARS-CoV-2/human/USA/NY-CDC-SURV0472NY	TTCACCGAGGCCACGCGGAGTACGATCGAGTGTACAGTGAA
MT434801.1 Severe acute respiratory syndrome coronavirus 2 isolate SARS-CoV-2/human/USA/NY-CDC-SURV0475NY	TTCACCGAGGCCACGCGGAGTACGATCGAGTGTACAGTGAA
MT434802.1 Severe acute respiratory syndrome coronavirus 2 isolate SARS-CoV-2/human/USA/NY-CDC-SURV0495NY	TTCACCGAGGCCACGCGGAGTACGATCGAGTGTACAGTGAA
MT434803.1 Severe acute respiratory syndrome coronavirus 2 isolate SARS-CoV-2/human/USA/NY-CDC-SURV0513NY	TTCACCGAGGCCACGCGGAGTACGATCGAGTGTACAGTGAA
MT434804.1 Severe acute respiratory syndrome coronavirus 2 isolate SARS-CoV-2/human/USA/NY-CDC-SURV0710NY	TTCACCGAGGCCACGCGGAGTACGATCGAGTGTACAGTGAA
MT434805.1 Severe acute respiratory syndrome coronavirus 2 isolate SARS-CoV-2/human/USA/NY-CDC-SURV0862NY	TTCACCGAGGCCACGCGGAGTACGATCGAGTGTACAGTGAA
MT434806.1 Severe acute respiratory syndrome coronavirus 2 isolate SARS-CoV-2/human/USA/NY-CDC-SURV0863NY	TTCACCGAGGCCACGCGGAGTACGATCGAGTGTACAGTGAA
MT434807.1 Severe acute respiratory syndrome coronavirus 2 isolate SARS-CoV-2/human/USA/NY-CDC-SURV0874NY	TTCACCGAGGCCACGCGGAGTACGATCGAGTGTACAGTGAA
MT434808.1 Severe acute respiratory syndrome coronavirus 2 isolate SARS-CoV-2/human/USA/NY-CDC-SURV0982NY	TTCACCGAGGCCACGCGGAGTACGATCGAGTGTACAGTGAA
MT434809.1 Severe acute respiratory syndrome coronavirus 2 isolate SARS-CoV-2/human/USA/NY-CDC-SURV0983NY	TTCACCGAGGCCACGCGGAGTACGATCGAGTGTACAGTGAA
MT434810.1 Severe acute respiratory syndrome coronavirus 2 isolate SARS-CoV-2/human/USA/NY-CDC-SURV0988NY	TTCACCGAGGCCACGCGGAGTACGATCGAGTGTACAGTGAA
MT434811.1 Severe acute respiratory syndrome coronavirus 2 isolate SARS-CoV-2/human/USA/NY-CDC-SURV0996NY	TTCACCGAGGCCACGCGGAGTACGATCGAGTGTACAGTGAA
MT434812.1 Severe acute respiratory syndrome coronavirus 2 isolate SARS-CoV-2/human/USA/NY-CDC-SURV1000NY	TTCACCGAGGCCACGCGGAGTACGATCGAGTGTACAGTGAA
MT434813.1 Severe acute respiratory syndrome coronavirus 2 isolate SARS-CoV-2/human/USA/NY-CDC-SURV0144NY	TTCACCGAGGCCACGCGGAGTACGATCGAGTGTACAGTGAA

MT434814.1 Severe acute respiratory syndrome coronavirus 2 isolate SARS-CoV-2/human/USA/NY-CDC-SURV0168NY	TTCACCGAGGCCACGCGGAGTACGATCGAGTGTACAGTGAA
MT434816.1 Severe acute respiratory syndrome coronavirus 2 isolate SARS-CoV-2/human/USA/NY-CDC-SURV0592NY	TTCACCGAGGCCACGCGGAGTACGATCGAGTGTACAGTGAA
MT434817.1 Severe acute respiratory syndrome coronavirus 2 isolate SARS-CoV-2/human/USA/NY-CDC-SURV0985NY	TTCACCGAGGCCACGCGGAGTACGATCGAGTGTACAGTGAA
MT435079.1 Severe acute respiratory syndrome coronavirus 2 isolate SARS-CoV-2/human/IND/GBRC2/2020, complet	TTCACCGAGGCCACGCGGAGTACGATCGAGTGTACAGTGAA
MT435080.1 Severe acute respiratory syndrome coronavirus 2 isolate SARS-CoV-2/human/IND/GBRC3/2020, complet	TTCACCGAGGCCACGCGGAGTACGATCGAGTGTACAGTGAA
MT435081.1 Severe acute respiratory syndrome coronavirus 2 isolate SARS-CoV-2/human/IND/GBRC4/2020, complet	TTCACCGAGGCCACGCGGAGTACGATCGAGTGTACAGTGAA
MT435082.1 Severe acute respiratory syndrome coronavirus 2 isolate SARS-CoV-2/human/IND/GBRC5/2020, complet	TTCACCGAGGCCACGCGGAGTACGATCGAGTGTACAGTGAA
MT435083.1 Severe acute respiratory syndrome coronavirus 2 isolate SARS-CoV-2/human/IND/GBRC6/2020, complet	TTCACCGAGGCCACGCGGAGTACGATCGAGTGTACAGTGAA
MT435084.1 Severe acute respiratory syndrome coronavirus 2 isolate SARS-CoV-2/human/IND/GBRC7/2020, complet	TTCACCGAGGCCACGCGGAGTACGATCGAGTGTACAGTGAA
MT435085.1 Severe acute respiratory syndrome coronavirus 2 isolate SARS-CoV-2/human/IND/GBRC8/2020, complet	TTCACCGAGGCCACGCGGAGTACGATCGAGTGTACAGTGAA
MT438718.1 Severe acute respiratory syndrome coronavirus 2 isolate SARS-CoV-2/human/USA/CA-CZB-1000/2020, c	TTCACCGAGGCCACGCGGAGTACGATCGAGTGTACAGTGAA
MT438719.1 Severe acute respiratory syndrome coronavirus 2 isolate SARS-CoV-2/human/USA/CA-CZB-1001/2020, c	TTCACCGAGGCCACGCGGAGTACGATCGAGTGTACAGTGAA
MT438720.1 Severe acute respiratory syndrome coronavirus 2 isolate SARS-CoV-2/human/USA/CA-CZB-1002/2020, c	TTCACCGAGGCCACGCGGAGTACGATCGAGTGTACAGTGAA
MT438721.1 Severe acute respiratory syndrome coronavirus 2 isolate SARS-CoV-2/human/USA/CA-CZB-1003/2020, c	TTCACCGAGGCCACGCGGAGTACGATCGAGTGTACAGTGAA
MT438722.1 Severe acute respiratory syndrome coronavirus 2 isolate SARS-CoV-2/human/USA/CA-CZB-1004/2020, c	TTCACCGAGGCCACGCGGAGTACGATCGAGTGTACAGTGAA
MT438723.1 Severe acute respiratory syndrome coronavirus 2 isolate SARS-CoV-2/human/USA/CA-CZB-1005/2020, c	TTCACCGAGGCCACGCGGAGTACGATCGAGTGTACAGTGAA
MT438724.1 Severe acute respiratory syndrome coronavirus 2 isolate SARS-CoV-2/human/USA/CA-CZB-1006/2020, c	TTCACCGAGGCCACGCGGAGTACGATCGAGTGTACAGTGAA
MT438725.1 Severe acute respiratory syndrome coronavirus 2 isolate SARS-CoV-2/human/USA/CA-CZB-1007/2020, c	TTCACCGAGGCCACGCGGAGTACGATCGAGTGTACAGTGAA
MT438726.1 Severe acute respiratory syndrome coronavirus 2 isolate SARS-CoV-2/human/USA/CA-CZB-1008/2020 O	TTCACCGAGGCCACGCGGAGTACGATCGAGTGTACAGTGAA
MT438727.1 Severe acute respiratory syndrome coronavirus 2 isolate SARS-CoV-2/human/USA/CA-CZB-1009/2020, c	TTCACCGAGGCCACGCGGAGTACGATCGAGTGTACAGTGAA
MT438728.1 Severe acute respiratory syndrome coronavirus 2 isolate SARS-CoV-2/human/USA/CA-CZB-1010/2020, c	TTCACCGAGGCCACGCGGAGTACGATCGAGTGTACAGTGAA
MT438729.1 Severe acute respiratory syndrome coronavirus 2 isolate SARS-CoV-2/human/USA/CA-CZB-1011/2020, c	TTCACCGAGGCCACGCGGAGTACGATCGAGTGTACAGTGAA
MT438730.1 Severe acute respiratory syndrome coronavirus 2 isolate SARS-CoV-2/human/USA/CA-CZB-1012/2020, c	TTCACCGAGGCCACGCGGAGTACGATCGAGTGTACAGTGAA
MT438731.1 Severe acute respiratory syndrome coronavirus 2 isolate SARS-CoV-2/human/USA/CA-CZB-1013/2020, c	TTCACCGAGGCCACGCGGAGTACGATCGAGTGTACAGTGAA
MT438732.1 Severe acute respiratory syndrome coronavirus 2 isolate SARS-CoV-2/human/USA/CA-CZB-1014/2020, c	TTCACCGAGGCCACGCGGAGTACGATCGAGTGTACAGTGAA
MT438733.1 Severe acute respiratory syndrome coronavirus 2 isolate SARS-CoV-2/human/USA/CA-CZB-1015/2020 O	TTCACCGAGGCCACGCGGAGTACGATCGAGTGTACAGTGAA
MT438734.1 Severe acute respiratory syndrome coronavirus 2 isolate SARS-CoV-2/human/USA/CA-CZB-1016/2020, c	TTCACCGAGGCCACGCGGAGTACGATCGAGTGTACAGTGAA
MT438736.1 Severe acute respiratory syndrome coronavirus 2 isolate SARS-CoV-2/human/USA/CA-CZB-1018/2020, c	TTCACCGAGGCCACGCGGAGTACGATCGAGTGTACAGTGAA
MT438737.1 Severe acute respiratory syndrome coronavirus 2 isolate SARS-CoV-2/human/USA/CA-CZB-1019/2020, c	TTCACCGAGGCCACGCGGAGTACGATCGAGTGTACAGTGAA
MT438738.1 Severe acute respiratory syndrome coronavirus 2 isolate SARS-CoV-2/human/USA/CA-CZB-1020/2020, c	TTCACCGAGGCCACGCGGAGTACGATCGAGTGTACAGTGAA
MT438739.1 Severe acute respiratory syndrome coronavirus 2 isolate SARS-CoV-2/human/USA/CA-CZB-1021/2020, c	TTCACCGAGGCCACGCGGAGTACGATCGAGTGTACAGTGAA
MT438740.1 Severe acute respiratory syndrome coronavirus 2 isolate SARS-CoV-2/human/USA/CA-CZB-1022/2020, c	TTCACCGAGGCCACGCGGAGTACGATCGAGTGTACAGTGAA
MT438741.1 Severe acute respiratory syndrome coronavirus 2 isolate SARS-CoV-2/human/USA/CA-CZB-1023/2020, c	TTCACCGAGGCCACGCGGAGTACGATCGAGTGTACAGTGAA

MT438742.1 Severe acute respiratory syndrome coronavirus 2 isolate SARS-CoV-2/human/USA/CA-CZB-1024/2020, c	TTCACCGAGGCCACGCGGAGTACGATCGAGTGTACAGTGAA
MT438743.1 Severe acute respiratory syndrome coronavirus 2 isolate SARS-CoV-2/human/USA/CA-CZB-1025/2020, c	TTCACCGAGGCCACGCGGAGTACGATCGAGTGTACAGTGAA
MT438744.1 Severe acute respiratory syndrome coronavirus 2 isolate SARS-CoV-2/human/USA/CA-CZB-1026/2020, c	TTCACCGAGGCCACGCGGAGTACGATCGAGTGTACAGTGAA
MT438746.1 Severe acute respiratory syndrome coronavirus 2 isolate SARS-CoV-2/human/USA/CA-CZB-1028/2020, c	TTCACCGAGGCCACGCGGAGTACGATCGAGTGTACAGTGAA
MT438747.1 Severe acute respiratory syndrome coronavirus 2 isolate SARS-CoV-2/human/USA/CA-CZB-1029/2020, c	TTCACCGAGGCCACGCGGAGTACGATCGAGTGTACAGTGAA
MT438748.1 Severe acute respiratory syndrome coronavirus 2 isolate SARS-CoV-2/human/USA/CA-CZB-1030/2020, c	TTCACCGAGGCCACGCGGAGTACGATCGAGTGTACAGTGAA
MT438749.1 Severe acute respiratory syndrome coronavirus 2 isolate SARS-CoV-2/human/USA/CA-CZB-1031/2020, c	TTCACCGAGGCCACGCGGAGTACGATCGAGTGTACAGTGAA
MT438750.1 Severe acute respiratory syndrome coronavirus 2 isolate SARS-CoV-2/human/USA/CA-CZB-1032/2020, c	TTCACCGAGGCCACGCGGAGTACGATCGAGTGTACAGTGAA
MT438751.1 Severe acute respiratory syndrome coronavirus 2 isolate SARS-CoV-2/human/USA/CA-CZB-1033/2020, c	TTCACCGAGGCCACGCGGAGTACGATCGAGTGTACAGTGAA
MT438752.1 Severe acute respiratory syndrome coronavirus 2 isolate SARS-CoV-2/human/USA/CA-CZB-1034/2020, c	TTCACCGAGGCCACGCGGAGTACGATCGAGTGTACAGTGAA
MT438753.1 Severe acute respiratory syndrome coronavirus 2 isolate SARS-CoV-2/human/USA/CA-CZB-1035/2020, c	TTCACCGAGGCCACGCGGAGTACGATCGAGTGTACAGTGAA
MT438754.1 Severe acute respiratory syndrome coronavirus 2 isolate SARS-CoV-2/human/USA/CA-CZB-1036/2020, c	TTCACCGAGGCCACGCGGAGTACGATCGAGTGTACAGTGAA
MT438755.1 Severe acute respiratory syndrome coronavirus 2 isolate SARS-CoV-2/human/USA/CA-CZB-1037/2020, c	TTCACCGAGGCCACGCGGAGTACGATCGAGTGTACAGTGAA
MT438756.1 Severe acute respiratory syndrome coronavirus 2 isolate SARS-CoV-2/human/USA/CA-CZB-1038/2020, c	TTCACCGAGGCCACGCGGAGTACGATCGAGTGTACAGTGAA
MT438757.1 Severe acute respiratory syndrome coronavirus 2 isolate SARS-CoV-2/human/USA/CA-CZB-1039/2020, c	TTCACCGAGGCCACGCGGAGTACGATCGAGTGTACAGTGAA
MT438758.1 Severe acute respiratory syndrome coronavirus 2 isolate SARS-CoV-2/human/USA/CA-CZB-1040/2020, c	TTCACCGAGGCCACGCGGAGTACGATCGAGTGTACAGTGAA
MT438759.1 Severe acute respiratory syndrome coronavirus 2 isolate SARS-CoV-2/human/USA/CA-CZB-1041/2020, c	TTCACCGAGGCCACGCGGAGTACGATCGAGTGTACAGTGAA
MT438760.1 Severe acute respiratory syndrome coronavirus 2 isolate SARS-CoV-2/human/USA/CA-CZB-1042/2020, c	TTCACCGAGGCCACGCGGAGTACGATCGAGTGTACAGTGAA
MT439230.1 Severe acute respiratory syndrome coronavirus 2 isolate SARS-CoV-2/human/USA/MI-MDHHS-SC20113/	TTCACCGAGGCCACGCGGAGTACGATCGAGTGTACAGTGAA
MT439231.1 Severe acute respiratory syndrome coronavirus 2 isolate SARS-CoV-2/human/USA/MI-MDHHS-SC20122/	TTCACCGAGGCCACGCGGAGTACGATCGAGTGTACAGTGAA
MT439232.1 Severe acute respiratory syndrome coronavirus 2 isolate SARS-CoV-2/human/USA/MI-MDHHS-SC20129/	TTCACCGAGGCCACGCGGAGTACGATCGAGTGTACAGTGAA
MT439233.1 Severe acute respiratory syndrome coronavirus 2 isolate SARS-CoV-2/human/USA/MI-MDHHS-SC20162/	TTCACCGAGGCCACGCGGAGTACGATCGAGTGTACAGTGAA
MT439234.1 Severe acute respiratory syndrome coronavirus 2 isolate SARS-CoV-2/human/USA/MI-MDHHS-SC20189/	TTCACCGAGGCCACGCGGAGTACGATCGAGTGTACAGTGAA
MT439235.1 Severe acute respiratory syndrome coronavirus 2 isolate SARS-CoV-2/human/USA/MI-MDHHS-SC20190/	TTCACCGAGGCCACGCGGAGTACGATCGAGTGTACAGTGAA
MT439236.1 Severe acute respiratory syndrome coronavirus 2 isolate SARS-CoV-2/human/USA/MI-MDHHS-SC20131/	TTCACCGAGGCCACGCGGAGTACGATCGAGTGTACAGTGAA
MT439237.1 Severe acute respiratory syndrome coronavirus 2 isolate SARS-CoV-2/human/USA/MI-MDHHS-SC20179/	TTCACCGAGGCCACGCGGAGTACGATCGAGTGTACAGTGAA
MT439238.1 Severe acute respiratory syndrome coronavirus 2 isolate SARS-CoV-2/human/USA/MI-MDHHS-SC20150/	TTCACCGAGGCCACGCGGAGTACGATCGAGTGTACAGTGAA
MT439239.1 Severe acute respiratory syndrome coronavirus 2 isolate SARS-CoV-2/human/USA/MI-MDHHS-SC20169/	TTCACCGAGGCCACGCGGAGTACGATCGAGTGTACAGTGAA
MT439242.1 Severe acute respiratory syndrome coronavirus 2 isolate SARS-CoV-2/human/USA/MI-MDHHS-SC20130/	TTCACCGAGGCCACGCGGAGTACGATCGAGTGTACAGTGAA
MT439243.1 Severe acute respiratory syndrome coronavirus 2 isolate SARS-CoV-2/human/USA/MI-MDHHS-SC20120/	TTCACCGAGGCCACGCGGAGTACGATCGAGTGTACAGTGAA
MT439244.1 Severe acute respiratory syndrome coronavirus 2 isolate SARS-CoV-2/human/USA/MI-MDHHS-SC20136/	TTCACCGAGGCCACGCGGAGTACGATCGAGTGTACAGTGAA
MT439245.1 Severe acute respiratory syndrome coronavirus 2 isolate SARS-CoV-2/human/USA/MI-MDHHS-SC20158/	TTCACCGAGGCCACGCGGAGTACGATCGAGTGTACAGTGAA
MT439247.1 Severe acute respiratory syndrome coronavirus 2 isolate SARS-CoV-2/human/USA/MI-MDHHS-SC20147/	TTCACCGAGGCCACGCGGAGTACGATCGAGTGTACAGTGAA

MT439248.1 Severe acute respiratory syndrome coronavirus 2 isolate SARS-CoV-2/human/USA/MI-MDHHS-SC20143/	TTCACCGAGGCCACGCGGAGTACGATCGAGTGTACAGTGAA
MT439249.1 Severe acute respiratory syndrome coronavirus 2 isolate SARS-CoV-2/human/USA/MI-MDHHS-SC20139/	TTCACCGAGGCCACGCGGAGTACGATCGAGTGTACAGTGAA
MT439250.1 Severe acute respiratory syndrome coronavirus 2 isolate SARS-CoV-2/human/USA/MI-MDHHS-SC20163/	TTCACCGAGGCCACGCGGAGTACGATCGAGTGTACAGTGAA
MT439251.1 Severe acute respiratory syndrome coronavirus 2 isolate SARS-CoV-2/human/USA/MI-MDHHS-SC20171/	TTCACCGAGGCCACGCGGAGTACGATCGAGTGTACAGTGAA
MT439252.1 Severe acute respiratory syndrome coronavirus 2 isolate SARS-CoV-2/human/USA/MI-MDHHS-SC20161/	TTCACCGAGGCCACGCGGAGTACGATCGAGTGTACAGTGAA
MT439253.1 Severe acute respiratory syndrome coronavirus 2 isolate SARS-CoV-2/human/USA/MI-MDHHS-SC20173/	TTCACCGAGGCCACGCGGAGTACGATCGAGTGTACAGTGAA
MT439254.1 Severe acute respiratory syndrome coronavirus 2 isolate SARS-CoV-2/human/USA/MI-MDHHS-SC20175/	TTCACCGAGGCCACGCGGAGTACGATCGAGTGTACAGTGAA
MT439256.1 Severe acute respiratory syndrome coronavirus 2 isolate SARS-CoV-2/human/USA/MI-MDHHS-SC20156/	TTCACCGAGGCCACGCGGAGTACGATCGAGTGTACAGTGAA
MT439258.1 Severe acute respiratory syndrome coronavirus 2 isolate SARS-CoV-2/human/USA/MI-MDHHS-SC20154/	TTCACCGAGGCCACGCGGAGTACGATCGAGTGTACAGTGAA
MT439260.1 Severe acute respiratory syndrome coronavirus 2 isolate SARS-CoV-2/human/USA/MI-MDHHS-SC20153/	TTCACCGAGGCCACGCGGAGTACGATCGAGTGTACAGTGAA
MT439261.1 Severe acute respiratory syndrome coronavirus 2 isolate SARS-CoV-2/human/USA/MI-MDHHS-SC20117/	TTCACCGAGGCCACGCGGAGTACGATCGAGTGTACAGTGAA
MT439262.1 Severe acute respiratory syndrome coronavirus 2 isolate SARS-CoV-2/human/USA/MI-MDHHS-SC20149/	TTCACCGAGGCCACGCGGAGTACGATCGAGTGTACAGTGAA
MT439263.1 Severe acute respiratory syndrome coronavirus 2 isolate SARS-CoV-2/human/USA/MI-MDHHS-SC20137/	TTCACCGAGGCCACGCGGAGTACGATCGAGTGTACAGTGAA
MT439264.1 Severe acute respiratory syndrome coronavirus 2 isolate SARS-CoV-2/human/USA/MI-MDHHS-SC20118/	TTCACCGAGGCCACGCGGAGTACGATCGAGTGTACAGTGAA
MT439265.1 Severe acute respiratory syndrome coronavirus 2 isolate SARS-CoV-2/human/USA/MI-MDHHS-SC20110/	TTCACCGAGGCCACGCGGAGTACGATCGAGTGTACAGTGAA
MT439267.1 Severe acute respiratory syndrome coronavirus 2 isolate SARS-CoV-2/human/USA/MI-MDHHS-SC20106/	TTCACCGAGGCCACGCGGAGTACGATCGAGTGTACAGTGAA
MT439268.1 Severe acute respiratory syndrome coronavirus 2 isolate SARS-CoV-2/human/USA/MI-MDHHS-SC20107/	TTCACCGAGGCCACGCGGAGTACGATCGAGTGTACAGTGAA
MT439269.1 Severe acute respiratory syndrome coronavirus 2 isolate SARS-CoV-2/human/USA/MI-MDHHS-SC20108/	TTCACCGAGGCCACGCGGAGTACGATCGAGTGTACAGTGAA
MT439270.1 Severe acute respiratory syndrome coronavirus 2 isolate SARS-CoV-2/human/USA/MI-MDHHS-SC20183/	TTCACCGAGGCCACGCGGAGTACGATCGAGTGTACAGTGAA
MT439271.1 Severe acute respiratory syndrome coronavirus 2 isolate SARS-CoV-2/human/USA/MI-MDHHS-SC20160/	TTCACCGAGGCCACGCGGAGTACGATCGAGTGTACAGTGAA
MT439272.1 Severe acute respiratory syndrome coronavirus 2 isolate SARS-CoV-2/human/USA/MI-MDHHS-SC20126/	TTCACCGAGGCCACGCGGAGTACGATCGAGTGTACAGTGAA
MT439273.1 Severe acute respiratory syndrome coronavirus 2 isolate SARS-CoV-2/human/USA/MI-MDHHS-SC20148/	TTCACCGAGGCCACGCGGAGTACGATCGAGTGTACAGTGAA
MT439274.1 Severe acute respiratory syndrome coronavirus 2 isolate SARS-CoV-2/human/USA/MI-MDHHS-SC20119/	TTCACCGAGGCCACGCGGAGTACGATCGAGTGTACAGTGAA
MT439275.1 Severe acute respiratory syndrome coronavirus 2 isolate SARS-CoV-2/human/USA/MI-MDHHS-SC20170/	TTCACCGAGGCCACGCGGAGTACGATCGAGTGTACAGTGAA
MT439276.1 Severe acute respiratory syndrome coronavirus 2 isolate SARS-CoV-2/human/USA/MI-MDHHS-SC20111/	TTCACCGAGGCCACGCGGAGTACGATCGAGTGTACAGTGAA
MT439277.1 Severe acute respiratory syndrome coronavirus 2 isolate SARS-CoV-2/human/USA/MI-MDHHS-SC20182/	TTCACCGAGGCCACGCGGAGTACGATCGAGTGTACAGTGAA
MT439278.1 Severe acute respiratory syndrome coronavirus 2 isolate SARS-CoV-2/human/USA/MI-MDHHS-SC20177/	TTCACCGAGGCCACGCGGAGTACGATCGAGTGTACAGTGAA
MT439279.1 Severe acute respiratory syndrome coronavirus 2 isolate SARS-CoV-2/human/USA/MI-MDHHS-SC20134/	TTCACCGAGGCCACGCGGAGTACGATCGAGTGTACAGTGAA
MT439280.1 Severe acute respiratory syndrome coronavirus 2 isolate SARS-CoV-2/human/USA/MI-MDHHS-SC20152/	TTCACCGAGGCCACGCGGAGTACGATCGAGTGTACAGTGAA
MT439281.1 Severe acute respiratory syndrome coronavirus 2 isolate SARS-CoV-2/human/USA/MI-MDHHS-SC20186/	TTCACCGAGGCCACGCGGAGTACGATCGAGTGTACAGTGAA
MT439282.1 Severe acute respiratory syndrome coronavirus 2 isolate SARS-CoV-2/human/USA/MI-MDHHS-SC20132/	TTCACCGAGGCCACGCGGAGTACGATCGAGTGTACAGTGAA
MT439283.1 Severe acute respiratory syndrome coronavirus 2 isolate SARS-CoV-2/human/USA/MI-MDHHS-SC20138/	TTCACCGAGGCCACGCGGAGTACGATCGAGTGTACAGTGAA
MT439284.1 Severe acute respiratory syndrome coronavirus 2 isolate SARS-CoV-2/human/USA/MI-MDHHS-SC20124/	TTCACCGAGGCCACACGGAGTACGATCGAGTGTACAGTGAA

MT439285.1 Severe acute respiratory syndrome coronavirus 2 isolate SARS-CoV-2/human/USA/MI-MDHHS-SC20133/	TTCACCGAGGCCACGCGGAGTACGATCGAGTGTACAGTGAA
MT439286.1 Severe acute respiratory syndrome coronavirus 2 isolate SARS-CoV-2/human/USA/MI-MDHHS-SC20121/	TTCACCGAGGCCACGCGGAGTACGATCGAGTGTACAGTGAA
MT439287.1 Severe acute respiratory syndrome coronavirus 2 isolate SARS-CoV-2/human/USA/MI-MDHHS-SC20180/	TTCACCGAGGCCACGCGGAGTACGATCGAGTGTACAGTGAA
MT439288.1 Severe acute respiratory syndrome coronavirus 2 isolate SARS-CoV-2/human/USA/MI-MDHHS-SC20115/	TTCACCGAGGCCACGCGGAGTACGATCGAGTGTACAGTGAA
MT439289.1 Severe acute respiratory syndrome coronavirus 2 isolate SARS-CoV-2/human/USA/MI-MDHHS-SC20104/	TTCACCGAGGCCACGCGGAGTACGATCGAGTGTACAGTGAA
MT439290.1 Severe acute respiratory syndrome coronavirus 2 isolate SARS-CoV-2/human/USA/MI-MDHHS-SC20109/	TTCACCGAGGCCACGCGGAGTACGATCGAGTGTACAGTGAA
MT439291.1 Severe acute respiratory syndrome coronavirus 2 isolate SARS-CoV-2/human/USA/MI-MDHHS-SC20101/	TTCACCGAGGCCACGCGGAGTACGATCGAGTGTACAGTGAA
MT439292.1 Severe acute respiratory syndrome coronavirus 2 isolate SARS-CoV-2/human/USA/MI-MDHHS-SC20174/	TTCACCGAGGCCACGCGGAGTACGATCGAGTGTACAGTGAA
MT439293.1 Severe acute respiratory syndrome coronavirus 2 isolate SARS-CoV-2/human/USA/MI-MDHHS-SC20116/	TTCACCGAGGCCACGCGGAGTACGATCGAGTGTACAGTGAA
MT439295.1 Severe acute respiratory syndrome coronavirus 2 isolate SARS-CoV-2/human/USA/MI-MDHHS-SC20164/	TTCACCGAGGCCACGCGGAGTACGATCGAGTGTACAGTGAA
MT439296.1 Severe acute respiratory syndrome coronavirus 2 isolate SARS-CoV-2/human/USA/MI-MDHHS-SC20142/	TTCACCGAGGCCACGCGGAGTACGATCGAGTGTACAGTGAA
MT439298.1 Severe acute respiratory syndrome coronavirus 2 isolate SARS-CoV-2/human/USA/MI-MDHHS-SC20102/	TTCACCGAGGCCACGCGGAGTACGATCGAGTGTACAGTGAA
MT439299.1 Severe acute respiratory syndrome coronavirus 2 isolate SARS-CoV-2/human/USA/MI-MDHHS-SC20167/	TTCACCGAGGCCACGCGGAGTACGATCGAGTGTACAGTGAA
MT439300.1 Severe acute respiratory syndrome coronavirus 2 isolate SARS-CoV-2/human/USA/MI-MDHHS-SC20135/	TTCACCGAGGCCACGCGGAGTACGATCGAGTGTACAGTGAA
MT439301.1 Severe acute respiratory syndrome coronavirus 2 isolate SARS-CoV-2/human/USA/MI-MDHHS-SC20128/	TTCACCGAGGCCACGCGGAGTACGATCGAGTGTACAGTGAA
MT439302.1 Severe acute respiratory syndrome coronavirus 2 isolate SARS-CoV-2/human/USA/MI-MDHHS-SC20181/	TTCACCGAGGCCACGCGGAGTACGATCGAGTGTACAGTGAA
MT439303.1 Severe acute respiratory syndrome coronavirus 2 isolate SARS-CoV-2/human/USA/MI-MDHHS-SC20157/	TTCACCGAGGCCACGCGGAGTACGATCGAGTGTACAGTGAA
MT439304.1 Severe acute respiratory syndrome coronavirus 2 isolate SARS-CoV-2/human/USA/MI-MDHHS-SC20141/	TTCACCGAGGCCACGCGGAGTACGATCGAGTGTACAGTGAA
MT439305.1 Severe acute respiratory syndrome coronavirus 2 isolate SARS-CoV-2/human/USA/MI-MDHHS-SC20114/	TTCACCGAGGCCACGCGGAGTACGATCGAGTGTACAGTGAA
MT439306.1 Severe acute respiratory syndrome coronavirus 2 isolate SARS-CoV-2/human/USA/MI-MDHHS-SC20185/	TTCACCGAGGCCACGCGGAGTACGATCGAGTGTACAGTGAA
MT439307.1 Severe acute respiratory syndrome coronavirus 2 isolate SARS-CoV-2/human/USA/MI-MDHHS-SC20144/	TTCACCGAGGCCACGCGGAGTACGATCGAGTGTACAGTGAA
MT439308.1 Severe acute respiratory syndrome coronavirus 2 isolate SARS-CoV-2/human/USA/MI-MDHHS-SC20125/	TTCACCGAGGCCACGCGGAGTACGATCGAGTGTACAGTGAA
MT439309.1 Severe acute respiratory syndrome coronavirus 2 isolate SARS-CoV-2/human/USA/MI-MDHHS-SC20184/	TTCACCGAGGCCACGCGGAGTACGATCGAGTGTACAGTGAA
MT439310.1 Severe acute respiratory syndrome coronavirus 2 isolate SARS-CoV-2/human/USA/MI-MDHHS-SC20188/	TTCACCGAGGCCACGCGGAGTACGATCGAGTGTACAGTGAA
MT439311.1 Severe acute respiratory syndrome coronavirus 2 isolate SARS-CoV-2/human/USA/MI-MDHHS-SC20165/	TTCACCGAGGCCACGCGGAGTACGATCGAGTGTACAGTGAA
MT439312.1 Severe acute respiratory syndrome coronavirus 2 isolate SARS-CoV-2/human/USA/MI-MDHHS-SC20146/	TTCACCGAGGCCACGCGGAGTACGATCGAGTGTACAGTGAA
MT439313.1 Severe acute respiratory syndrome coronavirus 2 isolate SARS-CoV-2/human/USA/MI-MDHHS-SC20123/	TTCACCGAGGCCACGCGGAGTACGATCGAGTGTACAGTGAA
MT439314.1 Severe acute respiratory syndrome coronavirus 2 isolate SARS-CoV-2/human/USA/MI-MDHHS-SC20178/	TTCACCGAGGCCACGCGGAGTACGATCGAGTGTACAGTGAA
MT439315.1 Severe acute respiratory syndrome coronavirus 2 isolate SARS-CoV-2/human/USA/MI-MDHHS-SC20155/	TTCACCGAGGCCACGCGGAGTACGATCGAGTGTACAGTGAA
MT439316.1 Severe acute respiratory syndrome coronavirus 2 isolate SARS-CoV-2/human/USA/MI-MDHHS-SC20151/	TTCACCGAGGCCACGCGGAGTACGATCGAGTGTACAGTGAA
MT439317.1 Severe acute respiratory syndrome coronavirus 2 isolate SARS-CoV-2/human/USA/MI-MDHHS-SC20166/	TTCACCGAGGCCACGCGGAGTACGATCGAGTGTACAGTGAA
MT439318.1 Severe acute respiratory syndrome coronavirus 2 isolate SARS-CoV-2/human/USA/MI-MDHHS-SC20168/	TTCACCGAGGCCACGCGGAGTACGATCGAGTGTACAGTGAA
MT439319.1 Severe acute respiratory syndrome coronavirus 2 isolate SARS-CoV-2/human/USA/MI-MDHHS-SC20105/	TTCACCGAGGCCACGCGGAGTACGATCGAGTGTACAGTGAA

MT439320.1 Severe acute respiratory syndrome coronavirus 2 isolate SARS-CoV-2/human/USA/MI-MDHHS-SC20103/	TTCACCGAGGCCACGCGGAGTACGATCGAGTGTACAGTGAA
MT439596.1 Severe acute respiratory syndrome coronavirus 2 isolate SARS-CoV-2/human/IND/nimh-4378/2020 ORF	TTCACCGAGGCCACGCGGAGTACGATCGAGTGTACAGTGAA
MT444148.1 Severe acute respiratory syndrome coronavirus 2 isolate SARS-CoV-2/human/USA/CA-NHRC-01/2020, co	TTCACCGAGGCCACGCGGAGTACGATCGAGTGTACAGTGAA
MT444515.1 Severe acute respiratory syndrome coronavirus 2 isolate SARS-CoV-2/human/USA/UT-00066/2020 ORF1	TTCACCGAGGCCACGCGGAGTACGATCGAGTGTACAGTGAA
MT444517.1 Severe acute respiratory syndrome coronavirus 2 isolate SARS-CoV-2/human/USA/UT-00069/2020 ORF1	TTCACCGAGGCCACGCGGAGTACGATCGAGTGTACAGTGAA
MT444518.1 Severe acute respiratory syndrome coronavirus 2 isolate SARS-CoV-2/human/USA/UT-00070/2020 ORF1	TTCACCGAGGCCACGCGGAGTACGATCGAGTGTACAGTGAA
MT444519.1 Severe acute respiratory syndrome coronavirus 2 isolate SARS-CoV-2/human/USA/UT-00071/2020 ORF1	TTCACCGAGGCCACGCGGAGTACGATCGAGTGTACAGTGAA
MT444520.1 Severe acute respiratory syndrome coronavirus 2 isolate SARS-CoV-2/human/USA/UT-00072/2020 ORF1	TTCACCGAGGCCACGCGGAGTACGATCGAGTGTACAGTGAA
MT444521.1 Severe acute respiratory syndrome coronavirus 2 isolate SARS-CoV-2/human/USA/UT-00074/2020 ORF1	TTCACCGAGGCCACGCGGAGTACGATCGAGTGTACAGTGAA
MT444522.1 Severe acute respiratory syndrome coronavirus 2 isolate SARS-CoV-2/human/USA/UT-00075/2020 ORF1	TTCACCGAGGCCACGCGGAGTACGATCGAGTGTACAGTGAA
MT444523.1 Severe acute respiratory syndrome coronavirus 2 isolate SARS-CoV-2/human/USA/UT-00077/2020 ORF1	TTCACCGAGGCCACGCGGAGTACGATCGAGTGTACAGTGAA
MT444526.1 Severe acute respiratory syndrome coronavirus 2 isolate SARS-CoV-2/human/USA/UT-00080/2020 ORF1	TTCACCGAGGCCACGCGGAGTACGATCGAGTGTACAGTGAA
MT444527.1 Severe acute respiratory syndrome coronavirus 2 isolate SARS-CoV-2/human/USA/UT-00083/2020 ORF1	TTCACCGAGGCCACGCGGAGTACGATCGAGTGTACAGTGAA
MT444528.1 Severe acute respiratory syndrome coronavirus 2 isolate SARS-CoV-2/human/USA/UT-00084/2020 ORF1	TTCACCGAGGCCACGCGGAGTACGATCGAGTGTACAGTGAA
MT444529.1 Severe acute respiratory syndrome coronavirus 2 isolate SARS-CoV-2/human/USA/UT-00085/2020 ORF1	TTCACCGAGGCCACGCGGAGTACGATCGAGTGTACAGTGAA
MT444530.1 Severe acute respiratory syndrome coronavirus 2 isolate SARS-CoV-2/human/USA/UT-00097/2020 ORF1	TTCACCGAGGCCACGCGGAGTACGATCGAGTGTACAGTGAA
MT444532.1 Severe acute respiratory syndrome coronavirus 2 isolate SARS-CoV-2/human/USA/UT-00099/2020 ORF1	TTCACCGAGGCCACGCGGAGTACGATCGAGTGTACAGTGAA
MT444533.1 Severe acute respiratory syndrome coronavirus 2 isolate SARS-CoV-2/human/USA/UT-00100/2020 ORF1	TTCACCGAGGCCACGCGGAGTACGATCGAGTGTACAGTGAA
MT444534.1 Severe acute respiratory syndrome coronavirus 2 isolate SARS-CoV-2/human/USA/UT-00370/2020 ORF1	TTCACCGAGGCCACGCGGAGTACGATCGAGTGTACAGTGAA
MT444536.1 Severe acute respiratory syndrome coronavirus 2 isolate SARS-CoV-2/human/USA/UT-00443/2020 ORF1	TTCACCGAGGCCACGCGGAGTACGATCGAGTGTACAGTGAA
MT444537.1 Severe acute respiratory syndrome coronavirus 2 isolate SARS-CoV-2/human/USA/UT-00444/2020 ORF1	TTCACCGAGGCCACGCGGAGTACGATCGAGTGTACAGTGAA
MT444539.1 Severe acute respiratory syndrome coronavirus 2 isolate SARS-CoV-2/human/USA/UT-00446/2020 ORF1	TTCACCGAGGCCACGCGGAGTACGATCGAGTGTACAGTGAA
MT444540.1 Severe acute respiratory syndrome coronavirus 2 isolate SARS-CoV-2/human/USA/UT-00447/2020, com	TTCACCGAGGCCACGCGGAGTACGATCGAGTGTACAGTGAA
MT444542.1 Severe acute respiratory syndrome coronavirus 2 isolate SARS-CoV-2/human/USA/UT-00492/2020 ORF1	TTCACCGAGGCCACGCGGAGTACGATCGAGTGTACAGTGAA
MT444552.1 Severe acute respiratory syndrome coronavirus 2 isolate SARS-CoV-2/human/USA/UT-00511/2020 ORF1	TTCACCGAGGCCACGCGGAGTACGATCGAGTGTACAGTGAA
MT444553.1 Severe acute respiratory syndrome coronavirus 2 isolate SARS-CoV-2/human/USA/UT-00513/2020 ORF1	TTCACCGAGGCCACGCGGAGTACGATCGAGTGTACAGTGAA
MT444555.1 Severe acute respiratory syndrome coronavirus 2 isolate SARS-CoV-2/human/USA/UT-00520/2020 ORF1	TTCACCGAGGCCACGCGGAGTACGATCGAGTGTACAGTGAA
MT444561.1 Severe acute respiratory syndrome coronavirus 2 isolate SARS-CoV-2/human/USA/UT-00106/2020 ORF1	TTCACCGAGGCCACGCGGAGTACGATCGAGTGTACAGTGAA
MT444565.1 Severe acute respiratory syndrome coronavirus 2 isolate SARS-CoV-2/human/USA/UT-00366/2020 ORF1	TTCACCGAGGCCACGCGGAGTACGATCGAGTGTACAGTGAA
MT444566.1 Severe acute respiratory syndrome coronavirus 2 isolate SARS-CoV-2/human/USA/UT-00367/2020 ORF1	TTCACCGAGGCCACGCGGAGTACGATCGAGTGTACAGTGAA
MT444568.1 Severe acute respiratory syndrome coronavirus 2 isolate SARS-CoV-2/human/USA/UT-00369/2020 ORF1	TTCACCGAGGCCACGCGGAGTACGATCGAGTGTACAGTGAA
MT444570.1 Severe acute respiratory syndrome coronavirus 2 isolate SARS-CoV-2/human/USA/UT-00522/2020 ORF1	TTCACCGAGGCCACGCGGAGTACGATCGAGTGTACAGTGAA
MT444571.1 Severe acute respiratory syndrome coronavirus 2 isolate SARS-CoV-2/human/USA/UT-00524/2020 ORF1	TTCACCGAGGCCACGCGGAGTACGATCGAGTGTACAGTGAA

MT444572.1 Severe acute respiratory syndrome coronavirus 2 isolate SARS-CoV-2/human/USA/UT-00526/2020 ORF1	TTCACCGAGGCCACGCGGAGTACGATCGAGTGTACAGTGAA
MT444573.1 Severe acute respiratory syndrome coronavirus 2 isolate SARS-CoV-2/human/USA/UT-00527/2020 ORF1	TTCACCGAGGCCACGCGGAGTACGATCGAGTGTACAGTGAA
MT444574.1 Severe acute respiratory syndrome coronavirus 2 isolate SARS-CoV-2/human/USA/UT-00529/2020 ORF1	TTCACCGAGGCCACGCGGAGTACGATCGAGTGTACAGTGAA
MT444575.1 Severe acute respiratory syndrome coronavirus 2 isolate SARS-CoV-2/human/USA/UT-00531/2020 ORF1	TTCACCGAGGCCACGCGGAGTACGATCGAGTGTACAGTGAA
MT444580.1 Severe acute respiratory syndrome coronavirus 2 isolate SARS-CoV-2/human/USA/UT-00537/2020 ORF1	TTCACCGAGGCCACGCGGAGTACGATCGAGTGTACAGTGAA
MT444581.1 Severe acute respiratory syndrome coronavirus 2 isolate SARS-CoV-2/human/USA/UT-00539/2020 ORF1	TTCACCGAGGCCACGCGGAGTACGATCGAGTGTACAGTGAA
MT444583.1 Severe acute respiratory syndrome coronavirus 2 isolate SARS-CoV-2/human/USA/UT-00541/2020 ORF1	TTCACCGAGGCCACGCGGAGTACGATCGAGTGTACAGTGAA
MT444587.1 Severe acute respiratory syndrome coronavirus 2 isolate SARS-CoV-2/human/USA/UT-00545/2020 ORF1	TTCACCGAGGCCACGCGGAGTACGATCGAGTGTACAGTGAA
MT444589.1 Severe acute respiratory syndrome coronavirus 2 isolate SARS-CoV-2/human/USA/UT-00678/2020 ORF1	TTCACCGAGGCCACGCGGAGTACGATCGAGTGTACAGTGAA
MT444590.1 Severe acute respiratory syndrome coronavirus 2 isolate SARS-CoV-2/human/USA/UT-00679/2020 ORF1	TTCACCGAGGCCACGCGGAGTACGATCGAGTGTACAGTGAA
MT444591.1 Severe acute respiratory syndrome coronavirus 2 isolate SARS-CoV-2/human/USA/UT-00680/2020 ORF1	TTCACCGAGGCCACGCGGAGTACGATCGAGTGTACAGTGAA
MT444593.1 Severe acute respiratory syndrome coronavirus 2 isolate SARS-CoV-2/human/USA/UT-00884/2020 ORF1	TTCACCGAGGCCACGCGGAGTACGATCGAGTGTACAGTGAA
MT444594.1 Severe acute respiratory syndrome coronavirus 2 isolate SARS-CoV-2/human/USA/UT-00885/2020 ORF1	TTCACCGAGGCCACGCGGAGTACGATCGAGTGTACAGTGAA
MT444595.1 Severe acute respiratory syndrome coronavirus 2 isolate SARS-CoV-2/human/USA/UT-00886/2020 ORF1	TTCACCGAGGCCACGCGGAGTACGATCGAGTGTACAGTGAA
MT444596.1 Severe acute respiratory syndrome coronavirus 2 isolate SARS-CoV-2/human/USA/UT-00887/2020 ORF1	TTCACCGAGGCCACGCGGAGTACGATCGAGTGTACAGTGAA
MT444598.1 Severe acute respiratory syndrome coronavirus 2 isolate SARS-CoV-2/human/USA/UT-00894/2020 ORF1	TTCACCGAGGCCACGCGGAGTACGATCGAGTGTACAGTGAA
MT444600.1 Severe acute respiratory syndrome coronavirus 2 isolate SARS-CoV-2/human/USA/UT-00896/2020 ORF1	TTCACCGAGGCCACGCGGAGTACGATCGAGTGTACAGTGAA
MT444602.1 Severe acute respiratory syndrome coronavirus 2 isolate SARS-CoV-2/human/USA/UT-00971/2020 ORF1	TTCACCGAGGCCACGCGGAGTACGATCGAGTGTACAGTGAA
MT444603.1 Severe acute respiratory syndrome coronavirus 2 isolate SARS-CoV-2/human/USA/UT-01216/2020 ORF1	TTCACCGAGGCCACGCGGAGTACGATCGAGTGTACAGTGAA
MT444604.1 Severe acute respiratory syndrome coronavirus 2 isolate SARS-CoV-2/human/USA/UT-01217/2020 ORF1	TTCACCGAGGCCACGCGGAGTACGATCGAGTGTACAGTGAA
MT444605.1 Severe acute respiratory syndrome coronavirus 2 isolate SARS-CoV-2/human/USA/UT-01218/2020 ORF1	TTCACCGAGGCCACGCGGAGTACGATCGAGTGTACAGTGAA
MT444607.1 Severe acute respiratory syndrome coronavirus 2 isolate SARS-CoV-2/human/USA/UT-01222/2020 ORF1	TTCACCGAGGCCACGCGGAGTACGATCGAGTGTACAGTGAA
MT444609.1 Severe acute respiratory syndrome coronavirus 2 isolate SARS-CoV-2/human/USA/UT-01227/2020 ORF1	TTCACCGAGGCCACGCGGAGTACGATCGAGTGTACAGTGAA
MT444610.1 Severe acute respiratory syndrome coronavirus 2 isolate SARS-CoV-2/human/USA/UT-01229/2020 ORF1	TTCACCGAGGCCACGCGGAGTACGATCGAGTGTACAGTGAA
MT444612.1 Severe acute respiratory syndrome coronavirus 2 isolate SARS-CoV-2/human/USA/UT-01434/2020 ORF1	TTCACCGAGGCCACGCGGAGTACGATCGAGTGTACAGTGAA
MT444613.1 Severe acute respiratory syndrome coronavirus 2 isolate SARS-CoV-2/human/USA/UT-01435/2020 ORF1	TTCACCGAGGCCACGCGGAGTACGATCGAGTGTACAGTGAA
MT444614.1 Severe acute respiratory syndrome coronavirus 2 isolate SARS-CoV-2/human/USA/UT-01436/2020 ORF1	TTCACCGAGGCCACGCGGAGTACGATCGAGTGTACAGTGAA
MT444616.1 Severe acute respiratory syndrome coronavirus 2 isolate SARS-CoV-2/human/USA/UT-02218/2020 ORF1	TTCACCGAGGCCACGCGGAGTACGATCGAGTGTACAGTGAA
MT444617.1 Severe acute respiratory syndrome coronavirus 2 isolate SARS-CoV-2/human/USA/UT-02219/2020 ORF1	TTCACCGAGGCCACGCGGAGTACGATCGAGTGTACAGTGAA
MT444618.1 Severe acute respiratory syndrome coronavirus 2 isolate SARS-CoV-2/human/USA/UT-02220/2020 ORF1	TTCACCGAGGCCACGCGGAGTACGATCGAGTGTACAGTGAA
MT444619.1 Severe acute respiratory syndrome coronavirus 2 isolate SARS-CoV-2/human/USA/UT-02221/2020, com	TTCACCGAGGCCACGCGGAGTACGATCGAGTGTACAGTGAA
MT444620.1 Severe acute respiratory syndrome coronavirus 2 isolate SARS-CoV-2/human/USA/UT-02222/2020 ORF1	TTCACCGAGGCCACGCGGAGTACGATCGAGTGTACAGTGAA
MT444621.1 Severe acute respiratory syndrome coronavirus 2 isolate SARS-CoV-2/human/USA/UT-02223/2020 ORF1	TTCACCGAGGCCACGCGGAGTACGATCGAGTGTACAGTGAA

MT444622.1 Severe acute respiratory syndrome coronavirus 2 isolate SARS-CoV-2/human/USA/UT-02224/2020 ORF1	TTCACCGAGGCCACGCGGAGTACGATCGAGTGTACAGTGAA
MT444623.1 Severe acute respiratory syndrome coronavirus 2 isolate SARS-CoV-2/human/USA/UT-02227/2020 ORF1	TTCACCGAGGCCACGCGGAGTACGATCGAGTGTACAGTGAA
MT444624.1 Severe acute respiratory syndrome coronavirus 2 isolate SARS-CoV-2/human/USA/UT-02228/2020, com	TTCACCGAGGCCACGCGGAGTACGATCGAGTGTACAGTGAA
MT444625.1 Severe acute respiratory syndrome coronavirus 2 isolate SARS-CoV-2/human/USA/UT-02229/2020, com	TTCACCGAGGCCACGCGGAGTACGATCGAGTGTACAGTGAA
MT444626.1 Severe acute respiratory syndrome coronavirus 2 isolate SARS-CoV-2/human/USA/UT-02231/2020, com	TTCACCGAGGCCACGCGGAGTACGATCGAGTGTACAGTGAA
MT444627.1 Severe acute respiratory syndrome coronavirus 2 isolate SARS-CoV-2/human/USA/UT-02232/2020 ORF1	TTCACCGAGGCCACGCGGAGTACGATCGAGTGTACAGTGAA
MT444628.1 Severe acute respiratory syndrome coronavirus 2 isolate SARS-CoV-2/human/USA/UT-02233/2020 ORF1	TTCACCGAGGCCACGCGGAGTACGATCGAGTGTACAGTGAA
MT444629.1 Severe acute respiratory syndrome coronavirus 2 isolate SARS-CoV-2/human/USA/UT-02234/2020 ORF1	TTCACCGAGGCCACGCGGAGTACGATCGAGTGTACAGTGAA
MT444631.1 Severe acute respiratory syndrome coronavirus 2 isolate SARS-CoV-2/human/USA/UT-02242/2020 ORF1	TTCACCGAGGCCACGCGGAGTACGATCGAGTGTACAGTGAA
MT444632.1 Severe acute respiratory syndrome coronavirus 2 isolate SARS-CoV-2/human/USA/UT-02243/2020 ORF1	TTCACCGAGGCCACGCGGAGTACGATCGAGTGTACAGTGAA
MT444633.1 Severe acute respiratory syndrome coronavirus 2 isolate SARS-CoV-2/human/USA/UT-02244/2020, com	TTCACCGAGGCCACGCGGAGTACGATCGAGTGTACAGTGAA
MT444634.1 Severe acute respiratory syndrome coronavirus 2 isolate SARS-CoV-2/human/USA/UT-02245/2020 ORF1	TTCACCGAGGCCACGCGGAGTACGATCGAGTGTACAGTGAA
MT444635.1 Severe acute respiratory syndrome coronavirus 2 isolate SARS-CoV-2/human/USA/UT-02247/2020, com	TTCACCGAGGCCACGCGGAGTACGATCGAGTGTACAGTGAA
MT446312.1 Severe acute respiratory syndrome coronavirus 2 isolate SARS-CoV-2/human/Guangzhou/IQTC05/2020,	TTCACCGAGGCCACGCGGAGTACGATCGAGTGTACAGTGAA
MT446339.1 Severe acute respiratory syndrome coronavirus 2 isolate SARS-CoV-2/human/USA/LA-BIE-096/2020, cor	TTCACCGAGGCCACGCGGAGTACGATCGAGTGTACAGTGAA
MT446340.1 Severe acute respiratory syndrome coronavirus 2 isolate SARS-CoV-2/human/USA/LA-BIE-072/2020 ORF	TTCACCGAGGCCACGCGGAGTACGATCGAGTGTACAGTGAA
MT446341.1 Severe acute respiratory syndrome coronavirus 2 isolate SARS-CoV-2/human/USA/LA-BIE-073/2020 ORF	TTCACCGAGGCCACGCGGAGTACGATCGAGTGTACAGTGAA
MT446342.1 Severe acute respiratory syndrome coronavirus 2 isolate SARS-CoV-2/human/USA/LA-BIE-074/2020, cor	TTCACCGAGGCCACGCGGAGTACGATCGAGTGTACAGTGAA
MT446343.1 Severe acute respiratory syndrome coronavirus 2 isolate SARS-CoV-2/human/USA/LA-BIE-075/2020, cor	TTCACCGAGGCCACGCGGAGTACGATCGAGTGTACAGTGAA
MT446344.1 Severe acute respiratory syndrome coronavirus 2 isolate SARS-CoV-2/human/USA/LA-BIE-076/2020, cor	TTCACCGAGGCCACGCGGAGTACGATCGAGTGTACAGTGAA
MT446345.1 Severe acute respiratory syndrome coronavirus 2 isolate SARS-CoV-2/human/USA/LA-BIE-077/2020, cor	TTCACCGAGGCCACGCGGAGTACGATCGAGTGTACAGTGAA
MT446346.1 Severe acute respiratory syndrome coronavirus 2 isolate SARS-CoV-2/human/USA/LA-BIE-078/2020, cor	TTCACCGAGGCCACGCGGAGTACGATCGAGTGTACAGTGAA
MT446347.1 Severe acute respiratory syndrome coronavirus 2 isolate SARS-CoV-2/human/USA/LA-BIE-079/2020, cor	TTCACCGAGGCCACGCGGAGTACGATCGAGTGTACAGTGAA
MT446348.1 Severe acute respiratory syndrome coronavirus 2 isolate SARS-CoV-2/human/USA/LA-BIE-080/2020, cor	TTCACCGAGGCCACGCGGAGTACGATCGAGTGTACAGTGAA
MT446349.1 Severe acute respiratory syndrome coronavirus 2 isolate SARS-CoV-2/human/USA/LA-BIE-081/2020, cor	TTCACCGAGGCCACGCGGAGTACGATCGAGTGTACAGTGAA
MT446350.1 Severe acute respiratory syndrome coronavirus 2 isolate SARS-CoV-2/human/USA/LA-BIE-082/2020, cor	TTCACCGAGGCCACGCGGAGTACGATCGAGTGTACAGTGAA
MT446351.1 Severe acute respiratory syndrome coronavirus 2 isolate SARS-CoV-2/human/USA/LA-BIE-083/2020 ORF	TTCACCGAGGCCACGCGGAGTACGATCGAGTGTACAGTGAA
MT446352.1 Severe acute respiratory syndrome coronavirus 2 isolate SARS-CoV-2/human/USA/LA-BIE-084/2020, cor	TTCACCGAGGCCACGCGGAGTACGATCGAGTGTACAGTGAA
MT446353.1 Severe acute respiratory syndrome coronavirus 2 isolate SARS-CoV-2/human/USA/LA-BIE-085/2020, cor	TTCACCGAGGCCACGCGGAGTACGATCGAGTGTACAGTGAA
MT446354.1 Severe acute respiratory syndrome coronavirus 2 isolate SARS-CoV-2/human/USA/LA-BIE-086/2020, cor	TTCACCGAGGCCACGCGGAGTACGATCGAGTGTACAGTGAA
MT446355.1 Severe acute respiratory syndrome coronavirus 2 isolate SARS-CoV-2/human/USA/LA-BIE-087/2020 ORF	TTCACCGAGGCCACGCGGAGTACGATCGAGTGTACAGTGAA
MT446357.1 Severe acute respiratory syndrome coronavirus 2 isolate SARS-CoV-2/human/USA/LA-BIE-089/2020, cor	TTCACCGAGGCCACGCGGAGTACGATCGAGTGTACAGTGAA
MT446358.1 Severe acute respiratory syndrome coronavirus 2 isolate SARS-CoV-2/human/USA/LA-BIE-090/2020, cor	TTCACCGAGGCCACGCGGAGTACGATCGAGTGTACAGTGAA

MT446359.1 Severe acute respiratory syndrome coronavirus 2 isolate SARS-CoV-2/human/USA/LA-BIE-092/2020, cor	TTCACCGAGGCCACGCGGAGTACGATCGAGTGTACAGTGAA
MT446361.1 Severe acute respiratory syndrome coronavirus 2 isolate SARS-CoV-2/human/USA/LA-BIE-094/2020 ORF	TTCACCGAGGCCACGCGGAGTACGATCGAGTGTACAGTGAA
MT447154.1 Severe acute respiratory syndrome coronavirus 2 isolate SARS-CoV-2/human/THA/SI200893-NT/2020, co	TTCACCGAGGCCACGCGGAGTACGATCGAGTGTACAGTGAA
MT447155.1 Severe acute respiratory syndrome coronavirus 2 isolate SARS-CoV-2/human/THA/SI200040-NT/2020, co	TTCACCGAGGCCACGCGGAGTACGATCGAGTGTACAGTGAA
MT447156.1 Severe acute respiratory syndrome coronavirus 2 isolate SARS-CoV-2/human/THA/SI200383-NT/2020, co	TTCACCGAGGCCACGCGGAGTACGATCGAGTGTACAGTGAA
MT447157.1 Severe acute respiratory syndrome coronavirus 2 isolate SARS-CoV-2/human/THA/SI200433-NT/2020, co	TTCACCGAGGCCACGCGGAGTACGATCGAGTGTACAGTGAA
MT447158.1 Severe acute respiratory syndrome coronavirus 2 isolate SARS-CoV-2/human/THA/SI200468-NT/2020, co	TTCACCGAGGCCACGCGGAGTACGATCGAGTGTACAGTGAA
MT447159.1 Severe acute respiratory syndrome coronavirus 2 isolate SARS-CoV-2/human/THA/SI200478-NST/2020,	TTCACCGAGGCCACGCGGAGTACGATCGAGTGTACAGTGAA
MT447161.1 Severe acute respiratory syndrome coronavirus 2 isolate SARS-CoV-2/human/THA/SI201712-NT/2020, co	TTCACCGAGGCCACGCGGAGTACGATCGAGTGTACAGTGAA
MT447162.1 Severe acute respiratory syndrome coronavirus 2 isolate SARS-CoV-2/human/THA/SI202205-NT/2020, co	TTCACCGAGGCCACGCGGAGTACGATCGAGTGTACAGTGAA
MT447163.1 Severe acute respiratory syndrome coronavirus 2 isolate SARS-CoV-2/human/THA/SI202620-NT/2020, co	TTCACCGAGGCCACGCGGAGTACGATCGAGTGTACAGTGAA
MT447166.1 Severe acute respiratory syndrome coronavirus 2 isolate SARS-CoV-2/human/THA/SI203285-NT/2020, co	TTCACCGAGGCCACGCGGAGTACGATCGAGTGTACAGTGAA
MT447169.1 Severe acute respiratory syndrome coronavirus 2 isolate SARS-CoV-2/human/THA/SI204514-NT/2020, co	TTCACCGAGGCCACGCGGAGTACGATCGAGTGTACAGTGAA
MT447171.1 Severe acute respiratory syndrome coronavirus 2 isolate SARS-CoV-2/human/THA/SI205457-NST/2020,	TTCACCGAGGCCACGCGGAGTACGATCGAGTGTACAGTGAA
MT447172.1 Severe acute respiratory syndrome coronavirus 2 isolate SARS-CoV-2/human/THA/SI205529-NT/2020, co	TTCACCGAGGCCACGCGGAGTACGATCGAGTGTACAGTGAA
MT447173.1 Severe acute respiratory syndrome coronavirus 2 isolate SARS-CoV-2/human/THA/SI205929-NT/2020, co	TTCACCGAGGCCACGCGGAGTACGATCGAGTGTACAGTGAA
MT447174.1 Severe acute respiratory syndrome coronavirus 2 isolate SARS-CoV-2/human/THA/SI206377-NST/2020,	TTCACCGAGGCCACGCGGAGTACGATCGAGTGTACAGTGAA
MT447175.1 Severe acute respiratory syndrome coronavirus 2 isolate SARS-CoV-2/human/THA/SI206587-NT/2020, co	TTCACCGAGGCCACGCGGAGTACGATCGAGTGTACAGTGAA
MT447176.1 Severe acute respiratory syndrome coronavirus 2 isolate SARS-CoV-2/human/THA/SI206917-NT/2020, co	TTCACCGAGGCCACGCGGAGTACGATCGAGTGTACAGTGAA
MT449637.1 Severe acute respiratory syndrome coronavirus 2 isolate SARS-CoV-2/human/USA/CA-CZB-1044/2020, c	TTCACCGAGGCCACGCGGAGTACGATCGAGTGTACAGTGAA
MT449638.1 Severe acute respiratory syndrome coronavirus 2 isolate SARS-CoV-2/human/USA/CA-CZB-1045/2020 O	TTCACCGAGGCCACGCGGAGTACGATCGAGTGTACAGTGAA
MT449639.1 Severe acute respiratory syndrome coronavirus 2 isolate SARS-CoV-2/human/USA/CA-CZB-1046/2020, c	TTCACCGAGGCCACGCGGAGTACGATCGAGTGTACAGTGAA
MT449640.1 Severe acute respiratory syndrome coronavirus 2 isolate SARS-CoV-2/human/USA/CA-CZB-1047/2020 O	TTCACCGAGGCCACGCGGAGTACGATCGAGTGTACAGTGAA
MT449641.1 Severe acute respiratory syndrome coronavirus 2 isolate SARS-CoV-2/human/USA/CA-CZB-1048/2020, c	TTCACCGAGGCCACGCGGAGTACGATCGAGTGTACAGTGAA
MT449642.1 Severe acute respiratory syndrome coronavirus 2 isolate SARS-CoV-2/human/USA/CA-CZB-1049/2020 O	TTCACCGAGGCCACGCGGAGTACGATCGAGTGTACAGTGAA
MT449643.1 Severe acute respiratory syndrome coronavirus 2 isolate SARS-CoV-2/human/USA/CA-CZB-1050/2020 O	TTCACCGAGGCCACGCGGAGTACGATCGAGTGTACAGTGAA
MT449644.1 Severe acute respiratory syndrome coronavirus 2 isolate SARS-CoV-2/human/USA/CA-CZB-1051/2020, c	TTCACCGAGGCCACGCGGAGTACGATCGAGTGTACAGTGAA
MT449646.1 Severe acute respiratory syndrome coronavirus 2 isolate SARS-CoV-2/human/USA/CA-CZB-1054/2020 O	TTCACCGAGGCCACGCGGAGTACGATCGAGTGTACAGTGAA
MT449647.1 Severe acute respiratory syndrome coronavirus 2 isolate SARS-CoV-2/human/USA/CA-CZB-1056/2020, c	TTCACCGAGGCCACGCGGAGTACGATCGAGTGTACAGTGAA
MT449648.1 Severe acute respiratory syndrome coronavirus 2 isolate SARS-CoV-2/human/USA/CA-CZB-1057/2020 O	TTCACCGAGGCCACGCGGAGTACGATCGAGTGTACAGTGAA
MT449649.1 Severe acute respiratory syndrome coronavirus 2 isolate SARS-CoV-2/human/USA/CA-CZB-1058/2020 O	TTCACCGAGGCCACGCGGAGTACGATCGAGTGTACAGTGAA
MT449651.1 Severe acute respiratory syndrome coronavirus 2 isolate SARS-CoV-2/human/USA/CA-CZB-1060/2020, c	TTCACCGAGGCCACGCGGAGTACGATCGAGTGTACAGTGAA
MT449652.1 Severe acute respiratory syndrome coronavirus 2 isolate SARS-CoV-2/human/USA/CA-CZB-1061/2020 O	TTCACCGAGGCCACGCGGAGTACGATCGAGTGTACAGTGAA

MT449653.1 Severe acute respiratory syndrome coronavirus 2 isolate SARS-CoV-2/human/USA/CA-CZB-1062/2020 O	TTCACCGAGGCCACGCGGAGTACGATCGAGTGTACAGTGAA
MT449654.1 Severe acute respiratory syndrome coronavirus 2 isolate SARS-CoV-2/human/USA/CA-CZB-1063/2020 O	TTCACCGAGGCCACGCGGAGTACGATCGAGTGTACAGTGAA
MT449655.1 Severe acute respiratory syndrome coronavirus 2 isolate SARS-CoV-2/human/USA/CA-CZB-1064/2020 O	TTCACCGAGGCCACGCGGAGTACGATCGAGTGTACAGTGAA
MT449656.1 Severe acute respiratory syndrome coronavirus 2 isolate SARS-CoV-2/human/USA/CA-CZB-1065/2020, c	TTCACCGAGGCCACGCGGAGTACGATCGAGTGTACAGTGAA
MT449657.1 Severe acute respiratory syndrome coronavirus 2 isolate SARS-CoV-2/human/USA/CA-CZB-1066/2020 O	TTCACCGAGGCCACGCGGAGTACGATCGAGTGTACAGTGAA
MT449658.1 Severe acute respiratory syndrome coronavirus 2 isolate SARS-CoV-2/human/USA/CA-CZB-1067/2020 O	TTCACCGAGGCCACGCGGAGTACGATCGAGTGTACAGTGAA
MT449659.1 Severe acute respiratory syndrome coronavirus 2 isolate SARS-CoV-2/human/USA/CA-CZB-1068/2020 O	TTCACCGAGGCCACGCGGAGTACGATCGAGTGTACAGTGAA
MT449660.1 Severe acute respiratory syndrome coronavirus 2 isolate SARS-CoV-2/human/USA/CA-CZB-1069/2020 O	TTCACCGAGGCCACGCGGAGTACGATCGAGTGTACAGTGAA
MT449662.1 Severe acute respiratory syndrome coronavirus 2 isolate SARS-CoV-2/human/USA/CA-CZB-1071/2020 O	TTCACCGAGGCCACGCGGAGTACGATCGAGTGTACAGTGAA
MT449663.1 Severe acute respiratory syndrome coronavirus 2 isolate SARS-CoV-2/human/USA/CA-CZB-1072/2020 O	TTCACCGAGGCCACGCGGAGTACGATCGAGTGTACAGTGAA
MT449664.1 Severe acute respiratory syndrome coronavirus 2 isolate SARS-CoV-2/human/USA/CA-CZB-1073/2020, c	TTCACCGAGGCCACGCGGAGTACGATCGAGTGTACAGTGAA
MT449665.1 Severe acute respiratory syndrome coronavirus 2 isolate SARS-CoV-2/human/USA/CA-CZB-1074/2020, c	TTCACCGAGGCCACGCGGAGTACGATCGAGTGTACAGTGAA
MT449666.1 Severe acute respiratory syndrome coronavirus 2 isolate SARS-CoV-2/human/USA/CA-CZB-1075/2020 O	TTCACCGAGGCCACGCGGAGTACGATCGAGTGTACAGTGAA
MT449667.1 Severe acute respiratory syndrome coronavirus 2 isolate SARS-CoV-2/human/USA/CA-CZB-1076/2020 O	TTCACCGAGGCCACGCGGAGTACGATCGAGTGTACAGTGAA
MT449668.1 Severe acute respiratory syndrome coronavirus 2 isolate SARS-CoV-2/human/USA/CA-CZB-1077/2020 O	TTCACCGAGGCCACGCGGAGTACGATCGAGTGTACAGTGAA
MT449669.1 Severe acute respiratory syndrome coronavirus 2 isolate SARS-CoV-2/human/USA/CA-CZB-1078/2020 O	TTCACCGAGGCCACGCGGAGTACGATCGAGTGTACAGTGAA
MT449670.1 Severe acute respiratory syndrome coronavirus 2 isolate SARS-CoV-2/human/USA/CA-CZB-1079/2020, c	TTCACCGAGGCCACGCGGAGTACGATCGAGTGTACAGTGAA
MT449671.1 Severe acute respiratory syndrome coronavirus 2 isolate SARS-CoV-2/human/USA/CA-CZB-1080/2020 O	TTCACCGAGGCCACGCGGAGTACGATCGAGTGTACAGTGAA
MT449672.1 Severe acute respiratory syndrome coronavirus 2 isolate SARS-CoV-2/human/USA/CA-CZB-1081/2020, c	TTCACCGAGGCCACGCGGAGTACGATCGAGTGTACAGTGAA
MT449673.1 Severe acute respiratory syndrome coronavirus 2 isolate SARS-CoV-2/human/USA/CA-CZB-1082/2020, c	TTCACCGAGGCCACGCGGAGTACGATCGAGTGTACAGTGAA
MT449674.1 Severe acute respiratory syndrome coronavirus 2 isolate SARS-CoV-2/human/USA/CA-CZB-1083/2020 O	TTCACCGAGGCCACGCGGAGTACGATCGAGTGTACAGTGAA
MT449675.1 Severe acute respiratory syndrome coronavirus 2 isolate SARS-CoV-2/human/USA/CA-CZB-1084/2020 O	TTCACCGAGGCCACGCGGAGTACGATCGAGTGTACAGTGAA
MT449676.1 Severe acute respiratory syndrome coronavirus 2 isolate SARS-CoV-2/human/USA/CA-CZB-1085/2020 O	TTCACCGAGGCCACGCGGAGTACGATCGAGTGTACAGTGAA
MT449677.1 Severe acute respiratory syndrome coronavirus 2 isolate SARS-CoV-2/human/USA/CA-CZB-1086/2020 O	TTCACCGAGGCCACGCGGAGTACGATCGAGTGTACAGTGAA
MT449678.1 Severe acute respiratory syndrome coronavirus 2 isolate SARS-CoV-2/human/USA/CA-CZB-1087/2020 O	TTCACCGAGGCCACGCGGAGTACGATCGAGTGTACAGTGAA
MT449679.1 Severe acute respiratory syndrome coronavirus 2 isolate SARS-CoV-2/human/USA/CA-CZB-1088/2020 O	TTCACCGAGGCCACGCGGAGTACGATCGAGTGTACAGTGAA
MT450872.1 Severe acute respiratory syndrome coronavirus 2 isolate SARS-CoV-2/human/SRB/KV26/2020, complete	TTCACCGAGGCCACGCGGAGTACGATCGAGTGTACAGTGAA
MT450875.1 Severe acute respiratory syndrome coronavirus 2 isolate SARS-CoV-2/human/USA/VA-DCLS-0173/2020,	TTCACCGAGGCCACGCGGAGTACGATCGAGTGTACAGTGAA
MT450877.1 Severe acute respiratory syndrome coronavirus 2 isolate SARS-CoV-2/human/USA/VA-DCLS-0175/2020,	TTCACCGAGGCCACGCGGAGTACGATCGAGTGTACAGTGAA
MT450879.1 Severe acute respiratory syndrome coronavirus 2 isolate SARS-CoV-2/human/USA/VA-DCLS-0177/2020,	TTCACCGAGGCCACGCGGAGTACGATCGAGTGTACAGTGAA
MT450880.1 Severe acute respiratory syndrome coronavirus 2 isolate SARS-CoV-2/human/USA/VA-DCLS-0178/2020,	TTCACCGAGGCCACGCGGAGTACGATCGAGTGTACAGTGAA
MT450882.1 Severe acute respiratory syndrome coronavirus 2 isolate SARS-CoV-2/human/USA/VA-DCLS-0180/2020,	TTCACCGAGGCCACGCGGAGTACGATCGAGTGTACAGTGAA
MT450885.1 Severe acute respiratory syndrome coronavirus 2 isolate SARS-CoV-2/human/USA/VA-DCLS-0183/2020,	TTCACCGAGGCCACGCGGAGTACGATCGAGTGTACAGTGAA

MT450888.1 Severe acute respiratory syndrome coronavirus 2 isolate SARS-CoV-2/human/USA/VA-DCLS-0186/2020	TTCACCGAGGCCACGCGGAGTACGATCGAGTGTACAGTGAA
MT450891.1 Severe acute respiratory syndrome coronavirus 2 isolate SARS-CoV-2/human/USA/VA-DCLS-0189/2020,	TTCACCGAGGCCACGCGGAGTACGATCGAGTGTACAGTGAA
MT450892.1 Severe acute respiratory syndrome coronavirus 2 isolate SARS-CoV-2/human/USA/VA-DCLS-0190/2020,	TTCACCGAGGCCACGCGGAGTACGATCGAGTGTACAGTGAA
MT450893.1 Severe acute respiratory syndrome coronavirus 2 isolate SARS-CoV-2/human/USA/VA-DCLS-0191/2020,	TTCACCGAGGCCACGCGGAGTACGATCGAGTGTACAGTGAA
MT450895.1 Severe acute respiratory syndrome coronavirus 2 isolate SARS-CoV-2/human/USA/VA-DCLS-0194/2020,	TTCACCGAGGCCACGCGGAGTACGATCGAGTGTACAGTGAA
MT450897.1 Severe acute respiratory syndrome coronavirus 2 isolate SARS-CoV-2/human/USA/VA-DCLS-0196/2020,	TTCACCGAGGCCACGCGGAGTACGATCGAGTGTACAGTGAA
MT450900.1 Severe acute respiratory syndrome coronavirus 2 isolate SARS-CoV-2/human/USA/VA-DCLS-0199/2020,	TTCACCGAGGCCACGCGGAGTACGATCGAGTGTACAGTGAA
MT450901.1 Severe acute respiratory syndrome coronavirus 2 isolate SARS-CoV-2/human/USA/VA-DCLS-0200/2020,	TTCACCGAGGCCACGCGGAGTACGATCGAGTGTACAGTGAA
MT450903.1 Severe acute respiratory syndrome coronavirus 2 isolate SARS-CoV-2/human/USA/VA-DCLS-0202/2020,	TTCACCGAGGCCACGCGGAGTACGATCGAGTGTACAGTGAA
MT450904.1 Severe acute respiratory syndrome coronavirus 2 isolate SARS-CoV-2/human/USA/VA-DCLS-0203/2020,	TTCACCGAGGCCACGCGGAGTACGATCGAGTGTACAGTGAA
MT450906.1 Severe acute respiratory syndrome coronavirus 2 isolate SARS-CoV-2/human/USA/VA-DCLS-0205/2020,	TTCACCGAGGCCACGCGGAGTACGATCGAGTGTACAGTGAA
MT450907.1 Severe acute respiratory syndrome coronavirus 2 isolate SARS-CoV-2/human/USA/VA-DCLS-0206/2020,	TTCACCGAGGCCACGCGGAGTACGATCGAGTGTACAGTGAA
MT450908.1 Severe acute respiratory syndrome coronavirus 2 isolate SARS-CoV-2/human/USA/VA-DCLS-0207/2020	TTCACCGAGGCCACGCGGAGTACGATCGAGTGTACAGTGAA
MT450909.1 Severe acute respiratory syndrome coronavirus 2 isolate SARS-CoV-2/human/USA/VA-DCLS-0208/2020,	TTCACCGAGGCCACGCGGAGTACGATCGAGTGTACAGTGAA
MT450911.1 Severe acute respiratory syndrome coronavirus 2 isolate SARS-CoV-2/human/USA/VA-DCLS-0210/2020,	TTCACCGAGGCCACGCGGAGTACGATCGAGTGTACAGTGAA
MT450912.1 Severe acute respiratory syndrome coronavirus 2 isolate SARS-CoV-2/human/USA/VA-DCLS-0211/2020,	TTCACCGAGGCCACGCGGAGTACGATCGAGTGTACAGTGAA
MT450915.1 Severe acute respiratory syndrome coronavirus 2 isolate SARS-CoV-2/human/USA/VA-DCLS-0214/2020,	TTCACCGAGGCCACGCGGAGTACGATCGAGTGTACAGTGAA
MT450916.1 Severe acute respiratory syndrome coronavirus 2 isolate SARS-CoV-2/human/USA/VA-DCLS-0215/2020	TTCACCGAGGCCACGCGGAGTACGATCGAGTGTACAGTGAA
MT450917.1 Severe acute respiratory syndrome coronavirus 2 isolate SARS-CoV-2/human/USA/VA-DCLS-0216/2020,	TTCACCGAGGCCACGCGGAGTACGATCGAGTGTACAGTGAA
MT450918.1 Severe acute respiratory syndrome coronavirus 2 isolate SARS-CoV-2/human/USA/VA-DCLS-0217/2020,	TTCACCGAGGCCACGCGGAGTACGATCGAGTGTACAGTGAA
MT450920.1 Severe acute respiratory syndrome coronavirus 2 isolate SARS-CoV-2/human/AUS/VIC03/2020 ORF1ab	TTCACCGAGGCCACGCGGAGTACGATCGAGTGTACAGTGAA
MT450922.1 Severe acute respiratory syndrome coronavirus 2 isolate SARS-CoV-2/human/AUS/VIC05/2020 ORF1ab	TTCACCGAGGCCACGCGGAGTACGATCGAGTGTACAGTGAA
MT450924.1 Severe acute respiratory syndrome coronavirus 2 isolate SARS-CoV-2/human/AUS/VIC07/2020 ORF1ab	TTCACCGAGGCCACACGGAGTACGATCGAGTGTACAGTGAA
MT450925.1 Severe acute respiratory syndrome coronavirus 2 isolate SARS-CoV-2/human/AUS/VIC08/2020 ORF1ab	TTCACCGAGGCCACGCGGAGTACGATCGAGTGTACAGTGAA
MT450928.1 Severe acute respiratory syndrome coronavirus 2 isolate SARS-CoV-2/human/AUS/VIC11/2020 ORF1ab	TTCACCGAGGCCACGCGGAGTACGATCGAGTGTACAGTGAA
MT450932.1 Severe acute respiratory syndrome coronavirus 2 isolate SARS-CoV-2/human/AUS/VIC17/2020, complet	TTCACCGAGGCCACGCGGAGTACGATCGAGTGTACAGTGAA
MT450933.1 Severe acute respiratory syndrome coronavirus 2 isolate SARS-CoV-2/human/AUS/VIC18/2020 ORF1ab	TTCACCGAGGCCACGCGGAGTACGATCGAGTGTACAGTGAA
MT450934.1 Severe acute respiratory syndrome coronavirus 2 isolate SARS-CoV-2/human/AUS/VIC19/2020 ORF1ab	TTCACCGAGGCCACGCGGAGTACGATCGAGTGTACAGTGAA
MT450935.1 Severe acute respiratory syndrome coronavirus 2 isolate SARS-CoV-2/human/AUS/VIC20/2020, complet	TTCACCGAGGCCACGCGGAGTACGATCGAGTGTACAGTGAA
MT450936.1 Severe acute respiratory syndrome coronavirus 2 isolate SARS-CoV-2/human/AUS/VIC21/2020, complet	TTCACCGAGGCCACGCGGAGTACGATCGAGTGTACAGTGAA
MT450937.1 Severe acute respiratory syndrome coronavirus 2 isolate SARS-CoV-2/human/AUS/VIC22/2020 ORF1ab	TTCACCGAGGCCACGCGGAGTACGATCGAGTGTACAGTGAA
MT450938.1 Severe acute respiratory syndrome coronavirus 2 isolate SARS-CoV-2/human/AUS/VIC23/2020, complet	TTCACCGAGGCCACGCGGAGTACGATCGAGTGTACAGTGAA
MT450939.1 Severe acute respiratory syndrome coronavirus 2 isolate SARS-CoV-2/human/AUS/VIC24/2020 ORF1ab	TTCACCGAGGCCACGCGGAGTACGATCGAGTGTACAGTGAA

MT450940.1 Severe acute respiratory syndrome coronavirus 2 isolate SARS-CoV-2/human/AUS/VIC25/2020 ORF1ab p	TTCACCGAGGCCACGCGGAGTACGATCGAGTGTACAGTGAA
MT450941.1 Severe acute respiratory syndrome coronavirus 2 isolate SARS-CoV-2/human/AUS/VIC26/2020, complet	TTCACCGAGGCCACGCGGAGTACGATCGAGTGTACAGTGAA
MT450942.1 Severe acute respiratory syndrome coronavirus 2 isolate SARS-CoV-2/human/AUS/VIC27/2020, complet	TTCACCGAGGCCACGCGGAGTACGATCGAGTGTACAGTGAA
MT450943.1 Severe acute respiratory syndrome coronavirus 2 isolate SARS-CoV-2/human/AUS/VIC28/2020, complet	TTCACCGAGGCCACGCGGAGTACGATCGAGTGTACAGTGAA
MT450944.1 Severe acute respiratory syndrome coronavirus 2 isolate SARS-CoV-2/human/AUS/VIC29/2020, complet	TTCACCGAGGCCACGCGGAGTACGATCGAGTGTACAGTGAA
MT450946.1 Severe acute respiratory syndrome coronavirus 2 isolate SARS-CoV-2/human/AUS/VIC31/2020, complet	TTCACCGAGGCCACGCGGAGTACGATCGAGTGTACAGTGAA
MT450947.1 Severe acute respiratory syndrome coronavirus 2 isolate SARS-CoV-2/human/AUS/VIC32/2020 ORF1ab p	TTCACCGAGGCCACGCGGAGTACGATCGAGTGTACAGTGAA
MT450948.1 Severe acute respiratory syndrome coronavirus 2 isolate SARS-CoV-2/human/AUS/VIC33/2020, complet	TTCACCGAGGCCACGCGGAGTACGATCGAGTGTACAGTGAA
MT450951.1 Severe acute respiratory syndrome coronavirus 2 isolate SARS-CoV-2/human/AUS/VIC36/2020, complet	TTCACCGAGGCCACGCGGAGTACGATCGAGTGTACAGTGAA
MT450952.1 Severe acute respiratory syndrome coronavirus 2 isolate SARS-CoV-2/human/AUS/VIC37/2020 ORF1ab p	TTCACCGAGGCCACGCGGAGTACGATCGAGTGTACAGTGAA
MT450953.1 Severe acute respiratory syndrome coronavirus 2 isolate SARS-CoV-2/human/AUS/VIC38/2020 ORF1ab p	TTCACCGAGGCCACGCGGAGTACGATCGAGTGTACAGTGAA
MT450954.1 Severe acute respiratory syndrome coronavirus 2 isolate SARS-CoV-2/human/AUS/VIC39/2020, complet	TTCACCGAGGCCACGCGGAGTACGATCGAGTGTACAGTGAA
MT450957.1 Severe acute respiratory syndrome coronavirus 2 isolate SARS-CoV-2/human/AUS/VIC42/2020, complet	TTCACCGAGGCCACGCGGAGTACGATCGAGTGTACAGTGAA
MT450958.1 Severe acute respiratory syndrome coronavirus 2 isolate SARS-CoV-2/human/AUS/VIC43/2020 ORF1ab p	TTCACCGAGGCCACGCGGAGTACGATCGAGTGTACAGTGAA
MT450959.1 Severe acute respiratory syndrome coronavirus 2 isolate SARS-CoV-2/human/AUS/VIC44/2020 ORF1ab p	TTCACCGAGGCCACGCGGAGTACGATCGAGTGTACAGTGAA
MT450960.1 Severe acute respiratory syndrome coronavirus 2 isolate SARS-CoV-2/human/AUS/VIC45/2020, complet	TTCACCGAGGCCACGCGGAGTACGATCGAGTGTACAGTGAA
MT450961.1 Severe acute respiratory syndrome coronavirus 2 isolate SARS-CoV-2/human/AUS/VIC46/2020, complet	TTCACCGAGGCCACGCGGAGTACGATCGAGTGTACAGTGAA
MT450962.1 Severe acute respiratory syndrome coronavirus 2 isolate SARS-CoV-2/human/AUS/VIC47/2020, complet	TTCACCGAGGCCACGCGGAGTACGATCGAGTGTACAGTGAA
MT450963.1 Severe acute respiratory syndrome coronavirus 2 isolate SARS-CoV-2/human/AUS/VIC48/2020, complet	TTCACCGAGGCCACGCGGAGTACGATCGAGTGTACAGTGAA
MT450964.1 Severe acute respiratory syndrome coronavirus 2 isolate SARS-CoV-2/human/AUS/VIC49/2020 ORF1ab p	TTCACCGAGGCCACGCGGAGTACGATCGAGTGTACAGTGAA
MT450965.1 Severe acute respiratory syndrome coronavirus 2 isolate SARS-CoV-2/human/AUS/VIC50/2020, complet	TTCACCGAGGCCACGCGGAGTACGATCGAGTGTACAGTGAA
MT450966.1 Severe acute respiratory syndrome coronavirus 2 isolate SARS-CoV-2/human/AUS/VIC51/2020, complet	TTCACCGAGGCCACGCGGAGTACGATCGAGTGTACAGTGAA
MT450967.1 Severe acute respiratory syndrome coronavirus 2 isolate SARS-CoV-2/human/AUS/VIC52/2020 ORF1ab p	TTCACCGAGGCCACGCGGAGTACGATCGAGTGTACAGTGAA
MT450968.1 Severe acute respiratory syndrome coronavirus 2 isolate SARS-CoV-2/human/AUS/VIC53/2020, complet	TTCACCGAGGCCACGCGGAGTACGATCGAGTGTACAGTGAA
MT450969.1 Severe acute respiratory syndrome coronavirus 2 isolate SARS-CoV-2/human/AUS/VIC54/2020, complet	TTCACCGAGGCCACGCGGAGTACGATCGAGTGTACAGTGAA
MT450970.1 Severe acute respiratory syndrome coronavirus 2 isolate SARS-CoV-2/human/AUS/VIC55/2020, complet	TTCACCGAGGCCACGCGGAGTACGATCGAGTGTACAGTGAA
MT450971.1 Severe acute respiratory syndrome coronavirus 2 isolate SARS-CoV-2/human/AUS/VIC56/2020, complet	TTCACCGAGGCCACGCGGAGTACGATCGAGTGTACAGTGAA
MT450972.1 Severe acute respiratory syndrome coronavirus 2 isolate SARS-CoV-2/human/AUS/VIC57/2020, complet	TTCACCGAGGCCACGCGGAGTACGATCGAGTGTACAGTGAA
MT450974.1 Severe acute respiratory syndrome coronavirus 2 isolate SARS-CoV-2/human/AUS/VIC59/2020, complet	TTCACCGAGGCCACGCGGAGTACGATCGAGTGTACAGTGAA
MT450975.1 Severe acute respiratory syndrome coronavirus 2 isolate SARS-CoV-2/human/AUS/VIC60/2020 ORF1ab p	TTCACCGAGGCCACGCGGAGTACGATCGAGTGTACAGTGAA
MT450976.1 Severe acute respiratory syndrome coronavirus 2 isolate SARS-CoV-2/human/AUS/VIC61/2020 ORF1ab p	TTCACCGAGGCCACGCGGAGTACGATCGAGTGTACAGTGAA
MT450977.1 Severe acute respiratory syndrome coronavirus 2 isolate SARS-CoV-2/human/AUS/VIC62/2020, complet	TTCACCGAGGCCACGCGGAGTACGATCGAGTGTACAGTGAA
MT450978.1 Severe acute respiratory syndrome coronavirus 2 isolate SARS-CoV-2/human/AUS/VIC63/2020 ORF1ab p	TTCACCGAGGCCACGCGGAGTACGATCGAGTGTACAGTGAA

MT450979.1 Severe acute respiratory syndrome coronavirus 2 isolate SARS-CoV-2/human/AUS/VIC64/2020 ORF1ab	TTCACCGAGGCCACGCGGAGTACGATCGAGTGTACAGTGAA
MT450981.1 Severe acute respiratory syndrome coronavirus 2 isolate SARS-CoV-2/human/AUS/VIC67/2020, complet	TTCACCGAGGCCACGCGGAGTACGATCGAGTGTACAGTGAA
MT450982.1 Severe acute respiratory syndrome coronavirus 2 isolate SARS-CoV-2/human/AUS/VIC68/2020, complet	TTCACCGAGGCCACGCGGAGTACGATCGAGTGTACAGTGAA
MT450983.1 Severe acute respiratory syndrome coronavirus 2 isolate SARS-CoV-2/human/AUS/VIC69/2020, complet	TTCACCGAGGCCACGCGGAGTACGATCGAGTGTACAGTGAA
MT450984.1 Severe acute respiratory syndrome coronavirus 2 isolate SARS-CoV-2/human/AUS/VIC70/2020, complet	TTCACCGAGGCCACGCGGAGTACGATCGAGTGTACAGTGAA
MT450985.1 Severe acute respiratory syndrome coronavirus 2 isolate SARS-CoV-2/human/AUS/VIC73/2020, complet	TTCACCGAGGCCACGCGGAGTACGATCGAGTGTACAGTGAA
MT450986.1 Severe acute respiratory syndrome coronavirus 2 isolate SARS-CoV-2/human/AUS/VIC75/2020 ORF1ab	TTCACCGAGGCCACGCGGAGTACGATCGAGTGTACAGTGAA
MT450988.1 Severe acute respiratory syndrome coronavirus 2 isolate SARS-CoV-2/human/AUS/VIC77/2020, complet	TTCACCGAGGCCACGCGGAGTACGATCGAGTGTACAGTGAA
MT450989.1 Severe acute respiratory syndrome coronavirus 2 isolate SARS-CoV-2/human/AUS/VIC79/2020, complet	TTCACCGAGGCCACGCGGAGTACGATCGAGTGTACAGTGAA
MT450990.1 Severe acute respiratory syndrome coronavirus 2 isolate SARS-CoV-2/human/AUS/VIC80/2020, complet	TTCACCGAGGCCACGCGGAGTACGATCGAGTGTACAGTGAA
MT450991.1 Severe acute respiratory syndrome coronavirus 2 isolate SARS-CoV-2/human/AUS/VIC81/2020, complet	TTCACCGAGGCCACGCGGAGTACGATCGAGTGTACAGTGAA
MT450992.1 Severe acute respiratory syndrome coronavirus 2 isolate SARS-CoV-2/human/AUS/VIC82/2020, complet	TTCACCGAGGCCACGCGGAGTACGATCGAGTGTACAGTGAA
MT450994.1 Severe acute respiratory syndrome coronavirus 2 isolate SARS-CoV-2/human/AUS/VIC84/2020, complet	TTCACCGAGGCCACGCGGAGTACGATCGAGTGTACAGTGAA
MT450995.1 Severe acute respiratory syndrome coronavirus 2 isolate SARS-CoV-2/human/AUS/VIC85/2020, complet	TTCACCGAGGCCACGCGGAGTACGATCGAGTGTACAGTGAA
MT450996.1 Severe acute respiratory syndrome coronavirus 2 isolate SARS-CoV-2/human/AUS/VIC86/2020, complet	TTCACCGAGGCCACGCGGAGTACGATCGAGTGTACAGTGAA
MT450997.1 Severe acute respiratory syndrome coronavirus 2 isolate SARS-CoV-2/human/AUS/VIC87/2020, complet	TTCACCGAGGCCACGCGGAGTACGATCGAGTGTACAGTGAA
MT451000.1 Severe acute respiratory syndrome coronavirus 2 isolate SARS-CoV-2/human/AUS/VIC90/2020, complet	TTCACCGAGGCCACGCGGAGTACGATCGAGTGTACAGTGAA
MT451001.1 Severe acute respiratory syndrome coronavirus 2 isolate SARS-CoV-2/human/AUS/VIC91/2020 ORF1ab	TTCACCGAGGCCACGCGGAGTACGATCGAGTGTACAGTGAA
MT451002.1 Severe acute respiratory syndrome coronavirus 2 isolate SARS-CoV-2/human/AUS/VIC92/2020, complet	TTCACCGAGGCCACGCGGAGTACGATCGAGTGTACAGTGAA
MT451003.1 Severe acute respiratory syndrome coronavirus 2 isolate SARS-CoV-2/human/AUS/VIC93/2020 ORF1ab	TTCACCGAGGCCACGCGGAGTACGATCGAGTGTACAGTGAA
MT451004.1 Severe acute respiratory syndrome coronavirus 2 isolate SARS-CoV-2/human/AUS/VIC94/2020, complet	TTCACCGAGGCCACGCGGAGTACGATCGAGTGTACAGTGAA
MT451005.1 Severe acute respiratory syndrome coronavirus 2 isolate SARS-CoV-2/human/AUS/VIC95/2020, complet	TTCACCGAGGCCACGCGGAGTACGATCGAGTGTACAGTGAA
MT451006.1 Severe acute respiratory syndrome coronavirus 2 isolate SARS-CoV-2/human/AUS/VIC96/2020, complet	TTCACCGAGGCCACGCGGAGTACGATCGAGTGTACAGTGAA
MT451008.1 Severe acute respiratory syndrome coronavirus 2 isolate SARS-CoV-2/human/AUS/VIC98/2020, complet	TTCACCGAGGCCACGCGGAGTACGATCGAGTGTACAGTGAA
MT451009.1 Severe acute respiratory syndrome coronavirus 2 isolate SARS-CoV-2/human/AUS/VIC99/2020, complet	TTCACCGAGGCCACGCGGAGTACGATCGAGTGTACAGTGAA
MT451010.1 Severe acute respiratory syndrome coronavirus 2 isolate SARS-CoV-2/human/AUS/VIC100/2020 ORF1ab	TTCACCGAGGCCACGCGGAGTACGATCGAGTGTACAGTGAA
MT451012.1 Severe acute respiratory syndrome coronavirus 2 isolate SARS-CoV-2/human/AUS/VIC102/2020 ORF1ab	TTCACCGAGGCCACGCGGAGTACGATCGAGTGTACAGTGAA
MT451013.1 Severe acute respiratory syndrome coronavirus 2 isolate SARS-CoV-2/human/AUS/VIC103/2020, comple	TTCACCGAGGCCACGCGGAGTACGATCGAGTGTACAGTGAA
MT451015.1 Severe acute respiratory syndrome coronavirus 2 isolate SARS-CoV-2/human/AUS/VIC105/2020, comple	TTCACCGAGGCCACGCGGAGTACGATCGAGTGTACAGTGAA
MT451016.1 Severe acute respiratory syndrome coronavirus 2 isolate SARS-CoV-2/human/AUS/VIC107/2020 ORF1ab	TTCACCGAGGCCACGCGGAGTACGATCGAGTGTACAGTGAA
MT451017.1 Severe acute respiratory syndrome coronavirus 2 isolate SARS-CoV-2/human/AUS/VIC108/2020, comple	TTCACCGAGGCCACGCGGAGTACGATCGAGTGTACAGTGAA
MT451018.1 Severe acute respiratory syndrome coronavirus 2 isolate SARS-CoV-2/human/AUS/VIC109/2020, comple	TTCACCGAGGCCACGCGGAGTACGATCGAGTGTACAGTGAA
MT451019.1 Severe acute respiratory syndrome coronavirus 2 isolate SARS-CoV-2/human/AUS/VIC110/2020, comple	TTCACCGAGGCCACGCGGAGTACGATCGAGTGTACAGTGAA

MT451021.1 Severe acute respiratory syndrome coronavirus 2 isolate SARS-CoV-2/human/AUS/VIC112/2020, comple	TTCACCGAGGCCACGCGGAGTACGATCGAGTGTACAGTGAA
MT451022.1 Severe acute respiratory syndrome coronavirus 2 isolate SARS-CoV-2/human/AUS/VIC113/2020, comple	TTCACCGAGGCCACGCGGAGTACGATCGAGTGTACAGTGAA
MT451023.1 Severe acute respiratory syndrome coronavirus 2 isolate SARS-CoV-2/human/AUS/VIC114/2020 ORF1ab	TTCACCGAGGCCACGCGGAGTACGATCGAGTGTACAGTGAA
MT451024.1 Severe acute respiratory syndrome coronavirus 2 isolate SARS-CoV-2/human/AUS/VIC115/2020, comple	TTCACCGAGGCCACGCGGAGTACGATCGAGTGTACAGTGAA
MT451025.1 Severe acute respiratory syndrome coronavirus 2 isolate SARS-CoV-2/human/AUS/VIC116/2020 ORF1ab	TTCACCGAGGCCACGCGGAGTACGATCGAGTGTACAGTGAA
MT451026.1 Severe acute respiratory syndrome coronavirus 2 isolate SARS-CoV-2/human/AUS/VIC117/2020, comple	TTCACCGAGGCCACGCGGAGTACGATCGAGTGTACAGTGAA
MT451027.1 Severe acute respiratory syndrome coronavirus 2 isolate SARS-CoV-2/human/AUS/VIC118/2020 ORF1ab	TTCACCGAGGCCACGCGGAGTACGATCGAGTGTACAGTGAA
MT451028.1 Severe acute respiratory syndrome coronavirus 2 isolate SARS-CoV-2/human/AUS/VIC119/2020 ORF1ab	TTCACCGAGGCCACGCGGAGTACGATCGAGTGTACAGTGAA
MT451029.1 Severe acute respiratory syndrome coronavirus 2 isolate SARS-CoV-2/human/AUS/VIC120/2020 ORF1ab	TTCACCGAGGCCACGCGGAGTACGATCGAGTGTACAGTGAA
MT451030.1 Severe acute respiratory syndrome coronavirus 2 isolate SARS-CoV-2/human/AUS/VIC121/2020 ORF1ab	TTCACCGAGGCCACGCGGAGTACGATCGAGTGTACAGTGAA
MT451031.1 Severe acute respiratory syndrome coronavirus 2 isolate SARS-CoV-2/human/AUS/VIC122/2020 ORF1ab	TTCACCGAGGCCACGCGGAGTACGATCGAGTGTACAGTGAA
MT451032.1 Severe acute respiratory syndrome coronavirus 2 isolate SARS-CoV-2/human/AUS/VIC123/2020 ORF1ab	TTCACCGAGGCCACGCGGAGTACGATCGAGTGTACAGTGAA
MT451033.1 Severe acute respiratory syndrome coronavirus 2 isolate SARS-CoV-2/human/AUS/VIC126/2020, comple	TTCACCGAGGCCACGCGGAGTACGATCGAGTGTACAGTGAA
MT451034.1 Severe acute respiratory syndrome coronavirus 2 isolate SARS-CoV-2/human/AUS/VIC127/2020 ORF1ab	TTCACCGAGGCCACGCGGAGTACGATCGAGTGTACAGTGAA
MT451035.1 Severe acute respiratory syndrome coronavirus 2 isolate SARS-CoV-2/human/AUS/VIC128/2020, comple	TTCACCGAGGCCACGCGGAGTACGATCGAGTGTACAGTGAA
MT451036.1 Severe acute respiratory syndrome coronavirus 2 isolate SARS-CoV-2/human/AUS/VIC130/2020, comple	TTCACCGAGGCCACGCGGAGTACGATCGAGTGTACAGTGAA
MT451037.1 Severe acute respiratory syndrome coronavirus 2 isolate SARS-CoV-2/human/AUS/VIC131/2020, comple	TTCACCGAGGCCACGCGGAGTACGATCGAGTGTACAGTGAA
MT451038.1 Severe acute respiratory syndrome coronavirus 2 isolate SARS-CoV-2/human/AUS/VIC134/2020, comple	TTCACCGAGGCCACGCGGAGTACGATCGAGTGTACAGTGAA
MT451039.1 Severe acute respiratory syndrome coronavirus 2 isolate SARS-CoV-2/human/AUS/VIC135/2020, comple	TTCACCGAGGCCACGCGGAGTACGATCGAGTGTACAGTGAA
MT451040.1 Severe acute respiratory syndrome coronavirus 2 isolate SARS-CoV-2/human/AUS/VIC136/2020, comple	TTCACCGAGGCCACGCGGAGTACGATCGAGTGTACAGTGAA
MT451041.1 Severe acute respiratory syndrome coronavirus 2 isolate SARS-CoV-2/human/AUS/VIC137/2020, comple	TTCACCGAGGCCACGCGGAGTACGATCGAGTGTACAGTGAA
MT451042.1 Severe acute respiratory syndrome coronavirus 2 isolate SARS-CoV-2/human/AUS/VIC138/2020, comple	TTCACCGAGGCCACGCGGAGTACGATCGAGTGTACAGTGAA
MT451043.1 Severe acute respiratory syndrome coronavirus 2 isolate SARS-CoV-2/human/AUS/VIC139/2020 ORF1ab	TTCACCGAGGCCACGCGGAGTACGATCGAGTGTACAGTGAA
MT451045.1 Severe acute respiratory syndrome coronavirus 2 isolate SARS-CoV-2/human/AUS/VIC141/2020, comple	TTCACCGAGGCCACGCGGAGTACGATCGAGTGTACAGTGAA
MT451046.1 Severe acute respiratory syndrome coronavirus 2 isolate SARS-CoV-2/human/AUS/VIC142/2020, comple	TTCACCGAGGCCACGCGGAGTACGATCGAGTGTACAGTGAA
MT451047.1 Severe acute respiratory syndrome coronavirus 2 isolate SARS-CoV-2/human/AUS/VIC143/2020, comple	TTCACCGAGGCCACGCGGAGTACGATCGAGTGTACAGTGAA
MT451048.1 Severe acute respiratory syndrome coronavirus 2 isolate SARS-CoV-2/human/AUS/VIC144/2020, comple	TTCACCGAGGCCACGCGGAGTACGATCGAGTGTACAGTGAA
MT451049.1 Severe acute respiratory syndrome coronavirus 2 isolate SARS-CoV-2/human/AUS/VIC145/2020, comple	TTCACCGAGGCCACGCGGAGTACGATCGAGTGTACAGTGAA
MT451050.1 Severe acute respiratory syndrome coronavirus 2 isolate SARS-CoV-2/human/AUS/VIC146/2020 ORF1ab	TTCACCGAGGCCACGCGGAGTACGATCGAGTGTACAGTGAA
MT451051.1 Severe acute respiratory syndrome coronavirus 2 isolate SARS-CoV-2/human/AUS/VIC147/2020, comple	TTCACCGAGGCCACGCGGAGTACGATCGAGTGTACAGTGAA
MT451052.1 Severe acute respiratory syndrome coronavirus 2 isolate SARS-CoV-2/human/AUS/VIC148/2020, comple	TTCACCGAGGCCACGCGGAGTACGATCGAGTGTACAGTGAA
MT451053.1 Severe acute respiratory syndrome coronavirus 2 isolate SARS-CoV-2/human/AUS/VIC149/2020 ORF1ab	TTCACCGAGGCCACGCGGAGTACGATCGAGTGTACAGTGAA
MT451054.1 Severe acute respiratory syndrome coronavirus 2 isolate SARS-CoV-2/human/AUS/VIC150/2020 ORF1ab	TTCACCGAGGCCACGCGGAGTACGATCGAGTGTACAGTGAA

MT451056.1 Severe acute respiratory syndrome coronavirus 2 isolate SARS-CoV-2/human/AUS/VIC152/2020, comple	TTCACCGAGGCCACGCGGAGTACGATCGAGTGTACAGTGAA
MT451057.1 Severe acute respiratory syndrome coronavirus 2 isolate SARS-CoV-2/human/AUS/VIC153/2020, comple	TTCACCGAGGCCACGCGGAGTACGATCGAGTGTACAGTGAA
MT451058.1 Severe acute respiratory syndrome coronavirus 2 isolate SARS-CoV-2/human/AUS/VIC154/2020, comple	TTCACCGAGGCCACGCGGAGTACGATCGAGTGTACAGTGAA
MT451059.1 Severe acute respiratory syndrome coronavirus 2 isolate SARS-CoV-2/human/AUS/VIC155/2020, comple	TTCACCGAGGCCACGCGGAGTACGATCGAGTGTACAGTGAA
MT451060.1 Severe acute respiratory syndrome coronavirus 2 isolate SARS-CoV-2/human/AUS/VIC156/2020, comple	TTCACCGAGGCCACGCGGAGTACGATCGAGTGTACAGTGAA
MT451062.1 Severe acute respiratory syndrome coronavirus 2 isolate SARS-CoV-2/human/AUS/VIC158/2020, comple	TTCACCGAGGCCACGCGGAGTACGATCGAGTGTACAGTGAA
MT451063.1 Severe acute respiratory syndrome coronavirus 2 isolate SARS-CoV-2/human/AUS/VIC159/2020 ORF1ab	TTCACCGAGGCCACGCGGAGTACGATCGAGTGTACAGTGAA
MT451064.1 Severe acute respiratory syndrome coronavirus 2 isolate SARS-CoV-2/human/AUS/VIC160/2020 ORF1ab	TTCACCGAGGCCACGCGGAGTACGATCGAGTGTACAGTGAA
MT451065.1 Severe acute respiratory syndrome coronavirus 2 isolate SARS-CoV-2/human/AUS/VIC162/2020, comple	TTCACCGAGGCCACGCGGAGTACGATCGAGTGTACAGTGAA
MT451066.1 Severe acute respiratory syndrome coronavirus 2 isolate SARS-CoV-2/human/AUS/VIC163/2020, comple	TTCACCGAGGCCACGCGGAGTACGATCGAGTGTACAGTGAA
MT451068.1 Severe acute respiratory syndrome coronavirus 2 isolate SARS-CoV-2/human/AUS/VIC165/2020 ORF1ab	TTCACCGAGGCCACGCGGAGTACGATCGAGTGTACAGTGAA
MT451069.1 Severe acute respiratory syndrome coronavirus 2 isolate SARS-CoV-2/human/AUS/VIC166/2020 ORF1ab	TTCACCGAGGCCACGCGGAGTACGATCGAGTGTACAGTGAA
MT451070.1 Severe acute respiratory syndrome coronavirus 2 isolate SARS-CoV-2/human/AUS/VIC167/2020 ORF1ab	TTCACCGAGGCCACGCGGAGTACGATCGAGTGTACAGTGAA
MT451071.1 Severe acute respiratory syndrome coronavirus 2 isolate SARS-CoV-2/human/AUS/VIC168/2020 ORF1ab	TTCACCGAGGCCACGCGGAGTACGATCGAGTGTACAGTGAA
MT451072.1 Severe acute respiratory syndrome coronavirus 2 isolate SARS-CoV-2/human/AUS/VIC169/2020, comple	TTCACCGAGGCCACGCGGAGTACGATCGAGTGTACAGTGAA
MT451073.1 Severe acute respiratory syndrome coronavirus 2 isolate SARS-CoV-2/human/AUS/VIC170/2020 ORF1ab	TTCACCGAGGCCACGCGGAGTACGATCGAGTGTACAGTGAA
MT451074.1 Severe acute respiratory syndrome coronavirus 2 isolate SARS-CoV-2/human/AUS/VIC171/2020, comple	TTCACCGAGGCCACGCGGAGTACGATCGAGTGTACAGTGAA
MT451075.1 Severe acute respiratory syndrome coronavirus 2 isolate SARS-CoV-2/human/AUS/VIC172/2020 ORF1ab	TTCACCGAGGCCACGCGGAGTACGATCGAGTGTACAGTGAA
MT451076.1 Severe acute respiratory syndrome coronavirus 2 isolate SARS-CoV-2/human/AUS/VIC173/2020, comple	TTCACCGAGGCCACGCGGAGTACGATCGAGTGTACAGTGAA
MT451077.1 Severe acute respiratory syndrome coronavirus 2 isolate SARS-CoV-2/human/AUS/VIC174/2020, comple	TTCACCGAGGCCACGCGGAGTACGATCGAGTGTACAGTGAA
MT451078.1 Severe acute respiratory syndrome coronavirus 2 isolate SARS-CoV-2/human/AUS/VIC175/2020 ORF1ab	TTCACCGAGGCCACGCGGAGTACGATCGAGTGTACAGTGAA
MT451079.1 Severe acute respiratory syndrome coronavirus 2 isolate SARS-CoV-2/human/AUS/VIC176/2020, comple	TTCACCGAGGCCACGCGGAGTACGATCGAGTGTACAGTGAA
MT451080.1 Severe acute respiratory syndrome coronavirus 2 isolate SARS-CoV-2/human/AUS/VIC177/2020 ORF1ab	TTCACCGAGGCCACGCGGAGTACGATCGAGTGTACAGTGAA
MT451081.1 Severe acute respiratory syndrome coronavirus 2 isolate SARS-CoV-2/human/AUS/VIC178/2020, comple	TTCACCGAGGCCACGCGGAGTACGATCGAGTGTACAGTGAA
MT451082.1 Severe acute respiratory syndrome coronavirus 2 isolate SARS-CoV-2/human/AUS/VIC179/2020 ORF1ab	TTCACCGAGGCCACGCGGAGTACGATCGAGTGTACAGTGAA
MT451083.1 Severe acute respiratory syndrome coronavirus 2 isolate SARS-CoV-2/human/AUS/VIC181/2020 ORF1ab	TTCACCGAGGCCACGCGGAGTACGATCGAGTGTACAGTGAA
MT451084.1 Severe acute respiratory syndrome coronavirus 2 isolate SARS-CoV-2/human/AUS/VIC182/2020 ORF1ab	TTCACCGAGGCCACGCGGAGTACGATCGAGTGTACAGTGAA
MT451085.1 Severe acute respiratory syndrome coronavirus 2 isolate SARS-CoV-2/human/AUS/VIC183/2020 ORF1ab	TTCACCGAGGCCACGCGGAGTACGATCGAGTGTACAGTGAA
MT451086.1 Severe acute respiratory syndrome coronavirus 2 isolate SARS-CoV-2/human/AUS/VIC184/2020, comple	TTCACCGAGGCCACGCGGAGTACGATCGAGTGTACAGTGAA
MT451087.1 Severe acute respiratory syndrome coronavirus 2 isolate SARS-CoV-2/human/AUS/VIC185/2020, comple	TTCACCGAGGCCACGCGGAGTACGATCGAGTGTACAGTGAA
MT451088.1 Severe acute respiratory syndrome coronavirus 2 isolate SARS-CoV-2/human/AUS/VIC186/2020, comple	TTCACCGAGGCCACGCGGAGTACGATCGAGTGTACAGTGAA
MT451089.1 Severe acute respiratory syndrome coronavirus 2 isolate SARS-CoV-2/human/AUS/VIC187/2020 ORF1ab	TTCACCGAGGCCACGCGGAGTACGATCGAGTGTACAGTGAA
MT451090.1 Severe acute respiratory syndrome coronavirus 2 isolate SARS-CoV-2/human/AUS/VIC188/2020, comple	TTCACCGAGGCCACGCGGAGTACGATCGAGTGTACAGTGAA

MT451091.1 Severe acute respiratory syndrome coronavirus 2 isolate SARS-CoV-2/human/AUS/VIC189/2020 ORF1ab	TTCACCGAGGCCACGCGGAGTACGATCGAGTGTACAGTGAA
MT451092.1 Severe acute respiratory syndrome coronavirus 2 isolate SARS-CoV-2/human/AUS/VIC190/2020, comple	TTCACCGAGGCCACGCGGAGTACGATCGAGTGTACAGTGAA
MT451093.1 Severe acute respiratory syndrome coronavirus 2 isolate SARS-CoV-2/human/AUS/VIC191/2020, comple	TTCACCGAGGCCACGCGGAGTACGATCGAGTGTACAGTGAA
MT451094.1 Severe acute respiratory syndrome coronavirus 2 isolate SARS-CoV-2/human/AUS/VIC192/2020, comple	TTCACCGAGGCCACGCGGAGTACGATCGAGTGTACAGTGAA
MT451096.1 Severe acute respiratory syndrome coronavirus 2 isolate SARS-CoV-2/human/AUS/VIC194/2020 ORF1ab	TTCACCGAGGCCACGCGGAGTACGATCGAGTGTACAGTGAA
MT451098.1 Severe acute respiratory syndrome coronavirus 2 isolate SARS-CoV-2/human/AUS/VIC196/2020, comple	TTCACCGAGGCCACGCGGAGTACGATCGAGTGTACAGTGAA
MT451099.1 Severe acute respiratory syndrome coronavirus 2 isolate SARS-CoV-2/human/AUS/VIC197/2020 ORF1ab	TTCACCGAGGCCACGCGGAGTACGATCGAGTGTACAGTGAA
MT451100.1 Severe acute respiratory syndrome coronavirus 2 isolate SARS-CoV-2/human/AUS/VIC198/2020, comple	TTCACCGAGGCCACGCGGAGTACGATCGAGTGTACAGTGAA
MT451101.1 Severe acute respiratory syndrome coronavirus 2 isolate SARS-CoV-2/human/AUS/VIC199/2020, comple	TTCACCGAGGCCACGCGGAGTACGATCGAGTGTACAGTGAA
MT451102.1 Severe acute respiratory syndrome coronavirus 2 isolate SARS-CoV-2/human/AUS/VIC201/2020, comple	TTCACCGAGGCCACGCGGAGTACGATCGAGTGTACAGTGAA
MT451103.1 Severe acute respiratory syndrome coronavirus 2 isolate SARS-CoV-2/human/AUS/VIC202/2020, comple	TTCACCGAGGCCACGCGGAGTACGATCGAGTGTACAGTGAA
MT451104.1 Severe acute respiratory syndrome coronavirus 2 isolate SARS-CoV-2/human/AUS/VIC203/2020, comple	TTCACCGAGGCCACGCGGAGTACGATCGAGTGTACAGTGAA
MT451105.1 Severe acute respiratory syndrome coronavirus 2 isolate SARS-CoV-2/human/AUS/VIC204/2020, comple	TTCACCGAGGCCACGCGGAGTACGATCGAGTGTACAGTGAA
MT451106.1 Severe acute respiratory syndrome coronavirus 2 isolate SARS-CoV-2/human/AUS/VIC205/2020 ORF1ab	TTCACCGAGGCCACGCGGAGTACGATCGAGTGTACAGTGAA
MT451108.1 Severe acute respiratory syndrome coronavirus 2 isolate SARS-CoV-2/human/AUS/VIC207/2020, comple	TTCACCGAGGCCACGCGGAGTACGATCGAGTGTACAGTGAA
MT451109.1 Severe acute respiratory syndrome coronavirus 2 isolate SARS-CoV-2/human/AUS/VIC208/2020, comple	TTCACCGAGGCCACGCGGAGTACGATCGAGTGTACAGTGAA
MT451110.1 Severe acute respiratory syndrome coronavirus 2 isolate SARS-CoV-2/human/AUS/VIC209/2020 ORF1ab	TTCACCGAGGCCACGCGGAGTACGATCGAGTGTACAGTGAA
MT451111.1 Severe acute respiratory syndrome coronavirus 2 isolate SARS-CoV-2/human/AUS/VIC210/2020 ORF1ab	TTCACCGAGGCCACGCGGAGTACGATCGAGTGTACAGTGAA
MT451112.1 Severe acute respiratory syndrome coronavirus 2 isolate SARS-CoV-2/human/AUS/VIC211/2020 ORF1ab	TTCACCGAGGCCACGCGGAGTACGATCGAGTGTACAGTGAA
MT451113.1 Severe acute respiratory syndrome coronavirus 2 isolate SARS-CoV-2/human/AUS/VIC212/2020 ORF1ab	TTCACCGAGGCCACGCGGAGTACGATCGAGTGTACAGTGAA
MT451114.1 Severe acute respiratory syndrome coronavirus 2 isolate SARS-CoV-2/human/AUS/VIC213/2020, comple	TTCACCGAGGCCACGCGGAGTACGATCGAGTGTACAGTGAA
MT451115.1 Severe acute respiratory syndrome coronavirus 2 isolate SARS-CoV-2/human/AUS/VIC214/2020, comple	TTCACCGAGGCCACGCGGAGTACGATCGAGTGTACAGTGAA
MT451116.1 Severe acute respiratory syndrome coronavirus 2 isolate SARS-CoV-2/human/AUS/VIC215/2020, comple	TTCACCGAGGCCACGCGGAGTACGATCGAGTGTACAGTGAA
MT451117.1 Severe acute respiratory syndrome coronavirus 2 isolate SARS-CoV-2/human/AUS/VIC216/2020, comple	TTCACCGAGGCCACGCGGAGTACGATCGAGTGTACAGTGAA
MT451118.1 Severe acute respiratory syndrome coronavirus 2 isolate SARS-CoV-2/human/AUS/VIC217/2020, comple	TTCACCGAGGCCACGCGGAGTACGATCGAGTGTACAGTGAA
MT451119.1 Severe acute respiratory syndrome coronavirus 2 isolate SARS-CoV-2/human/AUS/VIC218/2020, comple	TTCACCGAGGCCACGCGGAGTACGATCGAGTGTACAGTGAA
MT451120.1 Severe acute respiratory syndrome coronavirus 2 isolate SARS-CoV-2/human/AUS/VIC219/2020 ORF1ab	TTCACCGAGGCCACGCGGAGTACGATCGAGTGTACAGTGAA
MT451121.1 Severe acute respiratory syndrome coronavirus 2 isolate SARS-CoV-2/human/AUS/VIC220/2020, comple	TTCACCGAGGCCACGCGGAGTACGATCGAGTGTACAGTGAA
MT451122.1 Severe acute respiratory syndrome coronavirus 2 isolate SARS-CoV-2/human/AUS/VIC222/2020, comple	TTCACCGAGGCCACGCGGAGTACGATCGAGTGTACAGTGAA
MT451123.1 Severe acute respiratory syndrome coronavirus 2 isolate SARS-CoV-2/human/AUS/VIC223/2020, comple	TTCACCGAGGCCACGCGGAGTACGATCGAGTGTACAGTGAA
MT451124.1 Severe acute respiratory syndrome coronavirus 2 isolate SARS-CoV-2/human/AUS/VIC224/2020, comple	TTCACCGAGGCCACGCGGAGTACGATCGAGTGTACAGTGAA
MT451125.1 Severe acute respiratory syndrome coronavirus 2 isolate SARS-CoV-2/human/AUS/VIC225/2020, comple	TTCACCGAGGCCACGCGGAGTACGATCGAGTGTACAGTGAA
MT451126.1 Severe acute respiratory syndrome coronavirus 2 isolate SARS-CoV-2/human/AUS/VIC226/2020, comple	TTCACCGAGGCCACGCGGAGTACGATCGAGTGTACAGTGAA

MT451127.1 Severe acute respiratory syndrome coronavirus 2 isolate SARS-CoV-2/human/AUS/VIC227/2020, comple	TTCACCGAGGCCACGCGGAGTACGATCGAGTGTACAGTGAA
MT451128.1 Severe acute respiratory syndrome coronavirus 2 isolate SARS-CoV-2/human/AUS/VIC228/2020, comple	TTCACCGAGGCCACGCGGAGTACGATCGAGTGTACAGTGAA
MT451129.1 Severe acute respiratory syndrome coronavirus 2 isolate SARS-CoV-2/human/AUS/VIC229/2020, comple	TTCACCGAGGCCACGCGGAGTACGATCGAGTGTACAGTGAA
MT451130.1 Severe acute respiratory syndrome coronavirus 2 isolate SARS-CoV-2/human/AUS/VIC231/2020, comple	TTCACCGAGGCCACGCGGAGTACGATCGAGTGTACAGTGAA
MT451131.1 Severe acute respiratory syndrome coronavirus 2 isolate SARS-CoV-2/human/AUS/VIC232/2020, comple	TTCACCGAGGCCACGCGGAGTACGATCGAGTGTACAGTGAA
MT451132.1 Severe acute respiratory syndrome coronavirus 2 isolate SARS-CoV-2/human/AUS/VIC233/2020, comple	TTCACCGAGGCCACGCGGAGTACGATCGAGTGTACAGTGAA
MT451133.1 Severe acute respiratory syndrome coronavirus 2 isolate SARS-CoV-2/human/AUS/VIC234/2020, comple	TTCACCGAGGCCACGCGGAGTACGATCGAGTGTACAGTGAA
MT451134.1 Severe acute respiratory syndrome coronavirus 2 isolate SARS-CoV-2/human/AUS/VIC236/2020, comple	TTCACCGAGGCCACGCGGAGTACGATCGAGTGTACAGTGAA
MT451135.1 Severe acute respiratory syndrome coronavirus 2 isolate SARS-CoV-2/human/AUS/VIC237/2020, comple	TTCACCGAGGCCACGCGGAGTACGATCGAGTGTACAGTGAA
MT451136.1 Severe acute respiratory syndrome coronavirus 2 isolate SARS-CoV-2/human/AUS/VIC238/2020, comple	TTCACCGAGGCCACGCGGAGTACGATCGAGTGTACAGTGAA
MT451137.1 Severe acute respiratory syndrome coronavirus 2 isolate SARS-CoV-2/human/AUS/VIC239/2020, comple	TTCACCGAGGCCACGCGGAGTACGATCGAGTGTACAGTGAA
MT451138.1 Severe acute respiratory syndrome coronavirus 2 isolate SARS-CoV-2/human/AUS/VIC240/2020, comple	TTCACCGAGGCCACGCGGAGTACGATCGAGTGTACAGTGAA
MT451139.1 Severe acute respiratory syndrome coronavirus 2 isolate SARS-CoV-2/human/AUS/VIC241/2020, comple	TTCACCGAGGCCACGCGGAGTACGATCGAGTGTACAGTGAA
MT451140.1 Severe acute respiratory syndrome coronavirus 2 isolate SARS-CoV-2/human/AUS/VIC243/2020, comple	TTCACCGAGGCCACGCGGAGTACGATCGAGTGTACAGTGAA
MT451141.1 Severe acute respiratory syndrome coronavirus 2 isolate SARS-CoV-2/human/AUS/VIC244/2020, comple	TTCACCGAGGCCACGCGGAGTACGATCGAGTGTACAGTGAA
MT451142.1 Severe acute respiratory syndrome coronavirus 2 isolate SARS-CoV-2/human/AUS/VIC245/2020, comple	TTCACCGAGGCCACGCGGAGTACGATCGAGTGTACAGTGAA
MT451143.1 Severe acute respiratory syndrome coronavirus 2 isolate SARS-CoV-2/human/AUS/VIC246/2020, comple	TTCACCGAGGCCACGCGGAGTACGATCGAGTGTACAGTGAA
MT451144.1 Severe acute respiratory syndrome coronavirus 2 isolate SARS-CoV-2/human/AUS/VIC247/2020, comple	TTCACCGAGGCCACGCGGAGTACGATCGAGTGTACAGTGAA
MT451145.1 Severe acute respiratory syndrome coronavirus 2 isolate SARS-CoV-2/human/AUS/VIC248/2020, comple	TTCACCGAGGCCACGCGGAGTACGATCGAGTGTACAGTGAA
MT451146.1 Severe acute respiratory syndrome coronavirus 2 isolate SARS-CoV-2/human/AUS/VIC249/2020, comple	TTCACCGAGGCCACGCGGAGTACGATCGAGTGTACAGTGAA
MT451148.1 Severe acute respiratory syndrome coronavirus 2 isolate SARS-CoV-2/human/AUS/VIC251/2020, comple	TTCACCGAGGCCACGCGGAGTACGATCGAGTGTACAGTGAA
MT451149.1 Severe acute respiratory syndrome coronavirus 2 isolate SARS-CoV-2/human/AUS/VIC252/2020, comple	TTCACCGAGGCCACGCGGAGTACGATCGAGTGTACAGTGAA
MT451150.1 Severe acute respiratory syndrome coronavirus 2 isolate SARS-CoV-2/human/AUS/VIC253/2020, comple	TTCACCGAGGCCACGCGGAGTACGATCGAGTGTACAGTGAA
MT451151.1 Severe acute respiratory syndrome coronavirus 2 isolate SARS-CoV-2/human/AUS/VIC254/2020, comple	TTCACCGAGGCCACGCGGAGTACGATCGAGTGTACAGTGAA
MT451152.1 Severe acute respiratory syndrome coronavirus 2 isolate SARS-CoV-2/human/AUS/VIC256/2020, comple	TTCACCGAGGCCACGCGGAGTACGATCGAGTGTACAGTGAA
MT451153.1 Severe acute respiratory syndrome coronavirus 2 isolate SARS-CoV-2/human/AUS/VIC257/2020, comple	TTCACCGAGGCCACGCGGAGTACGATCGAGTGTACAGTGAA
MT451154.1 Severe acute respiratory syndrome coronavirus 2 isolate SARS-CoV-2/human/AUS/VIC258/2020, comple	TTCACCGAGGCCACGCGGAGTACGATCGAGTGTACAGTGAA
MT451155.1 Severe acute respiratory syndrome coronavirus 2 isolate SARS-CoV-2/human/AUS/VIC259/2020, comple	TTCACCGAGGCCACGCGGAGTACGATCGAGTGTACAGTGAA
MT451156.1 Severe acute respiratory syndrome coronavirus 2 isolate SARS-CoV-2/human/AUS/VIC260/2020, comple	TTCACCGAGGCCACGCGGAGTACGATCGAGTGTACAGTGAA
MT451157.1 Severe acute respiratory syndrome coronavirus 2 isolate SARS-CoV-2/human/AUS/VIC261/2020, comple	TTCACCGAGGCCACGCGGAGTACGATCGAGTGTACAGTGAA
MT451159.1 Severe acute respiratory syndrome coronavirus 2 isolate SARS-CoV-2/human/AUS/VIC263/2020, comple	TTCACCGAGGCCACGCGGAGTACGATCGAGTGTACAGTGAA
MT451160.1 Severe acute respiratory syndrome coronavirus 2 isolate SARS-CoV-2/human/AUS/VIC264/2020, comple	TTCACCGAGGCCACGCGGAGTACGATCGAGTGTACAGTGAA
MT451162.1 Severe acute respiratory syndrome coronavirus 2 isolate SARS-CoV-2/human/AUS/VIC266/2020, comple	TTCACCGAGGCCACGCGGAGTACGATCGAGTGTACAGTGAA

MT451163.1 Severe acute respiratory syndrome coronavirus 2 isolate SARS-CoV-2/human/AUS/VIC267/2020, comple	TTCACCGAGGCCACGCGGAGTACGATCGAGTGTACAGTGAA
MT451164.1 Severe acute respiratory syndrome coronavirus 2 isolate SARS-CoV-2/human/AUS/VIC268/2020, comple	TTCACCGAGGCCACGCGGAGTACGATCGAGTGTACAGTGAA
MT451165.1 Severe acute respiratory syndrome coronavirus 2 isolate SARS-CoV-2/human/AUS/VIC269/2020, comple	TTCACCGAGGCCACGCGGAGTACGATCGAGTGTACAGTGAA
MT451169.1 Severe acute respiratory syndrome coronavirus 2 isolate SARS-CoV-2/human/AUS/VIC273/2020, comple	TTCACCGAGGCCACGCGGAGTACGATCGAGTGTACAGTGAA
MT451170.1 Severe acute respiratory syndrome coronavirus 2 isolate SARS-CoV-2/human/AUS/VIC274/2020, comple	TTCACCGAGGCCACGCGGAGTACGATCGAGTGTACAGTGAA
MT451171.1 Severe acute respiratory syndrome coronavirus 2 isolate SARS-CoV-2/human/AUS/VIC275/2020, comple	TTCACCGAGGCCACGCGGAGTACGATCGAGTGTACAGTGAA
MT451172.1 Severe acute respiratory syndrome coronavirus 2 isolate SARS-CoV-2/human/AUS/VIC276/2020, comple	TTCACCGAGGCCACGCGGAGTACGATCGAGTGTACAGTGAA
MT451173.1 Severe acute respiratory syndrome coronavirus 2 isolate SARS-CoV-2/human/AUS/VIC277/2020, comple	TTCACCGAGGCCACGCGGAGTACGATCGAGTGTACAGTGAA
MT451174.1 Severe acute respiratory syndrome coronavirus 2 isolate SARS-CoV-2/human/AUS/VIC278/2020, comple	TTCACCGAGGCCACGCGGAGTACGATCGAGTGTACAGTGAA
MT451175.1 Severe acute respiratory syndrome coronavirus 2 isolate SARS-CoV-2/human/AUS/VIC279/2020, comple	TTCACCGAGGCCACGCGGAGTACGATCGAGTGTACAGTGAA
MT451176.1 Severe acute respiratory syndrome coronavirus 2 isolate SARS-CoV-2/human/AUS/VIC280/2020, comple	TTCACCGAGGCCACGCGGAGTACGATCGAGTGTACAGTGAA
MT451177.1 Severe acute respiratory syndrome coronavirus 2 isolate SARS-CoV-2/human/AUS/VIC283/2020, comple	TTCACCGAGGCCACGCGGAGTACGATCGAGTGTACAGTGAA
MT451179.1 Severe acute respiratory syndrome coronavirus 2 isolate SARS-CoV-2/human/AUS/VIC285/2020, comple	TTCACCGAGGCCACGCGGAGTACGATCGAGTGTACAGTGAA
MT451180.1 Severe acute respiratory syndrome coronavirus 2 isolate SARS-CoV-2/human/AUS/VIC286/2020, comple	TTCACCGAGGCCACGCGGAGTACGATCGAGTGTACAGTGAA
MT451181.1 Severe acute respiratory syndrome coronavirus 2 isolate SARS-CoV-2/human/AUS/VIC287/2020, comple	TTCACCGAGGCCACGCGGAGTACGATCGAGTGTACAGTGAA
MT451182.1 Severe acute respiratory syndrome coronavirus 2 isolate SARS-CoV-2/human/AUS/VIC288/2020, comple	TTCACCGAGGCCACGCGGAGTACGATCGAGTGTACAGTGAA
MT451183.1 Severe acute respiratory syndrome coronavirus 2 isolate SARS-CoV-2/human/AUS/VIC289/2020, comple	TTCACCGAGGCCACGCGGAGTACGATCGAGTGTACAGTGAA
MT451184.1 Severe acute respiratory syndrome coronavirus 2 isolate SARS-CoV-2/human/AUS/VIC292/2020, comple	TTCACCGAGGCCACGCGGAGTACGATCGAGTGTACAGTGAA
MT451185.1 Severe acute respiratory syndrome coronavirus 2 isolate SARS-CoV-2/human/AUS/VIC293/2020, comple	TTCACCGAGGCCACGCGGAGTACGATCGAGTGTACAGTGAA
MT451187.1 Severe acute respiratory syndrome coronavirus 2 isolate SARS-CoV-2/human/AUS/VIC295/2020, comple	TTCACCGAGGCCACGCGGAGTACGATCGAGTGTACAGTGAA
MT451188.1 Severe acute respiratory syndrome coronavirus 2 isolate SARS-CoV-2/human/AUS/VIC296/2020, comple	TTCACCGAGGCCACGCGGAGTACGATCGAGTGTACAGTGAA
MT451189.1 Severe acute respiratory syndrome coronavirus 2 isolate SARS-CoV-2/human/AUS/VIC297/2020, comple	TTCACCGAGGCCACGCGGAGTACGATCGAGTGTACAGTGAA
MT451190.1 Severe acute respiratory syndrome coronavirus 2 isolate SARS-CoV-2/human/AUS/VIC298/2020, comple	TTCACCGAGGCCACGCGGAGTACGATCGAGTGTACAGTGAA
MT451191.1 Severe acute respiratory syndrome coronavirus 2 isolate SARS-CoV-2/human/AUS/VIC299/2020, comple	TTCACCGAGGCCACGCGGAGTACGATCGAGTGTACAGTGAA
MT451192.1 Severe acute respiratory syndrome coronavirus 2 isolate SARS-CoV-2/human/AUS/VIC300/2020, comple	TTCACCGAGGCCACGCGGAGTACGATCGAGTGTACAGTGAA
MT451193.1 Severe acute respiratory syndrome coronavirus 2 isolate SARS-CoV-2/human/AUS/VIC301/2020 ORF1ab	TTCACCGAGGCCACGCGGAGTACGATCGAGTGTACAGTGAA
MT451195.1 Severe acute respiratory syndrome coronavirus 2 isolate SARS-CoV-2/human/AUS/VIC303/2020, comple	TTCACCGAGGCCACGCGGAGTACGATCGAGTGTACAGTGAA
MT451197.1 Severe acute respiratory syndrome coronavirus 2 isolate SARS-CoV-2/human/AUS/VIC305/2020, comple	TTCACCGAGGCCACGCGGAGTACGATCGAGTGTACAGTGAA
MT451198.1 Severe acute respiratory syndrome coronavirus 2 isolate SARS-CoV-2/human/AUS/VIC306/2020, comple	TTCACCGAGGCCACGCGGAGTACGATCGAGTGTACAGTGAA
MT451199.1 Severe acute respiratory syndrome coronavirus 2 isolate SARS-CoV-2/human/AUS/VIC307/2020, comple	TTCACCGAGGCCACGCGGAGTACGATCGAGTGTACAGTGAA
MT451200.1 Severe acute respiratory syndrome coronavirus 2 isolate SARS-CoV-2/human/AUS/VIC308/2020 ORF1ab	TTCACCGAGGCCACGCGGAGTACGATCGAGTGTACAGTGAA
MT451201.1 Severe acute respiratory syndrome coronavirus 2 isolate SARS-CoV-2/human/AUS/VIC309/2020, comple	TTCACCGAGGCCACGCGGAGTACGATCGAGTGTACAGTGAA
MT451202.1 Severe acute respiratory syndrome coronavirus 2 isolate SARS-CoV-2/human/AUS/VIC310/2020, comple	TTCACCGAGGCCACGCGGAGTACGATCGAGTGTACAGTGAA

MT451203.1 Severe acute respiratory syndrome coronavirus 2 isolate SARS-CoV-2/human/AUS/VIC311/2020, comple	TTCACCGAGGCCACGCGGAGTACGATCGAGTGTACAGTGAA
MT451204.1 Severe acute respiratory syndrome coronavirus 2 isolate SARS-CoV-2/human/AUS/VIC312/2020, comple	TTCACCGAGGCCACGCGGAGTACGATCGAGTGTACAGTGAA
MT451207.1 Severe acute respiratory syndrome coronavirus 2 isolate SARS-CoV-2/human/AUS/VIC315/2020, comple	TTCACCGAGGCCACGCGGAGTACGATCGAGTGTACAGTGAA
MT451209.1 Severe acute respiratory syndrome coronavirus 2 isolate SARS-CoV-2/human/AUS/VIC317/2020, comple	TTCACCGAGGCCACGCGGAGTACGATCGAGTGTACAGTGAA
MT451210.1 Severe acute respiratory syndrome coronavirus 2 isolate SARS-CoV-2/human/AUS/VIC318/2020, comple	TTCACCGAGGCCACGCGGAGTACGATCGAGTGTACAGTGAA
MT451211.1 Severe acute respiratory syndrome coronavirus 2 isolate SARS-CoV-2/human/AUS/VIC319/2020, comple	TTCACCGAGGCCACGCGGAGTACGATCGAGTGTACAGTGAA
MT451212.1 Severe acute respiratory syndrome coronavirus 2 isolate SARS-CoV-2/human/AUS/VIC320/2020, comple	TTCACCGAGGCCACGCGGAGTACGATCGAGTGTACAGTGAA
MT451213.1 Severe acute respiratory syndrome coronavirus 2 isolate SARS-CoV-2/human/AUS/VIC321/2020, comple	TTCACCGAGGCCACGCGGAGTACGATCGAGTGTACAGTGAA
MT451214.1 Severe acute respiratory syndrome coronavirus 2 isolate SARS-CoV-2/human/AUS/VIC322/2020, comple	TTCACCGAGGCCACGCGGAGTACGATCGAGTGTACAGTGAA
MT451215.1 Severe acute respiratory syndrome coronavirus 2 isolate SARS-CoV-2/human/AUS/VIC323/2020, comple	TTCACCGAGGCCACGCGGAGTACGATCGAGTGTACAGTGAA
MT451216.1 Severe acute respiratory syndrome coronavirus 2 isolate SARS-CoV-2/human/AUS/VIC324/2020, comple	TTCACCGAGGCCACGCGGAGTACGATCGAGTGTACAGTGAA
MT451218.1 Severe acute respiratory syndrome coronavirus 2 isolate SARS-CoV-2/human/AUS/VIC326/2020, comple	TTCACCGAGGCCACGCGGAGTACGATCGAGTGTACAGTGAA
MT451219.1 Severe acute respiratory syndrome coronavirus 2 isolate SARS-CoV-2/human/AUS/VIC327/2020, comple	TTCACCGAGGCCACGCGGAGTACGATCGAGTGTACAGTGAA
MT451220.1 Severe acute respiratory syndrome coronavirus 2 isolate SARS-CoV-2/human/AUS/VIC328/2020, comple	TTCACCGAGGCCACGCGGAGTACGATCGAGTGTACAGTGAA
MT451223.1 Severe acute respiratory syndrome coronavirus 2 isolate SARS-CoV-2/human/AUS/VIC332/2020 ORF1ab	TTCACCGAGGCCACGCGGAGTACGATCGAGTGTACAGTGAA
MT451224.1 Severe acute respiratory syndrome coronavirus 2 isolate SARS-CoV-2/human/AUS/VIC333/2020, comple	TTCACCGAGGCCACGCGGAGTACGATCGAGTGTACAGTGAA
MT451225.1 Severe acute respiratory syndrome coronavirus 2 isolate SARS-CoV-2/human/AUS/VIC334/2020, comple	TTCACCGAGGCCACGCGGAGTACGATCGAGTGTACAGTGAA
MT451226.1 Severe acute respiratory syndrome coronavirus 2 isolate SARS-CoV-2/human/AUS/VIC335/2020, comple	TTCACCGAGGCCACGCGGAGTACGATCGAGTGTACAGTGAA
MT451227.1 Severe acute respiratory syndrome coronavirus 2 isolate SARS-CoV-2/human/AUS/VIC336/2020, comple	TTCACCGAGGCCACGCGGAGTACGATCGAGTGTACAGTGAA
MT451229.1 Severe acute respiratory syndrome coronavirus 2 isolate SARS-CoV-2/human/AUS/VIC338/2020, comple	TTCACCGAGGCCACGCGGAGTACGATCGAGTGTACAGTGAA
MT451230.1 Severe acute respiratory syndrome coronavirus 2 isolate SARS-CoV-2/human/AUS/VIC339/2020, comple	TTCACCGAGGCCACGCGGAGTACGATCGAGTGTACAGTGAA
MT451231.1 Severe acute respiratory syndrome coronavirus 2 isolate SARS-CoV-2/human/AUS/VIC340/2020, comple	TTCACCGAGGCCACGCGGAGTACGATCGAGTGTACAGTGAA
MT451232.1 Severe acute respiratory syndrome coronavirus 2 isolate SARS-CoV-2/human/AUS/VIC341/2020, comple	TTCACCGAGGCCACGCGGAGTACGATCGAGTGTACAGTGAA
MT451233.1 Severe acute respiratory syndrome coronavirus 2 isolate SARS-CoV-2/human/AUS/VIC342/2020, comple	TTCACCGAGGCCACGCGGAGTACGATCGAGTGTACAGTGAA
MT451234.1 Severe acute respiratory syndrome coronavirus 2 isolate SARS-CoV-2/human/AUS/VIC343/2020 ORF1ab	TTCACCGAGGCCACGCGGAGTACGATCGAGTGTACAGTGAA
MT451235.1 Severe acute respiratory syndrome coronavirus 2 isolate SARS-CoV-2/human/AUS/VIC344/2020, comple	TTCACCGAGGCCACGCGGAGTACGATCGAGTGTACAGTGAA
MT451236.1 Severe acute respiratory syndrome coronavirus 2 isolate SARS-CoV-2/human/AUS/VIC345/2020, comple	TTCACCGAGGCCACGCGGAGTACGATCGAGTGTACAGTGAA
MT451238.1 Severe acute respiratory syndrome coronavirus 2 isolate SARS-CoV-2/human/AUS/VIC347/2020, comple	TTCACCGAGGCCACGCGGAGTACGATCGAGTGTACAGTGAA
MT451239.1 Severe acute respiratory syndrome coronavirus 2 isolate SARS-CoV-2/human/AUS/VIC348/2020, comple	TTCACCGAGGCCACGCGGAGTACGATCGAGTGTACAGTGAA
MT451240.1 Severe acute respiratory syndrome coronavirus 2 isolate SARS-CoV-2/human/AUS/VIC349/2020, comple	TTCACCGAGGCCACGCGGAGTACGATCGAGTGTACAGTGAA
MT451241.1 Severe acute respiratory syndrome coronavirus 2 isolate SARS-CoV-2/human/AUS/VIC350/2020, comple	TTCACCGAGGCCACGCGGAGTACGATCGAGTGTACAGTGAA
MT451242.1 Severe acute respiratory syndrome coronavirus 2 isolate SARS-CoV-2/human/AUS/VIC351/2020, comple	TTCACCGAGGCCACGCGGAGTACGATCGAGTGTACAGTGAA
MT451244.1 Severe acute respiratory syndrome coronavirus 2 isolate SARS-CoV-2/human/AUS/VIC353/2020, comple	TTCACCGAGGCCACGCGGAGTACGATCGAGTGTACAGTGAA

MT451245.1 Severe acute respiratory syndrome coronavirus 2 isolate SARS-CoV-2/human/AUS/VIC354/2020, comple	TTCACCGAGGCCACGCGGAGTACGATCGAGTGTACAGTGAA
MT451246.1 Severe acute respiratory syndrome coronavirus 2 isolate SARS-CoV-2/human/AUS/VIC355/2020, comple	TTCACCGAGGCCACGCGGAGTACGATCGAGTGTACAGTGAA
MT451247.1 Severe acute respiratory syndrome coronavirus 2 isolate SARS-CoV-2/human/AUS/VIC356/2020, comple	TTCACCGAGGCCACGCGGAGTACGATCGAGTGTACAGTGAA
MT451248.1 Severe acute respiratory syndrome coronavirus 2 isolate SARS-CoV-2/human/AUS/VIC357/2020, comple	TTCACCGAGGCCACGCGGAGTACGATCGAGTGTACAGTGAA
MT451250.1 Severe acute respiratory syndrome coronavirus 2 isolate SARS-CoV-2/human/AUS/VIC359/2020 ORF1ab	TTCACCGAGGCCACGCGGAGTACGATCGAGTGTACAGTGAA
MT451252.1 Severe acute respiratory syndrome coronavirus 2 isolate SARS-CoV-2/human/AUS/VIC361/2020 ORF1ab	TTCACCGAGGCCACGCGGAGTACGATCGAGTGTACAGTGAA
MT451253.1 Severe acute respiratory syndrome coronavirus 2 isolate SARS-CoV-2/human/AUS/VIC362/2020, comple	TTCACCGAGGCCACGCGGAGTACGATCGAGTGTACAGTGAA
MT451254.1 Severe acute respiratory syndrome coronavirus 2 isolate SARS-CoV-2/human/AUS/VIC363/2020 ORF1ab	TTCACCGAGGCCACGCGGAGTACGATCGAGTGTACAGTGAA
MT451255.1 Severe acute respiratory syndrome coronavirus 2 isolate SARS-CoV-2/human/AUS/VIC364/2020, comple	TTCACCGAGGCCACGCGGAGTACGATCGAGTGTACAGTGAA
MT451256.1 Severe acute respiratory syndrome coronavirus 2 isolate SARS-CoV-2/human/AUS/VIC365/2020, comple	TTCACCGAGGCCACGCGGAGTACGATCGAGTGTACAGTGAA
MT451258.1 Severe acute respiratory syndrome coronavirus 2 isolate SARS-CoV-2/human/AUS/VIC367/2020, comple	TTCACCGAGGCCACGCGGAGTACGATCGAGTGTACAGTGAA
MT451260.1 Severe acute respiratory syndrome coronavirus 2 isolate SARS-CoV-2/human/AUS/VIC372/2020 ORF1ab	TTCACCGAGGCCACGCGGAGTACGATCGAGTGTACAGTGAA
MT451264.1 Severe acute respiratory syndrome coronavirus 2 isolate SARS-CoV-2/human/AUS/VIC376/2020, comple	TTCACCGAGGCCACGCGGAGTACGATCGAGTGTACAGTGAA
MT451267.1 Severe acute respiratory syndrome coronavirus 2 isolate SARS-CoV-2/human/AUS/VIC380/2020 ORF1ab	TTCACCGAGGCCACGCGGAGTACGATCGAGTGTACAGTGAA
MT451268.1 Severe acute respiratory syndrome coronavirus 2 isolate SARS-CoV-2/human/AUS/VIC381/2020 ORF1ab	TTCACCGAGGCCACGCGGAGTACGATCGAGTGTACAGTGAA
MT451270.1 Severe acute respiratory syndrome coronavirus 2 isolate SARS-CoV-2/human/AUS/VIC383/2020 ORF1ab	TTCACCGAGGCCACGCGGAGTACGATCGAGTGTACAGTGAA
MT451271.1 Severe acute respiratory syndrome coronavirus 2 isolate SARS-CoV-2/human/AUS/VIC384/2020 ORF1ab	TTCACCGAGGCCACGCGGAGTACGATCGAGTGTACAGTGAA
MT451274.1 Severe acute respiratory syndrome coronavirus 2 isolate SARS-CoV-2/human/AUS/VIC387/2020, comple	TTCACCGAGGCCACGCGGAGTACGATCGAGTGTACAGTGAA
MT451277.1 Severe acute respiratory syndrome coronavirus 2 isolate SARS-CoV-2/human/AUS/VIC390/2020 ORF1ab	TTCACCGAGGCCACGCGGAGTACGATCGAGTGTACAGTGAA
MT451278.1 Severe acute respiratory syndrome coronavirus 2 isolate SARS-CoV-2/human/AUS/VIC394/2020, comple	TTCACCGAGGCCACGCGGAGTACGATCGAGTGTACAGTGAA
MT451281.1 Severe acute respiratory syndrome coronavirus 2 isolate SARS-CoV-2/human/AUS/VIC398/2020, comple	TTCACCGAGGCCACGCGGAGTACGATCGAGTGTACAGTGAA
MT451284.1 Severe acute respiratory syndrome coronavirus 2 isolate SARS-CoV-2/human/AUS/VIC404/2020, comple	TTCACCGAGGCCACGCGGAGTACGATCGAGTGTACAGTGAA
MT451288.1 Severe acute respiratory syndrome coronavirus 2 isolate SARS-CoV-2/human/AUS/VIC409/2020 ORF1ab	TTCACCGAGGCCACGCGGAGTACGATCGAGTGTACAGTGAA
MT451291.1 Severe acute respiratory syndrome coronavirus 2 isolate SARS-CoV-2/human/AUS/VIC415/2020 ORF1ab	TTCACCGAGGCCACGCGGAGTACGATCGAGTGTACAGTGAA
MT451295.1 Severe acute respiratory syndrome coronavirus 2 isolate SARS-CoV-2/human/AUS/VIC421/2020 ORF1ab	TTCACCGAGGCCACGCGGAGTACGATCGAGTGTACAGTGAA
MT451301.1 Severe acute respiratory syndrome coronavirus 2 isolate SARS-CoV-2/human/AUS/VIC427/2020 ORF1ab	TTCACCGAGGCCACGCGGAGTACGATCGAGTGTACAGTGAA
MT451303.1 Severe acute respiratory syndrome coronavirus 2 isolate SARS-CoV-2/human/AUS/VIC429/2020 ORF1ab	TTCACCGAGGCCACGCGGAGTACGATCGAGTGTACAGTGAA
MT451309.1 Severe acute respiratory syndrome coronavirus 2 isolate SARS-CoV-2/human/AUS/VIC438/2020 ORF1ab	TTCACCGAGGCCACGCGGAGTACGATCGAGTGTACAGTGAA
MT451310.1 Severe acute respiratory syndrome coronavirus 2 isolate SARS-CoV-2/human/AUS/VIC439/2020 ORF1ab	TTCACCGAGGCCACGCGGAGTACGATCGAGTGTACAGTGAA
MT451311.1 Severe acute respiratory syndrome coronavirus 2 isolate SARS-CoV-2/human/AUS/VIC440/2020 ORF1ab	TTCACCGAGGCCACGCGGAGTACGATCGAGTGTACAGTGAA
MT451314.1 Severe acute respiratory syndrome coronavirus 2 isolate SARS-CoV-2/human/AUS/VIC443/2020 ORF1ab	TTCACCGAGGCCACGCGGAGTACGATCGAGTGTACAGTGAA
MT451317.1 Severe acute respiratory syndrome coronavirus 2 isolate SARS-CoV-2/human/AUS/VIC449/2020 ORF1ab	TTCACCGAGGCCACGCGGAGTACGATCGAGTGTACAGTGAA
MT451318.1 Severe acute respiratory syndrome coronavirus 2 isolate SARS-CoV-2/human/AUS/VIC450/2020 ORF1ab	TTCACCGAGGCCACGCGGAGTACGATCGAGTGTACAGTGAA

MT451319.1 Severe acute respiratory syndrome coronavirus 2 isolate SARS-CoV-2/human/AUS/VIC451/2020 ORF1ab	TTCACCGAGGCCACGCGGAGTACGATCGAGTGTACAGTGAA
MT451324.1 Severe acute respiratory syndrome coronavirus 2 isolate SARS-CoV-2/human/AUS/VIC458/2020, comple	TTCACCGAGGCCACGCGGAGTACGATCGAGTGTACAGTGAA
MT451328.1 Severe acute respiratory syndrome coronavirus 2 isolate SARS-CoV-2/human/AUS/VIC463/2020 ORF1ab	TTCACCGAGGCCACGCGGAGTACGATCGAGTGTACAGTGAA
MT451341.1 Severe acute respiratory syndrome coronavirus 2 isolate SARS-CoV-2/human/AUS/VIC483/2020 ORF1ab	TTCACCGAGGCCACGCGGAGTACGATCGAGTGTACAGTGAA
MT451347.1 Severe acute respiratory syndrome coronavirus 2 isolate SARS-CoV-2/human/AUS/VIC489/2020 ORF1ab	TTCACCGAGGCCACGCGGAGTACGATCGAGTGTACAGTGAA
MT451353.1 Severe acute respiratory syndrome coronavirus 2 isolate SARS-CoV-2/human/AUS/VIC496/2020 ORF1ab	TTCACCGAGGCCACGCGGAGTACGATCGAGTGTACAGTGAA
MT451355.1 Severe acute respiratory syndrome coronavirus 2 isolate SARS-CoV-2/human/AUS/VIC500/2020, comple	TTCACCGAGGCCACGCGGAGTACGATCGAGTGTACAGTGAA
MT451356.1 Severe acute respiratory syndrome coronavirus 2 isolate SARS-CoV-2/human/AUS/VIC504/2020, comple	TTCACCGAGGCCACGCGGAGTACGATCGAGTGTACAGTGAA
MT451361.1 Severe acute respiratory syndrome coronavirus 2 isolate SARS-CoV-2/human/AUS/VIC527/2020 ORF1ab	TTCACCGAGGCCACGCGGAGTACGATCGAGTGTACAGTGAA
MT451362.1 Severe acute respiratory syndrome coronavirus 2 isolate SARS-CoV-2/human/AUS/VIC528/2020 ORF1ab	TTCACCGAGGCCACGCGGAGTACGATCGAGTGTACAGTGAA
MT451364.1 Severe acute respiratory syndrome coronavirus 2 isolate SARS-CoV-2/human/AUS/VIC530/2020 ORF1ab	TTCACCGAGGCCACGCGGAGTACGATCGAGTGTACAGTGAA
MT451365.1 Severe acute respiratory syndrome coronavirus 2 isolate SARS-CoV-2/human/AUS/VIC531/2020 ORF1ab	TTCACCGAGGCCACGCGGAGTACGATCGAGTGTACAGTGAA
MT451366.1 Severe acute respiratory syndrome coronavirus 2 isolate SARS-CoV-2/human/AUS/VIC532/2020, comple	TTCACCGAGGCCACGCGGAGTACGATCGAGTGTACAGTGAA
MT451367.1 Severe acute respiratory syndrome coronavirus 2 isolate SARS-CoV-2/human/AUS/VIC533/2020 ORF1ab	TTCACCGAGGCCACGCGGAGTACGATCGAGTGTACAGTGAA
MT451370.1 Severe acute respiratory syndrome coronavirus 2 isolate SARS-CoV-2/human/AUS/VIC538/2020 ORF1ab	TTCACCGAGGCCACGCGGAGTACGATCGAGTGTACAGTGAA
MT451372.1 Severe acute respiratory syndrome coronavirus 2 isolate SARS-CoV-2/human/AUS/VIC544/2020 ORF1ab	TTCACCGAGGCCACGCGGAGTACGATCGAGTGTACAGTGAA
MT451383.1 Severe acute respiratory syndrome coronavirus 2 isolate SARS-CoV-2/human/AUS/VIC558/2020 ORF1ab	TTCACCGAGGCCACGCGGAGTACGATCGAGTGTACAGTGAA
MT451388.1 Severe acute respiratory syndrome coronavirus 2 isolate SARS-CoV-2/human/AUS/VIC563/2020 ORF1ab	TTCACCGAGGCCACGCGGAGTACGATCGAGTGTACAGTGAA
MT451390.1 Severe acute respiratory syndrome coronavirus 2 isolate SARS-CoV-2/human/AUS/VIC566/2020 ORF1ab	TTCACCGAGGCCACGCGGAGTACGATCGAGTGTACAGTGAA
MT451392.1 Severe acute respiratory syndrome coronavirus 2 isolate SARS-CoV-2/human/AUS/VIC572/2020, comple	TTCACCGAGGCCACGCGGAGTACGATCGAGTGTACAGTGAA
MT451398.1 Severe acute respiratory syndrome coronavirus 2 isolate SARS-CoV-2/human/AUS/VIC582/2020 ORF1ab	TTCACCGAGGCCACGCGGAGTACGATCGAGTGTACAGTGAA
MT451408.1 Severe acute respiratory syndrome coronavirus 2 isolate SARS-CoV-2/human/AUS/VIC593/2020 ORF1ab	TTCACCGAGGCCACGCGGAGTACGATCGAGTGTACAGTGAA
MT451411.1 Severe acute respiratory syndrome coronavirus 2 isolate SARS-CoV-2/human/AUS/VIC598/2020, comple	TTCACCGAGGCCACGCGGAGTACGATCGAGTGTACAGTGAA
MT451412.1 Severe acute respiratory syndrome coronavirus 2 isolate SARS-CoV-2/human/AUS/VIC599/2020, comple	TTCACCGAGGCCACGCGGAGTACGATCGAGTGTACAGTGAA
MT451414.1 Severe acute respiratory syndrome coronavirus 2 isolate SARS-CoV-2/human/AUS/VIC605/2020 ORF1ab	TTCACCGAGGCCACGCGGAGTACGATCGAGTGTACAGTGAA
MT451415.1 Severe acute respiratory syndrome coronavirus 2 isolate SARS-CoV-2/human/AUS/VIC606/2020, comple	TTCACCGAGGCCACGCGGAGTACGATCGAGTGTACAGTGAA
MT451416.1 Severe acute respiratory syndrome coronavirus 2 isolate SARS-CoV-2/human/AUS/VIC607/2020 ORF1ab	TTCACCGAGGCCACGCGGAGTACGATCGAGTGTACAGTGAA
MT451418.1 Severe acute respiratory syndrome coronavirus 2 isolate SARS-CoV-2/human/AUS/VIC610/2020 ORF1ab	TTCACCGAGGCCACGCGGAGTACGATCGAGTGTACAGTGAA
MT451419.1 Severe acute respiratory syndrome coronavirus 2 isolate SARS-CoV-2/human/AUS/VIC611/2020 ORF1ab	TTCACCGAGGCCACGCGGAGTACGATCGAGTGTACAGTGAA
MT451425.1 Severe acute respiratory syndrome coronavirus 2 isolate SARS-CoV-2/human/AUS/VIC617/2020 ORF1ab	TTCACCGAGGCCACGCGGAGTACGATCGAGTGTACAGTGAA
MT451426.1 Severe acute respiratory syndrome coronavirus 2 isolate SARS-CoV-2/human/AUS/VIC619/2020 ORF1ab	TTCACCGAGGCCACGCGGAGTACGATCGAGTGTACAGTGAA
MT451427.1 Severe acute respiratory syndrome coronavirus 2 isolate SARS-CoV-2/human/AUS/VIC620/2020 ORF1ab	TTCACCGAGGCCACGCGGAGTACGATCGAGTGTACAGTGAA
MT451429.1 Severe acute respiratory syndrome coronavirus 2 isolate SARS-CoV-2/human/AUS/VIC622/2020 ORF1ab	TTCACCGAGGCCACGCGGAGTACGATCGAGTGTACAGTGAA

MT451430.1 Severe acute respiratory syndrome coronavirus 2 isolate SARS-CoV-2/human/AUS/VIC623/2020 ORF1ab	TTCACCGAGGCCACGCGGAGTACGATCGAGTGTACAGTGAA
MT451432.1 Severe acute respiratory syndrome coronavirus 2 isolate SARS-CoV-2/human/AUS/VIC625/2020 ORF1ab	TTCACCGAGGCCACGCGGAGTACGATCGAGTGTACAGTGAA
MT451434.1 Severe acute respiratory syndrome coronavirus 2 isolate SARS-CoV-2/human/AUS/VIC628/2020, comple	TTCACCGAGGCCACGCGGAGTACGATCGAGTGTACAGTGAA
MT451438.1 Severe acute respiratory syndrome coronavirus 2 isolate SARS-CoV-2/human/AUS/VIC635/2020 ORF1ab	TTCACCGAGGCCACGCGGAGTACGATCGAGTGTACAGTGAA
MT451439.1 Severe acute respiratory syndrome coronavirus 2 isolate SARS-CoV-2/human/AUS/VIC636/2020, comple	TTCACCGAGGCCACGCGGAGTACGATCGAGTGTACAGTGAA
MT451440.1 Severe acute respiratory syndrome coronavirus 2 isolate SARS-CoV-2/human/AUS/VIC637/2020 ORF1ab	TTCACCGAGGCCACGCGGAGTACGATCGAGTGTACAGTGAA
MT451441.1 Severe acute respiratory syndrome coronavirus 2 isolate SARS-CoV-2/human/AUS/VIC638/2020, comple	TTCACCGAGGCCACGCGGAGTACGATCGAGTGTACAGTGAA
MT451442.1 Severe acute respiratory syndrome coronavirus 2 isolate SARS-CoV-2/human/AUS/VIC639/2020, comple	TTCACCGAGGCCACGCGGAGTACGATCGAGTGTACAGTGAA
MT451444.1 Severe acute respiratory syndrome coronavirus 2 isolate SARS-CoV-2/human/AUS/VIC642/2020 ORF1ab	TTCACCGAGGCCACGCGGAGTACGATCGAGTGTACAGTGAA
MT451445.1 Severe acute respiratory syndrome coronavirus 2 isolate SARS-CoV-2/human/AUS/VIC643/2020 ORF1ab	TTCACCGAGGCCACGCGGAGTACGATCGAGTGTACAGTGAA
MT451446.1 Severe acute respiratory syndrome coronavirus 2 isolate SARS-CoV-2/human/AUS/VIC644/2020 ORF1ab	TTCACCGAGGCCACGCGGAGTACGATCGAGTGTACAGTGAA
MT451448.1 Severe acute respiratory syndrome coronavirus 2 isolate SARS-CoV-2/human/AUS/VIC646/2020, comple	TTCACCGAGGCCACGCGGAGTACGATCGAGTGTACAGTGAA
MT451449.1 Severe acute respiratory syndrome coronavirus 2 isolate SARS-CoV-2/human/AUS/VIC647/2020, comple	TTCACCGAGGCCACGCGGAGTACGATCGAGTGTACAGTGAA
MT451456.1 Severe acute respiratory syndrome coronavirus 2 isolate SARS-CoV-2/human/AUS/VIC656/2020, comple	TTCACCGAGGCCACGCGGAGTACGATCGAGTGTACAGTGAA
MT451457.1 Severe acute respiratory syndrome coronavirus 2 isolate SARS-CoV-2/human/AUS/VIC657/2020 ORF1ab	TTCACCGAGGCCACGCGGAGTACGATCGAGTGTACAGTGAA
MT451459.1 Severe acute respiratory syndrome coronavirus 2 isolate SARS-CoV-2/human/AUS/VIC659/2020 ORF1ab	TTCACCGAGGCCACGCGGAGTACGATCGAGTGTACAGTGAA
MT451460.1 Severe acute respiratory syndrome coronavirus 2 isolate SARS-CoV-2/human/AUS/VIC660/2020 ORF1ab	TTCACCGAGGCCACGCGGAGTACGATCGAGTGTACAGTGAA
MT451462.1 Severe acute respiratory syndrome coronavirus 2 isolate SARS-CoV-2/human/AUS/VIC662/2020 ORF1ab	TTCACCGAGGCCACGCGGAGTACGATCGAGTGTACAGTGAA
MT451465.1 Severe acute respiratory syndrome coronavirus 2 isolate SARS-CoV-2/human/AUS/VIC666/2020 ORF1ab	TTCACCGAGGCCACGCGGAGTACGATCGAGTGTACAGTGAA
MT451466.1 Severe acute respiratory syndrome coronavirus 2 isolate SARS-CoV-2/human/AUS/VIC667/2020, comple	TTCACCGAGGCCACGCGGAGTACGATCGAGTGTACAGTGAA
MT451467.1 Severe acute respiratory syndrome coronavirus 2 isolate SARS-CoV-2/human/AUS/VIC668/2020 ORF1ab	TTCACCGAGGCCACGCGGAGTACGATCGAGTGTACAGTGAA
MT451470.1 Severe acute respiratory syndrome coronavirus 2 isolate SARS-CoV-2/human/AUS/VIC672/2020, comple	TTCACCGAGGCCACGCGGAGTACGATCGAGTGTACAGTGAA
MT451472.1 Severe acute respiratory syndrome coronavirus 2 isolate SARS-CoV-2/human/AUS/VIC674/2020 ORF1ab	TTCACCGAGGCCACGCGGAGTACGATCGAGTGTACAGTGAA
MT451473.1 Severe acute respiratory syndrome coronavirus 2 isolate SARS-CoV-2/human/AUS/VIC676/2020 ORF1ab	TTCACCGAGGCCACGCGGAGTACGATCGAGTGTACAGTGAA
MT451478.1 Severe acute respiratory syndrome coronavirus 2 isolate SARS-CoV-2/human/AUS/VIC682/2020, comple	TTCACCGAGGCCACGCGGAGTACGATCGAGTGTACAGTGAA
MT451482.1 Severe acute respiratory syndrome coronavirus 2 isolate SARS-CoV-2/human/AUS/VIC687/2020 ORF1ab	TTCACCGAGGCCACGCGGAGTACGATCGAGTGTACAGTGAA
MT451484.1 Severe acute respiratory syndrome coronavirus 2 isolate SARS-CoV-2/human/AUS/VIC693/2020 ORF1ab	TTCACCGAGGCCACGCGGAGTACGATCGAGTGTACAGTGAA
MT451485.1 Severe acute respiratory syndrome coronavirus 2 isolate SARS-CoV-2/human/AUS/VIC694/2020 ORF1ab	TTCACCGAGGCCACGCGGAGTACGATCGAGTGTACAGTGAA
MT451487.1 Severe acute respiratory syndrome coronavirus 2 isolate SARS-CoV-2/human/AUS/VIC696/2020 ORF1ab	TTCACCGAGGCCACGCGGAGTACGATCGAGTGTACAGTGAA
MT451488.1 Severe acute respiratory syndrome coronavirus 2 isolate SARS-CoV-2/human/AUS/VIC697/2020, comple	TTCACCGAGGCCACGCGGAGTACGATCGAGTGTACAGTGAA
MT451493.1 Severe acute respiratory syndrome coronavirus 2 isolate SARS-CoV-2/human/AUS/VIC707/2020 ORF1ab	TTCACCGAGGCCACGCGGAGTACGATCGAGTGTACAGTGAA
MT451496.1 Severe acute respiratory syndrome coronavirus 2 isolate SARS-CoV-2/human/AUS/VIC712/2020, comple	TTCACCGAGGCCACGCGGAGTACGATCGAGTGTACAGTGAA
MT451498.1 Severe acute respiratory syndrome coronavirus 2 isolate SARS-CoV-2/human/AUS/VIC716/2020 ORF1ab	TTCACCGAGGCCACGCGGAGTACGATCGAGTGTACAGTGAA

MT451506.1 Severe acute respiratory syndrome coronavirus 2 isolate SARS-CoV-2/human/AUS/VIC731/2020 ORF1ab	TTCACCGAGGCCACGCGGAGTACGATCGAGTGTACAGTGAA
MT451507.1 Severe acute respiratory syndrome coronavirus 2 isolate SARS-CoV-2/human/AUS/VIC732/2020 ORF1ab	TTCACCGAGGCCACGCGGAGTACGATCGAGTGTACAGTGAA
MT451509.1 Severe acute respiratory syndrome coronavirus 2 isolate SARS-CoV-2/human/AUS/VIC734/2020 ORF1ab	TTCACCGAGGCCACGCGGAGTACGATCGAGTGTACAGTGAA
MT451510.1 Severe acute respiratory syndrome coronavirus 2 isolate SARS-CoV-2/human/AUS/VIC735/2020 ORF1ab	TTCACCGAGGCCACGCGGAGTACGATCGAGTGTACAGTGAA
MT451511.1 Severe acute respiratory syndrome coronavirus 2 isolate SARS-CoV-2/human/AUS/VIC736/2020 ORF1ab	TTCACCGAGGCCACGCGGAGTACGATCGAGTGTACAGTGAA
MT451512.1 Severe acute respiratory syndrome coronavirus 2 isolate SARS-CoV-2/human/AUS/VIC737/2020 ORF1ab	TTCACCGAGGCCACGCGGAGTACGATCGAGTGTACAGTGAA
MT451513.1 Severe acute respiratory syndrome coronavirus 2 isolate SARS-CoV-2/human/AUS/VIC738/2020 ORF1ab	TTCACCGAGGCCACGCGGAGTACGATCGAGTGTACAGTGAA
MT451514.1 Severe acute respiratory syndrome coronavirus 2 isolate SARS-CoV-2/human/AUS/VIC739/2020 ORF1ab	TTCACCGAGGCCACGCGGAGTACGATCGAGTGTACAGTGAA
MT451515.1 Severe acute respiratory syndrome coronavirus 2 isolate SARS-CoV-2/human/AUS/VIC741/2020 ORF1ab	TTCACCGAGGCCACGCGGAGTACGATCGAGTGTACAGTGAA
MT451516.1 Severe acute respiratory syndrome coronavirus 2 isolate SARS-CoV-2/human/AUS/VIC742/2020 ORF1ab	TTCACCGAGGCCACGCGGAGTACGATCGAGTGTACAGTGAA
MT451518.1 Severe acute respiratory syndrome coronavirus 2 isolate SARS-CoV-2/human/AUS/VIC745/2020 ORF1ab	TTCACCGAGGCCACGCGGAGTACGATCGAGTGTACAGTGAA
MT451519.1 Severe acute respiratory syndrome coronavirus 2 isolate SARS-CoV-2/human/AUS/VIC746/2020 ORF1ab	TTCACCGAGGCCACGCGGAGTACGATCGAGTGTACAGTGAA
MT451522.1 Severe acute respiratory syndrome coronavirus 2 isolate SARS-CoV-2/human/AUS/VIC751/2020 ORF1ab	TTCACCGAGGCCACGCGGAGTACGATCGAGTGTACAGTGAA
MT451525.1 Severe acute respiratory syndrome coronavirus 2 isolate SARS-CoV-2/human/AUS/VIC754/2020 ORF1ab	TTCACCGAGGCCACGCGGAGTACGATCGAGTGTACAGTGAA
MT451529.1 Severe acute respiratory syndrome coronavirus 2 isolate SARS-CoV-2/human/AUS/VIC762/2020 ORF1ab	TTCACCGAGGCCACGCGGAGTACGATCGAGTGTACAGTGAA
MT451531.1 Severe acute respiratory syndrome coronavirus 2 isolate SARS-CoV-2/human/AUS/VIC764/2020 ORF1ab	TTCACCGAGGCCACGCGGAGTACGATCGAGTGTACAGTGAA
MT451532.1 Severe acute respiratory syndrome coronavirus 2 isolate SARS-CoV-2/human/AUS/VIC765/2020 ORF1ab	TTCACCGAGGCCACGCGGAGTACGATCGAGTGTACAGTGAA
MT451535.1 Severe acute respiratory syndrome coronavirus 2 isolate SARS-CoV-2/human/AUS/VIC768/2020 ORF1ab	TTCACCGAGGCCACGCGGAGTACGATCGAGTGTACAGTGAA
MT451536.1 Severe acute respiratory syndrome coronavirus 2 isolate SARS-CoV-2/human/AUS/VIC769/2020 ORF1ab	TTCACCGAGGCCACGCGGAGTACGATCGAGTGTACAGTGAA
MT451537.1 Severe acute respiratory syndrome coronavirus 2 isolate SARS-CoV-2/human/AUS/VIC770/2020 ORF1ab	TTCACCGAGGCCACGCGGAGTACGATCGAGTGTACAGTGAA
MT451540.1 Severe acute respiratory syndrome coronavirus 2 isolate SARS-CoV-2/human/AUS/VIC773/2020 ORF1ab	TTCACCGAGGCCACGCGGAGTACGATCGAGTGTACAGTGAA
MT451546.1 Severe acute respiratory syndrome coronavirus 2 isolate SARS-CoV-2/human/AUS/VIC782/2020 ORF1ab	TTCACCGAGGCCACGCGGAGTACGATCGAGTGTACAGTGAA
MT451547.1 Severe acute respiratory syndrome coronavirus 2 isolate SARS-CoV-2/human/AUS/VIC783/2020 ORF1ab	TTCACCGAGGCCACGCGGAGTACGATCGAGTGTACAGTGAA
MT451549.1 Severe acute respiratory syndrome coronavirus 2 isolate SARS-CoV-2/human/AUS/VIC785/2020, comple	TTCACCGAGGCCACGCGGAGTACGATCGAGTGTACAGTGAA
MT451552.1 Severe acute respiratory syndrome coronavirus 2 isolate SARS-CoV-2/human/AUS/VIC788/2020 ORF1ab	TTCACCGAGGCCACGCGGAGTACGATCGAGTGTACAGTGAA
MT451559.1 Severe acute respiratory syndrome coronavirus 2 isolate SARS-CoV-2/human/AUS/VIC796/2020 ORF1ab	TTCACCGAGGCCACGCGGAGTACGATCGAGTGTACAGTGAA
MT451564.1 Severe acute respiratory syndrome coronavirus 2 isolate SARS-CoV-2/human/AUS/VIC802/2020 ORF1ab	TTCACCGAGGCCACGCGGAGTACGATCGAGTGTACAGTGAA
MT451567.1 Severe acute respiratory syndrome coronavirus 2 isolate SARS-CoV-2/human/AUS/VIC805/2020 ORF1ab	TTCACCGAGGCCACGCGGAGTACGATCGAGTGTACAGTGAA
MT451568.1 Severe acute respiratory syndrome coronavirus 2 isolate SARS-CoV-2/human/AUS/VIC806/2020 ORF1ab	TTCACCGAGGCCACGCGGAGTACGATCGAGTGTACAGTGAA
MT451569.1 Severe acute respiratory syndrome coronavirus 2 isolate SARS-CoV-2/human/AUS/VIC807/2020, comple	TTCACCGAGGCCACGCGGAGTACGATCGAGTGTACAGTGAA
MT451584.1 Severe acute respiratory syndrome coronavirus 2 isolate SARS-CoV-2/human/AUS/VIC836/2020, comple	TTCACCGAGGCCACGCGGAGTACGATCGAGTGTACAGTGAA
MT451585.1 Severe acute respiratory syndrome coronavirus 2 isolate SARS-CoV-2/human/AUS/VIC837/2020 ORF1ab	TTCACCGAGGCCACGCGGAGTACGATCGAGTGTACAGTGAA
MT451587.1 Severe acute respiratory syndrome coronavirus 2 isolate SARS-CoV-2/human/AUS/VIC843/2020 ORF1ab	TTCACCGAGGCCACGCGGAGTACGATCGAGTGTACAGTGAA

MT451591.1 Severe acute respiratory syndrome coronavirus 2 isolate SARS-CoV-2/human/AUS/VIC847/2020 ORF1ab	TTCACCGAGGCCACGCGGAGTACGATCGAGTGTACAGTGAA
MT451592.1 Severe acute respiratory syndrome coronavirus 2 isolate SARS-CoV-2/human/AUS/VIC848/2020, comple	TTCACCGAGGCCACGCGGAGTACGATCGAGTGTACAGTGAA
MT451594.1 Severe acute respiratory syndrome coronavirus 2 isolate SARS-CoV-2/human/AUS/VIC850/2020 ORF1ab	TTCACCGAGGCCACGCGGAGTACGATCGAGTGTACAGTGAA
MT451595.1 Severe acute respiratory syndrome coronavirus 2 isolate SARS-CoV-2/human/AUS/VIC851/2020 ORF1ab	TTCACCGAGGCCACGCGGAGTACGATCGAGTGTACAGTGAA
MT451597.1 Severe acute respiratory syndrome coronavirus 2 isolate SARS-CoV-2/human/AUS/VIC853/2020, comple	TTCACCGAGGCCACGCGGAGTACGATCGAGTGTACAGTGAA
MT451599.1 Severe acute respiratory syndrome coronavirus 2 isolate SARS-CoV-2/human/AUS/VIC856/2020, comple	TTCACCGAGGCCACGCGGAGTACGATCGAGTGTACAGTGAA
MT451601.1 Severe acute respiratory syndrome coronavirus 2 isolate SARS-CoV-2/human/AUS/VIC858/2020, comple	TTCACCGAGGCCACGCGGAGTACGATCGAGTGTACAGTGAA
MT451602.1 Severe acute respiratory syndrome coronavirus 2 isolate SARS-CoV-2/human/AUS/VIC860/2020, comple	TTCACCGAGGCCACGCGGAGTACGATCGAGTGTACAGTGAA
MT451603.1 Severe acute respiratory syndrome coronavirus 2 isolate SARS-CoV-2/human/AUS/VIC862/2020, comple	TTCACCGAGGCCACGCGGAGTACGATCGAGTGTACAGTGAA
MT451604.1 Severe acute respiratory syndrome coronavirus 2 isolate SARS-CoV-2/human/AUS/VIC863/2020, comple	TTCACCGAGGCCACGCGGAGTACGATCGAGTGTACAGTGAA
MT451607.1 Severe acute respiratory syndrome coronavirus 2 isolate SARS-CoV-2/human/AUS/VIC867/2020, comple	TTCACCGAGGCCACGCGGAGTACGATCGAGTGTACAGTGAA
MT451608.1 Severe acute respiratory syndrome coronavirus 2 isolate SARS-CoV-2/human/AUS/VIC868/2020, comple	TTCACCGAGGCCACGCGGAGTACGATCGAGTGTACAGTGAA
MT451609.1 Severe acute respiratory syndrome coronavirus 2 isolate SARS-CoV-2/human/AUS/VIC869/2020, comple	TTCACCGAGGCCACGCGGAGTACGATCGAGTGTACAGTGAA
MT451610.1 Severe acute respiratory syndrome coronavirus 2 isolate SARS-CoV-2/human/AUS/VIC870/2020 ORF1ab	TTCACCGAGGCCACGCGGAGTACGATCGAGTGTACAGTGAA
MT451611.1 Severe acute respiratory syndrome coronavirus 2 isolate SARS-CoV-2/human/AUS/VIC871/2020, comple	TTCACCGAGGCCACGCGGAGTACGATCGAGTGTACAGTGAA
MT451613.1 Severe acute respiratory syndrome coronavirus 2 isolate SARS-CoV-2/human/AUS/VIC874/2020 ORF1ab	TTCACCGAGGCCACGCGGAGTACGATCGAGTGTACAGTGAA
MT451615.1 Severe acute respiratory syndrome coronavirus 2 isolate SARS-CoV-2/human/AUS/VIC876/2020 ORF1ab	TTCACCGAGGCCACGCGGAGTACGATCGAGTGTACAGTGAA
MT451621.1 Severe acute respiratory syndrome coronavirus 2 isolate SARS-CoV-2/human/AUS/VIC886/2020 ORF1ab	TTCACCGAGGCCACGCGGAGTACGATCGAGTGTACAGTGAA
MT451625.1 Severe acute respiratory syndrome coronavirus 2 isolate SARS-CoV-2/human/AUS/VIC892/2020 ORF1ab	TTCACCGAGGCCACGCGGAGTACGATCGAGTGTACAGTGAA
MT451630.1 Severe acute respiratory syndrome coronavirus 2 isolate SARS-CoV-2/human/AUS/VIC912/2020 ORF1ab	TTCACCGAGGCCACGCGGAGTACGATCGAGTGTACAGTGAA
MT451631.1 Severe acute respiratory syndrome coronavirus 2 isolate SARS-CoV-2/human/AUS/VIC920/2020 ORF1ab	TTCACCGAGGCCACGCGGAGTACGATCGAGTGTACAGTGAA
MT451633.1 Severe acute respiratory syndrome coronavirus 2 isolate SARS-CoV-2/human/AUS/VIC928/2020 ORF1ab	TTCACCGAGGCCACGCGGAGTACGATCGAGTGTACAGTGAA
MT451636.1 Severe acute respiratory syndrome coronavirus 2 isolate SARS-CoV-2/human/AUS/VIC931/2020 ORF1ab	TTCACCGAGGCCACGCGGAGTACGATCGAGTGTACAGTGAA
MT451638.1 Severe acute respiratory syndrome coronavirus 2 isolate SARS-CoV-2/human/AUS/VIC935/2020 ORF1ab	TTCACCGAGGCCACGCGGAGTACGATCGAGTGTACAGTGAA
MT451640.1 Severe acute respiratory syndrome coronavirus 2 isolate SARS-CoV-2/human/AUS/VIC938/2020 ORF1ab	TTCACCGAGGCCACGCGGAGTACGATCGAGTGTACAGTGAA
MT451641.1 Severe acute respiratory syndrome coronavirus 2 isolate SARS-CoV-2/human/AUS/VIC942/2020, comple	TTCACCGAGGCCACGCGGAGTACGATCGAGTGTACAGTGAA
MT451642.1 Severe acute respiratory syndrome coronavirus 2 isolate SARS-CoV-2/human/AUS/VIC946/2020 ORF1ab	TTCACCGAGGCCACGCGGAGTACGATCGAGTGTACAGTGAA
MT451644.1 Severe acute respiratory syndrome coronavirus 2 isolate SARS-CoV-2/human/AUS/VIC948/2020 ORF1ab	TTCACCGAGGCCACGCGGAGTACGATCGAGTGTACAGTGAA
MT451646.1 Severe acute respiratory syndrome coronavirus 2 isolate SARS-CoV-2/human/AUS/VIC951/2020, comple	TTCACCGAGGCCACGCGGAGTACGATCGAGTGTACAGTGAA
MT451647.1 Severe acute respiratory syndrome coronavirus 2 isolate SARS-CoV-2/human/AUS/VIC953/2020 ORF1ab	TTCACCGAGGCCACGCGGAGTACGATCGAGTGTACAGTGAA
MT451650.1 Severe acute respiratory syndrome coronavirus 2 isolate SARS-CoV-2/human/AUS/VIC957/2020 ORF1ab	TTCACCGAGGCCACGCGGAGTACGATCGAGTGTACAGTGAA
MT451651.1 Severe acute respiratory syndrome coronavirus 2 isolate SARS-CoV-2/human/AUS/VIC959/2020, comple	TTCACCGAGGCCACGCGGAGTACGATCGAGTGTACAGTGAA
MT451653.1 Severe acute respiratory syndrome coronavirus 2 isolate SARS-CoV-2/human/AUS/VIC961/2020, comple	TTCACCGAGGCCACGCGGAGTACGATCGAGTGTACAGTGAA

MT451655.1 Severe acute respiratory syndrome coronavirus 2 isolate SARS-CoV-2/human/AUS/VIC964/2020, comple	TTCACCGAGGCCACGCGGAGTACGATCGAGTGTACAGTGAA
MT451659.1 Severe acute respiratory syndrome coronavirus 2 isolate SARS-CoV-2/human/AUS/VIC968/2020 ORF1ab	TTCACCGAGGCCACGCGGAGTACGATCGAGTGTACAGTGAA
MT451662.1 Severe acute respiratory syndrome coronavirus 2 isolate SARS-CoV-2/human/AUS/VIC971/2020 ORF1ab	TTCACCGAGGCCACGCGGAGTACGATCGAGTGTACAGTGAA
MT451664.1 Severe acute respiratory syndrome coronavirus 2 isolate SARS-CoV-2/human/AUS/VIC974/2020 ORF1ab	TTCACCGAGGCCACGCGGAGTACGATCGAGTGTACAGTGAA
MT451665.1 Severe acute respiratory syndrome coronavirus 2 isolate SARS-CoV-2/human/AUS/VIC975/2020 ORF1ab	TTCACCGAGGCCACGCGGAGTACGATCGAGTGTACAGTGAA
MT451666.1 Severe acute respiratory syndrome coronavirus 2 isolate SARS-CoV-2/human/AUS/VIC978/2020, comple	TTCACCGAGGCCACGCGGAGTACGATCGAGTGTACAGTGAA
MT451667.1 Severe acute respiratory syndrome coronavirus 2 isolate SARS-CoV-2/human/AUS/VIC982/2020, comple	TTCACCGAGGCCACGCGGAGTACGATCGAGTGTACAGTGAA
MT451668.1 Severe acute respiratory syndrome coronavirus 2 isolate SARS-CoV-2/human/AUS/VIC983/2020, comple	TTCACCGAGGCCACGCGGAGTACGATCGAGTGTACAGTGAA
MT451669.1 Severe acute respiratory syndrome coronavirus 2 isolate SARS-CoV-2/human/AUS/VIC984/2020 ORF1ab	TTCACCGAGGCCACGCGGAGTACGATCGAGTGTACAGTGAA
MT451670.1 Severe acute respiratory syndrome coronavirus 2 isolate SARS-CoV-2/human/AUS/VIC985/2020 ORF1ab	TTCACCGAGGCCACGCGGAGTACGATCGAGTGTACAGTGAA
MT451674.1 Severe acute respiratory syndrome coronavirus 2 isolate SARS-CoV-2/human/AUS/VIC989/2020, comple	TTCACCGAGGCCACGCGGAGTACGATCGAGTGTACAGTGAA
MT451675.1 Severe acute respiratory syndrome coronavirus 2 isolate SARS-CoV-2/human/AUS/VIC990/2020, comple	TTCACCGAGGCCACGCGGAGTACGATCGAGTGTACAGTGAA
MT451679.1 Severe acute respiratory syndrome coronavirus 2 isolate SARS-CoV-2/human/AUS/VIC994/2020 ORF1ab	TTCACCGAGGCCACGCGGAGTACGATCGAGTGTACAGTGAA
MT451680.1 Severe acute respiratory syndrome coronavirus 2 isolate SARS-CoV-2/human/AUS/VIC995/2020, comple	TTCACCGAGGCCACGCGGAGTACGATCGAGTGTACAGTGAA
MT451683.1 Severe acute respiratory syndrome coronavirus 2 isolate SARS-CoV-2/human/AUS/VIC998/2020 ORF1ab	TTCACCGAGGCCACGCGGAGTACGATCGAGTGTACAGTGAA
MT451684.1 Severe acute respiratory syndrome coronavirus 2 isolate SARS-CoV-2/human/AUS/VIC1000/2020, comp	TTCACCGAGGCCACGCGGAGTACGATCGAGTGTACAGTGAA
MT451685.1 Severe acute respiratory syndrome coronavirus 2 isolate SARS-CoV-2/human/AUS/VIC1001/2020 ORF1a	TTCACCGAGGCCACGCGGAGTACGATCGAGTGTACAGTGAA
MT451686.1 Severe acute respiratory syndrome coronavirus 2 isolate SARS-CoV-2/human/AUS/VIC1002/2020 ORF1a	TTCACCGAGGCCACGCGGAGTACGATCGAGTGTACAGTGAA
MT451690.1 Severe acute respiratory syndrome coronavirus 2 isolate SARS-CoV-2/human/AUS/VIC1008/2020 ORF1a	TTCACCGAGGCCACGCGGAGTACGATCGAGTGTACAGTGAA
MT451693.1 Severe acute respiratory syndrome coronavirus 2 isolate SARS-CoV-2/human/AUS/VIC1012/2020 ORF1a	TTCACCGAGGCCACGCGGAGTACGATCGAGTGTACAGTGAA
MT451694.1 Severe acute respiratory syndrome coronavirus 2 isolate SARS-CoV-2/human/AUS/VIC1013/2020, comp	TTCACCGAGGCCACGCGGAGTACGATCGAGTGTACAGTGAA
MT451696.1 Severe acute respiratory syndrome coronavirus 2 isolate SARS-CoV-2/human/AUS/VIC1015/2020, comp	TTCACCGAGGCCACGCGGAGTACGATCGAGTGTACAGTGAA
MT451697.1 Severe acute respiratory syndrome coronavirus 2 isolate SARS-CoV-2/human/AUS/VIC1016/2020 ORF1a	TTCACCGAGGCCACGCGGAGTACGATCGAGTGTACAGTGAA
MT451699.1 Severe acute respiratory syndrome coronavirus 2 isolate SARS-CoV-2/human/AUS/VIC1018/2020 ORF1a	TTCACCGAGGCCACGCGGAGTACGATCGAGTGTACAGTGAA
MT451700.1 Severe acute respiratory syndrome coronavirus 2 isolate SARS-CoV-2/human/AUS/VIC1020/2020 ORF1a	TTCACCGAGGCCACGCGGAGTACGATCGAGTGTACAGTGAA
MT451702.1 Severe acute respiratory syndrome coronavirus 2 isolate SARS-CoV-2/human/AUS/VIC1023/2020 ORF1a	TTCACCGAGGCCACGCGGAGTACGATCGAGTGTACAGTGAA
MT451704.1 Severe acute respiratory syndrome coronavirus 2 isolate SARS-CoV-2/human/AUS/VIC1025/2020, comp	TTCACCGAGGCCACGCGGAGTACGATCGAGTGTACAGTGAA
MT451708.1 Severe acute respiratory syndrome coronavirus 2 isolate SARS-CoV-2/human/AUS/VIC1037/2020, comp	TTCACCGAGGCCACGCGGAGTACGATCGAGTGTACAGTGAA
MT451710.1 Severe acute respiratory syndrome coronavirus 2 isolate SARS-CoV-2/human/AUS/VIC1039/2020, comp	TTCACCGAGGCCACGCGGAGTACGATCGAGTGTACAGTGAA
MT451714.1 Severe acute respiratory syndrome coronavirus 2 isolate SARS-CoV-2/human/AUS/VIC1044/2020 ORF1a	TTCACCGAGGCCACGCGGAGTACGATCGAGTGTACAGTGAA
MT451715.1 Severe acute respiratory syndrome coronavirus 2 isolate SARS-CoV-2/human/AUS/VIC1045/2020 ORF1a	TTCACCGAGGCCACGCGGAGTACGATCGAGTGTACAGTGAA
MT451716.1 Severe acute respiratory syndrome coronavirus 2 isolate SARS-CoV-2/human/AUS/VIC1046/2020, comp	TTCACCGAGGCCACGCGGAGTACGATCGAGTGTACAGTGAA
MT451722.1 Severe acute respiratory syndrome coronavirus 2 isolate SARS-CoV-2/human/AUS/VIC1055/2020 ORF1a	TTCACCGAGGCCACGCGGAGTACGATCGAGTGTACAGTGAA

MT451723.1 Severe acute respiratory syndrome coronavirus 2 isolate SARS-CoV-2/human/AUS/VIC1056/2020, comp	TTCACCGAGGCCACGCGGAGTACGATCGAGTGTACAGTGAA
MT451726.1 Severe acute respiratory syndrome coronavirus 2 isolate SARS-CoV-2/human/AUS/VIC1062/2020, comp	TTCACCGAGGCCACGCGGAGTACGATCGAGTGTACAGTGAA
MT451728.1 Severe acute respiratory syndrome coronavirus 2 isolate SARS-CoV-2/human/AUS/VIC1067/2020, comp	TTCACCGAGGCCACGCGGAGTACGATCGAGTGTACAGTGAA
MT451730.1 Severe acute respiratory syndrome coronavirus 2 isolate SARS-CoV-2/human/AUS/VIC1071/2020, comp	TTCACCGAGGCCACGCGGAGTACGATCGAGTGTACAGTGAA
MT451733.1 Severe acute respiratory syndrome coronavirus 2 isolate SARS-CoV-2/human/AUS/VIC1080/2020, comp	TTCACCGAGGCCACGCGGAGTACGATCGAGTGTACAGTGAA
MT451734.1 Severe acute respiratory syndrome coronavirus 2 isolate SARS-CoV-2/human/AUS/VIC1081/2020, comp	TTCACCGAGGCCACGCGGAGTACGATCGAGTGTACAGTGAA
MT451735.1 Severe acute respiratory syndrome coronavirus 2 isolate SARS-CoV-2/human/AUS/VIC1085/2020 ORF1a	TTCACCGAGGCCACGCGGAGTACGATCGAGTGTACAGTGAA
MT451736.1 Severe acute respiratory syndrome coronavirus 2 isolate SARS-CoV-2/human/AUS/VIC1086/2020, comp	TTCACCGAGGCCACGCGGAGTACGATCGAGTGTACAGTGAA
MT451737.1 Severe acute respiratory syndrome coronavirus 2 isolate SARS-CoV-2/human/AUS/VIC1087/2020, comp	TTCACCGAGGCCACGCGGAGTACGATCGAGTGTACAGTGAA
MT451738.1 Severe acute respiratory syndrome coronavirus 2 isolate SARS-CoV-2/human/AUS/VIC1089/2020 ORF1a	TTCACCGAGGCCACGCGGAGTACGATCGAGTGTACAGTGAA
MT451740.1 Severe acute respiratory syndrome coronavirus 2 isolate SARS-CoV-2/human/AUS/VIC1094/2020, comp	TTCACCGAGGCCACGCGGAGTACGATCGAGTGTACAGTGAA
MT451743.1 Severe acute respiratory syndrome coronavirus 2 isolate SARS-CoV-2/human/AUS/VIC1104/2020 ORF1a	TTCACCGAGGCCACGCGGAGTACGATCGAGTGTACAGTGAA
MT451744.1 Severe acute respiratory syndrome coronavirus 2 isolate SARS-CoV-2/human/AUS/VIC1105/2020 ORF1a	TTCACCGAGGCCACGCGGAGTACGATCGAGTGTACAGTGAA
MT451745.1 Severe acute respiratory syndrome coronavirus 2 isolate SARS-CoV-2/human/AUS/VIC1106/2020 ORF1a	TTCACCGAGGCCACGCGGAGTACGATCGAGTGTACAGTGAA
MT451746.1 Severe acute respiratory syndrome coronavirus 2 isolate SARS-CoV-2/human/AUS/VIC1107/2020 ORF1a	TTCACCGAGGCCACGCGGAGTACGATCGAGTGTACAGTGAA
MT451747.1 Severe acute respiratory syndrome coronavirus 2 isolate SARS-CoV-2/human/AUS/VIC1108/2020 ORF1a	TTCACCGAGGCCACGCGGAGTACGATCGAGTGTACAGTGAA
MT451748.1 Severe acute respiratory syndrome coronavirus 2 isolate SARS-CoV-2/human/AUS/VIC1109/2020, comp	TTCACCGAGGCCACGCGGAGTACGATCGAGTGTACAGTGAA
MT451749.1 Severe acute respiratory syndrome coronavirus 2 isolate SARS-CoV-2/human/AUS/VIC1110/2020, comp	TTCACCGAGGCCACGCGGAGTACGATCGAGTGTACAGTGAA
MT451751.1 Severe acute respiratory syndrome coronavirus 2 isolate SARS-CoV-2/human/AUS/VIC1112/2020, comp	TTCACCGAGGCCACGCGGAGTACGATCGAGTGTACAGTGAA
MT451752.1 Severe acute respiratory syndrome coronavirus 2 isolate SARS-CoV-2/human/AUS/VIC1113/2020 ORF1a	TTCACCGAGGCCACGCGGAGTACGATCGAGTGTACAGTGAA
MT451755.1 Severe acute respiratory syndrome coronavirus 2 isolate SARS-CoV-2/human/AUS/VIC1119/2020, comp	TTCACCGAGGCCACGCGGAGTACGATCGAGTGTACAGTGAA
MT451760.1 Severe acute respiratory syndrome coronavirus 2 isolate SARS-CoV-2/human/AUS/VIC1133/2020 ORF1a	TTCACCGAGGCCACGCGGAGTACGATCGAGTGTACAGTGAA
MT451761.1 Severe acute respiratory syndrome coronavirus 2 isolate SARS-CoV-2/human/AUS/VIC1134/2020 ORF1a	TTCACCGAGGCCACGCGGAGTACGATCGAGTGTACAGTGAA
MT451762.1 Severe acute respiratory syndrome coronavirus 2 isolate SARS-CoV-2/human/AUS/VIC1135/2020, comp	TTCACCGAGGCCACGCGGAGTACGATCGAGTGTACAGTGAA
MT451763.1 Severe acute respiratory syndrome coronavirus 2 isolate SARS-CoV-2/human/AUS/VIC1136/2020 ORF1a	TTCACCGAGGCCACGCGGAGTACGATCGAGTGTACAGTGAA
MT451765.1 Severe acute respiratory syndrome coronavirus 2 isolate SARS-CoV-2/human/AUS/VIC1138/2020 ORF1a	TTCACCGAGGCCACGCGGAGTACGATCGAGTGTACAGTGAA
MT451766.1 Severe acute respiratory syndrome coronavirus 2 isolate SARS-CoV-2/human/AUS/VIC1139/2020 ORF1a	TTCACCGAGGCCACGCGGAGTACGATCGAGTGTACAGTGAA
MT451770.1 Severe acute respiratory syndrome coronavirus 2 isolate SARS-CoV-2/human/AUS/VIC1143/2020 ORF1a	TTCACCGAGGCCACGCGGAGTACGATCGAGTGTACAGTGAA
MT451775.1 Severe acute respiratory syndrome coronavirus 2 isolate SARS-CoV-2/human/AUS/VIC1157/2020, comp	TTCACCGAGGCCACGCGGAGTACGATCGAGTGTACAGTGAA
MT451776.1 Severe acute respiratory syndrome coronavirus 2 isolate SARS-CoV-2/human/AUS/VIC1158/2020, comp	TTCACCGAGGCCACGCGGAGTACGATCGAGTGTACAGTGAA
MT451777.1 Severe acute respiratory syndrome coronavirus 2 isolate SARS-CoV-2/human/AUS/VIC1159/2020, comp	TTCACCGAGGCCACGCGGAGTACGATCGAGTGTACAGTGAA
MT451778.1 Severe acute respiratory syndrome coronavirus 2 isolate SARS-CoV-2/human/AUS/VIC1160/2020 ORF1a	TTCACCGAGGCCACGCGGAGTACGATCGAGTGTACAGTGAA
MT451779.1 Severe acute respiratory syndrome coronavirus 2 isolate SARS-CoV-2/human/AUS/VIC1161/2020, comp	TTCACCGAGGCCACGCGGAGTACGATCGAGTGTACAGTGAA

MT451780.1 Severe acute respiratory syndrome coronavirus 2 isolate SARS-CoV-2/human/AUS/VIC1162/2020 ORF1a	TTCACCGAGGCCACGCGGAGTACGATCGAGTGTACAGTGAA
MT451784.1 Severe acute respiratory syndrome coronavirus 2 isolate SARS-CoV-2/human/AUS/VIC1175/2020 ORF1a	TTCACCGAGGCCACGCGGAGTACGATCGAGTGTACAGTGAA
MT451786.1 Severe acute respiratory syndrome coronavirus 2 isolate SARS-CoV-2/human/AUS/VIC1178/2020, comp	TTCACCGAGGCCACGCGGAGTACGATCGAGTGTACAGTGAA
MT451788.1 Severe acute respiratory syndrome coronavirus 2 isolate SARS-CoV-2/human/AUS/VIC1181/2020 ORF1a	TTCACCGAGGCCACGCGGAGTACGATCGAGTGTACAGTGAA
MT451795.1 Severe acute respiratory syndrome coronavirus 2 isolate SARS-CoV-2/human/AUS/VIC1198/2020 ORF1a	TTCACCGAGGCCACGCGGAGTACGATCGAGTGTACAGTGAA
MT451796.1 Severe acute respiratory syndrome coronavirus 2 isolate SARS-CoV-2/human/AUS/VIC1199/2020 ORF1a	TTCACCGAGGCCACGCGGAGTACGATCGAGTGTACAGTGAA
MT451797.1 Severe acute respiratory syndrome coronavirus 2 isolate SARS-CoV-2/human/AUS/VIC1200/2020 ORF1a	TTCACCGAGGCCACGCGGAGTACGATCGAGTGTACAGTGAA
MT451799.1 Severe acute respiratory syndrome coronavirus 2 isolate SARS-CoV-2/human/AUS/VIC1208/2020 ORF1a	TTCACCGAGGCCACGCGGAGTACGATCGAGTGTACAGTGAA
MT451805.1 Severe acute respiratory syndrome coronavirus 2 isolate SARS-CoV-2/human/AUS/VIC1214/2020 ORF1a	TTCACCGAGGCCACGCGGAGTACGATCGAGTGTACAGTGAA
MT451809.1 Severe acute respiratory syndrome coronavirus 2 isolate SARS-CoV-2/human/AUS/VIC1219/2020 ORF1a	TTCACCGAGGCCACGCGGAGTACGATCGAGTGTACAGTGAA
MT451812.1 Severe acute respiratory syndrome coronavirus 2 isolate SARS-CoV-2/human/AUS/VIC1222/2020 ORF1a	TTCACCGAGGCCACGCGGAGTACGATCGAGTGTACAGTGAA
MT451813.1 Severe acute respiratory syndrome coronavirus 2 isolate SARS-CoV-2/human/AUS/VIC1224/2020 ORF1a	TTCACCGAGGCCACGCGGAGTACGATCGAGTGTACAGTGAA
MT451815.1 Severe acute respiratory syndrome coronavirus 2 isolate SARS-CoV-2/human/AUS/VIC1230/2020 ORF1a	TTCACCGAGGCCACGCGGAGTACGATCGAGTGTACAGTGAA
MT451817.1 Severe acute respiratory syndrome coronavirus 2 isolate SARS-CoV-2/human/AUS/VIC1234/2020 ORF1a	TTCACCGAGGCCACGCGGAGTACGATCGAGTGTACAGTGAA
MT451818.1 Severe acute respiratory syndrome coronavirus 2 isolate SARS-CoV-2/human/AUS/VIC1235/2020 ORF1a	TTCACCGAGGCCACGCGGAGTACGATCGAGTGTACAGTGAA
MT451820.1 Severe acute respiratory syndrome coronavirus 2 isolate SARS-CoV-2/human/AUS/VIC1237/2020 ORF1a	TTCACCGAGGCCACGCGGAGTACGATCGAGTGTACAGTGAA
MT451824.1 Severe acute respiratory syndrome coronavirus 2 isolate SARS-CoV-2/human/AUS/VIC1247/2020 ORF1a	TTCACCGAGGCCACGCGGAGTACGATCGAGTGTACAGTGAA
MT451825.1 Severe acute respiratory syndrome coronavirus 2 isolate SARS-CoV-2/human/AUS/VIC1248/2020 ORF1a	TTCACCGAGGCCACGCGGAGTACGATCGAGTGTACAGTGAA
MT451831.1 Severe acute respiratory syndrome coronavirus 2 isolate SARS-CoV-2/human/AUS/VIC1256/2020 ORF1a	TTCACCGAGGCCACGCGGAGTACGATCGAGTGTACAGTGAA
MT451837.1 Severe acute respiratory syndrome coronavirus 2 isolate SARS-CoV-2/human/AUS/VIC1267/2020 ORF1a	TTCACCGAGGCCACGCGGAGTACGATCGAGTGTACAGTGAA
MT451874.1 Severe acute respiratory syndrome coronavirus 2 isolate SARS-CoV-2/human/IND/GBRC10/2020, compl	TTCACCGAGGCCACGCGGAGTACGATCGAGTGTACAGTGAA
MT451875.1 Severe acute respiratory syndrome coronavirus 2 isolate SARS-CoV-2/human/IND/GBRC11/2020 ORF1ab	TTCACCGAGGCCACACGGAGTACGATCGAGTGTACAGTGAA
MT451876.1 Severe acute respiratory syndrome coronavirus 2 isolate SARS-CoV-2/human/IND/GBRC12/2020, compl	TTCACCGAGGCCACGCGGAGTACGATCGAGTGTACAGTGAA
MT451877.1 Severe acute respiratory syndrome coronavirus 2 isolate SARS-CoV-2/human/IND/GBRC13/2020, compl	TTCACCGAGGCCACACGGAGTACGATCGAGTGTACAGTGAA
MT451878.1 Severe acute respiratory syndrome coronavirus 2 isolate SARS-CoV-2/human/IND/GBRC14/2020, compl	TTCACCGAGGCCACGCGGAGTACGATCGAGTGTACAGTGAA
MT451879.1 Severe acute respiratory syndrome coronavirus 2 isolate SARS-CoV-2/human/IND/GBRC15/2020 ORF1ab	TTCACCGAGGCCACACGGAGTACGATCGAGTGTACAGTGAA
MT451880.1 Severe acute respiratory syndrome coronavirus 2 isolate SARS-CoV-2/human/IND/GBRC16/2020, compl	TTCACCGAGGCCACGCGGAGTACGATCGAGTGTACAGTGAA
MT451881.1 Severe acute respiratory syndrome coronavirus 2 isolate SARS-CoV-2/human/IND/GBRC17a/2020, comp	TTCACCGAGGCCACGCGGAGTACGATCGAGTGTACAGTGAA
MT451882.1 Severe acute respiratory syndrome coronavirus 2 isolate SARS-CoV-2/human/IND/GBRC17b/2020, comp	TTCACCGAGGCCACGCGGAGTACGATCGAGTGTACAGTGAA
MT451883.1 Severe acute respiratory syndrome coronavirus 2 isolate SARS-CoV-2/human/IND/GBRC18/2020, compl	TTCACCGAGGCCACGCGGAGTACGATCGAGTGTACAGTGAA
MT451884.1 Severe acute respiratory syndrome coronavirus 2 isolate SARS-CoV-2/human/IND/GBRC19/2020, compl	TTCACCGAGGCCACGCGGAGTACGATCGAGTGTACAGTGAA
MT451885.1 Severe acute respiratory syndrome coronavirus 2 isolate SARS-CoV-2/human/IND/GBRC20/2020, compl	TTCACCGAGGCCACGCGGAGTACGATCGAGTGTACAGTGAA
MT451886.1 Severe acute respiratory syndrome coronavirus 2 isolate SARS-CoV-2/human/IND/GBRC21/2020, compl	TTCACCGAGGCCACGCGGAGTACGATCGAGTGTACAGTGAA

MT451887.1 Severe acute respiratory syndrome coronavirus 2 isolate SARS-CoV-2/human/IND/GBRC22/2020, compl	TTCACCGAGGCCACGCGGAGTACGATCGAGTGTACAGTGAA
MT451888.1 Severe acute respiratory syndrome coronavirus 2 isolate SARS-CoV-2/human/IND/GBRC23/2020, compl	TTCACCGAGGCCACGCGGAGTACGATCGAGTGTACAGTGAA
MT451889.1 Severe acute respiratory syndrome coronavirus 2 isolate SARS-CoV-2/human/IND/GBRC24a/2020, comp	TTCACCGAGGCCACGCGGAGTACGATCGAGTGTACAGTGAA
MT451890.1 Severe acute respiratory syndrome coronavirus 2 isolate SARS-CoV-2/human/IND/GBRC24b/2020, comp	TTCACCGAGGCCACGCGGAGTACGATCGAGTGTACAGTGAA
MT452574.1 Severe acute respiratory syndrome coronavirus 2 isolate SAR-CoV-2/human/USA/NY-QDX-00000001/20	TTCACCGAGGCCACGCGGAGTACGATCGAGTGTACAGTGAA
MT452575.1 Severe acute respiratory syndrome coronavirus 2 isolate SAR-CoV-2/human/USA/WA-QDX-00000002/20	TTCACCGAGGCCACGCGGAGTACGATCGAGTGTACAGTGAA
MT452576.1 Severe acute respiratory syndrome coronavirus 2 isolate SAR-CoV-2/human/USA/CA-QDX-00000003/20	TTCACCGAGGCCACGCGGAGTACGATCGAGTGTACAGTGAA
MT457390.1 Severe acute respiratory syndrome coronavirus 2 isolate SARS-CoV-2/mink/NED/NB01_01KS/2020, com	TTCACCGAGGCCACGCGGAGTACGATCGAGTGTACAGTGAA
MT457392.1 Severe acute respiratory syndrome coronavirus 2 isolate SARS-CoV-2/mink/NED/NB01_03KS/2020, com	TTCACCGAGGCCACGCGGAGTACGATCGAGTGTACAGTGAA
MT457393.1 Severe acute respiratory syndrome coronavirus 2 isolate SARS-CoV-2/mink/NED/NB01_04KS/2020, com	TTCACCGAGGCCACGCGGAGTACGATCGAGTGTACAGTGAA
MT457394.1 Severe acute respiratory syndrome coronavirus 2 isolate SARS-CoV-2/mink/NED/NB02_03KS/2020, com	TTCACCGAGGCCACGCGGAGTACGATCGAGTGTACAGTGAA
MT457395.1 Severe acute respiratory syndrome coronavirus 2 isolate SARS-CoV-2/mink/NED/NB02_06KS/2020, com	TTCACCGAGGCCACGCGGAGTACGATCGAGTGTACAGTGAA
MT457396.1 Severe acute respiratory syndrome coronavirus 2 isolate SARS-CoV-2/mink/NED/NB02_07KS/2020, com	TTCACCGAGGCCACGCGGAGTACGATCGAGTGTACAGTGAA
MT457397.1 Severe acute respiratory syndrome coronavirus 2 isolate SARS-CoV-2/mink/NED/NB02_13KS/2020, com	TTCACCGAGGCCACGCGGAGTACGATCGAGTGTACAGTGAA
MT457398.1 Severe acute respiratory syndrome coronavirus 2 isolate SARS-CoV-2/mink/NED/NB02_index/2020, com	TTCACCGAGGCCACGCGGAGTACGATCGAGTGTACAGTGAA
MT457399.1 Severe acute respiratory syndrome coronavirus 2 isolate SARS-CoV-2/mink/NED/NB02_16RS/2020, com	TTCACCGAGGCCACGCGGAGTACGATCGAGTGTACAGTGAA
MT457400.1 Severe acute respiratory syndrome coronavirus 2 isolate SARS-CoV-2/mink/NED/NB03_index/2020, com	TTCACCGAGGCCACGCGGAGTACGATCGAGTGTACAGTGAA
MT457401.1 Severe acute respiratory syndrome coronavirus 2 isolate SARS-CoV-2/mink/NED/NB04_index/2020, com	TTCACCGAGGCCACGCGGAGTACGATCGAGTGTACAGTGAA
MT459832.1 Severe acute respiratory syndrome coronavirus 2 isolate SARS-CoV-2/human/GRC/34_36284/2020, com	TTCACCGAGGCCACGCGGAGTACGATCGAGTGTACAGTGAA
MT459833.1 Severe acute respiratory syndrome coronavirus 2 isolate SARS-CoV-2/human/GRC/43_35679/2020, com	TTCACCGAGGCCACGCGGAGTACGATCGAGTGTACAGTGAA
MT459834.1 Severe acute respiratory syndrome coronavirus 2 isolate SARS-CoV-2/human/GRC/50_36277/2020, com	TTCACCGAGGCCACGCGGAGTACGATCGAGTGTACAGTGAA
MT459835.1 Severe acute respiratory syndrome coronavirus 2 isolate SARS-CoV-2/human/GRC/55_36015/2020, com	TTCACCGAGGCCACGCGGAGTACGATCGAGTGTACAGTGAA
MT459836.1 Severe acute respiratory syndrome coronavirus 2 isolate SARS-CoV-2/human/GRC/152_33705/2020, co	TTCACCGAGGCCACGCGGAGTACGATCGAGTGTACAGTGAA
MT459837.1 Severe acute respiratory syndrome coronavirus 2 isolate SARS-CoV-2/human/GRC/35_36913/2020, com	TTCACCGAGGCCACGCGGAGTACGATCGAGTGTACAGTGAA
MT459838.1 Severe acute respiratory syndrome coronavirus 2 isolate SARS-CoV-2/human/GRC/36_36859/2020, com	TTCACCGAGGCCACGCGGAGTACGATCGAGTGTACAGTGAA
MT459839.1 Severe acute respiratory syndrome coronavirus 2 isolate SARS-CoV-2/human/GRC/37_35684/2020, com	TTCACCGAGGCCACGCGGAGTACGATCGAGTGTACAGTGAA
MT459840.1 Severe acute respiratory syndrome coronavirus 2 isolate SARS-CoV-2/human/GRC/38_36178/2020, com	TTCACCGAGGCCACGCGGAGTACGATCGAGTGTACAGTGAA
MT459841.1 Severe acute respiratory syndrome coronavirus 2 isolate SARS-CoV-2/human/GRC/39_36914/2020, com	TTCACCGAGGCCACGCGGAGTACGATCGAGTGTACAGTGAA
MT459842.1 Severe acute respiratory syndrome coronavirus 2 isolate SARS-CoV-2/human/GRC/44_36073/2020, com	TTCACCGAGGCCACGCGGAGTACGATCGAGTGTACAGTGAA
MT459843.1 Severe acute respiratory syndrome coronavirus 2 isolate SARS-CoV-2/human/GRC/46_36230/2020, com	TTCACCGAGGCCACGCGGAGTACGATCGAGTGTACAGTGAA
MT459844.1 Severe acute respiratory syndrome coronavirus 2 isolate SARS-CoV-2/human/GRC/47_36060/2020, com	TTCACCGAGGCCACGCGGAGTACGATCGAGTGTACAGTGAA
MT459845.1 Severe acute respiratory syndrome coronavirus 2 isolate SARS-CoV-2/human/GRC/48_35910/2020, com	TTCACCGAGGCCACGCGGAGTACGATCGAGTGTACAGTGAA
MT459846.1 Severe acute respiratory syndrome coronavirus 2 isolate SARS-CoV-2/human/GRC/51_36518/2020, com	TTCACCGAGGCCACGCGGAGTACGATCGAGTGTACAGTGAA

MT459847.1 Severe acute respiratory syndrome coronavirus 2 isolate SARS-CoV-2/human/GRC/53_38017/2020, com	TTCACCGAGGCCACGCGGAGTACGATCGAGTGTACAGTGAA
MT459848.1 Severe acute respiratory syndrome coronavirus 2 isolate SARS-CoV-2/human/GRC/54_37163/2020, com	TTCACCGAGGCCACGCGGAGTACGATCGAGTGTACAGTGAA
MT459849.1 Severe acute respiratory syndrome coronavirus 2 isolate SARS-CoV-2/human/GRC/133_31929/2020, co	TTCACCGAGGCCACGCGGAGTACGATCGAGTGTACAGTGAA
MT459850.1 Severe acute respiratory syndrome coronavirus 2 isolate SARS-CoV-2/human/GRC/134_32124/2020, co	TTCACCGAGGCCACGCGGAGTACGATCGAGTGTACAGTGAA
MT459851.1 Severe acute respiratory syndrome coronavirus 2 isolate SARS-CoV-2/human/GRC/135_32174/2020, co	TTCACCGAGGCCACGCGGAGTACGATCGAGTGTACAGTGAA
MT459852.1 Severe acute respiratory syndrome coronavirus 2 isolate SARS-CoV-2/human/GRC/136_32338/2020, co	TTCACCGAGGCCACGCGGAGTACGATCGAGTGTACAGTGAA
MT459853.1 Severe acute respiratory syndrome coronavirus 2 isolate SARS-CoV-2/human/GRC/137_32340/2020, co	TTCACCGAGGCCACGCGGAGTACGATCGAGTGTACAGTGAA
MT459854.1 Severe acute respiratory syndrome coronavirus 2 isolate SARS-CoV-2/human/GRC/138_32343/2020, co	TTCACCGAGGCCACGCGGAGTACGATCGAGTGTACAGTGAA
MT459855.1 Severe acute respiratory syndrome coronavirus 2 isolate SARS-CoV-2/human/GRC/139_32716/2020, co	TTCACCGAGGCCACGCGGAGTACGATCGAGTGTACAGTGAA
MT459856.1 Severe acute respiratory syndrome coronavirus 2 isolate SARS-CoV-2/human/GRC/140_32855/2020, co	TTCACCGAGGCCACGCGGAGTACGATCGAGTGTACAGTGAA
MT459857.1 Severe acute respiratory syndrome coronavirus 2 isolate SARS-CoV-2/human/GRC/141_32983/2020, co	TTCACCGAGGCCACGCGGAGTACGATCGAGTGTACAGTGAA
MT459858.1 Severe acute respiratory syndrome coronavirus 2 isolate SARS-CoV-2/human/GRC/142_33859/2020, co	TTCACCGAGGCCACGCGGAGTACGATCGAGTGTACAGTGAA
MT459859.1 Severe acute respiratory syndrome coronavirus 2 isolate SARS-CoV-2/human/GRC/143_34652/2020, co	TTCACCGAGGCCACGCGGAGTACGATCGAGTGTACAGTGAA
MT459860.1 Severe acute respiratory syndrome coronavirus 2 isolate SARS-CoV-2/human/GRC/144_34723/2020, co	TTCACCGAGGCCACGCGGAGTACGATCGAGTGTACAGTGAA
MT459861.1 Severe acute respiratory syndrome coronavirus 2 isolate SARS-CoV-2/human/GRC/145_34726/2020, co	TTCACCGAGGCCACGCGGAGTACGATCGAGTGTACAGTGAA
MT459862.1 Severe acute respiratory syndrome coronavirus 2 isolate SARS-CoV-2/human/GRC/146_35473/2020, co	TTCACCGAGGCCACGCGGAGTACGATCGAGTGTACAGTGAA
MT459863.1 Severe acute respiratory syndrome coronavirus 2 isolate SARS-CoV-2/human/GRC/148_31943/2020, co	TTCACCGAGGCCACGCGGAGTACGATCGAGTGTACAGTGAA
MT459864.1 Severe acute respiratory syndrome coronavirus 2 isolate SARS-CoV-2/human/GRC/149_32357/2020, co	TTCACCGAGGCCACGCGGAGTACGATCGAGTGTACAGTGAA
MT459865.1 Severe acute respiratory syndrome coronavirus 2 isolate SARS-CoV-2/human/GRC/150_32764/2020, co	TTCACCGAGGCCACGCGGAGTACGATCGAGTGTACAGTGAA
MT459866.1 Severe acute respiratory syndrome coronavirus 2 isolate SARS-CoV-2/human/GRC/151_33690/2020, co	TTCACCGAGGCCACGCGGAGTACGATCGAGTGTACAGTGAA
MT459867.1 Severe acute respiratory syndrome coronavirus 2 isolate SARS-CoV-2/human/GRC/154_35632/2020, co	TTCACCGAGGCCACGCGGAGTACGATCGAGTGTACAGTGAA
MT459868.1 Severe acute respiratory syndrome coronavirus 2 isolate SARS-CoV-2/human/GRC/155_35480/2020, co	TTCACCGAGGCCACGCGGAGTACGATCGAGTGTACAGTGAA
MT459869.1 Severe acute respiratory syndrome coronavirus 2 isolate SARS-CoV-2/human/GRC/156_34721/2020, co	TTCACCGAGGCCACGCGGAGTACGATCGAGTGTACAGTGAA
MT459870.1 Severe acute respiratory syndrome coronavirus 2 isolate SARS-CoV-2/human/GRC/202_32984/2020, co	TTCACCGAGGCCACGCGGAGTACGATCGAGTGTACAGTGAA
MT459871.1 Severe acute respiratory syndrome coronavirus 2 isolate SARS-CoV-2/human/GRC/203_33026/2020, co	TTCACCGAGGCCACGCGGAGTACGATCGAGTGTACAGTGAA
MT459872.1 Severe acute respiratory syndrome coronavirus 2 isolate SARS-CoV-2/human/GRC/204_33112/2020, co	TTCACCGAGGCCACGCGGAGTACGATCGAGTGTACAGTGAA
MT459873.1 Severe acute respiratory syndrome coronavirus 2 isolate SARS-CoV-2/human/GRC/205_33490/2020, co	TTCACCGAGGCCACGCGGAGTACGATCGAGTGTACAGTGAA
MT459874.1 Severe acute respiratory syndrome coronavirus 2 isolate SARS-CoV-2/human/GRC/206_33492/2020, co	TTCACCGAGGCCACGCGGAGTACGATCGAGTGTACAGTGAA
MT459875.1 Severe acute respiratory syndrome coronavirus 2 isolate SARS-CoV-2/human/GRC/207_33548/2020, co	TTCACCGAGGCCACGCGGAGTACGATCGAGTGTACAGTGAA
MT459876.1 Severe acute respiratory syndrome coronavirus 2 isolate SARS-CoV-2/human/GRC/208_33922/2020, co	TTCACCGAGGCCACGCGGAGTACGATCGAGTGTACAGTGAA
MT459877.1 Severe acute respiratory syndrome coronavirus 2 isolate SARS-CoV-2/human/GRC/209_33926/2020, co	TTCACCGAGGCCACGCGGAGTACGATCGAGTGTACAGTGAA
MT459878.1 Severe acute respiratory syndrome coronavirus 2 isolate SARS-CoV-2/human/GRC/210_33942/2020, co	TTCACCGAGGCCACGCGGAGTACGATCGAGTGTACAGTGAA
MT459879.1 Severe acute respiratory syndrome coronavirus 2 isolate SARS-CoV-2/human/GRC/211_34005/2020, co	TTCACCGAGGCCACGCGGAGTACGATCGAGTGTACAGTGAA

MT459880.1 Severe acute respiratory syndrome coronavirus 2 isolate SARS-CoV-2/human/GRC/212_34023/2020, cor	TTCACCGAGGCCACGCGGAGTACGATCGAGTGTACAGTGAA
MT459881.1 Severe acute respiratory syndrome coronavirus 2 isolate SARS-CoV-2/human/GRC/213_34051/2020, cor	TTCACCGAGGCCACGCGGAGTACGATCGAGTGTACAGTGAA
MT459882.1 Severe acute respiratory syndrome coronavirus 2 isolate SARS-CoV-2/human/GRC/214_34505/2020, cor	TTCACCGAGGCCACGCGGAGTACGATCGAGTGTACAGTGAA
MT459883.1 Severe acute respiratory syndrome coronavirus 2 isolate SARS-CoV-2/human/GRC/215_34644/2020, cor	TTCACCGAGGCCACGCGGAGTACGATCGAGTGTACAGTGAA
MT459884.1 Severe acute respiratory syndrome coronavirus 2 isolate SARS-CoV-2/human/GRC/216_34732/2020, cor	TTCACCGAGGCCACGCGGAGTACGATCGAGTGTACAGTGAA
MT459885.1 Severe acute respiratory syndrome coronavirus 2 isolate SARS-CoV-2/human/GRC/217_34885/2020, cor	TTCACCGAGGCCACGCGGAGTACGATCGAGTGTACAGTGAA
MT459886.1 Severe acute respiratory syndrome coronavirus 2 isolate SARS-CoV-2/human/GRC/218_35009/2020, cor	TTCACCGAGGCCACGCGGAGTACGATCGAGTGTACAGTGAA
MT459887.1 Severe acute respiratory syndrome coronavirus 2 isolate SARS-CoV-2/human/GRC/219_35198/2020, cor	TTCACCGAGGCCACGCGGAGTACGATCGAGTGTACAGTGAA
MT459888.1 Severe acute respiratory syndrome coronavirus 2 isolate SARS-CoV-2/human/GRC/220_35357/2020, cor	TTCACCGAGGCCACGCGGAGTACGATCGAGTGTACAGTGAA
MT459889.1 Severe acute respiratory syndrome coronavirus 2 isolate SARS-CoV-2/human/GRC/221_33038/2020, cor	TTCACCGAGGCCACGCGGAGTACGATCGAGTGTACAGTGAA
MT459890.1 Severe acute respiratory syndrome coronavirus 2 isolate SARS-CoV-2/human/GRC/222_33921/2020, cor	TTCACCGAGGCCACGCGGAGTACGATCGAGTGTACAGTGAA
MT459891.1 Severe acute respiratory syndrome coronavirus 2 isolate SARS-CoV-2/human/GRC/223_34904/2020, cor	TTCACCGAGGCCACGCGGAGTACGATCGAGTGTACAGTGAA
MT459892.1 Severe acute respiratory syndrome coronavirus 2 isolate SARS-CoV-2/human/GRC/224_35200/2020, cor	TTCACCGAGGCCACGCGGAGTACGATCGAGTGTACAGTGAA
MT459893.1 Severe acute respiratory syndrome coronavirus 2 isolate SARS-CoV-2/human/GRC/225_35203/2020, cor	TTCACCGAGGCCACGCGGAGTACGATCGAGTGTACAGTGAA
MT459894.1 Severe acute respiratory syndrome coronavirus 2 isolate SARS-CoV-2/human/GRC/226_35576/2020, cor	TTCACCGAGGCCACGCGGAGTACGATCGAGTGTACAGTGAA
MT459895.1 Severe acute respiratory syndrome coronavirus 2 isolate SARS-CoV-2/human/GRC/227_35969/2020, cor	TTCACCGAGGCCACGCGGAGTACGATCGAGTGTACAGTGAA
MT459896.1 Severe acute respiratory syndrome coronavirus 2 isolate SARS-CoV-2/human/GRC/33_36910/2020, com	TTCACCGAGGCCACGCGGAGTACGATCGAGTGTACAGTGAA
MT459897.1 Severe acute respiratory syndrome coronavirus 2 isolate SARS-CoV-2/human/GRC/234_31670/2020, cor	TTCACCGAGGCCACGCGGAGTACGATCGAGTGTACAGTGAA
MT459898.1 Severe acute respiratory syndrome coronavirus 2 isolate SARS-CoV-2/human/GRC/235_31783/2020, cor	TTCACCGAGGCCACGCGGAGTACGATCGAGTGTACAGTGAA
MT459899.1 Severe acute respiratory syndrome coronavirus 2 isolate SARS-CoV-2/human/GRC/236_31815/2020, cor	TTCACCGAGGCCACGCGGAGTACGATCGAGTGTACAGTGAA
MT459900.1 Severe acute respiratory syndrome coronavirus 2 isolate SARS-CoV-2/human/GRC/237_31890/2020, cor	TTCACCGAGGCCACGCGGAGTACGATCGAGTGTACAGTGAA
MT459901.1 Severe acute respiratory syndrome coronavirus 2 isolate SARS-CoV-2/human/GRC/238_31927/2020, cor	TTCACCGAGGCCACGCGGAGTACGATCGAGTGTACAGTGAA
MT459902.1 Severe acute respiratory syndrome coronavirus 2 isolate SARS-CoV-2/human/GRC/239_32178/2020, cor	TTCACCGAGGCCACGCGGAGTACGATCGAGTGTACAGTGAA
MT459904.1 Severe acute respiratory syndrome coronavirus 2 isolate SARS-CoV-2/human/GRC/41_36861/2020, com	TTCACCGAGGCCACGCGGAGTACGATCGAGTGTACAGTGAA
MT459905.1 Severe acute respiratory syndrome coronavirus 2 isolate SARS-CoV-2/human/GRC/42_36236/2020, com	TTCACCGAGGCCACGCGGAGTACGATCGAGTGTACAGTGAA
MT459906.1 Severe acute respiratory syndrome coronavirus 2 isolate SARS-CoV-2/human/GRC/45_35378/2020, com	TTCACCGAGGCCACGCGGAGTACGATCGAGTGTACAGTGAA
MT459907.1 Severe acute respiratory syndrome coronavirus 2 isolate SARS-CoV-2/human/GRC/247_32207/2020, cor	TTCACCGAGGCCACGCGGAGTACGATCGAGTGTACAGTGAA
MT459908.1 Severe acute respiratory syndrome coronavirus 2 isolate SARS-CoV-2/human/GRC/248_32261/2020, cor	TTCACCGAGGCCACGCGGAGTACGATCGAGTGTACAGTGAA
MT459909.1 Severe acute respiratory syndrome coronavirus 2 isolate SARS-CoV-2/human/GRC/250_32267/2020, cor	TTCACCGAGGCCACGCGGAGTACGATCGAGTGTACAGTGAA
MT459910.1 Severe acute respiratory syndrome coronavirus 2 isolate SARS-CoV-2/human/GRC/251_32400/2020, cor	TTCACCGAGGCCACGCGGAGTACGATCGAGTGTACAGTGAA
MT459911.1 Severe acute respiratory syndrome coronavirus 2 isolate SARS-CoV-2/human/GRC/153_33892/2020, cor	TTCACCGAGGCCACGCGGAGTACGATCGAGTGTACAGTGAA
MT459912.1 Severe acute respiratory syndrome coronavirus 2 isolate SARS-CoV-2/human/GRC/254_32422/2020, cor	TTCACCGAGGCCACGCGGAGTACGATCGAGTGTACAGTGAA
MT459913.1 Severe acute respiratory syndrome coronavirus 2 isolate SARS-CoV-2/human/GRC/255_32525/2020, cor	TTCACCGAGGCCACGCGGAGTACGATCGAGTGTACAGTGAA

MT459914.1 Severe acute respiratory syndrome coronavirus 2 isolate SARS-CoV-2/human/GRC/259_31928/2020, cor	TTCACCGAGGCCACGCGGAGTACGATCGAGTGTACAGTGAA
MT459915.1 Severe acute respiratory syndrome coronavirus 2 isolate SARS-CoV-2/human/GRC/243_32202/2020, cor	TTCACCGAGGCCACGCGGAGTACGATCGAGTGTACAGTGAA
MT459916.1 Severe acute respiratory syndrome coronavirus 2 isolate SARS-CoV-2/human/GRC/244_32204/2020, cor	TTCACCGAGGCCACGCGGAGTACGATCGAGTGTACAGTGAA
MT459917.1 Severe acute respiratory syndrome coronavirus 2 isolate SARS-CoV-2/human/GRC/246_32206/2020, cor	TTCACCGAGGCCACGCGGAGTACGATCGAGTGTACAGTGAA
MT459918.1 Severe acute respiratory syndrome coronavirus 2 isolate SARS-CoV-2/human/GRC/260_32216/2020 ORI	TTCACCGAGGCCACGCGGAGTACGATCGAGTGTACAGTGAA
MT459919.1 Severe acute respiratory syndrome coronavirus 2 isolate SARS-CoV-2/human/GRC/261_32221/2020, cor	TTCACCGAGGCCACGCGGAGTACGATCGAGTGTACAGTGAA
MT459920.1 Severe acute respiratory syndrome coronavirus 2 isolate SARS-CoV-2/human/GRC/262_32443/2020, cor	TTCACCGAGGCCACGCGGAGTACGATCGAGTGTACAGTGAA
MT459921.1 Severe acute respiratory syndrome coronavirus 2 isolate SARS-CoV-2/human/GRC/263_32477/2020, cor	TTCACCGAGGCCACGCGGAGTACGATCGAGTGTACAGTGAA
MT459922.1 Severe acute respiratory syndrome coronavirus 2 isolate SARS-CoV-2/human/GRC/264_32497/2020, cor	TTCACCGAGGCCACGCGGAGTACGATCGAGTGTACAGTGAA
MT459923.1 Severe acute respiratory syndrome coronavirus 2 isolate SARS-CoV-2/human/GRC/49_36716/2020, com	TTCACCGAGGCCACGCGGAGTACGATCGAGTGTACAGTGAA
MT459924.1 Severe acute respiratory syndrome coronavirus 2 isolate SARS-CoV-2/human/GRC/52_37266/2020, com	TTCACCGAGGCCACGCGGAGTACGATCGAGTGTACAGTGAA
MT459925.1 Severe acute respiratory syndrome coronavirus 2 isolate SARS-CoV-2/human/GRC/56_37161/2020, com	TTCACCGAGGCCACGCGGAGTACGATCGAGTGTACAGTGAA
MT459979.1 Severe acute respiratory syndrome coronavirus 2 isolate SARS-CoV-2/human/SRB/Novi Pazar-363/2020,	TTCACCGAGGCCACGCGGAGTACGATCGAGTGTACAGTGAA
MT459985.1 Severe acute respiratory syndrome coronavirus 2 isolate SARS-CoV-2/human/GUM/GU-NHG-01/2020, c	TTCACCGAGGCCACGCGGAGTACGATCGAGTGTACAGTGAA
MT459986.1 Severe acute respiratory syndrome coronavirus 2 isolate SARS-CoV-2/human/GUM/GU-NHG-02/2020, c	TTCACCGAGGCCACGCGGAGTACGATCGAGTGTACAGTGAA
MT459987.1 Severe acute respiratory syndrome coronavirus 2 isolate SARS-CoV-2/human/GUM/GU-NHG-03/2020, c	TTCACCGAGGCCACGCGGAGTACGATCGAGTGTACAGTGAA
MT459988.1 Severe acute respiratory syndrome coronavirus 2 isolate SARS-CoV-2/human/USA/SC-NHRC-01/2020, co	TTCACCGAGGCCACGCGGAGTACGATCGAGTGTACAGTGAA
MT459989.1 Severe acute respiratory syndrome coronavirus 2 isolate SARS-CoV-2/human/USA/SC-NHRC-02/2020, co	TTCACCGAGGCCACGCGGAGTACGATCGAGTGTACAGTGAA
MT459990.1 Severe acute respiratory syndrome coronavirus 2 isolate SARS-CoV-2/human/USA/SC-NHRC-03/2020, co	TTCACCGAGGCCACGCGGAGTACGATCGAGTGTACAGTGAA
MT460089.1 Severe acute respiratory syndrome coronavirus 2 isolate SARS-CoV-2/human/USA/CA-CZB-1089/2020, c	TTCACCGAGGCCACGCGGAGTACGATCGAGTGTACAGTGAA
MT460090.1 Severe acute respiratory syndrome coronavirus 2 isolate SARS-CoV-2/human/USA/CA-CZB-1090/2020, c	TTCACCGAGGCCACGCGGAGTACGATCGAGTGTACAGTGAA
MT460091.1 Severe acute respiratory syndrome coronavirus 2 isolate SARS-CoV-2/human/USA/CA-CZB-1091/2020, c	TTCACCGAGGCCACGCGGAGTACGATCGAGTGTACAGTGAA
MT460092.1 Severe acute respiratory syndrome coronavirus 2 isolate SARS-CoV-2/human/USA/CA-CZB-1092/2020, c	TTCACCGAGGCCACGCGGAGTACGATCGAGTGTACAGTGAA
MT460113.1 Severe acute respiratory syndrome coronavirus 2 isolate SARS-CoV-2/human/USA/CA-CZB-1093/2020, c	TTCACCGAGGCCACGCGGAGTACGATCGAGTGTACAGTGAA
MT460114.1 Severe acute respiratory syndrome coronavirus 2 isolate SARS-CoV-2/human/USA/CA-CZB-1094/2020, c	TTCACCGAGGCCACGCGGAGTACGATCGAGTGTACAGTGAA
MT460115.1 Severe acute respiratory syndrome coronavirus 2 isolate SARS-CoV-2/human/USA/CA-CZB-1095/2020, c	TTCACCGAGGCCACGCGGAGTACGATCGAGTGTACAGTGAA
MT460116.1 Severe acute respiratory syndrome coronavirus 2 isolate SARS-CoV-2/human/USA/CA-CZB-1096/2020, c	TTCACCGAGGCCACGCGGAGTACGATCGAGTGTACAGTGAA
MT460117.1 Severe acute respiratory syndrome coronavirus 2 isolate SARS-CoV-2/human/USA/CA-CZB-1097/2020, c	TTCACCGAGGCCACGCGGAGTACGATCGAGTGTACAGTGAA
MT460118.1 Severe acute respiratory syndrome coronavirus 2 isolate SARS-CoV-2/human/USA/CA-CZB-1098/2020 O	TTCACCGAGGCCACGCGGAGTACGATCGAGTGTACAGTGAA
MT460119.1 Severe acute respiratory syndrome coronavirus 2 isolate SARS-CoV-2/human/USA/CA-CZB-1099/2020, c	TTCACCGAGGCCACGCGGAGTACGATCGAGTGTACAGTGAA
MT460120.1 Severe acute respiratory syndrome coronavirus 2 isolate SARS-CoV-2/human/USA/CA-CZB-1100/2020, c	TTCACCGAGGCCACGCGGAGTACGATCGAGTGTACAGTGAA
MT460121.1 Severe acute respiratory syndrome coronavirus 2 isolate SARS-CoV-2/human/USA/CA-CZB-1101/2020, c	TTCACCGAGGCCACGCGGAGTACGATCGAGTGTACAGTGAA
MT460122.1 Severe acute respiratory syndrome coronavirus 2 isolate SARS-CoV-2/human/USA/CA-CZB-1102/2020 O	TTCACCGAGGCCACGCGGAGTACGATCGAGTGTACAGTGAA

MT460123.1 Severe acute respiratory syndrome coronavirus 2 isolate SARS-CoV-2/human/USA/CA-CZB-1103/2020 O	TTCACCGAGGCCACGCGGAGTACGATCGAGTGTACAGTGAA
MT460124.1 Severe acute respiratory syndrome coronavirus 2 isolate SARS-CoV-2/human/USA/CA-CZB-1104/2020, c	TTCACCGAGGCCACGCGGAGTACGATCGAGTGTACAGTGAA
MT460125.1 Severe acute respiratory syndrome coronavirus 2 isolate SARS-CoV-2/human/USA/CA-CZB-1105/2020, c	TTCACCGAGGCCACGCGGAGTACGATCGAGTGTACAGTGAA
MT460126.1 Severe acute respiratory syndrome coronavirus 2 isolate SARS-CoV-2/human/USA/CA-CZB-1106/2020 O	TTCACCGAGGCCACGCGGAGTACGATCGAGTGTACAGTGAA
MT460128.1 Severe acute respiratory syndrome coronavirus 2 isolate SARS-CoV-2/human/USA/CA-CZB-1108/2020, c	TTCACCGAGGCCACGCGGAGTACGATCGAGTGTACAGTGAA
MT460129.1 Severe acute respiratory syndrome coronavirus 2 isolate SARS-CoV-2/human/USA/CA-CZB-1109/2020, c	TTCACCGAGGCCACGCGGAGTACGATCGAGTGTACAGTGAA
MT460130.1 Severe acute respiratory syndrome coronavirus 2 isolate SARS-CoV-2/human/USA/CA-CZB-1110/2020, c	TTCACCGAGGCCACGCGGAGTACGATCGAGTGTACAGTGAA
MT460131.1 Severe acute respiratory syndrome coronavirus 2 isolate SARS-CoV-2/human/USA/CA-CZB-1111/2020 O	TTCACCGAGGCCACGCGGAGTACGATCGAGTGTACAGTGAA
MT460132.1 Severe acute respiratory syndrome coronavirus 2 isolate SARS-CoV-2/human/USA/CA-CZB-1112/2020, c	TTCACCGAGGCCACGCGGAGTACGATCGAGTGTACAGTGAA
MT460133.1 Severe acute respiratory syndrome coronavirus 2 isolate SARS-CoV-2/human/USA/CA-CZB-1113/2020, c	TTCACCGAGGCCACGCGGAGTACGATCGAGTGTACAGTGAA
MT460134.1 Severe acute respiratory syndrome coronavirus 2 isolate SARS-CoV-2/human/USA/CA-CZB-1114/2020 O	TTCACCGAGGCCACGCGGAGTACGATCGAGTGTACAGTGAA
MT460135.1 Severe acute respiratory syndrome coronavirus 2 isolate SARS-CoV-2/human/USA/CA-CZB-1115/2020, c	TTCACCGAGGCCACGCGGAGTACGATCGAGTGTACAGTGAA
MT460136.1 Severe acute respiratory syndrome coronavirus 2 isolate SARS-CoV-2/human/USA/CA-CZB-1116/2020, c	TTCACCGAGGCCACGCGGAGTACGATCGAGTGTACAGTGAA
MT460137.1 Severe acute respiratory syndrome coronavirus 2 isolate SARS-CoV-2/human/USA/CA-CZB-1117/2020, c	TTCACCGAGGCCACGCGGAGTACGATCGAGTGTACAGTGAA
MT460138.1 Severe acute respiratory syndrome coronavirus 2 isolate SARS-CoV-2/human/USA/CA-CZB-1118/2020, c	TTCACCGAGGCCACGCGGAGTACGATCGAGTGTACAGTGAA
MT460139.1 Severe acute respiratory syndrome coronavirus 2 isolate SARS-CoV-2/human/USA/CA-CZB-1119/2020, c	TTCACCGAGGCCACGCGGAGTACGATCGAGTGTACAGTGAA
MT460140.1 Severe acute respiratory syndrome coronavirus 2 isolate SARS-CoV-2/human/USA/CA-CZB-1120/2020, c	TTCACCGAGGCCACGCGGAGTACGATCGAGTGTACAGTGAA
MT461603.1 Severe acute respiratory syndrome coronavirus 2 isolate SARS-CoV-2/human/USA/UNKNOWN-UW-3982	TTCACCGAGGCCACGCGGAGTACGATCGAGTGTACAGTGAA
MT461604.1 Severe acute respiratory syndrome coronavirus 2 isolate SARS-CoV-2/human/USA/WA-UW-4009/2020,	TTCACCGAGGCCACGCGGAGTACGATCGAGTGTACAGTGAA
MT461605.1 Severe acute respiratory syndrome coronavirus 2 isolate SARS-CoV-2/human/USA/WA-UW-4014/2020,	TTCACCGAGGCCACGCGGAGTACGATCGAGTGTACAGTGAA
MT461606.1 Severe acute respiratory syndrome coronavirus 2 isolate SARS-CoV-2/human/USA/WA-UW-4015/2020,	TTCACCGAGGCCACGCGGAGTACGATCGAGTGTACAGTGAA
MT461607.1 Severe acute respiratory syndrome coronavirus 2 isolate SARS-CoV-2/human/USA/WA-UW-4021/2020,	TTCACCGAGGCCACGCGGAGTACGATCGAGTGTACAGTGAA
MT461608.1 Severe acute respiratory syndrome coronavirus 2 isolate SARS-CoV-2/human/USA/WA-UW-4024/2020,	TTCACCGAGGCCACGCGGAGTACGATCGAGTGTACAGTGAA
MT461609.1 Severe acute respiratory syndrome coronavirus 2 isolate SARS-CoV-2/human/USA/WA-UW-4026/2020,	TTCACCGAGGCCACGCGGAGTACGATCGAGTGTACAGTGAA
MT461610.1 Severe acute respiratory syndrome coronavirus 2 isolate SARS-CoV-2/human/USA/WA-UW-4032/2020,	TTCACCGAGGCCACGCGGAGTACGATCGAGTGTACAGTGAA
MT461611.1 Severe acute respiratory syndrome coronavirus 2 isolate SARS-CoV-2/human/USA/WA-UW-4041/2020,	TTCACCGAGGCCACGCGGAGTACGATCGAGTGTACAGTGAA
MT461612.1 Severe acute respiratory syndrome coronavirus 2 isolate SARS-CoV-2/human/USA/WA-UW-4046/2020,	TTCACCGAGGCCACGCGGAGTACGATCGAGTGTACAGTGAA
MT461613.1 Severe acute respiratory syndrome coronavirus 2 isolate SARS-CoV-2/human/USA/WA-UW-4051/2020,	TTCACCGAGGCCACGCGGAGTACGATCGAGTGTACAGTGAA
MT461614.1 Severe acute respiratory syndrome coronavirus 2 isolate SARS-CoV-2/human/USA/WA-UW-4058/2020,	TTCACCGAGGCCACGCGGAGTACGATCGAGTGTACAGTGAA
MT461615.1 Severe acute respiratory syndrome coronavirus 2 isolate SARS-CoV-2/human/USA/WA-UW-4061/2020,	TTCACCGAGGCCACGCGGAGTACGATCGAGTGTACAGTGAA
MT461616.1 Severe acute respiratory syndrome coronavirus 2 isolate SARS-CoV-2/human/USA/WA-UW-4065/2020,	TTCACCGAGGCCACGCGGAGTACGATCGAGTGTACAGTGAA
MT461617.1 Severe acute respiratory syndrome coronavirus 2 isolate SARS-CoV-2/human/USA/WA-UW-4067/2020,	TTCACCGAGGCCACGCGGAGTACGATCGAGTGTACAGTGAA
MT461618.1 Severe acute respiratory syndrome coronavirus 2 isolate SARS-CoV-2/human/USA/UNKNOWN-UW-408[illegible]	TTCACCGAGGCCACGCGGAGTACGATCGAGTGTACAGTGAA

MT461619.1 Severe acute respiratory syndrome coronavirus 2 isolate SARS-CoV-2/human/USA/UNKNOWN-UW-4097	TTCACCGAGGCCACGCGGAGTACGATCGAGTGTACAGTGAA
MT461621.1 Severe acute respiratory syndrome coronavirus 2 isolate SARS-CoV-2/human/USA/WA-UW-4210/2020, c	TTCACCGAGGCCACGCGGAGTACGATCGAGTGTACAGTGAA
MT461622.1 Severe acute respiratory syndrome coronavirus 2 isolate SARS-CoV-2/human/USA/WA-UW-4221/2020, c	TTCACCGAGGCCACGCGGAGTACGATCGAGTGTACAGTGAA
MT461623.1 Severe acute respiratory syndrome coronavirus 2 isolate SARS-CoV-2/human/USA/WA-UW-4225/2020, c	TTCACCGAGGCCACGCGGAGTACGATCGAGTGTACAGTGAA
MT461624.1 Severe acute respiratory syndrome coronavirus 2 isolate SARS-CoV-2/human/USA/CT-UW-4237/2020, co	TTCACCGAGGCCACGCGGAGTACGATCGAGTGTACAGTGAA
MT461625.1 Severe acute respiratory syndrome coronavirus 2 isolate SARS-CoV-2/human/USA/ID-UW-4254/2020, co	TTCACCGAGGCCACGCGGAGTACGATCGAGTGTACAGTGAA
MT461627.1 Severe acute respiratory syndrome coronavirus 2 isolate SARS-CoV-2/human/USA/WA-UW-4296/2020, c	TTCACCGAGGCCACGCGGAGTACGATCGAGTGTACAGTGAA
MT461628.1 Severe acute respiratory syndrome coronavirus 2 isolate SARS-CoV-2/human/USA/ID-UW-4308/2020, co	TTCACCGAGGCCACGCGGAGTACGATCGAGTGTACAGTGAA
MT461629.1 Severe acute respiratory syndrome coronavirus 2 isolate SARS-CoV-2/human/USA/WA-UW-4317/2020, c	TTCACCGAGGCCACGCGGAGTACGATCGAGTGTACAGTGAA
MT461630.1 Severe acute respiratory syndrome coronavirus 2 isolate SARS-CoV-2/human/USA/WA-UW-4414/2020, c	TTCACCGAGGCCACGCGGAGTACGATCGAGTGTACAGTGAA
MT461634.1 Severe acute respiratory syndrome coronavirus 2 isolate SARS-CoV-2/human/USA/ID-UW-6457/2020, co	TTCACCGAGGCCACGCGGAGTACGATCGAGTGTACAGTGAA
MT461635.1 Severe acute respiratory syndrome coronavirus 2 isolate SARS-CoV-2/human/USA/ID-UW-6466/2020, co	TTCACCGAGGCCACGCGGAGTACGATCGAGTGTACAGTGAA
MT461639.1 Severe acute respiratory syndrome coronavirus 2 isolate SARS-CoV-2/human/USA/WA-UW-6478/2020, c	TTCACCGAGGCCACGCGGAGTACGATCGAGTGTACAGTGAA
MT461640.1 Severe acute respiratory syndrome coronavirus 2 isolate SARS-CoV-2/human/USA/WA-UW-6480/2020, c	TTCACCGAGGCCACGCGGAGTACGATCGAGTGTACAGTGAA
MT461641.1 Severe acute respiratory syndrome coronavirus 2 isolate SARS-CoV-2/human/USA/WA-UW-6481/2020, c	TTCACCGAGGCCACGCGGAGTACGATCGAGTGTACAGTGAA
MT461643.1 Severe acute respiratory syndrome coronavirus 2 isolate SARS-CoV-2/human/USA/WA-UW-6484/2020, c	TTCACCGAGGCCACGCGGAGTACGATCGAGTGTACAGTGAA
MT461644.1 Severe acute respiratory syndrome coronavirus 2 isolate SARS-CoV-2/human/USA/WA-UW-6485/2020, c	TTCACCGAGGCCACGCGGAGTACGATCGAGTGTACAGTGAA
MT461645.1 Severe acute respiratory syndrome coronavirus 2 isolate SARS-CoV-2/human/USA/WA-UW-6486/2020, c	TTCACCGAGGCCACGCGGAGTACGATCGAGTGTACAGTGAA
MT461646.1 Severe acute respiratory syndrome coronavirus 2 isolate SARS-CoV-2/human/USA/WA-UW-6491/2020, c	TTCACCGAGGCCACGCGGAGTACGATCGAGTGTACAGTGAA
MT461647.1 Severe acute respiratory syndrome coronavirus 2 isolate SARS-CoV-2/human/USA/WA-UW-6497/2020, c	TTCACCGAGGCCACGCGGAGTACGATCGAGTGTACAGTGAA
MT461648.1 Severe acute respiratory syndrome coronavirus 2 isolate SARS-CoV-2/human/USA/WA-UW-6501/2020, c	TTCACCGAGGCCACGCGGAGTACGATCGAGTGTACAGTGAA
MT461650.1 Severe acute respiratory syndrome coronavirus 2 isolate SARS-CoV-2/human/USA/WA-UW-6507/2020, c	TTCACCGAGGCCACGCGGAGTACGATCGAGTGTACAGTGAA
MT461651.1 Severe acute respiratory syndrome coronavirus 2 isolate SARS-CoV-2/human/USA/WA-UW-6514/2020, c	TTCACCGAGGCCACGCGGAGTACGATCGAGTGTACAGTGAA
MT461652.1 Severe acute respiratory syndrome coronavirus 2 isolate SARS-CoV-2/human/USA/WA-UW-6520/2020, c	TTCACCGAGGCCACGCGGAGTACGATCGAGTGTACAGTGAA
MT461653.1 Severe acute respiratory syndrome coronavirus 2 isolate SARS-CoV-2/human/USA/WA-UW-6526/2020, c	TTCACCGAGGCCACGCGGAGTACGATCGAGTGTACAGTGAA
MT461654.1 Severe acute respiratory syndrome coronavirus 2 isolate SARS-CoV-2/human/USA/WA-UW-6527/2020, c	TTCACCGAGGCCACGCGGAGTACGATCGAGTGTACAGTGAA
MT461655.1 Severe acute respiratory syndrome coronavirus 2 isolate SARS-CoV-2/human/USA/WA-UW-6528/2020, c	TTCACCGAGGCCACGCGGAGTACGATCGAGTGTACAGTGAA
MT461656.1 Severe acute respiratory syndrome coronavirus 2 isolate SARS-CoV-2/human/USA/WA-UW-6535/2020, c	TTCACCGAGGCCACGCGGAGTACGATCGAGTGTACAGTGAA
MT461657.1 Severe acute respiratory syndrome coronavirus 2 isolate SARS-CoV-2/human/USA/WA-UW-6546/2020, c	TTCACCGAGGCCACGCGGAGTACGATCGAGTGTACAGTGAA
MT461658.1 Severe acute respiratory syndrome coronavirus 2 isolate SARS-CoV-2/human/USA/WA-UW-6547/2020, c	TTCACCGAGGCCACGCGGAGTACGATCGAGTGTACAGTGAA
MT461659.1 Severe acute respiratory syndrome coronavirus 2 isolate SARS-CoV-2/human/USA/WA-UW-6548/2020, c	TTCACCGAGGCCACGCGGAGTACGATCGAGTGTACAGTGAA
MT461660.1 Severe acute respiratory syndrome coronavirus 2 isolate SARS-CoV-2/human/USA/WA-UW-6559/2020, c	TTCACCGAGGCCACGCGGAGTACGATCGAGTGTACAGTGAA
MT461661.1 Severe acute respiratory syndrome coronavirus 2 isolate SARS-CoV-2/human/USA/WA-UW-6562/2020 C	TTCACCGAGGCCACGCGGAGTACGATCGAGTGTACAGTGAA

MT461662.1 Severe acute respiratory syndrome coronavirus 2 isolate SARS-CoV-2/human/USA/CT-UW-6564/2020, c	TTCACCGAGGCCACGCGGAGTACGATCGAGTGTACAGTGAA
MT461663.1 Severe acute respiratory syndrome coronavirus 2 isolate SARS-CoV-2/human/USA/CT-UW-6565/2020, c	TTCACCGAGGCCACGCGGAGTACGATCGAGTGTACAGTGAA
MT461664.1 Severe acute respiratory syndrome coronavirus 2 isolate SARS-CoV-2/human/USA/CT-UW-6569/2020, c	TTCACCGAGGCCACGCGGAGTACGATCGAGTGTACAGTGAA
MT461665.1 Severe acute respiratory syndrome coronavirus 2 isolate SARS-CoV-2/human/USA/CT-UW-6571/2020 O	TTCACCGAGGCCACGCGGAGTACGATCGAGTGTACAGTGAA
MT461666.1 Severe acute respiratory syndrome coronavirus 2 isolate SARS-CoV-2/human/USA/CT-UW-6573/2020, c	TTCACCGAGGCCACGCGGAGTACGATCGAGTGTACAGTGAA
MT461667.1 Severe acute respiratory syndrome coronavirus 2 isolate SARS-CoV-2/human/USA/CT-UW-6574/2020, c	TTCACCGAGGCCACGCGGAGTACGATCGAGTGTACAGTGAA
MT461668.1 Severe acute respiratory syndrome coronavirus 2 isolate SARS-CoV-2/human/USA/CT-UW-6576/2020, c	TTCACCGAGGCCACGCGGAGTACGATCGAGTGTACAGTGAA
MT466071.1 Severe acute respiratory syndrome coronavirus 2 isolate SARS-CoV-2/human/URY/Mdeo-1/2020, compl	TTCACCGAGGCCACGCGGAGTACGATCGAGTGTACAGTGAA
MT466583.1 Severe acute respiratory syndrome coronavirus 2 isolate SARS-CoV-2/human/USA/AK002/2020, comple	TTCACCGAGGCCACGCGGAGTACGATCGAGTGTACAGTGAA
MT467237.1 Severe acute respiratory syndrome coronavirus 2 isolate SARS-CoV-2/human/IND/GBRC25/2020, compl	TTCACCGAGGCCACGCGGAGTACGATCGAGTGTACAGTGAA
MT467238.1 Severe acute respiratory syndrome coronavirus 2 isolate SARS-CoV-2/human/IND/GBRC46/2020, compl	TTCACCGAGGCCACGCGGAGTACGATCGAGTGTACAGTGAA
MT467239.1 Severe acute respiratory syndrome coronavirus 2 isolate SARS-CoV-2/human/IND/GBRC26/2020, compl	TTCACCGAGGCCACGCGGAGTACGATCGAGTGTACAGTGAA
MT467240.1 Severe acute respiratory syndrome coronavirus 2 isolate SARS-CoV-2/human/IND/GBRC47/2020, compl	TTCACCGAGGCCACGCGGAGTACGATCGAGTGTACAGTGAA
MT467241.1 Severe acute respiratory syndrome coronavirus 2 isolate SARS-CoV-2/human/IND/GBRC27/2020, compl	TTCACCGAGGCCACGCGGAGTACGATCGAGTGTACAGTGAA
MT467242.1 Severe acute respiratory syndrome coronavirus 2 isolate SARS-CoV-2/human/IND/GBRC48/2020, compl	TTCACCGAGGCCACGCGGAGTACGATCGAGTGTACAGTGAA
MT467243.1 Severe acute respiratory syndrome coronavirus 2 isolate SARS-CoV-2/human/IND/GBRC28/2020, compl	TTCACCGAGGCCACGCGGAGTACGATCGAGTGTACAGTGAA
MT467244.1 Severe acute respiratory syndrome coronavirus 2 isolate SARS-CoV-2/human/IND/GBRC29/2020, compl	TTCACCGAGGCCACGCGGAGTACGATCGAGTGTACAGTGAA
MT467245.1 Severe acute respiratory syndrome coronavirus 2 isolate SARS-CoV-2/human/IND/GBRC30/2020, compl	TTCACCGAGGCCACGCGGAGTACGATCGAGTGTACAGTGAA
MT467246.1 Severe acute respiratory syndrome coronavirus 2 isolate SARS-CoV-2/human/IND/GBRC40/2020, compl	TTCACCGAGGCCACGCGGAGTACGATCGAGTGTACAGTGAA
MT467247.1 Severe acute respiratory syndrome coronavirus 2 isolate SARS-CoV-2/human/IND/GBRC31/2020, compl	TTCACCGAGGCCACGCGGAGTACGATCGAGTGTACAGTGAA
MT467248.1 Severe acute respiratory syndrome coronavirus 2 isolate SARS-CoV-2/human/IND/GBRC41/2020, compl	TTCACCGAGGCCACGCGGAGTACGATCGAGTGTACAGTGAA
MT467249.1 Severe acute respiratory syndrome coronavirus 2 isolate SARS-CoV-2/human/IND/GBRC32/2020, compl	TTCACCGAGGCCACGCGGAGTACGATCGAGTGTACAGTGAA
MT467250.1 Severe acute respiratory syndrome coronavirus 2 isolate SARS-CoV-2/human/IND/GBRC42/2020, compl	TTCACCGAGGCCACGCGGAGTACGATCGAGTGTACAGTGAA
MT467251.1 Severe acute respiratory syndrome coronavirus 2 isolate SARS-CoV-2/human/IND/GBRC43/2020, compl	TTCACCGAGGCCACGCGGAGTACGATCGAGTGTACAGTGAA
MT467252.1 Severe acute respiratory syndrome coronavirus 2 isolate SARS-CoV-2/human/IND/GBRC44/2020, compl	TTCACCGAGGCCACGCGGAGTACGATCGAGTGTACAGTGAA
MT467253.1 Severe acute respiratory syndrome coronavirus 2 isolate SARS-CoV-2/human/IND/GBRC45/2020, compl	TTCACCGAGGCCACGCGGAGTACGATCGAGTGTACAGTGAA
MT467254.1 Severe acute respiratory syndrome coronavirus 2 isolate SARS-CoV-2/human/IND/GBRC33/2020, compl	TTCACCGAGGCCACGCGGAGTACGATCGAGTGTACAGTGAA
MT467255.1 Severe acute respiratory syndrome coronavirus 2 isolate SARS-CoV-2/human/IND/GBRC49/2020, compl	TTCACCGAGGCCACGCGGAGTACGATCGAGTGTACAGTGAA
MT467256.1 Severe acute respiratory syndrome coronavirus 2 isolate SARS-CoV-2/human/IND/GBRC34/2020, compl	TTCACCGAGGCCACGCGGAGTACGATCGAGTGTACAGTGAA
MT467257.1 Severe acute respiratory syndrome coronavirus 2 isolate SARS-CoV-2/human/IND/GBRC50/2020, compl	TTCACCGAGGCCACGCGGAGTACGATCGAGTGTACAGTGAA
MT467258.1 Severe acute respiratory syndrome coronavirus 2 isolate SARS-CoV-2/human/IND/GBRC35/2020, compl	TTCACCGAGGCCACGCGGAGTACGATCGAGTGTACAGTGAA
MT467259.1 Severe acute respiratory syndrome coronavirus 2 isolate SARS-CoV-2/human/IND/GBRC51/2020, compl	TTCACCGAGGCCACGCGGAGTACGATCGAGTGTACAGTGAA
MT467260.1 Severe acute respiratory syndrome coronavirus 2 isolate SARS-CoV-2/human/IND/GBRC52/2020, compl	TTCACCGAGGCCACGCGGAGTACGATCGAGTGTACAGTGAA

MT467261.1 Severe acute respiratory syndrome coronavirus 2 isolate SARS-CoV-2/human/IND/GBRC53/2020, compl	TTCACCGAGGCCACGCGGAGTACGATCGAGTGTACAGTGAA
MT467262.1 Severe acute respiratory syndrome coronavirus 2 isolate SARS-CoV-2/human/IND/GBRC54/2020, compl	TTCACCGAGGCCACGCGGAGTACGATCGAGTGTACAGTGAA
MT467263.1 Severe acute respiratory syndrome coronavirus 2 isolate SARS-CoV-2/human/IND/GBRC55/2020, compl	TTCACCGAGGCCACGCGGAGTACGATCGAGTGTACAGTGAA
MT470100.1 Severe acute respiratory syndrome coronavirus 2 isolate SARS-CoV-2/human/France/10027AA/2020, co	TTCACCGAGGCCACGCGGAGTACGATCGAGTGTACAGTGAA
MT470101.1 Severe acute respiratory syndrome coronavirus 2 isolate SARS-CoV-2/human/France/70001GR/2020, co	TTCACCGAGGCCACGCGGAGTACGATCGAGTGTACAGTGAA
MT470102.1 Severe acute respiratory syndrome coronavirus 2 isolate SARS-CoV-2/human/France/40004SR/2020, cor	TTCACCGAGGCCACGCGGAGTACGATCGAGTGTACAGTGAA
MT470103.1 Severe acute respiratory syndrome coronavirus 2 isolate SARS-CoV-2/human/France/50010DC/2020, co	TTCACCGAGGCCACGCGGAGTACGATCGAGTGTACAGTGAA
MT470104.1 Severe acute respiratory syndrome coronavirus 2 isolate SARS-CoV-2/human/France/40005BH/2020, co	TTCACCGAGGCCACGCGGAGTACGATCGAGTGTACAGTGAA
MT470105.1 Severe acute respiratory syndrome coronavirus 2 isolate SARS-CoV-2/human/France/60003NG/2020, co	TTCACCGAGGCCACGCGGAGTACGATCGAGTGTACAGTGAA
MT470106.1 Severe acute respiratory syndrome coronavirus 2 isolate SARS-CoV-2/human/France/10036BG/2020, co	TTCACCGAGGCCACGCGGAGTACGATCGAGTGTACAGTGAA
MT470107.1 Severe acute respiratory syndrome coronavirus 2 isolate SARS-CoV-2/human/France/10037CJ/2020, cor	TTCACCGAGGCCACGCGGAGTACGATCGAGTGTACAGTGAA
MT470108.1 Severe acute respiratory syndrome coronavirus 2 isolate SARS-CoV-2/human/France/40006SG/2020, co	TTCACCGAGGCCACGCGGAGTACGATCGAGTGTACAGTGAA
MT470109.1 Severe acute respiratory syndrome coronavirus 2 isolate SARS-CoV-2/human/France/10030NV/2020, co	TTCACCGAGGCCACGCGGAGTACGATCGAGTGTACAGTGAA
MT470110.1 Severe acute respiratory syndrome coronavirus 2 isolate SARS-CoV-2/human/France/10040LJ/2020, con	TTCACCGAGGCCACGCGGAGTACGATCGAGTGTACAGTGAA
MT470111.1 Severe acute respiratory syndrome coronavirus 2 isolate SARS-CoV-2/human/France/10041MR/2020, c	TTCACCGAGGCCACGCGGAGTACGATCGAGTGTACAGTGAA
MT470112.1 Severe acute respiratory syndrome coronavirus 2 isolate SARS-CoV-2/human/France/20001SE/2020, cor	TTCACCGAGGCCACGCGGAGTACGATCGAGTGTACAGTGAA
MT470113.1 Severe acute respiratory syndrome coronavirus 2 isolate SARS-CoV-2/human/France/10048SA/2020, cor	TTCACCGAGGCCACGCGGAGTACGATCGAGTGTACAGTGAA
MT470114.1 Severe acute respiratory syndrome coronavirus 2 isolate SARS-CoV-2/human/France/10046GM/2020, c	TTCACCGAGGCCACGCGGAGTACGATCGAGTGTACAGTGAA
MT470115.1 Severe acute respiratory syndrome coronavirus 2 isolate SARS-CoV-2/human/France/10029DT/2020, co	TTCACCGAGGCCACGCGGAGTACGATCGAGTGTACAGTGAA
MT470116.1 Severe acute respiratory syndrome coronavirus 2 isolate SARS-CoV-2/human/France/10031BS/2020, cor	TTCACCGAGGCCACGCGGAGTACGATCGAGTGTACAGTGAA
MT470117.1 Severe acute respiratory syndrome coronavirus 2 isolate SARS-CoV-2/human/France/60002VV/2020, co	TTCACCGAGGCCACGCGGAGTACGATCGAGTGTACAGTGAA
MT470118.1 Severe acute respiratory syndrome coronavirus 2 isolate SARS-CoV-2/human/France/10032KL/2020, cor	TTCACCGAGGCCACGCGGAGTACGATCGAGTGTACAGTGAA
MT470119.1 Severe acute respiratory syndrome coronavirus 2 isolate SARS-CoV-2/human/France/50008KC/2020, cor	TTCACCGAGGCCACGCGGAGTACGATCGAGTGTACAGTGAA
MT470120.1 Severe acute respiratory syndrome coronavirus 2 isolate SARS-CoV-2/human/France/10033ZM/2020, c	TTCACCGAGGCCACGCGGAGTACGATCGAGTGTACAGTGAA
MT470121.1 Severe acute respiratory syndrome coronavirus 2 isolate SARS-CoV-2/human/France/10034HM/2020, c	TTCACCGAGGCCACGCGGAGTACGATCGAGTGTACAGTGAA
MT470122.1 Severe acute respiratory syndrome coronavirus 2 isolate SARS-CoV-2/human/France/10050RA/2020, co	TTCACCGAGGCCACGCGGAGTACGATCGAGTGTACAGTGAA
MT470123.1 Severe acute respiratory syndrome coronavirus 2 isolate SARS-CoV-2/human/France/10045DZ/2020, co	TTCACCGAGGCCACGCGGAGTACGATCGAGTGTACAGTGAA
MT470124.1 Severe acute respiratory syndrome coronavirus 2 isolate SARS-CoV-2/human/France/10051SM/2020, c	TTCACCGAGGCCACGCGGAGTACGATCGAGTGTACAGTGAA
MT470125.1 Severe acute respiratory syndrome coronavirus 2 isolate SARS-CoV-2/human/France/10063BI/2020, con	TTCACCGAGGCCACGCGGAGTACGATCGAGTGTACAGTGAA
MT470126.1 Severe acute respiratory syndrome coronavirus 2 isolate SARS-CoV-2/human/France/10066GJ/2020, cor	TTCACCGAGGCCACGCGGAGTACGATCGAGTGTACAGTGAA
MT470127.1 Severe acute respiratory syndrome coronavirus 2 isolate SARS-CoV-2/human/France/10069NM/2020, c	TTCACCGAGGCCACGCGGAGTACGATCGAGTGTACAGTGAA
MT470128.1 Severe acute respiratory syndrome coronavirus 2 isolate SARS-CoV-2/human/France/10071BR/2020, co	TTCACCGAGGCCACGCGGAGTACGATCGAGTGTACAGTGAA
MT470129.1 Severe acute respiratory syndrome coronavirus 2 isolate SARS-CoV-2/human/France/10060KV/2020, co	TTCACCGAGGCCACGCGGAGTACGATCGAGTGTACAGTGAA

MT470130.1 Severe acute respiratory syndrome coronavirus 2 isolate SARS-CoV-2/human/France/10074LD/2020, com	TTCACCGAGGCCACGCGGAGTACGATCGAGTGTACAGTGAA
MT470131.1 Severe acute respiratory syndrome coronavirus 2 isolate SARS-CoV-2/human/France/10072CG/2020, co	TTCACCGAGGCCACGCGGAGTACGATCGAGTGTACAGTGAA
MT470132.1 Severe acute respiratory syndrome coronavirus 2 isolate SARS-CoV-2/human/France/10076MH/2020, co	TTCACCGAGGCCACGCGGAGTACGATCGAGTGTACAGTGAA
MT470133.1 Severe acute respiratory syndrome coronavirus 2 isolate SARS-CoV-2/human/France/10078MA/2020, co	TTCACCGAGGCCACGCGGAGTACGATCGAGTGTACAGTGAA
MT470134.1 Severe acute respiratory syndrome coronavirus 2 isolate SARS-CoV-2/human/France/10057CJ/2020, com	TTCACCGAGGCCACGCGGAGTACGATCGAGTGTACAGTGAA
MT470135.1 Severe acute respiratory syndrome coronavirus 2 isolate SARS-CoV-2/human/France/10062MD/2020, co	TTCACCGAGGCCACGCGGAGTACGATCGAGTGTACAGTGAA
MT470136.1 Severe acute respiratory syndrome coronavirus 2 isolate SARS-CoV-2/human/France/10059IS/2020, com	TTCACCGAGGCCACGCGGAGTACGATCGAGTGTACAGTGAA
MT470137.1 Severe acute respiratory syndrome coronavirus 2 isolate SARS-CoV-2/human/France/10068ND/2020, co	TTCACCGAGGCCACGCGGAGTACGATCGAGTGTACAGTGAA
MT470138.1 Severe acute respiratory syndrome coronavirus 2 isolate SARS-CoV-2/human/France/40007RD/2020, co	TTCACCGAGGCCACGCGGAGTACGATCGAGTGTACAGTGAA
MT470139.1 Severe acute respiratory syndrome coronavirus 2 isolate SARS-CoV-2/human/France/5003KM/2020, com	TTCACCGAGGCCACGCGGAGTACGATCGAGTGTACAGTGAA
MT470140.1 Severe acute respiratory syndrome coronavirus 2 isolate SARS-CoV-2/human/France/50002BS/2020, com	TTCACCGAGGCCACGCGGAGTACGATCGAGTGTACAGTGAA
MT470141.1 Severe acute respiratory syndrome coronavirus 2 isolate SARS-CoV-2/human/France/10004CF/2020, com	TTCACCGAGGCCACGCGGAGTACGATCGAGTGTACAGTGAA
MT470142.1 Severe acute respiratory syndrome coronavirus 2 isolate SARS-CoV-2/human/France/10009EE/2020, com	TTCACCGAGGCCACGCGGAGTACGATCGAGTGTACAGTGAA
MT470143.1 Severe acute respiratory syndrome coronavirus 2 isolate SARS-CoV-2/human/France/10008DM/2020, co	TTCACCGAGGCCACGCGGAGTACGATCGAGTGTACAGTGAA
MT470144.1 Severe acute respiratory syndrome coronavirus 2 isolate SARS-CoV-2/human/France/50001AR/2020, co	TTCACCGAGGCCACGCGGAGTACGATCGAGTGTACAGTGAA
MT470145.1 Severe acute respiratory syndrome coronavirus 2 isolate SARS-CoV-2/human/France/10010LA/2020, com	TTCACCGAGGCCACGCGGAGTACGATCGAGTGTACAGTGAA
MT470146.1 Severe acute respiratory syndrome coronavirus 2 isolate SARS-CoV-2/human/France/10014BC/2020, com	TTCACCGAGGCCACGCGGAGTACGATCGAGTGTACAGTGAA
MT470147.1 Severe acute respiratory syndrome coronavirus 2 isolate SARS-CoV-2/human/France/10080AA/2020, co	TTCACCGAGGCCACGCGGAGTACGATCGAGTGTACAGTGAA
MT470148.1 Severe acute respiratory syndrome coronavirus 2 isolate SARS-CoV-2/human/France/10019LM/2020, co	TTCACCGAGGCCACGCGGAGTACGATCGAGTGTACAGTGAA
MT470149.1 Severe acute respiratory syndrome coronavirus 2 isolate SARS-CoV-2/human/France/10018HJ/2020, com	TTCACCGAGGCCACGCGGAGTACGATCGAGTGTACAGTGAA
MT470150.1 Severe acute respiratory syndrome coronavirus 2 isolate SARS-CoV-2/human/France/10020MM/2020, c	TTCACCGAGGCCACGCGGAGTACGATCGAGTGTACAGTGAA
MT470151.1 Severe acute respiratory syndrome coronavirus 2 isolate SARS-CoV-2/human/France/50005JM/2020, co	TTCACCGAGGCCACGCGGAGTACGATCGAGTGTACAGTGAA
MT470152.1 Severe acute respiratory syndrome coronavirus 2 isolate SARS-CoV-2/human/France/50004CS/2020, com	TTCACCGAGGCCACGCGGAGTACGATCGAGTGTACAGTGAA
MT470153.1 Severe acute respiratory syndrome coronavirus 2 isolate SARS-CoV-2/human/France/10016DC/2020, co	TTCACCGAGGCCACGCGGAGTACGATCGAGTGTACAGTGAA
MT470154.1 Severe acute respiratory syndrome coronavirus 2 isolate SARS-CoV-2/human/France/10015BY/2020, com	TTCACCGAGGCCACGCGGAGTACGATCGAGTGTACAGTGAA
MT470155.1 Severe acute respiratory syndrome coronavirus 2 isolate SARS-CoV-2/human/France/10012BM/2020, co	TTCACCGAGGCCACGCGGAGTACGATCGAGTGTACAGTGAA
MT470156.1 Severe acute respiratory syndrome coronavirus 2 isolate SARS-CoV-2/human/France/10017GS/2020, com	TTCACCGAGGCCACGCGGAGTACGATCGAGTGTACAGTGAA
MT470157.1 Severe acute respiratory syndrome coronavirus 2 isolate SARS-CoV-2/human/France/10013BA/2020, co	TTCACCGAGGCCACGCGGAGTACGATCGAGTGTACAGTGAA
MT470158.1 Severe acute respiratory syndrome coronavirus 2 isolate SARS-CoV-2/human/France/10021SB/2020, com	TTCACCGAGGCCACGCGGAGTACGATCGAGTGTACAGTGAA
MT470159.1 Severe acute respiratory syndrome coronavirus 2 isolate SARS-CoV-2/human/France/10011AS/2020, com	TTCACCGAGGCCACGCGGAGTACGATCGAGTGTACAGTGAA
MT470160.1 Severe acute respiratory syndrome coronavirus 2 isolate SARS-CoV-2/human/France/10023FD/2020, com	TTCACCGAGGCCACGCGGAGTACGATCGAGTGTACAGTGAA
MT470161.1 Severe acute respiratory syndrome coronavirus 2 isolate SARS-CoV-2/human/France/10022AR/2020, co	TTCACCGAGGCCACGCGGAGTACGATCGAGTGTACAGTGAA
MT470162.1 Severe acute respiratory syndrome coronavirus 2 isolate SARS-CoV-2/human/France/50011LM/2020, co	TTCACCGAGGCCACGCGGAGTACGATCGAGTGTACAGTGAA

MT470163.1 Severe acute respiratory syndrome coronavirus 2 isolate SARS-CoV-2/human/France/10001DM/2020, co	TTCACCGAGGCCACGCGGAGTACGATCGAGTGTACAGTGAA
MT470164.1 Severe acute respiratory syndrome coronavirus 2 isolate SARS-CoV-2/human/France/50007PI/2020, com	TTCACCGAGGCCACGCGGAGTACGATCGAGTGTACAGTGAA
MT470165.1 Severe acute respiratory syndrome coronavirus 2 isolate SARS-CoV-2/human/France/10003SN/2020, co	TTCACCGAGGCCACGCGGAGTACGATCGAGTGTACAGTGAA
MT470166.1 Severe acute respiratory syndrome coronavirus 2 isolate SARS-CoV-2/human/France/10025GM/2020, co	TTCACCGAGGCCACGCGGAGTACGATCGAGTGTACAGTGAA
MT470167.1 Severe acute respiratory syndrome coronavirus 2 isolate SARS-CoV-2/human/France/10002PM/2020, co	TTCACCGAGGCCACGCGGAGTACGATCGAGTGTACAGTGAA
MT470168.1 Severe acute respiratory syndrome coronavirus 2 isolate SARS-CoV-2/human/France/10026SL/2020, cor	TTCACCGAGGCCACGCGGAGTACGATCGAGTGTACAGTGAA
MT470169.1 Severe acute respiratory syndrome coronavirus 2 isolate SARS-CoV-2/human/France/30001MB/2020, co	TTCACCGAGGCCACGCGGAGTACGATCGAGTGTACAGTGAA
MT470170.1 Severe acute respiratory syndrome coronavirus 2 isolate SARS-CoV-2/human/France/40003KA/2020, co	TTCACCGAGGCCACGCGGAGTACGATCGAGTGTACAGTGAA
MT470171.1 Severe acute respiratory syndrome coronavirus 2 isolate SARS-CoV-2/human/France/10007LJ/2020, com	TTCACCGAGGCCACGCGGAGTACGATCGAGTGTACAGTGAA
MT470172.1 Severe acute respiratory syndrome coronavirus 2 isolate SARS-CoV-2/human/France/10028CD/2020, co	TTCACCGAGGCCACGCGGAGTACGATCGAGTGTACAGTGAA
MT470173.1 Severe acute respiratory syndrome coronavirus 2 isolate SARS-CoV-2/human/France/10006HC/2020, co	TTCACCGAGGCCACGCGGAGTACGATCGAGTGTACAGTGAA
MT470174.1 Severe acute respiratory syndrome coronavirus 2 isolate SARS-CoV-2/human/France/10053BP/2020, co	TTCACCGAGGCCACGCGGAGTACGATCGAGTGTACAGTGAA
MT470175.1 Severe acute respiratory syndrome coronavirus 2 isolate SARS-CoV-2/human/France/10064DR/2020, co	TTCACCGAGGCCACGCGGAGTACGATCGAGTGTACAGTGAA
MT470176.1 Severe acute respiratory syndrome coronavirus 2 isolate SARS-CoV-2/human/France/10065EE/2020, cor	TTCACCGAGGCCACGCGGAGTACGATCGAGTGTACAGTGAA
MT470177.1 Severe acute respiratory syndrome coronavirus 2 isolate SARS-CoV-2/human/France/10070SK/2020, cor	TTCACCGAGGCCACGCGGAGTACGATCGAGTGTACAGTGAA
MT470178.1 Severe acute respiratory syndrome coronavirus 2 isolate SARS-CoV-2/human/France/50006PT/2020, cor	TTCACCGAGGCCACGCGGAGTACGATCGAGTGTACAGTGAA
MT470179.1 Severe acute respiratory syndrome coronavirus 2 isolate SARS-CoV-2/human/France/40002VJ/2020, cor	TTCACCGAGGCCACGCGGAGTACGATCGAGTGTACAGTGAA
MT470219.1 Severe acute respiratory syndrome coronavirus 2 isolate SARS-CoV-2/human/COL/Cali-01/2020, comple	TTCACCGAGGCCACGCGGAGTACGATCGAGTGTACAGTGAA
MT472621.1 Severe acute respiratory syndrome coronavirus 2 isolate SARS-CoV-2/human/USA/PA-CDC-2908/2020, c	TTCACCGAGGCCACGCGGAGTACGATCGAGTGTACAGTGAA
MT472622.1 Severe acute respiratory syndrome coronavirus 2 isolate SARS-CoV-2/human/USA/MD-CDC-0025/2020,	TTCACCGAGGCCACGCGGAGTACGATCGAGTGTACAGTGAA
MT472623.1 Severe acute respiratory syndrome coronavirus 2 isolate SARS-CoV-2/human/USA/VT-CDC-0303/2020, c	TTCACCGAGGCCACGCGGAGTACGATCGAGTGTACAGTGAA
MT472624.1 Severe acute respiratory syndrome coronavirus 2 isolate SARS-CoV-2/human/USA/FL-CDC-7619/2020, co	TTCACCGAGGCCACGCGGAGTACGATCGAGTGTACAGTGAA
MT472625.1 Severe acute respiratory syndrome coronavirus 2 isolate SARS-CoV-2/human/USA/PR-CDC-3578/2020, c	TTCACCGAGGCCACGCGGAGTACGATCGAGTGTACAGTGAA
MT472626.1 Severe acute respiratory syndrome coronavirus 2 isolate SARS-CoV-2/human/USA/IA-CDC-8200/2020, co	TTCACCGAGGCCACGCGGAGTACGATCGAGTGTACAGTGAA
MT472627.1 Severe acute respiratory syndrome coronavirus 2 isolate SARS-CoV-2/human/USA/DC-CDC-0019/2020, c	TTCACCGAGGCCACGCGGAGTACGATCGAGTGTACAGTGAA
MT474126.1 Severe acute respiratory syndrome coronavirus 2 isolate SARS-CoV-2/human/USA/CA-CZB-1121/2020, c	TTCACCGAGGCCACGCGGAGTACGATCGAGTGTACAGTGAA
MT474127.1 Severe acute respiratory syndrome coronavirus 2 isolate SARS-CoV-2/human/USA/CA-CZB-1122/2020, c	TTCACCGAGGCCACGCGGAGTACGATCGAGTGTACAGTGAA
MT474128.1 Severe acute respiratory syndrome coronavirus 2 isolate SARS-CoV-2/human/USA/CA-CZB-1123/2020, c	TTCACCGAGGCCACGCGGAGTACGATCGAGTGTACAGTGAA
MT474129.1 Severe acute respiratory syndrome coronavirus 2 isolate SARS-CoV-2/human/USA/CA-CZB-1124/2020, c	TTCACCGAGGCCACGCGGAGTACGATCGAGTGTACAGTGAA
MT474130.1 Severe acute respiratory syndrome coronavirus 2 isolate SARS-CoV-2/human/USA/CA-CZB-1125/2020, c	TTCACCGAGGCCACGCGGAGTACGATCGAGTGTACAGTGAA
MT474131.1 Severe acute respiratory syndrome coronavirus 2 isolate SARS-CoV-2/human/USA/CA-CZB-1126/2020 O	TTCACCGAGGCCACGCGGAGTACGATCGAGTGTACAGTGAA
MT474132.1 Severe acute respiratory syndrome coronavirus 2 isolate SARS-CoV-2/human/USA/CA-CZB-1127/2020, c	TTCACCGAGGCCACGCGGAGTACGATCGAGTGTACAGTGAA
MT474133.1 Severe acute respiratory syndrome coronavirus 2 isolate SARS-CoV-2/human/USA/CA-CZB-1128/2020, c	TTCACCGAGGCCACGCGGAGTACGATCGAGTGTACAGTGAA

MT474134.1 Severe acute respiratory syndrome coronavirus 2 isolate SARS-CoV-2/human/USA/CA-CZB-1129/2020, c	TTCACCGAGGCCACGCGGAGTACGATCGAGTGTACAGTGAA
MT474135.1 Severe acute respiratory syndrome coronavirus 2 isolate SARS-CoV-2/human/USA/CA-CZB-1131/2020, c	TTCACCGAGGCCACGCGGAGTACGATCGAGTGTACAGTGAA
MT474136.1 Severe acute respiratory syndrome coronavirus 2 isolate SARS-CoV-2/human/USA/CA-CZB-1132/2020, c	TTCACCGAGGCCACGCGGAGTACGATCGAGTGTACAGTGAA
MT474137.1 Severe acute respiratory syndrome coronavirus 2 isolate SARS-CoV-2/human/USA/CA-CZB-1133/2020, c	TTCACCGAGGCCACGCGGAGTACGATCGAGTGTACAGTGAA
MT474138.1 Severe acute respiratory syndrome coronavirus 2 isolate SARS-CoV-2/human/USA/CA-CZB-1134/2020, c	TTCACCGAGGCCACGCGGAGTACGATCGAGTGTACAGTGAA
MT476384.1 Severe acute respiratory syndrome coronavirus 2 isolate SARS-CoV-2/ENV/USA/UF-11/2020, complete g	TTCACCGAGGCCACGCGGAGTACGATCGAGTGTACAGTGAA
MT476385.1 Severe acute respiratory syndrome coronavirus 2 isolate SARS-CoV-2/human/BGD/CHRF_0001/2020, co	TTCACCGAGGCCACGCGGAGTACGATCGAGTGTACAGTGAA
MT477835.1 Severe acute respiratory syndrome coronavirus 2 isolate SARS-CoV-2/human/USA/AK003/2020, complet	TTCACCGAGGCCACGCGGAGTACGATCGAGTGTACAGTGAA
MT477836.1 Severe acute respiratory syndrome coronavirus 2 isolate SARS-CoV-2/human/USA/AK006/2020, complet	TTCACCGAGGCCACGCGGAGTACGATCGAGTGTACAGTGAA
MT477837.1 Severe acute respiratory syndrome coronavirus 2 isolate SARS-CoV-2/human/USA/AK010/2020, complet	TTCACCGAGGCCACGCGGAGTACGATCGAGTGTACAGTGAA
MT477838.1 Severe acute respiratory syndrome coronavirus 2 isolate SARS-CoV-2/human/USA/AK011/2020, complet	TTCACCGAGGCCACGCGGAGTACGATCGAGTGTACAGTGAA
MT477839.1 Severe acute respiratory syndrome coronavirus 2 isolate SARS-CoV-2/human/USA/AK013/2020, complet	TTCACCGAGGCCACGCGGAGTACGATCGAGTGTACAGTGAA
MT477840.1 Severe acute respiratory syndrome coronavirus 2 isolate SARS-CoV-2/human/USA/AK015/2020, complet	TTCACCGAGGCCACGCGGAGTACGATCGAGTGTACAGTGAA
MT477841.1 Severe acute respiratory syndrome coronavirus 2 isolate SARS-CoV-2/human/USA/AK016/2020, complet	TTCACCGAGGCCACGCGGAGTACGATCGAGTGTACAGTGAA
MT477842.1 Severe acute respiratory syndrome coronavirus 2 isolate SARS-CoV-2/human/USA/AK034/2020, complet	TTCACCGAGGCCACGCGGAGTACGATCGAGTGTACAGTGAA
MT477843.1 Severe acute respiratory syndrome coronavirus 2 isolate SARS-CoV-2/human/USA/AK037/2020, complet	TTCACCGAGGCCACGCGGAGTACGATCGAGTGTACAGTGAA
MT477844.1 Severe acute respiratory syndrome coronavirus 2 isolate SARS-CoV-2/human/USA/AK040/2020, complet	TTCACCGAGGCCACGCGGAGTACGATCGAGTGTACAGTGAA
MT477845.1 Severe acute respiratory syndrome coronavirus 2 isolate SARS-CoV-2/human/USA/AK041/2020, complet	TTCACCGAGGCCACGCGGAGTACGATCGAGTGTACAGTGAA
MT477846.1 Severe acute respiratory syndrome coronavirus 2 isolate SARS-CoV-2/human/USA/AK052/2020, complet	TTCACCGAGGCCACGCGGAGTACGATCGAGTGTACAGTGAA
MT477847.1 Severe acute respiratory syndrome coronavirus 2 isolate SARS-CoV-2/human/USA/AK070/2020, complet	TTCACCGAGGCCACGCGGAGTACGATCGAGTGTACAGTGAA
MT477848.1 Severe acute respiratory syndrome coronavirus 2 isolate SARS-CoV-2/human/USA/AK074/2020, complet	TTCACCGAGGCCACGCGGAGTACGATCGAGTGTACAGTGAA
MT477849.1 Severe acute respiratory syndrome coronavirus 2 isolate SARS-CoV-2/human/USA/AK077/2020, complet	TTCACCGAGGCCACGCGGAGTACGATCGAGTGTACAGTGAA
MT477850.1 Severe acute respiratory syndrome coronavirus 2 isolate SARS-CoV-2/human/USA/AK092/2020, complet	TTCACCGAGGCCACGCGGAGTACGATCGAGTGTACAGTGAA
MT477851.1 Severe acute respiratory syndrome coronavirus 2 isolate SARS-CoV-2/human/USA/AK101/2020, complet	TTCACCGAGGCCACGCGGAGTACGATCGAGTGTACAGTGAA
MT477852.1 Severe acute respiratory syndrome coronavirus 2 isolate SARS-CoV-2/human/USA/AK115/2020, complet	TTCACCGAGGCCACGCGGAGTACGATCGAGTGTACAGTGAA
MT477853.1 Severe acute respiratory syndrome coronavirus 2 isolate SARS-CoV-2/human/USA/AK118/2020, complet	TTCACCGAGGCCACGCGGAGTACGATCGAGTGTACAGTGAA
MT477854.1 Severe acute respiratory syndrome coronavirus 2 isolate SARS-CoV-2/human/USA/AK122/2020, complet	TTCACCGAGGCCACGCGGAGTACGATCGAGTGTACAGTGAA
MT477855.1 Severe acute respiratory syndrome coronavirus 2 isolate SARS-CoV-2/human/USA/AK151/2020, complet	TTCACCGAGGCCACGCGGAGTACGATCGAGTGTACAGTGAA
MT477856.1 Severe acute respiratory syndrome coronavirus 2 isolate SARS-CoV-2/human/USA/AK152/2020, complet	TTCACCGAGGCCACGCGGAGTACGATCGAGTGTACAGTGAA
MT477857.1 Severe acute respiratory syndrome coronavirus 2 isolate SARS-CoV-2/human/USA/AK153/2020, complet	TTCACCGAGGCCACGCGGAGTACGATCGAGTGTACAGTGAA
MT477858.1 Severe acute respiratory syndrome coronavirus 2 isolate SARS-CoV-2/human/USA/AK178/2020, complet	TTCACCGAGGCCACGCGGAGTACGATCGAGTGTACAGTGAA
MT477859.1 Severe acute respiratory syndrome coronavirus 2 isolate SARS-CoV-2/human/USA/AK180/2020, complet	TTCACCGAGGCCACGCGGAGTACGATCGAGTGTACAGTGAA
MT477860.1 Severe acute respiratory syndrome coronavirus 2 isolate SARS-CoV-2/human/USA/AK193/2020, complet	TTCACCGAGGCCACGCGGAGTACGATCGAGTGTACAGTGAA

MT477861.1 Severe acute respiratory syndrome coronavirus 2 isolate SARS-CoV-2/human/USA/AK200/2020, complet	TTCACCGAGGCCACGCGGAGTACGATCGAGTGTACAGTGAA
MT477862.1 Severe acute respiratory syndrome coronavirus 2 isolate SARS-CoV-2/human/USA/AK283/2020, complet	TTCACCGAGGCCACGCGGAGTACGATCGAGTGTACAGTGAA
MT477885.1 Severe acute respiratory syndrome coronavirus 2 isolate SARS-CoV-2/human/IND/OUMRK1090/2020, co	TTCACCGAGGCCACGCGGAGTACGATCGAGTGTACAGTGAA
MT477903.1 Severe acute respiratory syndrome coronavirus 2 isolate SARS-CoV-2/human/USA/UF-10/2020, complet	TTCACCGAGGCCACGCGGAGTACGATCGAGTGTACAGTGAA
MT477904.1 Severe acute respiratory syndrome coronavirus 2 isolate SARS-CoV-2/human/USA/UF-9/2020, complete	TTCACCGAGGCCACGCGGAGTACGATCGAGTGTACAGTGAA
MT481895.1 Severe acute respiratory syndrome coronavirus 2 isolate SARS-CoV-2/human/IND/GBRC65a/2020, comp	TTCACCGAGGCCACGCGGAGTACGATCGAGTGTACAGTGAA
MT481896.1 Severe acute respiratory syndrome coronavirus 2 isolate SARS-CoV-2/human/IND/GBRC65b/2020, comp	TTCACCGAGGCCACGCGGAGTACGATCGAGTGTACAGTGAA
MT481897.1 Severe acute respiratory syndrome coronavirus 2 isolate SARS-CoV-2/human/IND/GBRC76/2020, compl	TTCACCGAGGCCACGCGGAGTACGATCGAGTGTACAGTGAA
MT481898.1 Severe acute respiratory syndrome coronavirus 2 isolate SARS-CoV-2/human/IND/GBRC85/2020, compl	TTCACCGAGGCCACGCGGAGTACGATCGAGTGTACAGTGAA
MT481899.1 Severe acute respiratory syndrome coronavirus 2 isolate SARS-CoV-2/human/IND/GBRC77/2020, compl	TTCACCGAGGCCACGCGGAGTACGATCGAGTGTACAGTGAA
MT481900.1 Severe acute respiratory syndrome coronavirus 2 isolate SARS-CoV-2/human/IND/GBRC86/2020, compl	TTCACCGAGGCCACGCGGAGTACGATCGAGTGTACAGTGAA
MT481901.1 Severe acute respiratory syndrome coronavirus 2 isolate SARS-CoV-2/human/IND/GBRC87/2020, compl	TTCACCGAGGCCACGCGGAGTACGATCGAGTGTACAGTGAA
MT481902.1 Severe acute respiratory syndrome coronavirus 2 isolate SARS-CoV-2/human/IND/GBRC88a/2020, comp	TTCACCGAGGCCACGCGGAGTACGATCGAGTGTACAGTGAA
MT481903.1 Severe acute respiratory syndrome coronavirus 2 isolate SARS-CoV-2/human/IND/GBRC88b/2020, comp	TTCACCGAGGCCACGCGGAGTACGATCGAGTGTACAGTGAA
MT481904.1 Severe acute respiratory syndrome coronavirus 2 isolate SARS-CoV-2/human/IND/GBRC89/2020, compl	TTCACCGAGGCCACGCGGAGTACGATCGAGTGTACAGTGAA
MT481905.1 Severe acute respiratory syndrome coronavirus 2 isolate SARS-CoV-2/human/IND/GBRC90/2020, compl	TTCACCGAGGCCACGCGGAGTACGATCGAGTGTACAGTGAA
MT481906.1 Severe acute respiratory syndrome coronavirus 2 isolate SARS-CoV-2/human/IND/GBRC91/2020, compl	TTCACCGAGGCCACGCGGAGTACGATCGAGTGTACAGTGAA
MT481907.1 Severe acute respiratory syndrome coronavirus 2 isolate SARS-CoV-2/human/IND/GBRC92/2020, compl	TTCACCGAGGCCACGCGGAGTACGATCGAGTGTACAGTGAA
MT481908.1 Severe acute respiratory syndrome coronavirus 2 isolate SARS-CoV-2/human/IND/GBRC93/2020, compl	TTCACCGAGGCCACGCGGAGTACGATCGAGTGTACAGTGAA
MT481927.1 Severe acute respiratory syndrome coronavirus 2 isolate SARS-CoV-2/human/USA/CA-CZB-1136/2020, c	TTCACCGAGGCCACGCGGAGTACGATCGAGTGTACAGTGAA
MT481928.1 Severe acute respiratory syndrome coronavirus 2 isolate SARS-CoV-2/human/USA/CA-CZB-1137/2020, c	TTCACCGAGGCCACGCGGAGTACGATCGAGTGTACAGTGAA
MT481929.1 Severe acute respiratory syndrome coronavirus 2 isolate SARS-CoV-2/human/USA/CA-CZB-1138/2020, c	TTCACCGAGGCCACGCGGAGTACGATCGAGTGTACAGTGAA
MT481930.1 Severe acute respiratory syndrome coronavirus 2 isolate SARS-CoV-2/human/USA/CA-CZB-1139/2020, c	TTCACCGAGGCCACGCGGAGTACGATCGAGTGTACAGTGAA
MT481931.1 Severe acute respiratory syndrome coronavirus 2 isolate SARS-CoV-2/human/USA/CA-CZB-1140/2020, c	TTCACCGAGGCCACGCGGAGTACGATCGAGTGTACAGTGAA
MT481932.1 Severe acute respiratory syndrome coronavirus 2 isolate SARS-CoV-2/human/USA/CA-CZB-1141/2020, c	TTCACCGAGGCCACGCGGAGTACGATCGAGTGTACAGTGAA
MT481933.1 Severe acute respiratory syndrome coronavirus 2 isolate SARS-CoV-2/human/USA/CA-CZB-1142/2020, c	TTCACCGAGGCCACGCGGAGTACGATCGAGTGTACAGTGAA
MT481934.1 Severe acute respiratory syndrome coronavirus 2 isolate SARS-CoV-2/human/USA/CA-CZB-1143/2020, c	TTCACCGAGGCCACGCGGAGTACGATCGAGTGTACAGTGAA
MT481935.1 Severe acute respiratory syndrome coronavirus 2 isolate SARS-CoV-2/human/USA/CA-CZB-1144/2020, c	TTCACCGAGGCCACGCGGAGTACGATCGAGTGTACAGTGAA
MT481936.1 Severe acute respiratory syndrome coronavirus 2 isolate SARS-CoV-2/human/USA/CA-CZB-1145/2020, c	TTCACCGAGGCCACGCGGAGTACGATCGAGTGTACAGTGAA
MT481992.1 Severe acute respiratory syndrome coronavirus 2 isolate SARS-CoV-2/human/USA/CA-CDC-0139/2020, c	TTCACCGAGGCCACGCGGAGTACGATCGAGTGTACAGTGAA
MT482111.1 Severe acute respiratory syndrome coronavirus 2 isolate SARS-CoV-2/human/USA/VA-DCLS-0120/2020,	TTCACCGAGGCCACGCGGAGTACGATCGAGTGTACAGTGAA
MT482112.1 Severe acute respiratory syndrome coronavirus 2 isolate SARS-CoV-2/human/USA/VA-DCLS-0126/2020 (	TTCACCGAGGCCACGCGGAGTACGATCGAGTGTACAGTGAA
MT482116.1 Severe acute respiratory syndrome coronavirus 2 isolate SARS-CoV-2/human/USA/VA-DCLS-0130/2020,	TTCACCGAGGCCACGCGGAGTACGATCGAGTGTACAGTGAA

MT482118.1 Severe acute respiratory syndrome coronavirus 2 isolate SARS-CoV-2/human/USA/VA-DCLS-0132/2020,	TTCACCGAGGCCACGCGGAGTACGATCGAGTGTACAGTGAA
MT482120.1 Severe acute respiratory syndrome coronavirus 2 isolate SARS-CoV-2/human/USA/VA-DCLS-0134/2020 (	TTCACCGAGGCCACGCGGAGTACGATCGAGTGTACAGTGAA
MT482122.1 Severe acute respiratory syndrome coronavirus 2 isolate SARS-CoV-2/human/USA/VA-DCLS-0136/2020,	TTCACCGAGGCCACGCGGAGTACGATCGAGTGTACAGTGAA
MT482128.1 Severe acute respiratory syndrome coronavirus 2 isolate SARS-CoV-2/human/USA/VA-DCLS-0142/2020,	TTCACCGAGGCCACGCGGAGTACGATCGAGTGTACAGTGAA
MT482131.1 Severe acute respiratory syndrome coronavirus 2 isolate SARS-CoV-2/human/USA/VA-DCLS-0151/2020,	TTCACCGAGGCCACGCGGAGTACGATCGAGTGTACAGTGAA
MT482132.1 Severe acute respiratory syndrome coronavirus 2 isolate SARS-CoV-2/human/USA/VA-DCLS-0152/2020,	TTCACCGAGGCCACGCGGAGTACGATCGAGTGTACAGTGAA
MT482133.1 Severe acute respiratory syndrome coronavirus 2 isolate SARS-CoV-2/human/USA/VA-DCLS-0153/2020,	TTCACCGAGGCCACGCGGAGTACGATCGAGTGTACAGTGAA
MT482134.1 Severe acute respiratory syndrome coronavirus 2 isolate SARS-CoV-2/human/USA/VA-DCLS-0154/2020,	TTCACCGAGGCCACGCGGAGTACGATCGAGTGTACAGTGAA
MT482138.1 Severe acute respiratory syndrome coronavirus 2 isolate SARS-CoV-2/human/USA/VA-DCLS-0158/2020,	TTCACCGAGGCCACGCGGAGTACGATCGAGTGTACAGTGAA
MT482140.1 Severe acute respiratory syndrome coronavirus 2 isolate SARS-CoV-2/human/USA/VA-DCLS-0160/2020,	TTCACCGAGGCCACGCGGAGTACGATCGAGTGTACAGTGAA
MT482143.1 Severe acute respiratory syndrome coronavirus 2 isolate SARS-CoV-2/human/USA/VA-DCLS-0163/2020,	TTCACCGAGGCCACGCGGAGTACGATCGAGTGTACAGTGAA
MT482144.1 Severe acute respiratory syndrome coronavirus 2 isolate SARS-CoV-2/human/USA/VA-DCLS-0164/2020,	TTCACCGAGGCCACGCGGAGTACGATCGAGTGTACAGTGAA
MT482145.1 Severe acute respiratory syndrome coronavirus 2 isolate SARS-CoV-2/human/USA/VA-DCLS-0165/2020,	TTCACCGAGGCCACGCGGAGTACGATCGAGTGTACAGTGAA
MT482147.1 Severe acute respiratory syndrome coronavirus 2 isolate SARS-CoV-2/human/USA/VA-DCLS-0167/2020,	TTCACCGAGGCCACGCGGAGTACGATCGAGTGTACAGTGAA
MT482149.1 Severe acute respiratory syndrome coronavirus 2 isolate SARS-CoV-2/human/USA/VA-DCLS-0169/2020,	TTCACCGAGGCCACGCGGAGTACGATCGAGTGTACAGTGAA
MT483553.1 Severe acute respiratory syndrome coronavirus 2 isolate SARS-CoV-2/human/IND/GBRC83/2020, comple	TTCACCGAGGCCACGCGGAGTACGATCGAGTGTACAGTGAA
MT483554.1 Severe acute respiratory syndrome coronavirus 2 isolate SARS-CoV-2/human/IND/GBRC81a/2020, comp	TTCACCGAGGCCACGCGGAGTACGATCGAGTGTACAGTGAA
MT483555.1 Severe acute respiratory syndrome coronavirus 2 isolate SARS-CoV-2/human/IND/GBRC79a/2020, comp	TTCACCGAGGCCACGCGGAGTACGATCGAGTGTACAGTGAA
MT483556.1 Severe acute respiratory syndrome coronavirus 2 isolate SARS-CoV-2/human/IND/GBRC82a/2020, comp	TTCACCGAGGCCACGCGGAGTACGATCGAGTGTACAGTGAA
MT483557.1 Severe acute respiratory syndrome coronavirus 2 isolate SARS-CoV-2/human/IND/GBRC79b/2020, comp	TTCACCGAGGCCACGCGGAGTACGATCGAGTGTACAGTGAA
MT483558.1 Severe acute respiratory syndrome coronavirus 2 isolate SARS-CoV-2/human/IND/GBRC82b/2020, comp	TTCACCGAGGCCACGCGGAGTACGATCGAGTGTACAGTGAA
MT483559.1 Severe acute respiratory syndrome coronavirus 2 isolate SARS-CoV-2/human/IND/GBRC84/2020, comple	TTCACCGAGGCCACGCGGAGTACGATCGAGTGTACAGTGAA
MT483560.1 Severe acute respiratory syndrome coronavirus 2 isolate SARS-CoV-2/human/IND/GBRC81b/2020, comp	TTCACCGAGGCCACGCGGAGTACGATCGAGTGTACAGTGAA
MT483563.1 Severe acute respiratory syndrome coronavirus 2 isolate SARS-CoV-2/human/USA/CA-CZB-1053/2020, c	TTCACCGAGGCCACGCGGAGTACGATCGAGTGTACAGTGAA
MT483564.1 Severe acute respiratory syndrome coronavirus 2 isolate SARS-CoV-2/human/USA/CA-CZB-1055/2020 O	TTCACCGAGGCCACGCGGAGTACGATCGAGTGTACAGTGAA
MT496972.1 Severe acute respiratory syndrome coronavirus 2 isolate SARS-CoV-2/human/IND/GBRC36/2020, comple	TTCACCGAGGCCACGCGGAGTACGATCGAGTGTACAGTGAA
MT496973.1 Severe acute respiratory syndrome coronavirus 2 isolate SARS-CoV-2/human/IND/GBRC37/2020, comple	TTCACCGAGGCCACGCGGAGTACGATCGAGTGTACAGTGAA
MT496974.1 Severe acute respiratory syndrome coronavirus 2 isolate SARS-CoV-2/human/IND/GBRC38/2020, comple	TTCACCGAGGCCACGCGGAGTACGATCGAGTGTACAGTGAA
MT496975.1 Severe acute respiratory syndrome coronavirus 2 isolate SARS-CoV-2/human/IND/GBRC39/2020, comple	TTCACCGAGGCCACGCGGAGTACGATCGAGTGTACAGTGAA
MT496976.1 Severe acute respiratory syndrome coronavirus 2 isolate SARS-CoV-2/human/IND/GBRC56a/2020, comp	TTCACCGAGGCCACGCGGAGTACGATCGAGTGTACAGTGAA
MT496977.1 Severe acute respiratory syndrome coronavirus 2 isolate SARS-CoV-2/human/IND/GBRC56b/2020, comp	TTCACCGAGGCCACGCGGAGTACGATCGAGTGTACAGTGAA
MT496978.1 Severe acute respiratory syndrome coronavirus 2 isolate SARS-CoV-2/human/IND/GBRC57/2020 ORF1ab	TTCACCGAGGCCACGCGGAGTACGATCGAGTGTACAGTGAA
MT496980.1 Severe acute respiratory syndrome coronavirus 2 isolate SARS-CoV-2/human/IND/GBRC58/2020, comple	TTCACCGAGGCCACGCGGAGTACGATCGAGTGTACAGTGAA

MT496981.1 Severe acute respiratory syndrome coronavirus 2 isolate SARS-CoV-2/human/IND/GBRC59/2020, compl	TTCACCGAGGCCACGCGGAGTACGATCGAGTGTACAGTGAA
MT496982.1 Severe acute respiratory syndrome coronavirus 2 isolate SARS-CoV-2/human/IND/GBRC60/2020, compl	TTCACCGAGGCCACGCGGAGTACGATCGAGTGTACAGTGAA
MT496983.1 Severe acute respiratory syndrome coronavirus 2 isolate SARS-CoV-2/human/IND/GBRC67a/2020, comp	TTCACCGAGGCCACGCGGAGTACGATCGAGTGTACAGTGAA
MT496984.1 Severe acute respiratory syndrome coronavirus 2 isolate SARS-CoV-2/human/IND/GBRC61/2020, compl	TTCACCGAGGCCACGCGGAGTACGATCGAGTGTACAGTGAA
MT496985.1 Severe acute respiratory syndrome coronavirus 2 isolate SARS-CoV-2/human/IND/GBRC67b/2020, comp	TTCACCGAGGCCACGCGGAGTACGATCGAGTGTACAGTGAA
MT496986.1 Severe acute respiratory syndrome coronavirus 2 isolate SARS-CoV-2/human/IND/GBRC68/2020, compl	TTCACCGAGGCCACGCGGAGTACGATCGAGTGTACAGTGAA
MT496987.1 Severe acute respiratory syndrome coronavirus 2 isolate SARS-CoV-2/human/IND/GBRC69/2020, compl	TTCACCGAGGCCACGCGGAGTACGATCGAGTGTACAGTGAA
MT496988.1 Severe acute respiratory syndrome coronavirus 2 isolate SARS-CoV-2/human/IND/GBRC62/2020, compl	TTCACCGAGGCCACGCGGAGTACGATCGAGTGTACAGTGAA
MT496989.1 Severe acute respiratory syndrome coronavirus 2 isolate SARS-CoV-2/human/IND/GBRC63/2020, compl	TTCACCGAGGCCACGCGGAGTACGATCGAGTGTACAGTGAA
MT496990.1 Severe acute respiratory syndrome coronavirus 2 isolate SARS-CoV-2/human/IND/GBRC70/2020, compl	TTCACCGAGGCCACGCGGAGTACGATCGAGTGTACAGTGAA
MT496991.1 Severe acute respiratory syndrome coronavirus 2 isolate SARS-CoV-2/human/IND/GBRC71/2020, compl	TTCACCGAGGCCACGCGGAGTACGATCGAGTGTACAGTGAA
MT496992.1 Severe acute respiratory syndrome coronavirus 2 isolate SARS-CoV-2/human/IND/GBRC64/2020, compl	TTCACCGAGGCCACGCGGAGTACGATCGAGTGTACAGTGAA
MT496993.1 Severe acute respiratory syndrome coronavirus 2 isolate SARS-CoV-2/human/IND/GBRC72a/2020, comp	TTCACCGAGGCCACGCGGAGTACGATCGAGTGTACAGTGAA
MT496994.1 Severe acute respiratory syndrome coronavirus 2 isolate SARS-CoV-2/human/IND/GBRC72b/2020, comp	TTCACCGAGGCCACGCGGAGTACGATCGAGTGTACAGTGAA
MT496995.1 Severe acute respiratory syndrome coronavirus 2 isolate SARS-CoV-2/human/IND/GBRC73/2020, compl	TTCACCGAGGCCACGCGGAGTACGATCGAGTGTACAGTGAA
MT496996.1 Severe acute respiratory syndrome coronavirus 2 isolate SARS-CoV-2/human/IND/GBRC74/2020, compl	TTCACCGAGGCCACGCGGAGTACGATCGAGTGTACAGTGAA
MT496997.1 Severe acute respiratory syndrome coronavirus 2 isolate SARS-CoV-2/human/IND/GBRC75/2020, compl	TTCACCGAGGCCACGCGGAGTACGATCGAGTGTACAGTGAA
MT499171.1 Severe acute respiratory syndrome coronavirus 2 isolate SARS-CoV-2/human/USA/CA-CZB-1196/2020, c	TTCACCGAGGCCACGCGGAGTACGATCGAGTGTACAGTGAA
MT499172.1 Severe acute respiratory syndrome coronavirus 2 isolate SARS-CoV-2/human/USA/CA-CZB-1197/2020, c	TTCACCGAGGCCACGCGGAGTACGATCGAGTGTACAGTGAA
MT499173.1 Severe acute respiratory syndrome coronavirus 2 isolate SARS-CoV-2/human/USA/CA-CZB-1198/2020, c	TTCACCGAGGCCACGCGGAGTACGATCGAGTGTACAGTGAA
MT499174.1 Severe acute respiratory syndrome coronavirus 2 isolate SARS-CoV-2/human/USA/CA-CZB-1199/2020, c	TTCACCGAGGCCACGCGGAGTACGATCGAGTGTACAGTGAA
MT499175.1 Severe acute respiratory syndrome coronavirus 2 isolate SARS-CoV-2/human/USA/CA-CZB-1200/2020, c	TTCACCGAGGCCACGCGGAGTACGATCGAGTGTACAGTGAA
MT499176.1 Severe acute respiratory syndrome coronavirus 2 isolate SARS-CoV-2/human/USA/CA-CZB-1201/2020, c	TTCACCGAGGCCACGCGGAGTACGATCGAGTGTACAGTGAA
MT499177.1 Severe acute respiratory syndrome coronavirus 2 isolate SARS-CoV-2/human/USA/CA-CZB-1202/2020, c	TTCACCGAGGCCACGCGGAGTACGATCGAGTGTACAGTGAA
MT499179.1 Severe acute respiratory syndrome coronavirus 2 isolate SARS-CoV-2/human/USA/CA-CZB-1204/2020, c	TTCACCGAGGCCACGCGGAGTACGATCGAGTGTACAGTGAA
MT499180.1 Severe acute respiratory syndrome coronavirus 2 isolate SARS-CoV-2/human/USA/CA-CZB-1205/2020, c	TTCACCGAGGCCACGCGGAGTACGATCGAGTGTACAGTGAA
MT499181.1 Severe acute respiratory syndrome coronavirus 2 isolate SARS-CoV-2/human/USA/CA-CZB-1206/2020, c	TTCACCGAGGCCACGCGGAGTACGATCGAGTGTACAGTGAA
MT499182.1 Severe acute respiratory syndrome coronavirus 2 isolate SARS-CoV-2/human/USA/CA-CZB-1207/2020, c	TTCACCGAGGCCACGCGGAGTACGATCGAGTGTACAGTGAA
MT499183.1 Severe acute respiratory syndrome coronavirus 2 isolate SARS-CoV-2/human/USA/CA-CZB-1208/2020, c	TTCACCGAGGCCACGCGGAGTACGATCGAGTGTACAGTGAA
MT499184.1 Severe acute respiratory syndrome coronavirus 2 isolate SARS-CoV-2/human/USA/CA-CZB-1209/2020, c	TTCACCGAGGCCACGCGGAGTACGATCGAGTGTACAGTGAA
MT499185.1 Severe acute respiratory syndrome coronavirus 2 isolate SARS-CoV-2/human/USA/CA-CZB-1210/2020, c	TTCACCGAGGCCACGCGGAGTACGATCGAGTGTACAGTGAA
MT499186.1 Severe acute respiratory syndrome coronavirus 2 isolate SARS-CoV-2/human/USA/CA-CZB-1211/2020, c	TTCACCGAGGCCACGCGGAGTACGATCGAGTGTACAGTGAA
MT499187.1 Severe acute respiratory syndrome coronavirus 2 isolate SARS-CoV-2/human/USA/CA-CZB-1212/2020, c	TTCACCGAGGCCACGCGGAGTACGATCGAGTGTACAGTGAA

MT499188.1 Severe acute respiratory syndrome coronavirus 2 isolate SARS-CoV-2/human/USA/CA-CZB-1213/2020, c	TTCACCGAGGCCACGCGGAGTACGATCGAGTGTACAGTGAA
MT499189.1 Severe acute respiratory syndrome coronavirus 2 isolate SARS-CoV-2/human/USA/CA-CZB-1214/2020, c	TTCACCGAGGCCACGCGGAGTACGATCGAGTGTACAGTGAA
MT499190.1 Severe acute respiratory syndrome coronavirus 2 isolate SARS-CoV-2/human/USA/CA-CZB-1215/2020, c	TTCACCGAGGCCACGCGGAGTACGATCGAGTGTACAGTGAA
MT499191.1 Severe acute respiratory syndrome coronavirus 2 isolate SARS-CoV-2/human/USA/CA-CZB-1216/2020, c	TTCACCGAGGCCACGCGGAGTACGATCGAGTGTACAGTGAA
MT499192.1 Severe acute respiratory syndrome coronavirus 2 isolate SARS-CoV-2/human/USA/CA-CZB-1217/2020, c	TTCACCGAGGCCACGCGGAGTACGATCGAGTGTACAGTGAA
MT499193.1 Severe acute respiratory syndrome coronavirus 2 isolate SARS-CoV-2/human/USA/CA-CZB-1218/2020, c	TTCACCGAGGCCACGCGGAGTACGATCGAGTGTACAGTGAA
MT499194.1 Severe acute respiratory syndrome coronavirus 2 isolate SARS-CoV-2/human/USA/CA-CZB-1219/2020, c	TTCACCGAGGCCACGCGGAGTACGATCGAGTGTACAGTGAA
MT499195.1 Severe acute respiratory syndrome coronavirus 2 isolate SARS-CoV-2/human/USA/CA-CZB-1220/2020, c	TTCACCGAGGCCACGCGGAGTACGATCGAGTGTACAGTGAA
MT499196.1 Severe acute respiratory syndrome coronavirus 2 isolate SARS-CoV-2/human/USA/CA-CZB-1221/2020, c	TTCACCGAGGCCACGCGGAGTACGATCGAGTGTACAGTGAA
MT499197.1 Severe acute respiratory syndrome coronavirus 2 isolate SARS-CoV-2/human/USA/CA-CZB-1222/2020, c	TTCACCGAGGCCACGCGGAGTACGATCGAGTGTACAGTGAA
MT499198.1 Severe acute respiratory syndrome coronavirus 2 isolate SARS-CoV-2/human/USA/CA-CZB-1223/2020, c	TTCACCGAGGCCACGCGGAGTACGATCGAGTGTACAGTGAA
MT499199.1 Severe acute respiratory syndrome coronavirus 2 isolate SARS-CoV-2/human/USA/CA-CZB-1224/2020, c	TTCACCGAGGCCACGCGGAGTACGATCGAGTGTACAGTGAA
MT499200.1 Severe acute respiratory syndrome coronavirus 2 isolate SARS-CoV-2/human/USA/CA-CZB-1225/2020, c	TTCACCGAGGCCACGCGGAGTACGATCGAGTGTACAGTGAA
MT499201.1 Severe acute respiratory syndrome coronavirus 2 isolate SARS-CoV-2/human/USA/CA-CZB-1226/2020, c	TTCACCGAGGCCACGCGGAGTACGATCGAGTGTACAGTGAA
MT499202.1 Severe acute respiratory syndrome coronavirus 2 isolate SARS-CoV-2/human/USA/CA-CZB-1227/2020, c	TTCACCGAGGCCACGCGGAGTACGATCGAGTGTACAGTGAA
MT499203.1 Severe acute respiratory syndrome coronavirus 2 isolate SARS-CoV-2/human/USA/CA-CZB-1228/2020, c	TTCACCGAGGCCACACGGAGTACGATCGAGTGTACAGTGAA
MT499204.1 Severe acute respiratory syndrome coronavirus 2 isolate SARS-CoV-2/human/USA/CA-CZB-1229/2020, c	TTCACCGAGGCCACGCGGAGTACGATCGAGTGTACAGTGAA
MT499205.1 Severe acute respiratory syndrome coronavirus 2 isolate SARS-CoV-2/human/USA/CA-CZB-1230/2020, c	TTCACCGAGGCCACGCGGAGTACGATCGAGTGTACAGTGAA
MT499206.1 Severe acute respiratory syndrome coronavirus 2 isolate SARS-CoV-2/human/USA/CA-CZB-1231/2020, c	TTCACCGAGGCCACGCGGAGTACGATCGAGTGTACAGTGAA
MT499207.1 Severe acute respiratory syndrome coronavirus 2 isolate SARS-CoV-2/human/USA/CA-CZB-1232/2020, c	TTCACCGAGGCCACGCGGAGTACGATCGAGTGTACAGTGAA
MT499208.1 Severe acute respiratory syndrome coronavirus 2 isolate SARS-CoV-2/human/POL/IHG_PAS_1_77/2020,	TTCACCGAGGCCACGCGGAGTACGATCGAGTGTACAGTGAA
MT499209.1 Severe acute respiratory syndrome coronavirus 2 isolate SARS-CoV-2/human/POL/IHG_PAS_1_69/2020,	TTCACCGAGGCCACGCGGAGTACGATCGAGTGTACAGTGAA
MT500122.1 Severe acute respiratory syndrome coronavirus 2 isolate SARS-CoV-2/human/PAK/KHI1/2020, complete	TTCACCGAGGCCACGCGGAGTACGATCGAGTGTACAGTGAA
MT502774.1 Severe acute respiratory syndrome coronavirus 2 isolate SARS-CoV-2/Human/BGD/BARJ_CVASU_CTG_5	TTCACCGAGGCCACACGGAGTACGATCGAGTGTACAGTGAA
MT506199.1 Severe acute respiratory syndrome coronavirus 2 isolate SARS-CoV-2/human/USA/MI-MDHHS-SC20359/	TTCACCGAGGCCACGCGGAGTACGATCGAGTGTACAGTGAA
MT506200.1 Severe acute respiratory syndrome coronavirus 2 isolate SARS-CoV-2/human/USA/MI-MDHHS-SC20360/	TTCACCGAGGCCACGCGGAGTACGATCGAGTGTACAGTGAA
MT506202.1 Severe acute respiratory syndrome coronavirus 2 isolate SARS-CoV-2/human/USA/MI-MDHHS-SC20362/	TTCACCGAGGCCACGCGGAGTACGATCGAGTGTACAGTGAA
MT506203.1 Severe acute respiratory syndrome coronavirus 2 isolate SARS-CoV-2/human/USA/MI-MDHHS-SC20363/	TTCACCGAGGCCACGCGGAGTACGATCGAGTGTACAGTGAA
MT506205.1 Severe acute respiratory syndrome coronavirus 2 isolate SARS-CoV-2/human/USA/MI-MDHHS-SC20365/	TTCACCGAGGCCACGCGGAGTACGATCGAGTGTACAGTGAA
MT506206.1 Severe acute respiratory syndrome coronavirus 2 isolate SARS-CoV-2/human/USA/MI-MDHHS-SC20366/	TTCACCGAGGCCACGCGGAGTACGATCGAGTGTACAGTGAA
MT506207.1 Severe acute respiratory syndrome coronavirus 2 isolate SARS-CoV-2/human/USA/MI-MDHHS-SC20367/	TTCACCGAGGCCACGCGGAGTACGATCGAGTGTACAGTGAA
MT506208.1 Severe acute respiratory syndrome coronavirus 2 isolate SARS-CoV-2/human/USA/MI-MDHHS-SC20368/	TTCACCGAGGCCACGCGGAGTACGATCGAGTGTACAGTGAA
MT506209.1 Severe acute respiratory syndrome coronavirus 2 isolate SARS-CoV-2/human/USA/MI-MDHHS-SC20369/	TTCACCGAGGCCACGCGGAGTACGATCGAGTGTACAGTGAA

MT506210.1 Severe acute respiratory syndrome coronavirus 2 isolate SARS-CoV-2/human/USA/OH-MDHHS-SC20370	TTCACCGAGGCCACGCGGAGTACGATCGAGTGTACAGTGAA
MT506211.1 Severe acute respiratory syndrome coronavirus 2 isolate SARS-CoV-2/human/USA/MI-MDHHS-SC20371/	TTCACCGAGGCCACGCGGAGTACGATCGAGTGTACAGTGAA
MT506212.1 Severe acute respiratory syndrome coronavirus 2 isolate SARS-CoV-2/human/USA/MI-MDHHS-SC20372/	TTCACCGAGGCCACGCGGAGTACGATCGAGTGTACAGTGAA
MT506213.1 Severe acute respiratory syndrome coronavirus 2 isolate SARS-CoV-2/human/USA/MI-MDHHS-SC20373/	TTCACCGAGGCCACGCGGAGTACGATCGAGTGTACAGTGAA
MT506214.1 Severe acute respiratory syndrome coronavirus 2 isolate SARS-CoV-2/human/USA/MI-MDHHS-SC20374/	TTCACCGAGGCCACGCGGAGTACGATCGAGTGTACAGTGAA
MT506216.1 Severe acute respiratory syndrome coronavirus 2 isolate SARS-CoV-2/human/USA/MI-MDHHS-SC20376/	TTCACCGAGGCCACGCGGAGTACGATCGAGTGTACAGTGAA
MT506217.1 Severe acute respiratory syndrome coronavirus 2 isolate SARS-CoV-2/human/USA/MI-MDHHS-SC20377/	TTCACCGAGGCCACGCGGAGTACGATCGAGTGTACAGTGAA
MT506219.1 Severe acute respiratory syndrome coronavirus 2 isolate SARS-CoV-2/human/USA/MI-MDHHS-SC20379/	TTCACCGAGGCCACGCGGAGTACGATCGAGTGTACAGTGAA
MT506220.1 Severe acute respiratory syndrome coronavirus 2 isolate SARS-CoV-2/human/USA/MI-MDHHS-SC20380/	TTCACCGAGGCCACGCGGAGTACGATCGAGTGTACAGTGAA
MT506221.1 Severe acute respiratory syndrome coronavirus 2 isolate SARS-CoV-2/human/USA/MI-MDHHS-SC20381/	TTCACCGAGGCCACGCGGAGTACGATCGAGTGTACAGTGAA
MT506223.1 Severe acute respiratory syndrome coronavirus 2 isolate SARS-CoV-2/human/USA/MI-MDHHS-SC20383/	TTCACCGAGGCCACGCGGAGTACGATCGAGTGTACAGTGAA
MT506224.1 Severe acute respiratory syndrome coronavirus 2 isolate SARS-CoV-2/human/USA/MI-MDHHS-SC20384/	TTCACCGAGGCCACGCGGAGTACGATCGAGTGTACAGTGAA
MT506225.1 Severe acute respiratory syndrome coronavirus 2 isolate SARS-CoV-2/human/USA/MI-MDHHS-SC20385/	TTCACCGAGGCCACGCGGAGTACGATCGAGTGTACAGTGAA
MT506226.1 Severe acute respiratory syndrome coronavirus 2 isolate SARS-CoV-2/human/USA/MI-MDHHS-SC20386/	TTCACCGAGGCCACGCGGAGTACGATCGAGTGTACAGTGAA
MT506227.1 Severe acute respiratory syndrome coronavirus 2 isolate SARS-CoV-2/human/USA/MI-MDHHS-SC20387/	TTCACCGAGGCCACGCGGAGTACGATCGAGTGTACAGTGAA
MT506228.1 Severe acute respiratory syndrome coronavirus 2 isolate SARS-CoV-2/human/USA/MI-MDHHS-SC20388/	TTCACCGAGGCCACGCGGAGTACGATCGAGTGTACAGTGAA
MT506229.1 Severe acute respiratory syndrome coronavirus 2 isolate SARS-CoV-2/human/USA/MI-MDHHS-SC20389/	TTCACCGAGGCCACGCGGAGTACGATCGAGTGTACAGTGAA
MT506526.1 Severe acute respiratory syndrome coronavirus 2 isolate SARS-CoV-2/human/USA/MI-MDHHS-SC20309/	TTCACCGAGGCCACGCGGAGTACGATCGAGTGTACAGTGAA
MT506527.1 Severe acute respiratory syndrome coronavirus 2 isolate SARS-CoV-2/human/USA/MI-MDHHS-SC20300/	TTCACCGAGGCCACGCGGAGTACGATCGAGTGTACAGTGAA
MT506528.1 Severe acute respiratory syndrome coronavirus 2 isolate SARS-CoV-2/human/USA/MI-MDHHS-SC20314/	TTCACCGAGGCCACGCGGAGTACGATCGAGTGTACAGTGAA
MT506529.1 Severe acute respiratory syndrome coronavirus 2 isolate SARS-CoV-2/human/USA/MI-MDHHS-SC20319/	TTCACCGAGGCCACGCGGAGTACGATCGAGTGTACAGTGAA
MT506530.1 Severe acute respiratory syndrome coronavirus 2 isolate SARS-CoV-2/human/USA/MI-MDHHS-SC20260/	TTCACCGAGGCCACGCGGAGTACGATCGAGTGTACAGTGAA
MT506531.1 Severe acute respiratory syndrome coronavirus 2 isolate SARS-CoV-2/human/USA/MI-MDHHS-SC20299/	TTCACCGAGGCCACGCGGAGTACGATCGAGTGTACAGTGAA
MT506532.1 Severe acute respiratory syndrome coronavirus 2 isolate SARS-CoV-2/human/USA/MI-MDHHS-SC20292/	TTCACCGAGGCCACGCGGAGTACGATCGAGTGTACAGTGAA
MT506533.1 Severe acute respiratory syndrome coronavirus 2 isolate SARS-CoV-2/human/USA/MI-MDHHS-SC20310/	TTCACCGAGGCCACGCGGAGTACGATCGAGTGTACAGTGAA
MT506534.1 Severe acute respiratory syndrome coronavirus 2 isolate SARS-CoV-2/human/USA/MI-MDHHS-SC20296/	TTCACCGAGGCCACGCGGAGTACGATCGAGTGTACAGTGAA
MT506535.1 Severe acute respiratory syndrome coronavirus 2 isolate SARS-CoV-2/human/USA/MI-MDHHS-SC20304/	TTCACCGAGGCCACGCGGAGTACGATCGAGTGTACAGTGAA
MT506536.1 Severe acute respiratory syndrome coronavirus 2 isolate SARS-CoV-2/human/USA/MI-MDHHS-SC20317/	TTCACCGAGGCCACGCGGAGTACGATCGAGTGTACAGTGAA
MT506537.1 Severe acute respiratory syndrome coronavirus 2 isolate SARS-CoV-2/human/USA/MI-MDHHS-SC20295/	TTCACCGAGGCCACGCGGAGTACGATCGAGTGTACAGTGAA
MT506538.1 Severe acute respiratory syndrome coronavirus 2 isolate SARS-CoV-2/human/USA/MI-MDHHS-SC20261/	TTCACCGAGGCCACGCGGAGTACGATCGAGTGTACAGTGAA
MT506539.1 Severe acute respiratory syndrome coronavirus 2 isolate SARS-CoV-2/human/USA/MI-MDHHS-SC20294/	TTCACCGAGGCCACGCGGAGTACGATCGAGTGTACAGTGAA
MT506540.1 Severe acute respiratory syndrome coronavirus 2 isolate SARS-CoV-2/human/USA/MI-MDHHS-SC20297/	TTCACCGAGGCCACGCGGAGTACGATCGAGTGTACAGTGAA
MT506541.1 Severe acute respiratory syndrome coronavirus 2 isolate SARS-CoV-2/human/USA/MI-MDHHS-SC20274/	TTCACCGAGGCCACGCGGAGTACGATCGAGTGTACAGTGAA

MT506542.1 Severe acute respiratory syndrome coronavirus 2 isolate SARS-CoV-2/human/USA/MI-MDHHS-SC20306/	TTCACCGAGGCCACGCGGAGTACGATCGAGTGTACAGTGAA
MT506543.1 Severe acute respiratory syndrome coronavirus 2 isolate SARS-CoV-2/human/USA/MI-MDHHS-SC20192/	TTCACCGAGGCCACGCGGAGTACGATCGAGTGTACAGTGAA
MT506544.1 Severe acute respiratory syndrome coronavirus 2 isolate SARS-CoV-2/human/USA/MI-MDHHS-SC20320/	TTCACCGAGGCCACGCGGAGTACGATCGAGTGTACAGTGAA
MT506545.1 Severe acute respiratory syndrome coronavirus 2 isolate SARS-CoV-2/human/USA/MI-MDHHS-SC20302/	TTCACCGAGGCCACGCGGAGTACGATCGAGTGTACAGTGAA
MT506546.1 Severe acute respiratory syndrome coronavirus 2 isolate SARS-CoV-2/human/USA/MI-MDHHS-SC20321/	TTCACCGAGGCCACGCGGAGTACGATCGAGTGTACAGTGAA
MT506547.1 Severe acute respiratory syndrome coronavirus 2 isolate SARS-CoV-2/human/USA/MI-MDHHS-SC20263/	TTCACCGAGGCCACGCGGAGTACGATCGAGTGTACAGTGAA
MT506548.1 Severe acute respiratory syndrome coronavirus 2 isolate SARS-CoV-2/human/USA/MI-MDHHS-SC20280/	TTCACCGAGGCCACGCGGAGTACGATCGAGTGTACAGTGAA
MT506549.1 Severe acute respiratory syndrome coronavirus 2 isolate SARS-CoV-2/human/USA/MI-MDHHS-SC20271/	TTCACCGAGGCCACGCGGAGTACGATCGAGTGTACAGTGAA
MT506550.1 Severe acute respiratory syndrome coronavirus 2 isolate SARS-CoV-2/human/USA/MI-MDHHS-SC20298/	TTCACCGAGGCCACGCGGAGTACGATCGAGTGTACAGTGAA
MT506551.1 Severe acute respiratory syndrome coronavirus 2 isolate SARS-CoV-2/human/USA/MI-MDHHS-SC20287/	TTCACCGAGGCCACGCGGAGTACGATCGAGTGTACAGTGAA
MT506552.1 Severe acute respiratory syndrome coronavirus 2 isolate SARS-CoV-2/human/USA/MI-MDHHS-SC20269/	TTCACCGAGGCCACGCGGAGTACGATCGAGTGTACAGTGAA
MT506553.1 Severe acute respiratory syndrome coronavirus 2 isolate SARS-CoV-2/human/USA/MI-MDHHS-SC20281/	TTCACCGAGGCCACGCGGAGTACGATCGAGTGTACAGTGAA
MT506554.1 Severe acute respiratory syndrome coronavirus 2 isolate SARS-CoV-2/human/USA/MI-MDHHS-SC20262/	TTCACCGAGGCCACGCGGAGTACGATCGAGTGTACAGTGAA
MT506555.1 Severe acute respiratory syndrome coronavirus 2 isolate SARS-CoV-2/human/USA/MI-MDHHS-SC20276/	TTCACCGAGGCCACGCGGAGTACGATCGAGTGTACAGTGAA
MT506556.1 Severe acute respiratory syndrome coronavirus 2 isolate SARS-CoV-2/human/USA/MI-MDHHS-SC20273/	TTCACCGAGGCCACGCGGAGTACGATCGAGTGTACAGTGAA
MT506558.1 Severe acute respiratory syndrome coronavirus 2 isolate SARS-CoV-2/human/USA/MI-MDHHS-SC20268/	TTCACCGAGGCCACGCGGAGTACGATCGAGTGTACAGTGAA
MT506559.1 Severe acute respiratory syndrome coronavirus 2 isolate SARS-CoV-2/human/USA/MI-MDHHS-SC20283/	TTCACCGAGGCCACGCGGAGTACGATCGAGTGTACAGTGAA
MT506560.1 Severe acute respiratory syndrome coronavirus 2 isolate SARS-CoV-2/human/USA/MI-MDHHS-SC20264/	TTCACCGAGGCCACGCGGAGTACGATCGAGTGTACAGTGAA
MT506561.1 Severe acute respiratory syndrome coronavirus 2 isolate SARS-CoV-2/human/USA/MI-MDHHS-SC20285/	TTCACCGAGGCCACGCGGAGTACGATCGAGTGTACAGTGAA
MT506562.1 Severe acute respiratory syndrome coronavirus 2 isolate SARS-CoV-2/human/USA/MI-MDHHS-SC20288/	TTCACCGAGGCCACGCGGAGTACGATCGAGTGTACAGTGAA
MT506563.1 Severe acute respiratory syndrome coronavirus 2 isolate SARS-CoV-2/human/USA/MI-MDHHS-SC20286/	TTCACCGAGGCCACGCGGAGTACGATCGAGTGTACAGTGAA
MT506564.1 Severe acute respiratory syndrome coronavirus 2 isolate SARS-CoV-2/human/USA/MI-MDHHS-SC20290/	TTCACCGAGGCCACGCGGAGTACGATCGAGTGTACAGTGAA
MT506565.1 Severe acute respiratory syndrome coronavirus 2 isolate SARS-CoV-2/human/USA/MI-MDHHS-SC20293/	TTCACCGAGGCCACGCGGAGTACGATCGAGTGTACAGTGAA
MT506566.1 Severe acute respiratory syndrome coronavirus 2 isolate SARS-CoV-2/human/USA/MI-MDHHS-SC20308/	TTCACCGAGGCCACGCGGAGTACGATCGAGTGTACAGTGAA
MT506567.1 Severe acute respiratory syndrome coronavirus 2 isolate SARS-CoV-2/human/USA/MI-MDHHS-SC20316/	TTCACCGAGGCCACGCGGAGTACGATCGAGTGTACAGTGAA
MT506568.1 Severe acute respiratory syndrome coronavirus 2 isolate SARS-CoV-2/human/USA/MI-MDHHS-SC20270/	TTCACCGAGGCCACGCGGAGTACGATCGAGTGTACAGTGAA
MT506569.1 Severe acute respiratory syndrome coronavirus 2 isolate SARS-CoV-2/human/USA/MI-MDHHS-SC20305/	TTCACCGAGGCCACGCGGAGTACGATCGAGTGTACAGTGAA
MT506570.1 Severe acute respiratory syndrome coronavirus 2 isolate SARS-CoV-2/human/USA/MI-MDHHS-SC20266/	TTCACCGAGGCCACGCGGAGTACGATCGAGTGTACAGTGAA
MT506571.1 Severe acute respiratory syndrome coronavirus 2 isolate SARS-CoV-2/human/USA/MI-MDHHS-SC20301/	TTCACCGAGGCCACGCGGAGTACGATCGAGTGTACAGTGAA
MT506572.1 Severe acute respiratory syndrome coronavirus 2 isolate SARS-CoV-2/human/USA/MI-MDHHS-SC20307/	TTCACCGAGGCCACGCGGAGTACGATCGAGTGTACAGTGAA
MT506573.1 Severe acute respiratory syndrome coronavirus 2 isolate SARS-CoV-2/human/USA/MI-MDHHS-SC20291/	TTCACCGAGGCCACGCGGAGTACGATCGAGTGTACAGTGAA
MT506574.1 Severe acute respiratory syndrome coronavirus 2 isolate SARS-CoV-2/human/USA/MI-MDHHS-SC20312/	TTCACCGAGGCCACGCGGAGTACGATCGAGTGTACAGTGAA
MT506576.1 Severe acute respiratory syndrome coronavirus 2 isolate SARS-CoV-2/human/USA/MI-MDHHS-SC20313/	TTCACCGAGGCCACGCGGAGTACGATCGAGTGTACAGTGAA

MT506577.1 Severe acute respiratory syndrome coronavirus 2 isolate SARS-CoV-2/human/USA/MI-MDHHS-SC20303/	TTCACCGAGGCCACGCGGAGTACGATCGAGTGTACAGTGAA
MT506578.1 Severe acute respiratory syndrome coronavirus 2 isolate SARS-CoV-2/human/USA/MI-MDHHS-SC20311/	TTCACCGAGGCCACGCGGAGTACGATCGAGTGTACAGTGAA
MT506579.1 Severe acute respiratory syndrome coronavirus 2 isolate SARS-CoV-2/human/USA/MI-MDHHS-SC20217/	TTCACCGAGGCCACGCGGAGTACGATCGAGTGTACAGTGAA
MT506580.1 Severe acute respiratory syndrome coronavirus 2 isolate SARS-CoV-2/human/USA/MI-MDHHS-SC20284/	TTCACCGAGGCCACGCGGAGTACGATCGAGTGTACAGTGAA
MT506581.1 Severe acute respiratory syndrome coronavirus 2 isolate SARS-CoV-2/human/USA/MI-MDHHS-SC20318/	TTCACCGAGGCCACGCGGAGTACGATCGAGTGTACAGTGAA
MT506582.1 Severe acute respiratory syndrome coronavirus 2 isolate SARS-CoV-2/human/USA/MI-MDHHS-SC20279/	TTCACCGAGGCCACGCGGAGTACGATCGAGTGTACAGTGAA
MT506583.1 Severe acute respiratory syndrome coronavirus 2 isolate SARS-CoV-2/human/USA/MI-MDHHS-SC20277/	TTCACCGAGGCCACGCGGAGTACGATCGAGTGTACAGTGAA
MT506584.1 Severe acute respiratory syndrome coronavirus 2 isolate SARS-CoV-2/human/USA/MI-MDHHS-SC20282/	TTCACCGAGGCCACGCGGAGTACGATCGAGTGTACAGTGAA
MT506585.1 Severe acute respiratory syndrome coronavirus 2 isolate SARS-CoV-2/human/USA/MI-MDHHS-SC20275/	TTCACCGAGGCCACGCGGAGTACGATCGAGTGTACAGTGAA
MT506586.1 Severe acute respiratory syndrome coronavirus 2 isolate SARS-CoV-2/human/USA/MI-MDHHS-SC20272/	TTCACCGAGGCCACGCGGAGTACGATCGAGTGTACAGTGAA
MT506587.1 Severe acute respiratory syndrome coronavirus 2 isolate SARS-CoV-2/human/USA/MI-MDHHS-SC20267/	TTCACCGAGGCCACGCGGAGTACGATCGAGTGTACAGTGAA
MT506637.1 Severe acute respiratory syndrome coronavirus 2 isolate SARS-CoV-2/human/USA/MI-MDHHS-SC20257/	TTCACCGAGGCCACGCGGAGTACGATCGAGTGTACAGTGAA
MT506638.1 Severe acute respiratory syndrome coronavirus 2 isolate SARS-CoV-2/human/USA/MI-MDHHS-SC20209/	TTCACCGAGGCCACGCGGAGTACGATCGAGTGTACAGTGAA
MT506639.1 Severe acute respiratory syndrome coronavirus 2 isolate SARS-CoV-2/human/USA/MI-MDHHS-SC20221/	TTCACCGAGGCCACGCGGAGTACGATCGAGTGTACAGTGAA
MT506640.1 Severe acute respiratory syndrome coronavirus 2 isolate SARS-CoV-2/human/USA/MI-MDHHS-SC20248/	TTCACCGAGGCCACGCGGAGTACGATCGAGTGTACAGTGAA
MT506641.1 Severe acute respiratory syndrome coronavirus 2 isolate SARS-CoV-2/human/USA/MI-MDHHS-SC20198/	TTCACCGAGGCCACGCGGAGTACGATCGAGTGTACAGTGAA
MT506642.1 Severe acute respiratory syndrome coronavirus 2 isolate SARS-CoV-2/human/USA/MI-MDHHS-SC20224/	TTCACCGAGGCCACGCGGAGTACGATCGAGTGTACAGTGAA
MT506643.1 Severe acute respiratory syndrome coronavirus 2 isolate SARS-CoV-2/human/USA/MI-MDHHS-SC20206/	TTCACCGAGGCCACGCGGAGTACGATCGAGTGTACAGTGAA
MT506644.1 Severe acute respiratory syndrome coronavirus 2 isolate SARS-CoV-2/human/USA/MI-MDHHS-SC20212/	TTCACCGAGGCCACGCGGAGTACGATCGAGTGTACAGTGAA
MT506645.1 Severe acute respiratory syndrome coronavirus 2 isolate SARS-CoV-2/human/USA/MI-MDHHS-SC20215/	TTCACCGAGGCCACGCGGAGTACGATCGAGTGTACAGTGAA
MT506646.1 Severe acute respiratory syndrome coronavirus 2 isolate SARS-CoV-2/human/USA/MI-MDHHS-SC20218/	TTCACCGAGGCCACGCGGAGTACGATCGAGTGTACAGTGAA
MT506647.1 Severe acute respiratory syndrome coronavirus 2 isolate SARS-CoV-2/human/USA/MI-MDHHS-SC20244/	TTCACCGAGGCCACGCGGAGTACGATCGAGTGTACAGTGAA
MT506648.1 Severe acute respiratory syndrome coronavirus 2 isolate SARS-CoV-2/human/USA/MI-MDHHS-SC20236/	TTCACCGAGGCCACGCGGAGTACGATCGAGTGTACAGTGAA
MT506649.1 Severe acute respiratory syndrome coronavirus 2 isolate SARS-CoV-2/human/USA/MI-MDHHS-SC20230/	TTCACCGAGGCCACGCGGAGTACGATCGAGTGTACAGTGAA
MT506650.1 Severe acute respiratory syndrome coronavirus 2 isolate SARS-CoV-2/human/USA/MI-MDHHS-SC20258/	TTCACCGAGGCCACGCGGAGTACGATCGAGTGTACAGTGAA
MT506651.1 Severe acute respiratory syndrome coronavirus 2 isolate SARS-CoV-2/human/USA/MI-MDHHS-SC20204/	TTCACCGAGGCCACGCGGAGTACGATCGAGTGTACAGTGAA
MT506652.1 Severe acute respiratory syndrome coronavirus 2 isolate SARS-CoV-2/human/USA/MI-MDHHS-SC20226/	TTCACCGAGGCCACGCGGAGTACGATCGAGTGTACAGTGAA
MT506654.1 Severe acute respiratory syndrome coronavirus 2 isolate SARS-CoV-2/human/USA/MI-MDHHS-SC20251/	TTCACCGAGGCCACGCGGAGTACGATCGAGTGTACAGTGAA
MT506655.1 Severe acute respiratory syndrome coronavirus 2 isolate SARS-CoV-2/human/USA/MI-MDHHS-SC20228/	TTCACCGAGGCCACGCGGAGTACGATCGAGTGTACAGTGAA
MT506656.1 Severe acute respiratory syndrome coronavirus 2 isolate SARS-CoV-2/human/USA/MI-MDHHS-SC20199/	TTCACCGAGGCCACGCGGAGTACGATCGAGTGTACAGTGAA
MT506657.1 Severe acute respiratory syndrome coronavirus 2 isolate SARS-CoV-2/human/USA/MI-MDHHS-SC20222/	TTCACCGAGGCCACGCGGAGTACGATCGAGTGTACAGTGAA
MT506658.1 Severe acute respiratory syndrome coronavirus 2 isolate SARS-CoV-2/human/USA/MI-MDHHS-SC20245/	TTCACCGAGGCCACGCGGAGTACGATCGAGTGTACAGTGAA
MT506659.1 Severe acute respiratory syndrome coronavirus 2 isolate SARS-CoV-2/human/USA/MI-MDHHS-SC20208/	TTCACCGAGGCCACGCGGAGTACGATCGAGTGTACAGTGAA

MT506660.1 Severe acute respiratory syndrome coronavirus 2 isolate SARS-CoV-2/human/USA/MI-MDHHS-SC20207/	TTCACCGAGGCCACGCGGAGTACGATCGAGTGTACAGTGAA
MT506661.1 Severe acute respiratory syndrome coronavirus 2 isolate SARS-CoV-2/human/USA/MI-MDHHS-SC20238/	TTCACCGAGGCCACGCGGAGTACGATCGAGTGTACAGTGAA
MT506662.1 Severe acute respiratory syndrome coronavirus 2 isolate SARS-CoV-2/human/USA/MI-MDHHS-SC20229/	TTCACCGAGGCCACGCGGAGTACGATCGAGTGTACAGTGAA
MT506663.1 Severe acute respiratory syndrome coronavirus 2 isolate SARS-CoV-2/human/USA/MI-MDHHS-SC20196/	TTCACCGAGGCCACGCGGAGTACGATCGAGTGTACAGTGAA
MT506665.1 Severe acute respiratory syndrome coronavirus 2 isolate SARS-CoV-2/human/USA/MI-MDHHS-SC20231/	TTCACCGAGGCCACGCGGAGTACGATCGAGTGTACAGTGAA
MT506666.1 Severe acute respiratory syndrome coronavirus 2 isolate SARS-CoV-2/human/USA/MI-MDHHS-SC20223/	TTCACCGAGGCCACGCGGAGTACGATCGAGTGTACAGTGAA
MT506667.1 Severe acute respiratory syndrome coronavirus 2 isolate SARS-CoV-2/human/USA/MI-MDHHS-SC20255/	TTCACCGAGGCCACGCGGAGTACGATCGAGTGTACAGTGAA
MT506668.1 Severe acute respiratory syndrome coronavirus 2 isolate SARS-CoV-2/human/USA/MI-MDHHS-SC20203/	TTCACCGAGGCCACGCGGAGTACGATCGAGTGTACAGTGAA
MT506669.1 Severe acute respiratory syndrome coronavirus 2 isolate SARS-CoV-2/human/USA/MI-MDHHS-SC20193/	TTCACCGAGGCCACGCGGAGTACGATCGAGTGTACAGTGAA
MT506670.1 Severe acute respiratory syndrome coronavirus 2 isolate SARS-CoV-2/human/USA/MI-MDHHS-SC20246/	TTCACCGAGGCCACGCGGAGTACGATCGAGTGTACAGTGAA
MT506671.1 Severe acute respiratory syndrome coronavirus 2 isolate SARS-CoV-2/human/USA/MI-MDHHS-SC20211/	TTCACCGAGGCCACGCGGAGTACGATCGAGTGTACAGTGAA
MT506672.1 Severe acute respiratory syndrome coronavirus 2 isolate SARS-CoV-2/human/USA/MI-MDHHS-SC20200/	TTCACCGAGGCCACGCGGAGTACGATCGAGTGTACAGTGAA
MT506673.1 Severe acute respiratory syndrome coronavirus 2 isolate SARS-CoV-2/human/USA/MI-MDHHS-SC20210/	TTCACCGAGGCCACGCGGAGTACGATCGAGTGTACAGTGAA
MT506674.1 Severe acute respiratory syndrome coronavirus 2 isolate SARS-CoV-2/human/USA/MI-MDHHS-SC20237/	TTCACCGAGGCCACGCGGAGTACGATCGAGTGTACAGTGAA
MT506675.1 Severe acute respiratory syndrome coronavirus 2 isolate SARS-CoV-2/human/USA/MI-MDHHS-SC20191/	TTCACCGAGGCCACGCGGAGTACGATCGAGTGTACAGTGAA
MT506676.1 Severe acute respiratory syndrome coronavirus 2 isolate SARS-CoV-2/human/USA/MI-MDHHS-SC20225/	TTCACCGAGGCCACGCGGAGTACGATCGAGTGTACAGTGAA
MT506677.1 Severe acute respiratory syndrome coronavirus 2 isolate SARS-CoV-2/human/USA/MI-MDHHS-SC20250/	TTCACCGAGGCCACGCGGAGTACGATCGAGTGTACAGTGAA
MT506678.1 Severe acute respiratory syndrome coronavirus 2 isolate SARS-CoV-2/human/USA/MI-MDHHS-SC20247/	TTCACCGAGGCCACGCGGAGTACGATCGAGTGTACAGTGAA
MT506679.1 Severe acute respiratory syndrome coronavirus 2 isolate SARS-CoV-2/human/USA/MI-MDHHS-SC20194/	TTCACCGAGGCCACGCGGAGTACGATCGAGTGTACAGTGAA
MT506680.1 Severe acute respiratory syndrome coronavirus 2 isolate SARS-CoV-2/human/USA/MI-MDHHS-SC20202/	TTCACCGAGGCCACGCGGAGTACGATCGAGTGTACAGTGAA
MT506681.1 Severe acute respiratory syndrome coronavirus 2 isolate SARS-CoV-2/human/USA/MI-MDHHS-SC20242/	TTCACCGAGGCCACGCGGAGTACGATCGAGTGTACAGTGAA
MT506682.1 Severe acute respiratory syndrome coronavirus 2 isolate SARS-CoV-2/human/USA/MI-MDHHS-SC20252/	TTCACCGAGGCCACGCGGAGTACGATCGAGTGTACAGTGAA
MT506683.1 Severe acute respiratory syndrome coronavirus 2 isolate SARS-CoV-2/human/USA/MI-MDHHS-SC20239/	TTCACCGAGGCCACGCGGAGTACGATCGAGTGTACAGTGAA
MT506684.1 Severe acute respiratory syndrome coronavirus 2 isolate SARS-CoV-2/human/USA/MI-MDHHS-SC20243/	TTCACCGAGGCCACGCGGAGTACGATCGAGTGTACAGTGAA
MT506685.1 Severe acute respiratory syndrome coronavirus 2 isolate SARS-CoV-2/human/USA/MI-MDHHS-SC20197/	TTCACCGAGGCCACGCGGAGTACGATCGAGTGTACAGTGAA
MT506686.1 Severe acute respiratory syndrome coronavirus 2 isolate SARS-CoV-2/human/USA/MI-MDHHS-SC20253/	TTCACCGAGGCCACGCGGAGTACGATCGAGTGTACAGTGAA
MT506687.1 Severe acute respiratory syndrome coronavirus 2 isolate SARS-CoV-2/human/USA/MI-MDHHS-SC20241/	TTCACCGAGGCCACGCGGAGTACGATCGAGTGTACAGTGAA
MT506689.1 Severe acute respiratory syndrome coronavirus 2 isolate SARS-CoV-2/human/USA/MI-MDHHS-SC20213/	TTCACCGAGGCCACGCGGAGTACGATCGAGTGTACAGTGAA
MT506690.1 Severe acute respiratory syndrome coronavirus 2 isolate SARS-CoV-2/human/USA/MI-MDHHS-SC20219/	TTCACCGAGGCCACGCGGAGTACGATCGAGTGTACAGTGAA
MT506691.1 Severe acute respiratory syndrome coronavirus 2 isolate SARS-CoV-2/human/USA/MI-MDHHS-SC20234/	TTCACCGAGGCCACGCGGAGTACGATCGAGTGTACAGTGAA
MT506692.1 Severe acute respiratory syndrome coronavirus 2 isolate SARS-CoV-2/human/USA/MI-MDHHS-SC20233/	TTCACCGAGGCCACGCGGAGTACGATCGAGTGTACAGTGAA
MT506693.1 Severe acute respiratory syndrome coronavirus 2 isolate SARS-CoV-2/human/USA/MI-MDHHS-SC20259/	TTCACCGAGGCCACGCGGAGTACGATCGAGTGTACAGTGAA
MT506694.1 Severe acute respiratory syndrome coronavirus 2 isolate SARS-CoV-2/human/USA/MI-MDHHS-SC20201/	TTCACCGAGGCCACGCGGAGTACGATCGAGTGTACAGTGAA

MT506695.1 Severe acute respiratory syndrome coronavirus 2 isolate SARS-CoV-2/human/USA/MI-MDHHS-SC20254/	TTCACCGAGGCCACGCGGAGTACGATCGAGTGTACAGTGAA
MT506696.1 Severe acute respiratory syndrome coronavirus 2 isolate SARS-CoV-2/human/USA/MI-MDHHS-SC20220/	TTCACCGAGGCCACGCGGAGTACGATCGAGTGTACAGTGAA
MT506697.1 Severe acute respiratory syndrome coronavirus 2 isolate SARS-CoV-2/human/USA/MI-MDHHS-SC20249/	TTCACCGAGGCCACGCGGAGTACGATCGAGTGTACAGTGAA
MT506698.1 Severe acute respiratory syndrome coronavirus 2 isolate SARS-CoV-2/human/USA/MI-MDHHS-SC20214/	TTCACCGAGGCCACGCGGAGTACGATCGAGTGTACAGTGAA
MT506699.1 Severe acute respiratory syndrome coronavirus 2 isolate SARS-CoV-2/human/USA/MI-MDHHS-SC20256/	TTCACCGAGGCCACGCGGAGTACGATCGAGTGTACAGTGAA
MT506701.1 Severe acute respiratory syndrome coronavirus 2 isolate SARS-CoV-2/human/USA/MI-MDHHS-SC20240/	TTCACCGAGGCCACGCGGAGTACGATCGAGTGTACAGTGAA
MT506702.1 Severe acute respiratory syndrome coronavirus 2 isolate SARS-CoV-2/human/USA/MI-MDHHS-SC20205/	TTCACCGAGGCCACGCGGAGTACGATCGAGTGTACAGTGAA
MT506707.1 Severe acute respiratory syndrome coronavirus 2 isolate SARS-CoV-2/human/USA/COVID19-AK087/2020	TTCACCGAGGCCACGCGGAGTACGATCGAGTGTACAGTGAA
MT506885.1 Severe acute respiratory syndrome coronavirus 2 isolate SARS-CoV-2/human/USA/WI-UW-20/2020, com	TTCACCGAGGCCACGCGGAGTACGATCGAGTGTACAGTGAA
MT506886.1 Severe acute respiratory syndrome coronavirus 2 isolate SARS-CoV-2/human/USA/WI-UW-347/2020, co	TTCACCGAGGCCACGCGGAGTACGATCGAGTGTACAGTGAA
MT506887.1 Severe acute respiratory syndrome coronavirus 2 isolate SARS-CoV-2/human/USA/WI-UW-348/2020, co	TTCACCGAGGCCACGCGGAGTACGATCGAGTGTACAGTGAA
MT506888.1 Severe acute respiratory syndrome coronavirus 2 isolate SARS-CoV-2/human/USA/WI-UW-349/2020, co	TTCACCGAGGCCACGCGGAGTACGATCGAGTGTACAGTGAA
MT506889.1 Severe acute respiratory syndrome coronavirus 2 isolate SARS-CoV-2/human/USA/WI-UW-350/2020, co	TTCACCGAGGCCACGCGGAGTACGATCGAGTGTACAGTGAA
MT506890.1 Severe acute respiratory syndrome coronavirus 2 isolate SARS-CoV-2/human/USA/WI-UW-351/2020, co	TTCACCGAGGCCACGCGGAGTACGATCGAGTGTACAGTGAA
MT506891.1 Severe acute respiratory syndrome coronavirus 2 isolate SARS-CoV-2/human/USA/WI-UW-352/2020, co	TTCACCGAGGCCACGCGGAGTACGATCGAGTGTACAGTGAA
MT506892.1 Severe acute respiratory syndrome coronavirus 2 isolate SARS-CoV-2/human/USA/WI-UW-353/2020, co	TTCACCGAGGCCACGCGGAGTACGATCGAGTGTACAGTGAA
MT506893.1 Severe acute respiratory syndrome coronavirus 2 isolate SARS-CoV-2/human/USA/WI-UW-354/2020, co	TTCACCGAGGCCACGCGGAGTACGATCGAGTGTACAGTGAA
MT506894.1 Severe acute respiratory syndrome coronavirus 2 isolate SARS-CoV-2/human/USA/WI-UW-355/2020, co	TTCACCGAGGCCACGCGGAGTACGATCGAGTGTACAGTGAA
MT506895.1 Severe acute respiratory syndrome coronavirus 2 isolate SARS-CoV-2/human/USA/WI-UW-356/2020, co	TTCACCGAGGCCACGCGGAGTACGATCGAGTGTACAGTGAA
MT506896.1 Severe acute respiratory syndrome coronavirus 2 isolate SARS-CoV-2/human/USA/WI-UW-357/2020, co	TTCACCGAGGCCACGCGGAGTACGATCGAGTGTACAGTGAA
MT506897.1 Severe acute respiratory syndrome coronavirus 2 isolate SARS-CoV-2/human/USA/WI-UW-358/2020, co	TTCACCGAGGCCACGCGGAGTACGATCGAGTGTACAGTGAA
MT506898.1 Severe acute respiratory syndrome coronavirus 2 isolate SARS-CoV-2/human/USA/WI-UW-359/2020, co	TTCACCGAGGCCACGCGGAGTACGATCGAGTGTACAGTGAA
MT506899.1 Severe acute respiratory syndrome coronavirus 2 isolate SARS-CoV-2/human/USA/WI-UW-360/2020, co	TTCACCGAGGCCACGCGGAGTACGATCGAGTGTACAGTGAA
MT506900.1 Severe acute respiratory syndrome coronavirus 2 isolate SARS-CoV-2/human/USA/WI-UW-361/2020, co	TTCACCGAGGCCACGCGGAGTACGATCGAGTGTACAGTGAA
MT506901.1 Severe acute respiratory syndrome coronavirus 2 isolate SARS-CoV-2/human/USA/WI-UW-362/2020, co	TTCACCGAGGCCACGCGGAGTACGATCGAGTGTACAGTGAA
MT506902.1 Severe acute respiratory syndrome coronavirus 2 isolate SARS-CoV-2/human/USA/WI-UW-363/2020, co	TTCACCGAGGCCACGCGGAGTACGATCGAGTGTACAGTGAA
MT506903.1 Severe acute respiratory syndrome coronavirus 2 isolate SARS-CoV-2/human/USA/WI-UW-364/2020, co	TTCACCGAGGCCACGCGGAGTACGATCGAGTGTACAGTGAA
MT506904.1 Severe acute respiratory syndrome coronavirus 2 isolate SARS-CoV-2/human/USA/WI-UW-365/2020, co	TTCACCGAGGCCACGCGGAGTACGATCGAGTGTACAGTGAA
MT506905.1 Severe acute respiratory syndrome coronavirus 2 isolate SARS-CoV-2/human/USA/WI-UW-366/2020, co	TTCACCGAGGCCACGCGGAGTACGATCGAGTGTACAGTGAA
MT506906.1 Severe acute respiratory syndrome coronavirus 2 isolate SARS-CoV-2/human/USA/WI-UW-367/2020, co	TTCACCGAGGCCACACGGAGTACGATCGAGTGTACAGTGAA
MT506907.1 Severe acute respiratory syndrome coronavirus 2 isolate SARS-CoV-2/human/USA/WI-UW-368/2020, co	TTCACCGAGGCCACGCGGAGTACGATCGAGTGTACAGTGAA
MT506974.1 Severe acute respiratory syndrome coronavirus 2 isolate SARS-CoV-2/human/USA/FL-BPHL-0001/2020 C	TTCACCGAGGCCACGCGGAGTACGATCGAGTGTACAGTGAA
MT506975.1 Severe acute respiratory syndrome coronavirus 2 isolate SARS-CoV-2/human/USA/FL-BPHL-0002/2020 C	TTCACCGAGGCCACGCGGAGTACGATCGAGTGTACAGTGAA

MT506977.1 Severe acute respiratory syndrome coronavirus 2 isolate SARS-CoV-2/human/USA/FL-BPHL-0004/2020 C	TTCACCGAGGCCACACGGAGTACGATCGAGTGTACAGTGAA
MT506981.1 Severe acute respiratory syndrome coronavirus 2 isolate SARS-CoV-2/human/USA/FL-BPHL-0008/2020, c	TTCACCGAGGCCACGCGGAGTACGATCGAGTGTACAGTGAA
MT506990.1 Severe acute respiratory syndrome coronavirus 2 isolate SARS-CoV-2/human/USA/FL-BPHL-0017/2020 C	TTCACCGAGGCCACGCGGAGTACGATCGAGTGTACAGTGAA
MT506991.1 Severe acute respiratory syndrome coronavirus 2 isolate SARS-CoV-2/human/USA/FL-BPHL-0018/2020 C	TTCACCGAGGCCACGCGGAGTACGATCGAGTGTACAGTGAA
MT506993.1 Severe acute respiratory syndrome coronavirus 2 isolate SARS-CoV-2/human/USA/FL-BPHL-0020/2020 C	TTCACCGAGGCCACGCGGAGTACGATCGAGTGTACAGTGAA
MT506997.1 Severe acute respiratory syndrome coronavirus 2 isolate SARS-CoV-2/human/USA/FL-BPHL-0024/2020 C	TTCACCGAGGCCACGCGGAGTACGATCGAGTGTACAGTGAA
MT507001.1 Severe acute respiratory syndrome coronavirus 2 isolate SARS-CoV-2/human/USA/FL-BPHL-0028/2020 C	TTCACCGAGGCCACGCGGAGTACGATCGAGTGTACAGTGAA
MT507002.1 Severe acute respiratory syndrome coronavirus 2 isolate SARS-CoV-2/human/USA/FL-BPHL-0029/2020 C	TTCACCGAGGCCACGCGGAGTACGATCGAGTGTACAGTGAA
MT507006.1 Severe acute respiratory syndrome coronavirus 2 isolate SARS-CoV-2/human/USA/FL-BPHL-0033/2020 C	TTCACCGAGGCCACGCGGAGTACGATCGAGTGTACAGTGAA
MT507018.1 Severe acute respiratory syndrome coronavirus 2 isolate SARS-CoV-2/human/USA/FL-BPHL-0045/2020, c	TTCACCGAGGCCACGCGGAGTACGATCGAGTGTACAGTGAA
MT507019.1 Severe acute respiratory syndrome coronavirus 2 isolate SARS-CoV-2/human/USA/FL-BPHL-0046/2020 C	TTCACCGAGGCCACGCGGAGTACGATCGAGTGTACAGTGAA
MT507020.1 Severe acute respiratory syndrome coronavirus 2 isolate SARS-CoV-2/human/USA/FL-BPHL-0047/2020 C	TTCACCGAGGCCACGCGGAGTACGATCGAGTGTACAGTGAA
MT507021.1 Severe acute respiratory syndrome coronavirus 2 isolate SARS-CoV-2/human/USA/FL-BPHL-0048/2020 C	TTCACCGAGGCCACGCGGAGTACGATCGAGTGTACAGTGAA
MT507024.1 Severe acute respiratory syndrome coronavirus 2 isolate SARS-CoV-2/human/USA/FL-BPHL-0051/2020 C	TTCACCGAGGCCACGCGGAGTACGATCGAGTGTACAGTGAA
MT507027.1 Severe acute respiratory syndrome coronavirus 2 isolate SARS-CoV-2/human/USA/FL-BPHL-0054/2020 C	TTCACCGAGGCCACGCGGAGTACGATCGAGTGTACAGTGAA
MT507031.1 Severe acute respiratory syndrome coronavirus 2 isolate SARS-CoV-2/human/USA/FL-BPHL-0058/2020 C	TTCACCGAGGCCACGCGGAGTACGATCGAGTGTACAGTGAA
MT507032.1 Severe acute respiratory syndrome coronavirus 2 isolate SARS-CoV-2/human/USA/FL-BPHL-0059/2020 C	TTCACCGAGGCCACGCGGAGTACGATCGAGTGTACAGTGAA
MT507271.1 Severe acute respiratory syndrome coronavirus 2 isolate SARS-CoV-2/human/JAM/JM-CDC-0869/2020, c	TTCACCGAGGCCACGCGGAGTACGATCGAGTGTACAGTGAA
MT507272.1 Severe acute respiratory syndrome coronavirus 2 isolate SARS-CoV-2/human/JAM/JM-CDC-4376/2020, c	TTCACCGAGGCCACGCGGAGTACGATCGAGTGTACAGTGAA
MT507273.1 Severe acute respiratory syndrome coronavirus 2 isolate SARS-CoV-2/human/JAM/JM-CDC-5836/2020, c	TTCACCGAGGCCACGCGGAGTACGATCGAGTGTACAGTGAA
MT507274.1 Severe acute respiratory syndrome coronavirus 2 isolate SARS-CoV-2/human/JAM/JM-CDC-6365/2020, c	TTCACCGAGGCCACGCGGAGTACGATCGAGTGTACAGTGAA
MT507275.1 Severe acute respiratory syndrome coronavirus 2 isolate SARS-CoV-2/human/JAM/JM-CDC-6603/2020, c	TTCACCGAGGCCACGCGGAGTACGATCGAGTGTACAGTGAA
MT507276.1 Severe acute respiratory syndrome coronavirus 2 isolate SARS-CoV-2/human/JAM/JM-CDC-7286/2020, c	TTCACCGAGGCCACGCGGAGTACGATCGAGTGTACAGTGAA
MT507277.1 Severe acute respiratory syndrome coronavirus 2 isolate SARS-CoV-2/human/USA/AR-CDC-2072/2020, c	TTCACCGAGGCCACGCGGAGTACGATCGAGTGTACAGTGAA
MT507278.1 Severe acute respiratory syndrome coronavirus 2 isolate SARS-CoV-2/human/USA/VI-CDC-3943/2020, co	TTCACCGAGGCCACGCGGAGTACGATCGAGTGTACAGTGAA
MT507279.1 Severe acute respiratory syndrome coronavirus 2 isolate SARS-CoV-2/human/USA/GA-CDC-4518/2020, c	TTCACCGAGGCCACGCGGAGTACGATCGAGTGTACAGTGAA
MT507280.1 Severe acute respiratory syndrome coronavirus 2 isolate SARS-CoV-2/human/USA/VI-CDC-3810/2020, co	TTCACCGAGGCCACGCGGAGTACGATCGAGTGTACAGTGAA
MT507281.1 Severe acute respiratory syndrome coronavirus 2 isolate SARS-CoV-2/human/USA/PA-CDC-9081/2020, c	TTCACCGAGGCCACGCGGAGTACGATCGAGTGTACAGTGAA
MT507282.1 Severe acute respiratory syndrome coronavirus 2 isolate SARS-CoV-2/human/USA/PA-CDC-8398/2020, c	TTCACCGAGGCCACGCGGAGTACGATCGAGTGTACAGTGAA
MT507793.1 Severe acute respiratory syndrome coronavirus 2 isolate SARS-CoV-2/homo sapien/JAM/JM-CDC-0078/2	TTCACCGAGGCCACGCGGAGTACGATCGAGTGTACAGTGAA
MT507795.1 Severe acute respiratory syndrome coronavirus 2 isolate SARS-CoV-2/homo sapien/USA/VI-CDC-3884/20	TTCACCGAGGCCACGCGGAGTACGATCGAGTGTACAGTGAA
MT507796.1 Severe acute respiratory syndrome coronavirus 2 isolate SARS-CoV-2/homo sapien/USA/VI-CDC-3849/20	TTCACCGAGGCCACGCGGAGTACGATCGAGTGTACAGTGAA
MT509452.1 Severe acute respiratory syndrome coronavirus 2 isolate SARS-CoV-2/human/USA/MD-HP00016/2020, c	TTCACCGAGGCCACGCGGAGTACGATCGAGTGTACAGTGAA

MT509453.1 Severe acute respiratory syndrome coronavirus 2 isolate SARS-CoV-2/human/USA/MD-HP00020/2020, c	TTCACCGAGGCCACGCGGAGTACGATCGAGTGTACAGTGAA
MT509454.1 Severe acute respiratory syndrome coronavirus 2 isolate SARS-CoV-2/human/USA/MD-HP00023/2020, c	TTCACCGAGGCCACGCGGAGTACGATCGAGTGTACAGTGAA
MT509455.1 Severe acute respiratory syndrome coronavirus 2 isolate SARS-CoV-2/human/USA/MD-HP00024/2020, c	TTCACCGAGGCCACGCGGAGTACGATCGAGTGTACAGTGAA
MT509456.1 Severe acute respiratory syndrome coronavirus 2 isolate SARS-CoV-2/human/USA/MD-HP00030/2020, c	TTCACCGAGGCCACGCGGAGTACGATCGAGTGTACAGTGAA
MT509457.1 Severe acute respiratory syndrome coronavirus 2 isolate SARS-CoV-2/human/USA/MD-HP00036/2020, c	TTCACCGAGGCCACGCGGAGTACGATCGAGTGTACAGTGAA
MT509458.1 Severe acute respiratory syndrome coronavirus 2 isolate SARS-CoV-2/human/USA/MD-HP00037/2020, c	TTCACCGAGGCCACGCGGAGTACGATCGAGTGTACAGTGAA
MT509459.1 Severe acute respiratory syndrome coronavirus 2 isolate SARS-CoV-2/human/USA/MD-HP00038/2020, c	TTCACCGAGGCCACGCGGAGTACGATCGAGTGTACAGTGAA
MT509460.1 Severe acute respiratory syndrome coronavirus 2 isolate SARS-CoV-2/human/USA/MD-HP00041/2020, c	TTCACCGAGGCCACGCGGAGTACGATCGAGTGTACAGTGAA
MT509461.1 Severe acute respiratory syndrome coronavirus 2 isolate SARS-CoV-2/human/USA/DC-HP00043/2020, co	TTCACCGAGGCCACGCGGAGTACGATCGAGTGTACAGTGAA
MT509462.1 Severe acute respiratory syndrome coronavirus 2 isolate SARS-CoV-2/human/USA/MD-HP00044/2020, c	TTCACCGAGGCCACGCGGAGTACGATCGAGTGTACAGTGAA
MT509463.1 Severe acute respiratory syndrome coronavirus 2 isolate SARS-CoV-2/human/USA/DC-HP00006/2020, co	TTCACCGAGGCCACGCGGAGTACGATCGAGTGTACAGTGAA
MT509464.1 Severe acute respiratory syndrome coronavirus 2 isolate SARS-CoV-2/human/USA/DC-HP00007/2020, co	TTCACCGAGGCCACGCGGAGTACGATCGAGTGTACAGTGAA
MT509466.1 Severe acute respiratory syndrome coronavirus 2 isolate SARS-CoV-2/human/USA/MD-HP00015/2020, c	TTCACCGAGGCCACGCGGAGTACGATCGAGTGTACAGTGAA
MT509467.1 Severe acute respiratory syndrome coronavirus 2 isolate SARS-CoV-2/human/USA/DC-HP00022/2020, co	TTCACCGAGGCCACGCGGAGTACGATCGAGTGTACAGTGAA
MT509469.1 Severe acute respiratory syndrome coronavirus 2 isolate SARS-CoV-2/human/USA/MD-HP00068/2020 O	TTCACCGAGGCCACGCGGAGTACGATCGAGTGTACAGTGAA
MT509470.1 Severe acute respiratory syndrome coronavirus 2 isolate SARS-CoV-2/human/USA/MD-HP00069/2020 O	TTCACCGAGGCCACGCGGAGTACGATCGAGTGTACAGTGAA
MT509471.1 Severe acute respiratory syndrome coronavirus 2 isolate SARS-CoV-2/human/USA/DC-HP00070/2020, co	TTCACCGAGGCCACGCGGAGTACGATCGAGTGTACAGTGAA
MT509472.1 Severe acute respiratory syndrome coronavirus 2 isolate SARS-CoV-2/human/USA/DC-HP00072/2020, co	TTCACCGAGGCCACGCGGAGTACGATCGAGTGTACAGTGAA
MT509473.1 Severe acute respiratory syndrome coronavirus 2 isolate SARS-CoV-2/human/USA/DC-HP00074/2020 OR	TTCACCGAGGCCACGCGGAGTACGATCGAGTGTACAGTGAA
MT509474.1 Severe acute respiratory syndrome coronavirus 2 isolate SARS-CoV-2/human/USA/MD-HP00075/2020, c	TTCACCGAGGCCACGCGGAGTACGATCGAGTGTACAGTGAA
MT509475.1 Severe acute respiratory syndrome coronavirus 2 isolate SARS-CoV-2/human/USA/MD-HP00076/2020, c	TTCACCGAGGCCACGCGGAGTACGATCGAGTGTACAGTGAA
MT509476.1 Severe acute respiratory syndrome coronavirus 2 isolate SARS-CoV-2/human/USA/MD-HP00079/2020 O	TTCACCGAGGCCACGCGGAGTACGATCGAGTGTACAGTGAA
MT509477.1 Severe acute respiratory syndrome coronavirus 2 isolate SARS-CoV-2/human/USA/DC-HP00080/2020, co	TTCACCGAGGCCACGCGGAGTACGATCGAGTGTACAGTGAA
MT509478.1 Severe acute respiratory syndrome coronavirus 2 isolate SARS-CoV-2/human/USA/MD-HP00082/2020 O	TTCACCGAGGCCACGCGGAGTACGATCGAGTGTACAGTGAA
MT509479.1 Severe acute respiratory syndrome coronavirus 2 isolate SARS-CoV-2/human/USA/MD-HP00083/2020 O	TTCACCGAGGCCACGCGGAGTACGATCGAGTGTACAGTGAA
MT509480.1 Severe acute respiratory syndrome coronavirus 2 isolate SARS-CoV-2/human/USA/MD-HP00085/2020, c	TTCACCGAGGCCACGCGGAGTACGATCGAGTGTACAGTGAA
MT509481.1 Severe acute respiratory syndrome coronavirus 2 isolate SARS-CoV-2/human/USA/MD-HP00087/2020 O	TTCACCGAGGCCACGCGGAGTACGATCGAGTGTACAGTGAA
MT509482.1 Severe acute respiratory syndrome coronavirus 2 isolate SARS-CoV-2/human/USA/DC-HP00089/2020 OR	TTCACCGAGGCCACGCGGAGTACGATCGAGTGTACAGTGAA
MT509483.1 Severe acute respiratory syndrome coronavirus 2 isolate SARS-CoV-2/human/USA/DC-HP00090/2020 OR	TTCACCGAGGCCACGCGGAGTACGATCGAGTGTACAGTGAA
MT509484.1 Severe acute respiratory syndrome coronavirus 2 isolate SARS-CoV-2/human/USA/DC-HP00099/2020 OR	TTCACCGAGGCCACGCGGAGTACGATCGAGTGTACAGTGAA
MT509485.1 Severe acute respiratory syndrome coronavirus 2 isolate SARS-CoV-2/human/USA/DC-HP00100/2020 OR	TTCACCGAGGCCACGCGGAGTACGATCGAGTGTACAGTGAA
MT509487.1 Severe acute respiratory syndrome coronavirus 2 isolate SARS-CoV-2/human/USA/MD-HP00105/2020 O	TTCACCGAGGCCACGCGGAGTACGATCGAGTGTACAGTGAA
MT509488.1 Severe acute respiratory syndrome coronavirus 2 isolate SARS-CoV-2/human/USA/MD-HP00033/2020, c	TTCACCGAGGCCACGCGGAGTACGATCGAGTGTACAGTGAA

MT509489.1 Severe acute respiratory syndrome coronavirus 2 isolate SARS-CoV-2/human/USA/MD-HP00025/2020, c	TTCACCGAGGCCACGCGGAGTACGATCGAGTGTACAGTGAA
MT509490.1 Severe acute respiratory syndrome coronavirus 2 isolate SARS-CoV-2/human/USA/DC-HP00026/2020, co	TTCACCGAGGCCACGCGGAGTACGATCGAGTGTACAGTGAA
MT509491.1 Severe acute respiratory syndrome coronavirus 2 isolate SARS-CoV-2/human/USA/MD-HP00028/2020, c	TTCACCGAGGCCACGCGGAGTACGATCGAGTGTACAGTGAA
MT509492.1 Severe acute respiratory syndrome coronavirus 2 isolate SARS-CoV-2/human/USA/DC-HP00073/2020, co	TTCACCGAGGCCACGCGGAGTACGATCGAGTGTACAGTGAA
MT509493.1 Severe acute respiratory syndrome coronavirus 2 isolate SARS-CoV-2/human/USA/MD-HP00078/2020, c	TTCACCGAGGCCACGCGGAGTACGATCGAGTGTACAGTGAA
MT509494.1 Severe acute respiratory syndrome coronavirus 2 isolate SARS-CoV-2/human/IND/GBRC110/2020, comp	TTCACCGAGGCCACGCGGAGTACGATCGAGTGTACAGTGAA
MT509496.1 Severe acute respiratory syndrome coronavirus 2 isolate SARS-CoV-2/human/IND/GBRC96/2020, compl	TTCACCGAGGCCACGCGGAGTACGATCGAGTGTACAGTGAA
MT509497.1 Severe acute respiratory syndrome coronavirus 2 isolate SARS-CoV-2/human/IND/GBRC98/2020, compl	TTCACCGAGGCCACGCGGAGTACGATCGAGTGTACAGTGAA
MT509500.1 Severe acute respiratory syndrome coronavirus 2 isolate SARS-CoV-2/human/IND/GBRC109/2020, comp	TTCACCGAGGCCACGCGGAGTACGATCGAGTGTACAGTGAA
MT509501.1 Severe acute respiratory syndrome coronavirus 2 isolate SARS-CoV-2/human/IND/GBRC105/2020, comp	TTCACCGAGGCCACGCGGAGTACGATCGAGTGTACAGTGAA
MT509502.1 Severe acute respiratory syndrome coronavirus 2 isolate SARS-CoV-2/human/IND/GBRC108/2020, comp	TTCACCGAGGCCACGCGGAGTACGATCGAGTGTACAGTGAA
MT509503.1 Severe acute respiratory syndrome coronavirus 2 isolate SARS-CoV-2/human/IND/GBRC94/2020, compl	TTCACCGAGGCCACGCGGAGTACGATCGAGTGTACAGTGAA
MT509504.1 Severe acute respiratory syndrome coronavirus 2 isolate SARS-CoV-2/human/IND/GBRC99/2020, compl	TTCACCGAGGCCACGCGGAGTACGATCGAGTGTACAGTGAA
MT509505.1 Severe acute respiratory syndrome coronavirus 2 isolate SARS-CoV-2/human/IND/GBRC95b/2020, comp	TTCACCGAGGCCACGCGGAGTACGATCGAGTGTACAGTGAA
MT509506.1 Severe acute respiratory syndrome coronavirus 2 isolate SARS-CoV-2/human/IND/GBRC102/2020, comp	TTCACCGAGGCCACACGGAGTACGATCGAGTGTACAGTGAA
MT509507.1 Severe acute respiratory syndrome coronavirus 2 isolate SARS-CoV-2/human/IND/GBRC97a/2020, comp	TTCACCGAGGCCACGCGGAGTACGATCGAGTGTACAGTGAA
MT509509.1 Severe acute respiratory syndrome coronavirus 2 isolate SARS-CoV-2/human/IND/GBRC95a/2020, comp	TTCACCGAGGCCACGCGGAGTACGATCGAGTGTACAGTGAA
MT509510.1 Severe acute respiratory syndrome coronavirus 2 isolate SARS-CoV-2/human/IND/GBRC97b/2020, comp	TTCACCGAGGCCACGCGGAGTACGATCGAGTGTACAGTGAA
MT509511.1 Severe acute respiratory syndrome coronavirus 2 isolate SARS-CoV-2/human/IND/GBRC101/2020, comp	TTCACCGAGGCCACACGGAGTACGATCGAGTGTACAGTGAA
MT509512.1 Severe acute respiratory syndrome coronavirus 2 isolate SARS-CoV-2/human/IND/GBRC111/2020, comp	TTCACCGAGGCCACGCGGAGTACGATCGAGTGTACAGTGAA
MT509650.1 Severe acute respiratory syndrome coronavirus 2 isolate SARS-CoV-2/human/IND/GBRC118/2020, comp	TTCACCGAGGCCACGCGGAGTACGATCGAGTGTACAGTGAA
MT509651.1 Severe acute respiratory syndrome coronavirus 2 isolate SARS-CoV-2/human/IND/GBRC117/2020, comp	TTCACCGAGGCCACGCGGAGTACGATCGAGTGTACAGTGAA
MT509656.1 Severe acute respiratory syndrome coronavirus 2 isolate SARS-CoV-2/human/IND/GBRC113/2020, comp	TTCACCGAGGCCACGCGGAGTACGATCGAGTGTACAGTGAA
MT509657.1 Severe acute respiratory syndrome coronavirus 2 isolate SARS-CoV-2/human/IND/GBRC114/2020, comp	TTCACCGAGGCCACGCGGAGTACGATCGAGTGTACAGTGAA
MT509658.1 Severe acute respiratory syndrome coronavirus 2 isolate SARS-CoV-2/human/IND/GBRC116/2020, comp	TTCACCGAGGCCACGCGGAGTACGATCGAGTGTACAGTGAA
MT509659.1 Severe acute respiratory syndrome coronavirus 2 isolate SARS-CoV-2/human/IND/GBRC115/2020, comp	TTCACCGAGGCCACGCGGAGTACGATCGAGTGTACAGTGAA
MT509669.1 Severe acute respiratory syndrome coronavirus 2 isolate SARS-CoV-2/human/USA/MI-MDHHS-SC20322/	TTCACCGAGGCCACGCGGAGTACGATCGAGTGTACAGTGAA
MT509670.1 Severe acute respiratory syndrome coronavirus 2 isolate SARS-CoV-2/human/USA/MI-MDHHS-SC20323/	TTCACCGAGGCCACGCGGAGTACGATCGAGTGTACAGTGAA
MT509671.1 Severe acute respiratory syndrome coronavirus 2 isolate SARS-CoV-2/human/USA/MI-MDHHS-SC20324/	TTCACCGAGGCCACGCGGAGTACGATCGAGTGTACAGTGAA
MT509672.1 Severe acute respiratory syndrome coronavirus 2 isolate SARS-CoV-2/human/USA/MI-MDHHS-SC20325/	TTCACCGAGGCCACGCGGAGTACGATCGAGTGTACAGTGAA
MT509673.1 Severe acute respiratory syndrome coronavirus 2 isolate SARS-CoV-2/human/USA/MI-MDHHS-SC20326/	TTCACCGAGGCCACGCGGAGTACGATCGAGTGTACAGTGAA
MT509674.1 Severe acute respiratory syndrome coronavirus 2 isolate SARS-CoV-2/human/USA/MI-MDHHS-SC20327/	TTCACCGAGGCCACGCGGAGTACGATCGAGTGTACAGTGAA
MT509675.1 Severe acute respiratory syndrome coronavirus 2 isolate SARS-CoV-2/human/USA/MI-MDHHS-SC20328/	TTCACCGAGGCCACGCGGAGTACGATCGAGTGTACAGTGAA

MT509676.1 Severe acute respiratory syndrome coronavirus 2 isolate SARS-CoV-2/human/USA/MI-MDHHS-SC20329/	TTCACCGAGGCCACGCGGAGTACGATCGAGTGTACAGTGAA
MT509677.1 Severe acute respiratory syndrome coronavirus 2 isolate SARS-CoV-2/human/USA/MI-MDHHS-SC20330/	TTCACCGAGGCCACGCGGAGTACGATCGAGTGTACAGTGAA
MT509678.1 Severe acute respiratory syndrome coronavirus 2 isolate SARS-CoV-2/human/USA/MI-MDHHS-SC20332/	TTCACCGAGGCCACGCGGAGTACGATCGAGTGTACAGTGAA
MT509679.1 Severe acute respiratory syndrome coronavirus 2 isolate SARS-CoV-2/human/USA/MI-MDHHS-SC20333/	TTCACCGAGGCCACGCGGAGTACGATCGAGTGTACAGTGAA
MT509680.1 Severe acute respiratory syndrome coronavirus 2 isolate SARS-CoV-2/human/USA/MI-MDHHS-SC20334/	TTCACCGAGGCCACGCGGAGTACGATCGAGTGTACAGTGAA
MT509681.1 Severe acute respiratory syndrome coronavirus 2 isolate SARS-CoV-2/human/USA/MI-MDHHS-SC20335/	TTCACCGAGGCCACGCGGAGTACGATCGAGTGTACAGTGAA
MT509682.1 Severe acute respiratory syndrome coronavirus 2 isolate SARS-CoV-2/human/USA/MI-MDHHS-SC20336/	TTCACCGAGGCCACGCGGAGTACGATCGAGTGTACAGTGAA
MT509683.1 Severe acute respiratory syndrome coronavirus 2 isolate SARS-CoV-2/human/USA/MI-MDHHS-SC20337/	TTCACCGAGGCCACGCGGAGTACGATCGAGTGTACAGTGAA
MT509684.1 Severe acute respiratory syndrome coronavirus 2 isolate SARS-CoV-2/human/USA/MI-MDHHS-SC20338/	TTCACCGAGGCCACGCGGAGTACGATCGAGTGTACAGTGAA
MT509685.1 Severe acute respiratory syndrome coronavirus 2 isolate SARS-CoV-2/human/USA/MI-MDHHS-SC20339/	TTCACCGAGGCCACGCGGAGTACGATCGAGTGTACAGTGAA
MT509686.1 Severe acute respiratory syndrome coronavirus 2 isolate SARS-CoV-2/human/USA/MI-MDHHS-SC20340/	TTCACCGAGGCCACGCGGAGTACGATCGAGTGTACAGTGAA
MT509687.1 Severe acute respiratory syndrome coronavirus 2 isolate SARS-CoV-2/human/USA/MI-MDHHS-SC20341/	TTCACCGAGGCCACGCGGAGTACGATCGAGTGTACAGTGAA
MT509688.1 Severe acute respiratory syndrome coronavirus 2 isolate SARS-CoV-2/human/USA/MI-MDHHS-SC20342/	TTCACCGAGGCCACGCGGAGTACGATCGAGTGTACAGTGAA
MT509689.1 Severe acute respiratory syndrome coronavirus 2 isolate SARS-CoV-2/human/USA/MI-MDHHS-SC20343/	TTCACCGAGGCCACGCGGAGTACGATCGAGTGTACAGTGAA
MT509690.1 Severe acute respiratory syndrome coronavirus 2 isolate SARS-CoV-2/human/USA/MI-MDHHS-SC20344/	TTCACCGAGGCCACGCGGAGTACGATCGAGTGTACAGTGAA
MT509691.1 Severe acute respiratory syndrome coronavirus 2 isolate SARS-CoV-2/human/USA/AL-MDHHS-SC20345/	TTCACCGAGGCCACGCGGAGTACGATCGAGTGTACAGTGAA
MT509692.1 Severe acute respiratory syndrome coronavirus 2 isolate SARS-CoV-2/human/USA/MI-MDHHS-SC20346/	TTCACCGAGGCCACGCGGAGTACGATCGAGTGTACAGTGAA
MT509693.1 Severe acute respiratory syndrome coronavirus 2 isolate SARS-CoV-2/human/USA/MI-MDHHS-SC20347/	TTCACCGAGGCCACGCGGAGTACGATCGAGTGTACAGTGAA
MT509694.1 Severe acute respiratory syndrome coronavirus 2 isolate SARS-CoV-2/human/USA/MI-MDHHS-SC20348/	TTCACCGAGGCCACGCGGAGTACGATCGAGTGTACAGTGAA
MT509695.1 Severe acute respiratory syndrome coronavirus 2 isolate SARS-CoV-2/human/USA/MI-MDHHS-SC20349/	TTCACCGAGGCCACGCGGAGTACGATCGAGTGTACAGTGAA
MT509696.1 Severe acute respiratory syndrome coronavirus 2 isolate SARS-CoV-2/human/USA/MI-MDHHS-SC20350/	TTCACCGAGGCCACGCGGAGTACGATCGAGTGTACAGTGAA
MT509697.1 Severe acute respiratory syndrome coronavirus 2 isolate SARS-CoV-2/human/USA/MI-MDHHS-SC20351/	TTCACCGAGGCCACGCGGAGTACGATCGAGTGTACAGTGAA
MT509698.1 Severe acute respiratory syndrome coronavirus 2 isolate SARS-CoV-2/human/USA/MI-MDHHS-SC20352/	TTCACCGAGGCCACGCGGAGTACGATCGAGTGTACAGTGAA
MT509699.1 Severe acute respiratory syndrome coronavirus 2 isolate SARS-CoV-2/human/USA/MI-MDHHS-SC20353/	TTCACCGAGGCCACGCGGAGTACGATCGAGTGTACAGTGAA
MT509700.1 Severe acute respiratory syndrome coronavirus 2 isolate SARS-CoV-2/human/USA/MI-MDHHS-SC20354/	TTCACCGAGGCCACGCGGAGTACGATCGAGTGTACAGTGAA
MT509701.1 Severe acute respiratory syndrome coronavirus 2 isolate SARS-CoV-2/human/USA/MI-MDHHS-SC20355/	TTCACCGAGGCCACGCGGAGTACGATCGAGTGTACAGTGAA
MT509702.1 Severe acute respiratory syndrome coronavirus 2 isolate SARS-CoV-2/human/USA/MI-MDHHS-SC20356/	TTCACCGAGGCCACGCGGAGTACGATCGAGTGTACAGTGAA
MT509703.1 Severe acute respiratory syndrome coronavirus 2 isolate SARS-CoV-2/human/USA/MI-MDHHS-SC20357/	TTCACCGAGGCCACGCGGAGTACGATCGAGTGTACAGTGAA
MT509704.1 Severe acute respiratory syndrome coronavirus 2 isolate SARS-CoV-2/human/USA/MI-MDHHS-SC20358/	TTCACCGAGGCCACGCGGAGTACGATCGAGTGTACAGTGAA
MT509958.1 Severe acute respiratory syndrome coronavirus 2 isolate SARS-CoV-2/human/BGD/NIB_01/2020, comple	TTCACCGAGGCCACGCGGAGTACGATCGAGTGTACAGTGAA
MT509959.1 Severe acute respiratory syndrome coronavirus 2 isolate SARS-CoV-2/human/IND/GBRC103/2020, comp	TTCACCGAGGCCACGCGGAGTACGATCGAGTGTACAGTGAA
MT510719.1 Severe acute respiratory syndrome coronavirus 2 isolate SARS-CoV-2/human/USA/hCoV-19-USA-CA-UCS	TTCACCGAGGCCACGCGGAGTACGATCGAGTGTACAGTGAA
MT510720.1 Severe acute respiratory syndrome coronavirus 2 isolate SARS-CoV-2/human/USA/hCoV-19-USA-CA-UCS	TTCACCGAGGCCACGCGGAGTACGATCGAGTGTACAGTGAA

MT510722.1 Severe acute respiratory syndrome coronavirus 2 isolate SARS-CoV-2/human/USA/hCoV-19-USA-CA-UCS	TTCACCGAGGCCACGCGGAGTACGATCGAGTGTACAGTGAA
MT510725.1 Severe acute respiratory syndrome coronavirus 2 isolate SARS-CoV-2/human/USA/hCoV-19-USA-CA-UCS	TTCACCGAGGCCACGCGGAGTACGATCGAGTGTACAGTGAA
MT510726.1 Severe acute respiratory syndrome coronavirus 2 isolate SARS-CoV-2/human/USA/hCoV-19-USA-CA-UCS	TTCACCGAGGCCACGCGGAGTACGATCGAGTGTACAGTGAA
MT510727.1 Severe acute respiratory syndrome coronavirus 2 isolate SARS-CoV-2/human/CHN/SARS-CoV-2-MZ02, M	TTCACCGAGGCCACGCGGAGTACGATCGAGTGTACAGTGAA
MT510728.1 Severe acute respiratory syndrome coronavirus 2 isolate SARS-CoV-2/human/CHN/SARS-CoV-2-MZ01, M	TTCACCGAGGCCACGCGGAGTACGATCGAGTGTACAGTGAA
MT510744.1 Severe acute respiratory syndrome coronavirus 2 isolate SARS-CoV-2/human/HKG/HKSH0003/2020 ORF	TTCACCGAGGCCACGCGGAGTACGATCGAGTGTACAGTGAA
MT510999.1 Severe acute respiratory syndrome coronavirus 2 isolate SARS-CoV-2/human/NLD/Leiden-0002/2020, co	TTCACCGAGGCCACGCGGAGTACGATCGAGTGTACAGTGAA
MT511069.1 Severe acute respiratory syndrome coronavirus 2 isolate SARS-CoV-2/human/POL/PL_P13/2020, comple	TTCACCGAGGCCACGCGGAGTACGATCGAGTGTACAGTGAA
MT511082.1 Severe acute respiratory syndrome coronavirus 2 isolate SARS-CoV-2/human/POL/PL_P26/2020, comple	TTCACCGAGGCCACGCGGAGTACGATCGAGTGTACAGTGAA
MT511686.1 Severe acute respiratory syndrome coronavirus 2 isolate SARS-CoV-2/Human/USA/FL-BPHL-0123/2020	TTCACCGAGGCCACGCGGAGTACGATCGAGTGTACAGTGAA
MT511688.1 Severe acute respiratory syndrome coronavirus 2 isolate SARS-CoV-2/Human/USA/FL-BPHL-0125/2020	TTCACCGAGGCCACGCGGAGTACGATCGAGTGTACAGTGAA
MT511691.1 Severe acute respiratory syndrome coronavirus 2 isolate SARS-CoV-2/Human/USA/FL-BPHL-0128/2020	TTCACCGAGGCCACGCGGAGTACGATCGAGTGTACAGTGAA
MT511693.1 Severe acute respiratory syndrome coronavirus 2 isolate SARS-CoV-2/Human/USA/FL-BPHL-0130/2020	TTCACCGAGGCCACGCGGAGTACGATCGAGTGTACAGTGAA
MT511694.1 Severe acute respiratory syndrome coronavirus 2 isolate SARS-CoV-2/Human/USA/FL-BPHL-0131/2020	TTCACCGAGGCCACGCGGAGTACGATCGAGTGTACAGTGAA
MT511698.1 Severe acute respiratory syndrome coronavirus 2 isolate SARS-CoV-2/Human/USA/FL-BPHL-0135/2020	TTCACCGAGGCCACGCGGAGTACGATCGAGTGTACAGTGAA
MT511700.1 Severe acute respiratory syndrome coronavirus 2 isolate SARS-CoV-2/Human/USA/FL-BPHL-0137/2020	TTCACCGAGGCCACGCGGAGTACGATCGAGTGTACAGTGAA
MT512415.1 Severe acute respiratory syndrome coronavirus 2 isolate SARS-CoV-2/human/USA/FL-CDC-2316/2020, c	TTCACCGAGGCCACGCGGAGTACGATCGAGTGTACAGTGAA
MT512416.1 Severe acute respiratory syndrome coronavirus 2 isolate SARS-CoV-2/human/USA/MN-CDC-0106/2020,	TTCACCGAGGCCACGCGGAGTACGATCGAGTGTACAGTGAA
MT512417.1 Severe acute respiratory syndrome coronavirus 2 isolate SARS-CoV-2/human/USA/IL-CDC-1732/2020, co	TTCACCGAGGCCACGCGGAGTACGATCGAGTGTACAGTGAA
MT512420.1 Severe acute respiratory syndrome coronavirus 2 isolate SARS-CoV-2/human/USA/GA-CDC-8816/2020, c	TTCACCGAGGCCACGCGGAGTACGATCGAGTGTACAGTGAA
MT512421.1 Severe acute respiratory syndrome coronavirus 2 isolate SARS-CoV-2/human/USA/VI-CDC-3661/2020, co	TTCACCGAGGCCACGCGGAGTACGATCGAGTGTACAGTGAA
MT512422.1 Severe acute respiratory syndrome coronavirus 2 isolate SARS-CoV-2/human/USA/VI-CDC-3705/2020, co	TTCACCGAGGCCACGCGGAGTACGATCGAGTGTACAGTGAA
MT512423.1 Severe acute respiratory syndrome coronavirus 2 isolate SARS-CoV-2/human/USA/VI-CDC-3688/2020, co	TTCACCGAGGCCACGCGGAGTACGATCGAGTGTACAGTGAA
MT512424.1 Severe acute respiratory syndrome coronavirus 2 isolate SARS-CoV-2/human/USA/NC-CDC-6999/2020, c	TTCACCGAGGCCACGCGGAGTACGATCGAGTGTACAGTGAA
MT512425.1 Severe acute respiratory syndrome coronavirus 2 isolate SARS-CoV-2/human/USA/GA-CDC-2059/2020, c	TTCACCGAGGCCACGCGGAGTACGATCGAGTGTACAGTGAA
MT512426.1 Severe acute respiratory syndrome coronavirus 2 isolate SARS-CoV-2/human/USA/NC-CDC-0034/2020, c	TTCACCGAGGCCACGCGGAGTACGATCGAGTGTACAGTGAA
MT512427.1 Severe acute respiratory syndrome coronavirus 2 isolate SARS-CoV-2/human/USA/CO-CDC-5610/2020, c	TTCACCGAGGCCACGCGGAGTACGATCGAGTGTACAGTGAA
MT512428.1 Severe acute respiratory syndrome coronavirus 2 isolate SARS-CoV-2/human/USA/CO-CDC-5667/2020, c	TTCACCGAGGCCACGCGGAGTACGATCGAGTGTACAGTGAA
MT512429.1 Severe acute respiratory syndrome coronavirus 2 isolate SARS-CoV-2/human/USA/CO-CDC-5780/2020, c	TTCACCGAGGCCACGCGGAGTACGATCGAGTGTACAGTGAA
MT512430.1 Severe acute respiratory syndrome coronavirus 2 isolate SARS-CoV-2/human/USA/CO-CDC-5607/2020, c	TTCACCGAGGCCACGCGGAGTACGATCGAGTGTACAGTGAA
MT512431.1 Severe acute respiratory syndrome coronavirus 2 isolate SARS-CoV-2/human/USA/IN-CDC-2013/2020, c	TTCACCGAGGCCACGCGGAGTACGATCGAGTGTACAGTGAA
MT512433.1 Severe acute respiratory syndrome coronavirus 2 isolate SARS-CoV-2/human/USA/TX-CDC-3008/2020, c	TTCACCGAGGCCACGCGGAGTACGATCGAGTGTACAGTGAA
MT512434.1 Severe acute respiratory syndrome coronavirus 2 isolate SARS-CoV-2/human/USA/LA-CDC-0499/2020, c	TTCACCGAGGCCACGCGGAGTACGATCGAGTGTACAGTGAA

MT512435.1 Severe acute respiratory syndrome coronavirus 2 isolate SARS-CoV-2/human/USA/LA-CDC-0757/2020, c	TTCACCGAGGCCACGCGGAGTACGATCGAGTGTACAGTGAA
MT512436.1 Severe acute respiratory syndrome coronavirus 2 isolate SARS-CoV-2/human/USA/MN-CDC-0103/2020,	TTCACCGAGGCCACGCGGAGTACGATCGAGTGTACAGTGAA
MT512437.1 Severe acute respiratory syndrome coronavirus 2 isolate SARS-CoV-2/human/USA/NM-CDC-3589/2020,	TTCACCGAGGCCACGCGGAGTACGATCGAGTGTACAGTGAA
MT512438.1 Severe acute respiratory syndrome coronavirus 2 isolate SARS-CoV-2/human/USA/FL-CDC-6318/2020, c	TTCACCGAGGCCACGCGGAGTACGATCGAGTGTACAGTGAA
MT512439.1 Severe acute respiratory syndrome coronavirus 2 isolate SARS-CoV-2/human/USA/FL-CDC-2718/2020, c	TTCACCGAGGCCACGCGGAGTACGATCGAGTGTACAGTGAA
MT512440.1 Severe acute respiratory syndrome coronavirus 2 isolate SARS-CoV-2/human/USA/TX-CDC-3009/2020, c	TTCACCGAGGCCACGCGGAGTACGATCGAGTGTACAGTGAA
MT512441.1 Severe acute respiratory syndrome coronavirus 2 isolate SARS-CoV-2/human/USA/MN-CDC-0108/2020,	TTCACCGAGGCCACGCGGAGTACGATCGAGTGTACAGTGAA
MT512442.1 Severe acute respiratory syndrome coronavirus 2 isolate SARS-CoV-2/human/USA/KS-CDC-4589/2020, c	TTCACCGAGGCCACGCGGAGTACGATCGAGTGTACAGTGAA
MT512443.1 Severe acute respiratory syndrome coronavirus 2 isolate SARS-CoV-2/human/USA/TX-CDC-8001/2020, c	TTCACCGAGGCCACGCGGAGTACGATCGAGTGTACAGTGAA
MT512444.1 Severe acute respiratory syndrome coronavirus 2 isolate SARS-CoV-2/human/USA/NJ-CDC-3036/2020, c	TTCACCGAGGCCACGCGGAGTACGATCGAGTGTACAGTGAA
MT512445.1 Severe acute respiratory syndrome coronavirus 2 isolate SARS-CoV-2/human/USA/NJ-CDC-7300/2020, c	TTCACCGAGGCCACGCGGAGTACGATCGAGTGTACAGTGAA
MT512446.1 Severe acute respiratory syndrome coronavirus 2 isolate SARS-CoV-2/human/USA/AZ-CDC-1911/2020, c	TTCACCGAGGCCACGCGGAGTACGATCGAGTGTACAGTGAA
MT512447.1 Severe acute respiratory syndrome coronavirus 2 isolate SARS-CoV-2/human/USA/VI-CDC-3588/2020, c	TTCACCGAGGCCACGCGGAGTACGATCGAGTGTACAGTGAA
MT512645.1 Severe acute respiratory syndrome coronavirus 2 isolate SARS-CoV-2/human/USA/VI-CDC-4670/2020, c	TTCACCGAGGCCACGCGGAGTACGATCGAGTGTACAGTGAA
MT513758.1 Severe acute respiratory syndrome coronavirus 2 isolate SARS-CoV-2/Humain/MAR/OUA677_19/2020, c	TTCACCGAGGCCACGCGGAGTACGATCGAGTGTACAGTGAA
MT517419.1 Severe acute respiratory syndrome coronavirus 2 isolate SARS-CoV-2/human/HKG/HKSH0007/2020 ORF	TTCACCGAGGCCACGCGGAGTACGATCGAGTGTACAGTGAA
MT517420.1 Severe acute respiratory syndrome coronavirus 2 isolate SARS-CoV-2/human/CZE/IAB_1/2020, complete	TTCACCGAGGCCACGCGGAGTACGATCGAGTGTACAGTGAA
MT517421.1 Severe acute respiratory syndrome coronavirus 2 isolate SARS-CoV-2/human/CZE/IAB_4/2020, complete	TTCACCGAGGCCACGCGGAGTACGATCGAGTGTACAGTGAA
MT517422.1 Severe acute respiratory syndrome coronavirus 2 isolate SARS-CoV-2/human/CZE/IAB_8/2020, complete	TTCACCGAGGCCACGCGGAGTACGATCGAGTGTACAGTGAA
MT517423.1 Severe acute respiratory syndrome coronavirus 2 isolate SARS-CoV-2/human/CZE/IAB_9/2020, complete	TTCACCGAGGCCACGCGGAGTACGATCGAGTGTACAGTGAA
MT517424.1 Severe acute respiratory syndrome coronavirus 2 isolate SARS-CoV-2/human/CZE/IAB_10/2020, comple	TTCACCGAGGCCACGCGGAGTACGATCGAGTGTACAGTGAA
MT517425.1 Severe acute respiratory syndrome coronavirus 2 isolate SARS-CoV-2/human/CZE/IAB_12/2020, comple	TTCACCGAGGCCACGCGGAGTACGATCGAGTGTACAGTGAA
MT517426.1 Severe acute respiratory syndrome coronavirus 2 isolate SARS-CoV-2/human/CZE/IAB_14/2020, comple	TTCACCGAGGCCACGCGGAGTACGATCGAGTGTACAGTGAA
MT517427.1 Severe acute respiratory syndrome coronavirus 2 isolate SARS-CoV-2/human/CZE/IAB_15/2020, comple	TTCACCGAGGCCACGCGGAGTACGATCGAGTGTACAGTGAA
MT517428.1 Severe acute respiratory syndrome coronavirus 2 isolate SARS-CoV-2/human/CZE/IAB_16/2020, comple	TTCACCGAGGCCACGCGGAGTACGATCGAGTGTACAGTGAA
MT517429.1 Severe acute respiratory syndrome coronavirus 2 isolate SARS-CoV-2/human/CZE/IAB_17/2020, comple	TTCACCGAGGCCACGCGGAGTACGATCGAGTGTACAGTGAA
MT517430.1 Severe acute respiratory syndrome coronavirus 2 isolate SARS-CoV-2/human/CZE/IAB_18/2020, comple	TTCACCGAGGCCACGCGGAGTACGATCGAGTGTACAGTGAA
MT517431.1 Severe acute respiratory syndrome coronavirus 2 isolate SARS-CoV-2/human/CZE/IAB_20/2020, comple	TTCACCGAGGCCACGCGGAGTACGATCGAGTGTACAGTGAA
MT517432.1 Severe acute respiratory syndrome coronavirus 2 isolate SARS-CoV-2/human/CZE/IAB_21/2020, comple	TTCACCGAGGCCACGCGGAGTACGATCGAGTGTACAGTGAA
MT517433.1 Severe acute respiratory syndrome coronavirus 2 isolate SARS-CoV-2/human/CZE/IAB_22/2020, comple	TTCACCGAGGCCACGCGGAGTACGATCGAGTGTACAGTGAA
MT517434.1 Severe acute respiratory syndrome coronavirus 2 isolate SARS-CoV-2/human/CZE/IAB_23/2020, comple	TTCACCGAGGCCACGCGGAGTACGATCGAGTGTACAGTGAA
MT517436.1 Severe acute respiratory syndrome coronavirus 2 isolate SARS-CoV-2/human/TWN/CGMH-CGU-26/2020	TTCACCGAGGCCACGCGGAGTACGATCGAGTGTACAGTGAA
MT517437.1 Severe acute respiratory syndrome coronavirus 2 isolate SARS-CoV-2/human/TWN/CGMH-CGU-27/2020	TTCACCGAGGCCACGCGGAGTACGATCGAGTGTACAGTGAA

MT520172.1 Severe acute respiratory syndrome coronavirus 2 isolate SARS-CoV-2/human/USA/MA_MGH_00568/20	TTCACCGAGGCCACGCGGAGTACGATCGAGTGTACAGTGAA
MT520173.1 Severe acute respiratory syndrome coronavirus 2 isolate SARS-CoV-2/human/USA/MA_MGH_00277/20	TTCACCGAGGCCACGCGGAGTACGATCGAGTGTACAGTGAA
MT520174.1 Severe acute respiratory syndrome coronavirus 2 isolate SARS-CoV-2/human/USA/MA_MGH_00461/20	TTCACCGAGGCCACGCGGAGTACGATCGAGTGTACAGTGAA
MT520175.1 Severe acute respiratory syndrome coronavirus 2 isolate SARS-CoV-2/human/USA/MA_MGH_00278/20	TTCACCGAGGCCACGCGGAGTACGATCGAGTGTACAGTGAA
MT520176.1 Severe acute respiratory syndrome coronavirus 2 isolate SARS-CoV-2/human/USA/MA_MGH_00016/20	TTCACCGAGGCCACGCGGAGTACGATCGAGTGTACAGTGAA
MT520177.1 Severe acute respiratory syndrome coronavirus 2 isolate SARS-CoV-2/human/USA/MA_MGH_00018/20	TTCACCGAGGCCACGCGGAGTACGATCGAGTGTACAGTGAA
MT520178.1 Severe acute respiratory syndrome coronavirus 2 isolate SARS-CoV-2/human/USA/MA_MGH_00426/20	TTCACCGAGGCCACGCGGAGTACGATCGAGTGTACAGTGAA
MT520179.1 Severe acute respiratory syndrome coronavirus 2 isolate SARS-CoV-2/human/USA/MA_MGH_00050/20	TTCACCGAGGCCACGCGGAGTACGATCGAGTGTACAGTGAA
MT520180.1 Severe acute respiratory syndrome coronavirus 2 isolate SARS-CoV-2/human/USA/MA_MGH_00042/20	TTCACCGAGGCCACGCGGAGTACGATCGAGTGTACAGTGAA
MT520181.1 Severe acute respiratory syndrome coronavirus 2 isolate SARS-CoV-2/human/USA/MA_MGH_00707/20	TTCACCGAGGCCACGCGGAGTACGATCGAGTGTACAGTGAA
MT520182.1 Severe acute respiratory syndrome coronavirus 2 isolate SARS-CoV-2/human/USA/MA_MGH_00157/20	TTCACCGAGGCCACGCGGAGTACGATCGAGTGTACAGTGAA
MT520183.1 Severe acute respiratory syndrome coronavirus 2 isolate SARS-CoV-2/human/USA/MA_MGH_00208/20	TTCACCGAGGCCACGCGGAGTACGATCGAGTGTACAGTGAA
MT520184.1 Severe acute respiratory syndrome coronavirus 2 isolate SARS-CoV-2/human/USA/MA_MGH_00067/20	TTCACCGAGGCCACGCGGAGTACGATCGAGTGTACAGTGAA
MT520186.1 Severe acute respiratory syndrome coronavirus 2 isolate SARS-CoV-2/human/USA/MA_MGH_00031/20	TTCACCGAGGCCACGCGGAGTACGATCGAGTGTACAGTGAA
MT520187.1 Severe acute respiratory syndrome coronavirus 2 isolate SARS-CoV-2/human/USA/MA_MGH_00708/20	TTCACCGAGGCCACGCGGAGTACGATCGAGTGTACAGTGAA
MT520188.1 Severe acute respiratory syndrome coronavirus 2 isolate SARS-CoV-2/human/USA/MA_MGH_00184/20	TTCACCGAGGCCACGCGGAGTACGATCGAGTGTACAGTGAA
MT520189.1 Severe acute respiratory syndrome coronavirus 2 isolate SARS-CoV-2/human/USA/MA_MGH_00143/20	TTCACCGAGGCCACGCGGAGTACGATCGAGTGTACAGTGAA
MT520190.1 Severe acute respiratory syndrome coronavirus 2 isolate SARS-CoV-2/human/USA/MA_MGH_00588/20	TTCACCGAGGCCACGCGGAGTACGATCGAGTGTACAGTGAA
MT520191.1 Severe acute respiratory syndrome coronavirus 2 isolate SARS-CoV-2/human/USA/MA_MGH_00440/20	TTCACCGAGGCCACGCGGAGTACGATCGAGTGTACAGTGAA
MT520192.1 Severe acute respiratory syndrome coronavirus 2 isolate SARS-CoV-2/human/USA/MA_MGH_00417/20	TTCACCGAGGCCACGCGGAGTACGATCGAGTGTACAGTGAA
MT520193.1 Severe acute respiratory syndrome coronavirus 2 isolate SARS-CoV-2/human/USA/MA_MGH_00678/20	TTCACCGAGGCCACGCGGAGTACGATCGAGTGTACAGTGAA
MT520194.1 Severe acute respiratory syndrome coronavirus 2 isolate SARS-CoV-2/human/USA/MA_MGH_00069/20	TTCACCGAGGCCACGCGGAGTACGATCGAGTGTACAGTGAA
MT520195.1 Severe acute respiratory syndrome coronavirus 2 isolate SARS-CoV-2/human/USA/MA_MGH_00656/20	TTCACCGAGGCCACGCGGAGTACGATCGAGTGTACAGTGAA
MT520196.1 Severe acute respiratory syndrome coronavirus 2 isolate SARS-CoV-2/human/USA/MA_MGH_00258/20	TTCACCGAGGCCACGCGGAGTACGATCGAGTGTACAGTGAA
MT520197.1 Severe acute respiratory syndrome coronavirus 2 isolate SARS-CoV-2/human/USA/MA_MGH_00080/20	TTCACCGAGGCCACGCGGAGTACGATCGAGTGTACAGTGAA
MT520198.1 Severe acute respiratory syndrome coronavirus 2 isolate SARS-CoV-2/human/USA/MA_MGH_00711/20	TTCACCGAGGCCACGCGGAGTACGATCGAGTGTACAGTGAA
MT520199.1 Severe acute respiratory syndrome coronavirus 2 isolate SARS-CoV-2/human/USA/MA_MGH_00182/20	TTCACCGAGGCCACGCGGAGTACGATCGAGTGTACAGTGAA
MT520200.1 Severe acute respiratory syndrome coronavirus 2 isolate SARS-CoV-2/human/USA/MA_MGH_00562/20	TTCACCGAGGCCACGCGGAGTACGATCGAGTGTACAGTGAA
MT520201.1 Severe acute respiratory syndrome coronavirus 2 isolate SARS-CoV-2/human/USA/MA_MGH_00065/20	TTCACCGAGGCCACGCGGAGTACGATCGAGTGTACAGTGAA
MT520202.1 Severe acute respiratory syndrome coronavirus 2 isolate SARS-CoV-2/human/USA/MA_MGH_00228/20	TTCACCGAGGCCACGCGGAGTACGATCGAGTGTACAGTGAA
MT520203.1 Severe acute respiratory syndrome coronavirus 2 isolate SARS-CoV-2/human/USA/MA_MGH_00100/20	TTCACCGAGGCCACGCGGAGTACGATCGAGTGTACAGTGAA
MT520204.1 Severe acute respiratory syndrome coronavirus 2 isolate SARS-CoV-2/human/USA/MA_MGH_00627/20	TTCACCGAGGCCACGCGGAGTACGATCGAGTGTACAGTGAA
MT520205.1 Severe acute respiratory syndrome coronavirus 2 isolate SARS-CoV-2/human/USA/MA_MGH_00706/20	TTCACCGAGGCCACGCGGAGTACGATCGAGTGTACAGTGAA

MT520206.1 Severe acute respiratory syndrome coronavirus 2 isolate SARS-CoV-2/human/USA/MA_MGH_00267/202	TTCACCGAGGCCACGCGGAGTACGATCGAGTGTACAGTGAA
MT520207.1 Severe acute respiratory syndrome coronavirus 2 isolate SARS-CoV-2/human/USA/MA_MGH_00687/202	TTCACCGAGGCCACGCGGAGTACGATCGAGTGTACAGTGAA
MT520208.1 Severe acute respiratory syndrome coronavirus 2 isolate SARS-CoV-2/human/USA/MA_MGH_00289/202	TTCACCGAGGCCACGCGGAGTACGATCGAGTGTACAGTGAA
MT520209.1 Severe acute respiratory syndrome coronavirus 2 isolate SARS-CoV-2/human/USA/MA_MGH_00611/202	TTCACCGAGGCCACGCGGAGTACGATCGAGTGTACAGTGAA
MT520210.1 Severe acute respiratory syndrome coronavirus 2 isolate SARS-CoV-2/human/USA/MA_MGH_00661/202	TTCACCGAGGCCACGCGGAGTACGATCGAGTGTACAGTGAA
MT520212.1 Severe acute respiratory syndrome coronavirus 2 isolate SARS-CoV-2/human/USA/MA_MGH_00628/202	TTCACCGAGGCCACGCGGAGTACGATCGAGTGTACAGTGAA
MT520213.1 Severe acute respiratory syndrome coronavirus 2 isolate SARS-CoV-2/human/USA/MA_MGH_00544/202	TTCACCGAGGCCACGCGGAGTACGATCGAGTGTACAGTGAA
MT520214.1 Severe acute respiratory syndrome coronavirus 2 isolate SARS-CoV-2/human/USA/MA_MGH_00651/202	TTCACCGAGGCCACGCGGAGTACGATCGAGTGTACAGTGAA
MT520215.1 Severe acute respiratory syndrome coronavirus 2 isolate SARS-CoV-2/human/USA/MA_MGH_00569/202	TTCACCGAGGCCACGCGGAGTACGATCGAGTGTACAGTGAA
MT520216.1 Severe acute respiratory syndrome coronavirus 2 isolate SARS-CoV-2/human/USA/MA_MGH_00266/202	TTCACCGAGGCCACGCGGAGTACGATCGAGTGTACAGTGAA
MT520217.1 Severe acute respiratory syndrome coronavirus 2 isolate SARS-CoV-2/human/USA/MA_MGH_00210/202	TTCACCGAGGCCACGCGGAGTACGATCGAGTGTACAGTGAA
MT520218.1 Severe acute respiratory syndrome coronavirus 2 isolate SARS-CoV-2/human/USA/MA_MGH_00054/202	TTCACCGAGGCCACGCGGAGTACGATCGAGTGTACAGTGAA
MT520219.1 Severe acute respiratory syndrome coronavirus 2 isolate SARS-CoV-2/human/USA/MA_MGH_00274/202	TTCACCGAGGCCACGCGGAGTACGATCGAGTGTACAGTGAA
MT520220.1 Severe acute respiratory syndrome coronavirus 2 isolate SARS-CoV-2/human/USA/MA_MGH_00086/202	TTCACCGAGGCCACGCGGAGTACGATCGAGTGTACAGTGAA
MT520221.1 Severe acute respiratory syndrome coronavirus 2 isolate SARS-CoV-2/human/USA/MA_MGH_00683/202	TTCACCGAGGCCACGCGGAGTACGATCGAGTGTACAGTGAA
MT520222.1 Severe acute respiratory syndrome coronavirus 2 isolate SARS-CoV-2/human/USA/MA_MGH_00242/202	TTCACCGAGGCCACGCGGAGTACGATCGAGTGTACAGTGAA
MT520223.1 Severe acute respiratory syndrome coronavirus 2 isolate SARS-CoV-2/human/USA/MA_MGH_00642/202	TTCACCGAGGCCACGCGGAGTACGATCGAGTGTACAGTGAA
MT520224.1 Severe acute respiratory syndrome coronavirus 2 isolate SARS-CoV-2/human/USA/MA_MGH_00023/202	TTCACCGAGGCCACGCGGAGTACGATCGAGTGTACAGTGAA
MT520226.1 Severe acute respiratory syndrome coronavirus 2 isolate SARS-CoV-2/human/USA/MA_MGH_00302/202	TTCACCGAGGCCACGCGGAGTACGATCGAGTGTACAGTGAA
MT520228.1 Severe acute respiratory syndrome coronavirus 2 isolate SARS-CoV-2/human/USA/MA_MGH_00675/202	TTCACCGAGGCCACGCGGAGTACGATCGAGTGTACAGTGAA
MT520229.1 Severe acute respiratory syndrome coronavirus 2 isolate SARS-CoV-2/human/USA/MA_MGH_00013/202	TTCACCGAGGCCACGCGGAGTACGATCGAGTGTACAGTGAA
MT520230.1 Severe acute respiratory syndrome coronavirus 2 isolate SARS-CoV-2/human/USA/MA_MGH_00288/202	TTCACCGAGGCCACGCGGAGTACGATCGAGTGTACAGTGAA
MT520231.1 Severe acute respiratory syndrome coronavirus 2 isolate SARS-CoV-2/human/USA/MA_MGH_00502/202	TTCACCGAGGCCACGCGGAGTACGATCGAGTGTACAGTGAA
MT520232.1 Severe acute respiratory syndrome coronavirus 2 isolate SARS-CoV-2/human/USA/MA_MGH_00297/202	TTCACCGAGGCCACGCGGAGTACGATCGAGTGTACAGTGAA
MT520233.1 Severe acute respiratory syndrome coronavirus 2 isolate SARS-CoV-2/human/USA/MA_MGH_00433/202	TTCACCGAGGCCACGCGGAGTACGATCGAGTGTACAGTGAA
MT520234.1 Severe acute respiratory syndrome coronavirus 2 isolate SARS-CoV-2/human/USA/MA_MGH_00467/202	TTCACCGAGGCCACGCGGAGTACGATCGAGTGTACAGTGAA
MT520235.1 Severe acute respiratory syndrome coronavirus 2 isolate SARS-CoV-2/human/USA/MA_MGH_00264/202	TTCACCGAGGCCACGCGGAGTACGATCGAGTGTACAGTGAA
MT520236.1 Severe acute respiratory syndrome coronavirus 2 isolate SARS-CoV-2/human/USA/MA_MGH_00603/202	TTCACCGAGGCCACGCGGAGTACGATCGAGTGTACAGTGAA
MT520237.1 Severe acute respiratory syndrome coronavirus 2 isolate SARS-CoV-2/human/USA/MA_MGH_00691/202	TTCACCGAGGCCACGCGGAGTACGATCGAGTGTACAGTGAA
MT520238.1 Severe acute respiratory syndrome coronavirus 2 isolate SARS-CoV-2/human/USA/MA_MGH_00602/202	TTCACCGAGGCCACGCGGAGTACGATCGAGTGTACAGTGAA
MT520239.1 Severe acute respiratory syndrome coronavirus 2 isolate SARS-CoV-2/human/USA/MA_MGH_00269/202	TTCACCGAGGCCACGCGGAGTACGATCGAGTGTACAGTGAA
MT520240.1 Severe acute respiratory syndrome coronavirus 2 isolate SARS-CoV-2/human/USA/MA_MGH_00666/202	TTCACCGAGGCCACGCGGAGTACGATCGAGTGTACAGTGAA
MT520243.1 Severe acute respiratory syndrome coronavirus 2 isolate SARS-CoV-2/human/USA/MA_MGH_00471/202	TTCACCGAGGCCACGCGGAGTACGATCGAGTGTACAGTGAA

MT520244.1 Severe acute respiratory syndrome coronavirus 2 isolate SARS-CoV-2/human/USA/MA_MGH_00285/202	TTCACCGAGGCCACGCGGAGTACGATCGAGTGTACAGTGAA
MT520245.1 Severe acute respiratory syndrome coronavirus 2 isolate SARS-CoV-2/human/USA/MA_MGH_00145/202	TTCACCGAGGCCACGCGGAGTACGATCGAGTGTACAGTGAA
MT520246.1 Severe acute respiratory syndrome coronavirus 2 isolate SARS-CoV-2/human/USA/MA_MGH_00291/202	TTCACCGAGGCCACGCGGAGTACGATCGAGTGTACAGTGAA
MT520247.1 Severe acute respiratory syndrome coronavirus 2 isolate SARS-CoV-2/human/USA/MA_MGH_00276/202	TTCACCGAGGCCACGCGGAGTACGATCGAGTGTACAGTGAA
MT520248.1 Severe acute respiratory syndrome coronavirus 2 isolate SARS-CoV-2/human/USA/MA_MGH_00254/202	TTCACCGAGGCCACGCGGAGTACGATCGAGTGTACAGTGAA
MT520249.1 Severe acute respiratory syndrome coronavirus 2 isolate SARS-CoV-2/human/USA/MA_MGH_00059/202	TTCACCGAGGCCACGCGGAGTACGATCGAGTGTACAGTGAA
MT520250.1 Severe acute respiratory syndrome coronavirus 2 isolate SARS-CoV-2/human/USA/MA_MGH_00055/202	TTCACCGAGGCCACGCGGAGTACGATCGAGTGTACAGTGAA
MT520251.1 Severe acute respiratory syndrome coronavirus 2 isolate SARS-CoV-2/human/USA/MA_MGH_00466/202	TTCACCGAGGCCACGCGGAGTACGATCGAGTGTACAGTGAA
MT520252.1 Severe acute respiratory syndrome coronavirus 2 isolate SARS-CoV-2/human/USA/MA_MGH_00464/202	TTCACCGAGGCCACGCGGAGTACGATCGAGTGTACAGTGAA
MT520253.1 Severe acute respiratory syndrome coronavirus 2 isolate SARS-CoV-2/human/USA/MA_MGH_00530/202	TTCACCGAGGCCACGCGGAGTACGATCGAGTGTACAGTGAA
MT520254.1 Severe acute respiratory syndrome coronavirus 2 isolate SARS-CoV-2/human/USA/MA_MGH_00174/202	TTCACCGAGGCCACGCGGAGTACGATCGAGTGTACAGTGAA
MT520255.1 Severe acute respiratory syndrome coronavirus 2 isolate SARS-CoV-2/human/USA/MA_MGH_00570/202	TTCACCGAGGCCACGCGGAGTACGATCGAGTGTACAGTGAA
MT520256.1 Severe acute respiratory syndrome coronavirus 2 isolate SARS-CoV-2/human/USA/MA_MGH_00283/202	TTCACCGAGGCCACGCGGAGTACGATCGAGTGTACAGTGAA
MT520257.1 Severe acute respiratory syndrome coronavirus 2 isolate SARS-CoV-2/human/USA/MA_MGH_00542/202	TTCACCGAGGCCACGCGGAGTACGATCGAGTGTACAGTGAA
MT520258.1 Severe acute respiratory syndrome coronavirus 2 isolate SARS-CoV-2/human/USA/MA_MGH_00161/202	TTCACCGAGGCCACGCGGAGTACGATCGAGTGTACAGTGAA
MT520259.1 Severe acute respiratory syndrome coronavirus 2 isolate SARS-CoV-2/human/USA/MA_MGH_00089/202	TTCACCGAGGCCACGCGGAGTACGATCGAGTGTACAGTGAA
MT520260.1 Severe acute respiratory syndrome coronavirus 2 isolate SARS-CoV-2/human/USA/MA_MGH_00488/202	TTCACCGAGGCCACGCGGAGTACGATCGAGTGTACAGTGAA
MT520261.1 Severe acute respiratory syndrome coronavirus 2 isolate SARS-CoV-2/human/USA/MA_MGH_00563/202	TTCACCGAGGCCACGCGGAGTACGATCGAGTGTACAGTGAA
MT520262.1 Severe acute respiratory syndrome coronavirus 2 isolate SARS-CoV-2/human/USA/MA_MGH_00690/202	TTCACCGAGGCCACGCGGAGTACGATCGAGTGTACAGTGAA
MT520263.1 Severe acute respiratory syndrome coronavirus 2 isolate SARS-CoV-2/human/USA/MA_MGH_00229/202	TTCACCGAGGCCACGCGGAGTACGATCGAGTGTACAGTGAA
MT520264.1 Severe acute respiratory syndrome coronavirus 2 isolate SARS-CoV-2/human/USA/MA_MGH_00633/202	TTCACCGAGGCCACGCGGAGTACGATCGAGTGTACAGTGAA
MT520265.1 Severe acute respiratory syndrome coronavirus 2 isolate SARS-CoV-2/human/USA/MA_MGH_00669/202	TTCACCGAGGCCACGCGGAGTACGATCGAGTGTACAGTGAA
MT520266.1 Severe acute respiratory syndrome coronavirus 2 isolate SARS-CoV-2/human/USA/MA_MGH_00695/202	TTCACCGAGGCCACGCGGAGTACGATCGAGTGTACAGTGAA
MT520267.1 Severe acute respiratory syndrome coronavirus 2 isolate SARS-CoV-2/human/USA/MA_MGH_00579/202	TTCACCGAGGCCACGCGGAGTACGATCGAGTGTACAGTGAA
MT520268.1 Severe acute respiratory syndrome coronavirus 2 isolate SARS-CoV-2/human/USA/MA_MGH_00286/202	TTCACCGAGGCCACGCGGAGTACGATCGAGTGTACAGTGAA
MT520270.1 Severe acute respiratory syndrome coronavirus 2 isolate SARS-CoV-2/human/USA/MA_MGH_00281/202	TTCACCGAGGCCACGCGGAGTACGATCGAGTGTACAGTGAA
MT520271.1 Severe acute respiratory syndrome coronavirus 2 isolate SARS-CoV-2/human/USA/MA_MGH_00290/202	TTCACCGAGGCCACGCGGAGTACGATCGAGTGTACAGTGAA
MT520272.1 Severe acute respiratory syndrome coronavirus 2 isolate SARS-CoV-2/human/USA/MA_MGH_00653/202	TTCACCGAGGCCACGCGGAGTACGATCGAGTGTACAGTGAA
MT520273.1 Severe acute respiratory syndrome coronavirus 2 isolate SARS-CoV-2/human/USA/MA_MGH_00445/202	TTCACCGAGGCCACGCGGAGTACGATCGAGTGTACAGTGAA
MT520274.1 Severe acute respiratory syndrome coronavirus 2 isolate SARS-CoV-2/human/USA/MA_MGH_00162/202	TTCACCGAGGCCACGCGGAGTACGATCGAGTGTACAGTGAA
MT520275.1 Severe acute respiratory syndrome coronavirus 2 isolate SARS-CoV-2/human/USA/MA_MGH_00135/202	TTCACCGAGGCCACGCGGAGTACGATCGAGTGTACAGTGAA
MT520276.1 Severe acute respiratory syndrome coronavirus 2 isolate SARS-CoV-2/human/USA/MA_MGH_00473/202	TTCACCGAGGCCACGCGGAGTACGATCGAGTGTACAGTGAA
MT520277.1 Severe acute respiratory syndrome coronavirus 2 isolate SARS-CoV-2/human/USA/MA_MGH_00076/202	TTCACCGAGGCCACGCGGAGTACGATCGAGTGTACAGTGAA

MT520278.1 Severe acute respiratory syndrome coronavirus 2 isolate SARS-CoV-2/human/USA/MA_MGH_00195/20	TTCACCGAGGCCACGCGGAGTACGATCGAGTGTACAGTGAA
MT520279.1 Severe acute respiratory syndrome coronavirus 2 isolate SARS-CoV-2/human/USA/MA_MGH_00486/20	TTCACCGAGGCCACGCGGAGTACGATCGAGTGTACAGTGAA
MT520280.1 Severe acute respiratory syndrome coronavirus 2 isolate SARS-CoV-2/human/USA/MA_MGH_00614/20	TTCACCGAGGCCACGCGGAGTACGATCGAGTGTACAGTGAA
MT520281.1 Severe acute respiratory syndrome coronavirus 2 isolate SARS-CoV-2/human/USA/MA_MGH_00032/20	TTCACCGAGGCCACGCGGAGTACGATCGAGTGTACAGTGAA
MT520282.1 Severe acute respiratory syndrome coronavirus 2 isolate SARS-CoV-2/human/USA/MA_MGH_00442/20	TTCACCGAGGCCACGCGGAGTACGATCGAGTGTACAGTGAA
MT520283.1 Severe acute respiratory syndrome coronavirus 2 isolate SARS-CoV-2/human/USA/MA_MGH_00477/20	TTCACCGAGGCCACGCGGAGTACGATCGAGTGTACAGTGAA
MT520284.1 Severe acute respiratory syndrome coronavirus 2 isolate SARS-CoV-2/human/USA/MA_MGH_00177/20	TTCACCGAGGCCACGCGGAGTACGATCGAGTGTACAGTGAA
MT520285.1 Severe acute respiratory syndrome coronavirus 2 isolate SARS-CoV-2/human/USA/MA_MGH_00280/20	TTCACCGAGGCCACGCGGAGTACGATCGAGTGTACAGTGAA
MT520286.1 Severe acute respiratory syndrome coronavirus 2 isolate SARS-CoV-2/human/USA/MA_MGH_00575/20	TTCACCGAGGCCACGCGGAGTACGATCGAGTGTACAGTGAA
MT520287.1 Severe acute respiratory syndrome coronavirus 2 isolate SARS-CoV-2/human/USA/MA_MGH_00427/20	TTCACCGAGGCCACGCGGAGTACGATCGAGTGTACAGTGAA
MT520288.1 Severe acute respiratory syndrome coronavirus 2 isolate SARS-CoV-2/human/USA/MA_MGH_00247/20	TTCACCGAGGCCACGCGGAGTACGATCGAGTGTACAGTGAA
MT520289.1 Severe acute respiratory syndrome coronavirus 2 isolate SARS-CoV-2/human/USA/MA_MGH_00063/20	TTCACCGAGGCCACGCGGAGTACGATCGAGTGTACAGTGAA
MT520290.1 Severe acute respiratory syndrome coronavirus 2 isolate SARS-CoV-2/human/USA/MA_MGH_00693/20	TTCACCGAGGCCACGCGGAGTACGATCGAGTGTACAGTGAA
MT520291.1 Severe acute respiratory syndrome coronavirus 2 isolate SARS-CoV-2/human/USA/MA_MGH_00541/20	TTCACCGAGGCCACGCGGAGTACGATCGAGTGTACAGTGAA
MT520292.1 Severe acute respiratory syndrome coronavirus 2 isolate SARS-CoV-2/human/USA/MA_MGH_00659/20	TTCACCGAGGCCACGCGGAGTACGATCGAGTGTACAGTGAA
MT520293.1 Severe acute respiratory syndrome coronavirus 2 isolate SARS-CoV-2/human/USA/MA_MGH_00044/20	TTCACCGAGGCCACGCGGAGTACGATCGAGTGTACAGTGAA
MT520294.1 Severe acute respiratory syndrome coronavirus 2 isolate SARS-CoV-2/human/USA/MA_MGH_00096/20	TTCACCGAGGCCACGCGGAGTACGATCGAGTGTACAGTGAA
MT520295.1 Severe acute respiratory syndrome coronavirus 2 isolate SARS-CoV-2/human/USA/MA_MGH_00460/20	TTCACCGAGGCCACGCGGAGTACGATCGAGTGTACAGTGAA
MT520296.1 Severe acute respiratory syndrome coronavirus 2 isolate SARS-CoV-2/human/USA/MA_MGH_00187/20	TTCACCGAGGCCACGCGGAGTACGATCGAGTGTACAGTGAA
MT520298.1 Severe acute respiratory syndrome coronavirus 2 isolate SARS-CoV-2/human/USA/MA_MGH_00454/20	TTCACCGAGGCCACGCGGAGTACGATCGAGTGTACAGTGAA
MT520299.1 Severe acute respiratory syndrome coronavirus 2 isolate SARS-CoV-2/human/USA/MA_MGH_00521/20	TTCACCGAGGCCACGCGGAGTACGATCGAGTGTACAGTGAA
MT520300.1 Severe acute respiratory syndrome coronavirus 2 isolate SARS-CoV-2/human/USA/MA_MGH_00475/20	TTCACCGAGGCCACGCGGAGTACGATCGAGTGTACAGTGAA
MT520301.1 Severe acute respiratory syndrome coronavirus 2 isolate SARS-CoV-2/human/USA/MA_MGH_00469/20	TTCACCGAGGCCACGCGGAGTACGATCGAGTGTACAGTGAA
MT520302.1 Severe acute respiratory syndrome coronavirus 2 isolate SARS-CoV-2/human/USA/MA_MGH_00480/20	TTCACCGAGGCCACGCGGAGTACGATCGAGTGTACAGTGAA
MT520304.1 Severe acute respiratory syndrome coronavirus 2 isolate SARS-CoV-2/human/USA/MA_MGH_00185/20	TTCACCGAGGCCACGCGGAGTACGATCGAGTGTACAGTGAA
MT520305.1 Severe acute respiratory syndrome coronavirus 2 isolate SARS-CoV-2/human/USA/MA_MGH_00474/20	TTCACCGAGGCCACGCGGAGTACGATCGAGTGTACAGTGAA
MT520306.1 Severe acute respiratory syndrome coronavirus 2 isolate SARS-CoV-2/human/USA/MA_MGH_00680/20	TTCACCGAGGCCACGCGGAGTACGATCGAGTGTACAGTGAA
MT520307.1 Severe acute respiratory syndrome coronavirus 2 isolate SARS-CoV-2/human/USA/MA_MGH_00555/20	TTCACCGAGGCCACGCGGAGTACGATCGAGTGTACAGTGAA
MT520308.1 Severe acute respiratory syndrome coronavirus 2 isolate SARS-CoV-2/human/USA/MA_MGH_00704/20	TTCACCGAGGCCACGCGGAGTACGATCGAGTGTACAGTGAA
MT520309.1 Severe acute respiratory syndrome coronavirus 2 isolate SARS-CoV-2/human/USA/MA_MGH_00287/20	TTCACCGAGGCCACGCGGAGTACGATCGAGTGTACAGTGAA
MT520310.1 Severe acute respiratory syndrome coronavirus 2 isolate SARS-CoV-2/human/USA/MA_MGH_00431/20	TTCACCGAGGCCACGCGGAGTACGATCGAGTGTACAGTGAA
MT520311.1 Severe acute respiratory syndrome coronavirus 2 isolate SARS-CoV-2/human/USA/MA_MGH_00168/20	TTCACCGAGGCCACGCGGAGTACGATCGAGTGTACAGTGAA
MT520312.1 Severe acute respiratory syndrome coronavirus 2 isolate SARS-CoV-2/human/USA/MA_MGH_00239/20	TTCACCGAGGCCACGCGGAGTACGATCGAGTGTACAGTGAA

MT520313.1 Severe acute respiratory syndrome coronavirus 2 isolate SARS-CoV-2/human/USA/MA_MGH_00151/202	TTCACCGAGGCCACGCGGAGTACGATCGAGTGTACAGTGAA
MT520314.1 Severe acute respiratory syndrome coronavirus 2 isolate SARS-CoV-2/human/USA/MA_MGH_00437/202	TTCACCGAGGCCACGCGGAGTACGATCGAGTGTACAGTGAA
MT520315.1 Severe acute respiratory syndrome coronavirus 2 isolate SARS-CoV-2/human/USA/MA_MGH_00037/202	TTCACCGAGGCCACGCGGAGTACGATCGAGTGTACAGTGAA
MT520316.1 Severe acute respiratory syndrome coronavirus 2 isolate SARS-CoV-2/human/USA/MA_MGH_00415/202	TTCACCGAGGCCACGCGGAGTACGATCGAGTGTACAGTGAA
MT520317.1 Severe acute respiratory syndrome coronavirus 2 isolate SARS-CoV-2/human/USA/MA_MGH_00206/202	TTCACCGAGGCCACGCGGAGTACGATCGAGTGTACAGTGAA
MT520318.1 Severe acute respiratory syndrome coronavirus 2 isolate SARS-CoV-2/human/USA/MA_MGH_00270/202	TTCACCGAGGCCACGCGGAGTACGATCGAGTGTACAGTGAA
MT520319.1 Severe acute respiratory syndrome coronavirus 2 isolate SARS-CoV-2/human/USA/MA_MGH_00463/202	TTCACCGAGGCCACGCGGAGTACGATCGAGTGTACAGTGAA
MT520320.1 Severe acute respiratory syndrome coronavirus 2 isolate SARS-CoV-2/human/USA/MA_MGH_00420/202	TTCACCGAGGCCACGCGGAGTACGATCGAGTGTACAGTGAA
MT520321.1 Severe acute respiratory syndrome coronavirus 2 isolate SARS-CoV-2/human/USA/MA_MGH_00717/202	TTCACCGAGGCCACGCGGAGTACGATCGAGTGTACAGTGAA
MT520323.1 Severe acute respiratory syndrome coronavirus 2 isolate SARS-CoV-2/human/USA/MA_MGH_00660/202	TTCACCGAGGCCACGCGGAGTACGATCGAGTGTACAGTGAA
MT520324.1 Severe acute respiratory syndrome coronavirus 2 isolate SARS-CoV-2/human/USA/MA_MGH_00273/202	TTCACCGAGGCCACGCGGAGTACGATCGAGTGTACAGTGAA
MT520325.1 Severe acute respiratory syndrome coronavirus 2 isolate SARS-CoV-2/human/USA/MA_MGH_00438/202	TTCACCGAGGCCACGCGGAGTACGATCGAGTGTACAGTGAA
MT520327.1 Severe acute respiratory syndrome coronavirus 2 isolate SARS-CoV-2/human/USA/MA_MGH_00435/202	TTCACCGAGGCCACGCGGAGTACGATCGAGTGTACAGTGAA
MT520328.1 Severe acute respiratory syndrome coronavirus 2 isolate SARS-CoV-2/human/USA/MA_MGH_00514/202	TTCACCGAGGCCACGCGGAGTACGATCGAGTGTACAGTGAA
MT520329.1 Severe acute respiratory syndrome coronavirus 2 isolate SARS-CoV-2/human/USA/MA_MGH_00650/202	TTCACCGAGGCCACGCGGAGTACGATCGAGTGTACAGTGAA
MT520330.1 Severe acute respiratory syndrome coronavirus 2 isolate SARS-CoV-2/human/USA/MA_MGH_00299/202	TTCACCGAGGCCACGCGGAGTACGATCGAGTGTACAGTGAA
MT520331.1 Severe acute respiratory syndrome coronavirus 2 isolate SARS-CoV-2/human/USA/MA_MGH_00657/202	TTCACCGAGGCCACGCGGAGTACGATCGAGTGTACAGTGAA
MT520332.1 Severe acute respiratory syndrome coronavirus 2 isolate SARS-CoV-2/human/USA/MA_MGH_00688/202	TTCACCGAGGCCACGCGGAGTACGATCGAGTGTACAGTGAA
MT520333.1 Severe acute respiratory syndrome coronavirus 2 isolate SARS-CoV-2/human/USA/MA_MGH_00501/202	TTCACCGAGGCCACGCGGAGTACGATCGAGTGTACAGTGAA
MT520335.1 Severe acute respiratory syndrome coronavirus 2 isolate SARS-CoV-2/human/USA/MA_MGH_00306/202	TTCACCGAGGCCACGCGGAGTACGATCGAGTGTACAGTGAA
MT520336.1 Severe acute respiratory syndrome coronavirus 2 isolate SARS-CoV-2/human/USA/MA_MGH_00156/202	TTCACCGAGGCCACGCGGAGTACGATCGAGTGTACAGTGAA
MT520337.1 Severe acute respiratory syndrome coronavirus 2 isolate SARS-CoV-2/human/USA/MA_MGH_00523/202	TTCACCGAGGCCACGCGGAGTACGATCGAGTGTACAGTGAA
MT520338.1 Severe acute respiratory syndrome coronavirus 2 isolate SARS-CoV-2/human/USA/MA_MGH_00190/202	TTCACCGAGGCCACGCGGAGTACGATCGAGTGTACAGTGAA
MT520339.1 Severe acute respiratory syndrome coronavirus 2 isolate SARS-CoV-2/human/USA/MA_MGH_00049/202	TTCACCGAGGCCACGCGGAGTACGATCGAGTGTACAGTGAA
MT520340.1 Severe acute respiratory syndrome coronavirus 2 isolate SARS-CoV-2/human/USA/MA_MGH_00298/202	TTCACCGAGGCCACGCGGAGTACGATCGAGTGTACAGTGAA
MT520341.1 Severe acute respiratory syndrome coronavirus 2 isolate SARS-CoV-2/human/USA/MA_MGH_00421/202	TTCACCGAGGCCACGCGGAGTACGATCGAGTGTACAGTGAA
MT520342.1 Severe acute respiratory syndrome coronavirus 2 isolate SARS-CoV-2/human/USA/MA_MGH_00681/202	TTCACCGAGGCCACGCGGAGTACGATCGAGTGTACAGTGAA
MT520343.1 Severe acute respiratory syndrome coronavirus 2 isolate SARS-CoV-2/human/USA/MA_MGH_00699/202	TTCACCGAGGCCACGCGGAGTACGATCGAGTGTACAGTGAA
MT520344.1 Severe acute respiratory syndrome coronavirus 2 isolate SARS-CoV-2/human/USA/MA_MGH_00093/202	TTCACCGAGGCCACGCGGAGTACGATCGAGTGTACAGTGAA
MT520345.1 Severe acute respiratory syndrome coronavirus 2 isolate SARS-CoV-2/human/USA/MA_MGH_00604/202	TTCACCGAGGCCACGCGGAGTACGATCGAGTGTACAGTGAA
MT520346.1 Severe acute respiratory syndrome coronavirus 2 isolate SARS-CoV-2/human/USA/MA_MGH_00014/202	TTCACCGAGGCCACGCGGAGTACGATCGAGTGTACAGTGAA
MT520347.1 Severe acute respiratory syndrome coronavirus 2 isolate SARS-CoV-2/human/USA/MA_MGH_00458/202	TTCACCGAGGCCACGCGGAGTACGATCGAGTGTACAGTGAA
MT520348.1 Severe acute respiratory syndrome coronavirus 2 isolate SARS-CoV-2/human/USA/MA_MGH_00580/202	TTCACCGAGGCCACGCGGAGTACGATCGAGTGTACAGTGAA

MT520349.1 Severe acute respiratory syndrome coronavirus 2 isolate SARS-CoV-2/human/USA/MA_MGH_00294/20[illegible]	TTCACCGAGGCCACGCGGAGTACGATCGAGTGTACAGTGAA
MT520350.1 Severe acute respiratory syndrome coronavirus 2 isolate SARS-CoV-2/human/USA/MA_MGH_00538/20[illegible]	TTCACCGAGGCCACGCGGAGTACGATCGAGTGTACAGTGAA
MT520351.1 Severe acute respiratory syndrome coronavirus 2 isolate SARS-CoV-2/human/USA/MA_MGH_00583/20[illegible]	TTCACCGAGGCCACGCGGAGTACGATCGAGTGTACAGTGAA
MT520352.1 Severe acute respiratory syndrome coronavirus 2 isolate SARS-CoV-2/human/USA/MA_MGH_00716/20[illegible]	TTCACCGAGGCCACGCGGAGTACGATCGAGTGTACAGTGAA
MT520353.1 Severe acute respiratory syndrome coronavirus 2 isolate SARS-CoV-2/human/USA/MA_MGH_00715/20[illegible]	TTCACCGAGGCCACGCGGAGTACGATCGAGTGTACAGTGAA
MT520354.1 Severe acute respiratory syndrome coronavirus 2 isolate SARS-CoV-2/human/USA/MA_MGH_00597/20[illegible]	TTCACCGAGGCCACGCGGAGTACGATCGAGTGTACAGTGAA
MT520355.1 Severe acute respiratory syndrome coronavirus 2 isolate SARS-CoV-2/human/USA/MA_MGH_00209/20[illegible]	TTCACCGAGGCCACGCGGAGTACGATCGAGTGTACAGTGAA
MT520356.1 Severe acute respiratory syndrome coronavirus 2 isolate SARS-CoV-2/human/USA/MA_MGH_00094/20[illegible]	TTCACCGAGGCCACGCGGAGTACGATCGAGTGTACAGTGAA
MT520357.1 Severe acute respiratory syndrome coronavirus 2 isolate SARS-CoV-2/human/USA/MA_MGH_00645/20[illegible]	TTCACCGAGGCCACGCGGAGTACGATCGAGTGTACAGTGAA
MT520358.1 Severe acute respiratory syndrome coronavirus 2 isolate SARS-CoV-2/human/USA/MA_MGH_00170/20[illegible]	TTCACCGAGGCCACGCGGAGTACGATCGAGTGTACAGTGAA
MT520359.1 Severe acute respiratory syndrome coronavirus 2 isolate SARS-CoV-2/human/USA/MA_MGH_00453/20[illegible]	TTCACCGAGGCCACGCGGAGTACGATCGAGTGTACAGTGAA
MT520360.1 Severe acute respiratory syndrome coronavirus 2 isolate SARS-CoV-2/human/USA/MA_MGH_00566/20[illegible]	TTCACCGAGGCCACGCGGAGTACGATCGAGTGTACAGTGAA
MT520361.1 Severe acute respiratory syndrome coronavirus 2 isolate SARS-CoV-2/human/USA/MA_MGH_00484/20[illegible]	TTCACCGAGGCCACGCGGAGTACGATCGAGTGTACAGTGAA
MT520363.1 Severe acute respiratory syndrome coronavirus 2 isolate SARS-CoV-2/human/USA/MA_MGH_00203/20[illegible]	TTCACCGAGGCCACGCGGAGTACGATCGAGTGTACAGTGAA
MT520364.1 Severe acute respiratory syndrome coronavirus 2 isolate SARS-CoV-2/human/USA/MA_MGH_00622/20[illegible]	TTCACCGAGGCCACGCGGAGTACGATCGAGTGTACAGTGAA
MT520365.1 Severe acute respiratory syndrome coronavirus 2 isolate SARS-CoV-2/human/USA/MA_MGH_00166/20[illegible]	TTCACCGAGGCCACGCGGAGTACGATCGAGTGTACAGTGAA
MT520366.1 Severe acute respiratory syndrome coronavirus 2 isolate SARS-CoV-2/human/USA/MA_MGH_00048/20[illegible]	TTCACCGAGGCCACGCGGAGTACGATCGAGTGTACAGTGAA
MT520367.1 Severe acute respiratory syndrome coronavirus 2 isolate SARS-CoV-2/human/USA/MA_MGH_00052/20[illegible]	TTCACCGAGGCCACGCGGAGTACGATCGAGTGTACAGTGAA
MT520368.1 Severe acute respiratory syndrome coronavirus 2 isolate SARS-CoV-2/human/USA/MA_MGH_00705/20[illegible]	TTCACCGAGGCCACGCGGAGTACGATCGAGTGTACAGTGAA
MT520369.1 Severe acute respiratory syndrome coronavirus 2 isolate SARS-CoV-2/human/USA/MA_MGH_00265/20[illegible]	TTCACCGAGGCCACGCGGAGTACGATCGAGTGTACAGTGAA
MT520370.1 Severe acute respiratory syndrome coronavirus 2 isolate SARS-CoV-2/human/USA/MA_MGH_00595/20[illegible]	TTCACCGAGGCCACGCGGAGTACGATCGAGTGTACAGTGAA
MT520371.1 Severe acute respiratory syndrome coronavirus 2 isolate SARS-CoV-2/human/USA/MA_MGH_00668/20[illegible]	TTCACCGAGGCCACGCGGAGTACGATCGAGTGTACAGTGAA
MT520372.1 Severe acute respiratory syndrome coronavirus 2 isolate SARS-CoV-2/human/USA/MA_MGH_00064/20[illegible]	TTCACCGAGGCCACGCGGAGTACGATCGAGTGTACAGTGAA
MT520373.1 Severe acute respiratory syndrome coronavirus 2 isolate SARS-CoV-2/human/USA/MA_MGH_00649/20[illegible]	TTCACCGAGGCCACGCGGAGTACGATCGAGTGTACAGTGAA
MT520374.1 Severe acute respiratory syndrome coronavirus 2 isolate SARS-CoV-2/human/USA/MA_MGH_00230/20[illegible]	TTCACCGAGGCCACGCGGAGTACGATCGAGTGTACAGTGAA
MT520375.1 Severe acute respiratory syndrome coronavirus 2 isolate SARS-CoV-2/human/USA/MA_MGH_00419/20[illegible]	TTCACCGAGGCCACGCGGAGTACGATCGAGTGTACAGTGAA
MT520377.1 Severe acute respiratory syndrome coronavirus 2 isolate SARS-CoV-2/human/USA/MA_MGH_00017/20[illegible]	TTCACCGAGGCCACGCGGAGTACGATCGAGTGTACAGTGAA
MT520378.1 Severe acute respiratory syndrome coronavirus 2 isolate SARS-CoV-2/human/USA/MA_MGH_00085/20[illegible]	TTCACCGAGGCCACGCGGAGTACGATCGAGTGTACAGTGAA
MT520379.1 Severe acute respiratory syndrome coronavirus 2 isolate SARS-CoV-2/human/USA/MA_MGH_00192/20[illegible]	TTCACCGAGGCCACGCGGAGTACGATCGAGTGTACAGTGAA
MT520380.1 Severe acute respiratory syndrome coronavirus 2 isolate SARS-CoV-2/human/USA/MA_MGH_00248/20[illegible]	TTCACCGAGGCCACGCGGAGTACGATCGAGTGTACAGTGAA
MT520381.1 Severe acute respiratory syndrome coronavirus 2 isolate SARS-CoV-2/human/USA/MA_MGH_00489/20[illegible]	TTCACCGAGGCCACGCGGAGTACGATCGAGTGTACAGTGAA
MT520382.1 Severe acute respiratory syndrome coronavirus 2 isolate SARS-CoV-2/human/USA/MA_MGH_00664/20[illegible]	TTCACCGAGGCCACGCGGAGTACGATCGAGTGTACAGTGAA
MT520383.1 Severe acute respiratory syndrome coronavirus 2 isolate SARS-CoV-2/human/USA/MA_MGH_00447/20[illegible]	TTCACCGAGGCCACGCGGAGTACGATCGAGTGTACAGTGAA

MT520385.1 Severe acute respiratory syndrome coronavirus 2 isolate SARS-CoV-2/human/USA/MA_MGH_00062/202	TTCACCGAGGCCACGCGGAGTACGATCGAGTGTACAGTGAA
MT520386.1 Severe acute respiratory syndrome coronavirus 2 isolate SARS-CoV-2/human/USA/MA_MGH_00003/202	TTCACCGAGGCCACGCGGAGTACGATCGAGTGTACAGTGAA
MT520387.1 Severe acute respiratory syndrome coronavirus 2 isolate SARS-CoV-2/human/USA/MA_MGH_00571/202	TTCACCGAGGCCACGCGGAGTACGATCGAGTGTACAGTGAA
MT520388.1 Severe acute respiratory syndrome coronavirus 2 isolate SARS-CoV-2/human/USA/MA_MGH_00425/202	TTCACCGAGGCCACGCGGAGTACGATCGAGTGTACAGTGAA
MT520389.1 Severe acute respiratory syndrome coronavirus 2 isolate SARS-CoV-2/human/USA/MA_MGH_00598/202	TTCACCGAGGCCACGCGGAGTACGATCGAGTGTACAGTGAA
MT520390.1 Severe acute respiratory syndrome coronavirus 2 isolate SARS-CoV-2/human/USA/MA_MGH_00718/202	TTCACCGAGGCCACGCGGAGTACGATCGAGTGTACAGTGAA
MT520391.1 Severe acute respiratory syndrome coronavirus 2 isolate SARS-CoV-2/human/USA/MA_MGH_00134/202	TTCACCGAGGCCACGCGGAGTACGATCGAGTGTACAGTGAA
MT520392.1 Severe acute respiratory syndrome coronavirus 2 isolate SARS-CoV-2/human/USA/MA_MGH_00592/202	TTCACCGAGGCCACGCGGAGTACGATCGAGTGTACAGTGAA
MT520394.1 Severe acute respiratory syndrome coronavirus 2 isolate SARS-CoV-2/human/USA/MA_MGH_00015/202	TTCACCGAGGCCACGCGGAGTACGATCGAGTGTACAGTGAA
MT520395.1 Severe acute respiratory syndrome coronavirus 2 isolate SARS-CoV-2/human/USA/MA_MGH_00019/202	TTCACCGAGGCCACGCGGAGTACGATCGAGTGTACAGTGAA
MT520396.1 Severe acute respiratory syndrome coronavirus 2 isolate SARS-CoV-2/human/USA/MA_MGH_00443/202	TTCACCGAGGCCACGCGGAGTACGATCGAGTGTACAGTGAA
MT520397.1 Severe acute respiratory syndrome coronavirus 2 isolate SARS-CoV-2/human/USA/MA_MGH_00214/202	TTCACCGAGGCCACGCGGAGTACGATCGAGTGTACAGTGAA
MT520398.1 Severe acute respiratory syndrome coronavirus 2 isolate SARS-CoV-2/human/USA/MA_MGH_00721/202	TTCACCGAGGCCACGCGGAGTACGATCGAGTGTACAGTGAA
MT520399.1 Severe acute respiratory syndrome coronavirus 2 isolate SARS-CoV-2/human/USA/MA_MGH_00024/202	TTCACCGAGGCCACGCGGAGTACGATCGAGTGTACAGTGAA
MT520400.1 Severe acute respiratory syndrome coronavirus 2 isolate SARS-CoV-2/human/USA/MA_MGH_00262/202	TTCACCGAGGCCACGCGGAGTACGATCGAGTGTACAGTGAA
MT520401.1 Severe acute respiratory syndrome coronavirus 2 isolate SARS-CoV-2/human/USA/MA_MGH_00097/202	TTCACCGAGGCCACGCGGAGTACGATCGAGTGTACAGTGAA
MT520402.1 Severe acute respiratory syndrome coronavirus 2 isolate SARS-CoV-2/human/USA/MA_MGH_00639/202	TTCACCGAGGCCACGCGGAGTACGATCGAGTGTACAGTGAA
MT520403.1 Severe acute respiratory syndrome coronavirus 2 isolate SARS-CoV-2/human/USA/MA_MGH_00665/202	TTCACCGAGGCCACGCGGAGTACGATCGAGTGTACAGTGAA
MT520404.1 Severe acute respiratory syndrome coronavirus 2 isolate SARS-CoV-2/human/USA/MA_MGH_00188/202	TTCACCGAGGCCACGCGGAGTACGATCGAGTGTACAGTGAA
MT520405.1 Severe acute respiratory syndrome coronavirus 2 isolate SARS-CoV-2/human/USA/MA_MGH_00654/202	TTCACCGAGGCCACGCGGAGTACGATCGAGTGTACAGTGAA
MT520406.1 Severe acute respiratory syndrome coronavirus 2 isolate SARS-CoV-2/human/USA/MA_MGH_00148/202	TTCACCGAGGCCACGCGGAGTACGATCGAGTGTACAGTGAA
MT520407.1 Severe acute respiratory syndrome coronavirus 2 isolate SARS-CoV-2/human/USA/MA_MGH_00263/202	TTCACCGAGGCCACGCGGAGTACGATCGAGTGTACAGTGAA
MT520408.1 Severe acute respiratory syndrome coronavirus 2 isolate SARS-CoV-2/human/USA/MA_MGH_00428/202	TTCACCGAGGCCACGCGGAGTACGATCGAGTGTACAGTGAA
MT520409.1 Severe acute respiratory syndrome coronavirus 2 isolate SARS-CoV-2/human/USA/MA_MGH_00039/202	TTCACCGAGGCCACGCGGAGTACGATCGAGTGTACAGTGAA
MT520410.1 Severe acute respiratory syndrome coronavirus 2 isolate SARS-CoV-2/human/USA/MA_MGH_00056/202	TTCACCGAGGCCACGCGGAGTACGATCGAGTGTACAGTGAA
MT520411.1 Severe acute respiratory syndrome coronavirus 2 isolate SARS-CoV-2/human/USA/MA_MGH_00698/202	TTCACCGAGGCCACGCGGAGTACGATCGAGTGTACAGTGAA
MT520412.1 Severe acute respiratory syndrome coronavirus 2 isolate SARS-CoV-2/human/USA/MA_MGH_00582/202	TTCACCGAGGCCACGCGGAGTACGATCGAGTGTACAGTGAA
MT520413.1 Severe acute respiratory syndrome coronavirus 2 isolate SARS-CoV-2/human/USA/MA_MGH_00551/202	TTCACCGAGGCCACGCGGAGTACGATCGAGTGTACAGTGAA
MT520414.1 Severe acute respiratory syndrome coronavirus 2 isolate SARS-CoV-2/human/USA/MA_MGH_00155/202	TTCACCGAGGCCACGCGGAGTACGATCGAGTGTACAGTGAA
MT520415.1 Severe acute respiratory syndrome coronavirus 2 isolate SARS-CoV-2/human/USA/MA_MGH_00615/202	TTCACCGAGGCCACGCGGAGTACGATCGAGTGTACAGTGAA
MT520416.1 Severe acute respiratory syndrome coronavirus 2 isolate SARS-CoV-2/human/USA/MA_MGH_00673/202	TTCACCGAGGCCACGCGGAGTACGATCGAGTGTACAGTGAA
MT520417.1 Severe acute respiratory syndrome coronavirus 2 isolate SARS-CoV-2/human/USA/MA_MGH_00296/202	TTCACCGAGGCCACGCGGAGTACGATCGAGTGTACAGTGAA
MT520418.1 Severe acute respiratory syndrome coronavirus 2 isolate SARS-CoV-2/human/USA/MA_MGH_00492/202	TTCACCGAGGCCACGCGGAGTACGATCGAGTGTACAGTGAA

MT520419.1 Severe acute respiratory syndrome coronavirus 2 isolate SARS-CoV-2/human/USA/MA_MGH_00528/202	TTCACCGAGGCCACGCGGAGTACGATCGAGTGTACAGTGAA
MT520420.1 Severe acute respiratory syndrome coronavirus 2 isolate SARS-CoV-2/human/USA/MA_MGH_00231/202	TTCACCGAGGCCACGCGGAGTACGATCGAGTGTACAGTGAA
MT520421.1 Severe acute respiratory syndrome coronavirus 2 isolate SARS-CoV-2/human/USA/MA_MGH_00243/202	TTCACCGAGGCCACGCGGAGTACGATCGAGTGTACAGTGAA
MT520422.1 Severe acute respiratory syndrome coronavirus 2 isolate SARS-CoV-2/human/USA/MA_MGH_00516/202	TTCACCGAGGCCACGCGGAGTACGATCGAGTGTACAGTGAA
MT520424.1 Severe acute respiratory syndrome coronavirus 2 isolate SARS-CoV-2/human/USA/MA_MGH_00553/202	TTCACCGAGGCCACGCGGAGTACGATCGAGTGTACAGTGAA
MT520426.1 Severe acute respiratory syndrome coronavirus 2 isolate SARS-CoV-2/human/USA/MA_MGH_00318/202	TTCACCGAGGCCACGCGGAGTACGATCGAGTGTACAGTGAA
MT520427.1 Severe acute respiratory syndrome coronavirus 2 isolate SARS-CoV-2/human/USA/MA_MGH_00446/202	TTCACCGAGGCCACGCGGAGTACGATCGAGTGTACAGTGAA
MT520428.1 Severe acute respiratory syndrome coronavirus 2 isolate SARS-CoV-2/human/USA/MA_MGH_00416/202	TTCACCGAGGCCACGCGGAGTACGATCGAGTGTACAGTGAA
MT520429.1 Severe acute respiratory syndrome coronavirus 2 isolate SARS-CoV-2/human/USA/MA_MGH_00444/202	TTCACCGAGGCCACGCGGAGTACGATCGAGTGTACAGTGAA
MT520430.1 Severe acute respiratory syndrome coronavirus 2 isolate SARS-CoV-2/human/USA/MA_MGH_00153/202	TTCACCGAGGCCACGCGGAGTACGATCGAGTGTACAGTGAA
MT520431.1 Severe acute respiratory syndrome coronavirus 2 isolate SARS-CoV-2/human/USA/MA_MGH_00670/202	TTCACCGAGGCCACGCGGAGTACGATCGAGTGTACAGTGAA
MT520432.1 Severe acute respiratory syndrome coronavirus 2 isolate SARS-CoV-2/human/USA/MA_MGH_00703/202	TTCACCGAGGCCACGCGGAGTACGATCGAGTGTACAGTGAA
MT520433.1 Severe acute respiratory syndrome coronavirus 2 isolate SARS-CoV-2/human/USA/MA_MGH_00701/202	TTCACCGAGGCCACGCGGAGTACGATCGAGTGTACAGTGAA
MT520434.1 Severe acute respiratory syndrome coronavirus 2 isolate SARS-CoV-2/human/USA/MA_MGH_00140/202	TTCACCGAGGCCACGCGGAGTACGATCGAGTGTACAGTGAA
MT520435.1 Severe acute respiratory syndrome coronavirus 2 isolate SARS-CoV-2/human/USA/MA_MGH_00261/202	TTCACCGAGGCCACGCGGAGTACGATCGAGTGTACAGTGAA
MT520436.1 Severe acute respiratory syndrome coronavirus 2 isolate SARS-CoV-2/human/USA/MA_MGH_00472/202	TTCACCGAGGCCACGCGGAGTACGATCGAGTGTACAGTGAA
MT520437.1 Severe acute respiratory syndrome coronavirus 2 isolate SARS-CoV-2/human/USA/MA_MGH_00183/202	TTCACCGAGGCCACGCGGAGTACGATCGAGTGTACAGTGAA
MT520439.1 Severe acute respiratory syndrome coronavirus 2 isolate SARS-CoV-2/human/USA/MA_MGH_00136/202	TTCACCGAGGCCACGCGGAGTACGATCGAGTGTACAGTGAA
MT520440.1 Severe acute respiratory syndrome coronavirus 2 isolate SARS-CoV-2/human/USA/MA_MGH_00238/202	TTCACCGAGGCCACGCGGAGTACGATCGAGTGTACAGTGAA
MT520441.1 Severe acute respiratory syndrome coronavirus 2 isolate SARS-CoV-2/human/USA/MA_MGH_00430/202	TTCACCGAGGCCACGCGGAGTACGATCGAGTGTACAGTGAA
MT520442.1 Severe acute respiratory syndrome coronavirus 2 isolate SARS-CoV-2/human/USA/MA_MGH_00167/202	TTCACCGAGGCCACGCGGAGTACGATCGAGTGTACAGTGAA
MT520443.1 Severe acute respiratory syndrome coronavirus 2 isolate SARS-CoV-2/human/USA/MA_MGH_00194/202	TTCACCGAGGCCACGCGGAGTACGATCGAGTGTACAGTGAA
MT520444.1 Severe acute respiratory syndrome coronavirus 2 isolate SARS-CoV-2/human/USA/MA_MGH_00225/202	TTCACCGAGGCCACGCGGAGTACGATCGAGTGTACAGTGAA
MT520445.1 Severe acute respiratory syndrome coronavirus 2 isolate SARS-CoV-2/human/USA/MA_MGH_00232/202	TTCACCGAGGCCACGCGGAGTACGATCGAGTGTACAGTGAA
MT520446.1 Severe acute respiratory syndrome coronavirus 2 isolate SARS-CoV-2/human/USA/MA_MGH_00418/202	TTCACCGAGGCCACGCGGAGTACGATCGAGTGTACAGTGAA
MT520447.1 Severe acute respiratory syndrome coronavirus 2 isolate SARS-CoV-2/human/USA/MA_MGH_00189/202	TTCACCGAGGCCACGCGGAGTACGATCGAGTGTACAGTGAA
MT520448.1 Severe acute respiratory syndrome coronavirus 2 isolate SARS-CoV-2/human/USA/MA_MGH_00251/202	TTCACCGAGGCCACGCGGAGTACGATCGAGTGTACAGTGAA
MT520451.1 Severe acute respiratory syndrome coronavirus 2 isolate SARS-CoV-2/human/USA/MA_MGH_00506/202	TTCACCGAGGCCACGCGGAGTACGATCGAGTGTACAGTGAA
MT520452.1 Severe acute respiratory syndrome coronavirus 2 isolate SARS-CoV-2/human/USA/MA_MGH_00663/202	TTCACCGAGGCCACGCGGAGTACGATCGAGTGTACAGTGAA
MT520453.1 Severe acute respiratory syndrome coronavirus 2 isolate SARS-CoV-2/human/USA/MA_MGH_00256/202	TTCACCGAGGCCACGCGGAGTACGATCGAGTGTACAGTGAA
MT520454.1 Severe acute respiratory syndrome coronavirus 2 isolate SARS-CoV-2/human/USA/MA_MGH_00303/202	TTCACCGAGGCCACGCGGAGTACGATCGAGTGTACAGTGAA
MT520455.1 Severe acute respiratory syndrome coronavirus 2 isolate SARS-CoV-2/human/USA/MA_MGH_00557/202	TTCACCGAGGCCACGCGGAGTACGATCGAGTGTACAGTGAA
MT520456.1 Severe acute respiratory syndrome coronavirus 2 isolate SARS-CoV-2/human/USA/MA_MGH_00532/202	TTCACCGAGGCCACGCGGAGTACGATCGAGTGTACAGTGAA

MT520457.1 Severe acute respiratory syndrome coronavirus 2 isolate SARS-CoV-2/human/USA/MA_MGH_00600/20	TTCACCGAGGCCACGCGGAGTACGATCGAGTGTACAGTGAA
MT520458.1 Severe acute respiratory syndrome coronavirus 2 isolate SARS-CoV-2/human/USA/MA_MGH_00456/20	TTCACCGAGGCCACGCGGAGTACGATCGAGTGTACAGTGAA
MT520459.1 Severe acute respiratory syndrome coronavirus 2 isolate SARS-CoV-2/human/USA/MA_MGH_00422/20	TTCACCGAGGCCACGCGGAGTACGATCGAGTGTACAGTGAA
MT520460.1 Severe acute respiratory syndrome coronavirus 2 isolate SARS-CoV-2/human/USA/MA_MGH_00468/20	TTCACCGAGGCCACGCGGAGTACGATCGAGTGTACAGTGAA
MT520461.1 Severe acute respiratory syndrome coronavirus 2 isolate SARS-CoV-2/human/USA/MA_MGH_00648/20	TTCACCGAGGCCACGCGGAGTACGATCGAGTGTACAGTGAA
MT520463.1 Severe acute respiratory syndrome coronavirus 2 isolate SARS-CoV-2/human/USA/MA_MGH_00676/20	TTCACCGAGGCCACGCGGAGTACGATCGAGTGTACAGTGAA
MT520464.1 Severe acute respiratory syndrome coronavirus 2 isolate SARS-CoV-2/human/USA/MA_MGH_00234/20	TTCACCGAGGCCACGCGGAGTACGATCGAGTGTACAGTGAA
MT520465.1 Severe acute respiratory syndrome coronavirus 2 isolate SARS-CoV-2/human/USA/MA_MGH_00465/20	TTCACCGAGGCCACGCGGAGTACGATCGAGTGTACAGTGAA
MT520466.1 Severe acute respiratory syndrome coronavirus 2 isolate SARS-CoV-2/human/USA/MA_MGH_00083/20	TTCACCGAGGCCACGCGGAGTACGATCGAGTGTACAGTGAA
MT520467.1 Severe acute respiratory syndrome coronavirus 2 isolate SARS-CoV-2/human/USA/MA_MGH_00479/20	TTCACCGAGGCCACGCGGAGTACGATCGAGTGTACAGTGAA
MT520469.1 Severe acute respiratory syndrome coronavirus 2 isolate SARS-CoV-2/human/USA/MA_MGH_00160/20	TTCACCGAGGCCACGCGGAGTACGATCGAGTGTACAGTGAA
MT520470.1 Severe acute respiratory syndrome coronavirus 2 isolate SARS-CoV-2/human/USA/MA_MGH_00434/20	TTCACCGAGGCCACGCGGAGTACGATCGAGTGTACAGTGAA
MT520471.1 Severe acute respiratory syndrome coronavirus 2 isolate SARS-CoV-2/human/USA/MA_MGH_00613/20	TTCACCGAGGCCACGCGGAGTACGATCGAGTGTACAGTGAA
MT520472.1 Severe acute respiratory syndrome coronavirus 2 isolate SARS-CoV-2/human/USA/MA_MGH_00424/20	TTCACCGAGGCCACGCGGAGTACGATCGAGTGTACAGTGAA
MT520473.1 Severe acute respiratory syndrome coronavirus 2 isolate SARS-CoV-2/human/USA/MA_MGH_00510/20	TTCACCGAGGCCACGCGGAGTACGATCGAGTGTACAGTGAA
MT520474.1 Severe acute respiratory syndrome coronavirus 2 isolate SARS-CoV-2/human/USA/MA_MGH_00576/20	TTCACCGAGGCCACGCGGAGTACGATCGAGTGTACAGTGAA
MT520475.1 Severe acute respiratory syndrome coronavirus 2 isolate SARS-CoV-2/human/USA/MA_MGH_00497/20	TTCACCGAGGCCACGCGGAGTACGATCGAGTGTACAGTGAA
MT520477.1 Severe acute respiratory syndrome coronavirus 2 isolate SARS-CoV-2/human/USA/MA_MGH_00658/20	TTCACCGAGGCCACGCGGAGTACGATCGAGTGTACAGTGAA
MT520478.1 Severe acute respiratory syndrome coronavirus 2 isolate SARS-CoV-2/human/USA/MA_MGH_00694/20	TTCACCGAGGCCACGCGGAGTACGATCGAGTGTACAGTGAA
MT520479.1 Severe acute respiratory syndrome coronavirus 2 isolate SARS-CoV-2/human/USA/MA_MGH_00257/20	TTCACCGAGGCCACGCGGAGTACGATCGAGTGTACAGTGAA
MT520480.1 Severe acute respiratory syndrome coronavirus 2 isolate SARS-CoV-2/human/USA/MA_MGH_00240/20	TTCACCGAGGCCACGCGGAGTACGATCGAGTGTACAGTGAA
MT520481.1 Severe acute respiratory syndrome coronavirus 2 isolate SARS-CoV-2/human/USA/MA_MGH_00684/20	TTCACCGAGGCCACGCGGAGTACGATCGAGTGTACAGTGAA
MT520482.1 Severe acute respiratory syndrome coronavirus 2 isolate SARS-CoV-2/human/USA/MA_MGH_00244/20	TTCACCGAGGCCACGCGGAGTACGATCGAGTGTACAGTGAA
MT520483.1 Severe acute respiratory syndrome coronavirus 2 isolate SARS-CoV-2/human/USA/MA_MGH_00282/20	TTCACCGAGGCCACGCGGAGTACGATCGAGTGTACAGTGAA
MT520484.1 Severe acute respiratory syndrome coronavirus 2 isolate SARS-CoV-2/human/USA/MA_MGH_00149/20	TTCACCGAGGCCACGCGGAGTACGATCGAGTGTACAGTGAA
MT520485.1 Severe acute respiratory syndrome coronavirus 2 isolate SARS-CoV-2/human/USA/MA_MGH_00200/20	TTCACCGAGGCCACGCGGAGTACGATCGAGTGTACAGTGAA
MT520486.1 Severe acute respiratory syndrome coronavirus 2 isolate SARS-CoV-2/human/USA/MA_MGH_00090/20	TTCACCGAGGCCACGCGGAGTACGATCGAGTGTACAGTGAA
MT520487.1 Severe acute respiratory syndrome coronavirus 2 isolate SARS-CoV-2/human/USA/MA_MGH_00637/20	TTCACCGAGGCCACGCGGAGTACGATCGAGTGTACAGTGAA
MT520488.1 Severe acute respiratory syndrome coronavirus 2 isolate SARS-CoV-2/human/USA/MA_MGH_00533/20	TTCACCGAGGCCACGCGGAGTACGATCGAGTGTACAGTGAA
MT520489.1 Severe acute respiratory syndrome coronavirus 2 isolate SARS-CoV-2/human/USA/MA_MGH_00689/20	TTCACCGAGGCCACGCGGAGTACGATCGAGTGTACAGTGAA
MT520490.1 Severe acute respiratory syndrome coronavirus 2 isolate SARS-CoV-2/human/USA/MA_MGH_00517/20	TTCACCGAGGCCACGCGGAGTACGATCGAGTGTACAGTGAA
MT520492.1 Severe acute respiratory syndrome coronavirus 2 isolate SARS-CoV-2/human/USA/MA_MGH_00164/20	TTCACCGAGGCCACGCGGAGTACGATCGAGTGTACAGTGAA
MT520493.1 Severe acute respiratory syndrome coronavirus 2 isolate SARS-CoV-2/human/USA/MA_MGH_00316/20	TTCACCGAGGCCACGCGGAGTACGATCGAGTGTACAGTGAA

MT520494.1 Severe acute respiratory syndrome coronavirus 2 isolate SARS-CoV-2/human/USA/MA_MGH_00646/202	TTCACCGAGGCCACGCGGAGTACGATCGAGTGTACAGTGAA
MT520495.1 Severe acute respiratory syndrome coronavirus 2 isolate SARS-CoV-2/human/USA/MA_MGH_00476/202	TTCACCGAGGCCACGCGGAGTACGATCGAGTGTACAGTGAA
MT520496.1 Severe acute respiratory syndrome coronavirus 2 isolate SARS-CoV-2/human/USA/MA_MGH_00036/202	TTCACCGAGGCCACGCGGAGTACGATCGAGTGTACAGTGAA
MT520497.1 Severe acute respiratory syndrome coronavirus 2 isolate SARS-CoV-2/human/USA/MA_MGH_00696/202	TTCACCGAGGCCACGCGGAGTACGATCGAGTGTACAGTGAA
MT520498.1 Severe acute respiratory syndrome coronavirus 2 isolate SARS-CoV-2/human/USA/MA_MGH_00565/202	TTCACCGAGGCCACGCGGAGTACGATCGAGTGTACAGTGAA
MT520499.1 Severe acute respiratory syndrome coronavirus 2 isolate SARS-CoV-2/human/USA/MA_MGH_00556/202	TTCACCGAGGCCACGCGGAGTACGATCGAGTGTACAGTGAA
MT520500.1 Severe acute respiratory syndrome coronavirus 2 isolate SARS-CoV-2/human/USA/MA_MGH_00211/202	TTCACCGAGGCCACGCGGAGTACGATCGAGTGTACAGTGAA
MT520501.1 Severe acute respiratory syndrome coronavirus 2 isolate SARS-CoV-2/human/USA/MA_MGH_00581/202	TTCACCGAGGCCACGCGGAGTACGATCGAGTGTACAGTGAA
MT520502.1 Severe acute respiratory syndrome coronavirus 2 isolate SARS-CoV-2/human/USA/MA_MGH_00495/202	TTCACCGAGGCCACGCGGAGTACGATCGAGTGTACAGTGAA
MT520503.1 Severe acute respiratory syndrome coronavirus 2 isolate SARS-CoV-2/human/USA/MA_MGH_00441/202	TTCACCGAGGCCACGCGGAGTACGATCGAGTGTACAGTGAA
MT520504.1 Severe acute respiratory syndrome coronavirus 2 isolate SARS-CoV-2/human/USA/MA_MGH_00252/202	TTCACCGAGGCCACGCGGAGTACGATCGAGTGTACAGTGAA
MT520505.1 Severe acute respiratory syndrome coronavirus 2 isolate SARS-CoV-2/human/USA/MA_MGH_00304/202	TTCACCGAGGCCACGCGGAGTACGATCGAGTGTACAGTGAA
MT520506.1 Severe acute respiratory syndrome coronavirus 2 isolate SARS-CoV-2/human/USA/MA_MGH_00307/202	TTCACCGAGGCCACGCGGAGTACGATCGAGTGTACAGTGAA
MT520507.1 Severe acute respiratory syndrome coronavirus 2 isolate SARS-CoV-2/human/USA/MA_MGH_00305/202	TTCACCGAGGCCACGCGGAGTACGATCGAGTGTACAGTGAA
MT520508.1 Severe acute respiratory syndrome coronavirus 2 isolate SARS-CoV-2/human/USA/MA_MGH_00147/202	TTCACCGAGGCCACGCGGAGTACGATCGAGTGTACAGTGAA
MT520509.1 Severe acute respiratory syndrome coronavirus 2 isolate SARS-CoV-2/human/USA/MA_MGH_00439/202	TTCACCGAGGCCACGCGGAGTACGATCGAGTGTACAGTGAA
MT520510.1 Severe acute respiratory syndrome coronavirus 2 isolate SARS-CoV-2/human/USA/MA_MGH_00245/202	TTCACCGAGGCCACGCGGAGTACGATCGAGTGTACAGTGAA
MT520511.1 Severe acute respiratory syndrome coronavirus 2 isolate SARS-CoV-2/human/USA/MA_MGH_00531/202	TTCACCGAGGCCACGCGGAGTACGATCGAGTGTACAGTGAA
MT520512.1 Severe acute respiratory syndrome coronavirus 2 isolate SARS-CoV-2/human/USA/MA_MGH_00202/202	TTCACCGAGGCCACGCGGAGTACGATCGAGTGTACAGTGAA
MT520515.1 Severe acute respiratory syndrome coronavirus 2 isolate SARS-CoV-2/human/USA/MA_MGH_00006/202	TTCACCGAGGCCACGCGGAGTACGATCGAGTGTACAGTGAA
MT520516.1 Severe acute respiratory syndrome coronavirus 2 isolate SARS-CoV-2/human/USA/MA_MGH_00481/202	TTCACCGAGGCCACGCGGAGTACGATCGAGTGTACAGTGAA
MT520517.1 Severe acute respiratory syndrome coronavirus 2 isolate SARS-CoV-2/human/USA/MA_MGH_00432/202	TTCACCGAGGCCACGCGGAGTACGATCGAGTGTACAGTGAA
MT520518.1 Severe acute respiratory syndrome coronavirus 2 isolate SARS-CoV-2/human/USA/MA_MGH_00635/202	TTCACCGAGGCCACGCGGAGTACGATCGAGTGTACAGTGAA
MT520519.1 Severe acute respiratory syndrome coronavirus 2 isolate SARS-CoV-2/human/USA/MA_MGH_00084/202	TTCACCGAGGCCACGCGGAGTACGATCGAGTGTACAGTGAA
MT520520.1 Severe acute respiratory syndrome coronavirus 2 isolate SARS-CoV-2/human/USA/MA_MGH_00505/202	TTCACCGAGGCCACGCGGAGTACGATCGAGTGTACAGTGAA
MT520521.1 Severe acute respiratory syndrome coronavirus 2 isolate SARS-CoV-2/human/USA/MA_MGH_00071/202	TTCACCGAGGCCACGCGGAGTACGATCGAGTGTACAGTGAA
MT520522.1 Severe acute respiratory syndrome coronavirus 2 isolate SARS-CoV-2/human/USA/MA_MGH_00152/202	TTCACCGAGGCCACGCGGAGTACGATCGAGTGTACAGTGAA
MT520523.1 Severe acute respiratory syndrome coronavirus 2 isolate SARS-CoV-2/human/USA/MA_MGH_00075/202	TTCACCGAGGCCACGCGGAGTACGATCGAGTGTACAGTGAA
MT520525.1 Severe acute respiratory syndrome coronavirus 2 isolate SARS-CoV-2/human/USA/MA_MGH_00233/202	TTCACCGAGGCCACGCGGAGTACGATCGAGTGTACAGTGAA
MT520526.1 Severe acute respiratory syndrome coronavirus 2 isolate SARS-CoV-2/human/USA/MA_MGH_00179/202	TTCACCGAGGCCACGCGGAGTACGATCGAGTGTACAGTGAA
MT520527.1 Severe acute respiratory syndrome coronavirus 2 isolate SARS-CoV-2/human/USA/MA_MGH_00539/202	TTCACCGAGGCCACGCGGAGTACGATCGAGTGTACAGTGAA
MT520528.1 Severe acute respiratory syndrome coronavirus 2 isolate SARS-CoV-2/human/USA/MA_MGH_00165/202	TTCACCGAGGCCACGCGGAGTACGATCGAGTGTACAGTGAA
MT520529.1 Severe acute respiratory syndrome coronavirus 2 isolate SARS-CoV-2/human/USA/MA_MGH_00459/202	TTCACCGAGGCCACGCGGAGTACGATCGAGTGTACAGTGAA

MT520530.1 Severe acute respiratory syndrome coronavirus 2 isolate SARS-CoV-2/human/USA/MA_MGH_00250/202	TTCACCGAGGCCACGCGGAGTACGATCGAGTGTACAGTGAA
MT520531.1 Severe acute respiratory syndrome coronavirus 2 isolate SARS-CoV-2/human/USA/MA_MGH_00552/202	TTCACCGAGGCCACGCGGAGTACGATCGAGTGTACAGTGAA
MT520532.1 Severe acute respiratory syndrome coronavirus 2 isolate SARS-CoV-2/human/USA/MA_MGH_00181/202	TTCACCGAGGCCACGCGGAGTACGATCGAGTGTACAGTGAA
MT520533.1 Severe acute respiratory syndrome coronavirus 2 isolate SARS-CoV-2/human/USA/MA_MGH_00525/202	TTCACCGAGGCCACGCGGAGTACGATCGAGTGTACAGTGAA
MT520534.1 Severe acute respiratory syndrome coronavirus 2 isolate SARS-CoV-2/human/USA/MA_MGH_00499/202	TTCACCGAGGCCACGCGGAGTACGATCGAGTGTACAGTGAA
MT520535.1 Severe acute respiratory syndrome coronavirus 2 isolate SARS-CoV-2/human/USA/MA_MGH_00537/202	TTCACCGAGGCCACGCGGAGTACGATCGAGTGTACAGTGAA
MT520536.1 Severe acute respiratory syndrome coronavirus 2 isolate SARS-CoV-2/human/USA/MA_MGH_00295/202	TTCACCGAGGCCACGCGGAGTACGATCGAGTGTACAGTGAA
MT520537.1 Severe acute respiratory syndrome coronavirus 2 isolate SARS-CoV-2/human/USA/MA_MGH_00150/202	TTCACCGAGGCCACGCGGAGTACGATCGAGTGTACAGTGAA
MT520538.1 Severe acute respiratory syndrome coronavirus 2 isolate SARS-CoV-2/human/USA/MA_MGH_00272/202	TTCACCGAGGCCACGCGGAGTACGATCGAGTGTACAGTGAA
MT520539.1 Severe acute respiratory syndrome coronavirus 2 isolate SARS-CoV-2/human/USA/MA_MGH_00271/202	TTCACCGAGGCCACGCGGAGTACGATCGAGTGTACAGTGAA
MT520540.1 Severe acute respiratory syndrome coronavirus 2 isolate SARS-CoV-2/human/USA/MA_MGH_00679/202	TTCACCGAGGCCACGCGGAGTACGATCGAGTGTACAGTGAA
MT520541.1 Severe acute respiratory syndrome coronavirus 2 isolate SARS-CoV-2/human/USA/MA_MGH_00178/202	TTCACCGAGGCCACGCGGAGTACGATCGAGTGTACAGTGAA
MT520542.1 Severe acute respiratory syndrome coronavirus 2 isolate SARS-CoV-2/human/USA/MA_MGH_00004/202	TTCACCGAGGCCACGCGGAGTACGATCGAGTGTACAGTGAA
MT520543.1 Severe acute respiratory syndrome coronavirus 2 isolate SARS-CoV-2/human/USA/MA_MGH_00199/202	TTCACCGAGGCCACGCGGAGTACGATCGAGTGTACAGTGAA
MT520544.1 Severe acute respiratory syndrome coronavirus 2 isolate SARS-CoV-2/human/USA/MA_MGH_00028/202	TTCACCGAGGCCACGCGGAGTACGATCGAGTGTACAGTGAA
MT520545.1 Severe acute respiratory syndrome coronavirus 2 isolate SARS-CoV-2/human/USA/MA_MGH_00132/202	TTCACCGAGGCCACGCGGAGTACGATCGAGTGTACAGTGAA
MT520546.1 Severe acute respiratory syndrome coronavirus 2 isolate SARS-CoV-2/human/USA/MA_MGH_00205/202	TTCACCGAGGCCACGCGGAGTACGATCGAGTGTACAGTGAA
MT520547.1 Severe acute respiratory syndrome coronavirus 2 isolate SARS-CoV-2/human/USA/MA_MGH_00005/202	TTCACCGAGGCCACGCGGAGTACGATCGAGTGTACAGTGAA
MT522852.1 Severe acute respiratory syndrome coronavirus 2 isolate SARS-CoV-2/human/USA/UNC_200213_2020/2	TTCACCGAGGCCACGCGGAGTACGATCGAGTGTACAGTGAA
MT522853.1 Severe acute respiratory syndrome coronavirus 2 isolate SARS-CoV-2/human/USA/UNC_200214_2020/2	TTCACCGAGGCCACGCGGAGTACGATCGAGTGTACAGTGAA
MT522854.1 Severe acute respiratory syndrome coronavirus 2 isolate SARS-CoV-2/human/USA/UNC_200215_2020/2	TTCACCGAGGCCACGCGGAGTACGATCGAGTGTACAGTGAA
MT522855.1 Severe acute respiratory syndrome coronavirus 2 isolate SARS-CoV-2/human/USA/UNC_200216_2020/2	TTCACCGAGGCCACGCGGAGTACGATCGAGTGTACAGTGAA
MT525950.1 Severe acute respiratory syndrome coronavirus 2 isolate SARS-CoV-2/human/ITA/INMI3/2020, complete	TTCACCGAGGCCACGCGGAGTACGATCGAGTGTACAGTGAA
MT527178.1 Severe acute respiratory syndrome coronavirus 2 isolate SARS-CoV-2/human/ITA/INMI4/2020, complete	TTCACCGAGGCCACGCGGAGTACGATCGAGTGTACAGTGAA
MT527184.1 Severe acute respiratory syndrome coronavirus 2 isolate SARS-CoV-2/human/ITA/INMI6/2020, complete	TTCACCGAGGCCACGCGGAGTACGATCGAGTGTACAGTGAA
MT528235.1 Severe acute respiratory syndrome coronavirus 2 isolate SARS-CoV-2/human/ITA/INMI7/2020, complete	TTCACCGAGGCCACGCGGAGTACGATCGAGTGTACAGTGAA
MT528237.1 Severe acute respiratory syndrome coronavirus 2 isolate SARS-CoV-2/human/ITA/INMI8/2020, complete	TTCACCGAGGCCACGCGGAGTACGATCGAGTGTACAGTGAA
MT528238.1 Severe acute respiratory syndrome coronavirus 2 isolate SARS-CoV-2/human/ITA/INMI9/2020, complete	TTCACCGAGGCCACGCGGAGTACGATCGAGTGTACAGTGAA
MT528239.1 Severe acute respiratory syndrome coronavirus 2 isolate SARS-CoV-2/human/ITA/INMI10/2020, complet	TTCACCGAGGCCACGCGGAGTACGATCGAGTGTACAGTGAA
MT528598.1 Severe acute respiratory syndrome coronavirus 2 isolate SARS-CoV-2/human/USA/UNC_200217/2020, c	TTCACCGAGGCCACGCGGAGTACGATCGAGTGTACAGTGAA
MT528599.1 Severe acute respiratory syndrome coronavirus 2 isolate SARS-CoV-2/human/USA/UNC_200219/2020, c	TTCACCGAGGCCACGCGGAGTACGATCGAGTGTACAGTGAA
MT528600.1 Severe acute respiratory syndrome coronavirus 2 isolate SARS-CoV-2/human/USA/UNC_200238/2020, c	TTCACCGAGGCCACGCGGAGTACGATCGAGTGTACAGTGAA
MT528601.1 Severe acute respiratory syndrome coronavirus 2 isolate SARS-CoV-2/human/USA/UNC_200241/2020, c	TTCACCGAGGCCACGCGGAGTACGATCGAGTGTACAGTGAA

MT528602.1 Severe acute respiratory syndrome coronavirus 2 isolate SARS-CoV-2/human/USA/UNC_200242/2020, c	TTCACCGAGGCCACGCGGAGTACGATCGAGTGTACAGTGAA
MT528603.1 Severe acute respiratory syndrome coronavirus 2 isolate SARS-CoV-2/human/USA/UNC_200243/2020, c	TTCACCGAGGCCACGCGGAGTACGATCGAGTGTACAGTGAA
MT528604.1 Severe acute respiratory syndrome coronavirus 2 isolate SARS-CoV-2/human/USA/UNC_200244/2020, c	TTCACCGAGGCCACGCGGAGTACGATCGAGTGTACAGTGAA
MT528605.1 Severe acute respiratory syndrome coronavirus 2 isolate SARS-CoV-2/human/USA/UNC_200245/2020, c	TTCACCGAGGCCACGCGGAGTACGATCGAGTGTACAGTGAA
MT528606.1 Severe acute respiratory syndrome coronavirus 2 isolate SARS-CoV-2/human/USA/UNC_200247/2020, c	TTCACCGAGGCCACGCGGAGTACGATCGAGTGTACAGTGAA
MT528607.1 Severe acute respiratory syndrome coronavirus 2 isolate SARS-CoV-2/human/USA/UNC_200254/2020, c	TTCACCGAGGCCACGCGGAGTACGATCGAGTGTACAGTGAA
MT528608.1 Severe acute respiratory syndrome coronavirus 2 isolate SARS-CoV-2/human/USA/UNC_200255/2020, c	TTCACCGAGGCCACGCGGAGTACGATCGAGTGTACAGTGAA
MT528609.1 Severe acute respiratory syndrome coronavirus 2 isolate SARS-CoV-2/human/USA/UNC_200256/2020, c	TTCACCGAGGCCACGCGGAGTACGATCGAGTGTACAGTGAA
MT528610.1 Severe acute respiratory syndrome coronavirus 2 isolate SARS-CoV-2/human/USA/UNC_200259/2020, c	TTCACCGAGGCCACGCGGAGTACGATCGAGTGTACAGTGAA
MT528611.1 Severe acute respiratory syndrome coronavirus 2 isolate SARS-CoV-2/human/USA/UNC_200264/2020, c	TTCACCGAGGCCACGCGGAGTACGATCGAGTGTACAGTGAA
MT528612.1 Severe acute respiratory syndrome coronavirus 2 isolate SARS-CoV-2/human/USA/UNC_200271/2020, c	TTCACCGAGGCCACGCGGAGTACGATCGAGTGTACAGTGAA
MT528613.1 Severe acute respiratory syndrome coronavirus 2 isolate SARS-CoV-2/human/USA/UNC_200272/2020, c	TTCACCGAGGCCACGCGGAGTACGATCGAGTGTACAGTGAA
MT528614.1 Severe acute respiratory syndrome coronavirus 2 isolate SARS-CoV-2/human/USA/UNC_200278/2020, c	TTCACCGAGGCCACGCGGAGTACGATCGAGTGTACAGTGAA
MT528615.1 Severe acute respiratory syndrome coronavirus 2 isolate SARS-CoV-2/human/USA/UNC_200290/2020, c	TTCACCGAGGCCACGCGGAGTACGATCGAGTGTACAGTGAA
MT528616.1 Severe acute respiratory syndrome coronavirus 2 isolate SARS-CoV-2/human/USA/UNC_200300/2020, c	TTCACCGAGGCCACGCGGAGTACGATCGAGTGTACAGTGAA
MT528617.1 Severe acute respiratory syndrome coronavirus 2 isolate SARS-CoV-2/human/USA/UNC_200306/2020, c	TTCACCGAGGCCACGCGGAGTACGATCGAGTGTACAGTGAA
MT528618.1 Severe acute respiratory syndrome coronavirus 2 isolate SARS-CoV-2/human/USA/UNC_200310/2020, c	TTCACCGAGGCCACGCGGAGTACGATCGAGTGTACAGTGAA
MT528619.1 Severe acute respiratory syndrome coronavirus 2 isolate SARS-CoV-2/human/USA/UNC_200312/2020, c	TTCACCGAGGCCACGCGGAGTACGATCGAGTGTACAGTGAA
MT528620.1 Severe acute respiratory syndrome coronavirus 2 isolate SARS-CoV-2/human/USA/UNC_200318/2020, c	TTCACCGAGGCCACGCGGAGTACGATCGAGTGTACAGTGAA
MT528621.1 Severe acute respiratory syndrome coronavirus 2 isolate SARS-CoV-2/human/USA/UNC_200319/2020, c	TTCACCGAGGCCACGCGGAGTACGATCGAGTGTACAGTGAA
MT528622.1 Severe acute respiratory syndrome coronavirus 2 isolate SARS-CoV-2/human/USA/UNC_200323/2020, c	TTCACCGAGGCCACGCGGAGTACGATCGAGTGTACAGTGAA
MT528623.1 Severe acute respiratory syndrome coronavirus 2 isolate SARS-CoV-2/human/USA/UNC_200325/2020, c	TTCACCGAGGCCACGCGGAGTACGATCGAGTGTACAGTGAA
MT528624.1 Severe acute respiratory syndrome coronavirus 2 isolate SARS-CoV-2/human/USA/UNC_200326/2020, c	TTCACCGAGGCCACGCGGAGTACGATCGAGTGTACAGTGAA
MT528625.1 Severe acute respiratory syndrome coronavirus 2 isolate SARS-CoV-2/human/USA/UNC_200331/2020, c	TTCACCGAGGCCACGCGGAGTACGATCGAGTGTACAGTGAA
MT528626.1 Severe acute respiratory syndrome coronavirus 2 isolate SARS-CoV-2/human/USA/UNC_200330/2020, c	TTCACCGAGGCCACGCGGAGTACGATCGAGTGTACAGTGAA
MT528628.1 Severe acute respiratory syndrome coronavirus 2 isolate SARS-CoV-2/human/USA/UNC_200253/2020, c	TTCACCGAGGCCACGCGGAGTACGATCGAGTGTACAGTGAA
MT528629.1 Severe acute respiratory syndrome coronavirus 2 isolate SARS-CoV-2/human/USA/UNC_200340/2020, c	TTCACCGAGGCCACGCGGAGTACGATCGAGTGTACAGTGAA
MT528630.1 Severe acute respiratory syndrome coronavirus 2 isolate SARS-CoV-2/human/USA/UNC_200386/2020, c	TTCACCGAGGCCACGCGGAGTACGATCGAGTGTACAGTGAA
MT528631.1 Severe acute respiratory syndrome coronavirus 2 isolate SARS-CoV-2/human/USA/UNC_200229/2020, c	TTCACCGAGGCCACGCGGAGTACGATCGAGTGTACAGTGAA
MT531537.2 Severe acute respiratory syndrome coronavirus 2 isolate SARS-CoV-2/human/ITA/Siena-1/2020, comple	TTCACCGAGGCCACGCGGAGTACGATCGAGTGTACAGTGAA
MT532435.1 Severe acute respiratory syndrome coronavirus 2 isolate SARS-CoV-2/human/USA/VI-CDC-4411/2020, c	TTCACCGAGGCCACGCGGAGTACGATCGAGTGTACAGTGAA
MT532436.1 Severe acute respiratory syndrome coronavirus 2 isolate SARS-CoV-2/human/USA/CT-CDC-8001/2020, c	TTCACCGAGGCCACGCGGAGTACGATCGAGTGTACAGTGAA
MT532437.1 Severe acute respiratory syndrome coronavirus 2 isolate SARS-CoV-2/human/USA/CT-CDC-6001/2020, c	TTCACCGAGGCCACGCGGAGTACGATCGAGTGTACAGTGAA

MT532438.1 Severe acute respiratory syndrome coronavirus 2 isolate SARS-CoV-2/human/USA/VI-CDC-4022/2020, co	TTCACCGAGGCCACGCGGAGTACGATCGAGTGTACAGTGAA
MT532557.1 Severe acute respiratory syndrome coronavirus 2 isolate SARS-CoV-2/human/USA/TX-CDC-0406/2020, c	TTCACCGAGGCCACGCGGAGTACGATCGAGTGTACAGTGAA
MT533197.1 Severe acute respiratory syndrome coronavirus 2 isolate SARS-CoV-2/human/USA/VI-CDC-4400/2020, co	TTCACCGAGGCCACGCGGAGTACGATCGAGTGTACAGTGAA
MT533198.1 Severe acute respiratory syndrome coronavirus 2 isolate SARS-CoV-2/human/USA/VI-CDC-4371/2020, co	TTCACCGAGGCCACGCGGAGTACGATCGAGTGTACAGTGAA
MT533199.1 Severe acute respiratory syndrome coronavirus 2 isolate SARS-CoV-2/human/USA/VI-CDC-4367/2020, co	TTCACCGAGGCCACGCGGAGTACGATCGAGTGTACAGTGAA
MT533200.1 Severe acute respiratory syndrome coronavirus 2 isolate SARS-CoV-2/human/USA/VI-CDC-4408/2020, co	TTCACCGAGGCCACGCGGAGTACGATCGAGTGTACAGTGAA
MT533201.1 Severe acute respiratory syndrome coronavirus 2 isolate SARS-CoV-2/human/USA/VI-CDC-4412/2020, co	TTCACCGAGGCCACGCGGAGTACGATCGAGTGTACAGTGAA
MT533202.1 Severe acute respiratory syndrome coronavirus 2 isolate SARS-CoV-2/human/USA/VI-CDC-4413/2020, co	TTCACCGAGGCCACGCGGAGTACGATCGAGTGTACAGTGAA
MT533203.1 Severe acute respiratory syndrome coronavirus 2 isolate SARS-CoV-2/human/USA/VI-CDC-4399/2020, co	TTCACCGAGGCCACGCGGAGTACGATCGAGTGTACAGTGAA
MT533205.1 Severe acute respiratory syndrome coronavirus 2 isolate SARS-CoV-2/human/USA/CA-CZB-1271/2020, c	TTCACCGAGGCCACGCGGAGTACGATCGAGTGTACAGTGAA
MT533206.1 Severe acute respiratory syndrome coronavirus 2 isolate SARS-CoV-2/human/USA/CA-CZB-1272/2020, c	TTCACCGAGGCCACGCGGAGTACGATCGAGTGTACAGTGAA
MT533209.1 Severe acute respiratory syndrome coronavirus 2 isolate SARS-CoV-2/human/USA/CA-CZB-1275/2020 O	TTCACCGAGGCCACGCGGAGTACGATCGAGTGTACAGTGAA
MT533212.1 Severe acute respiratory syndrome coronavirus 2 isolate SARS-CoV-2/human/USA/CA-CZB-1150/2020, c	TTCACCGAGGCCACGCGGAGTACGATCGAGTGTACAGTGAA
MT533214.1 Severe acute respiratory syndrome coronavirus 2 isolate SARS-CoV-2/human/USA/CA-CZB-1156/2020 O	TTCACCGAGGCCACGCGGAGTACGATCGAGTGTACAGTGAA
MT533216.1 Severe acute respiratory syndrome coronavirus 2 isolate SARS-CoV-2/human/USA/CA-CZB-1160/2020 O	TTCACCGAGGCCACGCGGAGTACGATCGAGTGTACAGTGAA
MT533217.1 Severe acute respiratory syndrome coronavirus 2 isolate SARS-CoV-2/human/USA/CA-CZB-1163/2020, c	TTCACCGAGGCCACGCGGAGTACGATCGAGTGTACAGTGAA
MT533218.1 Severe acute respiratory syndrome coronavirus 2 isolate SARS-CoV-2/human/USA/CA-CZB-1164/2020, c	TTCACCGAGGCCACGCGGAGTACGATCGAGTGTACAGTGAA
MT533219.1 Severe acute respiratory syndrome coronavirus 2 isolate SARS-CoV-2/human/USA/CA-CZB-1168/2020, c	TTCACCGAGGCCACGCGGAGTACGATCGAGTGTACAGTGAA
MT533221.1 Severe acute respiratory syndrome coronavirus 2 isolate SARS-CoV-2/human/USA/CA-CZB-1171/2020, c	TTCACCGAGGCCACGCGGAGTACGATCGAGTGTACAGTGAA
MT533222.1 Severe acute respiratory syndrome coronavirus 2 isolate SARS-CoV-2/human/USA/CA-CZB-1174/2020, c	TTCACCGAGGCCACGCGGAGTACGATCGAGTGTACAGTGAA
MT533223.1 Severe acute respiratory syndrome coronavirus 2 isolate SARS-CoV-2/human/USA/CA-CZB-1175/2020, c	TTCACCGAGGCCACGCGGAGTACGATCGAGTGTACAGTGAA
MT533224.1 Severe acute respiratory syndrome coronavirus 2 isolate SARS-CoV-2/human/USA/CA-CZB-1176/2020 O	TTCACCGAGGCCACGCGGAGTACGATCGAGTGTACAGTGAA
MT533225.1 Severe acute respiratory syndrome coronavirus 2 isolate SARS-CoV-2/human/USA/CA-CZB-1178/2020, c	TTCACCGAGGCCACGCGGAGTACGATCGAGTGTACAGTGAA
MT533226.1 Severe acute respiratory syndrome coronavirus 2 isolate SARS-CoV-2/human/USA/CA-CZB-1179/2020, c	TTCACCGAGGCCACGCGGAGTACGATCGAGTGTACAGTGAA
MT533227.1 Severe acute respiratory syndrome coronavirus 2 isolate SARS-CoV-2/human/USA/CA-CZB-1183/2020, c	TTCACCGAGGCCACGCGGAGTACGATCGAGTGTACAGTGAA
MT533228.1 Severe acute respiratory syndrome coronavirus 2 isolate SARS-CoV-2/human/USA/CA-CZB-1186/2020, c	TTCACCGAGGCCACGCGGAGTACGATCGAGTGTACAGTGAA
MT533229.1 Severe acute respiratory syndrome coronavirus 2 isolate SARS-CoV-2/human/USA/CA-CZB-1189/2020, c	TTCACCGAGGCCACGCGGAGTACGATCGAGTGTACAGTGAA
MT533230.1 Severe acute respiratory syndrome coronavirus 2 isolate SARS-CoV-2/human/USA/CA-CZB-1193/2020, c	TTCACCGAGGCCACGCGGAGTACGATCGAGTGTACAGTGAA
MT533231.1 Severe acute respiratory syndrome coronavirus 2 isolate SARS-CoV-2/human/USA/CA-CZB-1195/2020 O	TTCACCGAGGCCACGCGGAGTACGATCGAGTGTACAGTGAA
MT533232.1 Severe acute respiratory syndrome coronavirus 2 isolate SARS-CoV-2/human/USA/CA-CZB-1277/2020, c	TTCACCGAGGCCACGCGGAGTACGATCGAGTGTACAGTGAA
MT533233.1 Severe acute respiratory syndrome coronavirus 2 isolate SARS-CoV-2/human/USA/CA-CZB-1278/2020, c	TTCACCGAGGCCACGCGGAGTACGATCGAGTGTACAGTGAA
MT533235.1 Severe acute respiratory syndrome coronavirus 2 isolate SARS-CoV-2/human/USA/CA-CZB-1280/2020, c	TTCACCGAGGCCACGCGGAGTACGATCGAGTGTACAGTGAA
MT533236.1 Severe acute respiratory syndrome coronavirus 2 isolate SARS-CoV-2/human/USA/CA-CZB-1281/2020, c	TTCACCGAGGCCACGCGGAGTACGATCGAGTGTACAGTGAA

MT533237.1 Severe acute respiratory syndrome coronavirus 2 isolate SARS-CoV-2/human/USA/CA-CZB-1282/2020, c	TTCACCGAGGCCACGCGGAGTACGATCGAGTGTACAGTGAA
MT533238.1 Severe acute respiratory syndrome coronavirus 2 isolate SARS-CoV-2/human/USA/CA-CZB-1283/2020, c	TTCACCGAGGCCACGCGGAGTACGATCGAGTGTACAGTGAA
MT534285.1 Severe acute respiratory syndrome coronavirus 2 isolate SARS-CoV-2/human/CZE/SARS-CoV-2_Czechia_	TTCACCGAGGCCACGCGGAGTACGATCGAGTGTACAGTGAA
MT534307.1 Severe acute respiratory syndrome coronavirus 2 isolate SARS-CoV-2/human/USA/CA-CZB-1237/2020, c	TTCACCGAGGCCACGCGGAGTACGATCGAGTGTACAGTGAA
MT534308.1 Severe acute respiratory syndrome coronavirus 2 isolate SARS-CoV-2/human/USA/CA-CZB-1238/2020, c	TTCACCGAGGCCACGCGGAGTACGATCGAGTGTACAGTGAA
MT534309.1 Severe acute respiratory syndrome coronavirus 2 isolate SARS-CoV-2/human/USA/CA-CZB-1239/2020, c	TTCACCGAGGCCACGCGGAGTACGATCGAGTGTACAGTGAA
MT534311.1 Severe acute respiratory syndrome coronavirus 2 isolate SARS-CoV-2/human/USA/CA-CZB-1241/2020, c	TTCACCGAGGCCACGCGGAGTACGATCGAGTGTACAGTGAA
MT534313.1 Severe acute respiratory syndrome coronavirus 2 isolate SARS-CoV-2/human/USA/CA-CZB-1243/2020, c	TTCACCGAGGCCACGCGGAGTACGATCGAGTGTACAGTGAA
MT534314.1 Severe acute respiratory syndrome coronavirus 2 isolate SARS-CoV-2/human/USA/CA-CZB-1244/2020, c	TTCACCGAGGCCACGCGGAGTACGATCGAGTGTACAGTGAA
MT534315.1 Severe acute respiratory syndrome coronavirus 2 isolate SARS-CoV-2/human/USA/CA-CZB-1245/2020, c	TTCACCGAGGCCACGCGGAGTACGATCGAGTGTACAGTGAA
MT534316.1 Severe acute respiratory syndrome coronavirus 2 isolate SARS-CoV-2/human/USA/CA-CZB-1246/2020, c	TTCACCGAGGCCACGCGGAGTACGATCGAGTGTACAGTGAA
MT534317.1 Severe acute respiratory syndrome coronavirus 2 isolate SARS-CoV-2/human/USA/CA-CZB-1247/2020, c	TTCACCGAGGCCACGCGGAGTACGATCGAGTGTACAGTGAA
MT534318.1 Severe acute respiratory syndrome coronavirus 2 isolate SARS-CoV-2/human/USA/CA-CZB-1248/2020, c	TTCACCGAGGCCACGCGGAGTACGATCGAGTGTACAGTGAA
MT534323.1 Severe acute respiratory syndrome coronavirus 2 isolate SARS-CoV-2/human/USA/CA-CZB-1253/2020, c	TTCACCGAGGCCACGCGGAGTACGATCGAGTGTACAGTGAA
MT534324.1 Severe acute respiratory syndrome coronavirus 2 isolate SARS-CoV-2/human/USA/CA-CZB-1254/2020, c	TTCACCGAGGCCACGCGGAGTACGATCGAGTGTACAGTGAA
MT534326.1 Severe acute respiratory syndrome coronavirus 2 isolate SARS-CoV-2/human/USA/CA-CZB-1256/2020, c	TTCACCGAGGCCACGCGGAGTACGATCGAGTGTACAGTGAA
MT534327.1 Severe acute respiratory syndrome coronavirus 2 isolate SARS-CoV-2/human/USA/CA-CZB-1257/2020, c	TTCACCGAGGCCACGCGGAGTACGATCGAGTGTACAGTGAA
MT534328.1 Severe acute respiratory syndrome coronavirus 2 isolate SARS-CoV-2/human/USA/CA-CZB-1258/2020, c	TTCACCGAGGCCACGCGGAGTACGATCGAGTGTACAGTGAA
MT534329.1 Severe acute respiratory syndrome coronavirus 2 isolate SARS-CoV-2/human/USA/CA-CZB-1259/2020, c	TTCACCGAGGCCACGCGGAGTACGATCGAGTGTACAGTGAA
MT534330.1 Severe acute respiratory syndrome coronavirus 2 isolate SARS-CoV-2/human/USA/CA-CZB-1260/2020, c	TTCACCGAGGCCACGCGGAGTACGATCGAGTGTACAGTGAA
MT534331.1 Severe acute respiratory syndrome coronavirus 2 isolate SARS-CoV-2/human/USA/CA-CZB-1261/2020, c	TTCACCGAGGCCACGCGGAGTACGATCGAGTGTACAGTGAA
MT534333.1 Severe acute respiratory syndrome coronavirus 2 isolate SARS-CoV-2/human/USA/CA-CZB-1263/2020, c	TTCACCGAGGCCACGCGGAGTACGATCGAGTGTACAGTGAA
MT534334.1 Severe acute respiratory syndrome coronavirus 2 isolate SARS-CoV-2/human/USA/CA-CZB-1264/2020, c	TTCACCGAGGCCACGCGGAGTACGATCGAGTGTACAGTGAA
MT534335.1 Severe acute respiratory syndrome coronavirus 2 isolate SARS-CoV-2/human/USA/CA-CZB-1265/2020, c	TTCACCGAGGCCACGCGGAGTACGATCGAGTGTACAGTGAA
MT534336.1 Severe acute respiratory syndrome coronavirus 2 isolate SARS-CoV-2/human/USA/CA-CZB-1266/2020, c	TTCACCGAGGCCACGCGGAGTACGATCGAGTGTACAGTGAA
MT534338.1 Severe acute respiratory syndrome coronavirus 2 isolate SARS-CoV-2/human/USA/CA-CZB-1268/2020, c	TTCACCGAGGCCACGCGGAGTACGATCGAGTGTACAGTGAA
MT534339.1 Severe acute respiratory syndrome coronavirus 2 isolate SARS-CoV-2/human/USA/CA-CZB-1269/2020, c	TTCACCGAGGCCACGCGGAGTACGATCGAGTGTACAGTGAA
MT534630.1 Severe acute respiratory syndrome coronavirus 2 isolate SARS-CoV-2/human/CHN/Changzhou_JS27/202	TTCACCGAGGCCACGCGGAGTACGATCGAGTGTACAGTGAA
MT535472.1 Severe acute respiratory syndrome coronavirus 2 isolate SARS-CoV-2/Human/USA/UT-00301/2020, com	TTCACCGAGGCCACGCGGAGTACGATCGAGTGTACAGTGAA
MT535473.1 Severe acute respiratory syndrome coronavirus 2 isolate SARS-CoV-2/Human/USA/UT-00303/2020 ORF1	TTCACCGAGGCCACGCGGAGTACGATCGAGTGTACAGTGAA
MT535475.1 Severe acute respiratory syndrome coronavirus 2 isolate SARS-CoV-2/Human/USA/UT-00346/2020 ORF1	TTCACCGAGGCCACGCGGAGTACGATCGAGTGTACAGTGAA
MT535476.1 Severe acute respiratory syndrome coronavirus 2 isolate SARS-CoV-2/Human/USA/UT-00352/2020 ORF1	TTCACCGAGGCCACGCGGAGTACGATCGAGTGTACAGTGAA
MT535477.1 Severe acute respiratory syndrome coronavirus 2 isolate SARS-CoV-2/Human/USA/UT-00349/2020 ORF1	TTCACCGAGGCCACGCGGAGTACGATCGAGTGTACAGTGAA

MT535479.1 Severe acute respiratory syndrome coronavirus 2 isolate SARS-CoV-2/Human/USA/UT-00347/2020 ORF1	TTCACCGAGGCCACGCGGAGTACGATCGAGTGTACAGTGAA
MT535480.1 Severe acute respiratory syndrome coronavirus 2 isolate SARS-CoV-2/Human/USA/UT-00348/2020 ORF1	TTCACCGAGGCCACGCGGAGTACGATCGAGTGTACAGTGAA
MT535481.1 Severe acute respiratory syndrome coronavirus 2 isolate SARS-CoV-2/Human/USA/UT-00361/2020, com	TTCACCGAGGCCACGCGGAGTACGATCGAGTGTACAGTGAA
MT535493.1 Severe acute respiratory syndrome coronavirus 2 isolate SARS-CoV-2/Human/USA/UT-01573/2020, com	TTCACCGAGGCCACGCGGAGTACGATCGAGTGTACAGTGAA
MT535494.1 Severe acute respiratory syndrome coronavirus 2 isolate SARS-CoV-2/Human/USA/UT-01575/2020, com	TTCACCGAGGCCACGCGGAGTACGATCGAGTGTACAGTGAA
MT535495.1 Severe acute respiratory syndrome coronavirus 2 isolate SARS-CoV-2/Human/USA/UT-01578/2020, com	TTCACCGAGGCCACGCGGAGTACGATCGAGTGTACAGTGAA
MT535497.1 Severe acute respiratory syndrome coronavirus 2 isolate SARS-CoV-2/Human/USA/UT-01581/2020, com	TTCACCGAGGCCACGCGGAGTACGATCGAGTGTACAGTGAA
MT535498.1 Severe acute respiratory syndrome coronavirus 2 isolate SARS-CoV-2/Human/USA/UT-01582/2020 ORF1	TTCACCGAGGCCACGCGGAGTACGATCGAGTGTACAGTGAA
MT535499.1 Severe acute respiratory syndrome coronavirus 2 isolate SARS-CoV-2/Human/USA/UT-01583/2020, com	TTCACCGAGGCCACGCGGAGTACGATCGAGTGTACAGTGAA
MT535500.1 Severe acute respiratory syndrome coronavirus 2 isolate SARS-CoV-2/Human/USA/UT-01585/2020, com	TTCACCGAGGCCACGCGGAGTACGATCGAGTGTACAGTGAA
MT535502.1 Severe acute respiratory syndrome coronavirus 2 isolate SARS-CoV-2/Human/USA/UT-01590/2020, com	TTCACCGAGGCCACGCGGAGTACGATCGAGTGTACAGTGAA
MT535503.1 Severe acute respiratory syndrome coronavirus 2 isolate SARS-CoV-2/Human/USA/UT-01594/2020, com	TTCACCGAGGCCACGCGGAGTACGATCGAGTGTACAGTGAA
MT535506.1 Severe acute respiratory syndrome coronavirus 2 isolate SARS-CoV-2/Human/USA/UT-01601/2020 ORF1	TTCACCGAGGCCACGCGGAGTACGATCGAGTGTACAGTGAA
MT536176.1 Severe acute respiratory syndrome coronavirus 2 isolate SARS-CoV-2/human/USA/LA-BIE-051/2020, cor	TTCACCGAGGCCACGCGGAGTACGATCGAGTGTACAGTGAA
MT536177.1 Severe acute respiratory syndrome coronavirus 2 isolate SARS-CoV-2/human/USA/LA-BIE-054/2020, cor	TTCACCGAGGCCACGCGGAGTACGATCGAGTGTACAGTGAA
MT536178.1 Severe acute respiratory syndrome coronavirus 2 isolate SARS-CoV-2/human/USA/LA-BIE-055/2020, cor	TTCACCGAGGCCACGCGGAGTACGATCGAGTGTACAGTGAA
MT536179.1 Severe acute respiratory syndrome coronavirus 2 isolate SARS-CoV-2/human/USA/LA-BIE-056/2020, cor	TTCACCGAGGCCACGCGGAGTACGATCGAGTGTACAGTGAA
MT536180.1 Severe acute respiratory syndrome coronavirus 2 isolate SARS-CoV-2/human/USA/LA-BIE-058/2020, cor	TTCACCGAGGCCACGCGGAGTACGATCGAGTGTACAGTGAA
MT536181.1 Severe acute respiratory syndrome coronavirus 2 isolate SARS-CoV-2/human/USA/LA-BIE-059/2020, cor	TTCACCGAGGCCACGCGGAGTACGATCGAGTGTACAGTGAA
MT536182.1 Severe acute respiratory syndrome coronavirus 2 isolate SARS-CoV-2/human/USA/LA-BIE-061/2020, cor	TTCACCGAGGCCACGCGGAGTACGATCGAGTGTACAGTGAA
MT536183.1 Severe acute respiratory syndrome coronavirus 2 isolate SARS-CoV-2/human/USA/LA-BIE-062/2020, cor	TTCACCGAGGCCACGCGGAGTACGATCGAGTGTACAGTGAA
MT536184.1 Severe acute respiratory syndrome coronavirus 2 isolate SARS-CoV-2/human/USA/LA-BIE-063/2020, cor	TTCACCGAGGCCACGCGGAGTACGATCGAGTGTACAGTGAA
MT536185.1 Severe acute respiratory syndrome coronavirus 2 isolate SARS-CoV-2/human/USA/LA-BIE-064/2020, cor	TTCACCGAGGCCACGCGGAGTACGATCGAGTGTACAGTGAA
MT536186.1 Severe acute respiratory syndrome coronavirus 2 isolate SARS-CoV-2/human/USA/LA-BIE-065/2020, cor	TTCACCGAGGCCACGCGGAGTACGATCGAGTGTACAGTGAA
MT536187.1 Severe acute respiratory syndrome coronavirus 2 isolate SARS-CoV-2/human/USA/LA-BIE-066/2020, cor	TTCACCGAGGCCACGCGGAGTACGATCGAGTGTACAGTGAA
MT536188.1 Severe acute respiratory syndrome coronavirus 2 isolate SARS-CoV-2/human/USA/LA-BIE-068/2020, cor	TTCACCGAGGCCACGCGGAGTACGATCGAGTGTACAGTGAA
MT536189.1 Severe acute respiratory syndrome coronavirus 2 isolate SARS-CoV-2/human/USA/LA-BIE-069/2020, cor	TTCACCGAGGCCACGCGGAGTACGATCGAGTGTACAGTGAA
MT536190.1 Severe acute respiratory syndrome coronavirus 2 isolate SARS-CoV-2/human/USA/LA-BIE-070/2020, cor	TTCACCGAGGCCACGCGGAGTACGATCGAGTGTACAGTGAA
MT536954.1 Severe acute respiratory syndrome coronavirus 2 isolate SARS-CoV-2/Human/USA/UT-01677/2020 ORF1	TTCACCGAGGCCACGCGGAGTACGATCGAGTGTACAGTGAA
MT536955.1 Severe acute respiratory syndrome coronavirus 2 isolate SARS-CoV-2/Human/USA/UT-01679/2020 ORF1	TTCACCGAGGCCACGCGGAGTACGATCGAGTGTACAGTGAA
MT536956.1 Severe acute respiratory syndrome coronavirus 2 isolate SARS-CoV-2/Human/USA/UT-01683/2020, com	TTCACCGAGGCCACGCGGAGTACGATCGAGTGTACAGTGAA
MT536958.1 Severe acute respiratory syndrome coronavirus 2 isolate SARS-CoV-2/Human/USA/UT-01686/2020 ORF1	TTCACCGAGGCCACGCGGAGTACGATCGAGTGTACAGTGAA
MT536959.1 Severe acute respiratory syndrome coronavirus 2 isolate SARS-CoV-2/Human/USA/UT-01687/2020 ORF1	TTCACCGAGGCCACGCGGAGTACGATCGAGTGTACAGTGAA

MT536961.1 Severe acute respiratory syndrome coronavirus 2 isolate SARS-CoV-2/Human/USA/UT-01693/2020, com	TTCACCGAGGCCACGCGGAGTACGATCGAGTGTACAGTGAA
MT536962.1 Severe acute respiratory syndrome coronavirus 2 isolate SARS-CoV-2/Human/USA/UT-01694/2020, com	TTCACCGAGGCCACGCGGAGTACGATCGAGTGTACAGTGAA
MT536963.1 Severe acute respiratory syndrome coronavirus 2 isolate SARS-CoV-2/Human/USA/UT-01696/2020 ORF1	TTCACCGAGGCCACGCGGAGTACGATCGAGTGTACAGTGAA
MT536964.1 Severe acute respiratory syndrome coronavirus 2 isolate SARS-CoV-2/Human/USA/UT-01892/2020, com	TTCACCGAGGCCACGCGGAGTACGATCGAGTGTACAGTGAA
MT536965.1 Severe acute respiratory syndrome coronavirus 2 isolate SARS-CoV-2/Human/USA/UT-01893/2020, com	TTCACCGAGGCCACGCGGAGTACGATCGAGTGTACAGTGAA
MT536966.1 Severe acute respiratory syndrome coronavirus 2 isolate SARS-CoV-2/Human/USA/UT-01894/2020 ORF1	TTCACCGAGGCCACGCGGAGTACGATCGAGTGTACAGTGAA
MT536968.1 Severe acute respiratory syndrome coronavirus 2 isolate SARS-CoV-2/Human/USA/UT-01896/2020 ORF1	TTCACCGAGGCCACGCGGAGTACGATCGAGTGTACAGTGAA
MT536974.1 Severe acute respiratory syndrome coronavirus 2 isolate SARS-CoV-2/Human/USA/UT-03950/2020, com	TTCACCGAGGCCACGCGGAGTACGATCGAGTGTACAGTGAA
MT536976.1 Severe acute respiratory syndrome coronavirus 2 isolate SARS-CoV-2/Human/USA/UT-02024/2020, com	TTCACCGAGGCCACGCGGAGTACGATCGAGTGTACAGTGAA
MT536977.1 Severe acute respiratory syndrome coronavirus 2 isolate SARS-CoV-2/Human/USA/UT-02025/2020, com	TTCACCGAGGCCACGCGGAGTACGATCGAGTGTACAGTGAA
MT539158.1 Severe acute respiratory syndrome coronavirus 2 isolate SARS-CoV-2/human/BGD/BCSIR_NILMRC_007/	TTCACCGAGGCCACGCGGAGTACGATCGAGTGTACAGTGAA
MT539159.1 Severe acute respiratory syndrome coronavirus 2 isolate SARS-CoV-2/human/BGD/BCSIR_NILMRC_006/	TTCACCGATGCCACGCGGAGTACGATCGAGTGTACAGTGAA
MT539160.1 Severe acute respiratory syndrome coronavirus 2 isolate SARS-CoV-2/human/BGD/BCSIR_NILMRC_008/	TTCACCGAGGCCACGCGGAGTACGATCGAGTGTACAGTGAA
MT539162.1 Severe acute respiratory syndrome coronavirus 2 isolate SARS-CoV-2/human/USA/GA-EHC-009I/2020, c	TTCACCGAGGCCACGCGGAGTACGATCGAGTGTACAGTGAA
MT539163.1 Severe acute respiratory syndrome coronavirus 2 isolate SARS-CoV-2/human/USA/GA-EHC-086H/2020, c	TTCACCGAGGCCACGCGGAGTACGATCGAGTGTACAGTGAA
MT539164.1 Severe acute respiratory syndrome coronavirus 2 isolate SARS-CoV-2/human/IND/GBRC124/2020, comp	TTCACCGAGGCCACGCGGAGTACGATCGAGTGTACAGTGAA
MT539165.1 Severe acute respiratory syndrome coronavirus 2 isolate SARS-CoV-2/human/IND/GBRC125/2020, comp	TTCACCGAGGCCACGCGGAGTACGATCGAGTGTACAGTGAA
MT539166.1 Severe acute respiratory syndrome coronavirus 2 isolate SARS-CoV-2/human/IND/GBRC121/2020, comp	TTCACCGAGGCCACGCGGAGTACGATCGAGTGTACAGTGAA
MT539167.1 Severe acute respiratory syndrome coronavirus 2 isolate SARS-CoV-2/human/IND/GBRC126/2020, comp	TTCACCGAGGCCACGCGGAGTACGATCGAGTGTACAGTGAA
MT539168.1 Severe acute respiratory syndrome coronavirus 2 isolate SARS-CoV-2/human/IND/GBRC127/2020, comp	TTCACCGAGGCCACGCGGAGTACGATCGAGTGTACAGTGAA
MT539169.1 Severe acute respiratory syndrome coronavirus 2 isolate SARS-CoV-2/human/IND/GBRC129/2020, comp	TTCACCGAGGCCACGCGGAGTACGATCGAGTGTACAGTGAA
MT539170.1 Severe acute respiratory syndrome coronavirus 2 isolate SARS-CoV-2/human/IND/GBRC80/2020, comple	TTCACCGAGGCCACACGGAGTACGATCGAGTGTACAGTGAA
MT539171.1 Severe acute respiratory syndrome coronavirus 2 isolate SARS-CoV-2/human/IND/GBRC122/2020, comp	TTCACCGAGGCCACGCGGAGTACGATCGAGTGTACAGTGAA
MT539172.1 Severe acute respiratory syndrome coronavirus 2 isolate SARS-CoV-2/human/IND/GBRC128/2020, comp	TTCACCGAGGCCACGCGGAGTACGATCGAGTGTACAGTGAA
MT539173.1 Severe acute respiratory syndrome coronavirus 2 isolate SARS-CoV-2/human/IND/GBRC120/2020, comp	TTCACCGAGGCCACGCGGAGTACGATCGAGTGTACAGTGAA
MT539174.1 Severe acute respiratory syndrome coronavirus 2 isolate SARS-CoV-2/human/IND/GBRC119/2020, comp	TTCACCGAGGCCACACGGAGTACGATCGAGTGTACAGTGAA
MT539175.1 Severe acute respiratory syndrome coronavirus 2 isolate SARS-CoV-2/human/IND/GBRC130/2020, comp	TTCACCGAGGCCACGCGGAGTACGATCGAGTGTACAGTGAA
MT539176.1 Severe acute respiratory syndrome coronavirus 2 isolate SARS-CoV-2/human/IND/GBRC123/2020, comp	TTCACCGAGGCCACGCGGAGTACGATCGAGTGTACAGTGAA
MT539729.1 Severe acute respiratory syndrome coronavirus 2 isolate SARS-CoV-2/environment/USA/UF-12/2020, co	TTCACCGAGGCCACGCGGAGTACGATCGAGTGTACAGTGAA
MT547814.1 Severe acute respiratory syndrome coronavirus 2 isolate SARS-CoV-2/human/USA/NR-52282/2020, com	TTCACCGAGGCCACGCGGAGTACGATCGAGTGTACAGTGAA
MT549887.1 Severe acute respiratory syndrome coronavirus 2 isolate SARS-CoV-2/human/KEN/D5/2020 ORF1ab poly	TTCACCGAGGCCACGCGGAGTACGATCGAGTGTACAGTGAA
MT551604.1 Severe acute respiratory syndrome coronavirus 2 isolate SARS-CoV-2/human/USA/UNC_200459/2020, c	TTCACCGAGGCCACGCGGAGTACGATCGAGTGTACAGTGAA
MT551605.1 Severe acute respiratory syndrome coronavirus 2 isolate SARS-CoV-2/human/USA/UNC_200460/2020, c	TTCACCGAGGCCACGCGGAGTACGATCGAGTGTACAGTGAA

MT551606.1 Severe acute respiratory syndrome coronavirus 2 isolate SARS-CoV-2/human/USA/UNC_200467/2020, c	TTCACCGAGGCCACGCGGAGTACGATCGAGTGTACAGTGAA
MT551607.1 Severe acute respiratory syndrome coronavirus 2 isolate SARS-CoV-2/human/USA/UNC_200468/2020, c	TTCACCGAGGCCACGCGGAGTACGATCGAGTGTACAGTGAA
MT551608.1 Severe acute respiratory syndrome coronavirus 2 isolate SARS-CoV-2/human/USA/UNC_200486/2020, c	TTCACCGAGGCCACGCGGAGTACGATCGAGTGTACAGTGAA
MT551609.1 Severe acute respiratory syndrome coronavirus 2 isolate SARS-CoV-2/human/USA/UNC_200488/2020, c	TTCACCGAGGCCACGCGGAGTACGATCGAGTGTACAGTGAA
MT554052.1 Severe acute respiratory syndrome coronavirus 2 isolate SARS-CoV-2/human/USA/UNC_200222/2020, c	TTCACCGAGGCCACGCGGAGTACGATCGAGTGTACAGTGAA
MT557568.1 Severe acute respiratory syndrome coronavirus 2 isolate SARS-CoV-2/human/USA/UNC_200429/2020, c	TTCACCGAGGCCACACGGAGTACGATCGAGTGTACAGTGAA
MT557569.1 Severe acute respiratory syndrome coronavirus 2 isolate SARS-CoV-2/human/USA/UNC_200445/2020, c	TTCACCGAGGCCACGCGGAGTACGATCGAGTGTACAGTGAA
MT557570.1 Severe acute respiratory syndrome coronavirus 2 isolate SARS-CoV-2/human/USA/UNC_200446/2020, c	TTCACCGAGGCCACGCGGAGTACGATCGAGTGTACAGTGAA
MT558649.1 Severe acute respiratory syndrome coronavirus 2 isolate SARS-CoV-2/human/USA/VA-DCLS-0220/2020,	TTCACCGAGGCCACGCGGAGTACGATCGAGTGTACAGTGAA
MT558651.1 Severe acute respiratory syndrome coronavirus 2 isolate SARS-CoV-2/human/USA/VA-DCLS-0223/2020,	TTCACCGAGGCCACGCGGAGTACGATCGAGTGTACAGTGAA
MT558653.1 Severe acute respiratory syndrome coronavirus 2 isolate SARS-CoV-2/human/USA/VA-DCLS-0225/2020,	TTCACCGAGGCCACGCGGAGTACGATCGAGTGTACAGTGAA
MT558658.1 Severe acute respiratory syndrome coronavirus 2 isolate SARS-CoV-2/human/USA/VA-DCLS-0230/2020,	TTCACCGAGGCCACGCGGAGTACGATCGAGTGTACAGTGAA
MT558660.1 Severe acute respiratory syndrome coronavirus 2 isolate SARS-CoV-2/human/USA/VA-DCLS-0232/2020,	TTCACCGAGGCCACGCGGAGTACGATCGAGTGTACAGTGAA
MT558661.1 Severe acute respiratory syndrome coronavirus 2 isolate SARS-CoV-2/human/USA/VA-DCLS-0233/2020,	TTCACCGAGGCCACGCGGAGTACGATCGAGTGTACAGTGAA
MT558662.1 Severe acute respiratory syndrome coronavirus 2 isolate SARS-CoV-2/human/USA/VA-DCLS-0236/2020,	TTCACCGAGGCCACGCGGAGTACGATCGAGTGTACAGTGAA
MT558663.1 Severe acute respiratory syndrome coronavirus 2 isolate SARS-CoV-2/human/USA/VA-DCLS-0237/2020,	TTCACCGAGGCCACGCGGAGTACGATCGAGTGTACAGTGAA
MT558665.1 Severe acute respiratory syndrome coronavirus 2 isolate SARS-CoV-2/human/USA/VA-DCLS-0239/2020,	TTCACCGAGGCCACGCGGAGTACGATCGAGTGTACAGTGAA
MT558666.1 Severe acute respiratory syndrome coronavirus 2 isolate SARS-CoV-2/human/USA/VA-DCLS-0240/2020,	TTCACCGAGGCCACGCGGAGTACGATCGAGTGTACAGTGAA
MT558667.1 Severe acute respiratory syndrome coronavirus 2 isolate SARS-CoV-2/human/USA/VA-DCLS-0241/2020,	TTCACCGAGGCCACGCGGAGTACGATCGAGTGTACAGTGAA
MT558671.1 Severe acute respiratory syndrome coronavirus 2 isolate SARS-CoV-2/human/USA/VA-DCLS-0246/2020,	TTCACCGAGGCCACGCGGAGTACGATCGAGTGTACAGTGAA
MT558672.1 Severe acute respiratory syndrome coronavirus 2 isolate SARS-CoV-2/human/USA/VA-DCLS-0247/2020,	TTCACCGAGGCCACGCGGAGTACGATCGAGTGTACAGTGAA
MT558673.1 Severe acute respiratory syndrome coronavirus 2 isolate SARS-CoV-2/human/USA/VA-DCLS-0248/2020,	TTCACCGAGGCCACGCGGAGTACGATCGAGTGTACAGTGAA
MT558674.1 Severe acute respiratory syndrome coronavirus 2 isolate SARS-CoV-2/human/USA/VA-DCLS-0249/2020,	TTCACCGAGGCCACGCGGAGTACGATCGAGTGTACAGTGAA
MT558677.1 Severe acute respiratory syndrome coronavirus 2 isolate SARS-CoV-2/human/USA/VA-DCLS-0252/2020,	TTCACCGAGGCCACGCGGAGTACGATCGAGTGTACAGTGAA
MT558680.1 Severe acute respiratory syndrome coronavirus 2 isolate SARS-CoV-2/human/USA/VA-DCLS-0255/2020,	TTCACCGAGGCCACGCGGAGTACGATCGAGTGTACAGTGAA
MT558681.1 Severe acute respiratory syndrome coronavirus 2 isolate SARS-CoV-2/human/USA/VA-DCLS-0257/2020,	TTCACCGAGGCCACGCGGAGTACGATCGAGTGTACAGTGAA
MT558683.1 Severe acute respiratory syndrome coronavirus 2 isolate SARS-CoV-2/human/USA/VA-DCLS-0259/2020,	TTCACCGAGGCCACGCGGAGTACGATCGAGTGTACAGTGAA
MT558684.1 Severe acute respiratory syndrome coronavirus 2 isolate SARS-CoV-2/human/USA/VA-DCLS-0260/2020,	TTCACCGAGGCCACGCGGAGTACGATCGAGTGTACAGTGAA
MT558685.1 Severe acute respiratory syndrome coronavirus 2 isolate SARS-CoV-2/human/USA/VA-DCLS-0261/2020,	TTCACCGAGGCCACGCGGAGTACGATCGAGTGTACAGTGAA
MT558686.1 Severe acute respiratory syndrome coronavirus 2 isolate SARS-CoV-2/human/USA/VA-DCLS-0262/2020,	TTCACCGAGGCCACGCGGAGTACGATCGAGTGTACAGTGAA
MT558689.1 Severe acute respiratory syndrome coronavirus 2 isolate SARS-CoV-2/human/USA/VA-DCLS-0265/2020,	TTCACCGAGGCCACGCGGAGTACGATCGAGTGTACAGTGAA
MT558691.1 Severe acute respiratory syndrome coronavirus 2 isolate SARS-CoV-2/human/USA/VA-DCLS-0271/2020,	TTCACCGAGGCCACGCGGAGTACGATCGAGTGTACAGTGAA
MT558695.1 Severe acute respiratory syndrome coronavirus 2 isolate SARS-CoV-2/human/USA/VA-DCLS-0275/2020,	TTCACCGAGGCCACGCGGAGTACGATCGAGTGTACAGTGAA

MT558696.1 Severe acute respiratory syndrome coronavirus 2 isolate SARS-CoV-2/human/USA/VA-DCLS-0276/2020,	TTCACCGAGGCCACGCGGAGTACGATCGAGTGTACAGTGAA
MT558699.1 Severe acute respiratory syndrome coronavirus 2 isolate SARS-CoV-2/human/USA/VA-DCLS-0279/2020,	TTCACCGAGGCCACGCGGAGTACGATCGAGTGTACAGTGAA
MT558701.1 Severe acute respiratory syndrome coronavirus 2 isolate SARS-CoV-2/human/USA/VA-DCLS-0281/2020,	TTCACCGAGGCCACGCGGAGTACGATCGAGTGTACAGTGAA
MT558702.1 Severe acute respiratory syndrome coronavirus 2 isolate SARS-CoV-2/human/USA/VA-DCLS-0282/2020,	TTCACCGAGGCCACGCGGAGTACGATCGAGTGTACAGTGAA
MT558703.1 Severe acute respiratory syndrome coronavirus 2 isolate SARS-CoV-2/human/USA/VA-DCLS-0283/2020,	TTCACCGAGGCCACGCGGAGTACGATCGAGTGTACAGTGAA
MT558704.1 Severe acute respiratory syndrome coronavirus 2 isolate SARS-CoV-2/human/USA/VA-DCLS-0284/2020,	TTCACCGAGGCCACGCGGAGTACGATCGAGTGTACAGTGAA
MT558705.1 Severe acute respiratory syndrome coronavirus 2 isolate SARS-CoV-2/human/USA/VA-DCLS-0285/2020,	TTCACCGAGGCCACGCGGAGTACGATCGAGTGTACAGTGAA
MT559800.1 Severe acute respiratory syndrome coronavirus 2 isolate SARS-CoV-2/human/USA/UNC_200395_2020/2	TTCACCGAGGCCACGCGGAGTACGATCGAGTGTACAGTGAA
MT559801.1 Severe acute respiratory syndrome coronavirus 2 isolate SARS-CoV-2/human/USA/UNC_200399_2020/2	TTCACCGAGGCCACGCGGAGTACGATCGAGTGTACAGTGAA
MT559802.1 Severe acute respiratory syndrome coronavirus 2 isolate SARS-CoV-2/human/USA/UNC_200401_2020/2	TTCACCGAGGCCACGCGGAGTACGATCGAGTGTACAGTGAA
MT559803.1 Severe acute respiratory syndrome coronavirus 2 isolate SARS-CoV-2/human/USA/UNC_200409_2020/2	TTCACCGAGGCCACGCGGAGTACGATCGAGTGTACAGTGAA
MT560525.1 Severe acute respiratory syndrome coronavirus 2 isolate SARS-CoV-2/human/TUR/IMU-SP-02/2020, con	TTCACCGAGGCCACGCGGAGTACGATCGAGTGTACAGTGAA
MT560530.1 Severe acute respiratory syndrome coronavirus 2 isolate SARS-CoV-2/human/TUR/IMU-SP-01/2020, con	TTCACCGAGGCCACGCGGAGTACGATCGAGTGTACAGTGAA
MT560531.1 Severe acute respiratory syndrome coronavirus 2 isolate SARS-CoV-2/human/TUR/IMU-SP-04/2020, con	TTCACCGAGGCCACGCGGAGTACGATCGAGTGTACAGTGAA
MT560656.1 Severe acute respiratory syndrome coronavirus 2 isolate SARS-CoV-2/human/IND/GBRC131/2020, comp	TTCACCGAGGCCACGCGGAGTACGATCGAGTGTACAGTGAA
MT560657.1 Severe acute respiratory syndrome coronavirus 2 isolate SARS-CoV-2/human/IND/GBRC132/2020, comp	TTCACCGAGGCCACGCGGAGTACGATCGAGTGTACAGTGAA
MT560667.1 Severe acute respiratory syndrome coronavirus 2 isolate SARS-CoV-2/human/IND/GBRC135b/2020, com	TTCACCGAGGCCACGCGGAGTACGATCGAGTGTACAGTGAA
MT560668.1 Severe acute respiratory syndrome coronavirus 2 isolate SARS-CoV-2/human/IND/GBRC140/2020, comp	TTCACCGAGGCCACGCGGAGTACGATCGAGTGTACAGTGAA
MT560669.1 Severe acute respiratory syndrome coronavirus 2 isolate SARS-CoV-2/human/IND/GBRC146b/2020, com	TTCACCGAGGCCACGCGGAGTACGATCGAGTGTACAGTGAA
MT560670.1 Severe acute respiratory syndrome coronavirus 2 isolate SARS-CoV-2/human/IND/GBRC139b/2020, com	TTCACCGAGGCCACGCGGAGTACGATCGAGTGTACAGTGAA
MT560671.1 Severe acute respiratory syndrome coronavirus 2 isolate SARS-CoV-2/human/IND/GBRC133/2020, comp	TTCACCGAGGCCACGCGGAGTACGATCGAGTGTACAGTGAA
MT560672.1 Severe acute respiratory syndrome coronavirus 2 isolate SARS-CoV-2/human/IND/GBRC134/2020, comp	TTCACCGAGGCCACGCGGAGTACGATCGAGTGTACAGTGAA
MT560673.1 Severe acute respiratory syndrome coronavirus 2 isolate SARS-CoV-2/human/IND/GBRC145/2020, comp	TTCACCGAGGCCACGCGGAGTACGATCGAGTGTACAGTGAA
MT560674.1 Severe acute respiratory syndrome coronavirus 2 isolate SARS-CoV-2/human/IND/GBRC135a/2020, com	TTCACCGAGGCCACGCGGAGTACGATCGAGTGTACAGTGAA
MT560675.1 Severe acute respiratory syndrome coronavirus 2 isolate SARS-CoV-2/human/IND/GBRC146a/2020, com	TTCACCGAGGCCACGCGGAGTACGATCGAGTGTACAGTGAA
MT560677.1 Severe acute respiratory syndrome coronavirus 2 isolate SARS-CoV-2/human/IND/GBRC147/2020, comp	TTCACCGAGGCCACGCGGAGTACGATCGAGTGTACAGTGAA
MT560679.1 Severe acute respiratory syndrome coronavirus 2 isolate SARS-CoV-2/human/IND/GBRC136a/2020, com	TTCACCGAGGCCACGCGGAGTACGATCGAGTGTACAGTGAA
MT560680.1 Severe acute respiratory syndrome coronavirus 2 isolate SARS-CoV-2/human/IND/GBRC141a/2020, com	TTCACCGAGGCCACGCGGAGTACGATCGAGTGTACAGTGAA
MT560682.1 Severe acute respiratory syndrome coronavirus 2 isolate SARS-CoV-2/human/IND/GBRC141b/2020, com	TTCACCGAGGCCACGCGGAGTACGATCGAGTGTACAGTGAA
MT560689.1 Severe acute respiratory syndrome coronavirus 2 isolate SARS-CoV-2/human/IND/GBRC143/2020, comp	TTCACCGAGGCCACGCGGAGTACGATCGAGTGTACAGTGAA
MT560690.1 Severe acute respiratory syndrome coronavirus 2 isolate SARS-CoV-2/human/IND/GBRC142/2020, comp	TTCACCGAGGCCACGCGGAGTACGATCGAGTGTACAGTGAA
MT560692.1 Severe acute respiratory syndrome coronavirus 2 isolate SARS-CoV-2/human/IND/GBRC137a/2020, com	TTCACCGAGGCCACGCGGAGTACGATCGAGTGTACAGTGAA
MT560693.1 Severe acute respiratory syndrome coronavirus 2 isolate SARS-CoV-2/human/IND/GBRC137b/2020, com	TTCACCGAGGCCACGCGGAGTACGATCGAGTGTACAGTGAA

MT560694.1 Severe acute respiratory syndrome coronavirus 2 isolate SARS-CoV-2/human/IND/GBRC148a/2020, com	TTCACCGAGGCCACGCGGAGTACGATCGAGTGTACAGTGAA
MT560704.1 Severe acute respiratory syndrome coronavirus 2 isolate SARS-CoV-2/human/IND/GBRC136b/2020, com	TTCACCGAGGCCACGCGGAGTACGATCGAGTGTACAGTGAA
MT560705.1 Severe acute respiratory syndrome coronavirus 2 isolate SARS-CoV-2/human/IND/GBRC149/2020, comp	TTCACCGAGGCCACGCGGAGTACGATCGAGTGTACAGTGAA
MT560827.1 Severe acute respiratory syndrome coronavirus 2 isolate SARS-CoV-2/human/IND/GBRC144/2020, comp	TTCACCGAGGCCACGCGGAGTACGATCGAGTGTACAGTGAA
NC_045512.2 Severe acute respiratory syndrome coronavirus 2 isolate Wuhan-Hu-1, complete genome	TTCACCGAGGCCACGCGGAGTACGATCGAGTGTACAGTGAA
MT345876.1 Severe acute respiratory syndrome coronavirus 2 isolate SARS-CoV-2/human/USA/ID-UW-4378/2020, co	TTCACCGAGGCCACGCGGAGTACGATCGAGTGAACAATGCT
MT358650.1 Severe acute respiratory syndrome coronavirus 2 isolate SARS-CoV-2/human/USA/WA-UW-4228/2020,	TTCACCGAGGCCACGCGGAGTACGATCGAGTGTAAACAATG
MT412316.1 Severe acute respiratory syndrome coronavirus 2 isolate SARS-CoV-2/human/USA/WA-UW-6243/2020,	TTCACCGAGGCCACGCGGAGTACGATCGAGTGTAAACAATG
MT451044.1 Severe acute respiratory syndrome coronavirus 2 isolate SARS-CoV-2/human/AUS/VIC140/2020, comple	TTCACCGAGGCCACGCGGAGTACGATCGAGTGTAGTGAACA
MT461649.1 Severe acute respiratory syndrome coronavirus 2 isolate SARS-CoV-2/human/USA/UNKNOWN-UW-6504	TTCACCGAGGCCACGCGGAGTACGATCGAGTGTAAACAATG
MT509649.1 Severe acute respiratory syndrome coronavirus 2 isolate SARS-CoV-2/human/IND/GBRC112/2020, comp	TTCACCGAGGCCACGCGGAGTACGATCGAGTGAACAATGCT
MT114414.1 Severe acute respiratory syndrome coronavirus 2 isolate SARS-CoV-2/human/HKG/HKU-903a/2020, con	TTCACCGAGGCCACTCGGAGTACGATCGAGTGTACAGTGAA
MT114415.1 Severe acute respiratory syndrome coronavirus 2 isolate SARS-CoV-2/human/HKG/HKU-903b/2020, con	TTCACCGAGGCCACTCGGAGTACGATCGAGTGTACAGTGAA
MT258379.1 Severe acute respiratory syndrome coronavirus 2 isolate SARS-CoV-2/human/USA/CZB-RR057-007/2020	TTCACCGAGGCCACGCGGAATACGATCGAGTGTACAGTGAA
MT281530.2 Severe acute respiratory syndrome coronavirus 2 isolate SARS-CoV-2/human/IRN/HGRC-01-IPI-8206/20	TTCACCGAGGCCACTCGGAGTACGATCGAGTGTACAGTGAA
MT320891.2 Severe acute respiratory syndrome coronavirus 2 isolate SARS-CoV-2/human/IRN/HGRC-1.1-IPI-8206/20	TTCACCGAGGCCACTCGGAGTACGATCGAGTGTACAGTGAA
MT327745.1 Severe acute respiratory syndrome coronavirus 2 isolate SARS-CoV-2/human/TUR/ERAGEM-001/2020,	TTCACCGAGGCCACTCGGAGTACGATCGAGTGTACAGTGAA
MT365028.1 Severe acute respiratory syndrome coronavirus 2 isolate SARS-CoV-2/human/HKG/HKU-905a/2020, con	TTCACCGAGGCCACTCGGAGTACGATCGAGTGTACAGTGAA
MT370516.1 Severe acute respiratory syndrome coronavirus 2 isolate SARS-CoV-2/human/TWN/CGMH-CGU-03/2020	TTCACCGAGGCCACTCGGAGTACGATCGAGTGTACAGTGAA
MT370517.1 Severe acute respiratory syndrome coronavirus 2 isolate SARS-CoV-2/human/TWN/CGMH-CGU-04/2020	TTCACCGAGGCCACTCGGAGTACGATCGAGTGTACAGTGAA
MT370518.1 Severe acute respiratory syndrome coronavirus 2 isolate SARS-CoV-2/human/TWN/CGMH-CGU-05/2020	TTCACCGAGGCCACTCGGAGTACGATCGAGTGTACAGTGAA
MT370904.1 Severe acute respiratory syndrome coronavirus 2 isolate SARS-CoV-2/human/USA/NY1-PV08001/2020,	TTCACCGAGGCCACTCGGAGTACGATCGAGTGTACAGTGAA
MT370953.1 Severe acute respiratory syndrome coronavirus 2 isolate SARS-CoV-2/human/USA/NY-PV09122/2020, c	TTCACCGAGGCCACGTGGAGTACGATCGAGTGTACAGTGAA
MT371047.1 Severe acute respiratory syndrome coronavirus 2 isolate SARS-CoV-2/human/LKA/COV38/2020, comple	TTCACCGAGGCCACTCGGAGTACGATCGAGTGTACAGTGAA
MT374102.1 Severe acute respiratory syndrome coronavirus 2 isolate SARS-CoV-2/human/TWN/CGMH-CGU-06/2020	TTCACCGAGGCCACTCGGAGTACGATCGAGTGTACAGTGAA
MT374103.1 Severe acute respiratory syndrome coronavirus 2 isolate SARS-CoV-2/human/TWN/CGMH-CGU-07/2020	TTCACCGAGGCCACTCGGAGTACGATCGAGTGTACAGTGAA
MT374104.1 Severe acute respiratory syndrome coronavirus 2 isolate SARS-CoV-2/human/TWN/CGMH-CGU-08/2020	TTCACCGAGGCCACTCGGAGTACGATCGAGTGTACAGTGAA
MT374106.1 Severe acute respiratory syndrome coronavirus 2 isolate SARS-CoV-2/human/TWN/CGMH-CGU-10/2020	TTCACCGAGGCCACTCGGAGTACGATCGAGTGTACAGTGAA
MT374107.1 Severe acute respiratory syndrome coronavirus 2 isolate SARS-CoV-2/human/TWN/CGMH-CGU-11/2020	TTCACCGAGGCCACTCGGAGTACGATCGAGTGTACAGTGAA
MT375476.1 Severe acute respiratory syndrome coronavirus 2 isolate SARS-CoV-2/human/USA/WA-UW-5144/2020,	TTCACCGAGGCCACGCGGATTACGATCGAGTGTACAGTGAA
MT412159.1 Severe acute respiratory syndrome coronavirus 2 isolate SARS-CoV-2/human/USA/MI-MDHHS-SC20034	TTCACCGAGGCCACTCGGAGTACGATCGAGTGTACAGTGAA
MT439255.1 Severe acute respiratory syndrome coronavirus 2 isolate SARS-CoV-2/human/USA/MI-MDHHS-SC20159	TTCACCGAGGCCACTCGGAGTACGATCGAGTGTACAGTGAA
MT447177.1 Severe acute respiratory syndrome coronavirus 2 isolate SARS-CoV-2/human/IRN/HGRC-2-2162/2020, c	TTCACCGAGGCCACTCGGAGTACGATCGAGTGTACAGTGAA

MT450921.1 Severe acute respiratory syndrome coronavirus 2 isolate SARS-CoV-2/human/AUS/VIC04/2020 ORF1ab	TTCACCGAGGCCACTCGGAGTACGATCGAGTGTACAGTGAA
MT450930.1 Severe acute respiratory syndrome coronavirus 2 isolate SARS-CoV-2/human/AUS/VIC13/2020 ORF1ab	TTCACCGAGGCCACGTGGAGTACGATCGAGTGTACAGTGAA
MT450945.1 Severe acute respiratory syndrome coronavirus 2 isolate SARS-CoV-2/human/AUS/VIC30/2020 ORF1ab	TTCACCGAGGCCACTCGGAGTACGATCGAGTGTACAGTGAA
MT450949.1 Severe acute respiratory syndrome coronavirus 2 isolate SARS-CoV-2/human/AUS/VIC34/2020, complet	TTCACCGAGGCCACTCGGAGTACGATCGAGTGTACAGTGAA
MT450955.1 Severe acute respiratory syndrome coronavirus 2 isolate SARS-CoV-2/human/AUS/VIC40/2020, complet	TTCACCGAGGCCACGCGGAGTACTATCGAGTGTACAGTGAA
MT450956.1 Severe acute respiratory syndrome coronavirus 2 isolate SARS-CoV-2/human/AUS/VIC41/2020, complet	TTCACCGAGGCCACGCGGAGTACTATCGAGTGTACAGTGAA
MT450973.1 Severe acute respiratory syndrome coronavirus 2 isolate SARS-CoV-2/human/AUS/VIC58/2020, complet	TTCACCGAGGCCACTCGGAGTACGATCGAGTGTACAGTGAA
MT450980.1 Severe acute respiratory syndrome coronavirus 2 isolate SARS-CoV-2/human/AUS/VIC66/2020, complet	TTCACCGAGGCCACTCGGAGTACGATCGAGTGTACAGTGAA
MT451007.1 Severe acute respiratory syndrome coronavirus 2 isolate SARS-CoV-2/human/AUS/VIC97/2020, complet	TTCACCGAGGCCACTCGGAGTACGATCGAGTGTACAGTGAA
MT451168.1 Severe acute respiratory syndrome coronavirus 2 isolate SARS-CoV-2/human/AUS/VIC272/2020, compl	TTCACCGAGGCCACTCGGAGTACGATCGAGTGTACAGTGAA
MT451186.1 Severe acute respiratory syndrome coronavirus 2 isolate SARS-CoV-2/human/AUS/VIC294/2020, compl	TTCACCGAGGCCACTCGGAGTACGATCGAGTGTACAGTGAA
MT451315.1 Severe acute respiratory syndrome coronavirus 2 isolate SARS-CoV-2/human/AUS/VIC447/2020 ORF1ab	TTCACCGAGGCCACTCGGAGTACGATCGAGTGTACAGTGAA
MT451375.1 Severe acute respiratory syndrome coronavirus 2 isolate SARS-CoV-2/human/AUS/VIC547/2020, compl	TTCACCGAGGCCACTCGGAGTACGATCGAGTGTACAGTGAA
MT451423.1 Severe acute respiratory syndrome coronavirus 2 isolate SARS-CoV-2/human/AUS/VIC615/2020 ORF1ab	TTCACCGAGGCCACTCGGAGTACGATCGAGTGTACAGTGAA
MT451433.1 Severe acute respiratory syndrome coronavirus 2 isolate SARS-CoV-2/human/AUS/VIC627/2020 ORF1ab	TTCACCGAGGCCACTCGGAGTACGATCGAGTGTACAGTGAA
MT451435.1 Severe acute respiratory syndrome coronavirus 2 isolate SARS-CoV-2/human/AUS/VIC629/2020 ORF1ab	TTCACCGAGGCCACTCGGAGTACGATCGAGTGTACAGTGAA
MT451447.1 Severe acute respiratory syndrome coronavirus 2 isolate SARS-CoV-2/human/AUS/VIC645/2020, compl	TTCACCGAGGCCACTCGGAGTACGATCGAGTGTACAGTGAA
MT451476.1 Severe acute respiratory syndrome coronavirus 2 isolate SARS-CoV-2/human/AUS/VIC680/2020, compl	TTCACCGAGGCCACTCGGAGTACGATCGAGTGTACAGTGAA
MT451479.1 Severe acute respiratory syndrome coronavirus 2 isolate SARS-CoV-2/human/AUS/VIC683/2020 ORF1ab	TTCACCGAGGCCACTCGGAGTACGATCGAGTGTACAGTGAA
MT451520.1 Severe acute respiratory syndrome coronavirus 2 isolate SARS-CoV-2/human/AUS/VIC747/2020 ORF1ab	TTCACCGAGGCCACTCGGAGTACGATCGAGTGTACAGTGAA
MT451523.1 Severe acute respiratory syndrome coronavirus 2 isolate SARS-CoV-2/human/AUS/VIC752/2020 ORF1ab	TTCACCGAGGCCACTCGGAGTACGATCGAGTGTACAGTGAA
MT451582.1 Severe acute respiratory syndrome coronavirus 2 isolate SARS-CoV-2/human/AUS/VIC828/2020, compl	TTCACCGAGGCCACGCGTAGTACGATCGAGTGTACAGTGAA
MT451590.1 Severe acute respiratory syndrome coronavirus 2 isolate SARS-CoV-2/human/AUS/VIC846/2020, compl	TTCACCGAGGCCACTCGGAGTACGATCGAGTGTACAGTGAA
MT451616.1 Severe acute respiratory syndrome coronavirus 2 isolate SARS-CoV-2/human/AUS/VIC879/2020, compl	TTCACCGAGGCCACTCGGAGTACGATCGAGTGTACAGTGAA
MT451677.1 Severe acute respiratory syndrome coronavirus 2 isolate SARS-CoV-2/human/AUS/VIC992/2020 ORF1ab	TTCACCGAGGCCACTCGGAGTACGATCGAGTGTACAGTGAA
MT451681.1 Severe acute respiratory syndrome coronavirus 2 isolate SARS-CoV-2/human/AUS/VIC996/2020 ORF1ab	TTCACCGAGGCCACTCGGAGTACGATCGAGTGTACAGTGAA
MT451712.1 Severe acute respiratory syndrome coronavirus 2 isolate SARS-CoV-2/human/AUS/VIC1042/2020, comp	TTCACCGAGGCCACTCGGAGTACGATCGAGTGTACAGTGAA
MT451713.1 Severe acute respiratory syndrome coronavirus 2 isolate SARS-CoV-2/human/AUS/VIC1043/2020, comp	TTCACCGAGGCCACTCGGAGTACGATCGAGTGTACAGTGAA
MT451753.1 Severe acute respiratory syndrome coronavirus 2 isolate SARS-CoV-2/human/AUS/VIC1114/2020 ORF1a	TTCACCGAGGCCACTCGGAGTACGATCGAGTGTACAGTGAA
MT451754.1 Severe acute respiratory syndrome coronavirus 2 isolate SARS-CoV-2/human/AUS/VIC1115/2020, comp	TTCACCGAGGCCACTCGGAGTACGATCGAGTGTACAGTGAA
MT451771.1 Severe acute respiratory syndrome coronavirus 2 isolate SARS-CoV-2/human/AUS/VIC1144/2020, comp	TTCACCGAGGCCACTCGGAGTACGATCGAGTGTACAGTGAA
MT451801.1 Severe acute respiratory syndrome coronavirus 2 isolate SARS-CoV-2/human/AUS/VIC1210/2020 ORF1a	TTCACCGAGGCCACTCGGAGTACGATCGAGTGTACAGTGAA
MT451816.1 Severe acute respiratory syndrome coronavirus 2 isolate SARS-CoV-2/human/AUS/VIC1233/2020 ORF1a	TTCACCGAGGCCACTCGGAGTACGATCGAGTGTACAGTGAA

MT451830.1 Severe acute respiratory syndrome coronavirus 2 isolate SARS-CoV-2/human/AUS/VIC1255/2020 ORF1a	TTCACCGAGGCCACTCGGAGTACGATCGAGTGTACAGTGAA
MT459903.1 Severe acute respiratory syndrome coronavirus 2 isolate SARS-CoV-2/human/GRC/40_36244/2020, com	TTCACCGAGGCCACGCGGAGTACGATTGAGTGTACAGTGAA
MT459928.1 Severe acute respiratory syndrome coronavirus 2 isolate SARS-CoV-2/human/IRN/KHGRC-3-2178/2020	TTCACCGAGGCCACTCGGAGTACGATCGAGTGTACAGTGAA
MT461626.1 Severe acute respiratory syndrome coronavirus 2 isolate SARS-CoV-2/human/USA/WA-UW-4270/2020,	TTCACCGAGGCCACTCGGAGTACGATCGAGTGTACAGTGAA
MT509495.1 Severe acute respiratory syndrome coronavirus 2 isolate SARS-CoV-2/human/IND/GBRC100/2020, com	TTCACCGAGGCCACGTGGAGTACGATCGAGTGTACAGTGAA
MT510718.1 Severe acute respiratory syndrome coronavirus 2 isolate SARS-CoV-2/human/USA/hCoV-19-USA-CA-UC	TTCACCGAGGCCACGCGGAATACGATCGAGTGTACAGTGAA
MT520384.1 Severe acute respiratory syndrome coronavirus 2 isolate SARS-CoV-2/human/USA/MA_MGH_00227/20	TTCACCGAGGCCACGCGGAGTATGATCGAGTGTACAGTGAA
MT534320.1 Severe acute respiratory syndrome coronavirus 2 isolate SARS-CoV-2/human/USA/CA-CZB-1250/2020,	TTCACCGAGGCCACGCGGAGTACTATCGAGTGTACAGTGAA
MT558698.1 Severe acute respiratory syndrome coronavirus 2 isolate SARS-CoV-2/human/USA/VA-DCLS-0278/2020,	TTCACCGAGGCCACGCGTAGTACGATCGAGTGTACAGTGAA
MT560706.1 Severe acute respiratory syndrome coronavirus 2 isolate SARS-CoV-2/human/IND/GBRC148b/2020, con	TTCACCTAGGCCACGTGGAGTACGATCGAGTGTACAGTGAA
MT007544.1 Severe acute respiratory syndrome coronavirus 2 isolate Australia/VIC01/2020, complete genome	TTCACCGAGGCCACGCGGAGTATACAGTGAA

Supplementary table 2. s2m-containing insect and insect-associated virus accessions (TSA, wgs and nt databases).

GenBank fasta defline	s2m	Percent identical amino acids (tblastx against O. brumata ORF)	Percent similar amino acids (tblastx against O. brumata ORF)	Lenght of longest match (tblastx against O. brumata ORF; > 60 amino acids; word size 6)
JTDY01007770.1 Operophtera brumata isolate WM2013NL OBRU01_Sc07770, whole genome shotgun sequence	AAGACCGCGGCCACGGCGAGTAGCATCGAGGGTACAGTCTA	100.0	100.0	332
JTDY01006214.1 Operophtera brumata isolate WM2013NL OBRU01_Sc06214, whole genome shotgun sequence	AAGACCGCGGCCACGGCGAGTAGCATCGAGGGTACAGTCTA	98.9	98.9	276
GGEG01005650.1 TSA: Papaipema speciosissima TR11259_c3_g1_i1 transcribed RNA sequence	GGAACCGCGGCCACGGCGAGTAGCATCGAGGGTACAGTTCC	85.7	88.9	63
GFBH01062097.1 TSA: Eogystia hippophaecolus c59808.graph_c0 transcribed RNA sequence	GGAACCGCGGCCACGGCGAGTAGCATCGAGGGTACAGTTCC	81.5	91.3	276
GFQL01002404.1 TSA: Carposina sasakii c33921.graph_c0 transcribed RNA sequence	TTGACCGCGGCCACGGCGAGCAGCATCGAGGGTACAGTCAA	80.8	88.4	276
GGMY01058424.1 TSA: Carposina sasakii c61052.graph_c0 transcribed RNA sequence	TTGACCGCGGCCACGGCGAGCAGCATCGAGGGTACAGTCAA	80.4	88.0	276
GITA01057804.1 TSA: Busseola fusca TRINITY_DN9419_c1_g1_i2, transcribed RNA sequence	ACGACCGCGGCCACGGCGAGTAGCATCGAGGGTACAGTCGA	78.9	88.4	275
GISL01057532.1 TSA: Busseola fusca TRINITY_DN21055_c1_g1_i4, transcribed RNA sequence	ACGACCGCGGCCACGGCGAGTAGCATCGAGGGTACAGTCGA	78.9	88.4	275
ICPK01012159.1 TSA: Bombyx mori mRNA, KWMTBOMO08170.mrna1, mRNA sequence	AAGACCGCGGCCACGGCGAGTAGCATCGAGGGTACAGTCCA	78.6	87.0	276
PYSK01000168.1 Bombyx mandarina breed wild silkmoth isolate Bman2017 BmanSCF0168, whole genome shotgun sequence	AAGACCGCGGCCACGGCGAGTAGCATCGAGGGTACAGTCCA	78.6	87.0	276
BHWX01000014.1 Bombyx mori p50T DNA, chromosome 14, draft genome sequence, whole genome shotgun sequence	AAGACCGCGGCCACGGCGAGTAGCATCGAGGGTACAGTCCA	78.6	87.0	276
BABH01042137.1 Bombyx mori DNA, contig: Bm_scaf266_contig42137, strain: p50T/Dazao, build 2, whole genome shotgun sequence	AAGACCGCGGCCACGGCGAGTAGCATCGAGGGTACAGTCCA	78.6	87.0	276
BAAB01085593.1 Bombyx mori DNA, contig432075, whole genome shotgun sequence	AAGACCGCGGCCACGGCGAGTAGCATCGAGGGTACAGTCCA	78.6	87.0	276
AADK01022785.1 Bombyx mori strain Dazao Ctg022785, whole genome shotgun sequence	AAGACCGCGGCCACGGCGAGTAGCATCGAGGGTACAGTCCA	78.6	87.0	276
GITI01044303.1 TSA: Busseola fusca TRINITY_DN9382_c1_g1_i9, transcribed RNA sequence	ACGACCGCGGCCACGGCGAGTAGCATCGAGGGTACAGTCGA	78.5	88.0	275
GITI01044301.1 TSA: Busseola fusca TRINITY_DN9382_c1_g1_i7, transcribed RNA sequence	ACGACCGCGGCCACGGCGAGTAGCATCGAGGGTACAGTCGA	78.5	88.0	275
GITI01044299.1 TSA: Busseola fusca TRINITY_DN9382_c1_g1_i5, transcribed RNA sequence	ACGACCGCGGCCACGGCGAGTAGCATCGAGGGTACAGTCGA	78.5	88.0	275
GITB01044371.1 TSA: Busseola fusca TRINITY_DN7911_c2_g1_i4, transcribed RNA sequence	ACGACCGCGGCCACGGCGAGTAGCATCGAGGGTACAGTCGA	78.5	88.0	275

GITB01044368.1 TSA: Busseola fusca TRINITY_DN7911_c2_g1_i1, transcribed RNA sequence	ACGACCGCGGCCACGGCGAGTAGCATCGAGGGTACAGTCGA	78.5	88.0	275
GCOE01018299.1 TSA: Canaea sp. AD-2014 breed wildtype C86630_a_24_0_l_5249, transcribed RNA sequence	AAGACCGCGGCCACGGCGAGTAGCATCGAGGGTACAGTCAC	77.9	87.3	276
CADEBC010000479.1 Arctia plantaginis genome assembly, contig: WW_tarseq_467_arrow, whole genome shotgun sequence	AAGACCGCGGCCACGGCGAGTAGCATCGAGGGTACAGTCGA	76.7	88.4	275
CADEBC010000479.1 Arctia plantaginis genome assembly, contig: WW_tarseq_467_arrow, whole genome shotgun sequence, s2m copy2	AAGACCGCGGCCACGGCGAGTAGCATCGAGGGTACAGTCGA			
CADEBC010000479.1 Arctia plantaginis genome assembly, contig: WW_tarseq_467_arrow, whole genome shotgun sequence, s2m copy3	AAGACCGCGGCCACGGCGAGTAGCATCGAGGGTACAGTCGA			
CADEBC010000479.1 Arctia plantaginis genome assembly, contig: WW_tarseq_467_arrow, whole genome shotgun sequence, s2m copy4	AAGACCGCGGCCACGGCGAGTAGCATCGAGGGTACAGTCGA			
CADEBC010000479.1 Arctia plantaginis genome assembly, contig: WW_tarseq_467_arrow, whole genome shotgun sequence, s2m copy5	AAGACCGCGGCCACGGCGAGTAGCATCGAGGGTACAGTCGA			
GGJZ01050084.1 TSA: Colias eurytheme c49482_g1_i1 transcribed RNA sequence	AAGACCGCGGCCACGGCGAGTAGCATCGAGGGTACAGTCAA	75.6	87.6	275
JAAIXJ010000890.1 Heliconius hermathena isolate HHFA19 scaffold1039, whole genome shotgun sequence	TAGACCGCGGCCACGGCGAGTAGCATCGAGGGTACAGTCTA	75.3	85.6	194
GEOR01025604.1 TSA: Tineola bisselliella c16418_g1_i6 transcribed RNA sequence	CAGACCGCGGCCACCGCGAGTAGCATCGAGGGTACAGTCTG	74.3	87.1	70
GEOR01025603.1 TSA: Tineola bisselliella c16418_g1_i5 transcribed RNA sequence	CAGACCGCGGCCACCGCGAGTAGCATCGAGGGTACAGTCTG	74.3	87.1	70
GENN01043111.1 TSA: Tineola bisselliella k64_228137 transcribed RNA sequence	CAGACCGCGGCCACCGCGAGTAGCATCGAGGGTACAGTCTG	74.3	87.1	70
GENN01039634.1 TSA: Tineola bisselliella k62_244082 transcribed RNA sequence	CAGACCGCGGCCACCGCGAGTAGCATCGAGGGTACAGTCTG	74.3	87.1	70
FZQP02000013.1 Leptidea sinapis genome assembly, contig: scaffold_1007, whole genome shotgun sequence	ATTTCCGAGGCCACGCCGAGTAGGATCGAGGGTACAGATTT	72.7	85.6	132
GGMY01062431.1 TSA: Carposina sasakii c64255.graph_c0 transcribed RNA sequence	GAAACCGCGGCCACGGCGAGTAGCATCGAGGGTATATCCTA	72.1	79.8	262
FZQQ01051305.1 Leptidea sinapis genome assembly, contig: 389492, whole genome shotgun sequence	AAGACCGCGGCCACGGCGAGTAGCATCGAGGGTACAGTCAA	72.0	85.3	75
GFQL01065269.1 TSA: Carposina sasakii c47486.graph_c0 transcribed RNA sequence	GAAACCGCGGCCACGGCGAGTAGCATCGAGGGTATATCCTA	71.1	78.5	256
GEOR01017005.1 TSA: Tineola bisselliella c13348_g1_i1 transcribed RNA sequence	AGAACCGCGGCCACGGCGAGTAGCATCGAGGGTACAGTTCA	70.0	85.8	120
GENN01022965.1 TSA: Tineola bisselliella k54_280947 transcribed RNA sequence	AGAACCGCGGCCACGGCGAGTAGCATCGAGGGTACAGTTCA	70.0	85.8	120

GENN01016377.1 TSA: Tineola bisselliella k52_300886 transcribed RNA sequence	AGAACCGCGGCCACGGCGAGTAGCATCGAGGGTACAGTTCA	70.0	85.8	120
GCDF01003044.1 TSA: Tineola bisselliella breed wildtype C112123_a_13_0_l_1294, transcribed RNA sequence	AGAACCGCGGCCACGGCGAGTAGCATCGAGGGTACAGTTCA	70.0	85.0	120
GEOE01034415.1 TSA: Enteucha acetosae TR19729-c0_g1_i3 transcribed RNA sequence	AGAACCGCGGCCACGGCGAGTAGCATCGAGGGTACAGTTCA	67.1	77.0	152
FZQQ01004690.1 Leptidea sinapis genome assembly, contig: 13507, whole genome shotgun sequence	AGGACCGAGGCCACGGCGAGTAGCATCGAGGGTACAGTCAC	65.8	76.7	202
FZQP02000061.1 Leptidea sinapis genome assembly, contig: scaffold_1050, whole genome shotgun sequence	AGGACCGAGGCCACGGCGAGTAGCATCGAGGGTACAGTCAC	65.8	76.7	202
GEOP01024552.1 TSA: Palaephatus nielseni TR24637-c426_g1_i3 transcribed RNA sequence	GAGACCGCGGCCACGGCGAGTAGCATCGAGGGAGTACTCTG	64.0	80.9	89
VKGM01017184.1 Busseola fusca NODE_6615_length_6671_cov_10.7465, whole genome shotgun sequence	GGAACCGCGGCCACGGCGAGTAGCATCGAGGTTACAGTTCC	62.9	75.0	132
CACRSI010001100.1 Ophraella communa genome assembly, contig: utg2634, whole genome shotgun sequence	CTGATCGAGGCCACGCCGAGTAGGATCGAGGGTACAATCAT	57.9	71.1	76
JAATLQ010018920.1 Heliconius erato x Heliconius himera isolate 6700 EI_a_scaffold_18921, whole genome shotgun sequence	TAGACCGCGGCCACGGCGAGTAGCATCGAGGGTACAGTCTA	56.6	65.8	76
JAATLQ010011463.1 Heliconius erato x Heliconius himera isolate 6700 EI_a_scaffold_11463, whole genome shotgun sequence	TAGACCGCGGCCACGGCGAGTAGCATCGAGGGTACAGTCTA	56.6	65.8	76
FAPW01237974.1 Laparus doris genome assembly Heliconius_doris.JM-02-1939, contig HEL_1_TGACCA_L001_R1_002_(paired)_contig_237974, whole genome shotgun sequence	CGGACCGCGGCCACGGCGAGTAGCATCGAGGGTACAGTCTA	56.6	64.5	76
GCOG01003970.1 TSA: Harmaclona sp. AD-2014 breed wildtype C112859_a_6_0_l_614, transcribed RNA sequence	ATTGCCGAGGCCACGCCGAGTAGGATCGAGGGTACAGTTAG	54.1	79.7	74
NJDD01016235.1 Calephelis nemesis scaffold44209_cov46, whole genome shotgun sequence	TAAACCGAGGCCACGCCGAGTAGGAACGAGGGTACAGTTTC	51.7	69.1	149
GDRP01024643.1 TSA: Bactrocera dorsalis Unigene8561 transcribed RNA sequence	GAGAAGGGGGCCACGCCGAGTAGGATCGAGGGTACACAGTC	48.3	70.0	120
CACRSI010004861.1 Ophraella communa genome assembly, contig: utg23932, whole genome shotgun sequence	CTGATCGAGGCCACGCCGAGTAGGAACGAGGGTACAATCTT	48.1	64.8	162
JADDYM010000020.1 Tribolium madens breed KSU strain isolate multiple individuals Scaffold_20;HRSCAF=117;Tcas5.2_LG4, whole genome shotgun sequence	GCAACCGAGGCCACGGCGAGTAGCATCGAGGGTACAGTTAT	48.0	63.7	102
CAACVG010007633.1 Callosobruchus maculatus genome assembly, contig: 2572|quiver, whole genome shotgun sequence	AGCACCGAGGCCACGCCGAGTAGGATCGAGGGTACAGTATA	46.9	61.1	162
KX884680.1 Hubei tetragnatha maxillosa virus 9 strain QTM27093 hypothetical protein 5 gene, complete cds	AGAACGGAGGCCACGCCGAGTAGGATCGAGGGTACACTTCA	43.8	58.8	345

KX884603.1 Hubei tetragnatha maxillosa virus 9 strain arthropodmix14049 hypothetical protein 5 gene, complete cds	AGAACAGAGGCCACGCCGAGTAGGATCGAGGGTACACTTCA	43.8	58.8	345
GAKP01014522.1 TSA: Bactrocera dorsalis comp28584_c0_seq2 transcribed RNA sequence	GAGAAGGGGGCCACGCCGAGTAGGATCGAGGGTACACAGTC	43.0	61.4	158
GFOA01028225.1 TSA: Bactrocera bryoniae TR14889_c0_g1_i1 transcribed RNA sequence	GAGAAGGGGGCCACGCCGAGTAGGATCGAGGGTACACAGTC	42.4	60.8	158
GEYS01016804.1 TSA: Bactrocera dorsalis Unigene9384_All transcribed RNA sequence	GAGAAGGGGGCCACGCCGAGTAGGATCGAGGGTACACAGTC	42.4	61.4	158
MN661054.1 Atrato Partiti-like virus 1 strain Mati 1755-173 segment 2 genomic sequence		39.0	57.0	341
NC_033766.1 Wuhan cricket virus 2 strain WHXS3745 segment Seg 3 putative capsid protein gene, complete cds		33.0	48.0	321
GHZM01212397.1 TSA: Cryptotermes secundus isolate worker+soldier TR139812_c0_g1_i2_len=1211, transcribed RNA sequence	TAATCCGAGGCCACGGCGAGTAGCATCGAGGGTACAGATTA	32.3	49.2	65
NC_033454.1 Wuhan Millipede virus 4 strain GCM8225 segment Seg 4 putative capsid protein gene, complete cds		31.0	46.0	332
VMOF01000002.1 Trialeurodes vaporariorum isolate IVF HIC_SCAFFOLD_10, whole genome shotgun sequence	TTGGCCGAGGCCACGCGGCGTACGATCGAGGGTACAGTCAA	29.9	38.8	67
VMOF01000002.1 Trialeurodes vaporariorum isolate IVF HIC_SCAFFOLD_10, whole genome shotgun sequence, s2m copy2	TTGGCCGAGGCCACGCGGCGTACGATCGAGGGTACAGTCAA			
VMOF01000002.1 Trialeurodes vaporariorum isolate IVF HIC_SCAFFOLD_10, whole genome shotgun sequence, s2m copy3	TTGGCCGAGGCCACGCGGCGTACGATCGAGGGTACAGTCAA			
GBDP01030094.1 TSA: Kerria lacca L_7914_T_3/3_C_0.900_L_8212 transcribed RNA sequence	CAGACCGAGGCCACGCGGAGAAAGATCGAGGGTACAGCTAA	23.3	42.2	116
GBDP01006251.1 TSA: Kerria lacca L_7914_T_2/3_C_0.950_L_8318 transcribed RNA sequence	CAGACCGAGGCCACGCGGAGAAAGATCGAGGGTACAGCTAA	23.3	42.2	116
GBDO01056046.1 TSA: Kerria lacca L_15684_T_1/1_C_1.000_L_1707 transcribed RNA sequence	CAGACCGAGGCCACGCGGAGAAAGATCGAGGGTACAGCTAA	23.3	42.2	116
GBDY01029361.1 TSA: Kerria lacca L_1158_T_9/11_C_0.152_L_1268 transcribed RNA sequence	GAGACGGGGGCCACGCGGAGTACGATCGAGGGTACACAATC	21.2	45.8	118
GBDY01008231.1 TSA: Kerria lacca L_1158_T_2/11_C_0.152_L_1268 transcribed RNA sequence	GAGACGGGGGCCACGCGGAGTACGATCGAGGGTACACAATC	21.2	45.8	118
GBDY01003609.1 TSA: Kerria lacca L_1158_T_3/11_C_0.152_L_1268 transcribed RNA sequence	GAGACGGGGGCCACGCGGAGTACGATCGAGGGTACACAATC	21.2	45.8	118
JABDTM010027565.1 Tenebrio molitor breed Stoneville strain (Morales-Ramos et al 2019) 69394, whole genome shotgun sequence	TTGTCCGAGGCCACGCGGAGTACGATCGAGGGTACAGACAC			
JABDTM010027511.1 Tenebrio molitor breed Stoneville strain (Morales-Ramos et al 2019) 69337, whole genome shotgun sequence	TTGTCCGAGGCCACGCGGAGTACGATCGAGGGTACAGACAC			

GHMB01079684.1 TSA: Trialeurodes vaporariorum Cluster-3686.67963, transcribed RNA sequence	TTGGCCGAGGCCACGCGGCGTACGATCGAGGGTACAGTCAA			
GHMB01079682.1 TSA: Trialeurodes vaporariorum Cluster-3686.67965, transcribed RNA sequence	TTGGCCGAGGCCACGCGGCGTACGATCGAGGGTACAGTCAA			
GHMB01079681.1 TSA: Trialeurodes vaporariorum Cluster-3686.67964, transcribed RNA sequence	TTGGCCGAGGCCACGCGGCGTACGATCGAGGGTACAGTCAA			
GHMB01079679.1 TSA: Trialeurodes vaporariorum Cluster-3686.67966, transcribed RNA sequence	TTGGCCGAGGCCACGCGGCGTACGATCGAGGGTACAGTCAA			
GHMB01040353.1 TSA: Trialeurodes vaporariorum Cluster-2989.0, transcribed RNA sequence	TTGGCCGAGGCCACGCGGCGTACGATCGAGGGTACAGTCAA			
GHMB01040352.1 TSA: Trialeurodes vaporariorum Cluster-2989.1, transcribed RNA sequence	TTGGCCGAGGCCACGCGGCGTACGATCGAGGGTACAGTCAA			
GAWX02040816.1 TSA: Trialeurodes vaporariorum s1815_L_2005_1_a_90_8_l_998 transcribed RNA sequence	TTGGCCGAGGCCACGCGGCGTACGATCGAGGGTACAGTCAA			
GAWX02040815.1 TSA: Trialeurodes vaporariorum s1814_L_2005_0_a_85_8_l_1004 transcribed RNA sequence	TTGGCCGAGGCCACGCGGCGTACGATCGAGGGTACAGTCAA			
VJOP01000419.1 Trialeurodes vaporariorum isolate WFBEAN10G scaffold000447, whole genome shotgun sequence	TTGGCCGAGGCCACGCGGCGTACGATCGAGGGTACAGTCAA			
GERW01004890.1 TSA: Eupolybothrus fasciatus breed wildtype S4919_Lo_8219_0_abu3_0_le271, transcribed RNA sequence	TTGACCGAGGCCACGCCGAGTAGGTTCGAGGGTACAGTCTG			
JXOW01185347.1 Condylostylus patibulatus isolate BV_Cpatibulatus scaffold474029, whole genome shotgun sequence	TAGACGGGGGCCACGCCGAGTAGGATCGAGGGTACACAATC			
GAVB02021154.1 TSA: Triodia sylvina C170429_a_4_0_l_818 transcribed RNA sequence	TAGACCGCGGCCACGGCGAGTAGCATCGAGGGTACAGTCTA			
GHZM01212396.1 TSA: Cryptotermes secundus isolate worker+soldier TR139812_c0_g1_i1_len=1129, transcribed RNA sequence	TAATCCGAGGCCACGGCGAGTAGCATCGAGGGTACAGATTA			
GCXS01066566.1 TSA: Mesovelia mulsanti breed wildtype s27735_L_70523_0_5_6_LINEAR, transcribed RNA sequence	GTGACCGAGGCCACGGCGAGTAGCATCGAGGGTACAGTCAG			
GCQJ01007407.1 TSA: Ohmella baetica bolivari breed wildtype C128684_a_11_0_l_444, transcribed RNA sequence	GTGACCGAGGCCACGGCGAGTAGCAGCGAGGGTACAGTCTT			
GCSI01028243.1 TSA: Ornatoraphidia flavilabris breed wildtype C244629_a_24_0_l_1128, transcribed RNA sequence	GTGACCGAGGCCACGGCGAGTAGCAACGAGGGTACAGTCGT			
GCWA01009192.1 TSA: Aphelonotus fraterculus breed wildtype C138822_5_0, transcribed RNA sequence	GTGAACGAGGCCACGCCGAGTAGGATCGAGGGTACAGTCAC			
GGKE01046505.1 TSA: Aspidiotus destructor TRINITY_DN167863_c0_g1_i1, transcribed RNA sequence	GGCTCCGCGGCCACGGCGAGTAGCAACGAGGGTACAGAGCT			
GFCT01042449.1 TSA: Mythimna separata c29599_g3 transcribed RNA sequence	GGAACCGCGGCCACGGCGAGTAGCATCGAGGGTACAGTTCC			
GADH01002239.1 TSA: Corydalinae sp. KMRSPBM-2012 contig02402 mRNA sequence	GCGACCGAGGCCACGGCGAGTAGCGACGAGGGTACGGTCCT			
GGKE01301625.1 TSA: Aspidiotus destructor TRINITY_DN90852_c0_g1_i1, transcribed RNA sequence	GCAACCGCGGCCACGCCGAGTAGGATCGAGGGTACAGTTGA			

GGKE01086018.1 TSA: Aspidiotus destructor TRINITY_DN183318_c0_g1_i1, transcribed RNA sequence	GCAACCGCGACCACGCCGAGTAGGATCGAGGGTACAGTTTG			
GCXJ01017022.1 TSA: Acutaspis umbonifera breed wildtype C156212_3_0, transcribed RNA sequence	GATACCGCGGCCACGCCGAGTAGGATCGAGGGATCAAGTAC			
GASV02022734.1 TSA: Notostira elongata s3217_L_5239_0_a_5_0_l_557 transcribed RNA sequence	GAGTCCGAGGCCACGGCGAGTAGCAGCGAGGGTACAGGTGA			
GFMG01255032.1 TSA: Gryllus bimaculatus TRINITY_DN166296_c1_g1_i4 transcribed RNA sequence	GAGGCAGAGGCCACGCAGCACACGATCGAGGGTACAGCCTC			
GFMG01255032.1 TSA: Gryllus bimaculatus TRINITY_DN166296_c1_g1_i4 transcribed RNA sequence, s2m copy2	GAGGCAGAGGCCACGCAGCACACGATCGAGGGTACAGCCTC			
GFMG01255031.1 TSA: Gryllus bimaculatus TRINITY_DN166296_c1_g1_i3 transcribed RNA sequence	GAGGCAGAGGCCACGCAGCACACGATCGAGGGTACAGCCTC			
GFMG01255030.1 TSA: Gryllus bimaculatus TRINITY_DN166296_c1_g1_i2 transcribed RNA sequence	GAGGCAGAGGCCACGCAGCACACGATCGAGGGTACAGCCTC			
GFMG01255029.1 TSA: Gryllus bimaculatus TRINITY_DN166296_c1_g1_i1 transcribed RNA sequence	GAGGCAGAGGCCACGCAGCACACGATCGAGGGTACAGCCTC			
GBDY01033195.1 TSA: Kerria lacca L_1158_T_6/11_C_0.061_L_1594 transcribed RNA sequence	GAGACGGGGGCCACGCGGAGTACGATCGAGGGTACACAATC			
GBDY01029169.1 TSA: Kerria lacca L_1158_T_11/11_C_0.394_L_1713 transcribed RNA sequence	GAGACGGGGGCCACGCGGAGTACGATCGAGGGTACACAATC			
GBDY01019523.1 TSA: Kerria lacca L_1158_T_10/11_C_0.242_L_1717 transcribed RNA sequence	GAGACGGGGGCCACGCGGAGTACGATCGAGGGTACACAATC			
GBDY01009137.1 TSA: Kerria lacca L_1158_T_4/11_C_0.364_L_1722 transcribed RNA sequence	GAGACGGGGGCCACGCGGAGTACGATCGAGGGTACACAATC			
GEOP01024550.1 TSA: Palaephatus nielseni TR24637-c426_g1_i1 transcribed RNA sequence	GAGACCGCGGCCACGGCGAGTAGCATCGAGGGAGTACTCTG			
GFOA01028229.1 TSA: Bactrocera bryoniae TR14889_c0_g5_i1 transcribed RNA sequence	GAGAAGGGGGCCACGCCGAGTAGGATCGAGGGTACACAGTC			
GFOA01028227.1 TSA: Bactrocera bryoniae TR14889_c0_g3_i1 transcribed RNA sequence	GAGAAGGGGGCCACGCCGAGTAGGATCGAGGGTACACAGTC			
GBYB01011647.1 TSA: Fopius arisanus c19303_g2_i1 transcribed RNA sequence	GAGAAGGGGGCCACGCCGAGTAGGATCGAGGGTACACAGTC			
GBYB01010277.1 TSA: Fopius arisanus c19303_g1_i1 transcribed RNA sequence	GAGAAGGGGGCCACGCCGAGTAGGATCGAGGGTACACAGTC			
GAKP01014524.1 TSA: Bactrocera dorsalis comp28584_c0_seq4 transcribed RNA sequence	GAGAAGGGGGCCACGCCGAGTAGGATCGAGGGTACACAGTC			
GHMS01014999.1 TSA: Reticulitermes aculabialis Unigene0015003, transcribed RNA sequence	GAATCCGAGGCCACGGCGAGTAGCATCGAGGGTACATATAG			
GHNP01099614.1 TSA: Reticulitermes labralis Unigene0099649, transcribed RNA sequence	GAATCCGAGGCCACGGCGAGTAGCATCGAGGGTACAGATTG			
APLT01015893.1 Melitaea cinxia contig15894, whole genome shotgun sequence	GAAACCGAGGCCACGGCGAGTAGCAACGAGGGTACAGTTAC			

GAUI02002458.1 TSA: Xanthostigma xanthostigma C122321_a_9_0_l_220 transcribed RNA sequence	CTGACCAGGGCCACGCCGAGTAGGATCGAGGGTACAGTCAT			
FAQD01114249.1 Laparus doris doris genome assembly Heliconius_doris_doris.CAM008684, contig 9-8684.Hd.doris_1_(paired)_contig_114249, whole genome shotgun sequence	CGGACCGCGGCCACGGCGAGTAGCATCGAGGGTACAGTCTA			
GENN01063546.1 TSA: Tineola bisselliella k74_210074 transcribed RNA sequence	CAGACCGCGGCCACGGCGAGTAGCATCGAGGGTACAGTCTG			
GCDF01025700.1 TSA: Tineola bisselliella breed wildtype s814_L_724_0_a_36_6_l_6031, transcribed RNA sequence	CAGACCGCGGCCACGGCGAGTAGCATCGAGGGTACAGTCTG			
GENN01078192.1 TSA: Tineola bisselliella k80_167110 transcribed RNA sequence	CAGACCGCGGCCACGGCGAGTAGCATCGAGGGTACAGT---			
GEOR01025605.1 TSA: Tineola bisselliella c16418_g1_i7 transcribed RNA sequence	CAGACCGCGGCCACCGCGAGTAGCATCGAGGGTACAGTCTG			
GEOR01025601.1 TSA: Tineola bisselliella c16418_g1_i3 transcribed RNA sequence	CAGACCGCGGCCACCGCGAGTAGCATCGAGGGTACAGTCTG			
GEOR01025599.1 TSA: Tineola bisselliella c16418_g1_i1 transcribed RNA sequence	CAGACCGCGGCCACCGCGAGTAGCATCGAGGGTACAGTCTG			
GDTV01025808.1 TSA: Galloisiana sinensis breed wildtype C75350_a_3_0_l_244, transcribed RNA sequence	CAACCCGAGGCCACGGCGAGTAGCATCGAGGGTACAGGTTA			
GBDP01049806.1 TSA: Kerria lacca L_5134_T_1/1_C_1.000_L_2584 transcribed RNA sequence	ATGTCGGGGGTCACGCGGAGTACGATCGAGGGTACATAAGC			
GBDO01001516.1 TSA: Kerria lacca L_1727_T_9/10_C_0.360_L_3586 transcribed RNA sequence	ATGTCGGGGGTCACGCGGAGTACGATCGAGGGTACATAAGC			
GGRZ01047477.1 TSA: Spodoptera exigua TRINITY_DN23232_c0_g1_i1, transcribed RNA sequence	ATGACCGCGGCCACGGCGAGTAGCAACGAGGGTACAGTCAT			
GARL01088459.1 TSA: Spodoptera exigua SEUC46275_TC14 transcribed RNA sequence	ATGACCGCGGCCACGGCGAGTAGCAACGAGGGTACAGTCAT			
GARL01088455.1 TSA: Spodoptera exigua SEUC46275_TC10 transcribed RNA sequence	ATGACCGCGGCCACGGCGAGTAGCAACGAGGGTACAGTCAT			
GARL01088449.1 TSA: Spodoptera exigua SEUC46275_TC04 transcribed RNA sequence	ATGACCGCGGCCACGGCGAGTAGCAACGAGGGTACAGTCAT			
GARL01088448.1 TSA: Spodoptera exigua SEUC46275_TC03 transcribed RNA sequence	ATGACCGCGGCCACGGCGAGTAGCAACGAGGGTACAGTCAT			
WNNL01000127.1 Spodoptera exigua isolate WH-S Sexi_Sc00095, whole genome shotgun sequence	ATGACCGCGGCCACGGCGAGTAGCAACGAGGGTACAGTCAT			
WNNL01000061.1 Spodoptera exigua isolate WH-S Sexi_Sc00029, whole genome shotgun sequence	ATGACCGCGGCCACGGCGAGTAGCAACGAGGGTACAGTCAT			
GDQV01132221.1 TSA: Arachnocampa luminosa Alum67714_c0_seq1 transcribed RNA sequence	ATCGCCGCGGCCACGCCGAGTAGGATCGAGGGTACAGCTAA			
GCZJ01032636.1 TSA: Unaspis euonymi breed wildtype C220814_3_0, transcribed RNA sequence	ATAACCGAGGCCACGACGAGTAGCATCGAGGGTACGGTTGT			
GDVN01022328.1 TSA: Acheta domesticus C164279_a_10_0_l_3458 transcribed RNA sequence	AGGTGGAGGCCACGCGGGGGTACGATCGAGGGTACAGCCTC			

GBDY01017035.1 TSA: Kerria lacca L_7788_T_1/1_C_1.000_L_1482 transcribed RNA sequence	AGGACGGGGGCCACGCGGAGTACGATCGAGGGTACACAATC			
GCNI01020957.1 TSA: Meroplius fasciculatus C89938_a_3_0_l_858 transcribed RNA sequence	AGAGCGGGGGCCACGCCGAGTAGGATCGAGGGTACACATCT			
GDNP01012262.1 TSA: Curculio sp. AD-2015 breed wildtype C151279_a_3_0_l_836, transcribed RNA sequence	AGAACCGAGGCCACGGCGAGTAGCAATGAGGGTACAGTCAT			
GITJ01037036.1 TSA: Busseola fusca TRINITY_DN31552_c0_g1_i1, transcribed RNA sequence	ACGACCGCGGCCACGGCGAGTAGCATCGAGGGTACAGTCGA			
GCOE01005678.1 TSA: Canaea sp. AD-2014 breed wildtype C45133_a_20_0_l_366, transcribed RNA sequence	ACGACCGCGGCCACGGCGAGCAGCATCGAGGGTACAGTCGA			
OUUW01000001.1 Drosophila guanche genome assembly, contig: dgua6_s00001, whole genome shotgun sequence	AAGTTGGGGGCCACGCCGAGTAGGATCGAGGGTACACAGAC			
BABH01079232.1 Bombyx mori DNA, contig: Bm_scaf34022_contig79232, strain: p50T/Dazao, build 2, whole genome shotgun sequence	AAGACCGCGGCCACGGCGAGTAGCATCGAGGGTACAGTCCA			
GGLW01102941.1 TSA: Arctia plantaginis Col_TRINITY_DN8019_c0_g1_i1 transcribed RNA sequence	AAGACCGCGGCCACGGCGAGTAGCATCGAGGGTACAGTCAG			
GGLW01000636.1 TSA: Arctia plantaginis Al_TRINITY_DN35235_c0_g3_i1 transcribed RNA sequence	AAGACCGCGGCCACGGCGAGTAGCATCGAGGGTACAGTCAG			
CADEBD010000286.1 Arctia plantaginis genome assembly, contig: YY_tarseq_310_arrow_ctg1, whole genome shotgun sequence	AAGACCGCGGCCACGGCGAGTAGCATCGAGGGTACAGTCAG			
CADEBD010000275.1 Arctia plantaginis genome assembly, contig: YY_tarseq_300_arrow_ctg1, whole genome shotgun sequence	AAGACCGCGGCCACGGCGAGTAGCATCGAGGGTACAGTCAG			
CADEBC010000485.1 Arctia plantaginis genome assembly, contig: WW_tarseq_472_arrow, whole genome shotgun sequence	AAGACCGCGGCCACGGCGAGTAGCATCGAGGGTACAGTCAG			
GCUR01028769.1 TSA: Alucita desmodactyla breed wildtype s9441_L_28211_0_a_32_4_l_2501, transcribed RNA sequence	AAGACCGCGGCCACGGCGAGTAGCAACGAGGGTACAGTCTT			
GATG02018062.1 TSA: Corydalus cornutus s4503_L_19020_0_a_43_2_l_9192 transcribed RNA sequence	AAAACCGAGGCCACGGCGAGTAGCATCGAGGGTACAGTTGA			
